# Supplementary material for: Predominance of the SARS-CoV-2 Lineage P.1 and Its Sublineage P.1.2 in Patients from the Metropolitan Region of Porto Alegre, Southern Brazil in March 2021
Source: Pathogens. 2021 Aug 5;10(8):988. doi: 10.3390/pathogens10080988 (PMC8402156; doi:10.3390/pathogens10080988)
Supplement: Supplementary file 1 [file pathogens-10-00988-s001.zip › TableS1.pdf]

Genomic position	Reference nt	Changed nt	Gene / region	Effect, position inside gene and amino acid replacement	Mutation count	Frequency (%)
241	C	T	5' UTR	intergenic_region n.241C>T	56	100.0
507	ATGGTCATGTT ATGGT	A	ORF1ab	disruptive_inframe_deletion c.245_259delGTCATGTTATGGTTG p.Gly82_Val86del	1	1.8
733	T	C		synonymous_variant c.468T>C p.Asp156Asp	54	96.4
775	C	T		synonymous_variant c.510C>T p.Thr170Thr	1	1.8
792	A	G		missense_variant c.527A>G p.Glu176Gly	1	1.8
843	C	T		missense_variant c.578C>T p.Pro193Leu	1	1.8
851	T	C		missense_variant c.586T>C p.Tyr196His	1	1.8
1395	A	G		missense_variant c.1130A>G p.Glu377Gly	1	1.8
1420	C	T		synonymous_variant c.1155C>T p.Ala385Ala	2	3.6
1463	G	A		missense_variant c.1198G>A p.Gly400Ser	3	5.4
1471	C	T		synonymous_variant c.1206C>T p.Arg402Arg	2	3.6
1757	G	T		missense_variant c.1492G>T p.Ala498Ser	1	1.8
1912	C	T		synonymous_variant c.1647C>T p.Ser549Ser	11	19.6
1942	G	T		synonymous_variant c.1677G>T p.Val559Val	1	1.8
2113	C	T		synonymous_variant c.1848C>T p.Ile616Ile	1	1.8
2445	C	T		missense_variant c.2180C>T p.Thr727Ile	1	1.8
2550	A	G		missense_variant c.2285A>G p.Asp762Gly	11	19.6
2749	C	T		synonymous_variant c.2484C>T p.Asp828Asp	53	94.6
2783	A	G		missense_variant c.2518A>G p.Asn840Asp	1	1.8
2890	G	T		synonymous_variant c.2625G>T p.Val875Val	1	1.8
3037	C	T		synonymous_variant c.2772C>T p.Phe924Phe	56	100.0
3096	C	T		missense_variant c.2831C>T p.Ser944Leu	1	1.8
3411	C	T		missense_variant c.3146C>T p.Ala1049Val	2	3.6

3766	T	C	synonymous_variant c.3501T>C p.Asp1167Asp	1	1.8
3783	T	C	missense_variant c.3518T>C p.Val1173Ala	1	1.8
3787	C	T	synonymous_variant c.3522C>T p.Tyr1174Tyr	1	1.8
3828	C	T	missense_variant c.3563C>T p.Ser1188Leu	54	96.4
4655	C	T	missense_variant c.4390C>T p.Arg1464Trp	1	1.8
4705	T	C	synonymous_variant c.4440T>C p.Pro1480Pro	3	5.4
4708	T	C	synonymous_variant c.4443T>C p.Asp1481Asp	1	1.8
4901	C	T	synonymous_variant c.4636C>T p.Leu1546Leu	1	1.8
5063	G	A	missense_variant c.4798G>A p.Asp1600Asn	2	3.6
5407	C	T	synonymous_variant c.5142C>T p.Ile1714Ile	1	1.8
5648	A	C	missense_variant c.5383A>C p.Lys1795Gln	48	85.7
5724	C	T	missense_variant c.5459C>T p.Thr1820Ile	10	17.9
5986	C	T	synonymous_variant c.5721C>T p.Phe1907Phe	1	1.8
6218	T	C	synonymous_variant c.5953T>C p.Leu1985Leu	1	1.8
6319	A	G	synonymous_variant c.6054A>G p.Pro2018Pro	49	87.5
6363	C	T	missense_variant c.6098C>T p.Ala2033Val	1	1.8
6525	C	T	missense_variant c.6260C>T p.Thr2087Ile	1	1.8
6576	G	T	missense_variant c.6311G>T p.Ser2104Ile	1	1.8
6613	A	G	synonymous_variant c.6348A>G p.Val2116Val	54	96.4
6840	C	T	missense_variant c.6575C>T p.Ala2192Val	1	1.8
7201	A	G	synonymous_variant c.6936A>G p.Ser2312Ser	1	1.8
8092	C	T	synonymous_variant c.7827C>T p.Leu2609Leu	1	1.8
8128	A	T	synonymous_variant c.7863A>T p.Ala2621Ala	1	1.8
8392	T	A	synonymous_variant c.8127T>A p.Ile2709Ile	2	3.6
8849	G	T	missense_variant c.8584G>T p.Val2862Leu	4	7.1
8905	C	T	synonymous_variant c.8640C>T p.Asp2880Asp	1	1.8
9042	C	T	missense_variant c.8777C>T p.Ser2926Phe	1	1.8

9052	A	G	synonymous_variant c.8787A>G p.Pro2929Pro	1	1.8
9203	G	C	missense_variant c.8938G>C p.Asp2980His	1	1.8
9329	G	A	missense_variant c.9064G>A p.Asp3022Asn	1	1.8
9532	C	T	synonymous_variant c.9267C>T p.Phe3089Phe	1	1.8
9856	G	T	synonymous_variant c.9591G>T p.Val3197Val	1	1.8
10096	G	A	synonymous_variant c.9831G>A p.Glu3277Glu	2	3.6
10138	C	T	synonymous_variant c.9873C>T p.Asn3291Asn	1	1.8
10369	C	T	synonymous_variant c.10104C>T p.Arg3368Arg	1	1.8
10507	C	T	synonymous_variant c.10242C>T p.Asn3414Asn	4	7.1
10525	C	T	synonymous_variant c.10260C>T p.Val3420Val	1	1.8
10667	T	G	missense_variant c.10402T>G p.Leu3468Val	1	1.8
10851	C	T	missense_variant c.10586C>T p.Ala3529Val	1	1.8
10885	T	C	synonymous_variant c.10620T>C p.Asn3540Asn	1	1.8
10981	G	T	synonymous_variant c.10716G>T p.Val3572Val	1	1.8
11095	C	T	synonymous_variant c.10830C>T p.Ala3610Ala	3	5.4
11287	GTCTGGTTTT	G	conservative_inframe_deletion c.11023_11031delTCTGGTTTT p.Ser3675_Phe3677del	4	7.1
11291	G	A	missense_variant c.11026G>A p.Gly3676Ser	6	10.7
11296	T	G	missense_variant c.11031T>G p.Phe3677Leu	17	30.4
11518	C	T	synonymous_variant c.11253C>T p.Val3751Val	3	5.4
11750	C	T	missense_variant c.11485C>T p.Leu3829Phe	1	1.8
11824	C	T	synonymous_variant c.11559C>T p.Ile3853Ile	1	1.8
12053	C	T	missense_variant c.11788C>T p.Leu3930Phe	2	3.6
12174	A	G	missense_variant c.11909A>G p.Asn3970Ser	1	1.8
12191	G	T	missense_variant c.11926G>T p.Val3976Phe	1	1.8
12778	C	T	synonymous_variant c.12513C>T p.Tyr4171Tyr	53	94.6
12809	C	T	missense_variant c.12544C>T p.Leu4182Phe	1	1.8

12964	A	G	synonymous_variant c.12699A>G p.Gly4233Gly	1	1.8
13059	C	T	missense_variant c.12794C>T p.Thr4265Ile	1	1.8
13176	C	T	missense_variant c.12911C>T p.Thr4304Ile	1	1.8
13459	G	T	synonymous_variant c.13194G>T p.Ser4398Ser	1	1.8
13536	C	T	missense_variant c.13271C>T p.Thr4424Ile	1	1.8
13572	T	C	missense_variant c.13307T>C p.Val4436Ala	1	1.8
13724	C	T	synonymous_variant c.13459C>T p.Leu4487Leu	1	1.8
13860	C	T	missense_variant c.13595C>T p.Thr4532Ile	53	94.6
14408	C	T	synonymous_variant c.14143C>T p.Leu4715Leu	55	98.2
15043	G	A	missense_variant c.14778G>A p.Met4926Ile	1	1.8
15082	A	G	synonymous_variant c.14817A>G p.Pro4939Pro	1	1.8
15708	G	T	stop_lost c.15443G>T p.Ter5148Leuext*?	1	1.8
15903	A	G	missense_variant c.15638A>G p.Asn5213Ser	1	1.8
16611	C	T	missense_variant c.16346C>T p.Pro5449Leu	1	1.8
16794	T	C	missense_variant c.16529T>C p.Val5510Ala	1	1.8
16887	C	T	missense_variant c.16622C>T p.Thr5541Ile	3	5.4
16954	C	T	synonymous_variant c.16689C>T p.His5563His	1	1.8
17259	G	T	missense_variant c.16994G>T p.Ser5665Ile	53	94.6
17562	G	T	missense_variant c.17297G>T p.Gly5766Val	1	1.8
17706	G	A	missense_variant c.17441G>A p.Gly5814Asp	1	1.8
17889	A	G	missense_variant c.17624A>G p.Lys5875Arg	1	1.8
18973	G	A	synonymous_variant c.18708G>A p.Arg6236Arg	1	1.8
20149	A	C	synonymous_variant c.19884A>C p.Ser6628Ser	1	1.8
20730	G	T	missense_variant c.20465G>T p.Ser6822Ile	3	5.4
20877	T	C	missense_variant c.20612T>C p.Val6871Ala	1	1.8
20931	G	A	missense_variant c.20666G>A p.Arg6889Gln	1	1.8
21519	A	C	missense_variant c.21254A>C p.Glu7085Ala	1	1.8

21614	C	T		missense_variant c.52C>T p.Leu18Phe	54	96.4
21621	C	A		missense_variant c.59C>A p.Thr20Asn	53	94.6
21638	C	T		missense_variant c.76C>T p.Pro26Ser	54	96.4
21974	G	T		missense_variant c.412G>T p.Asp138Tyr	54	96.4
22020	T	C		missense_variant c.458T>C p.Met153Thr	1	1.8
22132	G	T		missense_variant c.570G>T p.Arg190Ser	54	96.4
22336	G	T		missense_variant c.774G>T p.Trp258Cys	1	1.8
22413	C	T		missense_variant c.851C>T p.Thr284Ile	1	1.8
22487	G	A		missense_variant c.925G>A p.Glu309Lys	1	1.8
22812	A	C		missense_variant c.1250A>C p.Lys417Thr	54	96.4
23012	G	A		missense_variant c.1450G>A p.Glu484Lys	54	96.4
23063	A	T	Spike	missense_variant c.1501A>T p.Asn501Tyr	54	96.4
23403	A	G		missense_variant c.1841A>G p.Asp614Gly	56	100.0
23417	G	C		missense_variant c.1855G>C p.Glu619Gln	1	1.8
23525	C	T		missense_variant c.1963C>T p.His655Tyr	54	96.4
23628	G	T		missense_variant c.2066G>T p.Ser689Ile	1	1.8
23639	A	T		missense_variant c.2077A>T p.Ile693Phe	1	1.8
24023	C	T		synonymous_variant c.2461C>T p.Leu821Leu	1	1.8
24616	C	T		synonymous_variant c.3054C>T p.Ile1018Ile	1	1.8
24642	C	T		missense_variant c.3080C>T p.Thr1027Ile	54	96.4
25088	G	T		missense_variant c.3526G>T p.Val1176Phe	56	100.0
25158	A	G		missense_variant c.3596A>G p.Asp1199Gly	1	1.8
25294	C	T		synonymous_variant c.3732C>T p.Leu1244Leu	1	1.8
25314	G	T		missense_variant c.3752G>T p.Gly1251Val	1	1.8
25423	G	C		missense_variant c.31G>C p.Gly11Arg	1	1.8
25471	G	T	ORF3a	missense_variant c.79G>T p.Asp27Tyr	1	1.8
25483	G	A		missense_variant c.91G>A p.Ala31Thr	1	1.8

25563	G	T		missense_variant c.171G>T p.Gln57His	1	1.8
25641	G	T		missense_variant c.249G>T p.Leu83Phe	8	14.3
25658	C	T		missense_variant c.266C>T p.Thr89Ile	1	1.8
25725	A	G		synonymous_variant c.333A>G p.Leu111Leu	1	1.8
25728	C	T		synonymous_variant c.336C>T p.Val112Val	2	3.6
25771	C	T		missense_variant c.379C>T p.Leu127Phe	1	1.8
25855	G	T		missense_variant c.463G>T p.Asp155Tyr	11	19.6
26063	G	T		missense_variant c.671G>T p.Gly224Val	1	1.8
26149	T	C		missense_variant c.757T>C p.Ser253Pro	53	94.6
26149	TCCG	CCCT		stop_gained c.757_760delTCCGinsCCCT p.SerGly253*	1	1.8
26171	T	A		missense_variant c.779T>A p.Met260Lys	4	7.1
26861	T	C		synonymous_variant c.339T>C p.Asn113Asn	3	5.4
26936	C	T	Membrane	synonymous_variant c.414C>T p.Leu138Leu	1	1.8
26988	C	T		synonymous_variant c.466C>T p.Leu156Leu	1	1.8
27441	G	T		missense_variant c.48G>T p.Glu16Asp	3	5.4
27467	G	T		missense_variant c.74G>T p.Arg25Ile	1	1.8
27496	T	A	ORF7a	missense_variant c.103T>A p.Cys35Ser	1	1.8
27506	G	T		missense_variant c.113G>T p.Gly38Val	1	1.8
27513	C	T		synonymous_variant c.120C>T p.Tyr40Tyr	1	1.8
27684	C	T		synonymous_variant c.291C>T p.Tyr97Tyr	1	1.8
27982	C	T		missense_variant c.89C>T p.Pro30Leu	1	1.8
28003	G	T		missense_variant c.110G>T p.Cys37Phe	1	1.8
28167	G	A	ORF8	missense_variant c.274G>A p.Glu92Lys	53	94.6
28199	T	C		synonymous_variant c.306T>C p.Cys102Cys	1	1.8
28253	C	T		synonymous_variant c.360C>T p.Phe120Phe	2	3.6
28333	C	T		synonymous_variant c.60C>T p.Pro20Pro	1	1.8
28436	G	T	Nucleocapsid	missense_variant c.163G>T p.Ala55Ser	1	1.8

28512	C	G		missense_variant c.239C>G p.Pro80Arg	54	96.4
28603	C	T		synonymous_variant c.330C>T p.Phe110Phe	1	1.8
28628	G	T		missense_variant c.355G>T p.Ala119Ser	1	1.8
28705	T	C		synonymous_variant c.432T>C p.Asp144Asp	1	1.8
28789	C	T		synonymous_variant c.516C>T p.Tyr172Tyr	11	19.6
28806	G	T		missense_variant c.533G>T p.Gly178Val	1	1.8
28817	G	T		missense_variant c.544G>T p.Ala182Ser	1	1.8
28877	AGTAGGG	TCTAAAC		missense_variant c.604_610delAGTAGGGinsTCTAAAC p.ArgGly203LysArg	54	96.4
28881	GGG	AAC		missense_variant c.608_610delGGGinsAAC p.ArgGly203LysArg	2	3.6
28893	C	T		missense_variant c.620C>T p.Pro207Leu	1	1.8
28975	G	T		missense_variant c.702G>T p.Met234Ile	1	1.8
29095	C	T		synonymous_variant c.822C>T p.Phe274Phe	3	5.4
29160	C	T		missense_variant c.887C>T p.Thr296Ile	1	1.8
29197	C	T		synonymous_variant c.924C>T p.Ala308Ala	1	1.8
29421	C	T		missense_variant c.1148C>T p.Pro383Leu	1	1.8
29511	G	T		missense_variant c.1238G>T p.Ser413Ile	1	1.8
29688	G	T	3' UTR	intergenic_region n.29688G>T	1	1.8
29736	G	T		intergenic_region n.29736G>T	1	1.8
