# Supplementary material for: Predominance of the SARS-CoV-2 Lineage P.1 and Its Sublineage P.1.2 in Patients from the Metropolitan Region of Porto Alegre, Southern Brazil in March 2021
Source: Pathogens. 2021 Aug 5;10(8):988. doi: 10.3390/pathogens10080988 (PMC8402156; doi:10.3390/pathogens10080988)
Supplement: Supplementary file 1 [file pathogens-10-00988-s001.zip › TableS2.pdf]

We gratefully acknowledge the following Authors from the Originating laboratories responsible for obtaining the specimens, as well as the Submitting laboratories where the genome data were generated and shared via GISAID, on which this research is based.

All Submitters of data may be contacted directly via www.gisaid.org

Authors are sorted alphabetically.

Accession ID	Originating Laboratory	Submitting Laboratory	Authors
EPI_ISL_1349621	Landesgesundheitsamt Baden-Württemberg	Robert Koch Institute	?
EPI_ISL_1432849	Eurofins LifeCodexx GmbH	Robert Koch Institute	?
EPI_ISL_1349034	Bayerisches Landesamt für Gesundheit und Lebensmittelsicherheit (LGL)	Robert Koch Institute	?
EPI_ISL_1349038	Bayerisches Landesamt für Gesundheit und Lebensmittelsicherheit (LGL)	Robert Koch Institute	?
EPI_ISL_1349120	Bayerisches Landesamt für Gesundheit und Lebensmittelsicherheit (LGL)	Robert Koch Institute	?
EPI_ISL_1357204	Bayerisches Landesamt für Gesundheit und Lebensmittelsicherheit (LGL)	Robert Koch Institute	?
EPI_ISL_1570339	Bayerisches Landesamt fÃ¼r Gesundheit und Lebensmittelsicherheit (LGL)	Robert Koch Institute	?
EPI_ISL_1152591	MVZ Medizinisches Labor Hannover GmbH	Robert Koch Institute	?
EPI_ISL_1144958	MVZ Labor Krone GbR	Robert Koch Institute	?
EPI_ISL_1644947	MVZ Labor Krone GbR	Robert Koch Institute	?
EPI_ISL_1573258	Labor Prof. Dr. G. Enders MVZ GbR	Robert Koch Institute	?
EPI_ISL_1573260	Labor Prof. Dr. G. Enders MVZ GbR	Robert Koch Institute	?
EPI_ISL_1014679	National Influenza Center, Virology Department	National Influenza Center	A Nejati et al
EPI_ISL_862078	National Influenza Center, Virology Department	National Influenza Center	A Nejati et al
EPI_ISL_1014684	National Influenza Center, Virology Department	National Influenza Center	A Nejati et al
EPI_ISL_791502	The Hosptial for Sick Children	The Hospital for Sick Children	Aaron Campigotto et al
EPI_ISL_445088	Human Genetic Research Center, Kawsar Biotech Company	Human Genetic Research Center, Kawsar Biotech Company	Abbasalipour Bashash et al
EPI_ISL_1341149	Laboratory of Virology, National center of expertise	RSE National Center of Expertise and RSE National center for Biotechnology	Abdaliyev Askar et al
EPI_ISL_1341150	Laboratory of Virology, National center of expertise	RSE National Center of Expertise and RSE National center for Biotechnology	Abdaliyev Askar et al
EPI_ISL_454585	Laboratory of virology, National Center of Expertise	Laboratory of molecular-genetic research, National Center for Expertise, Kazakhstan National Center for Biotechnology, Kazakhstan	Abdaliyev Askar et al
EPI_ISL_1341503	Laboratory of Virology, National center of expertise	RSE National Center of Expertise and RSE National center for Biotechnology	Abdaliyev Askar et al
EPI_ISL_454575	Laboratory of virology, National Center of Expertise	Laboratory of molecular-genetic research, National Center for Expertise, Kazakhstan National Center for Biotechnology, Kazakhstan	Abdaliyev Askar et al
EPI_ISL_1365697	Laboratory of Virology, National center of expertise	RSE National Center of Expertise and RSE National center for Biotechnology	Abdaliyev Askar et al

EPI_ISL_454571	National Center of Expertise	National Center for Expertise, National Center for Biotechnology, Kazakhstan	Abdaliyev Askar et al
EPI_ISL_454572	National Center of Expertise	National Center for Expertise, Kazakhstan National Center for Biotechnology, Kazakhstan	Abdaliyev Askar et al
EPI_ISL_1341643	Laboratory of Virology, National center of expertise	RSE National Center of Expertise and RSE National center for Biotechnology	Abdaliyev Askar et al
EPI_ISL_1341504	Laboratory of Virology, National center of expertise	RSE National Center of Expertise and RSE National center for Biotechnology	Abdaliyev Askar et al
EPI_ISL_1341641	Laboratory of Virology, National center of expertise	RSE National Center of Expertise and RSE National center for Biotechnology	Abdaliyev Askar et al
EPI_ISL_660446	Laboratoire de Microbiologie CHU Souro Sanou	Centre Muraz	Abdoul-Salam Ouedraogo et al
EPI_ISL_660478	Laboratoire de Microbiologie CHU Souro Sanou	Centre Muraz	Abdoul-Salam Ouedraogo et al
EPI_ISL_660450	Laboratoire de Microbiologie CHU Souro Sanou	Centre Muraz	Abdoul-Salam Ouedraogo et al
EPI_ISL_660451	Laboratoire de Microbiologie CHU Souro Sanou	Centre Muraz	Abdoul-Salam Ouedraogo et al
EPI_ISL_660479	Laboratoire de Microbiologie CHU Souro Sanou	Centre Muraz	Abdoul-Salam Ouedraogo et al
EPI_ISL_660452	Laboratoire de Microbiologie CHU Souro Sanou	Centre Muraz	Abdoul-Salam Ouedraogo et al
EPI_ISL_660496	Laboratoire de Microbiologie CHU Souro Sanou	Centre Muraz	Abdoul-Salam Ouedraogo et al
EPI_ISL_660510	Laboratoire de Microbiologie CHU Souro Sanou	Centre Muraz	Abdoul-Salam Ouedraogo et al
EPI_ISL_660460	Laboratoire de Microbiologie CHU Souro Sanou	Centre Muraz	Abdoul-Salam Ouedraogo et al
EPI_ISL_660520	Laboratoire de Microbiologie CHU Souro Sanou	Centre Muraz	Abdoul-Salam Ouedraogo et al
EPI_ISL_660464	Laboratoire de Microbiologie CHU Souro Sanou	Centre Muraz	Abdoul-Salam Ouedraogo et al
EPI_ISL_660518	Laboratoire de Microbiologie CHU Souro Sanou	Centre Muraz	Abdoul-Salam Ouedraogo et al
EPI_ISL_660506	Laboratoire de Microbiologie CHU Souro Sanou	Centre Muraz	Abdoul-Salam Ouedraogo et al
EPI_ISL_660466	Laboratoire de Microbiologie CHU Souro Sanou	Centre Muraz	Abdoul-Salam Ouedraogo et al
EPI_ISL_660521	Laboratoire de Microbiologie CHU Souro Sanou	Centre Muraz	Abdoul-Salam Ouedraogo et al
EPI_ISL_660507	Laboratoire de Microbiologie CHU Souro Sanou	Centre Muraz	Abdoul-Salam Ouedraogo et al
EPI_ISL_660511	Laboratoire de Microbiologie CHU Souro Sanou	Centre Muraz	Abdoul-Salam Ouedraogo et al
EPI_ISL_660497	Laboratoire de Microbiologie CHU Souro Sanou	Centre Muraz	Abdoul-Salam Ouedraogo et al
EPI_ISL_660473	Laboratoire de Microbiologie CHU Souro Sanou	Centre Muraz	Abdoul-Salam Ouedraogo et al
EPI_ISL_660519	Laboratoire de Microbiologie CHU Souro Sanou	Centre Muraz	Abdoul-Salam Ouedraogo et al
EPI_ISL_660476	Laboratoire de Microbiologie CHU Souro Sanou	Centre Muraz	Abdoul-Salam Ouedraogo et al
EPI_ISL_660522	Laboratoire de Microbiologie CHU Souro Sanou	Centre Muraz	Abdoul-Salam Ouedraogo et al
EPI_ISL_915192	MRCG at LSHTM Genomics lab	MRCG at LSHTM Genomics lab	Abdul Karim sesay et al

EPI_ISL_1620184	MRCG at LSHTM Genomics lab	MRCG at LSHTM Genomics lab	Abdul Karim sesay et al
EPI_ISL_1620185	MRCG at LSHTM Genomics lab	MRCG at LSHTM Genomics lab	Abdul Karim sesay et al
EPI_ISL_1620177	MRCG at LSHTM Genomics lab	MRCG at LSHTM Genomics lab	Abdul Karim sesay et al
EPI_ISL_1234531	MRCG at LSHTM Genomics lab	MRCG at LSHTM Genomics lab	Abdul Karim sesay et al
EPI_ISL_1234527	MRCG at LSHTM Genomics lab	MRCG at LSHTM Genomics lab	Abdul Karim sesay et al
EPI_ISL_1234534	MRCG at LSHTM Genomics lab	MRCG at LSHTM Genomics lab	Abdul Karim sesay et al
EPI_ISL_1234535	MRCG at LSHTM Genomics lab	MRCG at LSHTM Genomics lab	Abdul Karim sesay et al
EPI_ISL_561024	MRCG at LSHTM Genomics lab	MRCG at LSHTM Genomics lab	Abdul Karim sesay et al
EPI_ISL_561026	MRCG at LSHTM Genomics lab	MRCG at LSHTM Genomics lab	Abdul Karim sesay et al
EPI_ISL_561029	MRCG at LSHTM Genomics lab	MRCG at LSHTM Genomics lab	Abdul Karim sesay et al
EPI_ISL_561033	MRCG at LSHTM Genomics lab	MRCG at LSHTM Genomics lab	Abdul Karim sesay et al
EPI_ISL_561136	MRCG at LSHTM Genomics lab	MRCG at LSHTM Genomics lab	Abdul Karim sesay et al
EPI_ISL_561088	MRCG at LSHTM Genomics lab	MRCG at LSHTM Genomics lab	Abdul Karim sesay et al
EPI_ISL_561207	MRCG at LSHTM Genomics lab	MRCG at LSHTM Genomics lab	Abdul Karim sesay et al
EPI_ISL_561212	MRCG at LSHTM Genomics lab	MRCG at LSHTM Genomics lab	Abdul Karim sesay et al
EPI_ISL_561215	MRCG at LSHTM Genomics lab	MRCG at LSHTM Genomics lab	Abdul Karim sesay et al
EPI_ISL_561216	MRCG at LSHTM Genomics lab	MRCG at LSHTM Genomics lab	Abdul Karim sesay et al
EPI_ISL_561264	MRCG at LSHTM Genomics lab	MRCG at LSHTM Genomics lab	Abdul Karim sesay et al
EPI_ISL_561302	MRCG at LSHTM Genomics lab	MRCG at LSHTM Genomics lab	Abdul Karim sesay et al
EPI_ISL_561312	MRCG at LSHTM Genomics lab	MRCG at LSHTM Genomics lab	Abdul Karim sesay et al
EPI_ISL_1216072	MRCG at LSHTM Genomics lab	MRCG at LSHTM Genomics lab	Abdul Karim sesay et al
EPI_ISL_1216076	MRCG at LSHTM Genomics lab	MRCG at LSHTM Genomics lab	Abdul Karim sesay et al
EPI_ISL_1216077	MRCG at LSHTM Genomics lab	MRCG at LSHTM Genomics lab	Abdul Karim sesay et al
EPI_ISL_1216085	MRCG at LSHTM Genomics lab	MRCG at LSHTM Genomics lab	Abdul Karim sesay et al
EPI_ISL_1216087	MRCG at LSHTM Genomics lab	MRCG at LSHTM Genomics lab	Abdul Karim sesay et al
EPI_ISL_1216119	MRCG at LSHTM Genomics lab	MRCG at LSHTM Genomics lab	Abdul Karim sesay et al
EPI_ISL_1216123	MRCG at LSHTM Genomics lab	MRCG at LSHTM Genomics lab	Abdul Karim sesay et al
EPI_ISL_1216128	MRCG at LSHTM Genomics lab	MRCG at LSHTM Genomics lab	Abdul Karim sesay et al
EPI_ISL_1216130	MRCG at LSHTM Genomics lab	MRCG at LSHTM Genomics lab	Abdul Karim sesay et al
EPI_ISL_1216133	MRCG at LSHTM Genomics lab	MRCG at LSHTM Genomics lab	Abdul Karim sesay et al

EPI_ISL_810975	MRCG at LSHTM Genomics lab	MRCG at LSHTM Genomics lab	Abdul Karim sesay et al
EPI_ISL_810974	MRCG at LSHTM Genomics lab	MRCG at LSHTM Genomics lab	Abdul Karim sesay et al
EPI_ISL_811015	MRCG at LSHTM Genomics lab	MRCG at LSHTM Genomics lab	Abdul Karim sesay et al
EPI_ISL_810976	MRCG at LSHTM Genomics lab	MRCG at LSHTM Genomics lab	Abdul Karim sesay et al
EPI_ISL_810973	MRCG at LSHTM Genomics lab	MRCG at LSHTM Genomics lab	Abdul Karim sesay et al
EPI_ISL_810984	MRCG at LSHTM Genomics lab	MRCG at LSHTM Genomics lab	Abdul Karim sesay et al
EPI_ISL_811037	MRCG at LSHTM Genomics lab	MRCG at LSHTM Genomics lab	Abdul Karim sesay et al
EPI_ISL_810982	MRCG at LSHTM Genomics lab	MRCG at LSHTM Genomics lab	Abdul Karim sesay et al
EPI_ISL_810986	MRCG at LSHTM Genomics lab	MRCG at LSHTM Genomics lab	Abdul Karim sesay et al
EPI_ISL_1532295	Oman-National Influenza Center	Biotechnology & OMICs Laboratory	Abdul Latif Khan et al
EPI_ISL_1532297	Oman-National Influenza Center	Biotechnology & OMICs Laboratory	Abdul Latif Khan et al
EPI_ISL_1532299	Oman-National Influenza Center	Biotechnology & OMICs Laboratory	Abdul Latif Khan et al
EPI_ISL_1532300	Oman-National Influenza Center	Biotechnology & OMICs Laboratory	Abdul Latif Khan et al
EPI_ISL_1532305	Oman-National Influenza Center	Biotechnology & OMICs Laboratory	Abdul Latif Khan et al
EPI_ISL_518848	Oman-National Influenza Center	Biotechnology & OMICs Laboratory, Natural & Medical Sciences Research Center, University of Nizwa	Abdul Latif Khan et al
EPI_ISL_498554	Lebanese American University	Lebanese American University	Abi Habib et al
EPI_ISL_498556	Lebanese American University	Lebanese American University	Abi Habib et al
EPI_ISL_498551	Lebanese American University	Lebanese American University	Abi Habib et al
EPI_ISL_1533610	Shiraz University	Shiraz University	Abozar Ghorbani et al
EPI_ISL_1588437	Armed Forces Institute of Pathology (AFIP), Dhaka Cantonment	Genomic Research Lab, BCSIR	Abu Sayeed Mohammad Mahmud et al
EPI_ISL_1653926	DNA Solution Ltd.	Genomic Research Lab, BCSIR	Abu Sayeed Mohammad Mahmud et al
EPI_ISL_498818	National Institute of Laboratory Medicine and Referral Center	Genomic Research Lab, BCSIR	Abu Sayeed Mohammad Mahmud et al
EPI_ISL_498830	National Institute of Laboratory Medicine and Referral Center	Genomic Research Lab, BCSIR	Abu Sayeed Mohammad Mahmud et al
EPI_ISL_803867	National Institute of Laboratory Medicine and Referral Center	Genomic Research Lab, BCSIR	Abu Sayeed Mohammad Mahmud et al
EPI_ISL_956332	Department of Biology, University of Basrah	Department of Biology, University of Basrah	Abu-Ali et al
EPI_ISL_907075	Department of Biology, University of Basrah	Department of Biology, University of Basrah	Abu-Ali et al
EPI_ISL_547445	Dutch COVID-19 response team	National Institute for Public Health and the Environment (RIVM)	Adam Meijer et al
EPI_ISL_547446	Dutch COVID-19 response team	National Institute for Public Health and the Environment (RIVM)	Adam Meijer et al
EPI_ISL_547447	Dutch COVID-19 response team	National Institute for Public Health and the Environment (RIVM)	Adam Meijer et al
EPI_ISL_547448	Dutch COVID-19 response team	National Institute for Public Health and the Environment (RIVM)	Adam Meijer et al

EPI_ISL_547449	Dutch COVID-19 response team	National Institute for Public Health and the Environment (RIVM)	Adam Meijer et al
EPI_ISL_636513	Dutch COVID-19 response team	National Institute for Public Health and the Environment (RIVM)	Adam Meijer et al
EPI_ISL_636514	Dutch COVID-19 response team	National Institute for Public Health and the Environment (RIVM)	Adam Meijer et al
EPI_ISL_636515	Dutch COVID-19 response team	National Institute for Public Health and the Environment (RIVM)	Adam Meijer et al
EPI_ISL_636516	Dutch COVID-19 response team	National Institute for Public Health and the Environment (RIVM)	Adam Meijer et al
EPI_ISL_636517	Dutch COVID-19 response team	National Institute for Public Health and the Environment (RIVM)	Adam Meijer et al
EPI_ISL_636518	Dutch COVID-19 response team	National Institute for Public Health and the Environment (RIVM)	Adam Meijer et al
EPI_ISL_1059910	Dutch COVID-19 response team	National Institute for Public Health and the Environment (RIVM)	Adam Meijer et al
EPI_ISL_1014240	Dutch COVID-19 response team	National Institute for Public Health and the Environment (RIVM)	Adam Meijer et al
EPI_ISL_1014646	Dutch COVID-19 response team	National Institute for Public Health and the Environment (RIVM)	Adam Meijer et al
EPI_ISL_1014645	Dutch COVID-19 response team	National Institute for Public Health and the Environment (RIVM)	Adam Meijer et al
EPI_ISL_1089592	Dutch COVID-19 response team	National Institute for Public Health and the Environment (RIVM)	Adam Meijer et al
EPI_ISL_1090206	Dutch COVID-19 response team	National Institute for Public Health and the Environment (RIVM)	Adam Meijer et al
EPI_ISL_1165384	Dutch COVID-19 response team	National Institute for Public Health and the Environment (RIVM)	Adam Meijer et al
EPI_ISL_1165608	Dutch COVID-19 response team	National Institute for Public Health and the Environment (RIVM)	Adam Meijer et al
EPI_ISL_1165565	Dutch COVID-19 response team	National Institute for Public Health and the Environment (RIVM)	Adam Meijer et al
EPI_ISL_1165616	Dutch COVID-19 response team	National Institute for Public Health and the Environment (RIVM)	Adam Meijer et al
EPI_ISL_1232545	Dutch COVID-19 response team	National Institute for Public Health and the Environment (RIVM)	Adam Meijer et al
EPI_ISL_1232593	Dutch COVID-19 response team	National Institute for Public Health and the Environment (RIVM)	Adam Meijer et al
EPI_ISL_1232714	Dutch COVID-19 response team	National Institute for Public Health and the Environment (RIVM)	Adam Meijer et al
EPI_ISL_1289134	Dutch COVID-19 response team	National Institute for Public Health and the Environment (RIVM)	Adam Meijer et al
EPI_ISL_1289392	Dutch COVID-19 response team	National Institute for Public Health and the Environment (RIVM)	Adam Meijer et al
EPI_ISL_1289394	Dutch COVID-19 response team	National Institute for Public Health and the Environment (RIVM)	Adam Meijer et al
EPI_ISL_1457612	Dutch COVID-19 response team	National Institute for Public Health and the Environment (RIVM)	Adam Meijer et al
EPI_ISL_1370577	Dutch COVID-19 response team	National Institute for Public Health and the Environment (RIVM)	Adam Meijer et al
EPI_ISL_1521157	Dutch COVID-19 response team	National Institute for Public Health and the Environment (RIVM)	Adam Meijer et al
EPI_ISL_1521159	Dutch COVID-19 response team	National Institute for Public Health and the Environment (RIVM)	Adam Meijer et al
EPI_ISL_1521168	Dutch COVID-19 response team	National Institute for Public Health and the Environment (RIVM)	Adam Meijer et al
EPI_ISL_1521731	Dutch COVID-19 response team	National Institute for Public Health and the Environment (RIVM)	Adam Meijer et al
EPI_ISL_1596060	Dutch COVID-19 response team	National Institute for Public Health and the Environment (RIVM)	Adam Meijer et al

EPI_ISL_1596208	Dutch COVID-19 response team	National Institute for Public Health and the Environment (RIVM)	Adam Meijer et al
EPI_ISL_1596268	Dutch COVID-19 response team	National Institute for Public Health and the Environment (RIVM)	Adam Meijer et al
EPI_ISL_1232249	Dutch COVID-19 response team	National Institute for Public Health and the Environment (RIVM)	Adam Meijer et al
EPI_ISL_1232329	Dutch COVID-19 response team	National Institute for Public Health and the Environment (RIVM)	Adam Meijer et al
EPI_ISL_1232330	Dutch COVID-19 response team	National Institute for Public Health and the Environment (RIVM)	Adam Meijer et al
EPI_ISL_1232632	Dutch COVID-19 response team	National Institute for Public Health and the Environment (RIVM)	Adam Meijer et al
EPI_ISL_1232640	Dutch COVID-19 response team	National Institute for Public Health and the Environment (RIVM)	Adam Meijer et al
EPI_ISL_1232862	Dutch COVID-19 response team	National Institute for Public Health and the Environment (RIVM)	Adam Meijer et al
EPI_ISL_1232869	Dutch COVID-19 response team	National Institute for Public Health and the Environment (RIVM)	Adam Meijer et al
EPI_ISL_1288120	Dutch COVID-19 response team	National Institute for Public Health and the Environment (RIVM)	Adam Meijer et al
EPI_ISL_1288129	Dutch COVID-19 response team	National Institute for Public Health and the Environment (RIVM)	Adam Meijer et al
EPI_ISL_1289398	Dutch COVID-19 response team	National Institute for Public Health and the Environment (RIVM)	Adam Meijer et al
EPI_ISL_1288131	Dutch COVID-19 response team	National Institute for Public Health and the Environment (RIVM)	Adam Meijer et al
EPI_ISL_1289399	Dutch COVID-19 response team	National Institute for Public Health and the Environment (RIVM)	Adam Meijer et al
EPI_ISL_1371226	Dutch COVID-19 response team	National Institute for Public Health and the Environment (RIVM)	Adam Meijer et al
EPI_ISL_1371251	Dutch COVID-19 response team	National Institute for Public Health and the Environment (RIVM)	Adam Meijer et al
EPI_ISL_1371258	Dutch COVID-19 response team	National Institute for Public Health and the Environment (RIVM)	Adam Meijer et al
EPI_ISL_1457749	Dutch COVID-19 response team	National Institute for Public Health and the Environment (RIVM)	Adam Meijer et al
EPI_ISL_1457751	Dutch COVID-19 response team	National Institute for Public Health and the Environment (RIVM)	Adam Meijer et al
EPI_ISL_1521848	Dutch COVID-19 response team	National Institute for Public Health and the Environment (RIVM)	Adam Meijer et al
EPI_ISL_1521860	Dutch COVID-19 response team	National Institute for Public Health and the Environment (RIVM)	Adam Meijer et al
EPI_ISL_1596423	Dutch COVID-19 response team	National Institute for Public Health and the Environment (RIVM)	Adam Meijer et al
EPI_ISL_1596437	Dutch COVID-19 response team	National Institute for Public Health and the Environment (RIVM)	Adam Meijer et al
EPI_ISL_547450	Dutch COVID-19 response team	National Institute for Public Health and the Environment (RIVM)	Adam Meijer et al
EPI_ISL_547451	Dutch COVID-19 response team	National Institute for Public Health and the Environment (RIVM)	Adam Meijer et al
EPI_ISL_547452	Dutch COVID-19 response team	National Institute for Public Health and the Environment (RIVM)	Adam Meijer et al
EPI_ISL_547453	Dutch COVID-19 response team	National Institute for Public Health and the Environment (RIVM)	Adam Meijer et al
EPI_ISL_636492	Dutch COVID-19 response team	National Institute for Public Health and the Environment (RIVM)	Adam Meijer et al
EPI_ISL_636493	Dutch COVID-19 response team	National Institute for Public Health and the Environment (RIVM)	Adam Meijer et al
EPI_ISL_636519	Dutch COVID-19 response team	National Institute for Public Health and the Environment (RIVM)	Adam Meijer et al

EPI_ISL_636520	Dutch COVID-19 response team	National Institute for Public Health and the Environment (RIVM)	Adam Meijer et al
EPI_ISL_636521	Dutch COVID-19 response team	National Institute for Public Health and the Environment (RIVM)	Adam Meijer et al
EPI_ISL_1014596	Dutch COVID-19 response team	National Institute for Public Health and the Environment (RIVM)	Adam Meijer et al
EPI_ISL_1014595	Dutch COVID-19 response team	National Institute for Public Health and the Environment (RIVM)	Adam Meijer et al
EPI_ISL_1014591	Dutch COVID-19 response team	National Institute for Public Health and the Environment (RIVM)	Adam Meijer et al
EPI_ISL_1014590	Dutch COVID-19 response team	National Institute for Public Health and the Environment (RIVM)	Adam Meijer et al
EPI_ISL_1014589	Dutch COVID-19 response team	National Institute for Public Health and the Environment (RIVM)	Adam Meijer et al
EPI_ISL_1014587	Dutch COVID-19 response team	National Institute for Public Health and the Environment (RIVM)	Adam Meijer et al
EPI_ISL_1014586	Dutch COVID-19 response team	National Institute for Public Health and the Environment (RIVM)	Adam Meijer et al
EPI_ISL_1014583	Dutch COVID-19 response team	National Institute for Public Health and the Environment (RIVM)	Adam Meijer et al
EPI_ISL_1035771	Dutch COVID-19 response team	National Institute for Public Health and the Environment (RIVM)	Adam Meijer et al
EPI_ISL_1035770	Dutch COVID-19 response team	National Institute for Public Health and the Environment (RIVM)	Adam Meijer et al
EPI_ISL_1035767	Dutch COVID-19 response team	National Institute for Public Health and the Environment (RIVM)	Adam Meijer et al
EPI_ISL_1035766	Dutch COVID-19 response team	National Institute for Public Health and the Environment (RIVM)	Adam Meijer et al
EPI_ISL_1232248	Dutch COVID-19 response team	National Institute for Public Health and the Environment (RIVM)	Adam Meijer et al
EPI_ISL_1232269	Dutch COVID-19 response team	National Institute for Public Health and the Environment (RIVM)	Adam Meijer et al
EPI_ISL_1232282	Dutch COVID-19 response team	National Institute for Public Health and the Environment (RIVM)	Adam Meijer et al
EPI_ISL_1232290	Dutch COVID-19 response team	National Institute for Public Health and the Environment (RIVM)	Adam Meijer et al
EPI_ISL_1232292	Dutch COVID-19 response team	National Institute for Public Health and the Environment (RIVM)	Adam Meijer et al
EPI_ISL_1232326	Dutch COVID-19 response team	National Institute for Public Health and the Environment (RIVM)	Adam Meijer et al
EPI_ISL_1232327	Dutch COVID-19 response team	National Institute for Public Health and the Environment (RIVM)	Adam Meijer et al
EPI_ISL_1232328	Dutch COVID-19 response team	National Institute for Public Health and the Environment (RIVM)	Adam Meijer et al
EPI_ISL_1233007	Dutch COVID-19 response team	National Institute for Public Health and the Environment (RIVM)	Adam Meijer et al
EPI_ISL_1288141	Dutch COVID-19 response team	National Institute for Public Health and the Environment (RIVM)	Adam Meijer et al
EPI_ISL_1288143	Dutch COVID-19 response team	National Institute for Public Health and the Environment (RIVM)	Adam Meijer et al
EPI_ISL_1288144	Dutch COVID-19 response team	National Institute for Public Health and the Environment (RIVM)	Adam Meijer et al
EPI_ISL_1288149	Dutch COVID-19 response team	National Institute for Public Health and the Environment (RIVM)	Adam Meijer et al
EPI_ISL_1370641	Dutch COVID-19 response team	National Institute for Public Health and the Environment (RIVM)	Adam Meijer et al
EPI_ISL_1369778	Dutch COVID-19 response team	National Institute for Public Health and the Environment (RIVM)	Adam Meijer et al
EPI_ISL_1370667	Dutch COVID-19 response team	National Institute for Public Health and the Environment (RIVM)	Adam Meijer et al

EPI_ISL_1369786	Dutch COVID-19 response team	National Institute for Public Health and the Environment (RIVM)	Adam Meijer et al
EPI_ISL_1457573	Dutch COVID-19 response team	National Institute for Public Health and the Environment (RIVM)	Adam Meijer et al
EPI_ISL_1457641	Dutch COVID-19 response team	National Institute for Public Health and the Environment (RIVM)	Adam Meijer et al
EPI_ISL_1521490	Dutch COVID-19 response team	National Institute for Public Health and the Environment (RIVM)	Adam Meijer et al
EPI_ISL_1014579	Dutch COVID-19 response team	National Institute for Public Health and the Environment (RIVM)	Adam Meijer et al
EPI_ISL_1014578	Dutch COVID-19 response team	National Institute for Public Health and the Environment (RIVM)	Adam Meijer et al
EPI_ISL_1014577	Dutch COVID-19 response team	National Institute for Public Health and the Environment (RIVM)	Adam Meijer et al
EPI_ISL_1014576	Dutch COVID-19 response team	National Institute for Public Health and the Environment (RIVM)	Adam Meijer et al
EPI_ISL_1014575	Dutch COVID-19 response team	National Institute for Public Health and the Environment (RIVM)	Adam Meijer et al
EPI_ISL_1014574	Dutch COVID-19 response team	National Institute for Public Health and the Environment (RIVM)	Adam Meijer et al
EPI_ISL_1014573	Dutch COVID-19 response team	National Institute for Public Health and the Environment (RIVM)	Adam Meijer et al
EPI_ISL_1014572	Dutch COVID-19 response team	National Institute for Public Health and the Environment (RIVM)	Adam Meijer et al
EPI_ISL_1014570	Dutch COVID-19 response team	National Institute for Public Health and the Environment (RIVM)	Adam Meijer et al
EPI_ISL_1014569	Dutch COVID-19 response team	National Institute for Public Health and the Environment (RIVM)	Adam Meijer et al
EPI_ISL_1014568	Dutch COVID-19 response team	National Institute for Public Health and the Environment (RIVM)	Adam Meijer et al
EPI_ISL_1014567	Dutch COVID-19 response team	National Institute for Public Health and the Environment (RIVM)	Adam Meijer et al
EPI_ISL_1014566	Dutch COVID-19 response team	National Institute for Public Health and the Environment (RIVM)	Adam Meijer et al
EPI_ISL_1014564	Dutch COVID-19 response team	National Institute for Public Health and the Environment (RIVM)	Adam Meijer et al
EPI_ISL_1014563	Dutch COVID-19 response team	National Institute for Public Health and the Environment (RIVM)	Adam Meijer et al
EPI_ISL_1014562	Dutch COVID-19 response team	National Institute for Public Health and the Environment (RIVM)	Adam Meijer et al
EPI_ISL_1014560	Dutch COVID-19 response team	National Institute for Public Health and the Environment (RIVM)	Adam Meijer et al
EPI_ISL_1014559	Dutch COVID-19 response team	National Institute for Public Health and the Environment (RIVM)	Adam Meijer et al
EPI_ISL_1014557	Dutch COVID-19 response team	National Institute for Public Health and the Environment (RIVM)	Adam Meijer et al
EPI_ISL_1014556	Dutch COVID-19 response team	National Institute for Public Health and the Environment (RIVM)	Adam Meijer et al
EPI_ISL_1014555	Dutch COVID-19 response team	National Institute for Public Health and the Environment (RIVM)	Adam Meijer et al
EPI_ISL_1014554	Dutch COVID-19 response team	National Institute for Public Health and the Environment (RIVM)	Adam Meijer et al
EPI_ISL_1014553	Dutch COVID-19 response team	National Institute for Public Health and the Environment (RIVM)	Adam Meijer et al
EPI_ISL_1014552	Dutch COVID-19 response team	National Institute for Public Health and the Environment (RIVM)	Adam Meijer et al
EPI_ISL_1014551	Dutch COVID-19 response team	National Institute for Public Health and the Environment (RIVM)	Adam Meijer et al
EPI_ISL_1014549	Dutch COVID-19 response team	National Institute for Public Health and the Environment (RIVM)	Adam Meijer et al

EPI_ISL_1596269	Dutch COVID-19 response team	National Institute for Public Health and the Environment (RIVM)	Adam Meijer et al
EPI_ISL_1596270	Dutch COVID-19 response team	National Institute for Public Health and the Environment (RIVM)	Adam Meijer et al
EPI_ISL_1289218	Dutch COVID-19 response team	National Institute for Public Health and the Environment (RIVM)	Adam Meijer et al
EPI_ISL_1456646	Dutch COVID-19 response team	National Institute for Public Health and the Environment (RIVM)	Adam Meijer et al
EPI_ISL_1456652	Dutch COVID-19 response team	National Institute for Public Health and the Environment (RIVM)	Adam Meijer et al
EPI_ISL_1521313	Dutch COVID-19 response team	National Institute for Public Health and the Environment (RIVM)	Adam Meijer et al
EPI_ISL_1596573	Dutch COVID-19 response team	National Institute for Public Health and the Environment (RIVM)	Adam Meijer et al
EPI_ISL_547478	Dutch COVID-19 response team	National Institute for Public Health and the Environment (RIVM)	Adam Meijer et al
EPI_ISL_1035663	Dutch COVID-19 response team	National Institute for Public Health and the Environment (RIVM)	Adam Meijer et al
EPI_ISL_1597267	Dutch COVID-19 response team	National Institute for Public Health and the Environment (RIVM)	Adam Meijer et al
EPI_ISL_1090134	Dutch COVID-19 response team	National Institute for Public Health and the Environment (RIVM)	Adam Meijer et al
EPI_ISL_905383	Dutch COVID-19 response team	National Institute for Public Health and the Environment (RIVM)	Adam Meijer et al
EPI_ISL_1596936	Dutch COVID-19 response team	National Institute for Public Health and the Environment (RIVM)	Adam Meijer et al
EPI_ISL_547499	Dutch COVID-19 response team	National Institute for Public Health and the Environment (RIVM)	Adam Meijer et al
EPI_ISL_1014436	Dutch COVID-19 response team	National Institute for Public Health and the Environment (RIVM)	Adam Meijer et al
EPI_ISL_1014435	Dutch COVID-19 response team	National Institute for Public Health and the Environment (RIVM)	Adam Meijer et al
EPI_ISL_454750	Dutch COVID-19 response team	National Institute for Public Health and the Environment (RIVM)	Adam Meijer et al
EPI_ISL_454751	Dutch COVID-19 response team	National Institute for Public Health and the Environment (RIVM)	Adam Meijer et al
EPI_ISL_1014104	Dutch COVID-19 response team	National Institute for Public Health and the Environment (RIVM)	Adam Meijer et al
EPI_ISL_1035443	Dutch COVID-19 response team	National Institute for Public Health and the Environment (RIVM)	Adam Meijer et al
EPI_ISL_1035246	Dutch COVID-19 response team	National Institute for Public Health and the Environment (RIVM)	Adam Meijer et al
EPI_ISL_1035244	Dutch COVID-19 response team	National Institute for Public Health and the Environment (RIVM)	Adam Meijer et al
EPI_ISL_1035243	Dutch COVID-19 response team	National Institute for Public Health and the Environment (RIVM)	Adam Meijer et al
EPI_ISL_1035242	Dutch COVID-19 response team	National Institute for Public Health and the Environment (RIVM)	Adam Meijer et al
EPI_ISL_1035240	Dutch COVID-19 response team	National Institute for Public Health and the Environment (RIVM)	Adam Meijer et al
EPI_ISL_1035239	Dutch COVID-19 response team	National Institute for Public Health and the Environment (RIVM)	Adam Meijer et al
EPI_ISL_1089591	Dutch COVID-19 response team	National Institute for Public Health and the Environment (RIVM)	Adam Meijer et al
EPI_ISL_1595963	Dutch COVID-19 response team	National Institute for Public Health and the Environment (RIVM)	Adam Meijer et al
EPI_ISL_1595971	Dutch COVID-19 response team	National Institute for Public Health and the Environment (RIVM)	Adam Meijer et al
EPI_ISL_1595972	Dutch COVID-19 response team	National Institute for Public Health and the Environment (RIVM)	Adam Meijer et al

EPI_ISL_1595982	Dutch COVID-19 response team	National Institute for Public Health and the Environment (RIVM)	Adam Meijer et al
EPI_ISL_1595983	Dutch COVID-19 response team	National Institute for Public Health and the Environment (RIVM)	Adam Meijer et al
EPI_ISL_1596056	Dutch COVID-19 response team	National Institute for Public Health and the Environment (RIVM)	Adam Meijer et al
EPI_ISL_1596057	Dutch COVID-19 response team	National Institute for Public Health and the Environment (RIVM)	Adam Meijer et al
EPI_ISL_1596078	Dutch COVID-19 response team	National Institute for Public Health and the Environment (RIVM)	Adam Meijer et al
EPI_ISL_1596090	Dutch COVID-19 response team	National Institute for Public Health and the Environment (RIVM)	Adam Meijer et al
EPI_ISL_1522177	Dutch COVID-19 response team	National Institute for Public Health and the Environment (RIVM)	Adam Meijer et al
EPI_ISL_1522184	Dutch COVID-19 response team	National Institute for Public Health and the Environment (RIVM)	Adam Meijer et al
EPI_ISL_1522185	Dutch COVID-19 response team	National Institute for Public Health and the Environment (RIVM)	Adam Meijer et al
EPI_ISL_1522186	Dutch COVID-19 response team	National Institute for Public Health and the Environment (RIVM)	Adam Meijer et al
EPI_ISL_1522189	Dutch COVID-19 response team	National Institute for Public Health and the Environment (RIVM)	Adam Meijer et al
EPI_ISL_1521324	Dutch COVID-19 response team	National Institute for Public Health and the Environment (RIVM)	Adam Meijer et al
EPI_ISL_1522195	Dutch COVID-19 response team	National Institute for Public Health and the Environment (RIVM)	Adam Meijer et al
EPI_ISL_1522200	Dutch COVID-19 response team	National Institute for Public Health and the Environment (RIVM)	Adam Meijer et al
EPI_ISL_1522201	Dutch COVID-19 response team	National Institute for Public Health and the Environment (RIVM)	Adam Meijer et al
EPI_ISL_1522203	Dutch COVID-19 response team	National Institute for Public Health and the Environment (RIVM)	Adam Meijer et al
EPI_ISL_1522205	Dutch COVID-19 response team	National Institute for Public Health and the Environment (RIVM)	Adam Meijer et al
EPI_ISL_1522208	Dutch COVID-19 response team	National Institute for Public Health and the Environment (RIVM)	Adam Meijer et al
EPI_ISL_1522211	Dutch COVID-19 response team	National Institute for Public Health and the Environment (RIVM)	Adam Meijer et al
EPI_ISL_1522213	Dutch COVID-19 response team	National Institute for Public Health and the Environment (RIVM)	Adam Meijer et al
EPI_ISL_833494	RS PMI Bogor, Indonesia	Biosafety Level-3 Laboratory, Indonesian Institute of Sciences (LIPI)	Ade Andriani et al
EPI_ISL_977171	Microbiologia e Virologia	Istituto Zooprofilattico Sperimentale delle Venezie	Adelaide Milani et al
EPI_ISL_977172	Microbiologia e Virologia	Istituto Zooprofilattico Sperimentale delle Venezie	Adelaide Milani et al
EPI_ISL_875556	ULSS 2 Treviso	Istituto Zooprofilattico Sperimentale delle Venezie	Adelaide Milani et al
EPI_ISL_875553	ULSS 2 Treviso	Istituto Zooprofilattico Sperimentale delle Venezie	Adelaide Milani et al
EPI_ISL_875554	ULSS 2 Treviso	Istituto Zooprofilattico Sperimentale delle Venezie	Adelaide Milani et al
EPI_ISL_875555	ULSS 2 Treviso	Istituto Zooprofilattico Sperimentale delle Venezie	Adelaide Milani et al
EPI_ISL_1260963	ULSS 04 Veneto Orientale	Istituto Zooprofilattico Sperimentale delle Venezie	Adelaide Milani et al
EPI_ISL_424673	Instituto de Diagnostico y Referencia Epidemiologicos	Instituto de Diagnostico y Referencia Epidemiologicos	Adnan Araiza Rodríguez et al
EPI_ISL_1018088	Immunology, Noguchi Memorial Institute for Medical Research	Immunology, Noguchi Memorial Institute for Medical Research	Adu et al

EPI_ISL_1018089	Immunology, Noguchi Memorial Institute for Medical Research	Immunology, Noguchi Memorial Institute for Medical Research	Adu et al
EPI_ISL_1018091	Immunology, Noguchi Memorial Institute for Medical Research	Immunology, Noguchi Memorial Institute for Medical Research	Adu et al
EPI_ISL_1018087	Immunology, Noguchi Memorial Institute for Medical Research	Immunology, Noguchi Memorial Institute for Medical Research	Adu et al
EPI_ISL_747304	Division of Emerging Infectious Diseases, Bureau of Infectious Diseases Diagnosis Control, Korea Disease Control and Prevention Agency	Division of Emerging Infectious Diseases, Bureau of Infectious Diseases Diagnosis Control, Korea Disease Control and Prevention Agency	Ae Kyung Park et al
EPI_ISL_747307	Division of Emerging Infectious Diseases, Bureau of Infectious Diseases Diagnosis Control, Korea Disease Control and Prevention Agency	Division of Emerging Infectious Diseases, Bureau of Infectious Diseases Diagnosis Control, Korea Disease Control and Prevention Agency	Ae Kyung Park et al
EPI_ISL_747318	Division of Emerging Infectious Diseases, Bureau of Infectious Diseases Diagnosis Control, Korea Disease Control and Prevention Agency	Division of Emerging Infectious Diseases, Bureau of Infectious Diseases Diagnosis Control, Korea Disease Control and Prevention Agency	Ae Kyung Park et al
EPI_ISL_747333	Division of Emerging Infectious Diseases, Bureau of Infectious Diseases Diagnosis Control, Korea Disease Control and Prevention Agency	Division of Emerging Infectious Diseases, Bureau of Infectious Diseases Diagnosis Control, Korea Disease Control and Prevention Agency	Ae Kyung Park et al
EPI_ISL_747337	Division of Emerging Infectious Diseases, Bureau of Infectious Diseases Diagnosis Control, Korea Disease Control and Prevention Agency	Division of Emerging Infectious Diseases, Bureau of Infectious Diseases Diagnosis Control, Korea Disease Control and Prevention Agency	Ae Kyung Park et al
EPI_ISL_747342	Division of Emerging Infectious Diseases, Bureau of Infectious Diseases Diagnosis Control, Korea Disease Control and Prevention Agency	Division of Emerging Infectious Diseases, Bureau of Infectious Diseases Diagnosis Control, Korea Disease Control and Prevention Agency	Ae Kyung Park et al
EPI_ISL_747362	Division of Emerging Infectious Diseases, Bureau of Infectious Diseases Diagnosis Control, Korea Disease Control and Prevention Agency	Division of Emerging Infectious Diseases, Bureau of Infectious Diseases Diagnosis Control, Korea Disease Control and Prevention Agency	Ae Kyung Park et al
EPI_ISL_747449	Division of Emerging Infectious Diseases, Bureau of Infectious Diseases Diagnosis Control, Korea Disease Control and Prevention Agency	Division of Emerging Infectious Diseases, Bureau of Infectious Diseases Diagnosis Control, Korea Disease Control and Prevention Agency	Ae Kyung Park et al
EPI_ISL_760102	Division of Emerging Infectious Diseases, Bureau of Infectious Diseases Diagnosis Control, Korea Disease Control and Prevention Agency	Division of Emerging Infectious Diseases, Bureau of Infectious Diseases Diagnosis Control, Korea Disease Control and Prevention Agency	Ae Kyung Park et al
EPI_ISL_760103	Division of Emerging Infectious Diseases, Bureau of Infectious Diseases Diagnosis Control, Korea Disease Control and Prevention Agency	Division of Emerging Infectious Diseases, Bureau of Infectious Diseases Diagnosis Control, Korea Disease Control and Prevention Agency	Ae Kyung Park et al
EPI_ISL_833249	Division of Emerging Infectious Diseases, Bureau of Infectious Diseases Diagnosis Control, Korea Disease Control and Prevention Agency	Division of Emerging Infectious Diseases, Bureau of Infectious Diseases Diagnosis Control, Korea Disease Control and Prevention Agency	Ae Kyung Park et al
EPI_ISL_850198	Division of Emerging Infectious Diseases, Bureau of Infectious Diseases Diagnosis Control, Korea Disease Control and Prevention Agency	Division of Emerging Infectious Diseases, Bureau of Infectious Diseases Diagnosis Control, Korea Disease Control and Prevention Agency	Ae Kyung Park et al
EPI_ISL_850200	Division of Emerging Infectious Diseases, Bureau of Infectious Diseases Diagnosis Control, Korea Disease Control and Prevention Agency	Division of Emerging Infectious Diseases, Bureau of Infectious Diseases Diagnosis Control, Korea Disease Control and Prevention Agency	Ae Kyung Park et al
EPI_ISL_850211	Division of Emerging Infectious Diseases, Bureau of Infectious Diseases Diagnosis Control, Korea Disease Control and Prevention Agency	Division of Emerging Infectious Diseases, Bureau of Infectious Diseases Diagnosis Control, Korea Disease Control and Prevention Agency	Ae Kyung Park et al
EPI_ISL_850215	Division of Emerging Infectious Diseases, Bureau of Infectious Diseases Diagnosis Control, Korea Disease Control and Prevention Agency	Division of Emerging Infectious Diseases, Bureau of Infectious Diseases Diagnosis Control, Korea Disease Control and Prevention Agency	Ae Kyung Park et al
EPI_ISL_850391	Division of Emerging Infectious Diseases, Bureau of Infectious Diseases Diagnosis Control, Korea Disease Control and Prevention Agency	Division of Emerging Infectious Diseases, Bureau of Infectious Diseases Diagnosis Control, Korea Disease Control and Prevention Agency	Ae Kyung Park et al
EPI_ISL_995230	Division of Emerging Infectious Diseases, Bureau of Infectious Diseases Diagnosis Control, Korea Disease Control and Prevention Agency	Division of Emerging Infectious Diseases, Bureau of Infectious Diseases Diagnosis Control, Korea Disease Control and Prevention Agency	Ae Kyung Park et al
EPI_ISL_1063647	Division of Emerging Infectious Diseases, Bureau of Infectious Diseases Diagnosis Control, Korea Disease Control and Prevention Agency	Division of Emerging Infectious Diseases, Bureau of Infectious Diseases Diagnosis Control, Korea Disease Control and Prevention Agency	Ae Kyung Park et al
EPI_ISL_1063656	Division of Emerging Infectious Diseases, Bureau of Infectious Diseases Diagnosis Control, Korea Disease Control and Prevention Agency	Division of Emerging Infectious Diseases, Bureau of Infectious Diseases Diagnosis Control, Korea Disease Control and Prevention Agency	Ae Kyung Park et al
EPI_ISL_1063614	Division of Emerging Infectious Diseases, Bureau of Infectious Diseases Diagnosis Control, Korea Disease Control and Prevention Agency	Division of Emerging Infectious Diseases, Bureau of Infectious Diseases Diagnosis Control, Korea Disease Control and Prevention Agency	Ae Kyung Park et al

EPI_ISL_1063615	Division of Emerging Infectious Diseases, Bureau of Infectious Diseases Diagnosis Control, Korea Disease Control and Prevention Agency	Division of Emerging Infectious Diseases, Bureau of Infectious Diseases Diagnosis Control, Korea Disease Control and Prevention Agency	Ae Kyung Park et al
EPI_ISL_1063563	Division of Emerging Infectious Diseases, Bureau of Infectious Diseases Diagnosis Control, Korea Disease Control and Prevention Agency	Division of Emerging Infectious Diseases, Bureau of Infectious Diseases Diagnosis Control, Korea Disease Control and Prevention Agency	Ae Kyung Park et al
EPI_ISL_1063589	Division of Emerging Infectious Diseases, Bureau of Infectious Diseases Diagnosis Control, Korea Disease Control and Prevention Agency	Division of Emerging Infectious Diseases, Bureau of Infectious Diseases Diagnosis Control, Korea Disease Control and Prevention Agency	Ae Kyung Park et al
EPI_ISL_1063559	Division of Emerging Infectious Diseases, Bureau of Infectious Diseases Diagnosis Control, Korea Disease Control and Prevention Agency	Division of Emerging Infectious Diseases, Bureau of Infectious Diseases Diagnosis Control, Korea Disease Control and Prevention Agency	Ae Kyung Park et al
EPI_ISL_1063554	Division of Emerging Infectious Diseases, Bureau of Infectious Diseases Diagnosis Control, Korea Disease Control and Prevention Agency	Division of Emerging Infectious Diseases, Bureau of Infectious Diseases Diagnosis Control, Korea Disease Control and Prevention Agency	Ae Kyung Park et al
EPI_ISL_1063558	Division of Emerging Infectious Diseases, Bureau of Infectious Diseases Diagnosis Control, Korea Disease Control and Prevention Agency	Division of Emerging Infectious Diseases, Bureau of Infectious Diseases Diagnosis Control, Korea Disease Control and Prevention Agency	Ae Kyung Park et al
EPI_ISL_1063556	Division of Emerging Infectious Diseases, Bureau of Infectious Diseases Diagnosis Control, Korea Disease Control and Prevention Agency	Division of Emerging Infectious Diseases, Bureau of Infectious Diseases Diagnosis Control, Korea Disease Control and Prevention Agency	Ae Kyung Park et al
EPI_ISL_1063616	Division of Emerging Infectious Diseases, Bureau of Infectious Diseases Diagnosis Control, Korea Disease Control and Prevention Agency	Division of Emerging Infectious Diseases, Bureau of Infectious Diseases Diagnosis Control, Korea Disease Control and Prevention Agency	Ae Kyung Park et al
EPI_ISL_1063678	Division of Emerging Infectious Diseases, Bureau of Infectious Diseases Diagnosis Control, Korea Disease Control and Prevention Agency	Division of Emerging Infectious Diseases, Bureau of Infectious Diseases Diagnosis Control, Korea Disease Control and Prevention Agency	Ae Kyung Park et al
EPI_ISL_1063600	Division of Emerging Infectious Diseases, Bureau of Infectious Diseases Diagnosis Control, Korea Disease Control and Prevention Agency	Division of Emerging Infectious Diseases, Bureau of Infectious Diseases Diagnosis Control, Korea Disease Control and Prevention Agency	Ae Kyung Park et al
EPI_ISL_1063702	Division of Emerging Infectious Diseases, Bureau of Infectious Diseases Diagnosis Control, Korea Disease Control and Prevention Agency	Division of Emerging Infectious Diseases, Bureau of Infectious Diseases Diagnosis Control, Korea Disease Control and Prevention Agency	Ae Kyung Park et al
EPI_ISL_1063599	Division of Emerging Infectious Diseases, Bureau of Infectious Diseases Diagnosis Control, Korea Disease Control and Prevention Agency	Division of Emerging Infectious Diseases, Bureau of Infectious Diseases Diagnosis Control, Korea Disease Control and Prevention Agency	Ae Kyung Park et al
EPI_ISL_1063601	Division of Emerging Infectious Diseases, Bureau of Infectious Diseases Diagnosis Control, Korea Disease Control and Prevention Agency	Division of Emerging Infectious Diseases, Bureau of Infectious Diseases Diagnosis Control, Korea Disease Control and Prevention Agency	Ae Kyung Park et al
EPI_ISL_1063570	Division of Emerging Infectious Diseases, Bureau of Infectious Diseases Diagnosis Control, Korea Disease Control and Prevention Agency	Division of Emerging Infectious Diseases, Bureau of Infectious Diseases Diagnosis Control, Korea Disease Control and Prevention Agency	Ae Kyung Park et al
EPI_ISL_1165001	Division of Emerging Infectious Diseases, Bureau of Infectious Diseases Diagnosis Control, Korea Disease Control and Prevention Agency	Division of Emerging Infectious Diseases, Bureau of Infectious Diseases Diagnosis Control, Korea Disease Control and Prevention Agency	Ae Kyung Park et al
EPI_ISL_1165058	Division of Emerging Infectious Diseases, Bureau of Infectious Diseases Diagnosis Control, Korea Disease Control and Prevention Agency	Division of Emerging Infectious Diseases, Bureau of Infectious Diseases Diagnosis Control, Korea Disease Control and Prevention Agency	Ae Kyung Park et al
EPI_ISL_1209415	Division of Emerging Infectious Diseases, Bureau of Infectious Diseases Diagnosis Control, Korea Disease Control and Prevention Agency	Division of Emerging Infectious Diseases, Bureau of Infectious Diseases Diagnosis Control, Korea Disease Control and Prevention Agency	Ae Kyung Park et al
EPI_ISL_1490007	Division of Emerging Infectious Diseases, Bureau of Infectious Diseases Diagnosis Control, Korea Disease Control and Prevention Agency	Division of Emerging Infectious Diseases, Bureau of Infectious Diseases Diagnosis Control, Korea Disease Control and Prevention Agency	Ae Kyung Park et al
EPI_ISL_1587522	Division of Emerging Infectious Diseases, Bureau of Infectious Diseases Diagnosis Control, Korea Disease Control and Prevention Agency	Division of Emerging Infectious Diseases, Bureau of Infectious Diseases Diagnosis Control, Korea Disease Control and Prevention Agency	Ae Kyung Park et al
EPI_ISL_1587525	Division of Emerging Infectious Diseases, Bureau of Infectious Diseases Diagnosis Control, Korea Disease Control and Prevention Agency	Division of Emerging Infectious Diseases, Bureau of Infectious Diseases Diagnosis Control, Korea Disease Control and Prevention Agency	Ae Kyung Park et al
EPI_ISL_1587547	Division of Emerging Infectious Diseases, Bureau of Infectious Diseases Diagnosis Control, Korea Disease Control and Prevention Agency	Division of Emerging Infectious Diseases, Bureau of Infectious Diseases Diagnosis Control, Korea Disease Control and Prevention Agency	Ae Kyung Park et al
EPI_ISL_1587550	Division of Emerging Infectious Diseases, Bureau of Infectious Diseases Diagnosis Control, Korea Disease Control and Prevention Agency	Division of Emerging Infectious Diseases, Bureau of Infectious Diseases Diagnosis Control, Korea Disease Control and Prevention Agency	Ae Kyung Park et al

EPI_ISL_1622483	Division of Emerging Infectious Diseases, Bureau of Infectious Diseases Diagnosis Control, Korea Disease Control and Prevention Agency	Division of Emerging Infectious Diseases, Bureau of Infectious Diseases Diagnosis Control, Korea Disease Control and Prevention Agency	Ae Kyung Park et al
EPI_ISL_1622487	Division of Emerging Infectious Diseases, Bureau of Infectious Diseases Diagnosis Control, Korea Disease Control and Prevention Agency	Division of Emerging Infectious Diseases, Bureau of Infectious Diseases Diagnosis Control, Korea Disease Control and Prevention Agency	Ae Kyung Park et al
EPI_ISL_1622497	Division of Emerging Infectious Diseases, Bureau of Infectious Diseases Diagnosis Control, Korea Disease Control and Prevention Agency	Division of Emerging Infectious Diseases, Bureau of Infectious Diseases Diagnosis Control, Korea Disease Control and Prevention Agency	Ae Kyung Park et al
EPI_ISL_1622498	Division of Emerging Infectious Diseases, Bureau of Infectious Diseases Diagnosis Control, Korea Disease Control and Prevention Agency	Division of Emerging Infectious Diseases, Bureau of Infectious Diseases Diagnosis Control, Korea Disease Control and Prevention Agency	Ae Kyung Park et al
EPI_ISL_1622499	Division of Emerging Infectious Diseases, Bureau of Infectious Diseases Diagnosis Control, Korea Disease Control and Prevention Agency	Division of Emerging Infectious Diseases, Bureau of Infectious Diseases Diagnosis Control, Korea Disease Control and Prevention Agency	Ae Kyung Park et al
EPI_ISL_1647347	Division of Emerging Infectious Diseases, Bureau of Infectious Diseases Diagnosis Control, Korea Disease Control and Prevention Agency	Division of Emerging Infectious Diseases, Bureau of Infectious Diseases Diagnosis Control, Korea Disease Control and Prevention Agency	Ae Kyung Park et al
EPI_ISL_1647350	Division of Emerging Infectious Diseases, Bureau of Infectious Diseases Diagnosis Control, Korea Disease Control and Prevention Agency	Division of Emerging Infectious Diseases, Bureau of Infectious Diseases Diagnosis Control, Korea Disease Control and Prevention Agency	Ae Kyung Park et al
EPI_ISL_513008	Pathogen Genomics Lab King Abdullah University of Science and Technology (KAUST)	Pathogen Genomics Lab King Abdullah University of Science and Technology(KAUST)	Afrah Alsomali et al
EPI_ISL_513021	Pathogen Genomics Lab King Abdullah University of Science and Technology (KAUST)	Pathogen Genomics Lab King Abdullah University of Science and Technology(KAUST)	Afrah Alsomali et al
EPI_ISL_513192	Pathogen Genomics Lab King Abdullah University of Science and Technology (KAUST)	Pathogen Genomics Lab King Abdullah University of Science and Technology(KAUST)	Afrah Alsomali et al
EPI_ISL_882637	Azerbaijan National Hematology Center Division of Medical Genetics	Azerbaijan National Hematology Center Division of Medical Genetics	Aghayev Agha Rza et al
EPI_ISL_882639	Azerbaijan National Hematology Center Division of Medical Genetics	Azerbaijan National Hematology Center Division of Medical Genetics	Aghayev Agha Rza et al
EPI_ISL_882641	Azerbaijan National Hematology Center Division of Medical Genetics	Azerbaijan National Hematology Center Division of Medical Genetics	Aghayev Agha Rza et al
EPI_ISL_833130	National Institute of Health Research and Development	National Institute of Health Research and Development	Agustiningsih et al
EPI_ISL_1517192	Oman-National Influenza Center	Biotechnology & OMICs Laboratory	Ahmed Al Harrasi et al
EPI_ISL_1532284	Oman-National Influenza Center	Biotechnology & OMICs Laboratory	Ahmed Al Harrasi et al
EPI_ISL_1532285	Oman-National Influenza Center	Biotechnology & OMICs Laboratory	Ahmed Al Harrasi et al
EPI_ISL_1532301	Oman-National Influenza Center	Biotechnology & OMICs Laboratory	Ahmed Al Harrasi et al
EPI_ISL_1009123	School of Pharmacy	School of Pharmacy	Ahmed Kandeil et al
EPI_ISL_1009128	School of Pharmacy	School of Pharmacy	Ahmed Kandeil et al
EPI_ISL_1009160	School of Pharmacy	School of Pharmacy	Ahmed Kandeil et al
EPI_ISL_1532290	Oman-National Influenza Center	Biotechnology & OMICs Laboratory	Aisha Al-Amri et al
EPI_ISL_1532291	Oman-National Influenza Center	Biotechnology & OMICs Laboratory	Aisha Al-Amri et al
EPI_ISL_1532303	Oman-National Influenza Center	Biotechnology & OMICs Laboratory	Aisha Al-Amri et al
EPI_ISL_510528	Communicable Disease Laboratory, Public Health Directorate	Communicable Disease Laboratory, Public Health Directorate	Al Wasti et al
EPI_ISL_710483	Department of Medical Laboratory Sciences, Arab American University	Department of Medical Laboratory Sciences, Arab American University	Al-Jawabreh et al
EPI_ISL_710484	Department of Medical Laboratory Sciences, Arab American University	Department of Medical Laboratory Sciences, Arab American University	Al-Jawabreh et al

EPI_ISL_632900	Communicable Disease Laboratory, Public Health Directorate	Communicable Disease Laboratory, Public Health Directorate	AlAbbas et al
EPI_ISL_733579	Temporary Specimen Collection Centre	Hong Kong Department of Health	Alan K.L. Tsang et al
EPI_ISL_759892	Temporary Specimen Collection Centre	Hong Kong Department of Health	Alan K.L. Tsang et al
EPI_ISL_955905	Pamela Youde Nethersole Eastern Hospital	Hong Kong Department of Health	Alan K.L. Tsang et al
EPI_ISL_1630158	Laboratório de Microbiologia Molecular - Universidade FEEVALE	Laboratório de Microbiologia Molecular - Universidade FEEVALE	Alana Witt Hansen et al
EPI_ISL_1629809	Laboratório de Microbiologia Molecular - Universidade FEEVALE	Molecular Microbiology Laboratory	Alana Witt Hansen et al
EPI_ISL_458079	Mitra Keluarga Hospital Kenjeran	Institute of Tropical Disease, Universitas Airlangga	Aldise M Nastri et al
EPI_ISL_529964	RSUD Bangil Pasuruan	Institute of Tropical Disease, Universitas Airlangga	Aldise M Nastri et al
EPI_ISL_1335890	Elisabeth Hospital Medan	Faculty of Medicine, Universitas Sumatera Utara; Institute of Tropical Disease, Universitas Airlangga	Aldise M Nastri et al
EPI_ISL_956404	General Hospital - Strumica	Research Center for Genetic Engineering and Biotechnology Georgi D. Efremov" , Macedonian Academy of Sciences and Arts	Aleksandar J. Dimovski et al
EPI_ISL_943995	General Hospital - Veles	Research Center for Genetic Engineering and Biotechnology Georgi D. Efremov" , Macedonian Academy of Sciences and Arts	Aleksandar J. Dimovski et al
EPI_ISL_944106	General Hospital - Strumica	Research Center for Genetic Engineering and Biotechnology Georgi D. Efremov" , Macedonian Academy of Sciences and Arts	Aleksandar J. Dimovski et al
EPI_ISL_947678	General Hospital - Tetovo	Research Center for Genetic Engineering and Biotechnology Georgi D. Efremov" , Macedonian Academy of Sciences and Arts	Aleksandar J. Dimovski et al
EPI_ISL_954611	General Hospital - Struga	Research Center for Genetic Engineering and Biotechnology Georgi D. Efremov" , Macedonian Academy of Sciences and Arts	Aleksandar J. Dimovski et al
EPI_ISL_954745	General Hospital - Ohrid	Research Center for Genetic Engineering and Biotechnology Georgi D. Efremov" , Macedonian Academy of Sciences and Arts	Aleksandar J. Dimovski et al
EPI_ISL_956383	General Hospital - Prilep	Research Center for Genetic Engineering and Biotechnology Georgi D. Efremov" , Macedonian Academy of Sciences and Arts	Aleksandar J. Dimovski et al
EPI_ISL_1013583	General Hospital - Prilep	Research Center for Genetic Engineering and Biotechnology Georgi D. Efremov" , Macedonian Academy of Sciences and Arts	Aleksandar J. Dimovski et al
EPI_ISL_1138412	General Hospital - Shtip	Research Center for Genetic Engineering and Biotechnology Georgi D. Efremov" , Macedonian Academy of Sciences and Arts	Aleksandar J. Dimovski et al
EPI_ISL_1014939	General Hospital - Struga	Research Center for Genetic Engineering and Biotechnology Georgi D. Efremov" , Macedonian Academy of Sciences and Arts	Aleksandar J. Dimovski et al
EPI_ISL_1138424	General Hospital - Struga	Research Center for Genetic Engineering and Biotechnology Georgi D. Efremov" , Macedonian Academy of Sciences and Arts	Aleksandar J. Dimovski et al
EPI_ISL_1018109	General Hospital - Stuga	Research Center for Genetic Engineering and Biotechnology Georgi D. Efremov" , Macedonian Academy of Sciences and Arts	Aleksandar J. Dimovski et al
EPI_ISL_1143746	General Hospital - Kumanovo	Research Center for Genetic Engineering and Biotechnology Georgi D. Efremov" , Macedonian Academy of Sciences and Arts	Aleksandar J. Dimovski et al
EPI_ISL_942961	General Hospital - Bitola	Research Center for Genetic Engineering and Biotechnology Georgi D. Efremov" , Macedonian Academy of Sciences and Arts	Aleksandar J.Dimovski et al
EPI_ISL_1499088	Institute of Microbiology and Immunology, Faculty of Medicine, University of Ljubljana	Institute of Microbiology and Immunology, Faculty of Medicine, University of Ljubljana	Alen Suljič et al

EPI_ISL_1499099	Institute of Microbiology and Immunology, Faculty of Medicine, University of Ljubljana	Institute of Microbiology and Immunology, Faculty of Medicine, University of Ljubljana	Alen Suljič et al
EPI_ISL_1499104	Institute of Microbiology and Immunology, Faculty of Medicine, University of Ljubljana	Institute of Microbiology and Immunology, Faculty of Medicine, University of Ljubljana	Alen Suljič et al
EPI_ISL_1098776	Institute of Microbiology and Immunology, Faculty of Medicine, University of Ljubljana	Institute of Microbiology and Immunology, Faculty of Medicine, University of Ljubljana	Alen Suljič et al
EPI_ISL_1098778	Institute of Microbiology and Immunology, Faculty of Medicine, University of Ljubljana	Institute of Microbiology and Immunology, Faculty of Medicine, University of Ljubljana	Alen Suljič et al
EPI_ISL_1668553	Institute of Microbiology and Immunology, Faculty of Medicine, University of Ljubljana	Institute of Microbiology and Immunology, Faculty of Medicine, University of Ljubljana	Alen Suljič et al
EPI_ISL_1098789	Institute of Microbiology and Immunology, Faculty of Medicine, University of Ljubljana	Institute of Microbiology and Immunology, Faculty of Medicine, University of Ljubljana	Alen Suljič et al
EPI_ISL_1098795	Institute of Microbiology and Immunology, Faculty of Medicine, University of Ljubljana	Institute of Microbiology and Immunology, Faculty of Medicine, University of Ljubljana	Alen Suljič et al
EPI_ISL_1668820	Institute of Microbiology and Immunology, Faculty of Medicine, University of Ljubljana	Institute of Microbiology and Immunology, Faculty of Medicine, University of Ljubljana	Alen Suljič et al
EPI_ISL_1098796	Institute of Microbiology and Immunology, Faculty of Medicine, University of Ljubljana	Institute of Microbiology and Immunology, Faculty of Medicine, University of Ljubljana	Alen Suljič et al
EPI_ISL_1181825	Institute of Microbiology and Immunology, Faculty of Medicine, University of Ljubljana	Institute of Microbiology and Immunology, Faculty of Medicine, University of Ljubljana	Alen Suljič et al
EPI_ISL_1181830	Institute of Microbiology and Immunology, Faculty of Medicine, University of Ljubljana	Institute of Microbiology and Immunology, Faculty of Medicine, University of Ljubljana	Alen Suljič et al
EPI_ISL_1510994	Institute of Microbiology and Immunology, Faculty of Medicine, University of Ljubljana	Institute of Microbiology and Immunology, Faculty of Medicine, University of Ljubljana	Alen Suljič et al
EPI_ISL_1510968	Institute of Microbiology and Immunology, Faculty of Medicine, University of Ljubljana	Institute of Microbiology and Immunology, Faculty of Medicine, University of Ljubljana	Alen Suljič et al
EPI_ISL_1510958	Institute of Microbiology and Immunology, Faculty of Medicine, University of Ljubljana	Institute of Microbiology and Immunology, Faculty of Medicine, University of Ljubljana	Alen Suljič et al
EPI_ISL_1511108	Institute of Microbiology and Immunology, Faculty of Medicine, University of Ljubljana	Institute of Microbiology and Immunology, Faculty of Medicine, University of Ljubljana	Alen Suljič et al
EPI_ISL_1511077	Institute of Microbiology and Immunology, Faculty of Medicine, University of Ljubljana	Institute of Microbiology and Immunology, Faculty of Medicine, University of Ljubljana	Alen Suljič et al
EPI_ISL_1511003	Institute of Microbiology and Immunology, Faculty of Medicine, University of Ljubljana	Institute of Microbiology and Immunology, Faculty of Medicine, University of Ljubljana	Alen Suljič et al
EPI_ISL_1511004	Institute of Microbiology and Immunology, Faculty of Medicine, University of Ljubljana	Institute of Microbiology and Immunology, Faculty of Medicine, University of Ljubljana	Alen Suljič et al
EPI_ISL_1511097	Institute of Microbiology and Immunology, Faculty of Medicine, University of Ljubljana	Institute of Microbiology and Immunology, Faculty of Medicine, University of Ljubljana	Alen Suljič et al
EPI_ISL_1511040	Institute of Microbiology and Immunology, Faculty of Medicine, University of Ljubljana	Institute of Microbiology and Immunology, Faculty of Medicine, University of Ljubljana	Alen Suljič et al
EPI_ISL_1511093	Institute of Microbiology and Immunology, Faculty of Medicine, University of Ljubljana	Institute of Microbiology and Immunology, Faculty of Medicine, University of Ljubljana	Alen Suljič et al
EPI_ISL_1511102	Institute of Microbiology and Immunology, Faculty of Medicine, University of Ljubljana	Institute of Microbiology and Immunology, Faculty of Medicine, University of Ljubljana	Alen Suljič et al

EPI_ISL_1511013	Institute of Microbiology and Immunology, Faculty of Medicine, University of Ljubljana	Institute of Microbiology and Immunology, Faculty of Medicine, University of Ljubljana	Alen Suljič et al
EPI_ISL_1498931	Institute of Microbiology and Immunology, Faculty of Medicine, University of Ljubljana	Institute of Microbiology and Immunology, Faculty of Medicine, University of Ljubljana	Alen Suljič et al
EPI_ISL_1498951	Institute of Microbiology and Immunology, Faculty of Medicine, University of Ljubljana	Institute of Microbiology and Immunology, Faculty of Medicine, University of Ljubljana	Alen Suljič et al
EPI_ISL_1498956	Institute of Microbiology and Immunology, Faculty of Medicine, University of Ljubljana	Institute of Microbiology and Immunology, Faculty of Medicine, University of Ljubljana	Alen Suljič et al
EPI_ISL_1181860	Institute of Microbiology and Immunology, Faculty of Medicine, University of Ljubljana	Institute of Microbiology and Immunology, Faculty of Medicine, University of Ljubljana	Alen Suljič et al
EPI_ISL_1668569	Institute of Microbiology and Immunology, Faculty of Medicine, University of Ljubljana	Institute of Microbiology and Immunology, Faculty of Medicine, University of Ljubljana	Alen Suljič et al
EPI_ISL_1213399	LAFEM/UESC	Bioinformatics Laboratory / LNCC	Alessandra P Lamarca et al
EPI_ISL_1213226	LAFEM/UESC	Bioinformatics Laboratory / LNCC	Alessandra P Lamarca et al
EPI_ISL_1213294	LAFEM/UESC	Bioinformatics Laboratory / LNCC	Alessandra P Lamarca et al
EPI_ISL_1213401	LAFEM/UESC	Bioinformatics Laboratory / LNCC	Alessandra P Lamarca et al
EPI_ISL_1213384	LAFEM/UESC	Bioinformatics Laboratory / LNCC	Alessandra P Lamarca et al
EPI_ISL_1213342	LAFEM/UESC	Bioinformatics Laboratory / LNCC	Alessandra P Lamarca et al
EPI_ISL_1213232	LAFEM/UESC	Bioinformatics Laboratory / LNCC	Alessandra P Lamarca et al
EPI_ISL_1213298	LAFEM/UESC	Bioinformatics Laboratory / LNCC	Alessandra P Lamarca et al
EPI_ISL_1213390	LAFEM/UESC	Bioinformatics Laboratory / LNCC	Alessandra P Lamarca et al
EPI_ISL_1213170	LAFEM/UESC	Bioinformatics Laboratory / LNCC	Alessandra P Lamarca et al
EPI_ISL_1213302	LAFEM/UESC	Bioinformatics Laboratory / LNCC	Alessandra P Lamarca et al
EPI_ISL_1213303	LAFEM/UESC	Bioinformatics Laboratory / LNCC	Alessandra P Lamarca et al
EPI_ISL_1213220	LAFEM/UESC	Bioinformatics Laboratory / LNCC	Alessandra P Lamarca et al
EPI_ISL_1213237	LAFEM/UESC	Bioinformatics Laboratory / LNCC	Alessandra P Lamarca et al
EPI_ISL_1213413	LAFEM/UESC	Bioinformatics Laboratory / LNCC	Alessandra P Lamarca et al
EPI_ISL_1213269	LAFEM/UESC	Bioinformatics Laboratory / LNCC	Alessandra P Lamarca et al
EPI_ISL_1213241	LAFEM/UESC	Bioinformatics Laboratory / LNCC	Alessandra P Lamarca et al
EPI_ISL_1213402	LAFEM/UESC	Bioinformatics Laboratory / LNCC	Alessandra P Lamarca et al
EPI_ISL_1213328	LAFEM/UESC	Bioinformatics Laboratory / LNCC	Alessandra P Lamarca et al
EPI_ISL_1213441	LBM/UFPB	Bioinformatics Laboratory / LNCC	Alessandra P Lamarca et al
EPI_ISL_1213433	LBM/UFPB	Bioinformatics Laboratory / LNCC	Alessandra P Lamarca et al
EPI_ISL_1213427	LBM/UFPB	Bioinformatics Laboratory / LNCC	Alessandra P Lamarca et al

EPI_ISL_1213404	LBM/UFPB	Bioinformatics Laboratory / LNCC	Alessandra P Lamarca et al
EPI_ISL_1213367	LBM/UFPB	Bioinformatics Laboratory / LNCC	Alessandra P Lamarca et al
EPI_ISL_1213392	LBM/UFPB	Bioinformatics Laboratory / LNCC	Alessandra P Lamarca et al
EPI_ISL_1213153	LBM/UFPB	Bioinformatics Laboratory / LNCC	Alessandra P Lamarca et al
EPI_ISL_1213213	LBM/UFPB	Bioinformatics Laboratory / LNCC	Alessandra P Lamarca et al
EPI_ISL_1213161	LBM/UFPB	Bioinformatics Laboratory / LNCC	Alessandra P Lamarca et al
EPI_ISL_1213438	LBM/UFPB	Bioinformatics Laboratory / LNCC	Alessandra P Lamarca et al
EPI_ISL_1213454	LBM/UFPB	Bioinformatics Laboratory / LNCC	Alessandra P Lamarca et al
EPI_ISL_1213461	LBM/UFPB	Bioinformatics Laboratory / LNCC	Alessandra P Lamarca et al
EPI_ISL_1213443	LBM/UFPB	Bioinformatics Laboratory / LNCC	Alessandra P Lamarca et al
EPI_ISL_1213201	LBM/UFPB	Bioinformatics Laboratory / LNCC	Alessandra P Lamarca et al
EPI_ISL_1213429	LBM/UFPB	Bioinformatics Laboratory / LNCC	Alessandra P Lamarca et al
EPI_ISL_1213453	LBM/UFPB	Bioinformatics Laboratory / LNCC	Alessandra P Lamarca et al
EPI_ISL_1213439	LBM/UFPB	Bioinformatics Laboratory / LNCC	Alessandra P Lamarca et al
EPI_ISL_1213431	LBM/UFPB	Bioinformatics Laboratory / LNCC	Alessandra P Lamarca et al
EPI_ISL_1213456	LBM/UFPB	Bioinformatics Laboratory / LNCC	Alessandra P Lamarca et al
EPI_ISL_1213369	LBM/UFPB	Bioinformatics Laboratory / LNCC	Alessandra P Lamarca et al
EPI_ISL_1213458	LBM/UFPB	Bioinformatics Laboratory / LNCC	Alessandra P Lamarca et al
EPI_ISL_1213459	LBM/UFPB	Bioinformatics Laboratory / LNCC	Alessandra P Lamarca et al
EPI_ISL_1213444	LBM/UFPB	Bioinformatics Laboratory / LNCC	Alessandra P Lamarca et al
EPI_ISL_1213344	LBM/UFPB	Bioinformatics Laboratory / LNCC	Alessandra P Lamarca et al
EPI_ISL_1213436	LBM/UFPB	Bioinformatics Laboratory / LNCC	Alessandra P Lamarca et al
EPI_ISL_1213336	LBM/UFPB	Bioinformatics Laboratory / LNCC	Alessandra P Lamarca et al
EPI_ISL_1213156	LBM/UFPB	Bioinformatics Laboratory / LNCC	Alessandra P Lamarca et al
EPI_ISL_1213449	LBM/UFPB	Bioinformatics Laboratory / LNCC	Alessandra P Lamarca et al
EPI_ISL_1213406	LBM/UFPB	Bioinformatics Laboratory / LNCC	Alessandra P Lamarca et al
EPI_ISL_1213370	LBM/UFPB	Bioinformatics Laboratory / LNCC	Alessandra P Lamarca et al
EPI_ISL_1213451	LBM/UFPB	Bioinformatics Laboratory / LNCC	Alessandra P Lamarca et al
EPI_ISL_1213372	Laboratório HLA/UERJ	Bioinformatics Laboratory / LNCC	Alessandra P Lamarca et al
EPI_ISL_1213339	Laboratório HLA/UERJ	Bioinformatics Laboratory / LNCC	Alessandra P Lamarca et al

EPI_ISL_1213248	Laboratório HLA/UERJ	Bioinformatics Laboratory / LNCC	Alessandra P Lamarca et al
EPI_ISL_1213422	Laboratório HLA/UERJ	Bioinformatics Laboratory / LNCC	Alessandra P Lamarca et al
EPI_ISL_1213424	Laboratório HLA/UERJ	Bioinformatics Laboratory / LNCC	Alessandra P Lamarca et al
EPI_ISL_1213309	Laboratório HLA/UERJ	Bioinformatics Laboratory / LNCC	Alessandra P Lamarca et al
EPI_ISL_1213425	Laboratório HLA/UERJ	Bioinformatics Laboratory / LNCC	Alessandra P Lamarca et al
EPI_ISL_1213171	Laboratório HLA/UERJ	Bioinformatics Laboratory / LNCC	Alessandra P Lamarca et al
EPI_ISL_1213378	Laboratório HLA/UERJ	Bioinformatics Laboratory / LNCC	Alessandra P Lamarca et al
EPI_ISL_1213270	Laboratório HLA/UERJ	Bioinformatics Laboratory / LNCC	Alessandra P Lamarca et al
EPI_ISL_1213151	Laboratório HLA/UERJ	Bioinformatics Laboratory / LNCC	Alessandra P Lamarca et al
EPI_ISL_1213408	Laboratório HLA/UERJ	Bioinformatics Laboratory / LNCC	Alessandra P Lamarca et al
EPI_ISL_1213381	Laboratório HLA/UERJ	Bioinformatics Laboratory / LNCC	Alessandra P Lamarca et al
EPI_ISL_1213393	Laboratório HLA/UERJ	Bioinformatics Laboratory / LNCC	Alessandra P Lamarca et al
EPI_ISL_1213382	Laboratório HLA/UERJ	Bioinformatics Laboratory / LNCC	Alessandra P Lamarca et al
EPI_ISL_1213272	Laboratório HLA/UERJ	Bioinformatics Laboratory / LNCC	Alessandra P Lamarca et al
EPI_ISL_1213249	Laboratório HLA/UERJ	Bioinformatics Laboratory / LNCC	Alessandra P Lamarca et al
EPI_ISL_1213149	Laboratório HLA/UERJ	Bioinformatics Laboratory / LNCC	Alessandra P Lamarca et al
EPI_ISL_1213148	Laboratório HLA/UERJ	Bioinformatics Laboratory / LNCC	Alessandra P Lamarca et al
EPI_ISL_1213155	Laboratório HLA/UERJ	Bioinformatics Laboratory / LNCC	Alessandra P Lamarca et al
EPI_ISL_1213158	Laboratório HLA/UERJ	Bioinformatics Laboratory / LNCC	Alessandra P Lamarca et al
EPI_ISL_1213315	IMT-UFRN/RN	Bioinformatics Laboratory / LNCC	Alessandra P Lamarca et al
EPI_ISL_1213350	IMT-UFRN/RN	Bioinformatics Laboratory / LNCC	Alessandra P Lamarca et al
EPI_ISL_1213275	IMT-UFRN/RN	Bioinformatics Laboratory / LNCC	Alessandra P Lamarca et al
EPI_ISL_1213277	IMT-UFRN/RN	Bioinformatics Laboratory / LNCC	Alessandra P Lamarca et al
EPI_ISL_1213173	IMT-UFRN/RN	Bioinformatics Laboratory / LNCC	Alessandra P Lamarca et al
EPI_ISL_1213175	IMT-UFRN/RN	Bioinformatics Laboratory / LNCC	Alessandra P Lamarca et al
EPI_ISL_1213177	IMT-UFRN/RN	Bioinformatics Laboratory / LNCC	Alessandra P Lamarca et al
EPI_ISL_1213317	IMT-UFRN/RN	Bioinformatics Laboratory / LNCC	Alessandra P Lamarca et al
EPI_ISL_1213178	IMT-UFRN/RN	Bioinformatics Laboratory / LNCC	Alessandra P Lamarca et al
EPI_ISL_1213185	IMT-UFRN/RN	Bioinformatics Laboratory / LNCC	Alessandra P Lamarca et al
EPI_ISL_1213357	IMT-UFRN/RN	Bioinformatics Laboratory / LNCC	Alessandra P Lamarca et al

EPI_ISL_1213180	IMT-UFRN/RN	Bioinformatics Laboratory / LNCC	Alessandra P Lamarca et al
EPI_ISL_1213329	IMT-UFRN/RN	Bioinformatics Laboratory / LNCC	Alessandra P Lamarca et al
EPI_ISL_1213279	IMT-UFRN/RN	Bioinformatics Laboratory / LNCC	Alessandra P Lamarca et al
EPI_ISL_1213281	IMT-UFRN/RN	Bioinformatics Laboratory / LNCC	Alessandra P Lamarca et al
EPI_ISL_1213282	IMT-UFRN/RN	Bioinformatics Laboratory / LNCC	Alessandra P Lamarca et al
EPI_ISL_1213284	IMT-UFRN/RN	Bioinformatics Laboratory / LNCC	Alessandra P Lamarca et al
EPI_ISL_1213286	IMT-UFRN/RN	Bioinformatics Laboratory / LNCC	Alessandra P Lamarca et al
EPI_ISL_1213322	IMT-UFRN/RN	Bioinformatics Laboratory / LNCC	Alessandra P Lamarca et al
EPI_ISL_1213288	IMT-UFRN/RN	Bioinformatics Laboratory / LNCC	Alessandra P Lamarca et al
EPI_ISL_1213182	IMT-UFRN/RN	Bioinformatics Laboratory / LNCC	Alessandra P Lamarca et al
EPI_ISL_1213183	IMT-UFRN/RN	Bioinformatics Laboratory / LNCC	Alessandra P Lamarca et al
EPI_ISL_1213289	IMT-UFRN/RN	Bioinformatics Laboratory / LNCC	Alessandra P Lamarca et al
EPI_ISL_1213364	IMT-UFRN/RN	Bioinformatics Laboratory / LNCC	Alessandra P Lamarca et al
EPI_ISL_1213291	IMT-UFRN/RN	Bioinformatics Laboratory / LNCC	Alessandra P Lamarca et al
EPI_ISL_1213293	IMT-UFRN/RN	Bioinformatics Laboratory / LNCC	Alessandra P Lamarca et al
EPI_ISL_1213199	IMT-UFRN/RN	Bioinformatics Laboratory / LNCC	Alessandra P Lamarca et al
EPI_ISL_1620638	Hospital Municipal Dr. Ignacio Proença de Gouvea	Instituto Butantan / Mendelics	Alexander Roberto Precioso et al
EPI_ISL_454497	RSE National Center for Biotechnology	RSE National Center for Biotechnology	Alexandr Shevtsov et al
EPI_ISL_454507	RSE National Center for Biotechnology	RSE National Center for Biotechnology	Alexandr Shevtsov et al
EPI_ISL_454508	RSE National Center for Biotechnology	RSE National Center for Biotechnology	Alexandr Shevtsov et al
EPI_ISL_454511	RSE National Center for Biotechnology	RSE National Center for Biotechnology	Alexandr Shevtsov et al
EPI_ISL_454512	RSE National Center for Biotechnology	RSE National Center for Biotechnology	Alexandr Shevtsov et al
EPI_ISL_454520	RSE National Center for Biotechnology	RSE National Center for Biotechnology	Alexandr Shevtsov et al
EPI_ISL_435045	Laboratory of Applied Genetics	RSE National Center for Biotechnology	Alexandr Shevtsov et al
EPI_ISL_435046	Laboratory of Applied Genetics	RSE National Center for Biotechnology	Alexandr Shevtsov et al
EPI_ISL_435047	Laboratory of Applied Genetics	RSE National Center for Biotechnology	Alexandr Shevtsov et al
EPI_ISL_470568	Hermes Pardini	Bioinformatics Laboratory / LNCC	Alexandra Gerber et al
EPI_ISL_470570	Hermes Pardini	Bioinformatics Laboratory / LNCC	Alexandra Gerber et al
EPI_ISL_470571	Hermes Pardini	Bioinformatics Laboratory / LNCC	Alexandra Gerber et al
EPI_ISL_470572	Hermes Pardini	Bioinformatics Laboratory / LNCC	Alexandra Gerber et al

EPI_ISL_470573	Hermes Pardini	Bioinformatics Laboratory / LNCC	Alexandra Gerber et al
EPI_ISL_470574	Hermes Pardini	Bioinformatics Laboratory / LNCC	Alexandra Gerber et al
EPI_ISL_470575	Hermes Pardini	Bioinformatics Laboratory / LNCC	Alexandra Gerber et al
EPI_ISL_470576	Hermes Pardini	Bioinformatics Laboratory / LNCC	Alexandra Gerber et al
EPI_ISL_470577	Hermes Pardini	Bioinformatics Laboratory / LNCC	Alexandra Gerber et al
EPI_ISL_470580	Hermes Pardini	Bioinformatics Laboratory / LNCC	Alexandra Gerber et al
EPI_ISL_470582	Hermes Pardini	Bioinformatics Laboratory / LNCC	Alexandra Gerber et al
EPI_ISL_470583	Hermes Pardini	Bioinformatics Laboratory / LNCC	Alexandra Gerber et al
EPI_ISL_470584	Hermes Pardini	Bioinformatics Laboratory / LNCC	Alexandra Gerber et al
EPI_ISL_470585	Hermes Pardini	Bioinformatics Laboratory / LNCC	Alexandra Gerber et al
EPI_ISL_470586	Hermes Pardini	Bioinformatics Laboratory / LNCC	Alexandra Gerber et al
EPI_ISL_470587	Hermes Pardini	Bioinformatics Laboratory / LNCC	Alexandra Gerber et al
EPI_ISL_470588	Hermes Pardini	Bioinformatics Laboratory / LNCC	Alexandra Gerber et al
EPI_ISL_470589	Simile	Bioinformatics Laboratory / LNCC	Alexandra Gerber et al
EPI_ISL_470590	Simile	Bioinformatics Laboratory / LNCC	Alexandra Gerber et al
EPI_ISL_470591	Simile	Bioinformatics Laboratory / LNCC	Alexandra Gerber et al
EPI_ISL_470592	Simile	Bioinformatics Laboratory / LNCC	Alexandra Gerber et al
EPI_ISL_470593	Simile	Bioinformatics Laboratory / LNCC	Alexandra Gerber et al
EPI_ISL_470594	Simile	Bioinformatics Laboratory / LNCC	Alexandra Gerber et al
EPI_ISL_470595	Simile	Bioinformatics Laboratory / LNCC	Alexandra Gerber et al
EPI_ISL_470596	Simile	Bioinformatics Laboratory / LNCC	Alexandra Gerber et al
EPI_ISL_470599	Hermes Pardini	Bioinformatics Laboratory / LNCC	Alexandra Gerber et al
EPI_ISL_470600	Hermes Pardini	Bioinformatics Laboratory / LNCC	Alexandra Gerber et al
EPI_ISL_470602	Hermes Pardini	Bioinformatics Laboratory / LNCC	Alexandra Gerber et al
EPI_ISL_470604	Hermes Pardini	Bioinformatics Laboratory / LNCC	Alexandra Gerber et al
EPI_ISL_470605	Hermes Pardini	Bioinformatics Laboratory / LNCC	Alexandra Gerber et al
EPI_ISL_470612	Hermes Pardini	Bioinformatics Laboratory / LNCC	Alexandra Gerber et al
EPI_ISL_470613	Hermes Pardini	Bioinformatics Laboratory / LNCC	Alexandra Gerber et al
EPI_ISL_470614	Hermes Pardini	Bioinformatics Laboratory / LNCC	Alexandra Gerber et al
EPI_ISL_470615	Laboratorio de Virologia Molecular / UFRJ	Bioinformatics Laboratory / LNCC	Alexandra Gerber et al

EPI_ISL_470617	Laboratorio de Virologia Molecular / UFRJ	Bioinformatics Laboratory / LNCC	Alexandra Gerber et al
EPI_ISL_470618	Laboratorio de Virologia Molecular / UFRJ	Bioinformatics Laboratory / LNCC	Alexandra Gerber et al
EPI_ISL_470620	Laboratorio de Virologia Molecular / UFRJ	Bioinformatics Laboratory / LNCC	Alexandra Gerber et al
EPI_ISL_470621	Laboratorio de Virologia Molecular / UFRJ	Bioinformatics Laboratory / LNCC	Alexandra Gerber et al
EPI_ISL_470623	Laboratorio de Virologia Molecular / UFRJ	Bioinformatics Laboratory / LNCC	Alexandra Gerber et al
EPI_ISL_470638	Laboratorio de Virologia Molecular / UFRJ	Bioinformatics Laboratory / LNCC	Alexandra Gerber et al
EPI_ISL_470651	Hermes Pardini	Bioinformatics Laboratory / LNCC	Alexandra Gerber et al
EPI_ISL_470652	Hermes Pardini	Bioinformatics Laboratory / LNCC	Alexandra Gerber et al
EPI_ISL_470653	Hermes Pardini	Bioinformatics Laboratory / LNCC	Alexandra Gerber et al
EPI_ISL_470654	Hermes Pardini	Bioinformatics Laboratory / LNCC	Alexandra Gerber et al
EPI_ISL_470655	Hermes Pardini	Bioinformatics Laboratory / LNCC	Alexandra Gerber et al
EPI_ISL_419655	Center for Virology, Medical University of Vienna	Bergthaler laboratory, CeMM Research Center for Molecular Medicine of the Austrian Academy of Sciences	Alexandra Popa et al
EPI_ISL_419657	Center for Virology, Medical University of Vienna	Bergthaler laboratory, CeMM Research Center for Molecular Medicine of the Austrian Academy of Sciences	Alexandra Popa et al
EPI_ISL_583563	Center for Virology, Medical University of Vienna	Bergthaler laboratory, CeMM Research Center for Molecular Medicine of the Austrian Academy of Sciences	Alexandra Popa et al
EPI_ISL_437993	Center for Virology, Medical University of Vienna	Bergthaler laboratory, CeMM Research Center for Molecular Medicine of the Austrian Academy of Sciences	Alexandra Popa et al
EPI_ISL_437995	Center for Virology, Medical University of Vienna	Bergthaler laboratory, CeMM Research Center for Molecular Medicine of the Austrian Academy of Sciences	Alexandra Popa et al
EPI_ISL_475789	Center for Virology, Medical University of Vienna	Bergthaler laboratory, CeMM Research Center for Molecular Medicine of the Austrian Academy of Sciences	Alexandra Popa et al
EPI_ISL_475790	Center for Virology, Medical University of Vienna	Bergthaler laboratory, CeMM Research Center for Molecular Medicine of the Austrian Academy of Sciences	Alexandra Popa et al
EPI_ISL_475796	Center for Virology, Medical University of Vienna	Bergthaler laboratory, CeMM Research Center for Molecular Medicine of the Austrian Academy of Sciences	Alexandra Popa et al
EPI_ISL_475805	Center for Virology, Medical University of Vienna	Bergthaler laboratory, CeMM Research Center for Molecular Medicine of the Austrian Academy of Sciences	Alexandra Popa et al
EPI_ISL_475832	Austrian Agency for Health and Food Safety (AGES)	Bergthaler laboratory, CeMM Research Center for Molecular Medicine of the Austrian Academy of Sciences	Alexandra Popa et al
EPI_ISL_583573	Austrian Agency for Health and Food Safety (AGES)	Bergthaler laboratory, CeMM Research Center for Molecular Medicine of the Austrian Academy of Sciences	Alexandra Popa et al
EPI_ISL_475836	Austrian Agency for Health and Food Safety (AGES)	Bergthaler laboratory, CeMM Research Center for Molecular Medicine of the Austrian Academy of Sciences	Alexandra Popa et al
EPI_ISL_583575	Austrian Agency for Health and Food Safety (AGES)	Bergthaler laboratory, CeMM Research Center for Molecular Medicine of the Austrian Academy of Sciences	Alexandra Popa et al
EPI_ISL_475896	Zentralinstitut für medizinische und chemische Labordiagnostik, Universitätskliniken Insbruck	Bergthaler laboratory, CeMM Research Center for Molecular Medicine of the Austrian Academy of Sciences	Alexandra Popa et al

EPI_ISL_583604	Institut für Virologie am Department für Hygiene, Mikrobiologie und Public Health	Bergthaler laboratory, CeMM Research Center for Molecular Medicine of the Austrian Academy of Sciences	Alexandra Popa et al
EPI_ISL_583632	Austrian Agency for Health and Food Safety (AGES)	Bergthaler laboratory, CeMM Research Center for Molecular Medicine of the Austrian Academy of Sciences	Alexandra Popa et al
EPI_ISL_583638	Austrian Agency for Health and Food Safety (AGES)	Bergthaler laboratory, CeMM Research Center for Molecular Medicine of the Austrian Academy of Sciences	Alexandra Popa et al
EPI_ISL_583666	Austrian Agency for Health and Food Safety (AGES)	Bergthaler laboratory, CeMM Research Center for Molecular Medicine of the Austrian Academy of Sciences	Alexandra Popa et al
EPI_ISL_583670	Austrian Agency for Health and Food Safety (AGES)	Bergthaler laboratory, CeMM Research Center for Molecular Medicine of the Austrian Academy of Sciences	Alexandra Popa et al
EPI_ISL_583677	Austrian Agency for Health and Food Safety (AGES)	Bergthaler laboratory, CeMM Research Center for Molecular Medicine of the Austrian Academy of Sciences	Alexandra Popa et al
EPI_ISL_583717	Center for Virology, Medical University of Vienna	Bergthaler laboratory, CeMM Research Center for Molecular Medicine of the Austrian Academy of Sciences	Alexandra Popa et al
EPI_ISL_583734	Dr. Gernot Walder GmbH	Bergthaler laboratory, CeMM Research Center for Molecular Medicine of the Austrian Academy of Sciences	Alexandra Popa et al
EPI_ISL_583762	Dr. Gernot Walder GmbH	Bergthaler laboratory, CeMM Research Center for Molecular Medicine of the Austrian Academy of Sciences	Alexandra Popa et al
EPI_ISL_583854	Institute for Laboratory Diagnostics and Microbiology, Klinikum Klagenfurt am Worthersee	Bergthaler laboratory, CeMM Research Center for Molecular Medicine of the Austrian Academy of Sciences	Alexandra Popa et al
EPI_ISL_583883	Austrian Agency for Health and Food Safety (AGES)	Bergthaler laboratory, CeMM Research Center for Molecular Medicine of the Austrian Academy of Sciences	Alexandra Popa et al
EPI_ISL_583884	Austrian Agency for Health and Food Safety (AGES)	Bergthaler laboratory, CeMM Research Center for Molecular Medicine of the Austrian Academy of Sciences	Alexandra Popa et al
EPI_ISL_583885	Austrian Agency for Health and Food Safety (AGES)	Bergthaler laboratory, CeMM Research Center for Molecular Medicine of the Austrian Academy of Sciences	Alexandra Popa et al
EPI_ISL_583890	Austrian Agency for Health and Food Safety (AGES)	Bergthaler laboratory, CeMM Research Center for Molecular Medicine of the Austrian Academy of Sciences	Alexandra Popa et al
EPI_ISL_1396166	Laboratorio de Virología del Hospital de Niños Dr. Ricardo Gutierrez	Área de Secuenciación del Laboratorio de Virología del Hospital de Niños Dr. Ricardo Gutierrez on behalf of 'Proyecto Argentino Interinstitucional de genomica de SARS-CoV-2' (PAIS Consortium)	Alexay et al
EPI_ISL_1396170	Laboratorio de Virología del Hospital de Niños Dr. Ricardo Gutierrez	Área de Secuenciación del Laboratorio de Virología del Hospital de Niños Dr. Ricardo Gutierrez on behalf of 'Proyecto Argentino Interinstitucional de genomica de SARS-CoV-2' (PAIS Consortium)	Alexay et al
EPI_ISL_1396173	Laboratorio de Virología del Hospital de Niños Dr. Ricardo Gutierrez	Área de Secuenciación del Laboratorio de Virología del Hospital de Niños Dr. Ricardo Gutierrez on behalf of 'Proyecto Argentino Interinstitucional de genomica de SARS-CoV-2' (PAIS Consortium)	Alexay et al
EPI_ISL_1396175	Laboratorio de Virología del Hospital de Niños Dr. Ricardo Gutierrez	Área de Secuenciación del Laboratorio de Virología del Hospital de Niños Dr. Ricardo Gutierrez on behalf of 'Proyecto Argentino Interinstitucional de genomica de SARS-CoV-2' (PAIS Consortium)	Alexay et al
EPI_ISL_1396185	Laboratorio de Virología del Hospital de Niños Dr. Ricardo Gutierrez	Área de Secuenciación del Laboratorio de Virología del Hospital de Niños Dr. Ricardo Gutierrez on behalf of 'Proyecto Argentino Interinstitucional de genomica de SARS-CoV-2' (PAIS Consortium)	Alexay et al

EPI_ISL_1396191	Laboratorio de Virología del Hospital de Niños Dr. Ricardo Gutierrez	Área de Secuenciación del Laboratorio de Virología del Hospital de Niños Dr. Ricardo Gutierrez on behalf of 'Proyecto Argentino Interinstitucional de genomica de SARS-CoV-2' (PAIS Consortium)	Alexay et al
EPI_ISL_1396192	Laboratorio de Virología del Hospital de Niños Dr. Ricardo Gutierrez	Área de Secuenciación del Laboratorio de Virología del Hospital de Niños Dr. Ricardo Gutierrez on behalf of 'Proyecto Argentino Interinstitucional de genomica de SARS-CoV-2' (PAIS Consortium)	Alexay et al
EPI_ISL_1396193	Laboratorio de Virología del Hospital de Niños Dr. Ricardo Gutierrez	Área de Secuenciación del Laboratorio de Virología del Hospital de Niños Dr. Ricardo Gutierrez on behalf of 'Proyecto Argentino Interinstitucional de genomica de SARS-CoV-2' (PAIS Consortium)	Alexay et al
EPI_ISL_1396194	Laboratorio de Virología del Hospital de Niños Dr. Ricardo Gutierrez	Área de Secuenciación del Laboratorio de Virología del Hospital de Niños Dr. Ricardo Gutierrez on behalf of 'Proyecto Argentino Interinstitucional de genomica de SARS-CoV-2' (PAIS Consortium)	Alexay et al
EPI_ISL_1396197	Laboratorio de Virología del Hospital de Niños Dr. Ricardo Gutierrez	Área de Secuenciación del Laboratorio de Virología del Hospital de Niños Dr. Ricardo Gutierrez on behalf of 'Proyecto Argentino Interinstitucional de genomica de SARS-CoV-2' (PAIS Consortium)	Alexay et al
EPI_ISL_1396201	Laboratorio de Virología del Hospital de Niños Dr. Ricardo Gutierrez	Área de Secuenciación del Laboratorio de Virología del Hospital de Niños Dr. Ricardo Gutierrez on behalf of 'Proyecto Argentino Interinstitucional de genomica de SARS-CoV-2' (PAIS Consortium)	Alexay et al
EPI_ISL_1396204	Laboratorio de Virología del Hospital de Niños Dr. Ricardo Gutierrez	Área de Secuenciación del Laboratorio de Virología del Hospital de Niños Dr. Ricardo Gutierrez on behalf of 'Proyecto Argentino Interinstitucional de genomica de SARS-CoV-2' (PAIS Consortium)	Alexay et al
EPI_ISL_1396206	Laboratorio de Virología del Hospital de Niños Dr. Ricardo Gutierrez	Área de Secuenciación del Laboratorio de Virología del Hospital de Niños Dr. Ricardo Gutierrez on behalf of 'Proyecto Argentino Interinstitucional de genomica de SARS-CoV-2' (PAIS Consortium)	Alexay et al
EPI_ISL_1396215	Laboratorio de Virología del Hospital de Niños Dr. Ricardo Gutierrez	Área de Secuenciación del Laboratorio de Virología del Hospital de Niños Dr. Ricardo Gutierrez on behalf of 'Proyecto Argentino Interinstitucional de genomica de SARS-CoV-2' (PAIS Consortium)	Alexay et al
EPI_ISL_1396224	Laboratorio de Virología del Hospital de Niños Dr. Ricardo Gutierrez	Área de Secuenciación del Laboratorio de Virología del Hospital de Niños Dr. Ricardo Gutierrez on behalf of 'Proyecto Argentino Interinstitucional de genomica de SARS-CoV-2' (PAIS Consortium)	Alexay et al
EPI_ISL_1396225	Laboratorio de Virología del Hospital de Niños Dr. Ricardo Gutierrez	Área de Secuenciación del Laboratorio de Virología del Hospital de Niños Dr. Ricardo Gutierrez on behalf of 'Proyecto Argentino Interinstitucional de genomica de SARS-CoV-2' (PAIS Consortium)	Alexay et al
EPI_ISL_1396244	Laboratorio de Virología del Hospital de Niños Dr. Ricardo Gutierrez	Área de Secuenciación del Laboratorio de Virología del Hospital de Niños Dr. Ricardo Gutierrez on behalf of 'Proyecto Argentino Interinstitucional de genomica de SARS-CoV-2' (PAIS Consortium)	Alexay et al
EPI_ISL_1395778	Laboratorio de Virología del Hospital de Niños Dr. Ricardo Gutierrez	Área de Secuenciación del Laboratorio de Virología del Hospital de Niños Dr. Ricardo Gutierrez on behalf of 'Proyecto Argentino Interinstitucional de genomica de SARS-CoV-2' (PAIS Consortium)	Alexay et al
EPI_ISL_1395779	Laboratorio de Virología del Hospital de Niños Dr. Ricardo Gutierrez	Área de Secuenciación del Laboratorio de Virología del Hospital de Niños Dr. Ricardo Gutierrez on behalf of 'Proyecto Argentino Interinstitucional de genomica de SARS-CoV-2' (PAIS Consortium)	Alexay et al
EPI_ISL_1395780	Laboratorio de Virología del Hospital de Niños Dr. Ricardo Gutierrez	Área de Secuenciación del Laboratorio de Virología del Hospital de Niños Dr. Ricardo Gutierrez on behalf of 'Proyecto Argentino Interinstitucional de genomica de SARS-CoV-2' (PAIS Consortium)	Alexay et al

EPI_ISL_1395792	Laboratorio de Virología del Hospital de Niños Dr. Ricardo Gutierrez	Área de Secuenciación del Laboratorio de Virología del Hospital de Niños Dr. Ricardo Gutierrez on behalf of 'Proyecto Argentino Interinstitucional de genomica de SARS-CoV-2' (PAIS Consortium)	Alexay et al
EPI_ISL_1395793	Laboratorio de Virología del Hospital de Niños Dr. Ricardo Gutierrez	Área de Secuenciación del Laboratorio de Virología del Hospital de Niños Dr. Ricardo Gutierrez on behalf of 'Proyecto Argentino Interinstitucional de genomica de SARS-CoV-2' (PAIS Consortium)	Alexay et al
EPI_ISL_1396246	Laboratorio de Virología del Hospital de Niños Dr. Ricardo Gutierrez	Biocódices SA. on behalf of 'Proyecto Argentino Interinstitucional de genomica de SARS-CoV-2' (PAIS Consortium)	Alexay et al
EPI_ISL_1396304	Laboratorio de Virología del Hospital de Niños Dr. Ricardo Gutierrez	Biocódices SA. on behalf of 'Proyecto Argentino Interinstitucional de genomica de SARS-CoV-2' (PAIS Consortium)	Alexay et al
EPI_ISL_1396318	Laboratorio de Virología del Hospital de Niños Dr. Ricardo Gutierrez	Biocódices SA. on behalf of 'Proyecto Argentino Interinstitucional de genomica de SARS-CoV-2' (PAIS Consortium)	Alexay et al
EPI_ISL_1396327	Laboratorio de Virología del Hospital de Niños Dr. Ricardo Gutierrez	Biocódices SA. on behalf of 'Proyecto Argentino Interinstitucional de genomica de SARS-CoV-2' (PAIS Consortium)	Alexay et al
EPI_ISL_872637	Pathogenic Microorganisms Variability Laboratory	Pathogenic Microorganisms Variability Laboratory	Alexey Shchetinin et al
EPI_ISL_872638	Pathogenic Microorganisms Variability Laboratory	Pathogenic Microorganisms Variability Laboratory	Alexey Shchetinin et al
EPI_ISL_1233441	National Center for Infectious and Parasitic Diseases (NCIPD)	National Center for Infectious and Parasitic Diseases (NCIPD)	Alexiev et al
EPI_ISL_1233454	National Center for Infectious and Parasitic Diseases (NCIPD)	National Center for Infectious and Parasitic Diseases (NCIPD)	Alexiev et al
EPI_ISL_1233495	National Center for Infectious and Parasitic Diseases (NCIPD)	National Center for Infectious and Parasitic Diseases (NCIPD)	Alexiev et al
EPI_ISL_1233531	National Center for Infectious and Parasitic Diseases (NCIPD)	National Center for Infectious and Parasitic Diseases (NCIPD)	Alexiev et al
EPI_ISL_1233561	National Center for Infectious and Parasitic Diseases (NCIPD)	National Center for Infectious and Parasitic Diseases (NCIPD)	Alexiev et al
EPI_ISL_1233611	National Center for Infectious and Parasitic Diseases (NCIPD)	National Center for Infectious and Parasitic Diseases (NCIPD)	Alexiev et al
EPI_ISL_1233617	National Center for Infectious and Parasitic Diseases (NCIPD)	National Center for Infectious and Parasitic Diseases (NCIPD)	Alexiev et al
EPI_ISL_1233623	National Center for Infectious and Parasitic Diseases (NCIPD)	National Center for Infectious and Parasitic Diseases (NCIPD)	Alexiev et al
EPI_ISL_1231635	National Center for Infectious and Parasitic Diseases (NCIPD)	National Center for Infectious and Parasitic Diseases (NCIPD)	Alexiev et al
EPI_ISL_1401123	National Center of Infectious and Parasitic Diseases	National Center of Infectious and Parasitic Diseases	Alexiev et al
EPI_ISL_1401141	National Center of Infectious and Parasitic Diseases	National Center of Infectious and Parasitic Diseases	Alexiev et al
EPI_ISL_1401437	National Center of Infectious and Parasitic Diseases	National Center of Infectious and Parasitic Diseases	Alexiev et al
EPI_ISL_1608340	National Center of Infectious and Parasitic Diseases	National Center of Infectious and Parasitic Diseases	Alexiev et al
EPI_ISL_1608347	National Center of Infectious and Parasitic Diseases	National Center of Infectious and Parasitic Diseases	Alexiev et al
EPI_ISL_1608354	National Center of Infectious and Parasitic Diseases	National Center of Infectious and Parasitic Diseases	Alexiev et al
EPI_ISL_1608362	National Center of Infectious and Parasitic Diseases	National Center of Infectious and Parasitic Diseases	Alexiev et al
EPI_ISL_1608370	National Center of Infectious and Parasitic Diseases	National Center of Infectious and Parasitic Diseases	Alexiev et al
EPI_ISL_1608436	National Center of Infectious and Parasitic Diseases	National Center of Infectious and Parasitic Diseases	Alexiev et al
EPI_ISL_1608511	National Center of Infectious and Parasitic Diseases	National Center of Infectious and Parasitic Diseases	Alexiev et al

EPI_ISL_1302126	National Center of Infectious and Parasitic Diseases	National Center of Infectious and Parasitic Diseases	Alexiev et al
EPI_ISL_1302130	National Center of Infectious and Parasitic Diseases	National Center of Infectious and Parasitic Diseases	Alexiev et al
EPI_ISL_1302046	National Center of Infectious and Parasitic Diseases	National Center of Infectious and Parasitic Diseases	Alexiev et al
EPI_ISL_1509778	National Center of Infectious and Parasitic Diseases	National Center of Infectious and Parasitic Diseases	Alexiev et al
EPI_ISL_1509800	National Center of Infectious and Parasitic Diseases	National Center of Infectious and Parasitic Diseases	Alexiev et al
EPI_ISL_1509887	National Center of Infectious and Parasitic Diseases	National Center of Infectious and Parasitic Diseases	Alexiev et al
EPI_ISL_959923	University Medical Center Hamburg Eppendorf	Heinrich Pette Institute, Leibniz Institute for Experimental Virology	Alexis Robitaille et al
EPI_ISL_960056	University Medical Center Hamburg Eppendorf	Heinrich Pette Institute, Leibniz Institute for Experimental Virology	Alexis Robitaille et al
EPI_ISL_775788	University Medical Center Hamburg Eppendorf	Heinrich Pette Institute, Leibniz Institute for Experimental Virology	Alexis Robitaille et al
EPI_ISL_775789	University Medical Center Hamburg Eppendorf	Heinrich Pette Institute, Leibniz Institute for Experimental Virology	Alexis Robitaille et al
EPI_ISL_776012	University Medical Center Hamburg Eppendorf	Heinrich Pette Institute, Leibniz Institute for Experimental Virology	Alexis Robitaille et al
EPI_ISL_776539	University Medical Center Hamburg Eppendorf	Heinrich Pette Institute, Leibniz Institute for Experimental Virology	Alexis Robitaille et al
EPI_ISL_491945	Instituto Nacional de Investigación en Salud Pública - INSPI	INSPI - Charité	Alfredo Bruno Caicedo et al
EPI_ISL_491944	Instituto Nacional de Investigación en Salud Pública - INSPI	INSPI - Charité	Alfredo Bruno Caicedo et al
EPI_ISL_491948	Instituto Nacional de Investigación en Salud Pública - INSPI	INSPI - Charité	Alfredo Bruno Caicedo et al
EPI_ISL_491953	Instituto Nacional de Investigación en Salud Pública - INSPI	INSPI - Charité	Alfredo Bruno Caicedo et al
EPI_ISL_491950	Instituto Nacional de Investigación en Salud Pública - INSPI	INSPI - Charité	Alfredo Bruno Caicedo et al
EPI_ISL_491949	Instituto Nacional de Investigación en Salud Pública - INSPI	INSPI - Charité	Alfredo Bruno Caicedo et al
EPI_ISL_491952	Instituto Nacional de Investigación en Salud Pública - INSPI	INSPI - Charité	Alfredo Bruno Caicedo et al
EPI_ISL_491954	Instituto Nacional de Investigación en Salud Pública - INSPI	INSPI - Charité	Alfredo Bruno Caicedo et al
EPI_ISL_632905	Communicable Disease Laboratory, Public Health Directorate	Communicable Disease Laboratory, Public Health Directorate	AlHujairi et al
EPI_ISL_632908	Genomic Sciences, Rehman Medical Institute	Genomic Sciences, Rehman Medical Institute	Ali et al
EPI_ISL_943595	Central Laboratory of Public Health of Rio Grande do Sul (Lacen-RS)	State Center for Health Surveillance of the Health Department of the State of Rio Grande do Sul (CEVS/SES-RS)	Aline Campos et al
EPI_ISL_943596	Central Laboratory of Public Health of Rio Grande do Sul (Lacen-RS)	State Center for Health Surveillance of the Health Department of the State of Rio Grande do Sul (CEVS/SES-RS)	Aline Campos et al
EPI_ISL_943589	Central Laboratory of Public Health of Rio Grande do Sul (Lacen-RS)	State Center for Health Surveillance of the Health Department of the State of Rio Grande do Sul (CEVS/SES-RS)	Aline Campos et al
EPI_ISL_943597	Central Laboratory of Public Health of Rio Grande do Sul (Lacen-RS)	State Center for Health Surveillance of the Health Department of the State of Rio Grande do Sul (CEVS/SES-RS)	Aline Campos et al
EPI_ISL_943598	Central Laboratory of Public Health of Rio Grande do Sul (Lacen-RS)	State Center for Health Surveillance of the Health Department of the State of Rio Grande do Sul (CEVS/SES-RS)	Aline Campos et al
EPI_ISL_943590	Central Laboratory of Public Health of Rio Grande do Sul (Lacen-RS)	State Center for Health Surveillance of the Health Department of the State of Rio Grande do Sul (CEVS/SES-RS)	Aline Campos et al

EPI_ISL_943591	Central Laboratory of Public Health of Rio Grande do Sul (Lacen-RS)	State Center for Health Surveillance of the Health Department of the State of Rio Grande do Sul (CEVS/SES-RS)	Aline Campos et al
EPI_ISL_943593	Central Laboratory of Public Health of Rio Grande do Sul (Lacen-RS)	State Center for Health Surveillance of the Health Department of the State of Rio Grande do Sul (CEVS/SES-RS)	Aline Campos et al
EPI_ISL_943594	Central Laboratory of Public Health of Rio Grande do Sul (Lacen-RS)	State Center for Health Surveillance of the Health Department of the State of Rio Grande do Sul (CEVS/SES-RS)	Aline Campos et al
EPI_ISL_943600	Central Laboratory of Public Health of Rio Grande do Sul (Lacen-RS)	State Center for Health Surveillance of the Health Department of the State of Rio Grande do Sul (CEVS/SES-RS)	Aline Campos et al
EPI_ISL_943579	Central Laboratory of Public Health of Rio Grande do Sul (Lacen-RS)	State Center for Health Surveillance of the Health Department of the State of Rio Grande do Sul (CEVS/SES-RS)	Aline Campos et al
EPI_ISL_943580	Central Laboratory of Public Health of Rio Grande do Sul (Lacen-RS)	State Center for Health Surveillance of the Health Department of the State of Rio Grande do Sul (CEVS/SES-RS)	Aline Campos et al
EPI_ISL_943577	Central Laboratory of Public Health of Rio Grande do Sul (Lacen-RS)	State Center for Health Surveillance of the Health Department of the State of Rio Grande do Sul (CEVS/SES-RS)	Aline Campos et al
EPI_ISL_943578	Central Laboratory of Public Health of Rio Grande do Sul (Lacen-RS)	State Center for Health Surveillance of the Health Department of the State of Rio Grande do Sul (CEVS/SES-RS)	Aline Campos et al
EPI_ISL_943576	Central Laboratory of Public Health of Rio Grande do Sul (Lacen-RS)	State Center for Health Surveillance of the Health Department of the State of Rio Grande do Sul (CEVS/SES-RS)	Aline Campos et al
EPI_ISL_943581	Central Laboratory of Public Health of Rio Grande do Sul (Lacen-RS)	State Center for Health Surveillance of the Health Department of the State of Rio Grande do Sul (CEVS/SES-RS)	Aline Campos et al
EPI_ISL_943582	Central Laboratory of Public Health of Rio Grande do Sul (Lacen-RS)	State Center for Health Surveillance of the Health Department of the State of Rio Grande do Sul (CEVS/SES-RS)	Aline Campos et al
EPI_ISL_943583	Central Laboratory of Public Health of Rio Grande do Sul (Lacen-RS)	State Center for Health Surveillance of the Health Department of the State of Rio Grande do Sul (CEVS/SES-RS)	Aline Campos et al
EPI_ISL_943584	Central Laboratory of Public Health of Rio Grande do Sul (Lacen-RS)	State Center for Health Surveillance of the Health Department of the State of Rio Grande do Sul (CEVS/SES-RS)	Aline Campos et al
EPI_ISL_943585	Central Laboratory of Public Health of Rio Grande do Sul (Lacen-RS)	State Center for Health Surveillance of the Health Department of the State of Rio Grande do Sul (CEVS/SES-RS)	Aline Campos et al
EPI_ISL_943588	Central Laboratory of Public Health of Rio Grande do Sul (Lacen-RS)	State Center for Health Surveillance of the Health Department of the State of Rio Grande do Sul (CEVS/SES-RS)	Aline Campos et al
EPI_ISL_943586	Central Laboratory of Public Health of Rio Grande do Sul (Lacen-RS)	State Center for Health Surveillance of the Health Department of the State of Rio Grande do Sul (CEVS/SES-RS)	Aline Campos et al
EPI_ISL_943587	Central Laboratory of Public Health of Rio Grande do Sul (Lacen-RS)	State Center for Health Surveillance of the Health Department of the State of Rio Grande do Sul (CEVS/SES-RS)	Aline Campos et al
EPI_ISL_943574	Central Laboratory of Public Health of Rio Grande do Sul (Lacen-RS)	State Center for Health Surveillance of the Health Department of the State of Rio Grande do Sul (CEVS/SES-RS)	Aline Campos et al
EPI_ISL_943575	Central Laboratory of Public Health of Rio Grande do Sul (Lacen-RS)	State Center for Health Surveillance of the Health Department of the State of Rio Grande do Sul (CEVS/SES-RS)	Aline Campos et al
EPI_ISL_943592	Central Laboratory of Public Health of Rio Grande do Sul (Lacen-RS)	State Center for Health Surveillance of the Health Department of the State of Rio Grande do Sul (CEVS/SES-RS)	Aline Campos et al
EPI_ISL_943605	Central Laboratory of Public Health of Rio Grande do Sul (Lacen-RS)	State Center for Health Surveillance of the Health Department of the State of Rio Grande do Sul (CEVS/SES-RS)	Aline Campos et al
EPI_ISL_943606	Central Laboratory of Public Health of Rio Grande do Sul (Lacen-RS)	State Center for Health Surveillance of the Health Department of the State of Rio Grande do Sul (CEVS/SES-RS)	Aline Campos et al

EPI_ISL_943607	Central Laboratory of Public Health of Rio Grande do Sul (Lacen-RS)	State Center for Health Surveillance of the Health Department of the State of Rio Grande do Sul (CEVS/SES-RS)	Aline Campos et al
EPI_ISL_943608	Central Laboratory of Public Health of Rio Grande do Sul (Lacen-RS)	State Center for Health Surveillance of the Health Department of the State of Rio Grande do Sul (CEVS/SES-RS)	Aline Campos et al
EPI_ISL_943602	Central Laboratory of Public Health of Rio Grande do Sul (Lacen-RS)	State Center for Health Surveillance of the Health Department of the State of Rio Grande do Sul (CEVS/SES-RS)	Aline Campos et al
EPI_ISL_943603	Central Laboratory of Public Health of Rio Grande do Sul (Lacen-RS)	State Center for Health Surveillance of the Health Department of the State of Rio Grande do Sul (CEVS/SES-RS)	Aline Campos et al
EPI_ISL_943609	Central Laboratory of Public Health of Rio Grande do Sul (Lacen-RS)	State Center for Health Surveillance of the Health Department of the State of Rio Grande do Sul (CEVS/SES-RS)	Aline Campos et al
EPI_ISL_943611	Central Laboratory of Public Health of Rio Grande do Sul (Lacen-RS)	State Center for Health Surveillance of the Health Department of the State of Rio Grande do Sul (CEVS/SES-RS)	Aline Campos et al
EPI_ISL_943610	Central Laboratory of Public Health of Rio Grande do Sul (Lacen-RS)	State Center for Health Surveillance of the Health Department of the State of Rio Grande do Sul (CEVS/SES-RS)	Aline Campos et al
EPI_ISL_943612	Central Laboratory of Public Health of Rio Grande do Sul (Lacen-RS)	State Center for Health Surveillance of the Health Department of the State of Rio Grande do Sul (CEVS/SES-RS)	Aline Campos et al
EPI_ISL_943613	Central Laboratory of Public Health of Rio Grande do Sul (Lacen-RS)	State Center for Health Surveillance of the Health Department of the State of Rio Grande do Sul (CEVS/SES-RS)	Aline Campos et al
EPI_ISL_943604	Central Laboratory of Public Health of Rio Grande do Sul (Lacen-RS)	State Center for Health Surveillance of the Health Department of the State of Rio Grande do Sul (CEVS/SES-RS)	Aline Campos et al
EPI_ISL_983863	Central Laboratory of Public Health of Rio Grande do Sul (Lacen-RS)	State Center for Health Surveillance of the Health Department of the State of Rio Grande do Sul (CEVS/SES-RS)	Aline Campos et al
EPI_ISL_983864	Central Laboratory of Public Health of Rio Grande do Sul (Lacen-RS)	State Center for Health Surveillance of the Health Department of the State of Rio Grande do Sul (CEVS/SES-RS)	Aline Campos et al
EPI_ISL_983867	Central Laboratory of Public Health of Rio Grande do Sul (Lacen-RS)	State Center for Health Surveillance of the Health Department of the State of Rio Grande do Sul (CEVS/SES-RS)	Aline Campos et al
EPI_ISL_983869	Central Laboratory of Public Health of Rio Grande do Sul (Lacen-RS)	State Center for Health Surveillance of the Health Department of the State of Rio Grande do Sul (CEVS/SES-RS)	Aline Campos et al
EPI_ISL_983865	Central Laboratory of Public Health of Rio Grande do Sul (Lacen-RS)	State Center for Health Surveillance of the Health Department of the State of Rio Grande do Sul (CEVS/SES-RS)	Aline Campos et al
EPI_ISL_983868	Central Laboratory of Public Health of Rio Grande do Sul (Lacen-RS)	State Center for Health Surveillance of the Health Department of the State of Rio Grande do Sul (CEVS/SES-RS)	Aline Campos et al
EPI_ISL_983866	Central Laboratory of Public Health of Rio Grande do Sul (Lacen-RS)	State Center for Health Surveillance of the Health Department of the State of Rio Grande do Sul (CEVS/SES-RS)	Aline Campos et al
EPI_ISL_984620	Central Laboratory of Public Health of Rio Grande do Sul (Lacen-RS)	State Center for Health Surveillance of the Health Department of the State of Rio Grande do Sul (CEVS/SES-RS)	Aline Campos et al
EPI_ISL_984621	Central Laboratory of Public Health of Rio Grande do Sul (Lacen-RS)	State Center for Health Surveillance of the Health Department of the State of Rio Grande do Sul (CEVS/SES-RS)	Aline Campos et al
EPI_ISL_984619	Central Laboratory of Public Health of Rio Grande do Sul (Lacen-RS)	State Center for Health Surveillance of the Health Department of the State of Rio Grande do Sul (CEVS/SES-RS)	Aline Campos et al
EPI_ISL_943601	Central Laboratory of Public Health of Rio Grande do Sul (Lacen-RS)	State Center for Health Surveillance of the Health Department of the State of Rio Grande do Sul (CEVS/SES-RS)	Aline Campos et al
EPI_ISL_515155	National Institute for Communicable Diseases of the National Health Laboratory Service	National Institute for Communicable Diseases of the National Health Laboratory Service	Allam M et al

EPI_ISL_514386	National Institute for Communicable Diseases of the National Health Laboratory Service	National Institute for Communicable Diseases of the National Health Laboratory Service	Allam M et al
EPI_ISL_514408	National Institute for Communicable Diseases of the National Health Laboratory Service	National Institute for Communicable Diseases of the National Health Laboratory Service	Allam M et al
EPI_ISL_514417	National Institute for Communicable Diseases of the National Health Laboratory Service	National Institute for Communicable Diseases of the National Health Laboratory Service	Allam M et al
EPI_ISL_622982	National Health Laboratory Service	National Institute for Communicable Diseases of the National Health Laboratory Service	Allam M et al
EPI_ISL_622976	National Health Laboratory Service	National Institute for Communicable Diseases of the National Health Laboratory Service	Allam M et al
EPI_ISL_622977	National Health Laboratory Service	National Institute for Communicable Diseases of the National Health Laboratory Service	Allam M et al
EPI_ISL_450297	National Institute for Communicable Diseases of the National Health Laboratory Service	National Institute for Communicable Diseases of the National Health Laboratory Service	Allam M et al
EPI_ISL_470878	National Institute for Communicable Diseases of the National Health Laboratory Service	National Institute for Communicable Diseases of the National Health Laboratory Service	Allam M et al
EPI_ISL_470879	National Institute for Communicable Diseases of the National Health Laboratory Service	National Institute for Communicable Diseases of the National Health Laboratory Service	Allam M et al
EPI_ISL_853127	Toronto Invasive Bacterial Diseases Network	Ontario Institute for Cancer Research	Allison McGeer et al
EPI_ISL_853120	Toronto Invasive Bacterial Diseases Network	Ontario Institute for Cancer Research	Allison McGeer et al
EPI_ISL_755696	Toronto Invasive Bacterial Diseases Network	McMaster University	Allison McGeer et al
EPI_ISL_962186	Toronto Invasive Bacterial Diseases Network	McMaster University	Allison McGeer et al
EPI_ISL_962196	Toronto Invasive Bacterial Diseases Network	McMaster University	Allison McGeer et al
EPI_ISL_591267	Toronto Invasive Bacterial Diseases Network	McMaster University	Allison McGeer et al
EPI_ISL_489996	King Fahad Medical City	King Fahad Medical City	Alosaimi et al
EPI_ISL_489997	King Fahad Medical City	King Fahad Medical City	Alosaimi et al
EPI_ISL_489998	King Fahad Medical City	King Fahad Medical City	Alosaimi et al
EPI_ISL_490000	King Fahad Medical City	King Fahad Medical City	Alosaimi et al
EPI_ISL_490002	King Fahad Medical City	King Fahad Medical City	Alosaimi et al
EPI_ISL_490003	King Fahad Medical City	King Fahad Medical City	Alosaimi et al
EPI_ISL_490004	King Fahad Medical City	King Fahad Medical City	Alosaimi et al
EPI_ISL_788933	Centre de Recherche et de Formation en Infectiologie Guinée	TransVIHMI, IRD/INSERM/Monpellier University	Alpha Kabinet KEITA et al
EPI_ISL_788934	Centre de Recherche et de Formation en Infectiologie Guinée	TransVIHMI, IRD/INSERM/Monpellier University	Alpha Kabinet KEITA et al
EPI_ISL_788935	Centre de Recherche et de Formation en Infectiologie Guinée	TransVIHMI, IRD/INSERM/Monpellier University	Alpha Kabinet KEITA et al
EPI_ISL_788936	Centre de Recherche et de Formation en Infectiologie Guinée	TransVIHMI, IRD/INSERM/Monpellier University	Alpha Kabinet KEITA et al
EPI_ISL_788937	Centre de Recherche et de Formation en Infectiologie Guinée	TransVIHMI, IRD/INSERM/Monpellier University	Alpha Kabinet KEITA et al

EPI_ISL_788938	Centre de Recherche et de Formation en Infectiologie Guinée	TransVIHMI, IRD/INSERM/Monpellier University	Alpha Kabinet KEITA et al
EPI_ISL_632283	Communicable Disease Laboratory, Public Health Directorate	Communicable Disease Laboratory, Public Health Directorate	AlWasti et al
EPI_ISL_636973	Public Health Lab	Public Health Lab	Alwasti et al
EPI_ISL_632285	Communicable Disease Laboratory, Public Health Directorate	Communicable Disease Laboratory, Public Health Directorate	AlWasti et al
EPI_ISL_632262	Communicable Disease Laboratory, Public Health Directorate	Communicable Disease Laboratory, Public Health Directorate	AlWasti et al
EPI_ISL_632263	Communicable Disease Laboratory, Public Health Directorate	Communicable Disease Laboratory, Public Health Directorate	AlWasti et al
EPI_ISL_681309	Communicable Disease Laboratory, Public Health Directorate	Communicable Disease Laboratory, Public Health Directorate	Alwasti et al
EPI_ISL_682300	Communicable Disease Laboratory, Public Health Directorate	Communicable Disease Laboratory, Public Health Directorate	Alwasti et al
EPI_ISL_632269	Communicable Disease Laboratory, Public Health Directorate	Communicable Disease Laboratory, Public Health Directorate	AlWasti et al
EPI_ISL_632273	Communicable Disease Laboratory, Public Health Directorate	Communicable Disease Laboratory, Public Health Directorate	AlWasti et al
EPI_ISL_632274	Communicable Disease Laboratory, Public Health Directorate	Communicable Disease Laboratory, Public Health Directorate	AlWasti et al
EPI_ISL_632279	Communicable Disease Laboratory, Public Health Directorate	Communicable Disease Laboratory, Public Health Directorate	AlWasti et al
EPI_ISL_632252	Communicable Disease Laboratory, Public Health Directorate	Communicable Disease Laboratory, Public Health Directorate	AlWasti et al
EPI_ISL_632268	Communicable Disease Laboratory, Public Health Directorate	Communicable Disease Laboratory, Public Health Directorate	AlWasti et al
EPI_ISL_681315	Communicable Disease Laboratory, Public Health Directorate	Communicable Disease Laboratory, Public Health Directorate	Alwasti et al
EPI_ISL_684033	Communicable Disease Laboratory, Public Health Directorate	Communicable Disease Laboratory, Public Health Directorate	Alwasti et al
EPI_ISL_684032	Communicable Disease Laboratory, Public Health Directorate	Communicable Disease Laboratory, Public Health Directorate	Alwasti et al
EPI_ISL_682320	Communicable Disease Laboratory, Public Health Directorate	Communicable Disease Laboratory, Public Health Directorate	Alwasti et al
EPI_ISL_678270	Communicable Disease Laboratory, Public Health Directorate	Communicable Disease Laboratory, Public Health Directorate	Alwasti et al
EPI_ISL_1660462	Communicable Disease Laboratory, Public Health Directorate	Communicable Disease Laboratory, Public Health Directorate	Alwasti et al
EPI_ISL_1660461	Communicable Disease Laboratory, Public Health Directorate	Communicable Disease Laboratory, Public Health Directorate	Alwasti et al
EPI_ISL_1660463	Communicable Disease Laboratory, Public Health Directorate	Communicable Disease Laboratory, Public Health Directorate	Alwasti et al
EPI_ISL_1660464	Communicable Disease Laboratory, Public Health Directorate	Communicable Disease Laboratory, Public Health Directorate	Alwasti et al
EPI_ISL_1660465	Communicable Disease Laboratory, Public Health Directorate	Communicable Disease Laboratory, Public Health Directorate	Alwasti et al
EPI_ISL_1660466	Communicable Disease Laboratory, Public Health Directorate	Communicable Disease Laboratory, Public Health Directorate	Alwasti et al
EPI_ISL_1660467	Communicable Disease Laboratory, Public Health Directorate	Communicable Disease Laboratory, Public Health Directorate	Alwasti et al
EPI_ISL_678261	Communicable Disease Laboratory, Public Health Directorate	Communicable Disease Laboratory, Public Health Directorate	Alwasti et al
EPI_ISL_1660437	Communicable Disease Laboratory, Public Health Directorate	Communicable Disease Laboratory, Public Health Directorate	Alwasti et al
EPI_ISL_1660426	Communicable Disease Laboratory, Public Health Directorate	Communicable Disease Laboratory, Public Health Directorate	Alwasti et al
EPI_ISL_1660428	Communicable Disease Laboratory, Public Health Directorate	Communicable Disease Laboratory, Public Health Directorate	Alwasti et al

EPI_ISL_1660434	Communicable Disease Laboratory, Public Health Directorate	Communicable Disease Laboratory, Public Health Directorate	Alwasti et al
EPI_ISL_1660435	Communicable Disease Laboratory, Public Health Directorate	Communicable Disease Laboratory, Public Health Directorate	Alwasti et al
EPI_ISL_632266	Communicable Disease Laboratory, Public Health Directorate	Communicable Disease Laboratory, Public Health Directorate	AlWasti et al
EPI_ISL_632267	Communicable Disease Laboratory, Public Health Directorate	Communicable Disease Laboratory, Public Health Directorate	AlWasti et al
EPI_ISL_681301	Communicable Disease Laboratory, Public Health Directorate	Communicable Disease Laboratory, Public Health Directorate	Alwasti et al
EPI_ISL_682303	Communicable Disease Laboratory, Public Health Directorate	Communicable Disease Laboratory, Public Health Directorate	Alwasti et al
EPI_ISL_1660443	Communicable Disease Laboratory, Public Health Directorate	Communicable Disease Laboratory, Public Health Directorate	Alwasti et al
EPI_ISL_1660448	Communicable Disease Laboratory, Public Health Directorate	Communicable Disease Laboratory, Public Health Directorate	Alwasti et al
EPI_ISL_1660453	Communicable Disease Laboratory, Public Health Directorate	Communicable Disease Laboratory, Public Health Directorate	Alwasti et al
EPI_ISL_792525	Laboratorio del Hospital Interzonal General de Agudos Evita	Grupo de Genómica y Bioinformática del Instituto de Investigación de la Cadena Láctea CONICET-INTA on behalf of 'Proyecto Argentino Interinstitucional de genomica de SARS-CoV-2' (PAIS Consortium)	Amadio et al
EPI_ISL_1398364	Emam Ali Hospital	Razi Vaccine and Serum Research Institute	Amir Kaffashi et al
EPI_ISL_1398925	Emam Ali Hospital	Razi Vaccine and Serum Research Institute	Amir Kaffashi et al
EPI_ISL_1398937	Emam Ali Hospital	Razi Vaccine and Serum Research Institute	Amir Kaffashi et al
EPI_ISL_1239225	Kimberley Hospital, National Health Laboratory Services, Northern Cape, South Africa	National Institute for Communicable Diseases of the National Health Laboratory Service	Amoako DG et al
EPI_ISL_1239860	National Institute for Communicable Diseases,National Health Laboratory Services, Gauteng, South Africa	National Institute for Communicable Diseases of the National Health Laboratory Service	Amoako DG et al
EPI_ISL_425177	Public Health Ontario	Public Health Agency of Canada - National Microbiology Laboratory	Amrit S. Boese et al
EPI_ISL_534612	NHSGGC West of Scotland Specialist Virology Centre / MRC-University of Glasgow Centre for Virus Research	Wellcome Sanger Institute for the COVID-19 Genomics UK (COG-UK) consortium	Ana da Silva Filipe et al
EPI_ISL_534543	NHSGGC West of Scotland Specialist Virology Centre / MRC-University of Glasgow Centre for Virus Research	Wellcome Sanger Institute for the COVID-19 Genomics UK (COG-UK) consortium	Ana da Silva Filipe et al
EPI_ISL_802105	MSHS Clinical Microbiology Laboratories	MSHS Pathogen Surveillance Program	Ana S. Gonzalez-Reiche et al
EPI_ISL_802106	MSHS Clinical Microbiology Laboratories	MSHS Pathogen Surveillance Program	Ana S. Gonzalez-Reiche et al
EPI_ISL_1300937	MSHS Clinical Microbiology Laboratories	MSHS Pathogen Surveillance Program	Ana S. Gonzalez-Reiche et al
EPI_ISL_1301233	MSHS Clinical Microbiology Laboratories	MSHS Pathogen Surveillance Program	Ana S. Gonzalez-Reiche et al
EPI_ISL_1301238	MSHS Clinical Microbiology Laboratories	MSHS Pathogen Surveillance Program	Ana S. Gonzalez-Reiche et al
EPI_ISL_1395788	Laboratorio del Hospital Teresa Germani	Área de Secuenciación del Laboratorio de Virología del Hospital de Niños Dr. Ricardo Gutierrez on behalf of 'Proyecto Argentino Interinstitucional de genomica de SARS-CoV-2' (PAIS Consortium)	Anabel Luzio et al
EPI_ISL_1395807	Laboratorio del Hospital Teresa Germani	Área de Secuenciación del Laboratorio de Virología del Hospital de Niños Dr. Ricardo Gutierrez on behalf of 'Proyecto Argentino Interinstitucional de genomica de SARS-CoV-2' (PAIS Consortium)	Anabel Luzio et al
EPI_ISL_1081961	Stefan S. NicolauInstitute of Virology	Stefan S. NicolauInstitute of Virology	Anca Botezatu et al

EPI_ISL_1251275	Stefan S. NicolauInstitute of Virology	Stefan S. NicolauInstitute of Virology	Anca Botezatu et al
EPI_ISL_1508848	Medical Genetics Laboratory, Regional Centre of Medical Genetics, Emergency County Hospital Craiova	Medical Genetics Laboratory, Regional Centre of Medical Genetics, Emergency County Hospital Craiova	Anca-Lelia (Riza) Costache et al
EPI_ISL_1533052	Diagnostic and Research Center of Infectious Diseases, Medical Faculty, Andalas University	Diagnostic and Research Center of Infectious Diseases, Medical Faculty, Andalas University	Andani Eka Putra et al
EPI_ISL_1533065	Diagnostic and Research Center of Infectious Diseases, Medical Faculty, Andalas University	Diagnostic and Research Center of Infectious Diseases, Medical Faculty, Andalas University	Andani Eka Putra et al
EPI_ISL_1533066	Diagnostic and Research Center of Infectious Diseases, Medical Faculty, Andalas University	Diagnostic and Research Center of Infectious Diseases, Medical Faculty, Andalas University	Andani Eka Putra et al
EPI_ISL_1447336	Diagnostic and Research Center of Infectious Diseases, Medical Faculty, Andalas University	Diagnostic and Research Center of Infectious Diseases, Medical Faculty, Andalas University	Andani Eka Putra et al
EPI_ISL_539787	Institute of Microbiology, Universidad San Francisco de Quito	Institute of Microbiology, Universidad San Francisco de Quito	Andrea Macias et al
EPI_ISL_539788	Institute of Microbiology, Universidad San Francisco de Quito	Institute of Microbiology, Universidad San Francisco de Quito	Andrea Macias et al
EPI_ISL_697794	Institute of Microbiology, Universidad San Francisco de Quito	Institute of Microbiology, Universidad San Francisco de Quito	Andrea Macias et al
EPI_ISL_697795	Institute of Microbiology, Universidad San Francisco de Quito	Institute of Microbiology, Universidad San Francisco de Quito	Andrea Macias et al
EPI_ISL_949202	Departamento de Microbiología, CDB, Hospital Clínic, Barcelona	SeqCOVID-SPAIN consortium/IBV(CSIC)	Andrea Vergara et al
EPI_ISL_1469196	Klinisk mikrobiologi, Region Västerbotten	CBRN Defence and Security, Swedish Defence Research Agency	Andreas Sjödin et al
EPI_ISL_1469197	Klinisk mikrobiologi, Region Västerbotten	CBRN Defence and Security, Swedish Defence Research Agency	Andreas Sjödin et al
EPI_ISL_445259	CLINICA LAS CONDES S.A.	Instituto de Salud Publica de Chile	Andrés E Castillo et al
EPI_ISL_445260	CLINICA ALEMANA DE SANTIAGO S.A.	Instituto de Salud Publica de Chile	Andrés E Castillo et al
EPI_ISL_445318	C.DE SALUD FAMILIAR PABLO NERUDA	Instituto de Salud Publica de Chile	Andrés E Castillo et al
EPI_ISL_445247	UNIVERSIDAD DE LOS ANDES	Instituto de Salud Publica de Chile	Andrés E Castillo et al
EPI_ISL_445367	ASISTENCIA PUBLICA DR.ALEJANDRO DEL RIO	Instituto de Salud Publica de Chile	Andrés E Castillo et al
EPI_ISL_1091784	LDSP	Universidad Nacional de Colombia - Laboratorio Genómico One Health	Andres F. Cardona-Rios et al
EPI_ISL_1091785	LDSP	Universidad Nacional de Colombia - Laboratorio Genómico One Health	Andres F. Cardona-Rios et al
EPI_ISL_1091786	LDSP	Universidad Nacional de Colombia - Laboratorio Genómico One Health	Andres F. Cardona-Rios et al
EPI_ISL_1629760	LABMÉDICO	Universidad Nacional de Colombia - Laboratorio Genómico One Health	Andres F. Cardona-Rios et al
EPI_ISL_480321	Laboratorio Clínico San José	Charité Virology-University of Costa Rica	Andres Moreira-Soto et al
EPI_ISL_480326	Hospital Nacional de Niños	Charité Virology-University of Costa Rica	Andres Moreira-Soto et al
EPI_ISL_480327	Hospital Nacional de Niños	Charité Virology-University of Costa Rica	Andres Moreira-Soto et al
EPI_ISL_480328	Laboratorio LABIN	Charité Virology-University of Costa Rica	Andres Moreira-Soto et al
EPI_ISL_733077	HELIX LLC	WHO National Influenza Centre Russian Federation	Andrey Komissarov et al
EPI_ISL_733078	HELIX LLC	WHO National Influenza Centre Russian Federation	Andrey Komissarov et al

EPI_ISL_733151	HELIX LLC	WHO National Influenza Centre Russian Federation	Andrey Komissarov et al
EPI_ISL_733433	HELIX LLC	WHO National Influenza Centre Russian Federation	Andrey Komissarov et al
EPI_ISL_1400527	HELIX LLC	WHO National Influenza Centre Russian Federation	Andrey Komissarov et al
EPI_ISL_733452	HELIX LLC	WHO National Influenza Centre Russian Federation	Andrey Komissarov et al
EPI_ISL_733462	WHO National Influenza Centre Russian Federation	WHO National Influenza Centre Russian Federation	Andrey Komissarov et al
EPI_ISL_1372409	Pathogenic Microorganisms Variability Laboratory	WHO National Influenza Centre Russian Federation	Andrey Komissarov et al
EPI_ISL_1491590	Pathogenic Microorganisms Variability Laboratory	WHO National Influenza Centre Russian Federation	Andrey Komissarov et al
EPI_ISL_733166	Pathogenic Microorganisms Variability Laboratory	WHO National Influenza Centre Russian Federation	Andrey Komissarov et al
EPI_ISL_602423	HELIX LLC	WHO National Influenza Centre Russian Federation	Andrey Komissarov et al
EPI_ISL_733098	HELIX LLC	WHO National Influenza Centre Russian Federation	Andrey Komissarov et al
EPI_ISL_733444	HELIX LLC	WHO National Influenza Centre Russian Federation	Andrey Komissarov et al
EPI_ISL_733300	WHO National Influenza Centre Russian Federation	WHO National Influenza Centre Russian Federation	Andrey Komissarov et al
EPI_ISL_596324	Pathogenic Microorganisms Variability Laboratory	WHO National Influenza Centre Russian Federation	Andrey Komissarov et al
EPI_ISL_596337	Pathogenic Microorganisms Variability Laboratory	WHO National Influenza Centre Russian Federation	Andrey Komissarov et al
EPI_ISL_596338	Pathogenic Microorganisms Variability Laboratory	WHO National Influenza Centre Russian Federation	Andrey Komissarov et al
EPI_ISL_596347	Pathogenic Microorganisms Variability Laboratory	WHO National Influenza Centre Russian Federation	Andrey Komissarov et al
EPI_ISL_596348	Pathogenic Microorganisms Variability Laboratory	WHO National Influenza Centre Russian Federation	Andrey Komissarov et al
EPI_ISL_596352	Pathogenic Microorganisms Variability Laboratory	WHO National Influenza Centre Russian Federation	Andrey Komissarov et al
EPI_ISL_770670	Pathogenic Microorganisms Variability Laboratory	WHO National Influenza Centre Russian Federation	Andrey Komissarov et al
EPI_ISL_602408	HELIX LLC	WHO National Influenza Centre Russian Federation	Andrey Komissarov et al
EPI_ISL_1372355	HELIX LLC	WHO National Influenza Centre Russian Federation	Andrey Komissarov et al
EPI_ISL_596266	WHO National Influenza Centre Russian Federation	WHO National Influenza Centre Russian Federation	Andrey Komissarov et al
EPI_ISL_733008	WHO National Influenza Centre Russian Federation	WHO National Influenza Centre Russian Federation	Andrey Komissarov et al
EPI_ISL_733015	WHO National Influenza Centre Russian Federation	WHO National Influenza Centre Russian Federation	Andrey Komissarov et al
EPI_ISL_733016	WHO National Influenza Centre Russian Federation	WHO National Influenza Centre Russian Federation	Andrey Komissarov et al
EPI_ISL_733018	WHO National Influenza Centre Russian Federation	WHO National Influenza Centre Russian Federation	Andrey Komissarov et al
EPI_ISL_733021	WHO National Influenza Centre Russian Federation	WHO National Influenza Centre Russian Federation	Andrey Komissarov et al
EPI_ISL_733314	HELIX LLC	WHO National Influenza Centre Russian Federation	Andrey Komissarov et al
EPI_ISL_524015	WHO National Influenza Centre Russian Federation	WHO National Influenza Centre Russian Federation	Andrey Komissarov et al
EPI_ISL_1372283	WHO National Influenza Centre Russian Federation	WHO National Influenza Centre Russian Federation	Andrey Komissarov et al

EPI_ISL_1372287	WHO National Influenza Centre Russian Federation	WHO National Influenza Centre Russian Federation	Andrey Komissarov et al
EPI_ISL_1039159	WHO National Influenza Centre Russian Federation	WHO National Influenza Centre Russian Federation	Andrey Komissarov et al
EPI_ISL_732973	WHO National Influenza Centre Russian Federation	WHO National Influenza Centre Russian Federation	Andrey Komissarov et al
EPI_ISL_732972	WHO National Influenza Centre Russian Federation	WHO National Influenza Centre Russian Federation	Andrey Komissarov et al
EPI_ISL_1372345	HELIX LLC	WHO National Influenza Centre Russian Federation	Andrey Komissarov et al
EPI_ISL_1372347	HELIX LLC	WHO National Influenza Centre Russian Federation	Andrey Komissarov et al
EPI_ISL_1372379	HELIX LLC	WHO National Influenza Centre Russian Federation	Andrey Komissarov et al
EPI_ISL_1372392	HELIX LLC	WHO National Influenza Centre Russian Federation	Andrey Komissarov et al
EPI_ISL_1491700	HELIX LLC	WHO National Influenza Centre Russian Federation	Andrey Komissarov et al
EPI_ISL_1664642	HELIX LLC	WHO National Influenza Centre Russian Federation	Andrey Komissarov et al
EPI_ISL_1664718	HELIX LLC	WHO National Influenza Centre Russian Federation	Andrey Komissarov et al
EPI_ISL_1664720	HELIX LLC	WHO National Influenza Centre Russian Federation	Andrey Komissarov et al
EPI_ISL_1664726	HELIX LLC	WHO National Influenza Centre Russian Federation	Andrey Komissarov et al
EPI_ISL_1652545	HELIX LLC	WHO National Influenza Centre Russian Federation	Andrey Komissarov et al
EPI_ISL_1652551	HELIX LLC	WHO National Influenza Centre Russian Federation	Andrey Komissarov et al
EPI_ISL_1652560	HELIX LLC	WHO National Influenza Centre Russian Federation	Andrey Komissarov et al
EPI_ISL_1652563	HELIX LLC	WHO National Influenza Centre Russian Federation	Andrey Komissarov et al
EPI_ISL_1652577	HELIX LLC	WHO National Influenza Centre Russian Federation	Andrey Komissarov et al
EPI_ISL_1652584	HELIX LLC	WHO National Influenza Centre Russian Federation	Andrey Komissarov et al
EPI_ISL_1652601	HELIX LLC	WHO National Influenza Centre Russian Federation	Andrey Komissarov et al
EPI_ISL_1652611	HELIX LLC	WHO National Influenza Centre Russian Federation	Andrey Komissarov et al
EPI_ISL_1652643	HELIX LLC	WHO National Influenza Centre Russian Federation	Andrey Komissarov et al
EPI_ISL_596301	HELIX LCC	WHO National Influenza Centre Russian Federation	Andrey Komissarov et al
EPI_ISL_596279	HELIX LCC	WHO National Influenza Centre Russian Federation	Andrey Komissarov et al
EPI_ISL_1664772	UMMC-Health	WHO National Influenza Centre Russian Federation	Andrey Komissarov et al
EPI_ISL_732996	UMMC-Health	WHO National Influenza Centre Russian Federation	Andrey Komissarov et al
EPI_ISL_430091	WHO National Influenza Centre Russian Federation	WHO National Influenza Centre Russian Federation	Andrey Komissarov et al
EPI_ISL_450277	WHO National Influenza Centre Russian Federation	WHO National Influenza Centre Russian Federation	Andrey Komissarov et al
EPI_ISL_733121	HELIX LLC	WHO National Influenza Centre Russian Federation	Andrey Komissarov et al
EPI_ISL_1300661	RSUD Pasarminggu	Stem Cell Lab, Universitas Pembangunan Nasional Veteran Jakarta (UPNVJ)	Andri Pramesyanti Pramono et al

EPI_ISL_1300540	RSUD Pasarminggu	Stem Cell Lab, Universitas Pembangunan Nasional Veteran Jakarta (UPNVJ)	Andri Pramesyanti Pramono et al
EPI_ISL_833391	Klinik Apotek Dein, Jakarta, Indonesia	Biosafety Level-3 Laboratory, Indonesian Institute of Sciences (LIPI)	Andri Wardiana et al
EPI_ISL_833508	Klinik Apotek Dein, Jakarta, Indonesia	Biosafety Level-3 Laboratory, Indonesian Institute of Sciences (LIPI)	Andri Wardiana et al
EPI_ISL_1250844	Tanjungpura University Hospital	Tanjungpura University Hospital	Andriani et al
EPI_ISL_912354	Fondation Congolaise pour la recherche medicale (FCRM), Francine Ntoumi	NGS Competence Center Tuebingen, Institut für Medizinische Mikrobiologie und Hygiene, Universitaetsklinikum Tübingen	Angel Angelov et al
EPI_ISL_912361	Fondation Congolaise pour la recherche medicale (FCRM), Francine Ntoumi	NGS Competence Center Tuebingen, Institut für Medizinische Mikrobiologie und Hygiene, Universitaetsklinikum Tübingen	Angel Angelov et al
EPI_ISL_912363	Fondation Congolaise pour la recherche medicale (FCRM), Francine Ntoumi	NGS Competence Center Tuebingen, Institut für Medizinische Mikrobiologie und Hygiene, Universitaetsklinikum Tübingen	Angel Angelov et al
EPI_ISL_912367	Fondation Congolaise pour la recherche medicale (FCRM), Francine Ntoumi	NGS Competence Center Tuebingen, Institut für Medizinische Mikrobiologie und Hygiene, Universitaetsklinikum Tübingen	Angel Angelov et al
EPI_ISL_912368	Fondation Congolaise pour la recherche medicale (FCRM), Francine Ntoumi	NGS Competence Center Tuebingen, Institut für Medizinische Mikrobiologie und Hygiene, Universitaetsklinikum Tübingen	Angel Angelov et al
EPI_ISL_912375	Fondation Congolaise pour la recherche medicale (FCRM), Francine Ntoumi	NGS Competence Center Tuebingen, Institut für Medizinische Mikrobiologie und Hygiene, Universitaetsklinikum Tübingen	Angel Angelov et al
EPI_ISL_912376	Fondation Congolaise pour la recherche medicale (FCRM), Francine Ntoumi	NGS Competence Center Tuebingen, Institut für Medizinische Mikrobiologie und Hygiene, Universitaetsklinikum Tübingen	Angel Angelov et al
EPI_ISL_912377	Fondation Congolaise pour la recherche medicale (FCRM), Francine Ntoumi	NGS Competence Center Tuebingen, Institut für Medizinische Mikrobiologie und Hygiene, Universitaetsklinikum Tübingen	Angel Angelov et al
EPI_ISL_912380	Fondation Congolaise pour la recherche medicale (FCRM), Francine Ntoumi	NGS Competence Center Tuebingen, Institut für Medizinische Mikrobiologie und Hygiene, Universitaetsklinikum Tübingen	Angel Angelov et al
EPI_ISL_912387	Fondation Congolaise pour la recherche medicale (FCRM), Francine Ntoumi	NGS Competence Center Tuebingen, Institut für Medizinische Mikrobiologie und Hygiene, Universitaetsklinikum Tübingen	Angel Angelov et al
EPI_ISL_912388	Fondation Congolaise pour la recherche medicale (FCRM), Francine Ntoumi	NGS Competence Center Tuebingen, Institut für Medizinische Mikrobiologie und Hygiene, Universitaetsklinikum Tübingen	Angel Angelov et al
EPI_ISL_912389	Fondation Congolaise pour la recherche medicale (FCRM), Francine Ntoumi	NGS Competence Center Tuebingen, Institut für Medizinische Mikrobiologie und Hygiene, Universitaetsklinikum Tübingen	Angel Angelov et al
EPI_ISL_912394	Fondation Congolaise pour la recherche medicale (FCRM), Francine Ntoumi	NGS Competence Center Tuebingen, Institut für Medizinische Mikrobiologie und Hygiene, Universitaetsklinikum Tübingen	Angel Angelov et al
EPI_ISL_581487	Fondation Congolaise pour la recherche medicale (FCRM)	NGS Competence Center Tübingen, Institut für Medizinische Mikrobiologie und Hygiene, Universitätsklinikum Tübingen	Angel Angelov et al
EPI_ISL_581489	Fondation Congolaise pour la recherche medicale (FCRM)	NGS Competence Center Tübingen, Institut für Medizinische Mikrobiologie und Hygiene, Universitätsklinikum Tübingen	Angel Angelov et al
EPI_ISL_581455	Fondation Congolaise pour la recherche medicale (FCRM)	NGS Competence Center Tübingen, Institut für Medizinische Mikrobiologie und Hygiene, Universitätsklinikum Tübingen	Angel Angelov et al
EPI_ISL_581492	Fondation Congolaise pour la recherche medicale (FCRM)	NGS Competence Center Tübingen, Institut für Medizinische Mikrobiologie und Hygiene, Universitätsklinikum Tübingen	Angel Angelov et al
EPI_ISL_581493	Fondation Congolaise pour la recherche medicale (FCRM)	NGS Competence Center Tübingen, Institut für Medizinische Mikrobiologie und Hygiene, Universitätsklinikum Tübingen	Angel Angelov et al
EPI_ISL_485810	Institut für Virologie und Epidemiologie der Viruskrankheiten, Universitätsklinikum Tübingen	NGS Competence Center Tübingen, Institut für Medizinische Mikrobiologie und Hygiene, Universitätsklinikum Tübingen	Angelov et al.

EPI_ISL_1020195	Melania Hospital, Bogor , West Java	Biosafety Level-3 Laboratory, Indonesian Institute of Sciences (LIPI)	Anggia Prasetyoputri et al
EPI_ISL_1020192	Melania Hospital, Bagor , west Java	Biosafety Level-3 Laboratory, Indonesian Institute of Sciences (LIPI)	Anik Budhi Dharmayanthi et al
EPI_ISL_1020197	Dinkes, Bogor, West Java	Biosafety Level-3 Laboratory, Indonesian Institute of Sciences (LIPI)	Anik Budhi Dharmayanthi et al
EPI_ISL_419562	Laboratoire National de Santé, Microbiology, Virology	Laboratoire National de Santé, Microbiology, Epidemiology and Microbial Genomics	Anke Wienecke-Baldacchino et al
EPI_ISL_1383410	Laboratoire national de sante, Microbiology, Virology	Laboratoire national de sante, Microbiology, Microbial Genomics Platform	Anke Wienecke-Baldacchino et al
EPI_ISL_739793	Laboratoire national de santé, Microbiology, Virology	Laboratoire national de santé, Microbiology, Microbial Genomics Platform	Anke Wienecke-Baldacchino et al
EPI_ISL_419565	Laboratoire National de Santé, Microbiology, Virology	Laboratoire National de Santé, Microbiology, Epidemiology and Microbial Genomics	Anke Wienecke-Baldacchino et al
EPI_ISL_1383771	Laboratoire national de sante, Microbiology, Virology	Laboratoire national de sante, Microbiology, Microbial Genomics Platform	Anke Wienecke-Baldacchino et al
EPI_ISL_739835	Laboratoire national de santé, Microbiology, Virology	Laboratoire national de santé, Microbiology, Microbial Genomics Platform	Anke Wienecke-Baldacchino et al
EPI_ISL_911195	Laboratoire national de sante, Microbiology, Virology	Laboratoire national de sante, Microbiology, Microbial Genomics Platform	Anke Wienecke-Baldacchino et al
EPI_ISL_739880	Laboratoire national de santé, Microbiology, Virology	Laboratoire national de santé, Microbiology, Epidemiology and Microbial Genomics	Anke Wienecke-Baldacchino et al
EPI_ISL_1384017	Hospital Center Emile Mayrisch	Laboratoire national de sante, Microbiology, Microbial Genomics Platform	Anke Wienecke-Baldacchino et al
EPI_ISL_1383653	Laboratoire national de sante, Microbiology, Virology	Laboratoire national de sante, Microbiology, Microbial Genomics Platform	Anke Wienecke-Baldacchino et al
EPI_ISL_739934	Laboratoire national de santé, Microbiology, Virology	Laboratoire national de santé, Microbiology, Microbial Genomics Platform	Anke Wienecke-Baldacchino et al
EPI_ISL_1384349	Laboratoires d'analyses medicales - Ketterthill	Laboratoire national de sante, Microbiology, Microbial Genomics Platform	Anke Wienecke-Baldacchino et al
EPI_ISL_739947	Laboratoire national de santé, Microbiology, Virology	Laboratoire national de santé, Microbiology, Microbial Genomics Platform	Anke Wienecke-Baldacchino et al
EPI_ISL_739956	Laboratoire national de santé, Microbiology, Virology	Laboratoire national de santé, Microbiology, Microbial Genomics Platform	Anke Wienecke-Baldacchino et al
EPI_ISL_739977	Laboratoire national de santé, Microbiology, Virology	Laboratoire national de santé, Microbiology, Microbial Genomics Platform	Anke Wienecke-Baldacchino et al
EPI_ISL_739978	Laboratoire national de santé, Microbiology, Virology	Laboratoire national de santé, Microbiology, Microbial Genomics Platform	Anke Wienecke-Baldacchino et al
EPI_ISL_739987	Laboratoire national de santé, Microbiology, Virology	Laboratoire national de santé, Microbiology, Microbial Genomics Platform	Anke Wienecke-Baldacchino et al
EPI_ISL_740013	Laboratoire national de santé, Microbiology, Virology	Laboratoire national de santé, Microbiology, Microbial Genomics Platform	Anke Wienecke-Baldacchino et al
EPI_ISL_1498404	BioneXt Lab	Laboratoire national de sante, Microbiology, Microbial Genomics Platform	Anke Wienecke-Baldacchino et al
EPI_ISL_740098	Laboratoire national de santé, Microbiology, Virology	Laboratoire national de santé, Microbiology, Microbial Genomics Platform	Anke Wienecke-Baldacchino et al
EPI_ISL_740129	Laboratoire national de santé, Microbiology, Virology	Laboratoire national de santé, Microbiology, Epidemiology and Microbial Genomics	Anke Wienecke-Baldacchino et al
EPI_ISL_740202	Laboratoire national de santé, Microbiology, Virology	Laboratoire national de santé, Microbiology, Microbial Genomics Platform	Anke Wienecke-Baldacchino et al
EPI_ISL_1383541	Laboratoire national de sante, Microbiology, Virology	Laboratoire national de sante, Microbiology, Microbial Genomics Platform	Anke Wienecke-Baldacchino et al
EPI_ISL_740259	Laboratoire national de santé, Microbiology, Virology	Laboratoire national de santé, Microbiology, Microbial Genomics Platform	Anke Wienecke-Baldacchino et al
EPI_ISL_421735	Laboratoire National de Sante, Microbiology, Virology	Laboratoire National de Sante, Microbiology, Epidemiology and Microbial Genomics	Anke Wienecke-Baldacchino et al

EPI_ISL_740289	Laboratoire national de santé, Microbiology, Virology	Laboratoire national de santé, Microbiology, Microbial Genomics Platform	Anke Wienecke-Baldacchino et al
EPI_ISL_459896	Laboratoire National de Sante, Microbiology, Virology	Laboratoire National de Sante, Microbiology, Epidemiology and Microbial Genomics	Anke Wienecke-Baldacchino et al
EPI_ISL_459898	Laboratoire National de Sante, Microbiology, Virology	Laboratoire National de Sante, Microbiology, Epidemiology and Microbial Genomics	Anke Wienecke-Baldacchino et al
EPI_ISL_740408	Laboratoire national de santé, Microbiology, Virology	Laboratoire national de santé, Microbiology, Microbial Genomics Platform	Anke Wienecke-Baldacchino et al
EPI_ISL_740413	Laboratoire national de santé, Microbiology, Virology	Laboratoire national de santé, Microbiology, Microbial Genomics Platform	Anke Wienecke-Baldacchino et al
EPI_ISL_740486	Laboratoire national de santé, Microbiology, Virology	Laboratoire national de santé, Microbiology, Microbial Genomics Platform	Anke Wienecke-Baldacchino et al
EPI_ISL_434509	Laboratoire National de Sante, Microbiology, Virology	Laboratoire National de Sante, Microbiology, Epidemiology and Microbial Genomics	Anke Wienecke-Baldacchino et al
EPI_ISL_744135	Laboratoire national de santé, Microbiology, Virology	Laboratoire national de santé, Microbiology, Microbial Genomics Platform	Anke Wienecke-Baldacchino et al
EPI_ISL_744139	Laboratoire national de santé, Microbiology, Virology	Laboratoire national de santé, Microbiology, Microbial Genomics Platform	Anke Wienecke-Baldacchino et al
EPI_ISL_1522973	Laboratoire national de sante, Microbiology, Virology	Laboratoire national de sante, Microbiology, Microbial Genomics Platform	Anke Wienecke-Baldacchino et al
EPI_ISL_429738	Laboratoire National de Sante, Microbiology, Virology	Laboratoire National de Sante, Microbiology, Epidemiology and Microbial Genomics	Anke Wienecke-Baldacchino et al
EPI_ISL_744245	Laboratoire national de santé, Microbiology, Virology	Laboratoire national de santé, Microbiology, Microbial Genomics Platform	Anke Wienecke-Baldacchino et al
EPI_ISL_429774	Laboratoire National de Sante, Microbiology, Virology	Laboratoire National de Sante, Microbiology, Epidemiology and Microbial Genomics	Anke Wienecke-Baldacchino et al
EPI_ISL_744291	Laboratoire national de santé, Microbiology, Virology	Laboratoire national de santé, Microbiology, Microbial Genomics Platform	Anke Wienecke-Baldacchino et al
EPI_ISL_1383729	Laboratoire national de sante, Microbiology, Virology	Laboratoire national de sante, Microbiology, Microbial Genomics Platform	Anke Wienecke-Baldacchino et al
EPI_ISL_429731	Laboratoire National de Sante, Microbiology, Virology	Laboratoire National de Sante, Microbiology, Epidemiology and Microbial Genomics	Anke Wienecke-Baldacchino et al
EPI_ISL_429743	Laboratoire National de Sante, Microbiology, Virology	Laboratoire National de Sante, Microbiology, Epidemiology and Microbial Genomics	Anke Wienecke-Baldacchino et al
EPI_ISL_1384478	Laboratoires d'analyses medicales - Ketterthill	Laboratoire national de sante, Microbiology, Microbial Genomics Platform	Anke Wienecke-Baldacchino et al
EPI_ISL_459901	Laboratoire National de Sante, Microbiology, Virology	Laboratoire National de Sante, Microbiology, Epidemiology and Microbial Genomics	Anke Wienecke-Baldacchino et al
EPI_ISL_1384382	Laboratoires d'analyses medicales - Ketterthill	Laboratoire national de sante, Microbiology, Microbial Genomics Platform	Anke Wienecke-Baldacchino et al
EPI_ISL_1384184	Laboratoires d'analyses medicales - Ketterthill	Laboratoire national de sante, Microbiology, Microbial Genomics Platform	Anke Wienecke-Baldacchino et al
EPI_ISL_744480	Laboratoire national de santé, Microbiology, Virology	Laboratoire national de santé, Microbiology, Microbial Genomics Platform	Anke Wienecke-Baldacchino et al
EPI_ISL_1384156	Laboratoire national de sante, Microbiology, Virology	Laboratoire national de sante, Microbiology, Microbial Genomics Platform	Anke Wienecke-Baldacchino et al
EPI_ISL_744561	Laboratoire national de santé, Microbiology, Virology	Laboratoire national de santé, Microbiology, Microbial Genomics Platform	Anke Wienecke-Baldacchino et al
EPI_ISL_744574	Laboratoire national de santé, Microbiology, Virology	Laboratoire national de santé, Microbiology, Microbial Genomics Platform	Anke Wienecke-Baldacchino et al
EPI_ISL_744577	Laboratoire national de santé, Microbiology, Virology	Laboratoire national de santé, Microbiology, Microbial Genomics Platform	Anke Wienecke-Baldacchino et al
EPI_ISL_445062	Laboratoire National de Sante, Microbiology, Virology	Laboratoire National de Sante, Microbiology, Epidemiology and Microbial Genomics	Anke Wienecke-Baldacchino et al

EPI_ISL_459906	Laboratoire National de Sante, Microbiology, Virology	Laboratoire National de Sante, Microbiology, Epidemiology and Microbial Genomics	Anke Wienecke-Baldacchino et al
EPI_ISL_428940	Laboratoire National de Sante, Microbiology, Virology	Laboratoire National de Sante, Microbiology, Epidemiology and Microbial Genomics	Anke Wienecke-Baldacchino et al
EPI_ISL_1498386	BioneXt Lab	Laboratoire national de sante, Microbiology, Microbial Genomics Platform	Anke Wienecke-Baldacchino et al
EPI_ISL_1522970	Laboratoire national de sante, Microbiology, Virology	Laboratoire national de sante, Microbiology, Microbial Genomics Platform	Anke Wienecke-Baldacchino et al
EPI_ISL_1384419	Laboratoires d'analyses medicales - Ketterthill	Laboratoire national de sante, Microbiology, Microbial Genomics Platform	Anke Wienecke-Baldacchino et al
EPI_ISL_744674	Laboratoire national de santé, Microbiology, Virology	Laboratoire national de santé, Microbiology, Microbial Genomics Platform	Anke Wienecke-Baldacchino et al
EPI_ISL_744766	Laboratoire national de santé, Microbiology, Virology	Laboratoire national de santé, Microbiology, Microbial Genomics Platform	Anke Wienecke-Baldacchino et al
EPI_ISL_744773	Laboratoire national de santé, Microbiology, Virology	Laboratoire national de santé, Microbiology, Microbial Genomics Platform	Anke Wienecke-Baldacchino et al
EPI_ISL_1384207	Laboratoires d'analyses medicales - Ketterthill	Laboratoire national de sante, Microbiology, Microbial Genomics Platform	Anke Wienecke-Baldacchino et al
EPI_ISL_1384442	Laboratoires d'analyses medicales - Ketterthill	Laboratoire national de sante, Microbiology, Microbial Genomics Platform	Anke Wienecke-Baldacchino et al
EPI_ISL_744958	Laboratoire national de santé, Microbiology, Virology	Laboratoire national de santé, Microbiology, Microbial Genomics Platform	Anke Wienecke-Baldacchino et al
EPI_ISL_910820	Laboratoire national de sante, Microbiology, Virology	Laboratoire national de sante, Microbiology, Microbial Genomics Platform	Anke Wienecke-Baldacchino et al
EPI_ISL_745023	Laboratoire national de santé, Microbiology, Virology	Laboratoire national de santé, Microbiology, Microbial Genomics Platform	Anke Wienecke-Baldacchino et al
EPI_ISL_416480	R. G. Lugar Center for Public Health Research, National Center for Disease Control and Public Health (NCDC) of Georgia.	R. G. Lugar Center for Public Health Research, National Center for Disease Control and Public Health (NCDC) of Georgia.	Ann Machablishvili et al
EPI_ISL_935788	Cadham Provincial laboratory	National Microbiology Laboratory (NML)	Anna Majer et al
EPI_ISL_935789	Cadham Provincial laboratory	National Microbiology Laboratory (NML)	Anna Majer et al
EPI_ISL_935828	Cadham Provincial laboratory	National Microbiology Laboratory (NML)	Anna Majer et al
EPI_ISL_935834	Cadham Provincial laboratory	National Microbiology Laboratory (NML)	Anna Majer et al
EPI_ISL_935843	Cadham Provincial laboratory	National Microbiology Laboratory (NML)	Anna Majer et al
EPI_ISL_935847	Cadham Provincial laboratory	National Microbiology Laboratory (NML)	Anna Majer et al
EPI_ISL_935848	Cadham Provincial laboratory	National Microbiology Laboratory (NML)	Anna Majer et al
EPI_ISL_935872	Cadham Provincial laboratory	National Microbiology Laboratory (NML)	Anna Majer et al
EPI_ISL_935873	Cadham Provincial laboratory	National Microbiology Laboratory (NML)	Anna Majer et al
EPI_ISL_935875	Cadham Provincial laboratory	National Microbiology Laboratory (NML)	Anna Majer et al
EPI_ISL_1594113	MB-Cadham Provincial laboratory	National Microbiology Laboratory (NML)	Anna Majer et al
EPI_ISL_1594051	MB-Cadham Provincial laboratory	National Microbiology Laboratory (NML)	Anna Majer et al
EPI_ISL_1258081	MB-Cadham Provincial laboratory	National Microbiology Laboratory (NML)	Anna Majer et al
EPI_ISL_1258090	MB-Cadham Provincial laboratory	National Microbiology Laboratory (NML)	Anna Majer et al
EPI_ISL_1594071	MB-Cadham Provincial laboratory	National Microbiology Laboratory (NML)	Anna Majer et al

EPI_ISL_582342	Cadham Provincial Laboratory	National Microbiology Laboratory (NML)	Anna Majer et al
EPI_ISL_1164956	Hôpital Georges L. Dumont	National Microbiology Laboratory (NML)	Anna Majer et al
EPI_ISL_1272270	NB-Hôpital Georges L. Dumont	National Microbiology Laboratory (NML)	Anna Majer et al
EPI_ISL_1272256	NB-Hôpital Georges L. Dumont	National Microbiology Laboratory (NML)	Anna Majer et al
EPI_ISL_1588150	NB-Hôpital Georges L. Dumont	National Microbiology Laboratory (NML)	Anna Majer et al
EPI_ISL_1588200	NB-Hôpital Georges L. Dumont	National Microbiology Laboratory (NML)	Anna Majer et al
EPI_ISL_1588269	NB-Hôpital Georges L. Dumont	National Microbiology Laboratory (NML)	Anna Majer et al
EPI_ISL_481756	Dr. Georges-L.-Dumont University Hospital Centre	National Microbiology Laboratory	Anna Majer et al
EPI_ISL_1278151	NL-Dr. Leonard A. Miller Centre for Health Services	National Microbiology Laboratory (NML)	Anna Majer et al
EPI_ISL_1055752	QEII Health Sciences Centre	National Microbiology Laboratory (NML)	Anna Majer et al
EPI_ISL_1666730	NS-QEII Health Sciences Centre	National Microbiology Laboratory (NML)	Anna Majer et al
EPI_ISL_1055479	QEII Health Sciences Centre	National Microbiology Laboratory (NML)	Anna Majer et al
EPI_ISL_856714	Infectious Diseases Unit, Department of Internal Medicine, Azienda Ospedaliera-Universitaria di Padova	Laboratory of Infectious Diseases, Department of Biomedical and Clinical Sciences L. Sacco, University of Milan	Anna Maria Cattelan et al
EPI_ISL_856715	Infectious Diseases Unit, Department of Internal Medicine, Azienda Ospedaliera-Universitaria di Padova	Laboratory of Infectious Diseases, Department of Biomedical and Clinical Sciences L. Sacco, University of Milan	Anna Maria Cattelan et al
EPI_ISL_856721	Infectious Diseases Unit, Department of Internal Medicine, Azienda Ospedaliera-Universitaria di Padova	Laboratory of Infectious Diseases, Department of Biomedical and Clinical Sciences L. Sacco, University of Milan	Anna Maria Cattelan et al
EPI_ISL_406034	California Department of Public Health	Pathogen Discovery, Respiratory Viruses Branch, Division of Viral Diseases, Centers for Disease Control and Prevention	Anna Uehara et al
EPI_ISL_406036	California Department of Public Health	Pathogen Discovery, Respiratory Viruses Branch, Division of Viral Diseases, Centers for Disease Control and Prevention	Anna Uehara et al
EPI_ISL_419560	FL Bureau of Public Health Laboratories-Tampa	Pathogen Discovery, Respiratory Viruses Branch, Division of Viral Diseases, Centers for Disease Control and Prevention	Anna Uehara et al
EPI_ISL_454481	Karolinska Universitetslaboratoriet	The Public Health Agency of Sweden	Anna-Malin Linde et al
EPI_ISL_455902	Halmstad klinisk mikrobiologi	The Public Health Agency of Sweden	Anna-Malin Linde et al
EPI_ISL_534224	Universitetssjukhuset i Linkoping	The Public Health Agency of Sweden	Anna-Malin Linde et al
EPI_ISL_582789	Orebro klinisk mikrobiologi	The Public Health Agency of Sweden	Anna-Malin Linde et al
EPI_ISL_582791	Orebro klinisk mikrobiologi	The Public Health Agency of Sweden	Anna-Malin Linde et al
EPI_ISL_623085	Klinisk mikrobiologi, Skanes universitetssjukhus, Lund	The Public Health Agency of Sweden	Anna-Malin Linde et al
EPI_ISL_648189	The Public Health Agency of Sweden	The Public Health Agency of Sweden	Anna-Malin Linde et al
EPI_ISL_560973	Klinisk mikrobiologi NAL Trollhattan	The Public Health Agency of Sweden	Anna-Malin Linde et al
EPI_ISL_623087	Klinisk mikrobiologi NAL Trollhattan	The Public Health Agency of Sweden	Anna-Malin Linde et al
EPI_ISL_582797	Klinisk mikrobiologi Vasternorrland	The Public Health Agency of Sweden	Anna-Malin Linde et al

EPI_ISL_582798	Klinisk mikrobiologi Vasternorrland	The Public Health Agency of Sweden	Anna-Malin Linde et al
EPI_ISL_582800	Klinisk mikrobiologi Vasternorrland	The Public Health Agency of Sweden	Anna-Malin Linde et al
EPI_ISL_534248	Laboratoriemedicin Vasternorrland	The Public Health Agency of Sweden	Anna-Malin Linde et al
EPI_ISL_582807	Klinisk mikrobiologi SAS Boras	The Public Health Agency of Sweden	Anna-Malin Linde et al
EPI_ISL_648163	Gavle klinisk mikrobiologi	The Public Health Agency of Sweden	Anna-Malin Linde et al
EPI_ISL_648167	Eskilstuna	The Public Health Agency of Sweden	Anna-Malin Linde et al
EPI_ISL_648166	Eskilstuna	The Public Health Agency of Sweden	Anna-Malin Linde et al
EPI_ISL_913478	Klinisk mikrobiologi	The Public Health Agency of Sweden	Anna-Malin Linde et al
EPI_ISL_1008415	Klinisk mikrobiologi	The Public Health Agency of Sweden	Anna-Malin Linde et al
EPI_ISL_913475	Klinisk mikrobiologi	The Public Health Agency of Sweden	Anna-Malin Linde et al
EPI_ISL_913363	Klinisk mikrobiologi	The Public Health Agency of Sweden	Anna-Malin Linde et al
EPI_ISL_1008444	Klinisk mikrobiologi	The Public Health Agency of Sweden	Anna-Malin Linde et al
EPI_ISL_1008548	Klinisk mikrobiologi	The Public Health Agency of Sweden	Anna-Malin Linde et al
EPI_ISL_430846	General Intensive Care Unit, Raymond Poincaré Hospital (AP-HP), Lab Inflammation & Infection, U1173 University Paris Saclay-UVSQ/INSERM, Garches, France.	Institut Pasteur, Laboratory for Urgent Response to biological Threats	Annane Djillali et al
EPI_ISL_1593977	CHC Andrée Rosemon	Institut Pasteur de la Guyane	Anne Lavergne et al
EPI_ISL_1593978	CHC Andrée Rosemon	Institut Pasteur de la Guyane	Anne Lavergne et al
EPI_ISL_1627097	CHC Andrée Rosemon	Institut Pasteur de la Guyane	Anne Lavergne et al
EPI_ISL_1628333	Laboratoire Carage	Institut Pasteur de la Guyane	Anne Lavergne et al
EPI_ISL_1628334	CHC Andrée Rosemon	Institut Pasteur de la Guyane	Anne Lavergne et al
EPI_ISL_1628336	CNR Institut Pasteur de la Guyane	Institut Pasteur de la Guyane	Anne Lavergne et al
EPI_ISL_1628338	CNR Institut Pasteur de la Guyane	Institut Pasteur de la Guyane	Anne Lavergne et al
EPI_ISL_812267	Landstuhl Regional Medical Center	United States Air Force School of Aerospace Medicine	Anthony Fries et al
EPI_ISL_812271	Landstuhl Regional Medical Center	United States Air Force School of Aerospace Medicine	Anthony Fries et al
EPI_ISL_812275	Landstuhl Regional Medical Center	United States Air Force School of Aerospace Medicine	Anthony Fries et al
EPI_ISL_812280	Landstuhl Regional Medical Center	United States Air Force School of Aerospace Medicine	Anthony Fries et al
EPI_ISL_738323	Landstuhl Regional Medical Center	United States Air Force School of Aerospace Medicine	Anthony Fries et al
EPI_ISL_738321	Landstuhl Regional Medical Center	United States Air Force School of Aerospace Medicine	Anthony Fries et al
EPI_ISL_1038791	US Air Force School of Aerospace Medicine	US Air Force School of Aerospace Medicine	Anthony Fries et al
EPI_ISL_568946	MEPHI, Aix Marseille University	MEPHI, Aix Marseille University	Anthony LEVASSEUR et al

EPI_ISL_568968	MEPHI, Aix Marseille University	MEPHI, Aix Marseille University	Anthony LEVASSEUR et al
EPI_ISL_568978	MEPHI, Aix Marseille University	MEPHI, Aix Marseille University	Anthony LEVASSEUR et al
EPI_ISL_568983	MEPHI, Aix Marseille University	MEPHI, Aix Marseille University	Anthony LEVASSEUR et al
EPI_ISL_569007	MEPHI, Aix Marseille University	MEPHI, Aix Marseille University	Anthony LEVASSEUR et al
EPI_ISL_569055	MEPHI, Aix Marseille University	MEPHI, Aix Marseille University	Anthony LEVASSEUR et al
EPI_ISL_569095	MEPHI, Aix Marseille University	MEPHI, Aix Marseille University	Anthony LEVASSEUR et al
EPI_ISL_569171	MEPHI, Aix Marseille University	MEPHI, Aix Marseille University	Anthony LEVASSEUR et al
EPI_ISL_569218	MEPHI, Aix Marseille University	MEPHI, Aix Marseille University	Anthony LEVASSEUR et al
EPI_ISL_569241	MEPHI, Aix Marseille University	MEPHI, Aix Marseille University	Anthony LEVASSEUR et al
EPI_ISL_982230	MEPHI, Aix Marseille University	MEPHI, Aix Marseille University	Anthony LEVASSEUR et al
EPI_ISL_900144	MEPHI, Aix Marseille University	MEPHI, Aix Marseille University	Anthony LEVASSEUR et al
EPI_ISL_900163	MEPHI, Aix Marseille University	MEPHI, Aix Marseille University	Anthony LEVASSEUR et al
EPI_ISL_900165	MEPHI, Aix Marseille University	MEPHI, Aix Marseille University	Anthony LEVASSEUR et al
EPI_ISL_900080	MEPHI, Aix Marseille University	MEPHI, Aix Marseille University	Anthony LEVASSEUR et al
EPI_ISL_900192	MEPHI, Aix Marseille University	MEPHI, Aix Marseille University	Anthony LEVASSEUR et al
EPI_ISL_896130	MEPHI, Aix Marseille University	MEPHI, Aix Marseille University	Anthony LEVASSEUR et al
EPI_ISL_900082	MEPHI, Aix Marseille University	MEPHI, Aix Marseille University	Anthony LEVASSEUR et al
EPI_ISL_900168	MEPHI, Aix Marseille University	MEPHI, Aix Marseille University	Anthony LEVASSEUR et al
EPI_ISL_900176	MEPHI, Aix Marseille University	MEPHI, Aix Marseille University	Anthony LEVASSEUR et al
EPI_ISL_900196	MEPHI, Aix Marseille University	MEPHI, Aix Marseille University	Anthony LEVASSEUR et al
EPI_ISL_900169	MEPHI, Aix Marseille University	MEPHI, Aix Marseille University	Anthony LEVASSEUR et al
EPI_ISL_896134	MEPHI, Aix Marseille University	MEPHI, Aix Marseille University	Anthony LEVASSEUR et al
EPI_ISL_900181	MEPHI, Aix Marseille University	MEPHI, Aix Marseille University	Anthony LEVASSEUR et al
EPI_ISL_900125	MEPHI, Aix Marseille University	MEPHI, Aix Marseille University	Anthony LEVASSEUR et al
EPI_ISL_804478	MEPHI, Aix Marseille University	MEPHI, Aix Marseille University	Anthony LEVASSEUR et al
EPI_ISL_644375	MEPHI, Aix Marseille University	MEPHI, Aix Marseille University	Anthony LEVASSEUR et al
EPI_ISL_644475	MEPHI, Aix Marseille University	MEPHI, Aix Marseille University	Anthony LEVASSEUR et al
EPI_ISL_940274	Hôpital Bichat Claude Bernard, Laboratoire de Virologie	IAME UMR1137 Inserm, Université de Paris, Hôpital Bichat	Antoine Bridier-Nahmias et al
EPI_ISL_940552	Hôpital Bichat Claude Bernard, Laboratoire de Virologie	IAME UMR1137 Inserm, Université de Paris, Hôpital Bichat	Antoine Bridier-Nahmias et al
EPI_ISL_940530	Hôpital Bichat Claude Bernard, Laboratoire de Virologie	IAME UMR1137 Inserm, Université de Paris, Hôpital Bichat	Antoine Bridier-Nahmias et al

EPI_ISL_940372	Hôpital Bichat Claude Bernard, Laboratoire de Virologie	IAME UMR1137 Inserm, Université de Paris, Hôpital Bichat	Antoine Bridier-Nahmias et al
EPI_ISL_940325	Hôpital Bichat Claude Bernard, Laboratoire de Virologie	IAME UMR1137 Inserm, Université de Paris, Hôpital Bichat	Antoine Bridier-Nahmias et al
EPI_ISL_940186	Hôpital Bichat Claude Bernard, Laboratoire de Virologie	IAME UMR1137 Inserm, Université de Paris, Hôpital Bichat	Antoine Bridier-Nahmias et al
EPI_ISL_940175	Hôpital Bichat Claude Bernard, Laboratoire de Virologie	IAME UMR1137 Inserm, Université de Paris, Hôpital Bichat	Antoine Bridier-Nahmias et al
EPI_ISL_940177	Hôpital Bichat Claude Bernard, Laboratoire de Virologie	IAME UMR1137 Inserm, Université de Paris, Hôpital Bichat	Antoine Bridier-Nahmias et al
EPI_ISL_940165	Hôpital Bichat Claude Bernard, Laboratoire de Virologie	IAME UMR1137 Inserm, Université de Paris, Hôpital Bichat	Antoine Bridier-Nahmias et al
EPI_ISL_940340	Hôpital Bichat Claude Bernard, Laboratoire de Virologie	IAME UMR1137 Inserm, Université de Paris, Hôpital Bichat	Antoine Bridier-Nahmias et al
EPI_ISL_940427	Hôpital Bichat Claude Bernard, Laboratoire de Virologie	IAME UMR1137 Inserm, Université de Paris, Hôpital Bichat	Antoine Bridier-Nahmias et al
EPI_ISL_683835	CICM	Malaria Research and Training Center (MRTC-Parasito)	Antoine Dara et al
EPI_ISL_1135058	ORIAPOLE - BACTERIOLOGIE	CNR Virus des Infections Respiratoires - France SUD	Antonin Bal et al
EPI_ISL_660360	CHU Clermont-Ferrand	CNR Virus des Infections Respiratoires - France SUD	Antonin Bal et al
EPI_ISL_1371872	CERBALIANCE PROVENCE	CNR Virus des Infections Respiratoires - France SUD	Antonin Bal et al
EPI_ISL_645213	Unité des Virus Émergents	CNR Virus des Infections Respiratoires - France SUD	Antonin Bal et al
EPI_ISL_913666	Cerballiance	CNR Virus des Infections Respiratoires - France SUD	Antonin Bal et al
EPI_ISL_693387	CHU de Nice - Hôpital Archet 2	CNR Virus des Infections Respiratoires - France SUD	Antonin Bal et al
EPI_ISL_1336649	HOPITAL PRINCESSE GRACE	CNR Virus des Infections Respiratoires - France SUD	Antonin Bal et al
EPI_ISL_1135056	C.H. PRINCESSE GRACE	CNR Virus des Infections Respiratoires - France SUD	Antonin Bal et al
EPI_ISL_1371898	C H DE LA POLYNESIE FRANCAISE	CNR Virus des Infections Respiratoires - France SUD	Antonin Bal et al
EPI_ISL_1371903	C H DE LA POLYNESIE FRANCAISE	CNR Virus des Infections Respiratoires - France SUD	Antonin Bal et al
EPI_ISL_1371896	C H DE LA POLYNESIE FRANCAISE	CNR Virus des Infections Respiratoires - France SUD	Antonin Bal et al
EPI_ISL_1371902	C H DE LA POLYNESIE FRANCAISE	CNR Virus des Infections Respiratoires - France SUD	Antonin Bal et al
EPI_ISL_1371900	C H DE LA POLYNESIE FRANCAISE	CNR Virus des Infections Respiratoires - France SUD	Antonin Bal et al
EPI_ISL_1371904	C H DE LA POLYNESIE FRANCAISE	CNR Virus des Infections Respiratoires - France SUD	Antonin Bal et al
EPI_ISL_1371901	C H DE LA POLYNESIE FRANCAISE	CNR Virus des Infections Respiratoires - France SUD	Antonin Bal et al
EPI_ISL_1371905	C H DE LA POLYNESIE FRANCAISE	CNR Virus des Infections Respiratoires - France SUD	Antonin Bal et al
EPI_ISL_837279	Istituto Zooprofilattico Sperimentale del Mezzogiorno	TIGEM	Antonio Grimaldi et al
EPI_ISL_1086087	IZSM	TIGEM	Antonio Grimaldi et al
EPI_ISL_1086093	IZSM	TIGEM	Antonio Grimaldi et al
EPI_ISL_1086096	IZSM	TIGEM	Antonio Grimaldi et al
EPI_ISL_776981	Istituto Zooprofilattico Sperimentale del Mezzogiorno	TIGEM	Antonio Grimaldi et al

EPI_ISL_776973	Istituto Zooprofilattico Sperimentale del Mezzogiorno	TIGEM	Antonio Grimaldi et al
EPI_ISL_1086086	IZSM	TIGEM	Antonio Grimaldi et al
EPI_ISL_837300	Istituto Zooprofilattico Sperimentale del Mezzogiorno	TIGEM	Antonio Grimaldi et al
EPI_ISL_1656405	Istituto Zooprofilattico Sperimentale del Mezzogiorno	TIGEM	Antonio Grimaldi Patrizia Annunziata France
EPI_ISL_1196991	IZSM	TIGEM	Antonio Grimaldi Patrizia Annunziata France
EPI_ISL_1197001	IZSM	TIGEM	Antonio Grimaldi Patrizia Annunziata France
EPI_ISL_1197002	IZSM	TIGEM	Antonio Grimaldi Patrizia Annunziata France
EPI_ISL_1197005	IZSM	TIGEM	Antonio Grimaldi Patrizia Annunziata France
EPI_ISL_751339	IRCCS Sacro Cuore Don Calabria Hospital, Department of Infectious, Tropical Diseases & Microbiology	University of Verona, Department of Biotechnology	Antonio Mori et al
EPI_ISL_751342	IRCCS Sacro Cuore Don Calabria Hospital, Department of Infectious, Tropical Diseases & Microbiology	University of Verona, Department of Biotechnology	Antonio Mori et al
EPI_ISL_541031	Servicio de Microbiología, Hospital Miguel Servet, Zaragoza	SeqCOVID-SPAIN consortium/Institute of Biomedicine of Valencia, IBV-CSIC	Antonio Rezusta López et al
EPI_ISL_541034	Servicio de Microbiología, Hospital Miguel Servet, Zaragoza	SeqCOVID-SPAIN consortium/Institute of Biomedicine of Valencia, IBV-CSIC	Antonio Rezusta López et al
EPI_ISL_700595	Heideveld Emergency Centre	NHLS/UCT	Arash Iranzadeh et al
EPI_ISL_960172	Dr Abdurahman CDC wc DAC	National Health Laboratory Service/UCT	Arash Iranzadeh et al
EPI_ISL_640054	Groote Schuur Hospital wc GSH	NHLS/UCT	Arash Iranzadeh et al
EPI_ISL_640041	Green Point CDC wc GPH	NHLS/UCT	Arash Iranzadeh et al
EPI_ISL_640059	Du Noon CDC wc DNC	NHLS/UCT	Arash Iranzadeh et al
EPI_ISL_960142	Groote Schuur Hospital wc GSH	National Health Laboratory Service/UCT	Arash Iranzadeh et al
EPI_ISL_1040795	Groote Schuur Hospital wc GSH	NHLS/UCT	Arash Iranzadeh et al
EPI_ISL_1040796	Groote Schuur Hospital wc GSH	NHLS/UCT	Arash Iranzadeh et al
EPI_ISL_1040787	Groote Schuur Hospital wc GSH	NHLS/UCT	Arash Iranzadeh et al
EPI_ISL_1040793	Guguletu CHC wc GDH	NHLS/UCT	Arash Iranzadeh et al
EPI_ISL_1040783	Groote Schuur Hospital wc GSH	NHLS/UCT	Arash Iranzadeh et al
EPI_ISL_1040770	2 Military Hospital wc MAA	NHLS/UCT	Arash Iranzadeh et al
EPI_ISL_1040771	2 Military Hospital wc MAA	NHLS/UCT	Arash Iranzadeh et al
EPI_ISL_640129	Groote Schuur Hospital wc GSH	NHLS/UCT	Arash Iranzadeh et al
EPI_ISL_640130	Groote Schuur Hospital wc GSH	NHLS/UCT	Arash Iranzadeh et al
EPI_ISL_1040816	Groote Schuur Hospital wc GSH	NHLS/UCT	Arash Iranzadeh et al
EPI_ISL_700411	Hanover Park CHC wc HPH	NHLS/UCT	Arash Iranzadeh et al

esco Panariello Biancamaria Pierri Claudia T

esco Panariello Biancamaria Pierri Valentina

esco Panariello Biancamaria Pierri Valentina

esco Panariello Biancamaria Pierri Valentina

esco Panariello Biancamaria Pierri Valentina

EPI_ISL_700455	Guguletu CHC wc GDH	NHLS/UCT	Arash Iranzadeh et al
EPI_ISL_700481	Guguletu CHC wc GDH	NHLS/UCT	Arash Iranzadeh et al
EPI_ISL_1040815	Groote Schuur Hospital wc GSH	NHLS/UCT	Arash Iranzadeh et al
EPI_ISL_640138	Groote Schuur Hospital wc GSH	NHLS/UCT	Arash Iranzadeh et al
EPI_ISL_640091	Du Noon CDC wc DNC	NHLS/UCT	Arash Iranzadeh et al
EPI_ISL_960162	Heideveld Emergency Centre	National Health Laboratory Service/UCT	Arash Iranzadeh et al
EPI_ISL_1040667	Guguletu CHC wc GDH	NHLS/UCT	Arash Iranzadeh et al
EPI_ISL_1040761	Guguletu CHC	NHLS/UCT	Arash Iranzadeh et al
EPI_ISL_1040762	Bredasdorp clinic	NHLS/UCT	Arash Iranzadeh et al
EPI_ISL_1040760	Kensington CDC	NHLS/UCT	Arash Iranzadeh et al
EPI_ISL_1040647	Swellendam PHC	NHLS/UCT	Arash Iranzadeh et al
EPI_ISL_1040649	Groote Schuur Hospital wc GSH	NHLS/UCT	Arash Iranzadeh et al
EPI_ISL_1040767	Groote Schuur Hospital wc GSH	NHLS/UCT	Arash Iranzadeh et al
EPI_ISL_1040765	Groote Schuur Hospital wc GSH	NHLS/UCT	Arash Iranzadeh et al
EPI_ISL_1534309	Groote Schuur Hospital wc GSH	NHLS/UCT	Arash Iranzadeh et al
EPI_ISL_508337	Indian Institute of Science	National Institute of Biomedical Genomics	Arindam Maitra et al
EPI_ISL_508214	Government Medical College	National Institute of Biomedical Genomics	Arindam Maitra et al
EPI_ISL_508266	Government Medical College	National Institute of Biomedical Genomics	Arindam Maitra et al
EPI_ISL_1589741	INSACOG-WB	National Institute of Biomedical Genomics – INSACOG	Arindam Maitra et al
EPI_ISL_1589853	INSACOG-WB	National Institute of Biomedical Genomics – INSACOG	Arindam Maitra et al
EPI_ISL_1419217	INSACOG-WB	National Institute of Biomedical Genomics – INSACOG	Arindam Maitra et al
EPI_ISL_1589869	INSACOG-WB	National Institute of Biomedical Genomics – INSACOG	Arindam Maitra et al
EPI_ISL_1419562	INSACOG-WB	National Institute of Biomedical Genomics – INSACOG	Arindam Maitra et al
EPI_ISL_1419609	INSACOG-WB	National Institute of Biomedical Genomics – INSACOG	Arindam Maitra et al
EPI_ISL_1419620	INSACOG-WB	National Institute of Biomedical Genomics – INSACOG	Arindam Maitra et al
EPI_ISL_1419643	INSACOG-WB	National Institute of Biomedical Genomics – INSACOG	Arindam Maitra et al
EPI_ISL_1419631	INSACOG-WB	National Institute of Biomedical Genomics – INSACOG	Arindam Maitra et al
EPI_ISL_1419888	INSACOG-WB	National Institute of Biomedical Genomics – INSACOG	Arindam Maitra et al
EPI_ISL_1589966	INSACOG-WB	National Institute of Biomedical Genomics – INSACOG	Arindam Maitra et al
EPI_ISL_1419660	INSACOG-WB	National Institute of Biomedical Genomics – INSACOG	Arindam Maitra et al

EPI_ISL_1419659	INSACOG-WB	National Institute of Biomedical Genomics – INSACOG	Arindam Maitra et al
EPI_ISL_430852	Klinisk mikrobiologi och vardhygien Halmstad	The Public Health Agency of Sweden	Arne Kotz et al
EPI_ISL_1139157	National Center of Disease Control and Prevention of the Republic of Armenia	Institute of Molecular Biology NAS RA, Republic of Armenia, Department of Bioengineering, BioinformaticsInstitute and Molecular Biology IBMPh RAU, Republic of Armenia	Arsen Arakelyan et al
EPI_ISL_1139149	National Center of Disease Control and Prevention of the Republic of Armenia	Institute of Molecular Biology NAS RA, Republic of Armenia, Department of Bioengineering, BioinformaticsInstitute and Molecular Biology IBMPh RAU, Republic of Armenia	Arsen Arakelyan et al
EPI_ISL_1139150	National Center of Disease Control and Prevention of the Republic of Armenia	Institute of Molecular Biology NAS RA, Republic of Armenia, Department of Bioengineering, BioinformaticsInstitute and Molecular Biology IBMPh RAU, Republic of Armenia	Arsen Arakelyan et al
EPI_ISL_1139151	National Center of Disease Control and Prevention of the Republic of Armenia	Institute of Molecular Biology NAS RA, Republic of Armenia, Department of Bioengineering, BioinformaticsInstitute and Molecular Biology IBMPh RAU, Republic of Armenia	Arsen Arakelyan et al
EPI_ISL_1139153	National Center of Disease Control and Prevention of the Republic of Armenia	Institute of Molecular Biology NAS RA, Republic of Armenia, Department of Bioengineering, BioinformaticsInstitute and Molecular Biology IBMPh RAU, Republic of Armenia	Arsen Arakelyan et al
EPI_ISL_1591100	CMA Dano	Centre Muraz	Arsène Zongo et al
EPI_ISL_569776	Omsk Research Institute of Natural Focal Infections	WHO National Influenza Centre Russian Federation	Artem Fadeev et al
EPI_ISL_639919	Omsk Research Institute of Natural Focal Infections	WHO National Influenza Centre Russian Federation	Artem Fadeev et al
EPI_ISL_639920	Omsk Research Institute of Natural Focal Infections	WHO National Influenza Centre Russian Federation	Artem Fadeev et al
EPI_ISL_569821	Omsk Research Institute of Natural Focal Infections	WHO National Influenza Centre Russian Federation	Artem Fadeev et al
EPI_ISL_639932	Omsk Research Institute of Natural Focal Infections	WHO National Influenza Centre Russian Federation	Artem Fadeev et al
EPI_ISL_569803	Omsk Research Institute of Natural Focal Infections	WHO National Influenza Centre Russian Federation	Artem Fadeev et al
EPI_ISL_639933	Omsk Research Institute of Natural Focal Infections	WHO National Influenza Centre Russian Federation	Artem Fadeev et al
EPI_ISL_639935	Omsk Research Institute of Natural Focal Infections	WHO National Influenza Centre Russian Federation	Artem Fadeev et al
EPI_ISL_639943	Omsk Research Institute of Natural Focal Infections	WHO National Influenza Centre Russian Federation	Artem Fadeev et al
EPI_ISL_1299227	OLVZ Aalst	OLVZ Aalst	Astrid Holderbeke et al
EPI_ISL_1302291	Instituto Nacional de Enfermedades Respiratorias (INER)	Instituto de Biotecnología de la UNAM	Authors from IBT et al
EPI_ISL_1302152	Instituto Nacional de Enfermedades Respiratorias (INER)	Instituto de Biotecnología de la UNAM	Authors from IBT et al
EPI_ISL_1301499	Instituto Nacional de Enfermedades Respiratorias (INER)	Instituto de Biotecnología de la UNAM	Authors from IBT et al
EPI_ISL_1301507	Instituto Nacional de Enfermedades Respiratorias (INER)	Instituto de Biotecnología de la UNAM	Authors from IBT et al
EPI_ISL_1301474	Instituto de Diagnostico y Referencia Epidemiologicos InDRE_RNLSP	Instituto de Biotecnología de la UNAM	Authors from IBT et al
EPI_ISL_1302210	Instituto de Diagnostico y Referencia Epidemiologicos InDRE_RNLSP	Instituto de Biotecnología de la UNAM	Authors from IBT et al
EPI_ISL_1301519	Instituto de Diagnostico y Referencia Epidemiologicos InDRE_RNLSP	Instituto de Biotecnología de la UNAM	Authors from IBT et al

EPI_ISL_1340864	West Bandung Public Health	West Java Health Laboratory; School of Life Sciences and Technology, Institut Teknologi Bandung	Azzania Fibriani et al
EPI_ISL_1634465	Bandung Public Health	West Java Health Laboratory; School of Life Sciences and Technology, Institut Teknologi Bandung	Azzania Fibriani et al
EPI_ISL_1615589	Sumedang Public Health	West Java Health Laboratory; School of Life Sciences and Technology, Institut Teknologi Bandung	Azzania Fibriani et al
EPI_ISL_1615590	Subang Public Health	West Java Health Laboratory; School of Life Sciences and Technology, Institut Teknologi Bandung	Azzania Fibriani et al
EPI_ISL_1634442	Mitra Kasih Hospital	West Java Health Laboratory; School of Life Sciences and Technology, Institut Teknologi Bandung	Azzania Fibriani et al
EPI_ISL_1660419	Santosa Kopo Hospital	West Java Health Laboratory; School of Life Sciences and Technology, Institut Teknologi Bandung	Azzania Fibriani et al
EPI_ISL_747240	Al Islam Hospital	West Java Health Laboratory; School of Life Sciences and Technology, Institut Teknologi Bandung	Azzania Fibriani et al
EPI_ISL_1056959	Institute of Biocides and Medical Ecology, Belgarde, Serbia	Virology Department Institute of Microbiology and Immunology Faculty of Medicine University of Belgrade	Banko Ana et al
EPI_ISL_891272	Institute of Biocides and Medical Ecology, Belgarde, Serbia	Virology department Institute of microbiology and immunology Faculty of Medicine University of Belgrade	Banko Ana et al
EPI_ISL_1056968	Institute of Biocides and Medical Ecology, Belgarde, Serbia	Virology Department Institute of Microbiology and Immunology Faculty of Medicine University of Belgrade	Banko Ana et al
EPI_ISL_1057105	Institute of Biocides and Medical Ecology, Belgarde, Serbia	Virology Department Institute of Microbiology and Immunology Faculty of Medicine University of Belgrade	Banko Ana et al
EPI_ISL_1061431	Institute of Biocides and Medical Ecology, Belgarde, Serbia	Virology Department Institute of Microbiology and Immunology Faculty of Medicine University of Belgrade	Banko Ana et al
EPI_ISL_909737	Institute of Biocides and Medical Ecology, Belgrade, Serbia	Virology Department, Institute of microbiology and immunology, Faculty of Medicine University of Blegrade	Banko Ana et al
EPI_ISL_891271	Institute of Biocides and Medical Ecology, Belgarde, Serbia	Virology department Institute of microbiology and immunology Faculty of Medicine University of Belgrade	Banko Ana et al
EPI_ISL_1057040	Institute of Biocides and Medical Ecology, Belgarde, Serbia	Virology Department Institute of Microbiology and Immunology Faculty of Medicine University of Belgrade	Banko Ana et al
EPI_ISL_930854	Central Laboratory of Public Health of Rio Grande do Sul (Lacen-RS)	State Center for Health Surveillance of the Health Department of the State of Rio Grande do Sul (CEVS/SES-RS)	Barcellos R et al
EPI_ISL_930855	Central Laboratory of Public Health of Rio Grande do Sul (Lacen-RS)	State Center for Health Surveillance of the Health Department of the State of Rio Grande do Sul (CEVS/SES-RS)	Barcellos R et al
EPI_ISL_942407	Central Laboratory of Public Health of Rio Grande do Sul (Lacen-RS)	State Center for Health Surveillance of the Health Department of the State of Rio Grande do Sul (CEVS/SES-RS)	Barcellos R et al
EPI_ISL_930856	Central Laboratory of Public Health of Rio Grande do Sul (Lacen-RS)	State Center for Health Surveillance of the Health Department of the State of Rio Grande do Sul (CEVS/SES-RS)	Barcellos R et al
EPI_ISL_930857	Central Laboratory of Public Health of Rio Grande do Sul (Lacen-RS)	State Center for Health Surveillance of the Health Department of the State of Rio Grande do Sul (CEVS/SES-RS)	Barcellos R et al
EPI_ISL_930858	Central Laboratory of Public Health of Rio Grande do Sul (Lacen-RS)	State Center for Health Surveillance of the Health Department of the State of Rio Grande do Sul (CEVS/SES-RS)	Barcellos R et al
EPI_ISL_942374	Central Laboratory of Public Health of Rio Grande do Sul (Lacen-RS)	State Center for Health Surveillance of the Health Department of the State of Rio Grande do Sul (CEVS/SES-RS)	Barcellos R et al

EPI_ISL_942375	Central Laboratory of Public Health of Rio Grande do Sul (Lacen-RS)	State Center for Health Surveillance of the Health Department of the State of Rio Grande do Sul (CEVS/SES-RS)	Barcellos R et al
EPI_ISL_942896	Central Laboratory of Public Health of Rio Grande do Sul (Lacen-RS)	State Center for Health Surveillance of the Health Department of the State of Rio Grande do Sul (CEVS/SES-RS)	Barcellos R et al
EPI_ISL_942897	Central Laboratory of Public Health of Rio Grande do Sul (Lacen-RS)	State Center for Health Surveillance of the Health Department of the State of Rio Grande do Sul (CEVS/SES-RS)	Barcellos R et al
EPI_ISL_942898	Central Laboratory of Public Health of Rio Grande do Sul (Lacen-RS)	State Center for Health Surveillance of the Health Department of the State of Rio Grande do Sul (CEVS/SES-RS)	Barcellos R et al
EPI_ISL_942899	Central Laboratory of Public Health of Rio Grande do Sul (Lacen-RS)	State Center for Health Surveillance of the Health Department of the State of Rio Grande do Sul (CEVS/SES-RS)	Barcellos R et al
EPI_ISL_942930	Central Laboratory of Public Health of Rio Grande do Sul (Lacen-RS)	State Center for Health Surveillance of the Health Department of the State of Rio Grande do Sul (CEVS/SES-RS)	Barcellos R et al
EPI_ISL_942931	Central Laboratory of Public Health of Rio Grande do Sul (Lacen-RS)	State Center for Health Surveillance of the Health Department of the State of Rio Grande do Sul (CEVS/SES-RS)	Barcellos R et al
EPI_ISL_982236	National Institute of Laboratory Medicine and Referral Center	Genomic Research Lab, BCSIR	Barna Goswami et al
EPI_ISL_1040481	National Institute of Laboratory Medicine and Referral Center	Genomic Research Lab, BCSIR	Barna Goswami et al
EPI_ISL_483551	Kingdom of Bahrein Ministry of Health	Erasmus Medical Center	Bas Oude Munnink et al
EPI_ISL_483552	Kingdom of Bahrein Ministry of Health	Erasmus Medical Center	Bas Oude Munnink et al
EPI_ISL_483555	Kingdom of Bahrein Ministry of Health	Erasmus Medical Center	Bas Oude Munnink et al
EPI_ISL_483558	Kingdom of Bahrein Ministry of Health	Erasmus Medical Center	Bas Oude Munnink et al
EPI_ISL_483560	Kingdom of Bahrein Ministry of Health	Erasmus Medical Center	Bas Oude Munnink et al
EPI_ISL_483561	Kingdom of Bahrein Ministry of Health	Erasmus Medical Center	Bas Oude Munnink et al
EPI_ISL_483565	Kingdom of Bahrein Ministry of Health	Erasmus Medical Center	Bas Oude Munnink et al
EPI_ISL_523137	Dutch COVID-19 response team	Erasmus Medical Center	Bas Oude Munnink et al
EPI_ISL_523165	Dutch COVID-19 response team	Erasmus Medical Center	Bas Oude Munnink et al
EPI_ISL_801406	Dutch COVID-19 response team	Erasmus Medical Center	Bas Oude Munnink et al
EPI_ISL_801429	Dutch COVID-19 response team	Erasmus Medical Center	Bas Oude Munnink et al
EPI_ISL_422757	Dutch COVID-19 response team	Erasmus Medical Center	Bas Oude Munnink et al
EPI_ISL_422588	Dutch COVID-19 response team	Erasmus Medical Center	Bas Oude Munnink et al
EPI_ISL_455136	Dutch COVID-19 response team	Erasmus Medical Center	Bas Oude Munnink et al
EPI_ISL_455138	Dutch COVID-19 response team	Erasmus Medical Center	Bas Oude Munnink et al
EPI_ISL_455158	Dutch COVID-19 response team	Erasmus Medical Center	Bas Oude Munnink et al
EPI_ISL_455169	Dutch COVID-19 response team	Erasmus Medical Center	Bas Oude Munnink et al
EPI_ISL_460715	Dutch COVID-19 response team	Erasmus Medical Center	Bas Oude Munnink et al
EPI_ISL_461061	Dutch COVID-19 response team	Erasmus Medical Center	Bas Oude Munnink et al

EPI_ISL_461124	Dutch COVID-19 response team	Erasmus Medical Center	Bas Oude Munnink et al
EPI_ISL_422850	Dutch COVID-19 response team	Erasmus Medical Center	Bas Oude Munnink et al
EPI_ISL_523308	Dutch COVID-19 response team	Erasmus Medical Center	Bas Oude Munnink et al
EPI_ISL_523334	Dutch COVID-19 response team	Erasmus Medical Center	Bas Oude Munnink et al
EPI_ISL_523337	Dutch COVID-19 response team	Erasmus Medical Center	Bas Oude Munnink et al
EPI_ISL_523384	Dutch COVID-19 response team	Erasmus Medical Center	Bas Oude Munnink et al
EPI_ISL_632359	Dutch COVID-19 response team	Erasmus Medical Center	Bas Oude Munnink et al
EPI_ISL_722378	Dutch COVID-19 response team	Erasmus Medical Center	Bas Oude Munnink et al
EPI_ISL_763139	Dutch COVID-19 response team	Erasmus Medical Center	Bas Oude Munnink et al
EPI_ISL_1311245	Dutch COVID-19 response team	Erasmus Medical Center	Bas Oude Munnink et al
EPI_ISL_1311685	Dutch COVID-19 response team	Erasmus Medical Center	Bas Oude Munnink et al
EPI_ISL_1311275	Dutch COVID-19 response team	Erasmus Medical Center	Bas Oude Munnink et al
EPI_ISL_577849	Dutch COVID-19 response team	Erasmus Medical Center	Bas Oude Munnink et al
EPI_ISL_461158	Dutch COVID-19 response team	Erasmus Medical Center	Bas Oude Munnink et al
EPI_ISL_722292	Dutch COVID-19 response team	Erasmus Medical Center	Bas Oude Munnink et al
EPI_ISL_632327	Dutch COVID-19 response team	Erasmus Medical Center	Bas Oude Munnink et al
EPI_ISL_632417	Dutch COVID-19 response team	Erasmus Medical Center	Bas Oude Munnink et al
EPI_ISL_523547	Dutch COVID-19 response team	Erasmus Medical Center	Bas Oude Munnink et al
EPI_ISL_722396	Dutch COVID-19 response team	Erasmus Medical Center	Bas Oude Munnink et al
EPI_ISL_801441	Dutch COVID-19 response team	Erasmus Medical Center	Bas Oude Munnink et al
EPI_ISL_801442	Dutch COVID-19 response team	Erasmus Medical Center	Bas Oude Munnink et al
EPI_ISL_1310851	Dutch COVID-19 response team	Erasmus Medical Center	Bas Oude Munnink et al
EPI_ISL_1311252	Dutch COVID-19 response team	Erasmus Medical Center	Bas Oude Munnink et al
EPI_ISL_523645	Dutch COVID-19 response team	Erasmus Medical Center	Bas Oude Munnink et al
EPI_ISL_574789	Dutch COVID-19 response team	Erasmus Medical Center	Bas Oude Munnink et al
EPI_ISL_577932	Dutch COVID-19 response team	Erasmus Medical Center	Bas Oude Munnink et al
EPI_ISL_632400	Dutch COVID-19 response team	Erasmus Medical Center	Bas Oude Munnink et al
EPI_ISL_632769	Dutch COVID-19 response team	Erasmus Medical Center	Bas Oude Munnink et al
EPI_ISL_461315	Dutch COVID-19 response team	Erasmus Medical Center	Bas Oude Munnink et al
EPI_ISL_422933	Dutch COVID-19 response team	Erasmus Medical Center	Bas Oude Munnink et al

EPI_ISL_523510	Dutch COVID-19 response team	Erasmus Medical Center	Bas Oude Munnink et al
EPI_ISL_517616	Academic Hospital Paramaribo	Erasmus Medical Center	Bas Oude Munnink et al
EPI_ISL_517628	Academic Hospital Paramaribo	Erasmus Medical Center	Bas Oude Munnink et al
EPI_ISL_517629	Academic Hospital Paramaribo	Erasmus Medical Center	Bas Oude Munnink et al
EPI_ISL_517631	Academic Hospital Paramaribo	Erasmus Medical Center	Bas Oude Munnink et al
EPI_ISL_517632	Academic Hospital Paramaribo	Erasmus Medical Center	Bas Oude Munnink et al
EPI_ISL_517634	Academic Hospital Paramaribo	Erasmus Medical Center	Bas Oude Munnink et al
EPI_ISL_517635	Academic Hospital Paramaribo	Erasmus Medical Center	Bas Oude Munnink et al
EPI_ISL_517642	Academic Hospital Paramaribo	Erasmus Medical Center	Bas Oude Munnink et al
EPI_ISL_517643	Academic Hospital Paramaribo	Erasmus Medical Center	Bas Oude Munnink et al
EPI_ISL_517645	Academic Hospital Paramaribo	Erasmus Medical Center	Bas Oude Munnink et al
EPI_ISL_517657	Academic Hospital Paramaribo	Erasmus Medical Center	Bas Oude Munnink et al
EPI_ISL_518799	Academic Hospital Paramaribo	Erasmus Medical Center	Bas Oude Munnink et al
EPI_ISL_518800	Academic Hospital Paramaribo	Erasmus Medical Center	Bas Oude Munnink et al
EPI_ISL_518805	Academic Hospital Paramaribo	Erasmus Medical Center	Bas Oude Munnink et al
EPI_ISL_518807	Academic Hospital Paramaribo	Erasmus Medical Center	Bas Oude Munnink et al
EPI_ISL_518811	Academic Hospital Paramaribo	Erasmus Medical Center	Bas Oude Munnink et al
EPI_ISL_518812	Academic Hospital Paramaribo	Erasmus Medical Center	Bas Oude Munnink et al
EPI_ISL_517661	Academic Hospital Paramaribo	Erasmus Medical Center	Bas Oude Munnink et al
EPI_ISL_517662	Academic Hospital Paramaribo	Erasmus Medical Center	Bas Oude Munnink et al
EPI_ISL_518818	Academic Hospital Paramaribo	Erasmus Medical Center	Bas Oude Munnink et al
EPI_ISL_1259261	LHUB-ULB	UAntwerp, Laboratory of Medical Microbiology	Basil Britto Xavier et al
EPI_ISL_1259276	LHUB-ULB	UAntwerp, Laboratory of Medical Microbiology	Basil Britto Xavier et al
EPI_ISL_629023	Centro de Biotecnología Vegetal, Universidad Andrés Bello, Center for Genome Regulation	Center for Mathematical Modeling and Center for Genome Regulation. Santiago, Chile	Bastias M et al
EPI_ISL_629015	Centro de Biotecnología Vegetal, Universidad Andrés Bello, Center for Genome Regulation	Center for Mathematical Modeling and Center for Genome Regulation. Santiago, Chile	Bastias M et al
EPI_ISL_629016	Centro de Biotecnología Vegetal, Universidad Andrés Bello, Center for Genome Regulation	Center for Mathematical Modeling and Center for Genome Regulation. Santiago, Chile	Bastias M et al
EPI_ISL_849151	Servicio Virosis Respiratorias-Departamento Virología-INEI	Instituto Nacional Enfermedades Infecciosas C.G.Malbran	Baumeister E. et al
EPI_ISL_849154	Servicio Virosis Respiratorias-Departamento Virología-INEI	Instituto Nacional Enfermedades Infecciosas C.G.Malbran	Baumeister E. et al
EPI_ISL_849318	Servicio Virosis Respiratorias-Departamento Virología-INEI	Instituto Nacional Enfermedades Infecciosas C.G.Malbran	Baumeister E. et al

EPI_ISL_849301	Servicio Virosis Respiratorias-Departamento Virología-INEI	Instituto Nacional Enfermedades Infecciosas C.G.Malbran	Baumeister E. et al
EPI_ISL_849295	Servicio Virosis Respiratorias-Departamento Virología-INEI	Instituto Nacional Enfermedades Infecciosas C.G.Malbran	Baumeister E. et al
EPI_ISL_849298	Servicio Virosis Respiratorias-Departamento Virología-INEI	Instituto Nacional Enfermedades Infecciosas C.G.Malbran	Baumeister E. et al
EPI_ISL_849280	Servicio Virosis Respiratorias-Departamento Virología-INEI	Instituto Nacional Enfermedades Infecciosas C.G.Malbran	Baumeister E. et al
EPI_ISL_849285	Servicio Virosis Respiratorias-Departamento Virología-INEI	Instituto Nacional Enfermedades Infecciosas C.G.Malbran	Baumeister E. et al
EPI_ISL_849306	Servicio Virosis Respiratorias-Departamento Virología-INEI	Instituto Nacional Enfermedades Infecciosas C.G.Malbran	Baumeister E. et al
EPI_ISL_849281	Servicio Virosis Respiratorias-Departamento Virología-INEI	Instituto Nacional Enfermedades Infecciosas C.G.Malbran	Baumeister E. et al
EPI_ISL_849289	Servicio Virosis Respiratorias-Departamento Virología-INEI	Instituto Nacional Enfermedades Infecciosas C.G.Malbran	Baumeister E. et al
EPI_ISL_849284	Servicio Virosis Respiratorias-Departamento Virología-INEI	Instituto Nacional Enfermedades Infecciosas C.G.Malbran	Baumeister E. et al
EPI_ISL_849291	Servicio Virosis Respiratorias-Departamento Virología-INEI	Instituto Nacional Enfermedades Infecciosas C.G.Malbran	Baumeister E. et al
EPI_ISL_849373	Servicio Virosis Respiratorias-Departamento Virología-INEI	Instituto Nacional Enfermedades Infecciosas C.G.Malbran	Baumeister E. et al
EPI_ISL_849353	Servicio Virosis Respiratorias-Departamento Virología-INEI	Instituto Nacional Enfermedades Infecciosas C.G.Malbran	Baumeister E. et al
EPI_ISL_849361	Servicio Virosis Respiratorias-Departamento Virología-INEI	Instituto Nacional Enfermedades Infecciosas C.G.Malbran	Baumeister E. et al
EPI_ISL_849362	Servicio Virosis Respiratorias-Departamento Virología-INEI	Instituto Nacional Enfermedades Infecciosas C.G.Malbran	Baumeister E. et al
EPI_ISL_849369	Servicio Virosis Respiratorias-Departamento Virología-INEI	Instituto Nacional Enfermedades Infecciosas C.G.Malbran	Baumeister E. et al
EPI_ISL_856792	Servicio Virosis Respiratorias-Departamento Virología-INEI	Instituto Nacional Enfermedades Infecciosas C.G.Malbran	Baumeister E. et al
EPI_ISL_856766	Servicio Virosis Respiratorias-Departamento Virología-INEI	Instituto Nacional Enfermedades Infecciosas C.G.Malbran	Baumeister E. et al
EPI_ISL_856771	Servicio Virosis Respiratorias-Departamento Virología-INEI	Instituto Nacional Enfermedades Infecciosas C.G.Malbran	Baumeister E. et al
EPI_ISL_422563	Institute of Microbiology Universidad San Francisco de Quito	Institute of Microbiology Universidad San Francisco de Quito	Belen Prado-Vivar et al
EPI_ISL_477014	Institute of Microbiology, Universidad San Francisco de Quito	Institute of Microbiology, Universidad San Francisco de Quito	Belen Prado-Vivar et al
EPI_ISL_481244	Hospital IESS Babahoyo	Institute of Microbiology, Universidad San Francisco de Quito	Belén Prado-Vivar et al
EPI_ISL_481245	Hospital IESS Babahoyo	Institute of Microbiology, Universidad San Francisco de Quito	Belén Prado-Vivar et al
EPI_ISL_471268	Hospital IESS Babahoyo	Institute of Microbiology, Universidad San Francisco de Quito	Belén Prado-Vivar et al
EPI_ISL_481246	Hospital IESS Babahoyo	Institute of Microbiology, Universidad San Francisco de Quito	Belén Prado-Vivar et al
EPI_ISL_481248	Hospital IESS Babahoyo	Institute of Microbiology, Universidad San Francisco de Quito	Belén Prado-Vivar et al
EPI_ISL_491939	Institute of Microbiology, Universidad San Francisco de Quito	Institute of Microbiology, Universidad San Francisco de Quito	Belén Prado-Vivar et al
EPI_ISL_491940	Institute of Microbiology, Universidad San Francisco de Quito	Institute of Microbiology, Universidad San Francisco de Quito	Belén Prado-Vivar et al
EPI_ISL_491936	Institute of Microbiology, Universidad San Francisco de Quito	Institute of Microbiology, Universidad San Francisco de Quito	Belén Prado-Vivar et al
EPI_ISL_491938	Institute of Microbiology, Universidad San Francisco de Quito	Institute of Microbiology, Universidad San Francisco de Quito	Belén Prado-Vivar et al
EPI_ISL_697783	Institute of Microbiology, Universidad San Francisco de Quito	Institute of Microbiology, Universidad San Francisco de Quito	Belén Prado-Vivar et al

EPI_ISL_697784	Institute of Microbiology, Universidad San Francisco de Quito	Institute of Microbiology, Universidad San Francisco de Quito	Belén Prado-Vivar et al
EPI_ISL_697786	Institute of Microbiology, Universidad San Francisco de Quito	Institute of Microbiology, Universidad San Francisco de Quito	Belén Prado-Vivar et al
EPI_ISL_527812	Institute of Microbiology, Universidad San Francisco de Quito	Institute of Microbiology, Universidad San Francisco de Quito	Belén Prado-Vivar et al
EPI_ISL_527814	Institute of Microbiology, Universidad San Francisco de Quito	Institute of Microbiology, Universidad San Francisco de Quito	Belén Prado-Vivar et al
EPI_ISL_527817	Institute of Microbiology, Universidad San Francisco de Quito	Institute of Microbiology, Universidad San Francisco de Quito	Belén Prado-Vivar et al
EPI_ISL_539791	Institute of Microbiology, Universidad San Francisco de Quito	Institute of Microbiology, Universidad San Francisco de Quito	Belén Prado-Vivar et al
EPI_ISL_539792	Institute of Microbiology, Universidad San Francisco de Quito	Institute of Microbiology, Universidad San Francisco de Quito	Belén Prado-Vivar et al
EPI_ISL_697789	Institute of Microbiology, Universidad San Francisco de Quito	Institute of Microbiology, Universidad San Francisco de Quito	Belén Prado-Vivar et al
EPI_ISL_697790	Institute of Microbiology, Universidad San Francisco de Quito	Institute of Microbiology, Universidad San Francisco de Quito	Belén Prado-Vivar et al
EPI_ISL_697791	Institute of Microbiology, Universidad San Francisco de Quito	Institute of Microbiology, Universidad San Francisco de Quito	Belén Prado-Vivar et al
EPI_ISL_697792	Institute of Microbiology, Universidad San Francisco de Quito	Institute of Microbiology, Universidad San Francisco de Quito	Belén Prado-Vivar et al
EPI_ISL_697793	Institute of Microbiology, Universidad San Francisco de Quito	Institute of Microbiology, Universidad San Francisco de Quito	Belén Prado-Vivar et al
EPI_ISL_697798	Institute of Microbiology, Universidad San Francisco de Quito	Institute of Microbiology, Universidad San Francisco de Quito	Belén Prado-Vivar et al
EPI_ISL_697799	Institute of Microbiology, Universidad San Francisco de Quito	Institute of Microbiology, Universidad San Francisco de Quito	Belén Prado-Vivar et al
EPI_ISL_697800	Institute of Microbiology, Universidad San Francisco de Quito	Institute of Microbiology, Universidad San Francisco de Quito	Belén Prado-Vivar et al
EPI_ISL_824290	Institute of Microbiology, Universidad San Francisco de Quito	Institute of Microbiology, Universidad San Francisco de Quito	Belén Prado-Vivar et al
EPI_ISL_824286	Institute of Microbiology, Universidad San Francisco de Quito	Institute of Microbiology, Universidad San Francisco de Quito	Belén Prado-Vivar et al
EPI_ISL_824288	Institute of Microbiology, Universidad San Francisco de Quito	Institute of Microbiology, Universidad San Francisco de Quito	Belén Prado-Vivar et al
EPI_ISL_824289	Institute of Microbiology, Universidad San Francisco de Quito	Institute of Microbiology, Universidad San Francisco de Quito	Belén Prado-Vivar et al
EPI_ISL_824285	Institute of Microbiology, Universidad San Francisco de Quito	Institute of Microbiology, Universidad San Francisco de Quito	Belén Prado-Vivar et al
EPI_ISL_824293	Institute of Microbiology, Universidad San Francisco de Quito	Institute of Microbiology, Universidad San Francisco de Quito	Belén Prado-Vivar et al
EPI_ISL_824294	Institute of Microbiology, Universidad San Francisco de Quito	Institute of Microbiology, Universidad San Francisco de Quito	Belén Prado-Vivar et al
EPI_ISL_877561	Institute of Microbiology, Universidad San Francisco de Quito	Institute of Microbiology, Universidad San Francisco de Quito	Belén Prado-Vivar et al
EPI_ISL_877562	Institute of Microbiology, Universidad San Francisco de Quito	Institute of Microbiology, Universidad San Francisco de Quito	Belén Prado-Vivar et al
EPI_ISL_1656147	Institute of Microbiology, Universidad San Francisco de Quito	Institute of Microbiology, Universidad San Francisco de Quito	Belén Prado-Vivar et al
EPI_ISL_1540707	Universidad Tecnica Particular de Loja	Institute of Microbiology, Universidad San Francisco de Quito	Belén Prado-Vivar et al
EPI_ISL_1656146	Universidad Tecnica Particular de Loja	Institute of Microbiology, Universidad San Francisco de Quito	Belén Prado-Vivar et al
EPI_ISL_407896	Pathology Queensland	Public Health Virology Laboratory	Ben Huang et al
EPI_ISL_411951	Unit for Laboratory Development and Technology Transfer, Public Health Agency of Sweden	Unit for Laboratory Development and Technology Transfer, Public Health Agency of Sweden	Bengner et al
EPI_ISL_476778	Stanford clinical virology lab	Chan-Zuckerberg Biohub	Benjamin Pinksy et al

EPI_ISL_476782	Stanford clinical virology lab	Chan-Zuckerberg Biohub	Benjamin Pinksy et al
EPI_ISL_476790	Stanford clinical virology lab	Chan-Zuckerberg Biohub	Benjamin Pinksy et al
EPI_ISL_1540836	The Jackson Laboratory	The Jackson Laboratory	Bergeron D et al
EPI_ISL_1039831	National Public Health Center, COVID Laboratory	National Public Health Center, National Biosafety Laboratory	Bernadett Pályi et al
EPI_ISL_1172027	National Public Health Center, COVID Laboratory	National Public Health Center, National Biosafety Laboratory	Bernadett Pályi et al
EPI_ISL_1039986	National Public Health Center, COVID Laboratory	National Public Health Center, National Biosafety Laboratory	Bernadett Pályi et al
EPI_ISL_1039983	National Public Health Center, COVID Laboratory	National Public Health Center, National Biosafety Laboratory	Bernadett Pályi et al
EPI_ISL_1039981	National Public Health Center, COVID Laboratory	National Public Health Center, National Biosafety Laboratory	Bernadett Pályi et al
EPI_ISL_1039980	National Public Health Center, COVID Laboratory	National Public Health Center, National Biosafety Laboratory	Bernadett Pályi et al
EPI_ISL_1039961	National Public Health Center, COVID Laboratory	National Public Health Center, National Biosafety Laboratory	Bernadett Pályi et al
EPI_ISL_1039964	National Public Health Center, COVID Laboratory	National Public Health Center, National Biosafety Laboratory	Bernadett Pályi et al
EPI_ISL_1039837	National Public Health Center, COVID Laboratory	National Public Health Center, National Biosafety Laboratory	Bernadett Pályi et al
EPI_ISL_1039959	National Public Health Center, COVID Laboratory	National Public Health Center, National Biosafety Laboratory	Bernadett Pályi et al
EPI_ISL_1172022	National Public Health Center, COVID Laboratory	National Public Health Center, National Biosafety Laboratory	Bernadett Pályi et al
EPI_ISL_1095612	National Public Health Center, COVID Laboratory	National Public Health Center, National Biosafety Laboratory	Bernadett Pályi et al
EPI_ISL_1039993	National Public Health Center, COVID Laboratory	National Public Health Center, National Biosafety Laboratory	Bernadett Pályi et al
EPI_ISL_1195207	National Public Health Center, COVID Laboratory	National Public Health Center, National Biosafety Laboratory	Bernadett Pályi et al
EPI_ISL_1447296	National Public Health Center, COVID Laboratory	National Public Health Center, National Biosafety Laboratory	Bernadett Pályi et al
EPI_ISL_1195192	National Public Health Center, COVID Laboratory	National Public Health Center, National Biosafety Laboratory	Bernadett Pályi et al
EPI_ISL_1095621	National Public Health Center, COVID Laboratory	National Public Health Center, National Biosafety Laboratory	Bernadett Pályi et al
EPI_ISL_1095620	National Public Health Center, COVID Laboratory	National Public Health Center, National Biosafety Laboratory	Bernadett Pályi et al
EPI_ISL_415159	KU Leuven, Clinical and Epidemiological Virology	KU Leuven, Clinical and Epidemiological Virology	Bert Vanmechelen et al
EPI_ISL_407976	KU Leuven, Clinical and Epidemiological Virology	KU Leuven, Clinical and Epidemiological Virology	Bert Vanmechelen et al
EPI_ISL_763064	RS Kasih Ibu, Surakarta	Universitas Sebelas Maret (UNS); Rumah Sakit UNS (RS-UNS); National Institute of Health Research and Development, Indonesian Ministry of Health.	Betty Suryawati et al
EPI_ISL_1402340	CMBG FN Brno	CMBG FN Brno	Bezdicek M. et al
EPI_ISL_1402342	CMBG FN Brno	CMBG FN Brno	Bezdicek M. et al
EPI_ISL_1402345	CMBG FN Brno	CMBG FN Brno	Bezdicek M. et al
EPI_ISL_1402349	CMBG FN Brno	CMBG FN Brno	Bezdicek M. et al
EPI_ISL_1402369	Vyskov hospital	CMBG FN Brno	Bezdicek M. et al

EPI_ISL_492035	Instituto de Biologia do Exército	Laboratório Metabolismo Macromolecular FirminoTorres de Castro, Instituto de Biofísica Carlos Chagas Filho, Universidade Federal do Rio de Janeiro	Bianca Catarina Azevedo Cabral et al
EPI_ISL_492043	Instituto de Biologia do Exército	Laboratório Metabolismo Macromolecular FirminoTorres de Castro, Instituto de Biofísica Carlos Chagas Filho, Universidade Federal do Rio de Janeiro	Bianca Catarina Azevedo Cabral et al
EPI_ISL_492044	Instituto de Biologia do Exército	Laboratório Metabolismo Macromolecular FirminoTorres de Castro, Instituto de Biofísica Carlos Chagas Filho, Universidade Federal do Rio de Janeiro	Bianca Catarina Azevedo Cabral et al
EPI_ISL_492036	Instituto de Biologia do Exército	Laboratório Metabolismo Macromolecular FirminoTorres de Castro, Instituto de Biofísica Carlos Chagas Filho, Universidade Federal do Rio de Janeiro	Bianca Catarina Azevedo Cabral et al
EPI_ISL_576119	Laboratory, The Bio Arte Limited	Laboratory, The Bio Arte Limited	Biazzo et al
EPI_ISL_576121	Laboratory, The Bio Arte Limited	Laboratory, The Bio Arte Limited	Biazzo et al
EPI_ISL_576122	Laboratory, The Bio Arte Limited	Laboratory, The Bio Arte Limited	Biazzo et al
EPI_ISL_576123	Laboratory, The Bio Arte Limited	Laboratory, The Bio Arte Limited	Biazzo et al
EPI_ISL_576124	Laboratory, The Bio Arte Limited	Laboratory, The Bio Arte Limited	Biazzo et al
EPI_ISL_1629593	Clinical Microbiology, Infection Prevention and Control	Section for Molecular Diagnostics	Björn Hallström et al
EPI_ISL_1629603	Clinical Microbiology, Infection Prevention and Control	Section for Molecular Diagnostics	Björn Hallström et al
EPI_ISL_1629615	Clinical Microbiology, Infection Prevention and Control	Section for Molecular Diagnostics	Björn Hallström et al
EPI_ISL_1629655	Clinical Microbiology, Infection Prevention and Control	Section for Molecular Diagnostics	Björn Hallström et al
EPI_ISL_450209	Department of Virology	Department of Virology	Boehmer et al
EPI_ISL_450199	Department of Virology	Department of Virology	Boehmer et al
EPI_ISL_450200	Department of Virology	Department of Virology	Boehmer et al
EPI_ISL_450198	Department of Virology	Department of Virology	Boehmer et al
EPI_ISL_454057	unknown	Instituto Nacional de Saude (INSA)	Borges et al
EPI_ISL_454110	unknown	Instituto Nacional de Saude (INSA)	Borges et al
EPI_ISL_454172	unknown	Instituto Nacional de Saude (INSA)	Borges et al
EPI_ISL_454259	unknown	Instituto Nacional de Saude (INSA)	Borges et al
EPI_ISL_511103	Instituto Nacional de Saude (INSA)	Instituto Nacional de Saude (INSA)	Borges et al
EPI_ISL_511105	Instituto Nacional de Saude (INSA)	Instituto Nacional de Saude (INSA)	Borges et al
EPI_ISL_511142	Instituto Nacional de Saude (INSA)	Instituto Nacional de Saude (INSA)	Borges et al
EPI_ISL_511579	Instituto Nacional de Saude (INSA)	Instituto Nacional de Saude (INSA)	Borges et al
EPI_ISL_511190	Instituto Nacional de Saude (INSA) and Instituto Gulbenkian de Ciencia (IGC)	Instituto Nacional de Saude (INSA) and Instituto Gulbenkian de Ciencia (IGC)	Borges et al
EPI_ISL_693550	Instituto Nacional de Saude (INSA)	Instituto Nacional de Saude (INSA)	Borges et al
EPI_ISL_693596	Instituto Nacional de Saude (INSA)	Instituto Nacional de Saude (INSA)	Borges et al

EPI_ISL_693531	Instituto Nacional de Saude (INSA)	Instituto Nacional de Saude (INSA)	Borges et al
EPI_ISL_693618	Instituto Nacional de Saude (INSA)	Instituto Nacional de Saude (INSA)	Borges et al
EPI_ISL_693619	Instituto Nacional de Saude (INSA)	Instituto Nacional de Saude (INSA)	Borges et al
EPI_ISL_693532	Instituto Nacional de Saude (INSA)	Instituto Nacional de Saude (INSA)	Borges et al
EPI_ISL_693537	Hospital Vila Franca de Xira	Instituto Nacional de Saude (INSA)	Borges et al
EPI_ISL_693536	Hospital Vila Franca de Xira	Instituto Nacional de Saude (INSA)	Borges et al
EPI_ISL_693535	Hospital Vila Franca de Xira	Instituto Nacional de Saude (INSA)	Borges et al
EPI_ISL_732116	Instituto Nacional de Saude (INSA)	Instituto Nacional de Saude (INSA)	Borges et al
EPI_ISL_732179	Instituto Nacional de Saude (INSA) and Instituto Gulbenkian de Ciencia (IGC)	Instituto Nacional de Saude (INSA) and Instituto Gulbenkian de Ciencia (IGC)	Borges et al
EPI_ISL_732180	Instituto Nacional de Saude (INSA) and Instituto Gulbenkian de Ciencia (IGC)	Instituto Nacional de Saude (INSA) and Instituto Gulbenkian de Ciencia (IGC)	Borges et al
EPI_ISL_732183	Instituto Nacional de Saude (INSA) and Instituto Gulbenkian de Ciencia (IGC)	Instituto Nacional de Saude (INSA) and Instituto Gulbenkian de Ciencia (IGC)	Borges et al
EPI_ISL_732184	Instituto Nacional de Saude (INSA) and Instituto Gulbenkian de Ciencia (IGC)	Instituto Nacional de Saude (INSA) and Instituto Gulbenkian de Ciencia (IGC)	Borges et al
EPI_ISL_861535	Instituto Nacional de Saude (INSA)	Instituto Nacional de Saude (INSA)	Borges et al
EPI_ISL_941550	Instituto Nacional de Saude (INSA)	Instituto Nacional de Saude (INSA)	Borges et al
EPI_ISL_941552	Instituto Nacional de Saude (INSA)	Instituto Nacional de Saude (INSA)	Borges et al
EPI_ISL_941583	Instituto Nacional de Saude (INSA)	Instituto Nacional de Saude (INSA)	Borges et al
EPI_ISL_941896	Instituto Nacional de Saude (INSA) and Instituto Gulbenkian de Ciencia (IGC)	Instituto Nacional de Saude (INSA) and Instituto Gulbenkian de Ciencia (IGC)	Borges et al
EPI_ISL_1023463	Instituto Nacional de Saude (INSA)	Instituto Nacional de Saude (INSA)	Borges et al
EPI_ISL_1260860	Instituto Nacional de Saude (INSA)	Instituto Nacional de Saude (INSA)	Borges et al
EPI_ISL_1399630	Instituto Nacional de Saude (INSA) and BioSystems & Integrative Sciences Institute (BioISI) Genomics Unit, FCUL	Instituto Nacional de Saude (INSA) and BioSystems & Integrative Sciences Institute (BioISI) Genomics Unit, FCUL	Borges et al
EPI_ISL_1399631	Instituto Nacional de Saude (INSA) and Instituto Gulbenkian de Ciencia (IGC)	Instituto Nacional de Saude (INSA) and Instituto Gulbenkian de Ciencia (IGC)	Borges et al
EPI_ISL_1399632	Instituto Nacional de Saude (INSA) and Instituto Gulbenkian de Ciencia (IGC)	Instituto Nacional de Saude (INSA) and Instituto Gulbenkian de Ciencia (IGC)	Borges et al
EPI_ISL_428451	Guangdong Provincial Center for Diseases Control and Prevention;Guangdong Provincial Institute of Public Health	School of Public Health, The University of Hong Kong	Bosheng Li et al
EPI_ISL_1060884	DB Diagnosticos do Brasil	Instituto de Medicina Tropical de Sao Paulo	Brazil-UK Centre for Arbovirus Discovery Di
EPI_ISL_1060918	DB Diagnosticos do Brasil	Instituto de Medicina Tropical de Sao Paulo	Brazil-UK Centre for Arbovirus Discovery Di
EPI_ISL_1060943	DB Diagnosticos do Brasil	Instituto de Medicina Tropical de Sao Paulo	Brazil-UK Centre for Arbovirus Discovery Di
EPI_ISL_1060954	DB Diagnosticos do Brasil	Instituto de Medicina Tropical de Sao Paulo	Brazil-UK Centre for Arbovirus Discovery Di

agnosis Genomics et al
agnosis Genomics et al
agnosis Genomics et al
agnosis Genomics et al

EPI_ISL_1060926	DB Diagnosticos do Brasil	Instituto de Medicina Tropical de Sao Paulo	Brazil-UK Centre for Arbovirus Discovery Di
EPI_ISL_1060947	DB Diagnosticos do Brasil	Instituto de Medicina Tropical de Sao Paulo	Brazil-UK Centre for Arbovirus Discovery Di
EPI_ISL_1060889	CDL Laboratorio Santos e Vidal LTDA.	Instituto de Medicina Tropical de Sao Paulo	Brazil-UK Centre for Arbovirus Discovery Di
EPI_ISL_1060897	CDL Laboratorio Santos e Vidal LTDA.	Instituto de Medicina Tropical de Sao Paulo	Brazil-UK Centre for Arbovirus Discovery Di
EPI_ISL_1060880	CDL Laboratorio Santos e Vidal LTDA.	Instituto de Medicina Tropical de Sao Paulo	Brazil-UK Centre for Arbovirus Discovery Di
EPI_ISL_1064736	CDL Laboratorio Santos e Vidal LTDA.	Instituto de Medicina Tropical de Sao Paulo	Brazil-UK Centre for Arbovirus Discovery Di
EPI_ISL_1060892	CDL Laboratorio Santos e Vidal LTDA.	Instituto de Medicina Tropical de Sao Paulo	Brazil-UK Centre for Arbovirus Discovery Di
EPI_ISL_1060896	CDL Laboratorio Santos e Vidal LTDA.	Instituto de Medicina Tropical de Sao Paulo	Brazil-UK Centre for Arbovirus Discovery Di
EPI_ISL_1060931	CDL Laboratorio Santos e Vidal LTDA.	Instituto de Medicina Tropical de Sao Paulo	Brazil-UK Centre for Arbovirus Discovery Di
EPI_ISL_1060955	CDL Laboratorio Santos e Vidal LTDA.	Instituto de Medicina Tropical de Sao Paulo	Brazil-UK Centre for Arbovirus Discovery Di
EPI_ISL_1060956	CDL Laboratorio Santos e Vidal LTDA.	Instituto de Medicina Tropical de Sao Paulo	Brazil-UK Centre for Arbovirus Discovery Di
EPI_ISL_1060959	CDL Laboratorio Santos e Vidal LTDA.	Instituto de Medicina Tropical de Sao Paulo	Brazil-UK Centre for Arbovirus Discovery Di
EPI_ISL_1060898	CDL Laboratorio Santos e Vidal LTDA.	Instituto de Medicina Tropical de Sao Paulo	Brazil-UK Centre for Arbovirus Discovery Di
EPI_ISL_1060948	CDL Laboratorio Santos e Vidal LTDA.	Instituto de Medicina Tropical de Sao Paulo	Brazil-UK Centre for Arbovirus Discovery Di
EPI_ISL_1060911	CDL Laboratorio Santos e Vidal LTDA.	Instituto de Medicina Tropical de Sao Paulo	Brazil-UK Centre for Arbovirus Discovery Di
EPI_ISL_1060899	CDL Laboratorio Santos e Vidal LTDA.	Instituto de Medicina Tropical de Sao Paulo	Brazil-UK Centre for Arbovirus Discovery Di
EPI_ISL_1060915	CDL Laboratorio Santos e Vidal LTDA.	Instituto de Medicina Tropical de Sao Paulo	Brazil-UK Centre for Arbovirus Discovery Di
EPI_ISL_1060890	CDL Laboratorio Santos e Vidal LTDA.	Instituto de Medicina Tropical de Sao Paulo	Brazil-UK Centre for Arbovirus Discovery Di
EPI_ISL_1064745	CDL Laboratorio Santos e Vidal LTDA.	Instituto de Medicina Tropical de Sao Paulo	Brazil-UK Centre for Arbovirus Discovery Di
EPI_ISL_1060894	CDL Laboratorio Santos e Vidal LTDA.	Instituto de Medicina Tropical de Sao Paulo	Brazil-UK Centre for Arbovirus Discovery Di
EPI_ISL_1060944	CDL Laboratorio Santos e Vidal LTDA.	Instituto de Medicina Tropical de Sao Paulo	Brazil-UK Centre for Arbovirus Discovery Di
EPI_ISL_1060879	CDL Laboratorio Santos e Vidal LTDA.	Instituto de Medicina Tropical de Sao Paulo	Brazil-UK Centre for Arbovirus Discovery Di
EPI_ISL_1060908	CDL Laboratorio Santos e Vidal LTDA.	Instituto de Medicina Tropical de Sao Paulo	Brazil-UK Centre for Arbovirus Discovery Di
EPI_ISL_1060877	CDL Laboratorio Santos e Vidal LTDA.	Instituto de Medicina Tropical de Sao Paulo	Brazil-UK Centre for Arbovirus Discovery Di
EPI_ISL_1060921	CDL Laboratorio Santos e Vidal LTDA.	Instituto de Medicina Tropical de Sao Paulo	Brazil-UK Centre for Arbovirus Discovery Di
EPI_ISL_1060881	CDL Laboratorio Santos e Vidal LTDA.	Instituto de Medicina Tropical de Sao Paulo	Brazil-UK Centre for Arbovirus Discovery Di
EPI_ISL_1060907	CDL Laboratorio Santos e Vidal LTDA.	Instituto de Medicina Tropical de Sao Paulo	Brazil-UK Centre for Arbovirus Discovery Di
EPI_ISL_722139	DB Diagnosticos do Brasil	Laboratório de Parasitologia Médica - Instituto de Medicina Tropical - Universidade de São Paulo	Brazil-UK Centre for Arbovirus Discovery Di
EPI_ISL_722136	DB Diagnosticos do Brasil	Laboratório de Parasitologia Médica - Instituto de Medicina Tropical - Universidade de São Paulo	Brazil-UK Centre for Arbovirus Discovery Di

agnosis Genomics et al
agnosis Genomics et al
agnosis Genomics et al
agnosis Genomics et al
agnosis Genomics et al
agnosis Genomics et al
agnosis Genomics et al
agnosis Genomics et al
agnosis Genomics et al
agnosis Genomics et al
agnosis Genomics et al
agnosis Genomics et al
agnosis Genomics et al
agnosis Genomics et al
agnosis Genomics et al
agnosis Genomics et al
agnosis Genomics et al
agnosis Genomics et al
agnosis Genomics et al
agnosis Genomics et al
agnosis Genomics et al
agnosis Genomics et al
agnosis Genomics et al
agnosis Genomics et al
agnosis Genomics et al
agnosis Genomics et al
agnosis Genomics et al
agnosis Genomics et al
agnosis Genomics et al

EPI_ISL_1084726	DB Diagnosticos do Brasil	Laboratório de Parasitologia Médica - Instituto de Medicina Tropical - Universidade de São Paulo	Brazil-UK Centre for Arbovirus Discovery Di
EPI_ISL_1084730	DB Diagnosticos do Brasil	Laboratório de Parasitologia Médica - Instituto de Medicina Tropical - Universidade de São Paulo	Brazil-UK Centre for Arbovirus Discovery Di
EPI_ISL_672666	DB Diagnosticos do Brasil	Laboratório de Parasitologia Médica - Instituto de Medicina Tropical - Universidade de São Paulo	Brazil-UK Centre for Arbovirus Discovery Di
EPI_ISL_672667	DB Diagnosticos do Brasil	Laboratório de Parasitologia Médica - Instituto de Medicina Tropical - Universidade de São Paulo	Brazil-UK Centre for Arbovirus Discovery Di
EPI_ISL_722146	DB Diagnosticos do Brasil	Laboratório de Parasitologia Médica - Instituto de Medicina Tropical - Universidade de São Paulo	Brazil-UK Centre for Arbovirus Discovery Di
EPI_ISL_722140	DB Diagnosticos do Brasil	Laboratório de Parasitologia Médica - Instituto de Medicina Tropical - Universidade de São Paulo	Brazil-UK Centre for Arbovirus Discovery Di
EPI_ISL_904018	DB Diagnosticos do Brasil	Laboratório de Parasitologia Médica - Instituto de Medicina Tropical - Universidade de São Paulo	Brazil-UK Centre for Arbovirus Discovery Di
EPI_ISL_672672	DB Diagnosticos do Brasil	Laboratório de Parasitologia Médica - Instituto de Medicina Tropical - Universidade de São Paulo	Brazil-UK Centre for Arbovirus Discovery Di
EPI_ISL_904024	DB Diagnosticos do Brasil	Laboratório de Parasitologia Médica - Instituto de Medicina Tropical - Universidade de São Paulo	Brazil-UK Centre for Arbovirus Discovery Di
EPI_ISL_904025	DB Diagnosticos do Brasil	Laboratório de Parasitologia Médica - Instituto de Medicina Tropical - Universidade de São Paulo	Brazil-UK Centre for Arbovirus Discovery Di
EPI_ISL_904026	DB Diagnosticos do Brasil	Laboratório de Parasitologia Médica - Instituto de Medicina Tropical - Universidade de São Paulo	Brazil-UK Centre for Arbovirus Discovery Di
EPI_ISL_904028	DB Diagnosticos do Brasil	Laboratório de Parasitologia Médica - Instituto de Medicina Tropical - Universidade de São Paulo	Brazil-UK Centre for Arbovirus Discovery Di
EPI_ISL_904029	DB Diagnosticos do Brasil	Laboratório de Parasitologia Médica - Instituto de Medicina Tropical - Universidade de São Paulo	Brazil-UK Centre for Arbovirus Discovery Di
EPI_ISL_672670	DB Diagnosticos do Brasil	Laboratório de Parasitologia Médica - Instituto de Medicina Tropical - Universidade de São Paulo	Brazil-UK Centre for Arbovirus Discovery Di
EPI_ISL_672675	DB Diagnosticos do Brasil	Laboratório de Parasitologia Médica - Instituto de Medicina Tropical - Universidade de São Paulo	Brazil-UK Centre for Arbovirus Discovery Di
EPI_ISL_672679	DB Diagnosticos do Brasil	Laboratório de Parasitologia Médica - Instituto de Medicina Tropical - Universidade de São Paulo	Brazil-UK Centre for Arbovirus Discovery Di
EPI_ISL_672681	DB Diagnosticos do Brasil	Laboratório de Parasitologia Médica - Instituto de Medicina Tropical - Universidade de São Paulo	Brazil-UK Centre for Arbovirus Discovery Di
EPI_ISL_722148	DB Diagnosticos do Brasil	Laboratório de Parasitologia Médica - Instituto de Medicina Tropical - Universidade de São Paulo	Brazil-UK Centre for Arbovirus Discovery Di
EPI_ISL_722134	DB Diagnosticos do Brasil	Laboratório de Parasitologia Médica - Instituto de Medicina Tropical - Universidade de São Paulo	Brazil-UK Centre for Arbovirus Discovery Di
EPI_ISL_722135	DB Diagnosticos do Brasil	Laboratório de Parasitologia Médica - Instituto de Medicina Tropical - Universidade de São Paulo	Brazil-UK Centre for Arbovirus Discovery Di
EPI_ISL_722149	DB Diagnosticos do Brasil	Laboratório de Parasitologia Médica - Instituto de Medicina Tropical - Universidade de São Paulo	Brazil-UK Centre for Arbovirus Discovery Di
EPI_ISL_1009675	Diagnósticos da América - DASA	Instituto de Medicina Tropical de Sao Paulo	Brazil-UK Centre for Arbovirus Discovery Di

agnosis Genomics et al

agnosis Genomics et al

agnosis Genomics et al

agnosis Genomics et al

agnosis Genomics et al

agnosis Genomics et al

agnosis Genomics et al

agnosis Genomics et al

agnosis Genomics et al

agnosis Genomics et al

agnosis Genomics et al

agnosis Genomics et al

agnosis Genomics et al

agnosis Genomics et al

agnosis Genomics et al

agnosis Genomics et al

agnosis Genomics et al

agnosis Genomics et al

agnosis Genomics et al

agnosis Genomics et al

agnosis Genomics et al

agnosis Genomics et al

EPI_ISL_722147	DB Diagnosticos do Brasil	Laboratório de Parasitologia Médica - Instituto de Medicina Tropical - Universidade de São Paulo	Brazil-UK Centre for Arbovirus Discovery Di
EPI_ISL_672684	DB Diagnosticos do Brasil	Laboratório de Parasitologia Médica - Instituto de Medicina Tropical - Universidade de São Paulo	Brazil-UK Centre for Arbovirus Discovery Di
EPI_ISL_722144	DB Diagnosticos do Brasil	Laboratório de Parasitologia Médica - Instituto de Medicina Tropical - Universidade de São Paulo	Brazil-UK Centre for Arbovirus Discovery Di
EPI_ISL_672705	Institute of Tropical Medicine at the University of São Paulo (IMT-USP)	Laboratório de Parasitologia Médica - Instituto de Medicina Tropical - Universidade de São Paulo	Brazil-UK Centre for Arbovirus Discovery Di
EPI_ISL_672712	Institute of Tropical Medicine at the University of São Paulo (IMT-USP)	Laboratório de Parasitologia Médica - Instituto de Medicina Tropical - Universidade de São Paulo	Brazil-UK Centre for Arbovirus Discovery Di
EPI_ISL_672720	Institute of Tropical Medicine at the University of São Paulo (IMT-USP)	Laboratório de Parasitologia Médica - Instituto de Medicina Tropical - Universidade de São Paulo	Brazil-UK Centre for Arbovirus Discovery Di
EPI_ISL_722004	Hospital das Clínicas Universidade de São Paulo Medical School	Laboratório de Parasitologia Médica - Instituto de Medicina Tropical - Universidade de São Paulo	Brazil-UK Centre for Arbovirus Discovery Di
EPI_ISL_722002	Hospital das Clínicas Universidade de São Paulo Medical School	Laboratório de Parasitologia Médica - Instituto de Medicina Tropical - Universidade de São Paulo	Brazil-UK Centre for Arbovirus Discovery Di
EPI_ISL_722040	Hospital das Clínicas Universidade de São Paulo Medical School	Laboratório de Parasitologia Médica - Instituto de Medicina Tropical - Universidade de São Paulo	Brazil-UK Centre for Arbovirus Discovery Di
EPI_ISL_722022	Hospital das Clínicas Universidade de São Paulo Medical School	Laboratório de Parasitologia Médica - Instituto de Medicina Tropical - Universidade de São Paulo	Brazil-UK Centre for Arbovirus Discovery Di
EPI_ISL_721995	Hospital das Clínicas Universidade de São Paulo Medical School	Laboratório de Parasitologia Médica - Instituto de Medicina Tropical - Universidade de São Paulo	Brazil-UK Centre for Arbovirus Discovery Di
EPI_ISL_722010	Hospital das Clínicas Universidade de São Paulo Medical School	Laboratório de Parasitologia Médica - Instituto de Medicina Tropical - Universidade de São Paulo	Brazil-UK Centre for Arbovirus Discovery Di
EPI_ISL_722017	Hospital das Clínicas Universidade de São Paulo Medical School	Laboratório de Parasitologia Médica - Instituto de Medicina Tropical - Universidade de São Paulo	Brazil-UK Centre for Arbovirus Discovery Di
EPI_ISL_1171871	IMT_USP	Laboratório de Parasitologia Médica - Instituto de Medicina Tropical - Universidade de São Paulo	Brazil-UK Centre for Arbovirus Discovery Di
EPI_ISL_1084729	IMT_USP	Laboratório de Parasitologia Médica - Instituto de Medicina Tropical - Universidade de São Paulo	Brazil-UK Centre for Arbovirus Discovery Di
EPI_ISL_525996	OHSU Lab Services Molecular Microbiology Lab	Oregon SARS-CoV-2 Genome Sequencing Center	Brendan L. O'Connell et al
EPI_ISL_1167936	PA Department of Health, Bureau of Laboratories	Genomics and Discovery, Respiratory Viruses Branch, Division of Viral Diseases, Centers for Disease Control and Prevention	Brian Lynch et al
EPI_ISL_1492545	Institute of Virology, Biomedical Research Center of the Slovak Academy of Sciences, Bratislava	Faculty of Natural Sciences, Comenius University, Bratislava	Brona Brejova et al
EPI_ISL_903986	Institute of Virology, Biomedical Research Center of the Slovak Academy of Sciences, Bratislava	Faculty of Natural Sciences, Comenius University, Bratislava	Broňa Brejová et al
EPI_ISL_959632	Institute of Virology, Biomedical Research Center of the Slovak Academy of Sciences, Bratislava	Faculty of Natural Sciences, Comenius University, Bratislava	Broňa Brejová et al
EPI_ISL_1239415	Institute of Virology, Biomedical Research Center of the Slovak Academy of Sciences, Bratislava	Faculty of Natural Sciences, Comenius University, Bratislava	Broňa Brejová et al
EPI_ISL_577740	Institute of Virology, Biomedical Research Center of the Slovak Academy of Sciences, Bratislava	Faculty of Natural Sciences, Comenius University, Bratislava	Broňa Brejová et al

agnosis Genomics et al

agnosis Genomics et al

agnosis Genomics et al

agnosis Genomics et al

agnosis Genomics et al

agnosis Genomics et al

agnosis Genomics et al

agnosis Genomics et al

agnosis Genomics et al

agnosis Genomics et al

agnosis Genomics et al

agnosis Genomics et al

agnosis Genomics et al

agnosis Genomics et al

agnosis Genomics et al

EPI_ISL_577741	Institute of Virology, Biomedical Research Center of the Slovak Academy of Sciences, Bratislava	Faculty of Natural Sciences, Comenius University, Bratislava	Broňa Brejová et al
EPI_ISL_583487	Institute of Virology, Biomedical Research Center of the Slovak Academy of Sciences, Bratislava	Faculty of Natural Sciences, Comenius University, Bratislava	Broňa Brejová et al
EPI_ISL_1405498	Johns Hopkins Hospital Department of Pathology	Johns Hopkins Hospital Department of Pathology	C. Paul Morris et al
EPI_ISL_671343	National Virus Reference Laboratory	Irish Coronavirus Sequencing Consortium - Teagasc Moorepark	Calm Walsh et al
EPI_ISL_767724	National Virus Reference Laboratory	Irish Coronavirus Sequencing Consortium - Teagasc Moorepark	Calum Walsh et al
EPI_ISL_671381	National Virus Reference Laboratory	Irish Coronavirus Sequencing Consortium - Teagasc Moorepark	Calum Walsh et al
EPI_ISL_671400	National Virus Reference Laboratory	Irish Coronavirus Sequencing Consortium - Teagasc Moorepark	Calum Walsh et al
EPI_ISL_562239	Victorian Infectious Diseases Reference Laboratory (VIDRL)	VIDRL and MDU-PHL	Caly et al
EPI_ISL_480778	Victorian Infectious Diseases Reference Laboratory (VIDRL)	VIDRL and MDU-PHL	Caly L. et al
EPI_ISL_480779	Victorian Infectious Diseases Reference Laboratory (VIDRL)	VIDRL and MDU-PHL	Caly L. et al
EPI_ISL_480781	Victorian Infectious Diseases Reference Laboratory (VIDRL)	VIDRL and MDU-PHL	Caly L. et al
EPI_ISL_430637	Victorian Infectious Diseases Reference Laboratory (VIDRL)	Microbiological Diagnostic Unit Public Health Laboratory and Victorian Infectious Diseases Reference Laboratory, The Peter Doherty Institute for Infection and Immunity	Caly L. et al
EPI_ISL_419733	Victorian Infectious Diseases Reference Laboratory (VIDRL)	Victorian Infectious Diseases Reference Laboratory and Microbiological Diagnostic Unit Public Health Laboratory, Doherty Institute	Caly L. et al
EPI_ISL_419735	Victorian Infectious Diseases Reference Laboratory (VIDRL)	Victorian Infectious Diseases Reference Laboratory and Microbiological Diagnostic Unit Public Health Laboratory, Doherty Institute	Caly L. et al
EPI_ISL_456535	Victorian Infectious Diseases Reference Laboratory (VIDRL)	Microbiological Diagnostic Unit Public Health Laboratory and Victorian Infectious Diseases Reference Laboratory, Doherty Institute	Caly L. et al
EPI_ISL_456552	Victorian Infectious Diseases Reference Laboratory (VIDRL)	Microbiological Diagnostic Unit Public Health Laboratory and Victorian Infectious Diseases Reference Laboratory, Doherty Institute	Caly L. et al
EPI_ISL_456571	Victorian Infectious Diseases Reference Laboratory (VIDRL)	Microbiological Diagnostic Unit Public Health Laboratory and Victorian Infectious Diseases Reference Laboratory, Doherty Institute	Caly L. et al
EPI_ISL_456633	Victorian Infectious Diseases Reference Laboratory (VIDRL)	Microbiological Diagnostic Unit Public Health Laboratory and Victorian Infectious Diseases Reference Laboratory, Doherty Institute	Caly L. et al
EPI_ISL_456635	Victorian Infectious Diseases Reference Laboratory (VIDRL)	Microbiological Diagnostic Unit Public Health Laboratory and Victorian Infectious Diseases Reference Laboratory, Doherty Institute	Caly L. et al
EPI_ISL_779617	Victorian Infectious Diseases Reference Laboratory (VIDRL)	VIDRL and MDU-PHL	Caly L. et al
EPI_ISL_521976	Victorian Infectious Diseases Reference Laboratory (VIDRL)	VIDRL and MDU-PHL	Caly L. et al
EPI_ISL_520762	Victorian Infectious Diseases Reference Laboratory (VIDRL)	VIDRL and MDU-PHL	Caly L. et al
EPI_ISL_1396344	Laboratorio del Hospital Regional Ushuaia Gdor. Ernesto Campos	Nodo de Secuenciación Tierra del Fuego - Hospital Regional Ushuaia - Centro Austral De Investigaciones Científicas - Universidad Nacional De Tierra Del Fuego on behalf of 'Proyecto Argentino Interinstitucional de genomica de SARS-CoV-2' (PAIS Consortium)	Carina Andrea De Roccis et al

EPI_ISL_1396351	Laboratorio del Hospital Regional Ushuaia Gdor. Ernesto Campos	Nodo de Secuenciación Tierra del Fuego - Hospital Regional Ushuaia - Centro Austral De Investigaciones Científicas - Universidad Nacional De Tierra Del Fuego on behalf of 'Proyecto Argentino Interinstitucional de genomica de SARS-CoV-2' (PAIS Consortium)	Carina Andrea De Roccis et al
EPI_ISL_1396353	Laboratorio del Hospital Regional Ushuaia Gdor. Ernesto Campos	Nodo de Secuenciación Tierra del Fuego - Hospital Regional Ushuaia - Centro Austral De Investigaciones Científicas - Universidad Nacional De Tierra Del Fuego on behalf of 'Proyecto Argentino Interinstitucional de genomica de SARS-CoV-2' (PAIS Consortium)	Carina Andrea De Roccis et al
EPI_ISL_1396356	Laboratorio del Hospital Regional Ushuaia Gdor. Ernesto Campos	Nodo de Secuenciación Tierra del Fuego - Hospital Regional Ushuaia - Centro Austral De Investigaciones Científicas - Universidad Nacional De Tierra Del Fuego on behalf of 'Proyecto Argentino Interinstitucional de genomica de SARS-CoV-2' (PAIS Consortium)	Carina Andrea De Roccis et al
EPI_ISL_691625	Servicio de Microbiología, Hospital Universitario Son Espases	SeqCOVID-SPAIN consortium/IBV(CSIC)	Carla López-Causapé et al
EPI_ISL_431833	National Institutes of Health, University of the Philippines Manila	Philippine Genome Center, University of the Philippines System	Carlo M. Lapid et al
EPI_ISL_434554	National Institutes of Health, University of the Philippines Manila	Philippine Genome Center	Carlo M. Lapid et al
EPI_ISL_434555	National Institutes of Health, University of the Philippines Manila	Philippine Genome Center	Carlo M. Lapid et al
EPI_ISL_434556	National Institutes of Health, University of the Philippines Manila	Philippine Genome Center	Carlo M. Lapid et al
EPI_ISL_434557	National Institutes of Health, University of the Philippines Manila	Philippine Genome Center	Carlo M. Lapid et al
EPI_ISL_649155	Laboratorio de Investigaciones de Baney	University Hospital Basel, Clinical Bacteriology	Carlos Cortes et al
EPI_ISL_648305	Laboratorio de Investigaciones de Baney	University Hospital Basel, Clinical Bacteriology	Carlos Cortes et al
EPI_ISL_953402	Laboratorio de Investigaciones de Baney	Swiss Tropical and Public Health Institute	Carlos Cortes et al
EPI_ISL_648311	Laboratorio de Investigaciones de Baney	University Hospital Basel, Clinical Bacteriology	Carlos Cortes et al
EPI_ISL_649160	Laboratorio de Investigaciones de Baney	University Hospital Basel, Clinical Bacteriology	Carlos Cortes et al
EPI_ISL_648314	Laboratorio de Investigaciones de Baney	University Hospital Basel, Clinical Bacteriology	Carlos Cortes et al
EPI_ISL_648364	Laboratorio de Investigaciones de Baney	University Hospital Basel, Clinical Bacteriology	Carlos Cortes et al
EPI_ISL_953405	Laboratorio de Investigaciones de Baney	Swiss Tropical and Public Health Institute	Carlos Cortes et al
EPI_ISL_648368	Laboratorio de Investigaciones de Baney	University Hospital Basel, Clinical Bacteriology	Carlos Cortes et al
EPI_ISL_648316	Laboratorio de Investigaciones de Baney	University Hospital Basel, Clinical Bacteriology	Carlos Cortes et al
EPI_ISL_648318	Laboratorio de Investigaciones de Baney	University Hospital Basel, Clinical Bacteriology	Carlos Cortes et al
EPI_ISL_648319	Laboratorio de Investigaciones de Baney	University Hospital Basel, Clinical Bacteriology	Carlos Cortes et al
EPI_ISL_953412	Laboratorio de Investigaciones de Baney	Swiss Tropical and Public Health Institute	Carlos Cortes et al
EPI_ISL_648323	Laboratorio de Investigaciones de Baney	University Hospital Basel, Clinical Bacteriology	Carlos Cortes et al
EPI_ISL_953408	Laboratorio de Investigaciones de Baney	Swiss Tropical and Public Health Institute	Carlos Cortes et al
EPI_ISL_648329	Laboratorio de Investigaciones de Baney	University Hospital Basel, Clinical Bacteriology	Carlos Cortes et al
EPI_ISL_648332	Laboratorio de Investigaciones de Baney	University Hospital Basel, Clinical Bacteriology	Carlos Cortes et al

EPI_ISL_648378	Laboratorio de Investigaciones de Baney	University Hospital Basel, Clinical Bacteriology	Carlos Cortes et al
EPI_ISL_648337	Laboratorio de Investigaciones de Baney	University Hospital Basel, Clinical Bacteriology	Carlos Cortes et al
EPI_ISL_953411	Laboratorio de Investigaciones de Baney	Swiss Tropical and Public Health Institute	Carlos Cortes et al
EPI_ISL_648338	Laboratorio de Investigaciones de Baney	University Hospital Basel, Clinical Bacteriology	Carlos Cortes et al
EPI_ISL_648340	Laboratorio de Investigaciones de Baney	University Hospital Basel, Clinical Bacteriology	Carlos Cortes et al
EPI_ISL_953403	Laboratorio de Investigaciones de Baney	Swiss Tropical and Public Health Institute	Carlos Cortes et al
EPI_ISL_648349	Laboratorio de Investigaciones de Baney	University Hospital Basel, Clinical Bacteriology	Carlos Cortes et al
EPI_ISL_648355	Laboratorio de Investigaciones de Baney	University Hospital Basel, Clinical Bacteriology	Carlos Cortes et al
EPI_ISL_1093178	Laboratorio de Referencia Nacional de Virus Respiratorio. Instituto Nacional de Salud Perú	Laboratorio de Referencia Nacional de Biotecnología y Biología Molecular. Instituto Nacional de Salud Perú	Carlos Padilla Rojas et al
EPI_ISL_1534641	Laboratorio de Referencia Nacional de Virus Respiratorio. Instituto Nacional de Salud Perú	Laboratorio de Referencia Nacional de Biotecnología y Biología Molecular. Instituto Nacional de Salud Perú	Carlos Padilla Rojas et al
EPI_ISL_1532151	Laboratorio de Referencia Nacional de Virus Respiratorio. Instituto Nacional de Salud Perú	Laboratorio de Referencia Nacional de Biotecnología y Biología Molecular. Instituto Nacional de Salud Perú	Carlos Padilla Rojas et al
EPI_ISL_1532158	Laboratorio de Referencia Nacional de Virus Respiratorio. Instituto Nacional de Salud Perú	Laboratorio de Referencia Nacional de Biotecnología y Biología Molecular. Instituto Nacional de Salud Perú	Carlos Padilla Rojas et al
EPI_ISL_1534653	Laboratorio de Referencia Nacional de Virus Respiratorio. Instituto Nacional de Salud Perú	Laboratorio de Referencia Nacional de Biotecnología y Biología Molecular. Instituto Nacional de Salud Perú	Carlos Padilla Rojas et al
EPI_ISL_1534655	Laboratorio de Referencia Nacional de Virus Respiratorio. Instituto Nacional de Salud Perú	Laboratorio de Referencia Nacional de Biotecnología y Biología Molecular. Instituto Nacional de Salud Perú	Carlos Padilla Rojas et al
EPI_ISL_1093162	Laboratorio de Referencia Nacional de Virus Respiratorio. Instituto Nacional de Salud Perú	Laboratorio de Referencia Nacional de Biotecnología y Biología Molecular. Instituto Nacional de Salud Perú	Carlos Padilla Rojas et al
EPI_ISL_1532197	Laboratorio de Referencia Nacional de Virus Respiratorio. Instituto Nacional de Salud Perú	Laboratorio de Referencia Nacional de Biotecnología y Biología Molecular. Instituto Nacional de Salud Perú	Carlos Padilla Rojas et al
EPI_ISL_517531	Laboratorio de Referencia Nacional de Virus Respiratorio. Centro Nacional de Salud Publica. Instituto Nacional de Salud Peru.	Laboratorio de Referencia Nacional de Biotecnología y Biología Molecular. Centro Nacional de Salud Publica. Instituto Nacional de Salud Peru.	Carlos Padilla Rojas et al
EPI_ISL_516647	Laboratorio de Referencia Nacional de Virus Respiratorio. Centro Nacional de Salud Publica. Instituto Nacional de Salud Peru.	Laboratorio de Referencia Nacional de Biotecnología y Biología Molecular. Centro Nacional de Salud Publica. Instituto Nacional de Salud Peru.	Carlos Padilla Rojas et al
EPI_ISL_1532163	Laboratorio de Referencia Nacional de Virus Respiratorio. Instituto Nacional de Salud Perú	Laboratorio de Referencia Nacional de Biotecnología y Biología Molecular. Instituto Nacional de Salud Perú	Carlos Padilla Rojas et al
EPI_ISL_1532164	Laboratorio de Referencia Nacional de Virus Respiratorio. Instituto Nacional de Salud Perú	Laboratorio de Referencia Nacional de Biotecnología y Biología Molecular. Instituto Nacional de Salud Perú	Carlos Padilla Rojas et al
EPI_ISL_1093172	Laboratorio de Referencia Nacional de Virus Respiratorio. Instituto Nacional de Salud Perú	Laboratorio de Referencia Nacional de Biotecnología y Biología Molecular. Instituto Nacional de Salud Perú	Carlos Padilla Rojas et al
EPI_ISL_1532199	Laboratorio de Referencia Nacional de Virus Respiratorio. Instituto Nacional de Salud Perú	Laboratorio de Referencia Nacional de Biotecnología y Biología Molecular. Instituto Nacional de Salud Perú	Carlos Padilla Rojas et al
EPI_ISL_489988	Laboratorio de Referencia Nacional de Virus Respiratorio. Instituto Nacional de Salud Perú	Laboratorio de Referencia Nacional de Biotecnología y Biología Molecular. Instituto Nacional de Salud Perú	Carlos Padilla Rojas et al
EPI_ISL_491427	Laboratorio de Referencia Nacional de Virus Respiratorio. Instituto Nacional de Salud Perú	Laboratorio de Referencia Nacional de Biotecnología y Biología Molecular. Instituto Nacional de Salud Perú	Carlos Padilla Rojas et al

EPI_ISL_491429	Laboratorio de Referencia Nacional de Virus Respiratorio. Instituto Nacional de Salud Perú	Laboratorio de Referencia Nacional de Biotecnología y Biología Molecular. Instituto Nacional de Salud Perú	Carlos Padilla Rojas et al
EPI_ISL_491430	Laboratorio de Referencia Nacional de Virus Respiratorio. Instituto Nacional de Salud Perú	Laboratorio de Referencia Nacional de Biotecnología y Biología Molecular. Instituto Nacional de Salud Perú	Carlos Padilla Rojas et al
EPI_ISL_491432	Laboratorio de Referencia Nacional de Virus Respiratorio. Instituto Nacional de Salud.Perú	Laboratorio de Referencia Nacional de Biotecnología y Biología Molecular. Instituto Nacional de Salud.Perú	Carlos Padilla Rojas et al
EPI_ISL_517959	Laboratorio de Referencia Nacional de Virus Respiratorio. Centro Nacional de Salud Publica. Instituto Nacional de Salud Peru	Laboratorio de Referencia Nacional de Biotecnología y Biología Molecular. Centro Nacional de Salud Publica. Instituto Nacional de Salud Peru.	Carlos Padilla Rojas et al
EPI_ISL_516722	Laboratorio de Referencia Nacional de Virus Respiratorio. Centro Nacional de Salud Publica. Instituto Nacional de Salud Peru.	Laboratorio de Referencia Nacional de Biotecnología y Biología Molecular. Centro Nacional de Salud Publica. Instituto Nacional de Salud Peru.	Carlos Padilla Rojas et al
EPI_ISL_1092344	Laboratorio de Referencia Nacional de Virus Respiratorio. Instituto Nacional de Salud Perú	Laboratorio de Referencia Nacional de Biotecnología y Biología Molecular. Instituto Nacional de Salud Perú	Carlos Padilla Rojas et al
EPI_ISL_1092336	Laboratorio de Referencia Nacional de Virus Respiratorio. Instituto Nacional de Salud Perú	Laboratorio de Referencia Nacional de Biotecnología y Biología Molecular. Instituto Nacional de Salud Perú	Carlos Padilla Rojas et al
EPI_ISL_1092353	Laboratorio de Referencia Nacional de Virus Respiratorio. Instituto Nacional de Salud Perú	Laboratorio de Referencia Nacional de Biotecnología y Biología Molecular. Instituto Nacional de Salud Perú	Carlos Padilla Rojas et al
EPI_ISL_1093157	Laboratorio de Referencia Nacional de Virus Respiratorio. Instituto Nacional de Salud Perú	Laboratorio de Referencia Nacional de Biotecnología y Biología Molecular. Instituto Nacional de Salud Perú	Carlos Padilla Rojas et al
EPI_ISL_1137488	Laboratorio de Referencia Nacional de Virus Respiratorio. Instituto Nacional de Salud Perú	Laboratorio de Referencia Nacional de Biotecnología y Biología Molecular. Instituto Nacional de Salud Perú	Carlos Padilla Rojas et al
EPI_ISL_1137489	Laboratorio de Referencia Nacional de Virus Respiratorio. Instituto Nacional de Salud Perú	Laboratorio de Referencia Nacional de Biotecnología y Biología Molecular. Instituto Nacional de Salud Perú	Carlos Padilla Rojas et al
EPI_ISL_1664258	Laboratorio de Referencia Nacional de Virus Respiratorio. Instituto Nacional de Salud Perú	Laboratorio de Referencia Nacional de Biotecnología y Biología Molecular. Instituto Nacional de Salud Perú	Carlos Padilla Rojas et al
EPI_ISL_1532206	Laboratorio de Referencia Nacional de Virus Respiratorio. Instituto Nacional de Salud Perú	Laboratorio de Referencia Nacional de Biotecnología y Biología Molecular. Instituto Nacional de Salud Perú	Carlos Padilla Rojas et al
EPI_ISL_1532225	Laboratorio de Referencia Nacional de Virus Respiratorio. Instituto Nacional de Salud Perú	Laboratorio de Referencia Nacional de Biotecnología y Biología Molecular. Instituto Nacional de Salud Perú	Carlos Padilla Rojas et al
EPI_ISL_1532226	Laboratorio de Referencia Nacional de Virus Respiratorio. Instituto Nacional de Salud Perú	Laboratorio de Referencia Nacional de Biotecnología y Biología Molecular. Instituto Nacional de Salud Perú	Carlos Padilla Rojas et al
EPI_ISL_1534627	Laboratorio de Referencia Nacional de Virus Respiratorio. Instituto Nacional de Salud Perú	Laboratorio de Referencia Nacional de Biotecnología y Biología Molecular. Instituto Nacional de Salud Perú	Carlos Padilla Rojas et al
EPI_ISL_1534629	Laboratorio de Referencia Nacional de Virus Respiratorio. Instituto Nacional de Salud Perú	Laboratorio de Referencia Nacional de Biotecnología y Biología Molecular. Instituto Nacional de Salud Perú	Carlos Padilla Rojas et al
EPI_ISL_1534630	Laboratorio de Referencia Nacional de Virus Respiratorio. Instituto Nacional de Salud Perú	Laboratorio de Referencia Nacional de Biotecnología y Biología Molecular. Instituto Nacional de Salud Perú	Carlos Padilla Rojas et al
EPI_ISL_756293	Center for Biotechnology and Cell Therapy, São Rafael Hospital, Salvador, Brazil	Center for Biotechnology and Cell Therapy, São Rafael Hospital, Salvador, Brazil	Carolina Kymie Vasques Nonaka et al
EPI_ISL_756294	Center for Biotechnology and Cell Therapy, São Rafael Hospital, Salvador, Brazil	Center for Biotechnology and Cell Therapy, São Rafael Hospital, Salvador, Brazil	Carolina Kymie Vasques Nonaka et al
EPI_ISL_1608170	Hospital São Rafael - IDOR	Hospital São Rafael - IDOR	Carolina Kymie Vasques Nonaka et al
EPI_ISL_623104	Simile Medicina Diagnóstica	Bioinformatics Laboratory / LNCC	Carolina M Voloch et al
EPI_ISL_623105	Simile Medicina Diagnóstica	Bioinformatics Laboratory / LNCC	Carolina M Voloch et al

EPI_ISL_623107	Laboratorio de Virologia Molecular / UFRJ	Bioinformatics Laboratory / LNCC	Carolina M Voloch et al
EPI_ISL_623113	Laboratorio de Virologia Molecular / UFRJ	Bioinformatics Laboratory / LNCC	Carolina M Voloch et al
EPI_ISL_623121	Laboratorio de Virologia Molecular / UFRJ	Bioinformatics Laboratory / LNCC	Carolina M Voloch et al
EPI_ISL_623125	Laboratorio de Virologia Molecular / UFRJ	Bioinformatics Laboratory / LNCC	Carolina M Voloch et al
EPI_ISL_623130	Laboratorio de Virologia Molecular / UFRJ	Bioinformatics Laboratory / LNCC	Carolina M Voloch et al
EPI_ISL_623131	Laboratorio de Virologia Molecular / UFRJ	Bioinformatics Laboratory / LNCC	Carolina M Voloch et al
EPI_ISL_623151	Laboratorio de Virologia Molecular / UFRJ	Bioinformatics Laboratory / LNCC	Carolina M Voloch et al
EPI_ISL_623155	Laboratorio de Virologia Molecular / UFRJ	Bioinformatics Laboratory / LNCC	Carolina M Voloch et al
EPI_ISL_623159	Laboratorio de Virologia Molecular / UFRJ	Bioinformatics Laboratory / LNCC	Carolina M Voloch et al
EPI_ISL_623164	Laboratorio de Virologia Molecular / UFRJ	Bioinformatics Laboratory / LNCC	Carolina M Voloch et al
EPI_ISL_717807	Laboratorio de Virologia Molecular / UFRJ	Bioinformatics Laboratory / LNCC	Carolina M Voloch et al
EPI_ISL_717808	Laboratorio de Virologia Molecular / UFRJ	Bioinformatics Laboratory / LNCC	Carolina M Voloch et al
EPI_ISL_717810	Laboratorio de Virologia Molecular / UFRJ	Bioinformatics Laboratory / LNCC	Carolina M Voloch et al
EPI_ISL_717811	Laboratorio de Virologia Molecular / UFRJ	Bioinformatics Laboratory / LNCC	Carolina M Voloch et al
EPI_ISL_717812	Laboratorio de Virologia Molecular / UFRJ	Bioinformatics Laboratory / LNCC	Carolina M Voloch et al
EPI_ISL_717813	Laboratorio de Virologia Molecular / UFRJ	Bioinformatics Laboratory / LNCC	Carolina M Voloch et al
EPI_ISL_717814	Laboratorio de Virologia Molecular / UFRJ	Bioinformatics Laboratory / LNCC	Carolina M Voloch et al
EPI_ISL_717815	Laboratorio de Virologia Molecular / UFRJ	Bioinformatics Laboratory / LNCC	Carolina M Voloch et al
EPI_ISL_717816	Laboratorio de Virologia Molecular / UFRJ	Bioinformatics Laboratory / LNCC	Carolina M Voloch et al
EPI_ISL_717817	Laboratorio de Virologia Molecular / UFRJ	Bioinformatics Laboratory / LNCC	Carolina M Voloch et al
EPI_ISL_717819	Laboratorio de Virologia Molecular / UFRJ	Bioinformatics Laboratory / LNCC	Carolina M Voloch et al
EPI_ISL_717821	Laboratorio de Virologia Molecular / UFRJ	Bioinformatics Laboratory / LNCC	Carolina M Voloch et al
EPI_ISL_717823	Laboratorio de Virologia Molecular / UFRJ	Bioinformatics Laboratory / LNCC	Carolina M Voloch et al
EPI_ISL_717825	Laboratorio de Virologia Molecular / UFRJ	Bioinformatics Laboratory / LNCC	Carolina M Voloch et al
EPI_ISL_717826	Laboratorio de Virologia Molecular / UFRJ	Bioinformatics Laboratory / LNCC	Carolina M Voloch et al
EPI_ISL_717827	Laboratorio de Virologia Molecular / UFRJ	Bioinformatics Laboratory / LNCC	Carolina M Voloch et al
EPI_ISL_717828	Laboratorio de Virologia Molecular / UFRJ	Bioinformatics Laboratory / LNCC	Carolina M Voloch et al
EPI_ISL_717830	Laboratorio de Virologia Molecular / UFRJ	Bioinformatics Laboratory / LNCC	Carolina M Voloch et al
EPI_ISL_717787	LACEN RJ - Noel Nutels	Bioinformatics Laboratory / LNCC	Carolina M Voloch et al
EPI_ISL_717794	LACEN RJ - Noel Nutels	Bioinformatics Laboratory / LNCC	Carolina M Voloch et al

EPI_ISL_717832	LACEN Dr. Francisco Rimolo Neto	Bioinformatics Laboratory / LNCC	Carolina M Voloch et al
EPI_ISL_717835	LACEN Dr. Francisco Rimolo Neto	Bioinformatics Laboratory / LNCC	Carolina M Voloch et al
EPI_ISL_717851	Laboratorio de Virologia Molecular / UFRJ	Bioinformatics Laboratory / LNCC	Carolina M Voloch et al
EPI_ISL_717857	Laboratorio de Virologia Molecular / UFRJ	Bioinformatics Laboratory / LNCC	Carolina M Voloch et al
EPI_ISL_717863	Laboratorio de Virologia Molecular / UFRJ	Bioinformatics Laboratory / LNCC	Carolina M Voloch et al
EPI_ISL_717869	Laboratorio de Virologia Molecular / UFRJ	Bioinformatics Laboratory / LNCC	Carolina M Voloch et al
EPI_ISL_717870	Laboratorio de Virologia Molecular / UFRJ	Bioinformatics Laboratory / LNCC	Carolina M Voloch et al
EPI_ISL_717871	Laboratorio de Virologia Molecular / UFRJ	Bioinformatics Laboratory / LNCC	Carolina M Voloch et al
EPI_ISL_717872	Laboratorio de Virologia Molecular / UFRJ	Bioinformatics Laboratory / LNCC	Carolina M Voloch et al
EPI_ISL_717873	Laboratorio de Virologia Molecular / UFRJ	Bioinformatics Laboratory / LNCC	Carolina M Voloch et al
EPI_ISL_717793	Laboratorio de Virologia Molecular / UFRJ	Bioinformatics Laboratory / LNCC	Carolina M Voloch et al
EPI_ISL_717877	Laboratorio de Virologia Molecular / UFRJ	Bioinformatics Laboratory / LNCC	Carolina M Voloch et al
EPI_ISL_717878	Laboratorio de Virologia Molecular / UFRJ	Bioinformatics Laboratory / LNCC	Carolina M Voloch et al
EPI_ISL_717887	Laboratorio de Virologia Molecular / UFRJ	Bioinformatics Laboratory / LNCC	Carolina M Voloch et al
EPI_ISL_717889	Laboratorio de Virologia Molecular / UFRJ	Bioinformatics Laboratory / LNCC	Carolina M Voloch et al
EPI_ISL_717890	Laboratorio de Virologia Molecular / UFRJ	Bioinformatics Laboratory / LNCC	Carolina M Voloch et al
EPI_ISL_717891	Laboratorio de Virologia Molecular / UFRJ	Bioinformatics Laboratory / LNCC	Carolina M Voloch et al
EPI_ISL_717893	Laboratorio de Virologia Molecular / UFRJ	Bioinformatics Laboratory / LNCC	Carolina M Voloch et al
EPI_ISL_717894	Laboratorio de Virologia Molecular / UFRJ	Bioinformatics Laboratory / LNCC	Carolina M Voloch et al
EPI_ISL_717895	Laboratorio de Virologia Molecular / UFRJ	Bioinformatics Laboratory / LNCC	Carolina M Voloch et al
EPI_ISL_717896	Laboratorio de Virologia Molecular / UFRJ	Bioinformatics Laboratory / LNCC	Carolina M Voloch et al
EPI_ISL_717900	LACEN RJ - Noel Nutels	Bioinformatics Laboratory / LNCC	Carolina M Voloch et al
EPI_ISL_717901	LACEN RJ - Noel Nutels	Bioinformatics Laboratory / LNCC	Carolina M Voloch et al
EPI_ISL_717797	LACEN RJ - Noel Nutels	Bioinformatics Laboratory / LNCC	Carolina M Voloch et al
EPI_ISL_717788	LACEN RJ - Noel Nutels	Bioinformatics Laboratory / LNCC	Carolina M Voloch et al
EPI_ISL_717903	LACEN RJ - Noel Nutels	Bioinformatics Laboratory / LNCC	Carolina M Voloch et al
EPI_ISL_717904	LACEN RJ - Noel Nutels	Bioinformatics Laboratory / LNCC	Carolina M Voloch et al
EPI_ISL_717907	LACEN RJ - Noel Nutels	Bioinformatics Laboratory / LNCC	Carolina M Voloch et al
EPI_ISL_717908	LACEN RJ - Noel Nutels	Bioinformatics Laboratory / LNCC	Carolina M Voloch et al
EPI_ISL_717789	LACEN RJ - Noel Nutels	Bioinformatics Laboratory / LNCC	Carolina M Voloch et al

EPI_ISL_717916	LACEN Dr. Francisco Rimolo Neto	Bioinformatics Laboratory / LNCC	Carolina M Voloch et al
EPI_ISL_717919	LACEN Dr. Francisco Rimolo Neto	Bioinformatics Laboratory / LNCC	Carolina M Voloch et al
EPI_ISL_717785	LACEN RJ - Noel Nutels	Bioinformatics Laboratory / LNCC	Carolina M Voloch et al
EPI_ISL_717921	Laboratorio de Virologia Molecular / UFRJ	Bioinformatics Laboratory / LNCC	Carolina M Voloch et al
EPI_ISL_717922	Laboratorio de Virologia Molecular / UFRJ	Bioinformatics Laboratory / LNCC	Carolina M Voloch et al
EPI_ISL_717924	Laboratorio de Virologia Molecular / UFRJ	Bioinformatics Laboratory / LNCC	Carolina M Voloch et al
EPI_ISL_717925	Laboratorio de Virologia Molecular / UFRJ	Bioinformatics Laboratory / LNCC	Carolina M Voloch et al
EPI_ISL_717926	Laboratorio de Virologia Molecular / UFRJ	Bioinformatics Laboratory / LNCC	Carolina M Voloch et al
EPI_ISL_717929	Laboratorio de Virologia Molecular / UFRJ	Bioinformatics Laboratory / LNCC	Carolina M Voloch et al
EPI_ISL_717930	Laboratorio de Virologia Molecular / UFRJ	Bioinformatics Laboratory / LNCC	Carolina M Voloch et al
EPI_ISL_717931	Laboratorio de Virologia Molecular / UFRJ	Bioinformatics Laboratory / LNCC	Carolina M Voloch et al
EPI_ISL_717934	Laboratorio de Virologia Molecular / UFRJ	Bioinformatics Laboratory / LNCC	Carolina M Voloch et al
EPI_ISL_717935	Laboratorio de Virologia Molecular / UFRJ	Bioinformatics Laboratory / LNCC	Carolina M Voloch et al
EPI_ISL_717941	Laboratorio de Virologia Molecular / UFRJ	Bioinformatics Laboratory / LNCC	Carolina M Voloch et al
EPI_ISL_717944	Laboratorio de Virologia Molecular / UFRJ	Bioinformatics Laboratory / LNCC	Carolina M Voloch et al
EPI_ISL_717945	Laboratorio de Virologia Molecular / UFRJ	Bioinformatics Laboratory / LNCC	Carolina M Voloch et al
EPI_ISL_717946	Laboratorio de Virologia Molecular / UFRJ	Bioinformatics Laboratory / LNCC	Carolina M Voloch et al
EPI_ISL_717955	Laboratorio de Virologia Molecular / UFRJ	Bioinformatics Laboratory / LNCC	Carolina M Voloch et al
EPI_ISL_717957	Laboratorio de Virologia Molecular / UFRJ	Bioinformatics Laboratory / LNCC	Carolina M Voloch et al
EPI_ISL_717961	Laboratorio de Virologia Molecular / UFRJ	Bioinformatics Laboratory / LNCC	Carolina M Voloch et al
EPI_ISL_717963	LACEN Dr. Francisco Rimolo Neto	Bioinformatics Laboratory / LNCC	Carolina M Voloch et al
EPI_ISL_1632717	Klinisk mikrobiologi, Region Västerbotten	CBRN Defence and Security, Swedish Defence Research Agency	Caroline Öhrman et al
EPI_ISL_960703	Germano de sousa	Instituto Gulbenkian de Ciencia	Cathy Paulino et al
EPI_ISL_960728	Germano de sousa	Instituto Gulbenkian de Ciencia	Cathy Paulino et al
EPI_ISL_515911	California Department of Public Health	California Department of Public Health	CDPH IDLB COVIDNet et al
EPI_ISL_792467	Laboratorio del Hospital Regional Ushuaia Gdor. Ernesto Campos	Hospital Regional Ushuaia - Centro Austral De Investigaciones Científicas - Universidad Nacional De Tierra Del Fuego on behalf of 'Proyecto Argentino Interinstitucional de genomica de SARS-CoV-2' (PAIS Consortium)	Ceballos et al
EPI_ISL_1190749	CREMER(Centre de Rechercherches sur les Maladies Emergentes et Ré-émergentes)	TransVIHMI(Recherches Translationnelles sur le VIH et les Maladies Infectieuses)	Celestin Godwe et al
EPI_ISL_1190755	CREMER(Centre de Rechercherches sur les Maladies Emergentes et Ré-émergentes)	TransVIHMI(Recherches Translationnelles sur le VIH et les Maladies Infectieuses)	Celestin Godwe et al

EPI_ISL_1190756	CREMER(Centre de Rechercherches sur les Maladies Emergentes et Ré-émergentes)	TransVIHMI(Recherches Translationnelles sur le VIH et les Maladies Infectieuses)	Celestin Godwe et al
EPI_ISL_1190757	CREMER(Centre de Rechercherches sur les Maladies Emergentes et Ré-émergentes)	TransVIHMI(Recherches Translationnelles sur le VIH et les Maladies Infectieuses)	Celestin Godwe et al
EPI_ISL_1190758	CREMER(Centre de Rechercherches sur les Maladies Emergentes et Ré-émergentes)	TransVIHMI(Recherches Translationnelles sur le VIH et les Maladies Infectieuses)	Celestin Godwe et al
EPI_ISL_1190759	CREMER(Centre de Rechercherches sur les Maladies Emergentes et Ré-émergentes)	TransVIHMI(Recherches Translationnelles sur le VIH et les Maladies Infectieuses)	Celestin Godwe et al
EPI_ISL_1190760	CREMER(Centre de Rechercherches sur les Maladies Emergentes et Ré-émergentes)	TransVIHMI(Recherches Translationnelles sur le VIH et les Maladies Infectieuses)	Celestin Godwe et al
EPI_ISL_1190761	CREMER(Centre de Rechercherches sur les Maladies Emergentes et Ré-émergentes)	TransVIHMI(Recherches Translationnelles sur le VIH et les Maladies Infectieuses)	Celestin Godwe et al
EPI_ISL_1190765	CREMER(Centre de Rechercherches sur les Maladies Emergentes et Ré-émergentes)	TransVIHMI(Recherches Translationnelles sur le VIH et les Maladies Infectieuses)	Celestin Godwe et al
EPI_ISL_1190768	CREMER(Centre de Rechercherches sur les Maladies Emergentes et Ré-émergentes)	TransVIHMI(Recherches Translationnelles sur le VIH et les Maladies Infectieuses)	Celestin Godwe et al
EPI_ISL_1190752	CREMER(Centre de Rechercherches sur les Maladies Emergentes et Ré-émergentes)	TransVIHMI(Recherches Translationnelles sur le VIH et les Maladies Infectieuses)	Celestin Godwe et al
EPI_ISL_837621	Instituto Nacional de Enfermedades Respiratorias (INER)	Instituto Nacional de Enfermedades Respiratorias (INER)	Celia Boukadida et al
EPI_ISL_837694	Instituto Nacional de Enfermedades Respiratorias (INER)	Instituto Nacional de Enfermedades Respiratorias (INER)	Celia Boukadida et al
EPI_ISL_837736	Instituto Nacional de Enfermedades Respiratorias (INER)	Instituto Nacional de Enfermedades Respiratorias (INER)	Celia Boukadida et al
EPI_ISL_1620797	Plateforme de testing Namuroise	Plateforme de testing Namuroise	Céline Maschietto et al
EPI_ISL_1620773	Plateforme de testing Namuroise	Plateforme de testing Namuroise	Céline Maschietto et al
EPI_ISL_1670621	Plateforme de testing Namuroise	Plateforme de testing Namuroise	Céline Maschietto et al
EPI_ISL_451302	Laboratory of Virology, INMI Lazzaro Spallanzani IRCCS	Laboratory of Virology, INMI Lazzaro Spallanzani IRCCS	Cesare E.M. Gruber et al
EPI_ISL_1318216	Ministry of Health Hospitals	Institute of Health and Community Medicine	Chan Chia Jui et al
EPI_ISL_1318217	Ministry of Health Hospitals	Institute of Health and Community Medicine	Chan Chia Jui et al
EPI_ISL_434571	Microbiology, The University of Hong Kong	Microbiology, The University of Hong Kong	Chan et al
EPI_ISL_1184582	Genelabs Medical (Pvt) Ltd	Genelabs Medical (Pvt) Ltd	Chandanamali Punchihewa et al
EPI_ISL_862717	Centre for Dengue Research and AICBU, Department of Immunology and Molecular Medicine	Centre for Dengue Research and AICBU, Department of Immunology and Molecular Medicine	Chandima Jeewandara et al
EPI_ISL_862719	Centre for Dengue Research and AICBU, Department of Immunology and Molecular Medicine	Centre for Dengue Research and AICBU, Department of Immunology and Molecular Medicine	Chandima Jeewandara et al
EPI_ISL_668447	Centre for Dengue Research, Department of Immunology and Molecular Medicine	Centre for Dengue Research, Department of Immunology and Molecular Medicine	Chandima Jeewandara et al
EPI_ISL_668448	Centre for Dengue Research, Department of Immunology and Molecular Medicine	Centre for Dengue Research, Department of Immunology and Molecular Medicine	Chandima Jeewandara et al
EPI_ISL_668453	Centre for Dengue Research, Department of Immunology and Molecular Medicine	Centre for Dengue Research, Department of Immunology and Molecular Medicine	Chandima Jeewandara et al

EPI_ISL_668449	Centre for Dengue Research, Department of Immunology and Molecular Medicine	Centre for Dengue Research, Department of Immunology and Molecular Medicine	Chandima Jeewandara et al
EPI_ISL_668450	Centre for Dengue Research, Department of Immunology and Molecular Medicine	Centre for Dengue Research, Department of Immunology and Molecular Medicine	Chandima Jeewandara et al
EPI_ISL_668452	Centre for Dengue Research, Department of Immunology and Molecular Medicine	Centre for Dengue Research, Department of Immunology and Molecular Medicine	Chandima Jeewandara et al
EPI_ISL_978919	Centre for Dengue Research and AICBU, Department of Immunology and Molecular Medicine	Centre for Dengue Research and AICBU, Department of Immunology and Molecular Medicine	Chandima Jeewandara et al
EPI_ISL_525488	Centre for Dengue Research	Centre for Dengue Research	Chandima Jeewandara et al
EPI_ISL_525489	Centre for Dengue Research	Centre for Dengue Research	Chandima Jeewandara et al
EPI_ISL_525486	Centre for Dengue Research	Centre for Dengue Research	Chandima Jeewandara et al
EPI_ISL_525474	Centre for Dengue Research	Centre for Dengue Research, USJ, SL	Chandima Jeewandara et al
EPI_ISL_525476	Centre for Dengue Research	Centre for Dengue Research	Chandima Jeewandara et al
EPI_ISL_525478	Centre for Dengue Research	Centre for Dengue Research	Chandima Jeewandara et al
EPI_ISL_525479	Centre for Dengue Research	Centre for Dengue Research	Chandima Jeewandara et al
EPI_ISL_1533806	Centre for Dengue Research and AICBU, Department of Immunology and Molecular Medicine	Centre for Dengue Research and AICBU, Department of Immunology and Molecular Medicine	Chandima Jeewandara et al
EPI_ISL_1233112	Centre for Dengue Research and AICBU, Department of Immunology and Molecular Medicine	Centre for Dengue Research and AICBU, Department of Immunology and Molecular Medicine	Chandima Jeewandara et al
EPI_ISL_1533821	Centre for Dengue Research and AICBU, Department of Immunology and Molecular Medicine	Centre for Dengue Research and AICBU, Department of Immunology and Molecular Medicine	Chandima Jeewandara et al
EPI_ISL_602565	Centre for Dengue Research, Department of Immunology and Molecular Medicine	Centre for Dengue Research, Department of Immunology and Molecular Medicine,	Chandima Jeewandara et al
EPI_ISL_792548	Centre for Dengue Research and AICBU, Department of Immunology and Molecular Medicine	Centre for Dengue Research and AICBU, Department of Immunology and Molecular Medicine	Chandima Jeewandara et al
EPI_ISL_792549	Centre for Dengue Research and AICBU, Department of Immunology and Molecular Medicine	Centre for Dengue Research and AICBU, Department of Immunology and Molecular Medicine	Chandima Jeewandara et al
EPI_ISL_978920	Centre for Dengue Research and AICBU, Department of Immunology and Molecular Medicine	Centre for Dengue Research and AICBU, Department of Immunology and Molecular Medicine	Chandima Jeewandara et al
EPI_ISL_792550	Centre for Dengue Research and AICBU, Department of Immunology and Molecular Medicine	Centre for Dengue Research and AICBU, Department of Immunology and Molecular Medicine	Chandima Jeewandara et al
EPI_ISL_978921	Centre for Dengue Research and AICBU, Department of Immunology and Molecular Medicine	Centre for Dengue Research and AICBU, Department of Immunology and Molecular Medicine	Chandima Jeewandara et al
EPI_ISL_978923	Centre for Dengue Research and AICBU, Department of Immunology and Molecular Medicine	Centre for Dengue Research and AICBU, Department of Immunology and Molecular Medicine	Chandima Jeewandara et al
EPI_ISL_978986	Centre for Dengue Research and AICBU, Department of Immunology and Molecular Medicine	Centre for Dengue Research and AICBU, Department of Immunology and Molecular Medicine	Chandima Jeewandara et al
EPI_ISL_1233096	Centre for Dengue Research and AICBU, Department of Immunology and Molecular Medicine	Centre for Dengue Research and AICBU, Department of Immunology and Molecular Medicine	Chandima Jeewandara et al
EPI_ISL_1533823	Centre for Dengue Research and AICBU, Department of Immunology and Molecular Medicine	Centre for Dengue Research and AICBU, Department of Immunology and Molecular Medicine	Chandima Jeewandara et al

EPI_ISL_1533831	Centre for Dengue Research and AICBU, Department of Immunology and Molecular Medicine	Centre for Dengue Research and AICBU, Department of Immunology and Molecular Medicine	Chandima Jeewandara et al
EPI_ISL_1533836	Centre for Dengue Research and AICBU, Department of Immunology and Molecular Medicine	Centre for Dengue Research and AICBU, Department of Immunology and Molecular Medicine	Chandima Jeewandara et al
EPI_ISL_428670	Centre for Dengue Research	Centre for Dengue Research	Chandima Jeewandara et al
EPI_ISL_428673	Centre for Dengue Research	Centre for Dengue Research	Chandima Jeewandara et al
EPI_ISL_428672	Centre for Dengue Research	Centre for Dengue Research	Chandima Jeewandara et al
EPI_ISL_1533838	Centre for Dengue Research and AICBU, Department of Immunology and Molecular Medicine	Centre for Dengue Research and AICBU, Department of Immunology and Molecular Medicine	Chandima Jeewandara et al
EPI_ISL_996060	Centre for Dengue Research and AICBU, Department of Immunology and Molecular Medicine	Centre for Dengue Research and AICBU, Department of Immunology and Molecular Medicine	Chandima Jeewandara et al
EPI_ISL_996036	Centre for Dengue Research and AICBU, Department of Immunology and Molecular Medicine	Centre for Dengue Research and AICBU, Department of Immunology and Molecular Medicine	Chandima Jeewandara et al
EPI_ISL_1233114	Centre for Dengue Research and AICBU, Department of Immunology and Molecular Medicine	Centre for Dengue Research and AICBU, Department of Immunology and Molecular Medicine	Chandima Jeewandara et al
EPI_ISL_792552	Centre for Dengue Research and AICBU, Department of Immunology and Molecular Medicine	Centre for Dengue Research and AICBU, Department of Immunology and Molecular Medicine	Chandima Jeewandara et al
EPI_ISL_1233061	Centre for Dengue Research and AICBU, Department of Immunology and Molecular Medicine	Centre for Dengue Research and AICBU, Department of Immunology and Molecular Medicine	Chandima Jeewandara et al
EPI_ISL_1533848	Centre for Dengue Research and AICBU, Department of Immunology and Molecular Medicine	Centre for Dengue Research and AICBU, Department of Immunology and Molecular Medicine	Chandima Jeewandara et al
EPI_ISL_1582415	Centre for Dengue Research and AICBU, Department of Immunology and Molecular Medicine	Centre for Dengue Research and AICBU, Department of Immunology and Molecular Medicine	Chandima Jeewandara et al
EPI_ISL_792556	Centre for Dengue Research and AICBU, Department of Immunology and Molecular Medicine	Centre for Dengue Research and AICBU, Department of Immunology and Molecular Medicine	Chandima Jeewandara et al
EPI_ISL_602566	Centre for Dengue Research, Department of Immunology and Molecular Medicine	Centre for Dengue Research, Department of Immunology and Molecular Medicine	Chandima Jeewandara et al
EPI_ISL_668454	Centre for Dengue Research, Department of Immunology and Molecular Medicine	Centre for Dengue Research, Department of Immunology and Molecular Medicine	Chandima Jeewandara et al
EPI_ISL_1662584	Molecular diagnostic unit for viral haemorrhagic fevers and emerging viruses, Bouaké CHU Laboratory	Project group Epidemology of Highly Pathogenic Microorganisms, Robert Koch-Institute	Chantal Akoua-Koffi et al
EPI_ISL_1662591	Molecular diagnostic unit for viral haemorrhagic fevers and emerging viruses, Bouaké CHU Laboratory	Project group Epidemology of Highly Pathogenic Microorganisms, Robert Koch-Institute	Chantal Akoua-Koffi et al
EPI_ISL_1663657	Molecular diagnostic unit for viral haemorrhagic fevers and emerging viruses, Bouaké CHU Laboratory	Molecular diagnostic unit for viral haemorrhagic fevers and emerging viruses, Bouaké CHU Laboratory	Chantal Akoua-Koffi et al
EPI_ISL_1663664	Molecular diagnostic unit for viral haemorrhagic fevers and emerging viruses, Bouaké CHU Laboratory	Molecular diagnostic unit for viral haemorrhagic fevers and emerging viruses, Bouaké CHU Laboratory	Chantal Akoua-Koffi et al
EPI_ISL_1663669	Molecular diagnostic unit for viral haemorrhagic fevers and emerging viruses, Bouaké CHU Laboratory	Molecular diagnostic unit for viral haemorrhagic fevers and emerging viruses, Bouaké CHU Laboratory	Chantal Akoua-Koffi et al
EPI_ISL_1663661	Molecular diagnostic unit for viral haemorrhagic fevers and emerging viruses, Bouaké CHU Laboratory	Molecular diagnostic unit for viral haemorrhagic fevers and emerging viruses, Bouaké CHU Laboratory	Chantal Akoua-Koffi et al
EPI_ISL_1663662	Molecular diagnostic unit for viral haemorrhagic fevers and emerging viruses, Bouaké CHU Laboratory	Molecular diagnostic unit for viral haemorrhagic fevers and emerging viruses, Bouaké CHU Laboratory	Chantal Akoua-Koffi et al

EPI_ISL_1663670	Molecular diagnostic unit for viral haemorrhagic fevers and emerging viruses, Bouaké CHU Laboratory	Molecular diagnostic unit for viral haemorrhagic fevers and emerging viruses, Bouaké CHU Laboratory	Chantal Akoua-Koffi et al
EPI_ISL_1663673	Molecular diagnostic unit for viral haemorrhagic fevers and emerging viruses, Bouaké CHU Laboratory	Molecular diagnostic unit for viral haemorrhagic fevers and emerging viruses, Bouaké CHU Laboratory	Chantal Akoua-Koffi et al
EPI_ISL_1663659	Molecular diagnostic unit for viral haemorrhagic fevers and emerging viruses, Bouaké CHU Laboratory	Molecular diagnostic unit for viral haemorrhagic fevers and emerging viruses, Bouaké CHU Laboratory	Chantal Akoua-Koffi et al
EPI_ISL_1663656	Molecular diagnostic unit for viral haemorrhagic fevers and emerging viruses, Bouaké CHU Laboratory	Molecular diagnostic unit for viral haemorrhagic fevers and emerging viruses, Bouaké CHU Laboratory	Chantal Akoua-Koffi et al
EPI_ISL_1663674	Molecular diagnostic unit for viral haemorrhagic fevers and emerging viruses, Bouaké CHU Laboratory	Molecular diagnostic unit for viral haemorrhagic fevers and emerging viruses, Bouaké CHU Laboratory	Chantal Akoua-Koffi et al
EPI_ISL_1663677	Molecular diagnostic unit for viral haemorrhagic fevers and emerging viruses, Bouaké CHU Laboratory	Molecular diagnostic unit for viral haemorrhagic fevers and emerging viruses, Bouaké CHU Laboratory	Chantal Akoua-Koffi et al
EPI_ISL_1663680	Molecular diagnostic unit for viral haemorrhagic fevers and emerging viruses, Bouaké CHU Laboratory	Molecular diagnostic unit for viral haemorrhagic fevers and emerging viruses, Bouaké CHU Laboratory	Chantal Akoua-Koffi et al
EPI_ISL_1663678	Molecular diagnostic unit for viral haemorrhagic fevers and emerging viruses, Bouaké CHU Laboratory	Molecular diagnostic unit for viral haemorrhagic fevers and emerging viruses, Bouaké CHU Laboratory	Chantal Akoua-Koffi et al
EPI_ISL_1663679	Molecular diagnostic unit for viral haemorrhagic fevers and emerging viruses, Bouaké CHU Laboratory	Molecular diagnostic unit for viral haemorrhagic fevers and emerging viruses, Bouaké CHU Laboratory	Chantal Akoua-Koffi et al
EPI_ISL_1365032	Molecular diagnostic unit for viral haemorrhagic fevers and emerging viruses, Bouaké CHU Laboratory	Molecular diagnostic unit for viral haemorrhagic fevers and emerging viruses, Bouaké CHU Laboratory	Chantal Akoua-Koffi et al
EPI_ISL_1365027	Molecular diagnostic unit for viral haemorrhagic fevers and emerging viruses, Bouaké CHU Laboratory	Molecular diagnostic unit for viral haemorrhagic fevers and emerging viruses, Bouaké CHU Laboratory	Chantal Akoua-Koffi et al
EPI_ISL_614348	Molecular diagnostic unit for viral haemorrhagic fevers and emerging viruses, Bouaké CHU Laboratory	Project group Epidemology of Highly Pathogenic Microorganisms, Robert Koch-Institute	Chantal Akoua-Koffi et al
EPI_ISL_614349	Molecular diagnostic unit for viral haemorrhagic fevers and emerging viruses, Bouaké CHU Laboratory	Project group Epidemology of Highly Pathogenic Microorganisms, Robert Koch-Institute	Chantal Akoua-Koffi et al
EPI_ISL_614350	Molecular diagnostic unit for viral haemorrhagic fevers and emerging viruses, Bouaké CHU Laboratory	Project group Epidemology of Highly Pathogenic Microorganisms, Robert Koch-Institute	Chantal Akoua-Koffi et al
EPI_ISL_614363	Molecular diagnostic unit for viral haemorrhagic fevers and emerging viruses, Bouaké CHU Laboratory	Project group Epidemology of Highly Pathogenic Microorganisms, Robert Koch-Institute	Chantal Akoua-Koffi et al
EPI_ISL_614366	Molecular diagnostic unit for viral haemorrhagic fevers and emerging viruses, Bouaké CHU Laboratory	Project group Epidemology of Highly Pathogenic Microorganisms, Robert Koch-Institute	Chantal Akoua-Koffi et al
EPI_ISL_614370	Molecular diagnostic unit for viral haemorrhagic fevers and emerging viruses, Bouaké CHU Laboratory	Project group Epidemology of Highly Pathogenic Microorganisms, Robert Koch-Institute	Chantal Akoua-Koffi et al
EPI_ISL_614371	Molecular diagnostic unit for viral haemorrhagic fevers and emerging viruses, Bouaké CHU Laboratory	Project group Epidemology of Highly Pathogenic Microorganisms, Robert Koch-Institute	Chantal Akoua-Koffi et al
EPI_ISL_614373	Molecular diagnostic unit for viral haemorrhagic fevers and emerging viruses, Bouaké CHU Laboratory	Project group Epidemology of Highly Pathogenic Microorganisms, Robert Koch-Institute	Chantal Akoua-Koffi et al
EPI_ISL_614374	Molecular diagnostic unit for viral haemorrhagic fevers and emerging viruses, Bouaké CHU Laboratory	Project group Epidemology of Highly Pathogenic Microorganisms, Robert Koch-Institute	Chantal Akoua-Koffi et al
EPI_ISL_682059	Molecular diagnostic unit for viral haemorrhagic fevers and emerging viruses, Bouaké CHU Laboratory	Project group Epidemology of Highly Pathogenic Microorganisms, Robert Koch-Institute	Chantal Akoua-Koffi et al
EPI_ISL_681837	Molecular diagnostic unit for viral haemorrhagic fevers and emerging viruses, Bouaké CHU Laboratory	Project group Epidemology of Highly Pathogenic Microorganisms, Robert Koch-Institute	Chantal Akoua-Koffi et al

EPI_ISL_681839	Molecular diagnostic unit for viral haemorrhagic fevers and emerging viruses, Bouaké CHU Laboratory	Project group Epidemology of Highly Pathogenic Microorganisms, Robert Koch-Institute	Chantal Akoua-Koffi et al
EPI_ISL_614387	Molecular diagnostic unit for viral haemorrhagic fevers and emerging viruses, Bouaké CHU Laboratory	Project group Epidemology of Highly Pathogenic Microorganisms, Robert Koch-Institute	Chantal Akoua-Koffi et al
EPI_ISL_614390	Molecular diagnostic unit for viral haemorrhagic fevers and emerging viruses, Bouaké CHU Laboratory	Project group Epidemology of Highly Pathogenic Microorganisms, Robert Koch-Institute	Chantal Akoua-Koffi et al
EPI_ISL_614391	Molecular diagnostic unit for viral haemorrhagic fevers and emerging viruses, Bouaké CHU Laboratory	Project group Epidemology of Highly Pathogenic Microorganisms, Robert Koch-Institute	Chantal Akoua-Koffi et al
EPI_ISL_614392	Molecular diagnostic unit for viral haemorrhagic fevers and emerging viruses, Bouaké CHU Laboratory	Project group Epidemology of Highly Pathogenic Microorganisms, Robert Koch-Institute	Chantal Akoua-Koffi et al
EPI_ISL_1367697	Molecular diagnostic unit for viral haemorrhagic fevers and emerging viruses, Bouaké CHU Laboratory	Molecular diagnostic unit for viral haemorrhagic fevers and emerging viruses, Bouaké CHU Laboratory	Chantal Akoua-Koffi et al
EPI_ISL_1367689	Molecular diagnostic unit for viral haemorrhagic fevers and emerging viruses, Bouaké CHU Laboratory	Molecular diagnostic unit for viral haemorrhagic fevers and emerging viruses, Bouaké CHU Laboratory	Chantal Akoua-Koffi et al
EPI_ISL_1367698	Molecular diagnostic unit for viral haemorrhagic fevers and emerging viruses, Bouaké CHU Laboratory	Molecular diagnostic unit for viral haemorrhagic fevers and emerging viruses, Bouaké CHU Laboratory	Chantal Akoua-Koffi et al
EPI_ISL_1367695	Molecular diagnostic unit for viral haemorrhagic fevers and emerging viruses, Bouaké CHU Laboratory	Molecular diagnostic unit for viral haemorrhagic fevers and emerging viruses, Bouaké CHU Laboratory	Chantal Akoua-Koffi et al
EPI_ISL_682058	Molecular diagnostic unit for viral haemorrhagic fevers and emerging viruses, Bouaké CHU Laboratory	Project group Epidemology of Highly Pathogenic Microorganisms, Robert Koch-Institute	Chantal Akoua-Koffi et al
EPI_ISL_729091	Viollier AG	Department of Biosystems Science and Engineering, ETH Zürich	Chaoran Chen et al
EPI_ISL_1408304	Viollier AG	Department of Biosystems Science and Engineering, ETH Zürich	Chaoran Chen et al
EPI_ISL_1408276	Viollier AG	Department of Biosystems Science and Engineering, ETH Zürich	Chaoran Chen et al
EPI_ISL_1408279	Viollier AG	Department of Biosystems Science and Engineering, ETH Zürich	Chaoran Chen et al
EPI_ISL_1407582	Viollier AG	Department of Biosystems Science and Engineering, ETH Zürich	Chaoran Chen et al
EPI_ISL_1408265	Viollier AG	Department of Biosystems Science and Engineering, ETH Zürich	Chaoran Chen et al
EPI_ISL_1407598	Viollier AG	Department of Biosystems Science and Engineering, ETH Zürich	Chaoran Chen et al
EPI_ISL_1407551	Viollier AG	Department of Biosystems Science and Engineering, ETH Zürich	Chaoran Chen et al
EPI_ISL_1408290	Viollier AG	Department of Biosystems Science and Engineering, ETH Zürich	Chaoran Chen et al
EPI_ISL_1407619	Viollier AG	Department of Biosystems Science and Engineering, ETH Zürich	Chaoran Chen et al
EPI_ISL_1408278	Viollier AG	Department of Biosystems Science and Engineering, ETH Zürich	Chaoran Chen et al
EPI_ISL_1659173	Viollier AG	Department of Biosystems Science and Engineering, ETH Zürich	Chaoran Chen et al
EPI_ISL_648803	Department of Laboratory Medicine, Tan Tock Seng Hospital	Department of Laboratory Medicine, Tan Tock Seng Hospital	Chen YYC et al
EPI_ISL_648805	Department of Laboratory Medicine, Tan Tock Seng Hospital	Department of Laboratory Medicine, Tan Tock Seng Hospital	Chen YYC et al
EPI_ISL_648806	Department of Laboratory Medicine, Tan Tock Seng Hospital	Department of Laboratory Medicine, Tan Tock Seng Hospital	Chen YYC et al
EPI_ISL_648818	Department of Laboratory Medicine, Tan Tock Seng Hospital	Department of Laboratory Medicine, Tan Tock Seng Hospital	Chen YYC et al
EPI_ISL_648706	Department of Laboratory Medicine, Tan Tock Seng Hospital	Department of Laboratory Medicine, Tan Tock Seng Hospital	Chen YYC et al

EPI_ISL_648715	Department of Laboratory Medicine, Tan Tock Seng Hospital	Department of Laboratory Medicine, Tan Tock Seng Hospital	Chen YYC et al
EPI_ISL_648718	Department of Laboratory Medicine, Tan Tock Seng Hospital	Department of Laboratory Medicine, Tan Tock Seng Hospital	Chen YYC et al
EPI_ISL_648736	Department of Laboratory Medicine, Tan Tock Seng Hospital	Department of Laboratory Medicine, Tan Tock Seng Hospital	Chen YYC et al
EPI_ISL_507006	Department of Laboratory Medicine, Tan Tock Seng Hospital	Department of Laboratory Medicine, Tan Tock Seng Hospital	Chen YYC et al
EPI_ISL_538436	Department of Laboratory Medicine, Tan Tock Seng Hospital	Department of Laboratory Medicine, Tan Tock Seng Hospital	Chen YYC et al
EPI_ISL_538456	Department of Laboratory Medicine, Tan Tock Seng Hospital	Department of Laboratory Medicine, Tan Tock Seng Hospital	Chen YYC et al
EPI_ISL_538458	Department of Laboratory Medicine, Tan Tock Seng Hospital	Department of Laboratory Medicine, Tan Tock Seng Hospital	Chen YYC et al
EPI_ISL_426629	TSGH-CP molecular lab	TSGH-CP molecular lab	Cherng-Lih Perng et al
EPI_ISL_437336	TSGH-CP molecular lab, Division of Clinical Pathology, Department of Pathology	TSGH-CP molecular lab, Division of Clinical Pathology, Department of Pathology	Cherng-Lih Perng et al
EPI_ISL_447252	TSGH-CP molecular lab	TSGH-CP molecular lab	Cherng-Lih Perng et al
EPI_ISL_447256	TSGH-CP molecular lab	TSGH-CP molecular lab	Cherng-Lih Perng et al
EPI_ISL_447593	TSGH-CP molecular lab	TSGH-CP molecular lab	Cherng-Lih Perng et al
EPI_ISL_803911	BBMP Urban PHC	Department of Neurovirology, National Institute of Mental Health and Neurosciences (NIMHANS)	Chitra Pattabiraman et al
EPI_ISL_803903	Shimoga Institute of Medical Sciences	Department of Neurovirology, National Institute of Mental Health and Neurosciences (NIMHANS)	Chitra Pattabiraman et al
EPI_ISL_995747	Railway Hospital	Department of Neurovirology, National Institute of Mental Health and Neurosciences (NIMHANS)	Chitra Pattabiraman et al
EPI_ISL_995753	RAILWAY HOSPITAL	Department of Neurovirology, National Institute of Mental Health and Neurosciences (NIMHANS)	Chitra Pattabiraman et al
EPI_ISL_484073	Centre for Clinical Infection and Diagnostics Research and Genomics Innovation Unit, Guy's and St. Thomas' NHS Trust	COVID-19 Genomics UK (COG-UK) Consortium	Chloe Fisher et al
EPI_ISL_1360415	Child Health Research Foundation	Child Health Research Foundation	CHRF Bangladesh Genomics Team et al
EPI_ISL_1360430	Child Health Research Foundation	Child Health Research Foundation	CHRF Bangladesh Genomics Team et al
EPI_ISL_1360437	Shimantik Pathology and Diagnostic Center	Child Health Research Foundation	CHRF Bangladesh Genomics Team et al
EPI_ISL_1360439	Shimantik Pathology and Diagnostic Center	Child Health Research Foundation	CHRF Bangladesh Genomics Team et al
EPI_ISL_1360445	Shimantik Pathology and Diagnostic Center	Child Health Research Foundation	CHRF Bangladesh Genomics Team et al
EPI_ISL_1360451	Child Health Research Foundation	Child Health Research Foundation	CHRF Bangladesh Genomics Team et al
EPI_ISL_1469973	Child Health Research Foundation	Child Health Research Foundation	CHRF Bangladesh Genomics Team et al
EPI_ISL_1469978	Child Health Research Foundation	Child Health Research Foundation	CHRF Bangladesh Genomics Team et al
EPI_ISL_1469982	Child Health Research Foundation	Child Health Research Foundation	CHRF Bangladesh Genomics Team et al
EPI_ISL_1634449	Child Health Research Foundation	Child Health Research Foundation	CHRF Bangladesh Genomics Team et al
EPI_ISL_486462	Viollier AG	Department of Biosystems Science and Engineering, ETH Zürich	Christian Beisel et al

EPI_ISL_1129407	Viollier AG	Department of Biosystems Science and Engineering, ETH Zürich	Christian Beisel et al
EPI_ISL_468285	Viollier AG	Department of Biosystems Science and Engineering, ETH Zürich	Christian Beisel et al
EPI_ISL_523870	Viollier AG	Department of Biosystems Science and Engineering, ETH Zürich	Christian Beisel et al
EPI_ISL_1658258	Viollier AG	Department of Biosystems Science and Engineering, ETH Zurich	Christian Beisel et al
EPI_ISL_574887	Viollier AG	Department of Biosystems Science and Engineering, ETH Zürich	Christian Beisel et al
EPI_ISL_468290	Viollier AG	Department of Biosystems Science and Engineering, ETH Zürich	Christian Beisel et al
EPI_ISL_476094	Viollier AG	Department of Biosystems Science and Engineering, ETH Zürich	Christian Beisel et al
EPI_ISL_486521	Viollier AG	Department of Biosystems Science and Engineering, ETH Zürich	Christian Beisel et al
EPI_ISL_486528	Viollier AG	Department of Biosystems Science and Engineering, ETH Zürich	Christian Beisel et al
EPI_ISL_1406815	Viollier AG	Department of Biosystems Science and Engineering, ETH Zürich	Christian Beisel et al
EPI_ISL_560458	Viollier AG	Department of Biosystems Science and Engineering, ETH Zürich	Christian Beisel et al
EPI_ISL_1496225	Viollier AG	Department of Biosystems Science and Engineering, ETH Zürich	Christian Beisel et al
EPI_ISL_451774	Viollier AG	Department of Biosystems Science and Engineering, ETH Zürich	Christian Beisel et al
EPI_ISL_467001	Viollier AG	Department of Biosystems Science and Engineering, ETH Zürich	Christian Beisel et al
EPI_ISL_1130121	Viollier AG	Department of Biosystems Science and Engineering, ETH Zürich	Christian Beisel et al
EPI_ISL_468269	Viollier AG	Department of Biosystems Science and Engineering, ETH Zürich	Christian Beisel et al
EPI_ISL_1130149	Viollier AG	Department of Biosystems Science and Engineering, ETH Zürich	Christian Beisel et al
EPI_ISL_486506	Viollier AG	Department of Biosystems Science and Engineering, ETH Zürich	Christian Beisel et al
EPI_ISL_468295	Viollier AG	Department of Biosystems Science and Engineering, ETH Zürich	Christian Beisel et al
EPI_ISL_1130695	Viollier AG	Department of Biosystems Science and Engineering, ETH Zürich	Christian Beisel et al
EPI_ISL_1130704	Viollier AG	Department of Biosystems Science and Engineering, ETH Zürich	Christian Beisel et al
EPI_ISL_539418	Viollier AG	Department of Biosystems Science and Engineering, ETH Zürich	Christian Beisel et al
EPI_ISL_693858	Viollier AG	Department of Biosystems Science and Engineering, ETH Zürich	Christian Beisel et al
EPI_ISL_468281	Viollier AG	Department of Biosystems Science and Engineering, ETH Zürich	Christian Beisel et al
EPI_ISL_523920	Viollier AG	Department of Biosystems Science and Engineering, ETH Zürich	Christian Beisel et al
EPI_ISL_541509	Viollier AG	Department of Biosystems Science and Engineering, ETH Zürich	Christian Beisel et al
EPI_ISL_451726	Viollier AG	Department of Biosystems Science and Engineering, ETH Zürich	Christian Beisel et al
EPI_ISL_483659	Viollier AG	Department of Biosystems Science and Engineering, ETH Zürich	Christian Beisel et al
EPI_ISL_523913	Viollier AG	Department of Biosystems Science and Engineering, ETH Zürich	Christian Beisel et al
EPI_ISL_541512	Viollier AG	Department of Biosystems Science and Engineering, ETH Zürich	Christian Beisel et al

EPI_ISL_1260530	Viollier AG	Department of Biosystems Science and Engineering, ETH Zürich	Christian Beisel et al
EPI_ISL_1260508	Viollier AG	Department of Biosystems Science and Engineering, ETH Zürich	Christian Beisel et al
EPI_ISL_1361446	Viollier AG	Department of Biosystems Science and Engineering, ETH Zürich	Christian Beisel et al
EPI_ISL_508862	Virology Unit, Institut Pasteur de Madagascar	Virology Unit, Institut Pasteur de Madagascar	Christian Ranaivoson et al
EPI_ISL_508863	Virology Unit, Institut Pasteur de Madagascar	Virology Unit, Institut Pasteur de Madagascar	Christian Ranaivoson et al
EPI_ISL_1660239	Virology Unit, Institut Pasteur de Madagascar	Virology Unit, Institut Pasteur de Madagascar	Christian Ranaivoson et al
EPI_ISL_1660240	Virology Unit, Institut Pasteur de Madagascar	Virology Unit, Institut Pasteur de Madagascar	Christian Ranaivoson et al
EPI_ISL_1660263	Virology Unit, Institut Pasteur de Madagascar	Virology Unit, Institut Pasteur de Madagascar	Christian Ranaivoson et al
EPI_ISL_1660272	Virology Unit, Institut Pasteur de Madagascar	Virology Unit, Institut Pasteur de Madagascar	Christian Ranaivoson et al
EPI_ISL_1660273	Virology Unit, Institut Pasteur de Madagascar	Virology Unit, Institut Pasteur de Madagascar	Christian Ranaivoson et al
EPI_ISL_1660274	Virology Unit, Institut Pasteur de Madagascar	Virology Unit, Institut Pasteur de Madagascar	Christian Ranaivoson et al
EPI_ISL_1660279	Virology Unit, Institut Pasteur de Madagascar	Virology Unit, Institut Pasteur de Madagascar	Christian Ranaivoson et al
EPI_ISL_1660286	Virology Unit, Institut Pasteur de Madagascar	Virology Unit, Institut Pasteur de Madagascar	Christian Ranaivoson et al
EPI_ISL_1660288	Virology Unit, Institut Pasteur de Madagascar	Virology Unit, Institut Pasteur de Madagascar	Christian Ranaivoson et al
EPI_ISL_1660289	Virology Unit, Institut Pasteur de Madagascar	Virology Unit, Institut Pasteur de Madagascar	Christian Ranaivoson et al
EPI_ISL_1660290	Virology Unit, Institut Pasteur de Madagascar	Virology Unit, Institut Pasteur de Madagascar	Christian Ranaivoson et al
EPI_ISL_1660291	Virology Unit, Institut Pasteur de Madagascar	Virology Unit, Institut Pasteur de Madagascar	Christian Ranaivoson et al
EPI_ISL_1660225	Virology Unit, Institut Pasteur de Madagascar	Virology Unit, Institut Pasteur de Madagascar	Christian Ranaivoson et al
EPI_ISL_1660226	Virology Unit, Institut Pasteur de Madagascar	Virology Unit, Institut Pasteur de Madagascar	Christian Ranaivoson et al
EPI_ISL_1660227	Virology Unit, Institut Pasteur de Madagascar	Virology Unit, Institut Pasteur de Madagascar	Christian Ranaivoson et al
EPI_ISL_1660234	Virology Unit, Institut Pasteur de Madagascar	Virology Unit, Institut Pasteur de Madagascar	Christian Ranaivoson et al
EPI_ISL_677635	Virology Unit, Institut Pasteur de Madagascar	Virology Unit, Institut Pasteur de Madagascar	Christian Ranaivoson et al
EPI_ISL_677636	Virology Unit, Institut Pasteur de Madagascar	Virology Unit, Institut Pasteur de Madagascar	Christian Ranaivoson et al
EPI_ISL_677634	Virology Unit, Institut Pasteur de Madagascar	Virology Unit, Institut Pasteur de Madagascar	Christian Ranaivoson et al
EPI_ISL_625456	Virology Unit, Institut Pasteur de Madagascar	Virology Unit, Institut Pasteur de Madagascar	Christian Ranaivoson et al
EPI_ISL_1404498	Hôpital Henri Mondor	Department of Virology, Henri Mondor University Hospital, Assistance Publique Hôpitaux de Paris, Université Paris-Est Créteil, INSERM U955	Christophe Rodriguez et al
EPI_ISL_1404538	Hôpital Henri Mondor	Department of Virology, Henri Mondor University Hospital, Assistance Publique Hôpitaux de Paris, Université Paris-Est Créteil, INSERM U955	Christophe Rodriguez et al
EPI_ISL_451957	Molecular Pathology Division, Department of Pathology, Hong Kong Sanatorium & Hospital	Molecular Pathology Division, Department of Pathology, Hong Kong Sanatorium & Hospital	Chun Hang AU et al
EPI_ISL_578701	Molecular Pathology Division, Department of Pathology, Hong Kong Sanatorium & Hospital	Molecular Pathology Division, Department of Pathology, Hong Kong Sanatorium & Hospital	Chun Hang AU et al

EPI_ISL_578702	Molecular Pathology Division, Department of Pathology, Hong Kong Sanatorium & Hospital	Molecular Pathology Division, Department of Pathology, Hong Kong Sanatorium & Hospital	Chun Hang AU et al
EPI_ISL_593698	South Eastern Area Laboratory Services (SEALS)	NSW Health Pathology - Institute of Clinical Pathology and Medical Research; Westmead Hospital; University of Sydney	CIDM-PH et al.
EPI_ISL_593737	South Eastern Area Laboratory Services (SEALS)	NSW Health Pathology - Institute of Clinical Pathology and Medical Research; Westmead Hospital; University of Sydney	CIDM-PH et al.
EPI_ISL_593739	South Eastern Area Laboratory Services (SEALS)	NSW Health Pathology - Institute of Clinical Pathology and Medical Research; Westmead Hospital; University of Sydney	CIDM-PH et al.
EPI_ISL_629004	South Eastern Area Laboratory Services (SEALS)	NSW Health Pathology - Institute of Clinical Pathology and Medical Research; Westmead Hospital; University of Sydney	CIDM-PH et al.
EPI_ISL_667800	South Eastern Area Laboratory Services (SEALS)	NSW Health Pathology - Institute of Clinical Pathology and Medical Research; Westmead Hospital; University of Sydney	CIDM-PH et al.
EPI_ISL_767864	Sydney South West Pathology Service (SSWPS) - Royal Prince Alfred Hospital - NSW Health Pathology	NSW Health Pathology - Institute of Clinical Pathology and Medical Research; Westmead Hospital; University of Sydney	CIDM-PH et al.
EPI_ISL_767865	Sydney South West Pathology Service (SSWPS) - Royal Prince Alfred Hospital - NSW Health Pathology	NSW Health Pathology - Institute of Clinical Pathology and Medical Research; Westmead Hospital; University of Sydney	CIDM-PH et al.
EPI_ISL_1098652	South Eastern Area Laboratory Services (SEALS)	NSW Health Pathology - Institute of Clinical Pathology and Medical Research; Westmead Hospital; University of Sydney	CIDM-PH et al.
EPI_ISL_451558	Medlab Pathology	NSW Health Pathology - Institute of Clinical Pathology and Medical Research; Westmead Hospital; University of Sydney	CIDM-PH et al.
EPI_ISL_451594	Childrens Hospital Westmead	NSW Health Pathology - Institute of Clinical Pathology and Medical Research; Westmead Hospital; University of Sydney	CIDM-PH et al.
EPI_ISL_478718	Sydney South West Pathology Service (SSWPS) - Concord Repatriation General Hospital - NSW Health Pathology	NSW Health Pathology - Institute of Clinical Pathology and Medical Research; Westmead Hospital; University of Sydney	CIDM-PH et al.
EPI_ISL_513316	South Eastern Area Laboratory Services (SEALS)	NSW Health Pathology - Institute of Clinical Pathology and Medical Research; Westmead Hospital; University of Sydney	CIDM-PH et al.
EPI_ISL_542246	ASST GOM Niguarda	Dep. Of Oncology and Hemato-Oncology University of Milan	Claudia Alteri et al
EPI_ISL_1424051	LESP Ciudad de Mexico	Instituto de Diagnostico y Referencia Epidemiologicos (INDRE)	Claudia Wong-Arambula et al
EPI_ISL_1626820	LESP Guanajuato	Instituto de Diagnostico y Referencia Epidemiologicos (INDRE)	Claudia Wong-Arambula et al
EPI_ISL_1093146	Laboratorio de Diagnostico de Enfermedades Emergentes y Reemergentes	Instituto de Diagnostico y Referencia Epidemiologicos (INDRE)	Claudia Wong-Arambula et al
EPI_ISL_1093147	Laboratorio de Diagnostico de Enfermedades Emergentes y Reemergentes	Instituto de Diagnostico y Referencia Epidemiologicos (INDRE)	Claudia Wong-Arambula et al
EPI_ISL_1516762	LESP Jalisco/UdeG	Instituto de Diagnostico y Referencia Epidemiologicos (INDRE)	Claudia Wong-Arambula et al
EPI_ISL_913968	Instituto de Diagnostico y Referencia Epidemiologicos INDRE_RNLSP	Instituto de Diagnostico y Referencia Epidemiologicos (INDRE)	Claudia Wong-Arambula et al
EPI_ISL_1168466	Instituto de Diagnostico y Referencia Epidemiologicos INDRE_RNLSP	Instituto de Diagnostico y Referencia Epidemiologicos (INDRE)	Claudia Wong-Arambula et al
EPI_ISL_1324767	LESP Quintana Roo	Instituto de Diagnostico y Referencia Epidemiologicos (INDRE)	Claudia Wong-Arambula et al
EPI_ISL_1626821	LESP Quintana Roo	Instituto de Diagnostico y Referencia Epidemiologicos (INDRE)	Claudia Wong-Arambula et al
EPI_ISL_913952	Instituto de Diagnostico y Referencia Epidemiologicos INDRE_RNLSP	Instituto de Diagnostico y Referencia Epidemiologicos (INDRE)	Claudia Wong-Arambula et al
EPI_ISL_913966	Instituto de Diagnostico y Referencia Epidemiologicos INDRE_RNLSP	Instituto de Diagnostico y Referencia Epidemiologicos (INDRE)	Claudia Wong-Arambula et al

EPI_ISL_913944	Instituto de Diagnostico y Referencia Epidemiologicos INDRE_RNLSP	Instituto de Diagnostico y Referencia Epidemiologicos (INDRE)	Claudia Wong-Arambula et al
EPI_ISL_940617	LACEN-PI DR. Costa Alvarenga	Instituto Adolfo Lutz, Interdiciplinary Procedures Center, Strategic Laboratory	Claudio Tavares Sacchi et al
EPI_ISL_882663	LACEN do Distrito Federal	Instituto Adolfo Lutz, Interdiciplinary Procedures Center, Strategic Laboratory	Claudio Tavares Sacchi et al
EPI_ISL_882664	LACEN do Distrito Federal	Instituto Adolfo Lutz, Interdiciplinary Procedures Center, Strategic Laboratory	Claudio Tavares Sacchi et al
EPI_ISL_882657	LACEN do Estado do Piaui, Dr. Costa Alvarenga	Instituto Adolfo Lutz, Interdiciplinary Procedures Center, Strategic Laboratory	Claudio Tavares Sacchi et al
EPI_ISL_1293051	LACEN do Distrito Federal	Instituto Adolfo Lutz, Interdiciplinary Procedures Center, Strategic Laboratory	Claudio Tavares Sacchi et al
EPI_ISL_1196285	LACEN do Distrito Federal	Instituto Adolfo Lutz, Interdiciplinary Procedures Center, Strategic Laboratory	Claudio Tavares Sacchi et al
EPI_ISL_1196286	LACEN do Distrito Federal	Instituto Adolfo Lutz, Interdiciplinary Procedures Center, Strategic Laboratory	Claudio Tavares Sacchi et al
EPI_ISL_1196287	LACEN do Distrito Federal	Instituto Adolfo Lutz, Interdiciplinary Procedures Center, Strategic Laboratory	Claudio Tavares Sacchi et al
EPI_ISL_1196288	LACEN do Distrito Federal	Instituto Adolfo Lutz, Interdiciplinary Procedures Center, Strategic Laboratory	Claudio Tavares Sacchi et al
EPI_ISL_1196289	LACEN do Distrito Federal	Instituto Adolfo Lutz, Interdiciplinary Procedures Center, Strategic Laboratory	Claudio Tavares Sacchi et al
EPI_ISL_1196290	LACEN do Distrito Federal	Instituto Adolfo Lutz, Interdiciplinary Procedures Center, Strategic Laboratory	Claudio Tavares Sacchi et al
EPI_ISL_1196291	LACEN do Distrito Federal	Instituto Adolfo Lutz, Interdiciplinary Procedures Center, Strategic Laboratory	Claudio Tavares Sacchi et al
EPI_ISL_1196292	LACEN do Distrito Federal	Instituto Adolfo Lutz, Interdiciplinary Procedures Center, Strategic Laboratory	Claudio Tavares Sacchi et al
EPI_ISL_1196293	LACEN do Distrito Federal	Instituto Adolfo Lutz, Interdiciplinary Procedures Center, Strategic Laboratory	Claudio Tavares Sacchi et al
EPI_ISL_1196294	LACEN do Distrito Federal	Instituto Adolfo Lutz, Interdiciplinary Procedures Center, Strategic Laboratory	Claudio Tavares Sacchi et al
EPI_ISL_1303506	LACEN do Distrito Federal	Instituto Adolfo Lutz, Interdiciplinary Procedures Center, Strategic Laboratory	Claudio Tavares Sacchi et al
EPI_ISL_1303507	LACEN do Distrito Federal	Instituto Adolfo Lutz, Interdiciplinary Procedures Center, Strategic Laboratory	Claudio Tavares Sacchi et al
EPI_ISL_1303508	LACEN do Distrito Federal	Instituto Adolfo Lutz, Interdiciplinary Procedures Center, Strategic Laboratory	Claudio Tavares Sacchi et al
EPI_ISL_882661	Hospital de Santa Barbara de Goias	Instituto Adolfo Lutz, Interdiciplinary Procedures Center, Strategic Laboratory	Claudio Tavares Sacchi et al
EPI_ISL_882662	Hospital de Santa Barbara de Goias	Instituto Adolfo Lutz, Interdiciplinary Procedures Center, Strategic Laboratory	Claudio Tavares Sacchi et al
EPI_ISL_940612	Hospital de Campanha para Enfrentamento do Coronavirus - Goiania	Instituto Adolfo Lutz, Interdiciplinary Procedures Center, Strategic Laboratory	Claudio Tavares Sacchi et al

EPI_ISL_943988	LACEN do Estado de Goias	Instituto Adolfo Lutz, Interdiciplinary Procedures Center, Strategic Laboratory	Claudio Tavares Sacchi et al
EPI_ISL_943989	LACEN do Estado de Goias	Instituto Adolfo Lutz, Interdiciplinary Procedures Center, Strategic Laboratory	Claudio Tavares Sacchi et al
EPI_ISL_1039691	LACEN do Estado de Goias	Instituto Adolfo Lutz, Interdiciplinary Procedures Center, Strategic Laboratory	Claudio Tavares Sacchi et al
EPI_ISL_1039692	LACEN do Estado de Goias	Instituto Adolfo Lutz, Interdiciplinary Procedures Center, Strategic Laboratory	Claudio Tavares Sacchi et al
EPI_ISL_1039693	LACEN do Estado de Goias	Instituto Adolfo Lutz, Interdiciplinary Procedures Center, Strategic Laboratory	Claudio Tavares Sacchi et al
EPI_ISL_1039694	LACEN do Estado de Goias	Instituto Adolfo Lutz, Interdiciplinary Procedures Center, Strategic Laboratory	Claudio Tavares Sacchi et al
EPI_ISL_1039695	LACEN do Estado de Goias	Instituto Adolfo Lutz, Interdiciplinary Procedures Center, Strategic Laboratory	Claudio Tavares Sacchi et al
EPI_ISL_1041509	LACEN do Estado de Goias	Instituto Adolfo Lutz, Interdiciplinary Procedures Center, Strategic Laboratory	Claudio Tavares Sacchi et al
EPI_ISL_1303510	LACEN do Estado de Goias	Instituto Adolfo Lutz, Interdiciplinary Procedures Center, Strategic Laboratory	Claudio Tavares Sacchi et al
EPI_ISL_1303511	LACEN do Estado de Goias	Instituto Adolfo Lutz, Interdiciplinary Procedures Center, Strategic Laboratory	Claudio Tavares Sacchi et al
EPI_ISL_1303512	LACEN do Estado de Goias	Instituto Adolfo Lutz, Interdiciplinary Procedures Center, Strategic Laboratory	Claudio Tavares Sacchi et al
EPI_ISL_1303513	LACEN do Estado de Goias	Instituto Adolfo Lutz, Interdiciplinary Procedures Center, Strategic Laboratory	Claudio Tavares Sacchi et al
EPI_ISL_1303514	LACEN do Estado de Goias	Instituto Adolfo Lutz, Interdiciplinary Procedures Center, Strategic Laboratory	Claudio Tavares Sacchi et al
EPI_ISL_1303515	LACEN do Estado de Goias	Instituto Adolfo Lutz, Interdiciplinary Procedures Center, Strategic Laboratory	Claudio Tavares Sacchi et al
EPI_ISL_1303516	LACEN do Estado de Goias	Instituto Adolfo Lutz, Interdiciplinary Procedures Center, Strategic Laboratory	Claudio Tavares Sacchi et al
EPI_ISL_1303517	LACEN do Estado de Goias	Instituto Adolfo Lutz, Interdiciplinary Procedures Center, Strategic Laboratory	Claudio Tavares Sacchi et al
EPI_ISL_1201896	Diagnosticos da America - DASA	Instituto Adolfo Lutz, Interdiciplinary Procedures Center, Strategic Laboratory	Claudio Tavares Sacchi et al
EPI_ISL_1468431	LACEN do Estado de Goias	Instituto Adolfo Lutz, Interdiciplinary Procedures Center, Strategic Laboratory	Claudio Tavares Sacchi et al
EPI_ISL_1468413	LACEN do Estado de Goias	Instituto Adolfo Lutz, Interdiciplinary Procedures Center, Strategic Laboratory	Claudio Tavares Sacchi et al
EPI_ISL_1468414	LACEN do Estado de Goias	Instituto Adolfo Lutz, Interdiciplinary Procedures Center, Strategic Laboratory	Claudio Tavares Sacchi et al
EPI_ISL_1468415	LACEN do Estado de Goias	Instituto Adolfo Lutz, Interdiciplinary Procedures Center, Strategic Laboratory	Claudio Tavares Sacchi et al
EPI_ISL_1628363	LACEN do Estado de Goias	Instituto Adolfo Lutz, Interdiciplinary Procedures Center, Strategic Laboratory	Claudio Tavares Sacchi et al

EPI_ISL_1628364	LACEN do Estado de Goias	Instituto Adolfo Lutz, Interdiciplinary Procedures Center, Strategic Laboratory	Claudio Tavares Sacchi et al
EPI_ISL_1628365	LACEN do Estado de Goias	Instituto Adolfo Lutz, Interdiciplinary Procedures Center, Strategic Laboratory	Claudio Tavares Sacchi et al
EPI_ISL_1493577	LACEN do Estado de Goias	Instituto Adolfo Lutz, Interdiciplinary Procedures Center, Strategic Laboratory	Claudio Tavares Sacchi et al
EPI_ISL_1493576	LACEN do Estado de Goias	Instituto Adolfo Lutz, Interdiciplinary Procedures Center, Strategic Laboratory	Claudio Tavares Sacchi et al
EPI_ISL_1493575	LACEN do Estado de Goias	Instituto Adolfo Lutz, Interdiciplinary Procedures Center, Strategic Laboratory	Claudio Tavares Sacchi et al
EPI_ISL_1493574	LACEN do Estado de Goias	Instituto Adolfo Lutz, Interdiciplinary Procedures Center, Strategic Laboratory	Claudio Tavares Sacchi et al
EPI_ISL_1493573	LACEN do Estado de Goias	Instituto Adolfo Lutz, Interdiciplinary Procedures Center, Strategic Laboratory	Claudio Tavares Sacchi et al
EPI_ISL_1040825	LACEN do Mato Grosso do Sul	Instituto Adolfo Lutz, Interdiciplinary Procedures Center, Strategic Laboratory	Claudio Tavares Sacchi et al
EPI_ISL_1040826	LACEN do Mato Grosso do Sul	Instituto Adolfo Lutz, Interdiciplinary Procedures Center, Strategic Laboratory	Claudio Tavares Sacchi et al
EPI_ISL_1171621	LACEN do Mato Grosso do Sul	Instituto Adolfo Lutz, Interdiciplinary Procedures Center, Strategic Laboratory	Claudio Tavares Sacchi et al
EPI_ISL_1040832	LACEN do Mato Grosso do Sul	Instituto Adolfo Lutz, Interdiciplinary Procedures Center, Strategic Laboratory	Claudio Tavares Sacchi et al
EPI_ISL_1040830	LACEN do Mato Grosso do Sul	Instituto Adolfo Lutz, Interdiciplinary Procedures Center, Strategic Laboratory	Claudio Tavares Sacchi et al
EPI_ISL_1040831	LACEN do Mato Grosso do Sul	Instituto Adolfo Lutz, Interdiciplinary Procedures Center, Strategic Laboratory	Claudio Tavares Sacchi et al
EPI_ISL_1040827	LACEN do Mato Grosso do Sul	Instituto Adolfo Lutz, Interdiciplinary Procedures Center, Strategic Laboratory	Claudio Tavares Sacchi et al
EPI_ISL_1040828	LACEN do Mato Grosso do Sul	Instituto Adolfo Lutz, Interdiciplinary Procedures Center, Strategic Laboratory	Claudio Tavares Sacchi et al
EPI_ISL_1040829	LACEN do Mato Grosso do Sul	Instituto Adolfo Lutz, Interdiciplinary Procedures Center, Strategic Laboratory	Claudio Tavares Sacchi et al
EPI_ISL_1040824	LACEN do Mato Grosso do Sul	Instituto Adolfo Lutz, Interdiciplinary Procedures Center, Strategic Laboratory	Claudio Tavares Sacchi et al
EPI_ISL_1040833	LACEN do Mato Grosso do Sul	Instituto Adolfo Lutz, Interdiciplinary Procedures Center, Strategic Laboratory	Claudio Tavares Sacchi et al
EPI_ISL_1040834	LACEN do Mato Grosso do Sul	Instituto Adolfo Lutz, Interdiciplinary Procedures Center, Strategic Laboratory	Claudio Tavares Sacchi et al
EPI_ISL_1040835	LACEN do Mato Grosso do Sul	Instituto Adolfo Lutz, Interdiciplinary Procedures Center, Strategic Laboratory	Claudio Tavares Sacchi et al
EPI_ISL_1040836	LACEN do Mato Grosso do Sul	Instituto Adolfo Lutz, Interdiciplinary Procedures Center, Strategic Laboratory	Claudio Tavares Sacchi et al
EPI_ISL_1040837	LACEN do Mato Grosso do Sul	Instituto Adolfo Lutz, Interdiciplinary Procedures Center, Strategic Laboratory	Claudio Tavares Sacchi et al

EPI_ISL_1040838	LACEN do Mato Grosso do Sul	Instituto Adolfo Lutz, Interdiciplinary Procedures Center, Strategic Laboratory	Claudio Tavares Sacchi et al
EPI_ISL_1040839	LACEN do Mato Grosso do Sul	Instituto Adolfo Lutz, Interdiciplinary Procedures Center, Strategic Laboratory	Claudio Tavares Sacchi et al
EPI_ISL_1040840	LACEN do Mato Grosso do Sul	Instituto Adolfo Lutz, Interdiciplinary Procedures Center, Strategic Laboratory	Claudio Tavares Sacchi et al
EPI_ISL_1040842	LACEN do Mato Grosso do Sul	Instituto Adolfo Lutz, Interdiciplinary Procedures Center, Strategic Laboratory	Claudio Tavares Sacchi et al
EPI_ISL_1040841	LACEN do Mato Grosso do Sul	Instituto Adolfo Lutz, Interdiciplinary Procedures Center, Strategic Laboratory	Claudio Tavares Sacchi et al
EPI_ISL_1040843	LACEN do Mato Grosso do Sul	Instituto Adolfo Lutz, Interdiciplinary Procedures Center, Strategic Laboratory	Claudio Tavares Sacchi et al
EPI_ISL_1121327	LACEN do Mato Grosso do Sul	Instituto Adolfo Lutz, Interdiciplinary Procedures Center, Strategic Laboratory	Claudio Tavares Sacchi et al
EPI_ISL_1040847	LACEN do Mato Grosso do Sul	Instituto Adolfo Lutz, Interdiciplinary Procedures Center, Strategic Laboratory	Claudio Tavares Sacchi et al
EPI_ISL_1040848	LACEN do Mato Grosso do Sul	Instituto Adolfo Lutz, Interdiciplinary Procedures Center, Strategic Laboratory	Claudio Tavares Sacchi et al
EPI_ISL_1040846	LACEN do Mato Grosso do Sul	Instituto Adolfo Lutz, Interdiciplinary Procedures Center, Strategic Laboratory	Claudio Tavares Sacchi et al
EPI_ISL_1040844	LACEN do Mato Grosso do Sul	Instituto Adolfo Lutz, Interdiciplinary Procedures Center, Strategic Laboratory	Claudio Tavares Sacchi et al
EPI_ISL_1040845	LACEN do Mato Grosso do Sul	Instituto Adolfo Lutz, Interdiciplinary Procedures Center, Strategic Laboratory	Claudio Tavares Sacchi et al
EPI_ISL_1040850	LACEN do Mato Grosso do Sul	Instituto Adolfo Lutz, Interdiciplinary Procedures Center, Strategic Laboratory	Claudio Tavares Sacchi et al
EPI_ISL_1040851	LACEN do Mato Grosso do Sul	Instituto Adolfo Lutz, Interdiciplinary Procedures Center, Strategic Laboratory	Claudio Tavares Sacchi et al
EPI_ISL_1040849	LACEN do Mato Grosso do Sul	Instituto Adolfo Lutz, Interdiciplinary Procedures Center, Strategic Laboratory	Claudio Tavares Sacchi et al
EPI_ISL_1121328	LACEN do Mato Grosso do Sul	Instituto Adolfo Lutz, Interdiciplinary Procedures Center, Strategic Laboratory	Claudio Tavares Sacchi et al
EPI_ISL_1121329	LACEN do Mato Grosso do Sul	Instituto Adolfo Lutz, Interdiciplinary Procedures Center, Strategic Laboratory	Claudio Tavares Sacchi et al
EPI_ISL_1121330	LACEN do Mato Grosso do Sul	Instituto Adolfo Lutz, Interdiciplinary Procedures Center, Strategic Laboratory	Claudio Tavares Sacchi et al
EPI_ISL_1196282	LACEN do Mato Grosso do Sul	Instituto Adolfo Lutz, Interdiciplinary Procedures Center, Strategic Laboratory	Claudio Tavares Sacchi et al
EPI_ISL_1196283	LACEN do Mato Grosso do Sul	Instituto Adolfo Lutz, Interdiciplinary Procedures Center, Strategic Laboratory	Claudio Tavares Sacchi et al
EPI_ISL_1196284	LACEN do Mato Grosso do Sul	Instituto Adolfo Lutz, Interdiciplinary Procedures Center, Strategic Laboratory	Claudio Tavares Sacchi et al
EPI_ISL_1139052	LACEN do Mato Grosso do Sul	Instituto Adolfo Lutz, Interdiciplinary Procedures Center, Strategic Laboratory	Claudio Tavares Sacchi et al

EPI_ISL_1139053	LACEN do Mato Grosso do Sul	Instituto Adolfo Lutz, Interdiciplinary Procedures Center, Strategic Laboratory	Claudio Tavares Sacchi et al
EPI_ISL_1139054	LACEN do Mato Grosso do Sul	Instituto Adolfo Lutz, Interdiciplinary Procedures Center, Strategic Laboratory	Claudio Tavares Sacchi et al
EPI_ISL_1139055	LACEN do Mato Grosso do Sul	Instituto Adolfo Lutz, Interdiciplinary Procedures Center, Strategic Laboratory	Claudio Tavares Sacchi et al
EPI_ISL_1139056	LACEN do Mato Grosso do Sul	Instituto Adolfo Lutz, Interdiciplinary Procedures Center, Strategic Laboratory	Claudio Tavares Sacchi et al
EPI_ISL_1139057	LACEN do Mato Grosso do Sul	Instituto Adolfo Lutz, Interdiciplinary Procedures Center, Strategic Laboratory	Claudio Tavares Sacchi et al
EPI_ISL_1139058	LACEN do Mato Grosso do Sul	Instituto Adolfo Lutz, Interdiciplinary Procedures Center, Strategic Laboratory	Claudio Tavares Sacchi et al
EPI_ISL_1139059	LACEN do Mato Grosso do Sul	Instituto Adolfo Lutz, Interdiciplinary Procedures Center, Strategic Laboratory	Claudio Tavares Sacchi et al
EPI_ISL_1139060	LACEN do Mato Grosso do Sul	Instituto Adolfo Lutz, Interdiciplinary Procedures Center, Strategic Laboratory	Claudio Tavares Sacchi et al
EPI_ISL_1139061	LACEN do Mato Grosso do Sul	Instituto Adolfo Lutz, Interdiciplinary Procedures Center, Strategic Laboratory	Claudio Tavares Sacchi et al
EPI_ISL_1139062	LACEN do Mato Grosso do Sul	Instituto Adolfo Lutz, Interdiciplinary Procedures Center, Strategic Laboratory	Claudio Tavares Sacchi et al
EPI_ISL_1139063	LACEN do Mato Grosso do Sul	Instituto Adolfo Lutz, Interdiciplinary Procedures Center, Strategic Laboratory	Claudio Tavares Sacchi et al
EPI_ISL_1139064	LACEN do Mato Grosso do Sul	Instituto Adolfo Lutz, Interdiciplinary Procedures Center, Strategic Laboratory	Claudio Tavares Sacchi et al
EPI_ISL_1139065	LACEN do Mato Grosso do Sul	Instituto Adolfo Lutz, Interdiciplinary Procedures Center, Strategic Laboratory	Claudio Tavares Sacchi et al
EPI_ISL_1139066	LACEN do Mato Grosso do Sul	Instituto Adolfo Lutz, Interdiciplinary Procedures Center, Strategic Laboratory	Claudio Tavares Sacchi et al
EPI_ISL_1139067	LACEN do Mato Grosso do Sul	Instituto Adolfo Lutz, Interdiciplinary Procedures Center, Strategic Laboratory	Claudio Tavares Sacchi et al
EPI_ISL_1139068	LACEN do Mato Grosso do Sul	Instituto Adolfo Lutz, Interdiciplinary Procedures Center, Strategic Laboratory	Claudio Tavares Sacchi et al
EPI_ISL_1201883	LACEN do Mato Grosso do Sul	Instituto Adolfo Lutz, Interdiciplinary Procedures Center, Strategic Laboratory	Claudio Tavares Sacchi et al
EPI_ISL_1358307	LACEN do Mato Grosso do Sul	Instituto Adolfo Lutz, Interdiciplinary Procedures Center, Strategic Laboratory	Claudio Tavares Sacchi et al
EPI_ISL_1358305	LACEN do Mato Grosso do Sul	Instituto Adolfo Lutz, Interdiciplinary Procedures Center, Strategic Laboratory	Claudio Tavares Sacchi et al
EPI_ISL_1358309	LACEN do Mato Grosso do Sul	Instituto Adolfo Lutz, Interdiciplinary Procedures Center, Strategic Laboratory	Claudio Tavares Sacchi et al
EPI_ISL_1358304	LACEN do Mato Grosso do Sul	Instituto Adolfo Lutz, Interdiciplinary Procedures Center, Strategic Laboratory	Claudio Tavares Sacchi et al
EPI_ISL_1358308	LACEN do Mato Grosso do Sul	Instituto Adolfo Lutz, Interdiciplinary Procedures Center, Strategic Laboratory	Claudio Tavares Sacchi et al

EPI_ISL_1358306	LACEN do Mato Grosso do Sul	Instituto Adolfo Lutz, Interdiciplinary Procedures Center, Strategic Laboratory	Claudio Tavares Sacchi et al
EPI_ISL_1358316	LACEN do Mato Grosso do Sul	Instituto Adolfo Lutz, Interdiciplinary Procedures Center, Strategic Laboratory	Claudio Tavares Sacchi et al
EPI_ISL_1358317	LACEN do Mato Grosso do Sul	Instituto Adolfo Lutz, Interdiciplinary Procedures Center, Strategic Laboratory	Claudio Tavares Sacchi et al
EPI_ISL_1358311	LACEN do Mato Grosso do Sul	Instituto Adolfo Lutz, Interdiciplinary Procedures Center, Strategic Laboratory	Claudio Tavares Sacchi et al
EPI_ISL_1358312	LACEN do Mato Grosso do Sul	Instituto Adolfo Lutz, Interdiciplinary Procedures Center, Strategic Laboratory	Claudio Tavares Sacchi et al
EPI_ISL_1358313	LACEN do Mato Grosso do Sul	Instituto Adolfo Lutz, Interdiciplinary Procedures Center, Strategic Laboratory	Claudio Tavares Sacchi et al
EPI_ISL_1358310	LACEN do Mato Grosso do Sul	Instituto Adolfo Lutz, Interdiciplinary Procedures Center, Strategic Laboratory	Claudio Tavares Sacchi et al
EPI_ISL_1358314	LACEN do Mato Grosso do Sul	Instituto Adolfo Lutz, Interdiciplinary Procedures Center, Strategic Laboratory	Claudio Tavares Sacchi et al
EPI_ISL_1358315	LACEN do Mato Grosso do Sul	Instituto Adolfo Lutz, Interdiciplinary Procedures Center, Strategic Laboratory	Claudio Tavares Sacchi et al
EPI_ISL_1468432	LACEN do Mato Grosso do Sul	Instituto Adolfo Lutz, Interdiciplinary Procedures Center, Strategic Laboratory	Claudio Tavares Sacchi et al
EPI_ISL_1468433	LACEN do Mato Grosso do Sul	Instituto Adolfo Lutz, Interdiciplinary Procedures Center, Strategic Laboratory	Claudio Tavares Sacchi et al
EPI_ISL_1468434	LACEN do Mato Grosso do Sul	Instituto Adolfo Lutz, Interdiciplinary Procedures Center, Strategic Laboratory	Claudio Tavares Sacchi et al
EPI_ISL_1468435	LACEN do Mato Grosso do Sul	Instituto Adolfo Lutz, Interdiciplinary Procedures Center, Strategic Laboratory	Claudio Tavares Sacchi et al
EPI_ISL_1468436	LACEN do Mato Grosso do Sul	Instituto Adolfo Lutz, Interdiciplinary Procedures Center, Strategic Laboratory	Claudio Tavares Sacchi et al
EPI_ISL_1468437	LACEN do Mato Grosso do Sul	Instituto Adolfo Lutz, Interdiciplinary Procedures Center, Strategic Laboratory	Claudio Tavares Sacchi et al
EPI_ISL_1468438	LACEN do Mato Grosso do Sul	Instituto Adolfo Lutz, Interdiciplinary Procedures Center, Strategic Laboratory	Claudio Tavares Sacchi et al
EPI_ISL_1468439	LACEN do Mato Grosso do Sul	Instituto Adolfo Lutz, Interdiciplinary Procedures Center, Strategic Laboratory	Claudio Tavares Sacchi et al
EPI_ISL_1468440	LACEN do Mato Grosso do Sul	Instituto Adolfo Lutz, Interdiciplinary Procedures Center, Strategic Laboratory	Claudio Tavares Sacchi et al
EPI_ISL_1468441	LACEN do Mato Grosso do Sul	Instituto Adolfo Lutz, Interdiciplinary Procedures Center, Strategic Laboratory	Claudio Tavares Sacchi et al
EPI_ISL_1381066	LACEN do Mato Grosso do Sul	Instituto Adolfo Lutz, Interdiciplinary Procedures Center, Strategic Laboratory	Claudio Tavares Sacchi et al
EPI_ISL_1381067	LACEN do Mato Grosso do Sul	Instituto Adolfo Lutz, Interdiciplinary Procedures Center, Strategic Laboratory	Claudio Tavares Sacchi et al
EPI_ISL_940613	LACEN-PI DR. Costa Alvarenga	Instituto Adolfo Lutz, Interdiciplinary Procedures Center, Strategic Laboratory	Claudio Tavares Sacchi et al

EPI_ISL_1293052	LACEN de Rondonia	Instituto Adolfo Lutz, Interdiciplinary Procedures Center, Strategic Laboratory	Claudio Tavares Sacchi et al
EPI_ISL_1293053	LACEN de Rondonia	Instituto Adolfo Lutz, Interdiciplinary Procedures Center, Strategic Laboratory	Claudio Tavares Sacchi et al
EPI_ISL_1293054	LACEN de Rondonia	Instituto Adolfo Lutz, Interdiciplinary Procedures Center, Strategic Laboratory	Claudio Tavares Sacchi et al
EPI_ISL_1303499	LACEN de Rondonia	Instituto Adolfo Lutz, Interdiciplinary Procedures Center, Strategic Laboratory	Claudio Tavares Sacchi et al
EPI_ISL_1303500	LACEN de Rondonia	Instituto Adolfo Lutz, Interdiciplinary Procedures Center, Strategic Laboratory	Claudio Tavares Sacchi et al
EPI_ISL_1303501	LACEN de Rondonia	Instituto Adolfo Lutz, Interdiciplinary Procedures Center, Strategic Laboratory	Claudio Tavares Sacchi et al
EPI_ISL_1303502	LACEN de Rondonia	Instituto Adolfo Lutz, Interdiciplinary Procedures Center, Strategic Laboratory	Claudio Tavares Sacchi et al
EPI_ISL_1303504	LACEN de Rondonia	Instituto Adolfo Lutz, Interdiciplinary Procedures Center, Strategic Laboratory	Claudio Tavares Sacchi et al
EPI_ISL_1303505	LACEN de Rondonia	Instituto Adolfo Lutz, Interdiciplinary Procedures Center, Strategic Laboratory	Claudio Tavares Sacchi et al
EPI_ISL_1520107	LACEN do Estado de Rondonia	Instituto Adolfo Lutz, Interdiciplinary Procedures Center, Strategic Laboratory	Claudio Tavares Sacchi et al
EPI_ISL_1520108	LACEN do Estado de Rondonia	Instituto Adolfo Lutz, Interdiciplinary Procedures Center, Strategic Laboratory	Claudio Tavares Sacchi et al
EPI_ISL_1520109	LACEN do Estado de Rondonia	Instituto Adolfo Lutz, Interdiciplinary Procedures Center, Strategic Laboratory	Claudio Tavares Sacchi et al
EPI_ISL_1493600	LACEN do Estado de Rondonia	Instituto Adolfo Lutz, Interdiciplinary Procedures Center, Strategic Laboratory	Claudio Tavares Sacchi et al
EPI_ISL_1493599	LACEN do Estado de Rondonia	Instituto Adolfo Lutz, Interdiciplinary Procedures Center, Strategic Laboratory	Claudio Tavares Sacchi et al
EPI_ISL_1493597	LACEN do Estado de Rondonia	Instituto Adolfo Lutz, Interdiciplinary Procedures Center, Strategic Laboratory	Claudio Tavares Sacchi et al
EPI_ISL_1493598	LACEN do Estado de Rondonia	Instituto Adolfo Lutz, Interdiciplinary Procedures Center, Strategic Laboratory	Claudio Tavares Sacchi et al
EPI_ISL_1493595	LACEN do Estado de Rondonia	Instituto Adolfo Lutz, Interdiciplinary Procedures Center, Strategic Laboratory	Claudio Tavares Sacchi et al
EPI_ISL_1493596	LACEN do Estado de Rondonia	Instituto Adolfo Lutz, Interdiciplinary Procedures Center, Strategic Laboratory	Claudio Tavares Sacchi et al
EPI_ISL_1494924	LACEN do Estado de Rondonia	Instituto Adolfo Lutz, Interdiciplinary Procedures Center, Strategic Laboratory	Claudio Tavares Sacchi et al
EPI_ISL_1493584	LACEN do Estado de Rondonia	Instituto Adolfo Lutz, Interdiciplinary Procedures Center, Strategic Laboratory	Claudio Tavares Sacchi et al
EPI_ISL_1493579	LACEN do Estado de Rondonia	Instituto Adolfo Lutz, Interdiciplinary Procedures Center, Strategic Laboratory	Claudio Tavares Sacchi et al
EPI_ISL_1493578	LACEN do Estado de Rondonia	Instituto Adolfo Lutz, Interdiciplinary Procedures Center, Strategic Laboratory	Claudio Tavares Sacchi et al

EPI_ISL_940630	Hospital Geral de Sao Paulo	Instituto Adolfo Lutz, Interdiciplinary Procedures Center, Strategic Laboratory	Claudio Tavares Sacchi et al
EPI_ISL_943967	Hospital Geral de Sao Paulo	Instituto Adolfo Lutz, Interdiciplinary Procedures Center, Strategic Laboratory	Claudio Tavares Sacchi et al
EPI_ISL_943968	Hospital Geral de Sao Paulo	Instituto Adolfo Lutz, Interdiciplinary Procedures Center, Strategic Laboratory	Claudio Tavares Sacchi et al
EPI_ISL_943969	Hospital Geral de Sao Paulo	Instituto Adolfo Lutz, Interdiciplinary Procedures Center, Strategic Laboratory	Claudio Tavares Sacchi et al
EPI_ISL_943970	Hospital Geral de Sao Paulo	Instituto Adolfo Lutz, Interdiciplinary Procedures Center, Strategic Laboratory	Claudio Tavares Sacchi et al
EPI_ISL_943971	Hospital Geral de Sao Paulo	Instituto Adolfo Lutz, Interdiciplinary Procedures Center, Strategic Laboratory	Claudio Tavares Sacchi et al
EPI_ISL_1121316	LACEN do Rio Grande do Sul	Instituto Adolfo Lutz, Interdiciplinary Procedures Center, Strategic Laboratory	Claudio Tavares Sacchi et al
EPI_ISL_985318	LACEN de Santa Catarina	Instituto Adolfo Lutz, Interdiciplinary Procedures Center, Strategic Laboratory	Claudio Tavares Sacchi et al
EPI_ISL_985319	LACEN de Santa Catarina	Instituto Adolfo Lutz, Interdiciplinary Procedures Center, Strategic Laboratory	Claudio Tavares Sacchi et al
EPI_ISL_414016	Hospital São Joaquim Beneficencia Portuguesa	Instituto Adolfo Lutz, Interdiciplinary Procedures Center, Strategic Laboratory	Claudio Tavares Sacchi et al
EPI_ISL_414015	Hospital São Joaquim Beneficencia Portuguesa	Instituto Adolfo Lutz, Interdiciplinary Procedures Center, Strategic Laboratory	Claudio Tavares Sacchi et al
EPI_ISL_416032	National Influenza Center - Instituto Adolfo Lutz	Instituto Adolfo Lutz, Interdiciplinary Procedures Center, Strategic Laboratory	Claudio Tavares Sacchi et al
EPI_ISL_861680	Hospital e Pronto Socorro Portinari	Instituto Adolfo Lutz, Interdiciplinary Procedures Center, Strategic Laboratory	Claudio Tavares Sacchi et al
EPI_ISL_861682	UPA Vila Santa Catarina	Instituto Adolfo Lutz, Interdiciplinary Procedures Center, Strategic Laboratory	Claudio Tavares Sacchi et al
EPI_ISL_882665	Unidade de Pronto Atendimento Dra Zilda Arns	Instituto Adolfo Lutz, Interdiciplinary Procedures Center, Strategic Laboratory	Claudio Tavares Sacchi et al
EPI_ISL_882671	Hospital Municipal Dr. Guido Guida	Instituto Adolfo Lutz, Interdiciplinary Procedures Center, Strategic Laboratory	Claudio Tavares Sacchi et al
EPI_ISL_882672	Hospital Municipal Dr. Guido Guida	Instituto Adolfo Lutz, Interdiciplinary Procedures Center, Strategic Laboratory	Claudio Tavares Sacchi et al
EPI_ISL_977471	Instituto Adolfo Lutz - Regional de Presidente Prudente	Instituto Adolfo Lutz, Interdiciplinary Procedures Center, Strategic Laboratory	Claudio Tavares Sacchi et al
EPI_ISL_977472	Instituto Adolfo Lutz Central	Instituto Adolfo Lutz, Interdiciplinary Procedures Center, Strategic Laboratory	Claudio Tavares Sacchi et al
EPI_ISL_977474	Instituto Adolfo Lutz Central	Instituto Adolfo Lutz, Interdiciplinary Procedures Center, Strategic Laboratory	Claudio Tavares Sacchi et al
EPI_ISL_985172	Instituto Adolfo Lutz - Regional de Taubate	Instituto Adolfo Lutz, Interdiciplinary Procedures Center, Strategic Laboratory	Claudio Tavares Sacchi et al
EPI_ISL_985173	Instituto Adolfo Lutz - Regional de Taubate	Instituto Adolfo Lutz, Interdiciplinary Procedures Center, Strategic Laboratory	Claudio Tavares Sacchi et al

EPI_ISL_985175	Instituto Adolfo Lutz Central	Instituto Adolfo Lutz, Interdiciplinary Procedures Center, Strategic Laboratory	Claudio Tavares Sacchi et al
EPI_ISL_985171	Instituto Adolfo Lutz - Regional de Taubate	Instituto Adolfo Lutz, Interdiciplinary Procedures Center, Strategic Laboratory	Claudio Tavares Sacchi et al
EPI_ISL_1171620	Instituto Adolfo Lutz Central	Instituto Adolfo Lutz, Interdiciplinary Procedures Center, Strategic Laboratory	Claudio Tavares Sacchi et al
EPI_ISL_977476	Instituto Adolfo Lutz Central	Instituto Adolfo Lutz, Interdiciplinary Procedures Center, Strategic Laboratory	Claudio Tavares Sacchi et al
EPI_ISL_977477	Instituto Adolfo Lutz Central	Instituto Adolfo Lutz, Interdiciplinary Procedures Center, Strategic Laboratory	Claudio Tavares Sacchi et al
EPI_ISL_984243	Instituto Adolfo Lutz - Regional de Marilia	Instituto Adolfo Lutz, Interdiciplinary Procedures Center, Strategic Laboratory	Claudio Tavares Sacchi et al
EPI_ISL_984244	Instituto Adolfo Lutz - Regional de Marilia	Instituto Adolfo Lutz, Interdiciplinary Procedures Center, Strategic Laboratory	Claudio Tavares Sacchi et al
EPI_ISL_984246	Instituto Adolfo Lutz Central	Instituto Adolfo Lutz, Interdiciplinary Procedures Center, Strategic Laboratory	Claudio Tavares Sacchi et al
EPI_ISL_1039697	Instituto Adolfo Lutz Central	Instituto Adolfo Lutz, Interdiciplinary Procedures Center, Strategic Laboratory	Claudio Tavares Sacchi et al
EPI_ISL_1039698	Lab Loc - Itapecerica da Serra	Instituto Adolfo Lutz, Interdiciplinary Procedures Center, Strategic Laboratory	Claudio Tavares Sacchi et al
EPI_ISL_1039699	Instituto Adolfo Lutz - Regional de Taubate	Instituto Adolfo Lutz, Interdiciplinary Procedures Center, Strategic Laboratory	Claudio Tavares Sacchi et al
EPI_ISL_1039700	Instituto Adolfo Lutz Central	Instituto Adolfo Lutz, Interdiciplinary Procedures Center, Strategic Laboratory	Claudio Tavares Sacchi et al
EPI_ISL_1039701	Instituto Adolfo Lutz Central	Instituto Adolfo Lutz, Interdiciplinary Procedures Center, Strategic Laboratory	Claudio Tavares Sacchi et al
EPI_ISL_1039703	Instituto Adolfo Lutz - Regional de Taubate	Instituto Adolfo Lutz, Interdiciplinary Procedures Center, Strategic Laboratory	Claudio Tavares Sacchi et al
EPI_ISL_1039704	Lab Loc - Itapecerica da Serra	Instituto Adolfo Lutz, Interdiciplinary Procedures Center, Strategic Laboratory	Claudio Tavares Sacchi et al
EPI_ISL_1039705	Instituto Adolfo Lutz Central	Instituto Adolfo Lutz, Interdiciplinary Procedures Center, Strategic Laboratory	Claudio Tavares Sacchi et al
EPI_ISL_1039706	Instituto Adolfo Lutz Central	Instituto Adolfo Lutz, Interdiciplinary Procedures Center, Strategic Laboratory	Claudio Tavares Sacchi et al
EPI_ISL_1039707	Instituto Adolfo Lutz Central	Instituto Adolfo Lutz, Interdiciplinary Procedures Center, Strategic Laboratory	Claudio Tavares Sacchi et al
EPI_ISL_1039708	Instituto Adolfo Lutz Central	Instituto Adolfo Lutz, Interdiciplinary Procedures Center, Strategic Laboratory	Claudio Tavares Sacchi et al
EPI_ISL_1039709	Instituto Adolfo Lutz Central	Instituto Adolfo Lutz, Interdiciplinary Procedures Center, Strategic Laboratory	Claudio Tavares Sacchi et al
EPI_ISL_1039710	Instituto Adolfo Lutz Central	Instituto Adolfo Lutz, Interdiciplinary Procedures Center, Strategic Laboratory	Claudio Tavares Sacchi et al
EPI_ISL_977482	Instituto Adolfo Lutz - Regional de Aracatuba	Instituto Adolfo Lutz, Interdiciplinary Procedures Center, Strategic Laboratory	Claudio Tavares Sacchi et al

EPI_ISL_977483	Instituto Adolfo Lutz Central	Instituto Adolfo Lutz, Interdiciplinary Procedures Center, Strategic Laboratory	Claudio Tavares Sacchi et al
EPI_ISL_977487	Instituto Adolfo Lutz Central	Instituto Adolfo Lutz, Interdiciplinary Procedures Center, Strategic Laboratory	Claudio Tavares Sacchi et al
EPI_ISL_977488	Instituto Adolfo Lutz - Regional de Presidente Prudente	Instituto Adolfo Lutz, Interdiciplinary Procedures Center, Strategic Laboratory	Claudio Tavares Sacchi et al
EPI_ISL_984252	IAL Regional de Marilia	Instituto Adolfo Lutz, Interdiciplinary Procedures Center, Strategic Laboratory	Claudio Tavares Sacchi et al
EPI_ISL_984253	IAL Regional de Marilia	Instituto Adolfo Lutz, Interdiciplinary Procedures Center, Strategic Laboratory	Claudio Tavares Sacchi et al
EPI_ISL_984254	IAL Regional de Marilia	Instituto Adolfo Lutz, Interdiciplinary Procedures Center, Strategic Laboratory	Claudio Tavares Sacchi et al
EPI_ISL_984263	IAL Regional de Bauru	Instituto Adolfo Lutz, Interdiciplinary Procedures Center, Strategic Laboratory	Claudio Tavares Sacchi et al
EPI_ISL_1121326	IAL Regional de Bauru	Instituto Adolfo Lutz, Interdiciplinary Procedures Center, Strategic Laboratory	Claudio Tavares Sacchi et al
EPI_ISL_468311	Hospital Municipal Dr Ignacio Proenca de Gouvea	Instituto Adolfo Lutz, Interdiciplinary Procedures Center, Strategic Laboratory	Claudio Tavares Sacchi et al
EPI_ISL_1219031	Aeroporto Internacional de Guarulhos	Instituto Adolfo Lutz, Interdiciplinary Procedures Center, Strategic Laboratory	Claudio Tavares Sacchi et al
EPI_ISL_1171625	IAL Regional de Santos	Instituto Adolfo Lutz, Interdiciplinary Procedures Center, Strategic Laboratory	Claudio Tavares Sacchi et al
EPI_ISL_1171627	IAL Regional de Santos	Instituto Adolfo Lutz, Interdiciplinary Procedures Center, Strategic Laboratory	Claudio Tavares Sacchi et al
EPI_ISL_1171631	IAL Regional de Santos	Instituto Adolfo Lutz, Interdiciplinary Procedures Center, Strategic Laboratory	Claudio Tavares Sacchi et al
EPI_ISL_1171633	IAL Regional de Santos	Instituto Adolfo Lutz, Interdiciplinary Procedures Center, Strategic Laboratory	Claudio Tavares Sacchi et al
EPI_ISL_1171635	IAL Regional de Santos	Instituto Adolfo Lutz, Interdiciplinary Procedures Center, Strategic Laboratory	Claudio Tavares Sacchi et al
EPI_ISL_1196297	IAL Regional de Marilia	Instituto Adolfo Lutz, Interdiciplinary Procedures Center, Strategic Laboratory	Claudio Tavares Sacchi et al
EPI_ISL_1171645	IAL Regional de Marilia	Instituto Adolfo Lutz, Interdiciplinary Procedures Center, Strategic Laboratory	Claudio Tavares Sacchi et al
EPI_ISL_1201890	IAL Regional de Marilia	Instituto Adolfo Lutz, Interdiciplinary Procedures Center, Strategic Laboratory	Claudio Tavares Sacchi et al
EPI_ISL_1201891	IAL Regional de Marilia	Instituto Adolfo Lutz, Interdiciplinary Procedures Center, Strategic Laboratory	Claudio Tavares Sacchi et al
EPI_ISL_1201892	IAL Regional de Marilia	Instituto Adolfo Lutz, Interdiciplinary Procedures Center, Strategic Laboratory	Claudio Tavares Sacchi et al
EPI_ISL_523969	Hospital Sao Paulo de Ensino da Unifesp	Instituto Adolfo Lutz, Interdiciplinary Procedures Center, Strategic Laboratory	Claudio Tavares Sacchi et al
EPI_ISL_1079008	IAL Regional de Bauru	Instituto Adolfo Lutz, Interdiciplinary Procedures Center, Strategic Laboratory	Claudio Tavares Sacchi et al

EPI_ISL_1086051	IAL Regional de Bauru	Instituto Adolfo Lutz, Interdiciplinary Procedures Center, Strategic Laboratory	Claudio Tavares Sacchi et al
EPI_ISL_1078999	IAL Regional de Bauru	Instituto Adolfo Lutz, Interdiciplinary Procedures Center, Strategic Laboratory	Claudio Tavares Sacchi et al
EPI_ISL_1171654	IAL Regional de Presidente Prudente	Instituto Adolfo Lutz, Interdiciplinary Procedures Center, Strategic Laboratory	Claudio Tavares Sacchi et al
EPI_ISL_1171667	IAL Regional de Presidente Prudente	Instituto Adolfo Lutz, Interdiciplinary Procedures Center, Strategic Laboratory	Claudio Tavares Sacchi et al
EPI_ISL_1171668	IAL Regional de Presidente Prudente	Instituto Adolfo Lutz, Interdiciplinary Procedures Center, Strategic Laboratory	Claudio Tavares Sacchi et al
EPI_ISL_1171669	IAL Regional de Presidente Prudente	Instituto Adolfo Lutz, Interdiciplinary Procedures Center, Strategic Laboratory	Claudio Tavares Sacchi et al
EPI_ISL_1219027	IAL Regional de Presidente Prudente	Instituto Adolfo Lutz, Interdiciplinary Procedures Center, Strategic Laboratory	Claudio Tavares Sacchi et al
EPI_ISL_1293057	IAL Regional de Sorocaba	Instituto Adolfo Lutz, Interdiciplinary Procedures Center, Strategic Laboratory	Claudio Tavares Sacchi et al
EPI_ISL_1293069	IAL Regional de Sorocaba	Instituto Adolfo Lutz, Interdiciplinary Procedures Center, Strategic Laboratory	Claudio Tavares Sacchi et al
EPI_ISL_1293071	IAL Regional de Sorocaba	Instituto Adolfo Lutz, Interdiciplinary Procedures Center, Strategic Laboratory	Claudio Tavares Sacchi et al
EPI_ISL_1303527	IAL Regional de São Jose do Rio Preto	Instituto Adolfo Lutz, Interdiciplinary Procedures Center, Strategic Laboratory	Claudio Tavares Sacchi et al
EPI_ISL_1358295	IAL Regional de Santo Andre	Instituto Adolfo Lutz, Interdiciplinary Procedures Center, Strategic Laboratory	Claudio Tavares Sacchi et al
EPI_ISL_1381051	IAL Regional de Santo Andre	Instituto Adolfo Lutz, Interdiciplinary Procedures Center, Strategic Laboratory	Claudio Tavares Sacchi et al
EPI_ISL_1468449	Santa Casa de Aracatuba Hospital Sagrado Coracao de Jesus	Instituto Adolfo Lutz, Interdiciplinary Procedures Center, Strategic Laboratory	Claudio Tavares Sacchi et al
EPI_ISL_1468453	Santa Casa de Misericordia de Pereira Barreto	Instituto Adolfo Lutz, Interdiciplinary Procedures Center, Strategic Laboratory	Claudio Tavares Sacchi et al
EPI_ISL_1468454	Santa Casa de Andradina	Instituto Adolfo Lutz, Interdiciplinary Procedures Center, Strategic Laboratory	Claudio Tavares Sacchi et al
EPI_ISL_1468459	Secretaria Municipal de Saude de Valparaiso SP	Instituto Adolfo Lutz, Interdiciplinary Procedures Center, Strategic Laboratory	Claudio Tavares Sacchi et al
EPI_ISL_1468469	Secretaria Municipal de Saude Porto Ferreira	Instituto Adolfo Lutz, Interdiciplinary Procedures Center, Strategic Laboratory	Claudio Tavares Sacchi et al
EPI_ISL_1520113	Hospital Santo Antonio de Juquia Juquia	Instituto Adolfo Lutz, Interdiciplinary Procedures Center, Strategic Laboratory	Claudio Tavares Sacchi et al
EPI_ISL_1533697	Hospital Estadual de Vila Alpina	Instituto Adolfo Lutz, Interdiciplinary Procedures Center, Strategic Laboratory	Claudio Tavares Sacchi et al
EPI_ISL_1533700	Hospital Estadual de Sapopemba Sao Paulo	Instituto Adolfo Lutz, Interdiciplinary Procedures Center, Strategic Laboratory	Claudio Tavares Sacchi et al
EPI_ISL_527868	Hospital e Maternidade do Braz	Instituto Adolfo Lutz, Interdiciplinary Procedures Center, Strategic Laboratory	Claudio Tavares Sacchi et al

EPI_ISL_1628346	Instituto Adolfo Lutz - Regional de Rio Claro	Instituto Adolfo Lutz, Interdiciplinary Procedures Center, Strategic Laboratory	Claudio Tavares Sacchi et al
EPI_ISL_524468	Hospital Municipal Vereador Jose Storopolli	Instituto Adolfo Lutz, Interdiciplinary Procedures Center, Strategic Laboratory	Claudio Tavares Sacchi et al
EPI_ISL_693196	Hospital Santa Clara	Instituto Adolfo Lutz, Interdiciplinary Procedures Center, Strategic Laboratory	Claudio Tavares Sacchi et al
EPI_ISL_693198	Santa Casa de Misericordia de Sao Paulo - Hospital Central	Instituto Adolfo Lutz, Interdiciplinary Procedures Center, Strategic Laboratory	Claudio Tavares Sacchi et al
EPI_ISL_693199	Hospital do Servidor Publico Estadual Francisco Morato de Oliveira	Instituto Adolfo Lutz, Interdiciplinary Procedures Center, Strategic Laboratory	Claudio Tavares Sacchi et al
EPI_ISL_534319	Hospital do Serv Pub ESTAFCO Morato de Oliveira	Instituto Adolfo Lutz, Interdiciplinary Procedures Center, Strategic Laboratory	Claudio Tavares Sacchi et al
EPI_ISL_534320	Hospital do Serv Pub ESTAFCO Morato de Oliveira	Instituto Adolfo Lutz, Interdiciplinary Procedures Center, Strategic Laboratory	Claudio Tavares Sacchi et al
EPI_ISL_524464	Santa Casa de Santa Isabel	Instituto Adolfo Lutz, Interdiciplinary Procedures Center, Strategic Laboratory	Claudio Tavares Sacchi et al
EPI_ISL_527858	Pronto Atendimento Sancta Maggiore Jardim Paulista	Instituto Adolfo Lutz, Interdiciplinary Procedures Center, Strategic Laboratory	Claudio Tavares Sacchi et al
EPI_ISL_527856	Hospital Municipal Prof. Waldomiro de Paula	Instituto Adolfo Lutz, Interdiciplinary Procedures Center, Strategic Laboratory	Claudio Tavares Sacchi et al
EPI_ISL_534314	Hospital Universitario da USP de SP	Instituto Adolfo Lutz, Interdiciplinary Procedures Center, Strategic Laboratory	Claudio Tavares Sacchi et al
EPI_ISL_534318	Hospital Municipal Antonio Giglio	Instituto Adolfo Lutz, Interdiciplinary Procedures Center, Strategic Laboratory	Claudio Tavares Sacchi et al
EPI_ISL_547573	Vigilância em Saúde de Cajamar	Instituto Adolfo Lutz, Interdiciplinary Procedures Center, Strategic Laboratory	Claudio Tavares Sacchi et al
EPI_ISL_583495	Serviço de Verificação de Óbitos SVO Guaruulhos	Instituto Adolfo Lutz, Interdiciplinary Procedures Center, Strategic Laboratory	Claudio Tavares Sacchi et al
EPI_ISL_547575	SVO Jundiaí	Instituto Adolfo Lutz, Interdiciplinary Procedures Center, Strategic Laboratory	Claudio Tavares Sacchi et al
EPI_ISL_693207	Cs II Doutor Antonio Vicoso Moreira de Rezende	Instituto Adolfo Lutz, Interdiciplinary Procedures Center, Strategic Laboratory	Claudio Tavares Sacchi et al
EPI_ISL_583499	Distrito Sanitario Sul Campinas	Instituto Adolfo Lutz, Interdiciplinary Procedures Center, Strategic Laboratory	Claudio Tavares Sacchi et al
EPI_ISL_574597	Secretaria Municipal de Saude de Jarinu	Instituto Adolfo Lutz, Interdiciplinary Procedures Center, Strategic Laboratory	Claudio Tavares Sacchi et al
EPI_ISL_574598	Servico de Verificacao de Obito SVO	Instituto Adolfo Lutz, Interdiciplinary Procedures Center, Strategic Laboratory	Claudio Tavares Sacchi et al
EPI_ISL_583502	Serv de Vig Sanitaria Epidemio e CTRL de Zoonoses Guaruja	Instituto Adolfo Lutz, Interdiciplinary Procedures Center, Strategic Laboratory	Claudio Tavares Sacchi et al
EPI_ISL_583505	Casa de Saude Stella Maris	Instituto Adolfo Lutz, Interdiciplinary Procedures Center, Strategic Laboratory	Claudio Tavares Sacchi et al
EPI_ISL_693208	Hospital Municipal Antonio Giglio	Instituto Adolfo Lutz, Interdiciplinary Procedures Center, Strategic Laboratory	Claudio Tavares Sacchi et al

EPI_ISL_547577	Hospital e Maternidade Nossa Senhora das Graças	Instituto Adolfo Lutz, Interdiciplinary Procedures Center, Strategic Laboratory	Claudio Tavares Sacchi et al
EPI_ISL_547579	Santa Casa de Misericórdia de Araçatuba	Instituto Adolfo Lutz, Interdiciplinary Procedures Center, Strategic Laboratory	Claudio Tavares Sacchi et al
EPI_ISL_574594	Hospital Escola da Universidade de Taubate	Instituto Adolfo Lutz, Interdiciplinary Procedures Center, Strategic Laboratory	Claudio Tavares Sacchi et al
EPI_ISL_603037	Hospital Geral de Pedreira	Instituto Adolfo Lutz, Interdiciplinary Procedures Center, Strategic Laboratory	Claudio Tavares Sacchi et al
EPI_ISL_471552	Hospital Sancta Maggiore	Instituto Adolfo Lutz, Interdiciplinary Procedures Center, Strategic Laboratory	Claudio Tavares Sacchi et al
EPI_ISL_735396	Hospital de Camplanha COVID 19 SER	Instituto Adolfo Lutz, Interdiciplinary Procedures Center, Strategic Laboratory	Claudio Tavares Sacchi et al
EPI_ISL_693215	Secretaria Municipal de Saúde de Iracemapolis	Instituto Adolfo Lutz, Interdiciplinary Procedures Center, Strategic Laboratory	Claudio Tavares Sacchi et al
EPI_ISL_471545	Hospital Sao Paulo de Ensino da Unifesp	Instituto Adolfo Lutz, Interdiciplinary Procedures Center, Strategic Laboratory	Claudio Tavares Sacchi et al
EPI_ISL_515566	PS Municipal Dr Lauro Ribas Braga	Instituto Adolfo Lutz, Interdiciplinary Procedures Center, Strategic Laboratory	Claudio Tavares Sacchi et al
EPI_ISL_515554	Pronto Socorro Municipal de Perus	Instituto Adolfo Lutz, Interdiciplinary Procedures Center, Strategic Laboratory	Claudio Tavares Sacchi et al
EPI_ISL_735402	Instituto Adolfo Lutz - Regional de Rio Claro	Instituto Adolfo Lutz, Interdiciplinary Procedures Center, Strategic Laboratory	Claudio Tavares Sacchi et al
EPI_ISL_735403	Instituto Adolfo Lutz - Regional de Rio Claro	Instituto Adolfo Lutz, Interdiciplinary Procedures Center, Strategic Laboratory	Claudio Tavares Sacchi et al
EPI_ISL_735404	Instituto Adolfo Lutz - Regional de Rio Claro	Instituto Adolfo Lutz, Interdiciplinary Procedures Center, Strategic Laboratory	Claudio Tavares Sacchi et al
EPI_ISL_735406	Unidade de Pronto Atendimento UPA I Sta Isabel	Instituto Adolfo Lutz, Interdiciplinary Procedures Center, Strategic Laboratory	Claudio Tavares Sacchi et al
EPI_ISL_735412	Hospital e Pronto Socorro Portinari	Instituto Adolfo Lutz, Interdiciplinary Procedures Center, Strategic Laboratory	Claudio Tavares Sacchi et al
EPI_ISL_735413	Militello Centro de Diagnosticos e Biopesquisa Clinica	Instituto Adolfo Lutz, Interdiciplinary Procedures Center, Strategic Laboratory	Claudio Tavares Sacchi et al
EPI_ISL_735418	Hospital Regional do Vale do Paraiba	Instituto Adolfo Lutz, Interdiciplinary Procedures Center, Strategic Laboratory	Claudio Tavares Sacchi et al
EPI_ISL_735422	UBS Dematchi	Instituto Adolfo Lutz, Interdiciplinary Procedures Center, Strategic Laboratory	Claudio Tavares Sacchi et al
EPI_ISL_776764	Instituto Adolfo Lutz - Regional de Santo Andre	Instituto Adolfo Lutz, Interdiciplinary Procedures Center, Strategic Laboratory	Claudio Tavares Sacchi et al
EPI_ISL_776765	Instituto Adolfo Lutz - Regional de Santo Andre	Instituto Adolfo Lutz, Interdiciplinary Procedures Center, Strategic Laboratory	Claudio Tavares Sacchi et al
EPI_ISL_792116	Instituto Adolfo Lutz - Regional de Taubate	Instituto Adolfo Lutz, Interdiciplinary Procedures Center, Strategic Laboratory	Claudio Tavares Sacchi et al
EPI_ISL_735433	Posto de Atendimento Saude Cidade Pasc Cajati	Instituto Adolfo Lutz, Interdiciplinary Procedures Center, Strategic Laboratory	Claudio Tavares Sacchi et al

EPI_ISL_940608	Laboratório Sao Lucas	Instituto Adolfo Lutz, Interdiciplinary Procedures Center, Strategic Laboratory	Claudio Tavares Sacchi et al
EPI_ISL_833156	Instituto Adolfo Lutz - Regional de Sorocaba	Instituto Adolfo Lutz, Interdiciplinary Procedures Center, Strategic Laboratory	Claudio Tavares Sacchi et al
EPI_ISL_861627	Instituto Adolfo Lutz - Central	Instituto Adolfo Lutz, Interdiciplinary Procedures Center, Strategic Laboratory	Claudio Tavares Sacchi et al
EPI_ISL_776752	Instituto Adolfo Lutz - Central	Instituto Adolfo Lutz, Interdiciplinary Procedures Center, Strategic Laboratory	Claudio Tavares Sacchi et al
EPI_ISL_792108	Instituto Adolfo Lutz - Central	Instituto Adolfo Lutz, Interdiciplinary Procedures Center, Strategic Laboratory	Claudio Tavares Sacchi et al
EPI_ISL_792107	Instituto Adolfo Lutz - Central	Instituto Adolfo Lutz, Interdiciplinary Procedures Center, Strategic Laboratory	Claudio Tavares Sacchi et al
EPI_ISL_708530	Secretaria Municipal de Saude de Fernandópolis	Instituto Adolfo Lutz, Interdiciplinary Procedures Center, Strategic Laboratory	Claudio Tavares Sacchi et al
EPI_ISL_861636	Hospital Geral de Sao Mateus São Paulo	Instituto Adolfo Lutz, Interdiciplinary Procedures Center, Strategic Laboratory	Claudio Tavares Sacchi et al
EPI_ISL_861639	Hospital Sao Paulo de Ensino da Unifesp	Instituto Adolfo Lutz, Interdiciplinary Procedures Center, Strategic Laboratory	Claudio Tavares Sacchi et al
EPI_ISL_776766	Instituto Adolfo Lutz - Regional de Santo Andre	Instituto Adolfo Lutz, Interdiciplinary Procedures Center, Strategic Laboratory	Claudio Tavares Sacchi et al
EPI_ISL_776767	Instituto Adolfo Lutz - Regional de Marilia	Instituto Adolfo Lutz, Interdiciplinary Procedures Center, Strategic Laboratory	Claudio Tavares Sacchi et al
EPI_ISL_776768	Instituto Adolfo Lutz - Regional de Aracatuba	Instituto Adolfo Lutz, Interdiciplinary Procedures Center, Strategic Laboratory	Claudio Tavares Sacchi et al
EPI_ISL_755641	Instituto Adolfo Lutz - Regional de Santo Andre	Instituto Adolfo Lutz, Interdiciplinary Procedures Center, Strategic Laboratory	Claudio Tavares Sacchi et al
EPI_ISL_755642	Instituto Adolfo Lutz - Central	Instituto Adolfo Lutz, Interdiciplinary Procedures Center, Strategic Laboratory	Claudio Tavares Sacchi et al
EPI_ISL_755647	Instituto Adolfo Lutz - Regional de Santo Andre	Instituto Adolfo Lutz, Interdiciplinary Procedures Center, Strategic Laboratory	Claudio Tavares Sacchi et al
EPI_ISL_755648	Instituto Adolfo Lutz - Regional de Taubate	Instituto Adolfo Lutz, Interdiciplinary Procedures Center, Strategic Laboratory	Claudio Tavares Sacchi et al
EPI_ISL_755653	Instituto Adolfo Lutz - Central	Instituto Adolfo Lutz, Interdiciplinary Procedures Center, Strategic Laboratory	Claudio Tavares Sacchi et al
EPI_ISL_755654	Instituto Adolfo Lutz - Central	Instituto Adolfo Lutz, Interdiciplinary Procedures Center, Strategic Laboratory	Claudio Tavares Sacchi et al
EPI_ISL_861668	Centro de Triagem Covid19	Instituto Adolfo Lutz, Interdiciplinary Procedures Center, Strategic Laboratory	Claudio Tavares Sacchi et al
EPI_ISL_833157	Instituto Adolfo Lutz - Regional de Santo Andre	Instituto Adolfo Lutz, Interdiciplinary Procedures Center, Strategic Laboratory	Claudio Tavares Sacchi et al
EPI_ISL_833158	Instituto Adolfo Lutz - Regional de Santo Andre	Instituto Adolfo Lutz, Interdiciplinary Procedures Center, Strategic Laboratory	Claudio Tavares Sacchi et al
EPI_ISL_861669	Lab LOC - Itapecerica da Serra	Instituto Adolfo Lutz, Interdiciplinary Procedures Center, Strategic Laboratory	Claudio Tavares Sacchi et al

EPI_ISL_837053	UBS Darcy Alves e Robalinho	Instituto Adolfo Lutz, Interdiciplinary Procedures Center, Strategic Laboratory	Claudio Tavares Sacchi et al
EPI_ISL_837054	UBS Jose Sabino Ferreira	Instituto Adolfo Lutz, Interdiciplinary Procedures Center, Strategic Laboratory	Claudio Tavares Sacchi et al
EPI_ISL_833163	Instituto Adolfo Lutz - Regional de Marilia	Instituto Adolfo Lutz, Interdiciplinary Procedures Center, Strategic Laboratory	Claudio Tavares Sacchi et al
EPI_ISL_833164	Secretaria Municipal de Saude de Santa Barbara d'oeste	Instituto Adolfo Lutz, Interdiciplinary Procedures Center, Strategic Laboratory	Claudio Tavares Sacchi et al
EPI_ISL_882658	Secretaria Municipal de Saude	Instituto Adolfo Lutz, Interdiciplinary Procedures Center, Strategic Laboratory	Claudio Tavares Sacchi et al
EPI_ISL_882659	Centro de Triagem Covid19	Instituto Adolfo Lutz, Interdiciplinary Procedures Center, Strategic Laboratory	Claudio Tavares Sacchi et al
EPI_ISL_882660	Hospital Municipal Prefeito Waldemar Costa Filho	Instituto Adolfo Lutz, Interdiciplinary Procedures Center, Strategic Laboratory	Claudio Tavares Sacchi et al
EPI_ISL_861671	Hospital Municipal Prefeito Waldemar Costa Filho	Instituto Adolfo Lutz, Interdiciplinary Procedures Center, Strategic Laboratory	Claudio Tavares Sacchi et al
EPI_ISL_861672	Day Hospital de Ermelino Matarazzo	Instituto Adolfo Lutz, Interdiciplinary Procedures Center, Strategic Laboratory	Claudio Tavares Sacchi et al
EPI_ISL_861673	PA Novo Osasco	Instituto Adolfo Lutz, Interdiciplinary Procedures Center, Strategic Laboratory	Claudio Tavares Sacchi et al
EPI_ISL_861645	Hospital e Pronto Socorro Comunitario Vila Iolanda	Instituto Adolfo Lutz, Interdiciplinary Procedures Center, Strategic Laboratory	Claudio Tavares Sacchi et al
EPI_ISL_861646	Hospital Santa Marcelina Sao Paulo	Instituto Adolfo Lutz, Interdiciplinary Procedures Center, Strategic Laboratory	Claudio Tavares Sacchi et al
EPI_ISL_861652	AMA Wamberto Dias da Costa	Instituto Adolfo Lutz, Interdiciplinary Procedures Center, Strategic Laboratory	Claudio Tavares Sacchi et al
EPI_ISL_861658	Hospital Municipal Antônio Giglio	Instituto Adolfo Lutz, Interdiciplinary Procedures Center, Strategic Laboratory	Claudio Tavares Sacchi et al
EPI_ISL_861659	PS e Maternidade Nair Fonseca Leitao Arantes	Instituto Adolfo Lutz, Interdiciplinary Procedures Center, Strategic Laboratory	Claudio Tavares Sacchi et al
EPI_ISL_861660	PS e Maternidade Nair Fonseca Leitao Arantes	Instituto Adolfo Lutz, Interdiciplinary Procedures Center, Strategic Laboratory	Claudio Tavares Sacchi et al
EPI_ISL_861676	UPA Vila Santa Catarina	Instituto Adolfo Lutz, Interdiciplinary Procedures Center, Strategic Laboratory	Claudio Tavares Sacchi et al
EPI_ISL_861679	Instituto Adolfo Lutz - Regional de Taubate	Instituto Adolfo Lutz, Interdiciplinary Procedures Center, Strategic Laboratory	Claudio Tavares Sacchi et al
EPI_ISL_1493591	Centro de Saude II Dr Alcides Facundo Arroyo	Instituto Adolfo Lutz, Interdiciplinary Procedures Center, Strategic Laboratory	Claudio Tavares Sacchi et al
EPI_ISL_1201886	Aeroporto Internacional de Guarulhos	Instituto Adolfo Lutz, Interdiciplinary Procedures Center, Strategic Laboratory	Claudio Tavares Sacchi et al
EPI_ISL_943973	LACEN do Estado de Tocantins	Instituto Adolfo Lutz, Interdiciplinary Procedures Center, Strategic Laboratory	Claudio Tavares Sacchi et al
EPI_ISL_943974	LACEN do Estado de Tocantins	Instituto Adolfo Lutz, Interdiciplinary Procedures Center, Strategic Laboratory	Claudio Tavares Sacchi et al

EPI_ISL_943975	LACEN do Estado de Tocantins	Instituto Adolfo Lutz, Interdiciplinary Procedures Center, Strategic Laboratory	Claudio Tavares Sacchi et al
EPI_ISL_943976	LACEN do Estado de Tocantins	Instituto Adolfo Lutz, Interdiciplinary Procedures Center, Strategic Laboratory	Claudio Tavares Sacchi et al
EPI_ISL_943977	LACEN do Estado de Tocantins	Instituto Adolfo Lutz, Interdiciplinary Procedures Center, Strategic Laboratory	Claudio Tavares Sacchi et al
EPI_ISL_943978	LACEN do Estado de Tocantins	Instituto Adolfo Lutz, Interdiciplinary Procedures Center, Strategic Laboratory	Claudio Tavares Sacchi et al
EPI_ISL_943979	LACEN do Estado de Tocantins	Instituto Adolfo Lutz, Interdiciplinary Procedures Center, Strategic Laboratory	Claudio Tavares Sacchi et al
EPI_ISL_943980	LACEN do Estado de Tocantins	Instituto Adolfo Lutz, Interdiciplinary Procedures Center, Strategic Laboratory	Claudio Tavares Sacchi et al
EPI_ISL_943981	LACEN do Estado de Tocantins	Instituto Adolfo Lutz, Interdiciplinary Procedures Center, Strategic Laboratory	Claudio Tavares Sacchi et al
EPI_ISL_943982	LACEN do Estado de Tocantins	Instituto Adolfo Lutz, Interdiciplinary Procedures Center, Strategic Laboratory	Claudio Tavares Sacchi et al
EPI_ISL_943983	LACEN do Estado de Tocantins	Instituto Adolfo Lutz, Interdiciplinary Procedures Center, Strategic Laboratory	Claudio Tavares Sacchi et al
EPI_ISL_943991	LACEN do Estado de Tocantins	Instituto Adolfo Lutz, Interdiciplinary Procedures Center, Strategic Laboratory	Claudio Tavares Sacchi et al
EPI_ISL_943985	LACEN do Estado de Tocantins	Instituto Adolfo Lutz, Interdiciplinary Procedures Center, Strategic Laboratory	Claudio Tavares Sacchi et al
EPI_ISL_943986	LACEN do Estado de Tocantins	Instituto Adolfo Lutz, Interdiciplinary Procedures Center, Strategic Laboratory	Claudio Tavares Sacchi et al
EPI_ISL_943987	LACEN do Estado de Tocantins	Instituto Adolfo Lutz, Interdiciplinary Procedures Center, Strategic Laboratory	Claudio Tavares Sacchi et al
EPI_ISL_1303509	LACEN do Estado de Tocantins	Instituto Adolfo Lutz, Interdiciplinary Procedures Center, Strategic Laboratory	Claudio Tavares Sacchi et al
EPI_ISL_1358300	Lacen de Tocantins	Instituto Adolfo Lutz, Interdiciplinary Procedures Center, Strategic Laboratory	Claudio Tavares Sacchi et al
EPI_ISL_1358301	Lacen de Tocantins	Instituto Adolfo Lutz, Interdiciplinary Procedures Center, Strategic Laboratory	Claudio Tavares Sacchi et al
EPI_ISL_1358302	Lacen de Tocantins	Instituto Adolfo Lutz, Interdiciplinary Procedures Center, Strategic Laboratory	Claudio Tavares Sacchi et al
EPI_ISL_1358303	Lacen de Tocantins	Instituto Adolfo Lutz, Interdiciplinary Procedures Center, Strategic Laboratory	Claudio Tavares Sacchi et al
EPI_ISL_409067	Massachusetts Department of Public Health	Pathogen Discovery, Respiratory Viruses Branch, Division of Viral Diseases, Centers for Disease Control and Prevention	Clinton R. Paden et al
EPI_ISL_1255105	West African Centre for Cell Biology of Infectious Pathogens (WACCBIP), University of Ghana, Volta Road, Legon-Accra, Ghana	West African Centre for Cell Biology of Infectious Pathogens (WACCBIP), University of Ghana, Volta Road, Legon-Accra, Ghana	Collins M. Morang'a et al
EPI_ISL_1255109	West African Centre for Cell Biology of Infectious Pathogens (WACCBIP), University of Ghana, Accra, Ghana	West African Centre for Cell Biology of Infectious Pathogens (WACCBIP), University of Ghana, Volta Road, Legon-Accra, Ghana	Collins M. Morang'a et al
EPI_ISL_1255112	West African Centre for Cell Biology of Infectious Pathogens (WACCBIP), University of Ghana, Accra, Ghana	West African Centre for Cell Biology of Infectious Pathogens (WACCBIP), University of Ghana, Volta Road, Legon-Accra, Ghana	Collins M. Morang'a et al

EPI_ISL_1255113	West African Centre for Cell Biology of Infectious Pathogens (WACCBIP), University of Ghana, Accra, Ghana	West African Centre for Cell Biology of Infectious Pathogens (WACCBIP), University of Ghana, Volta Road, Legon-Accra, Ghana	Collins M. Morang'a et al
EPI_ISL_1255142	West African Centre for Cell Biology of Infectious Pathogens (WACCBIP), University of Ghana, Accra, Ghana	West African Centre for Cell Biology of Infectious Pathogens (WACCBIP), University of Ghana, Volta Road, Legon-Accra, Ghana	Collins M. Morang'a et al
EPI_ISL_1255145	West African Centre for Cell Biology of Infectious Pathogens (WACCBIP), University of Ghana, Accra, Ghana	West African Centre for Cell Biology of Infectious Pathogens (WACCBIP), University of Ghana, Volta Road, Legon-Accra, Ghana	Collins M. Morang'a et al
EPI_ISL_1255153	West African Centre for Cell Biology of Infectious Pathogens (WACCBIP), University of Ghana, Accra, Ghana	West African Centre for Cell Biology of Infectious Pathogens (WACCBIP), University of Ghana, Volta Road, Legon-Accra, Ghana	Collins M. Morang'a et al
EPI_ISL_1255164	West African Centre for Cell Biology of Infectious Pathogens (WACCBIP), University of Ghana, Accra, Ghana	West African Centre for Cell Biology of Infectious Pathogens (WACCBIP), University of Ghana, Volta Road, Legon-Accra, Ghana	Collins M. Morang'a et al
EPI_ISL_1255175	West African Centre for Cell Biology of Infectious Pathogens (WACCBIP), University of Ghana, Accra, Ghana	West African Centre for Cell Biology of Infectious Pathogens (WACCBIP), University of Ghana, Volta Road, Legon-Accra, Ghana	Collins M. Morang'a et al
EPI_ISL_1255178	West African Centre for Cell Biology of Infectious Pathogens (WACCBIP), University of Ghana, Accra, Ghana	West African Centre for Cell Biology of Infectious Pathogens (WACCBIP), University of Ghana, Volta Road, Legon-Accra, Ghana	Collins M. Morang'a et al
EPI_ISL_1255186	West African Centre for Cell Biology of Infectious Pathogens (WACCBIP), University of Ghana, Accra, Ghana	West African Centre for Cell Biology of Infectious Pathogens (WACCBIP), University of Ghana, Volta Road, Legon-Accra, Ghana	Collins M. Morang'a et al
EPI_ISL_1255196	West African Centre for Cell Biology of Infectious Pathogens (WACCBIP), University of Ghana, Accra, Ghana	West African Centre for Cell Biology of Infectious Pathogens (WACCBIP), University of Ghana, Volta Road, Legon-Accra, Ghana	Collins M. Morang'a et al
EPI_ISL_1255197	West African Centre for Cell Biology of Infectious Pathogens (WACCBIP), University of Ghana, Accra, Ghana	West African Centre for Cell Biology of Infectious Pathogens (WACCBIP), University of Ghana, Volta Road, Legon-Accra, Ghana	Collins M. Morang'a et al
EPI_ISL_1255198	West African Centre for Cell Biology of Infectious Pathogens (WACCBIP), University of Ghana, Accra, Ghana	West African Centre for Cell Biology of Infectious Pathogens (WACCBIP), University of Ghana, Volta Road, Legon-Accra, Ghana	Collins M. Morang'a et al
EPI_ISL_1255199	West African Centre for Cell Biology of Infectious Pathogens (WACCBIP), University of Ghana, Accra, Ghana	West African Centre for Cell Biology of Infectious Pathogens (WACCBIP), University of Ghana, Volta Road, Legon-Accra, Ghana	Collins M. Morang'a et al
EPI_ISL_1255211	West African Centre for Cell Biology of Infectious Pathogens (WACCBIP), University of Ghana, Accra, Ghana	West African Centre for Cell Biology of Infectious Pathogens (WACCBIP), University of Ghana, Volta Road, Legon-Accra, Ghana	Collins M. Morang'a et al
EPI_ISL_1255231	West African Centre for Cell Biology of Infectious Pathogens (WACCBIP), University of Ghana, Accra, Ghana	West African Centre for Cell Biology of Infectious Pathogens (WACCBIP), University of Ghana, Volta Road, Legon-Accra, Ghana	Collins M. Morang'a et al
EPI_ISL_1255233	West African Centre for Cell Biology of Infectious Pathogens (WACCBIP), University of Ghana, Accra, Ghana	West African Centre for Cell Biology of Infectious Pathogens (WACCBIP), University of Ghana, Volta Road, Legon-Accra, Ghana	Collins M. Morang'a et al
EPI_ISL_1255234	West African Centre for Cell Biology of Infectious Pathogens (WACCBIP), University of Ghana, Accra, Ghana	West African Centre for Cell Biology of Infectious Pathogens (WACCBIP), University of Ghana, Volta Road, Legon-Accra, Ghana	Collins M. Morang'a et al
EPI_ISL_1255240	West African Centre for Cell Biology of Infectious Pathogens (WACCBIP), University of Ghana, Accra, Ghana	West African Centre for Cell Biology of Infectious Pathogens (WACCBIP), University of Ghana, Volta Road, Legon-Accra, Ghana	Collins M. Morang'a et al
EPI_ISL_1255241	West African Centre for Cell Biology of Infectious Pathogens (WACCBIP), University of Ghana, Accra, Ghana	West African Centre for Cell Biology of Infectious Pathogens (WACCBIP), University of Ghana, Volta Road, Legon-Accra, Ghana	Collins M. Morang'a et al
EPI_ISL_1255243	West African Centre for Cell Biology of Infectious Pathogens (WACCBIP), University of Ghana, Accra, Ghana	West African Centre for Cell Biology of Infectious Pathogens (WACCBIP), University of Ghana, Volta Road, Legon-Accra, Ghana	Collins M. Morang'a et al
EPI_ISL_1255250	West African Centre for Cell Biology of Infectious Pathogens (WACCBIP), University of Ghana, Accra, Ghana	West African Centre for Cell Biology of Infectious Pathogens (WACCBIP), University of Ghana, Volta Road, Legon-Accra, Ghana	Collins M. Morang'a et al
EPI_ISL_1255256	West African Centre for Cell Biology of Infectious Pathogens (WACCBIP), University of Ghana, Accra, Ghana	West African Centre for Cell Biology of Infectious Pathogens (WACCBIP), University of Ghana, Volta Road, Legon-Accra, Ghana	Collins M. Morang'a et al
EPI_ISL_1255257	West African Centre for Cell Biology of Infectious Pathogens (WACCBIP), University of Ghana, Accra, Ghana	West African Centre for Cell Biology of Infectious Pathogens (WACCBIP), University of Ghana, Volta Road, Legon-Accra, Ghana	Collins M. Morang'a et al

EPI_ISL_1255258	West African Centre for Cell Biology of Infectious Pathogens (WACCBIP), University of Ghana, Accra, Ghana	West African Centre for Cell Biology of Infectious Pathogens (WACCBIP), University of Ghana, Volta Road, Legon-Accra, Ghana	Collins M. Morang'a et al
EPI_ISL_1255264	West African Centre for Cell Biology of Infectious Pathogens (WACCBIP), University of Ghana, Accra, Ghana	West African Centre for Cell Biology of Infectious Pathogens (WACCBIP), University of Ghana, Volta Road, Legon-Accra, Ghana	Collins M. Morang'a et al
EPI_ISL_1255267	West African Centre for Cell Biology of Infectious Pathogens (WACCBIP), University of Ghana, Accra, Ghana	West African Centre for Cell Biology of Infectious Pathogens (WACCBIP), University of Ghana, Volta Road, Legon-Accra, Ghana	Collins M. Morang'a et al
EPI_ISL_1255270	West African Centre for Cell Biology of Infectious Pathogens (WACCBIP), University of Ghana, Accra, Ghana	West African Centre for Cell Biology of Infectious Pathogens (WACCBIP), University of Ghana, Volta Road, Legon-Accra, Ghana	Collins M. Morang'a et al
EPI_ISL_448951	Regional Virus Laboratory, Belfast Health and Social Care Trust	COVID-19 Genomics UK (COG-UK) Consortium	Conall McCaughey et al
EPI_ISL_1662047	Centro de Investigacion Biomedica del Noreste (CIBIN)	Unidad de Genomica Avanzada	Consorcio Mexicano de Vigilancia Genomic
EPI_ISL_1662101	Centro de Investigacion Biomedica de Occidente (CIBO)	Unidad de Genomica Avanzada	Consorcio Mexicano de Vigilancia Genomic
EPI_ISL_1279276	Centro de Investigación Biomédica del Noreste (CIBIN)	Instituto Nacional de Enfermedades Respiratorias (INER): Centro de Investigación en Enfermedades Infecciosas (CIENI)	Consorcio Mexicano de Vigilancia Genómic
EPI_ISL_1416455	Laboratorio Central de Epidemiología (LCE)	Instituto de Biotecnología de la UNAM	Consorcio Mexicano de Vigilancia Genómic
EPI_ISL_522686	Royal Hobart Hospital Microbiology Department	MDU-PHL	Cooley L. et al
EPI_ISL_1663229	National Institute of Infectious Diseases-Prof. Dr. Matei Bals Molecular Diagnostics Laboratory	National Institute of Infectious Diseases-Prof. Dr. Matei Bals Molecular Diagnostics Laboratory	Corina Casangiu et al
EPI_ISL_1657093	National Institute of Infectious Diseases-Prof. Dr. Matei Bals Molecular Diagnostics Laboratory	National Institute of Infectious Diseases-Prof. Dr. Matei Bals Molecular Diagnostics Laboratory	Corina Casangiu et al
EPI_ISL_1657104	Department of Public Health Bucharest	National Institute of Infectious Diseases-Prof. Dr. Matei Bals Molecular Diagnostics Laboratory	Corina Casangiu et al
EPI_ISL_1657105	Department of Public Health Bucharest	National Institute of Infectious Diseases-Prof. Dr. Matei Bals Molecular Diagnostics Laboratory	Corina Casangiu et al
EPI_ISL_1657100	National Institute of Infectious Diseases-Prof. Dr. Matei Bals Molecular Diagnostics Laboratory	National Institute of Infectious Diseases-Prof. Dr. Matei Bals Molecular Diagnostics Laboratory	Corina Casangiu et al
EPI_ISL_1517387	Inciensa, Instituto Costarricense de Investigación y Enseñanza en Nutrición y Salud	Inciensa, Instituto Costarricense de Investigación y Enseñanza en Nutrición y Salud	Cristian Peréz-Corrales et al
EPI_ISL_1517392	Inciensa, Instituto Costarricense de Investigación y Enseñanza en Nutrición y Salud	Inciensa, Instituto Costarricense de Investigación y Enseñanza en Nutrición y Salud	Cristian Peréz-Corrales et al
EPI_ISL_1517395	Inciensa, Instituto Costarricense de Investigación y Enseñanza en Nutrición y Salud	Inciensa, Instituto Costarricense de Investigación y Enseñanza en Nutrición y Salud	Cristian Peréz-Corrales et al
EPI_ISL_1517400	Inciensa, Instituto Costarricense de Investigación y Enseñanza en Nutrición y Salud	Inciensa, Instituto Costarricense de Investigación y Enseñanza en Nutrición y Salud	Cristian Peréz-Corrales et al
EPI_ISL_1517403	Inciensa, Instituto Costarricense de Investigación y Enseñanza en Nutrición y Salud	Inciensa, Instituto Costarricense de Investigación y Enseñanza en Nutrición y Salud	Cristian Peréz-Corrales et al
EPI_ISL_1517411	Inciensa, Instituto Costarricense de Investigación y Enseñanza en Nutrición y Salud	Inciensa, Instituto Costarricense de Investigación y Enseñanza en Nutrición y Salud	Cristian Peréz-Corrales et al
EPI_ISL_1517432	Inciensa, Instituto Costarricense de Investigación y Enseñanza en Nutrición y Salud	Inciensa, Instituto Costarricense de Investigación y Enseñanza en Nutrición y Salud	Cristian Peréz-Corrales et al
EPI_ISL_1517433	Inciensa, Instituto Costarricense de Investigación y Enseñanza en Nutrición y Salud	Inciensa, Instituto Costarricense de Investigación y Enseñanza en Nutrición y Salud	Cristian Peréz-Corrales et al
EPI_ISL_824483	Hospital Universitari Vall d'Hebron - Vall d'Hebron Institut de Recerca	Hospital Universitari Vall d'Hebron - Vall d'Hebron Institut de Recerca	Cristina Andrés et al

a (CoViGen-Mex). Authors (in alphabetical [illegible]

a (CoViGen-Mex). Authors (in alphabetical [illegible]

a (CoViGen-Mex). Authors (in alphabetical [illegible]

a (CoViGen-Mex). Authors (in alphabetical [illegible]

EPI_ISL_1116556	Hospital Universitari Vall d'Hebron - Vall d'Hebron Institut de Recerca	Hospital Universitari Vall d'Hebron - Vall d'Hebron Institut de Recerca	Cristina Andrés et al
EPI_ISL_824425	Hospital Universitari Vall d'Hebron - Vall d'Hebron Institut de Recerca	Hospital Universitari Vall d'Hebron - Vall d'Hebron Institut de Recerca	Cristina Andrés et al
EPI_ISL_824490	Hospital Universitari Vall d'Hebron - Vall d'Hebron Institut de Recerca	Hospital Universitari Vall d'Hebron - Vall d'Hebron Institut de Recerca	Cristina Andrés et al
EPI_ISL_1058178	Hospital Universitari Arnau de Vilanova	Hospital Universitari Vall d'Hebron - Vall d'Hebron Institut de Recerca	Cristina Andrés et al
EPI_ISL_824481	Hospital Universitari Vall d'Hebron - Vall d'Hebron Institut de Recerca	Hospital Universitari Vall d'Hebron - Vall d'Hebron Institut de Recerca	Cristina Andrés et al
EPI_ISL_445171	UCSF Clinical Microbiology Laboratory	Chan-Zuckerberg Biohub	CZB Cliahub Consortium et al
EPI_ISL_445177	UCSF Clinical Microbiology Laboratory	Chan-Zuckerberg Biohub	CZB Cliahub Consortium et al
EPI_ISL_445179	UCSF Clinical Microbiology Laboratory	Chan-Zuckerberg Biohub	CZB Cliahub Consortium et al
EPI_ISL_739361	Alameda County Public Health Lab	Chan-Zuckerberg Biohub	CZB Cliahub Consortium et al
EPI_ISL_1093065	Santa Clara County Public Health Laboratory	Chan-Zuckerberg Biohub	CZB Cliahub Consortium et al
EPI_ISL_582914	County of Santa Clara Public Health Department	Chan-Zuckerberg Biohub	CZB Cliahub Consortium et al
EPI_ISL_1648355	Quest Diagnostics Incorporated	Centers for Disease Control and Prevention Division of Viral Diseases, Pathogen Discovery	Dakota Howard et al
EPI_ISL_1649154	Aegis Sciences Corporation	Centers for Disease Control and Prevention Division of Viral Diseases, Pathogen Discovery	Dakota Howard et al
EPI_ISL_476496	Hospital Garrahan	Héritas	Dalmacio Pereyra et al
EPI_ISL_1357603	Centre For Biotechnology Research And Development	THE AFRICA GENOMICS CENTRE AND CONSULTANCY	Damaris Matoke-Muhia et al
EPI_ISL_760034	Hong Kong Department of Health	School of Public Health, The University of Hong Kong	Daniel Chu et al
EPI_ISL_1586072	The National University Hospital of Iceland	deCODE genetics	Daniel F Gudbjartsson et al
EPI_ISL_417765	The National University Hospital of Iceland	deCODE genetics	Daniel F Gudbjartsson et al
EPI_ISL_1586507	deCODE genetics	deCODE genetics	Daniel F Gudbjartsson et al
EPI_ISL_1586817	deCODE genetics	deCODE genetics	Daniel F Gudbjartsson et al
EPI_ISL_1586864	The National University Hospital of Iceland	deCODE genetics	Daniel F Gudbjartsson et al
EPI_ISL_1586118	The National University Hospital of Iceland	deCODE genetics	Daniel F Gudbjartsson et al
EPI_ISL_1586099	deCODE genetics	deCODE genetics	Daniel F Gudbjartsson et al
EPI_ISL_417554	The National University Hospital of Iceland	deCODE genetics	Daniel F Gudbjartsson et al
EPI_ISL_826728	deCODE genetics	deCODE genetics	Daniel F Gudbjartsson et al
EPI_ISL_826731	deCODE genetics	deCODE genetics	Daniel F Gudbjartsson et al
EPI_ISL_826896	deCODE genetics	deCODE genetics	Daniel F Gudbjartsson et al
EPI_ISL_826918	deCODE genetics	deCODE genetics	Daniel F Gudbjartsson et al
EPI_ISL_827051	The National University Hospital of Iceland	deCODE genetics	Daniel F Gudbjartsson et al
EPI_ISL_827128	deCODE genetics	deCODE genetics	Daniel F Gudbjartsson et al

EPI_ISL_827202	The National University Hospital of Iceland	deCODE genetics	Daniel F Gudbjartsson et al
EPI_ISL_827280	deCODE genetics	deCODE genetics	Daniel F Gudbjartsson et al
EPI_ISL_827382	deCODE genetics	deCODE genetics	Daniel F Gudbjartsson et al
EPI_ISL_827862	deCODE genetics	deCODE genetics	Daniel F Gudbjartsson et al
EPI_ISL_827924	deCODE genetics	deCODE genetics	Daniel F Gudbjartsson et al
EPI_ISL_828009	The National University Hospital of Iceland	deCODE genetics	Daniel F Gudbjartsson et al
EPI_ISL_828071	deCODE genetics	deCODE genetics	Daniel F Gudbjartsson et al
EPI_ISL_828215	The National University Hospital of Iceland	deCODE genetics	Daniel F Gudbjartsson et al
EPI_ISL_828262	deCODE genetics	deCODE genetics	Daniel F Gudbjartsson et al
EPI_ISL_828618	The National University Hospital of Iceland	deCODE genetics	Daniel F Gudbjartsson et al
EPI_ISL_828826	The National University Hospital of Iceland	deCODE genetics	Daniel F Gudbjartsson et al
EPI_ISL_424544	deCODE genetics	deCODE genetics	Daniel F Gudbjartsson et al
EPI_ISL_829138	deCODE genetics	deCODE genetics	Daniel F Gudbjartsson et al
EPI_ISL_829198	deCODE genetics	deCODE genetics	Daniel F Gudbjartsson et al
EPI_ISL_829202	deCODE genetics	deCODE genetics	Daniel F Gudbjartsson et al
EPI_ISL_829731	deCODE genetics	deCODE genetics	Daniel F Gudbjartsson et al
EPI_ISL_829740	deCODE genetics	deCODE genetics	Daniel F Gudbjartsson et al
EPI_ISL_829777	deCODE genetics	deCODE genetics	Daniel F Gudbjartsson et al
EPI_ISL_829891	The National University Hospital of Iceland	deCODE genetics	Daniel F Gudbjartsson et al
EPI_ISL_830189	deCODE genetics	deCODE genetics	Daniel F Gudbjartsson et al
EPI_ISL_830209	The National University Hospital of Iceland	deCODE genetics	Daniel F Gudbjartsson et al
EPI_ISL_830398	deCODE genetics	deCODE genetics	Daniel F Gudbjartsson et al
EPI_ISL_830529	The National University Hospital of Iceland	deCODE genetics	Daniel F Gudbjartsson et al
EPI_ISL_1586696	The National University Hospital of Iceland	deCODE genetics	Daniel F Gudbjartsson et al
EPI_ISL_414378	National Centre for Infectious Diseases	Programme in Emerging Infectious Diseases, Duke-NUS Medical School	Danielle E Anderson et al
EPI_ISL_410535	National Centre for Infectious Diseases	Programme in Emerging Infectious Diseases, Duke-NUS Medical School	Danielle E Anderson et al
EPI_ISL_496605	Gorgas Memorial Laboratory of Health Studies	Gorgas Memorial Laboratory of Health Studies	Danilo Franco et al
EPI_ISL_496606	Gorgas Memorial Laboratory of Health Studies	Gorgas Memorial Laboratory of Health Studies	Danilo Franco et al
EPI_ISL_496607	Gorgas Memorial Laboratory of Health Studies	Gorgas Memorial Laboratory of Health Studies	Danilo Franco et al
EPI_ISL_496609	Gorgas Memorial Laboratory of Health Studies	Gorgas Memorial Laboratory of Health Studies	Danilo Franco et al

EPI_ISL_496615	Gorgas Memorial Laboratory of Health Studies	Gorgas Memorial Laboratory of Health Studies	Danilo Franco et al
EPI_ISL_496649	Gorgas Memorial Laboratory of Health Studies	Gorgas Memorial Laboratory of Health Studies	Danilo Franco et al
EPI_ISL_496685	Gorgas Memorial Laboratory of Health Studies	Gorgas Memorial Laboratory of Health Studies	Danilo Franco et al
EPI_ISL_496736	Gorgas Memorial Laboratory of Health Studies	Gorgas Memorial Laboratory of Health Studies	Danilo Franco et al
EPI_ISL_496771	Gorgas Memorial Laboratory of Health Studies	Gorgas Memorial Laboratory of Health Studies	Danilo Franco et al
EPI_ISL_496776	Gorgas Memorial Laboratory of Health Studies	Gorgas Memorial Laboratory of Health Studies	Danilo Franco et al
EPI_ISL_496785	Gorgas Memorial Laboratory of Health Studies	Gorgas Memorial Laboratory of Health Studies	Danilo Franco et al
EPI_ISL_496799	Gorgas Memorial Laboratory of Health Studies	Gorgas Memorial Laboratory of Health Studies	Danilo Franco et al
EPI_ISL_496804	Gorgas Memorial Laboratory of Health Studies	Gorgas Memorial Laboratory of Health Studies	Danilo Franco et al
EPI_ISL_496825	Gorgas Memorial Laboratory of Health Studies	Gorgas Memorial Laboratory of Health Studies	Danilo Franco et al
EPI_ISL_496832	Gorgas Memorial Laboratory of Health Studies	Gorgas Memorial Laboratory of Health Studies	Danilo Franco et al
EPI_ISL_496836	Gorgas Memorial Laboratory of Health Studies	Gorgas Memorial Laboratory of Health Studies	Danilo Franco et al
EPI_ISL_613544	Department of Virus and Microbiological Special Diagnostics, Statens Serum Institut, Denmark	Albertsen lab, Department of Chemistry and Bioscience, Aalborg University, Denmark	Danish Corona Genome Consortia et al
EPI_ISL_617943	Department of Virus and Microbiological Special Diagnostics, Statens Serum Institut, Denmark	Albertsen lab, Department of Chemistry and Bioscience, Aalborg University, Denmark	Danish Covid-19 Genome Consortia et al
EPI_ISL_622420	Department of Virus and Microbiological Special Diagnostics, Statens Serum Institut, Denmark	Albertsen lab, Department of Chemistry and Bioscience, Aalborg University, Denmark	Danish Covid-19 Genome Consortia et al
EPI_ISL_618102	Department of Virus and Microbiological Special Diagnostics, Statens Serum Institut, Denmark	Albertsen lab, Department of Chemistry and Bioscience, Aalborg University, Denmark	Danish Covid-19 Genome Consortia et al
EPI_ISL_617982	Department of Virus and Microbiological Special Diagnostics, Statens Serum Institut, Denmark	Albertsen lab, Department of Chemistry and Bioscience, Aalborg University, Denmark	Danish Covid-19 Genome Consortia et al
EPI_ISL_618127	Department of Virus and Microbiological Special Diagnostics, Statens Serum Institut, Denmark	Albertsen lab, Department of Chemistry and Bioscience, Aalborg University, Denmark	Danish Covid-19 Genome Consortia et al
EPI_ISL_618137	Department of Virus and Microbiological Special Diagnostics, Statens Serum Institut, Denmark	Albertsen lab, Department of Chemistry and Bioscience, Aalborg University, Denmark	Danish Covid-19 Genome Consortia et al
EPI_ISL_618138	Department of Virus and Microbiological Special Diagnostics, Statens Serum Institut, Denmark	Albertsen lab, Department of Chemistry and Bioscience, Aalborg University, Denmark	Danish Covid-19 Genome Consortia et al
EPI_ISL_619318	Department of Virus and Microbiological Special Diagnostics, Statens Serum Institut, Denmark	Albertsen lab, Department of Chemistry and Bioscience, Aalborg University, Denmark	Danish Covid-19 Genome Consortia et al
EPI_ISL_617946	Department of Virus and Microbiological Special Diagnostics, Statens Serum Institut, Denmark	Albertsen lab, Department of Chemistry and Bioscience, Aalborg University, Denmark	Danish Covid-19 Genome Consortia et al
EPI_ISL_618212	Department of Virus and Microbiological Special Diagnostics, Statens Serum Institut, Denmark	Albertsen lab, Department of Chemistry and Bioscience, Aalborg University, Denmark	Danish Covid-19 Genome Consortia et al
EPI_ISL_622515	Department of Virus and Microbiological Special Diagnostics, Statens Serum Institut, Denmark	Albertsen lab, Department of Chemistry and Bioscience, Aalborg University, Denmark	Danish Covid-19 Genome Consortia et al
EPI_ISL_622531	Department of Virus and Microbiological Special Diagnostics, Statens Serum Institut, Denmark	Albertsen lab, Department of Chemistry and Bioscience, Aalborg University, Denmark	Danish Covid-19 Genome Consortia et al

EPI_ISL_618328	Department of Virus and Microbiological Special Diagnostics, Statens Serum Institut, Denmark	Albertsen lab, Department of Chemistry and Bioscience, Aalborg University, Denmark	Danish Covid-19 Genome Consortia et al
EPI_ISL_618323	Department of Virus and Microbiological Special Diagnostics, Statens Serum Institut, Denmark	Albertsen lab, Department of Chemistry and Bioscience, Aalborg University, Denmark	Danish Covid-19 Genome Consortia et al
EPI_ISL_618223	Department of Virus and Microbiological Special Diagnostics, Statens Serum Institut, Denmark	Albertsen lab, Department of Chemistry and Bioscience, Aalborg University, Denmark	Danish Covid-19 Genome Consortia et al
EPI_ISL_619321	Department of Virus and Microbiological Special Diagnostics, Statens Serum Institut, Denmark	Albertsen lab, Department of Chemistry and Bioscience, Aalborg University, Denmark	Danish Covid-19 Genome Consortia et al
EPI_ISL_617658	Department of Virus and Microbiological Special Diagnostics, Statens Serum Institut, Denmark	Albertsen lab, Department of Chemistry and Bioscience, Aalborg University, Denmark	Danish Covid-19 Genome Consortia et al
EPI_ISL_617424	Department of Virus and Microbiological Special Diagnostics, Statens Serum Institut, Denmark	Albertsen lab, Department of Chemistry and Bioscience, Aalborg University, Denmark	Danish Covid-19 Genome Consortia et al
EPI_ISL_616408	Department of Virus and Microbiological Special Diagnostics, Statens Serum Institut, Denmark	Albertsen lab, Department of Chemistry and Bioscience, Aalborg University, Denmark	Danish Covid-19 Genome Consortia et al
EPI_ISL_622643	Department of Virus and Microbiological Special Diagnostics, Statens Serum Institut, Denmark	Albertsen lab, Department of Chemistry and Bioscience, Aalborg University, Denmark	Danish Covid-19 Genome Consortia et al
EPI_ISL_622733	Department of Virus and Microbiological Special Diagnostics, Statens Serum Institut, Denmark	Albertsen lab, Department of Chemistry and Bioscience, Aalborg University, Denmark	Danish Covid-19 Genome Consortia et al
EPI_ISL_622356	Department of Virus and Microbiological Special Diagnostics, Statens Serum Institut, Denmark	Albertsen lab, Department of Chemistry and Bioscience, Aalborg University, Denmark	Danish Covid-19 Genome Consortia et al
EPI_ISL_619715	Department of Virus and Microbiological Special Diagnostics, Statens Serum Institut, Denmark	Albertsen lab, Department of Chemistry and Bioscience, Aalborg University, Denmark	Danish Covid-19 Genome Consortia et al
EPI_ISL_619846	Department of Virus and Microbiological Special Diagnostics, Statens Serum Institut, Denmark	Albertsen lab, Department of Chemistry and Bioscience, Aalborg University, Denmark	Danish Covid-19 Genome Consortia et al
EPI_ISL_619301	Department of Virus and Microbiological Special Diagnostics, Statens Serum Institut, Denmark	Albertsen lab, Department of Chemistry and Bioscience, Aalborg University, Denmark	Danish Covid-19 Genome Consortia et al
EPI_ISL_619291	Department of Virus and Microbiological Special Diagnostics, Statens Serum Institut, Denmark	Albertsen lab, Department of Chemistry and Bioscience, Aalborg University, Denmark	Danish Covid-19 Genome Consortia et al
EPI_ISL_619393	Department of Virus and Microbiological Special Diagnostics, Statens Serum Institut, Denmark	Albertsen lab, Department of Chemistry and Bioscience, Aalborg University, Denmark	Danish Covid-19 Genome Consortia et al
EPI_ISL_614421	Department of Virus and Microbiological Special Diagnostics, Statens Serum Institut, Denmark	Albertsen lab, Department of Chemistry and Bioscience, Aalborg University, Denmark	Danish Covid-19 Genome Consortia et al
EPI_ISL_614754	Department of Virus and Microbiological Special Diagnostics, Statens Serum Institut, Denmark	Albertsen lab, Department of Chemistry and Bioscience, Aalborg University, Denmark	Danish Covid-19 Genome Consortia et al
EPI_ISL_614815	Department of Virus and Microbiological Special Diagnostics, Statens Serum Institut, Denmark	Albertsen lab, Department of Chemistry and Bioscience, Aalborg University, Denmark	Danish Covid-19 Genome Consortia et al
EPI_ISL_614799	Department of Virus and Microbiological Special Diagnostics, Statens Serum Institut, Denmark	Albertsen lab, Department of Chemistry and Bioscience, Aalborg University, Denmark	Danish Covid-19 Genome Consortia et al
EPI_ISL_614426	Department of Virus and Microbiological Special Diagnostics, Statens Serum Institut, Denmark	Albertsen lab, Department of Chemistry and Bioscience, Aalborg University, Denmark	Danish Covid-19 Genome Consortia et al
EPI_ISL_614424	Department of Virus and Microbiological Special Diagnostics, Statens Serum Institut, Denmark	Albertsen lab, Department of Chemistry and Bioscience, Aalborg University, Denmark	Danish Covid-19 Genome Consortia et al
EPI_ISL_614753	Department of Virus and Microbiological Special Diagnostics, Statens Serum Institut, Denmark	Albertsen lab, Department of Chemistry and Bioscience, Aalborg University, Denmark	Danish Covid-19 Genome Consortia et al

EPI_ISL_614411	Department of Virus and Microbiological Special Diagnostics, Statens Serum Institut, Denmark	Albertsen lab, Department of Chemistry and Bioscience, Aalborg University, Denmark	Danish Covid-19 Genome Consortia et al
EPI_ISL_614802	Department of Virus and Microbiological Special Diagnostics, Statens Serum Institut, Denmark	Albertsen lab, Department of Chemistry and Bioscience, Aalborg University, Denmark	Danish Covid-19 Genome Consortia et al
EPI_ISL_670281	Department of Virus and Microbiological Special Diagnostics, Statens Serum Institut, Copenhagen, Denmark	Albertsen Lab, Department of Chemistry and Bioscience, Aalborg University, Denmark	Danish Covid-19 Genome Consortium et al
EPI_ISL_670299	Department of Virus and Microbiological Special Diagnostics, Statens Serum Institut, Copenhagen, Denmark	Albertsen Lab, Department of Chemistry and Bioscience, Aalborg University, Denmark	Danish Covid-19 Genome Consortium et al
EPI_ISL_682605	Department of Virus and Microbiological Special Diagnostics, Statens Serum Institut, Copenhagen, Denmark	Albertsen Lab, Department of Chemistry and Bioscience, Aalborg University, Denmark	Danish Covid-19 Genome Consortium et al
EPI_ISL_845385	Department of Virus and Microbiological Special Diagnostics, Statens Serum Institut, Copenhagen, Denmark	Albertsen Lab, Department of Chemistry and Bioscience, Aalborg University, Denmark	Danish Covid-19 Genome Consortium et al
EPI_ISL_847449	Department of Virus and Microbiological Special Diagnostics, Statens Serum Institut, Copenhagen, Denmark	Albertsen Lab, Department of Chemistry and Bioscience, Aalborg University, Denmark	Danish Covid-19 Genome Consortium et al
EPI_ISL_926776	Department of Virus and Microbiological Special Diagnostics, Statens Serum Institut, Copenhagen, Denmark	Aalborg University	Danish Covid-19 Genome Consortium et al
EPI_ISL_927497	Department of Virus and Microbiological Special Diagnostics, Statens Serum Institut, Copenhagen, Denmark	Aalborg University	Danish Covid-19 Genome Consortium et al
EPI_ISL_971627	Department of Virus and Microbiological Special Diagnostics, Statens Serum Institut, Copenhagen, Denmark	Aalborg University	Danish Covid-19 Genome Consortium et al
EPI_ISL_973071	Department of Virus and Microbiological Special Diagnostics, Statens Serum Institut, Copenhagen, Denmark	Aalborg University	Danish Covid-19 Genome Consortium et al
EPI_ISL_973216	Department of Virus and Microbiological Special Diagnostics, Statens Serum Institut, Copenhagen, Denmark	Aalborg University	Danish Covid-19 Genome Consortium et al
EPI_ISL_1023986	Department of Virus and Microbiological Special Diagnostics, Statens Serum Institut, Copenhagen, Denmark	Aalborg University	Danish Covid-19 Genome Consortium et al
EPI_ISL_1024655	Department of Virus and Microbiological Special Diagnostics, Statens Serum Institut, Copenhagen, Denmark	Aalborg University	Danish Covid-19 Genome Consortium et al
EPI_ISL_1065177	Department of Virus and Microbiological Special Diagnostics, Statens Serum Institut, Copenhagen, Denmark	Aalborg University	Danish Covid-19 Genome Consortium et al
EPI_ISL_1066314	Department of Virus and Microbiological Special Diagnostics, Statens Serum Institut, Copenhagen, Denmark	Aalborg University	Danish Covid-19 Genome Consortium et al
EPI_ISL_654194	Hospital General Universitario Gregorio Marañón	SeqCOVID-SPAIN consortium/IBV(CSIC)	Darío García de Viedma et al
EPI_ISL_654170	Hospital General Universitario Gregorio Marañón	SeqCOVID-SPAIN consortium/IBV(CSIC)	Darío García de Viedma et al
EPI_ISL_813997	Hospital General Universitario Gregorio Marañón	SeqCOVID-SPAIN consortium/IBV(CSIC)	Darío García de Viedma et al
EPI_ISL_852976	Hospital General Universitario Gregorio Marañón	SeqCOVID-SPAIN consortium/IBV(CSIC)	Darío García de Viedma et al
EPI_ISL_610275	New Mexico Department of Health Scientific Laboratory Division	Center for Global Health, University of New Mexico Health Sciences Center	Daryl Domman et al
EPI_ISL_610278	New Mexico Department of Health Scientific Laboratory Division	Center for Global Health, University of New Mexico Health Sciences Center	Daryl Domman et al
EPI_ISL_1181310	MVZ Dr. Eberhard & Partner Dortmund	Bielefeld University	David Brandt et al
EPI_ISL_654496	Servicio de Microbiología. Hospital Clínico Universitario de Valencia	SeqCOVID-SPAIN consortium/IBV(CSIC)	David Navarro Ortega et al

EPI_ISL_413593	Laboratoire National de Santé	Erasmus Medical Center	David Nieuwenhuijse et al
EPI_ISL_413570	RIVM	Erasmus Medical Center	David Nieuwenhuijse et al
EPI_ISL_413574	MHC West-Brabant	Erasmus Medical Center	David Nieuwenhuijse et al
EPI_ISL_414546	Dutch COVID-19 response team	Erasmus Medical Center	David Nieuwenhuijse et al
EPI_ISL_413586	Foundation Elisabeth-Tweesteden Ziekenhuis	Erasmus Medical Center	David Nieuwenhuijse et al
EPI_ISL_718146	Ministry of Health Hospitals	Institute of Health and Community Medicine	David Perera et al
EPI_ISL_718143	Ministry of Health Hospitals	Institute of Health and Community Medicine	David Perera et al
EPI_ISL_718214	Ministry of Health Hospitals	Institute of Health and Community Medicine	David Perera et al
EPI_ISL_718213	Ministry of Health Hospitals	Institute of Health and Community Medicine	David Perera et al
EPI_ISL_718168	Ministry of Health Hospitals	Institute of Health and Community Medicine	David Perera et al
EPI_ISL_718169	Ministry of Health Hospitals	Institute of Health and Community Medicine	David Perera et al
EPI_ISL_718170	Ministry of Health Hospitals	Institute of Health and Community Medicine	David Perera et al
EPI_ISL_804016	Facultad de Medicina, Universidad de Atacama	Facultad de Ciencias de la Vida, UNAB	Dayán Sanhueza et al
EPI_ISL_676519	Klinsisk mikrobiologi Linkoping	The Public Health Agency of Sweden	Department of Microbiology et al
EPI_ISL_676520	Klinisk mikrobiologi	The Public Health Agency of Sweden	Department of Microbiology et al
EPI_ISL_676528	Uppsala klinisk mikrobiologi	The Public Health Agency of Sweden	Department of Microbiology et al
EPI_ISL_710577	Vardcentralen Brinken	The Public Health Agency of Sweden	Department of Microbiology et al
EPI_ISL_766625	Klinisk mikrobiologi	The Public Health Agency of Sweden	Department of Microbiology et al
EPI_ISL_766628	Klinisk mikrobiologi	The Public Health Agency of Sweden	Department of Microbiology et al
EPI_ISL_491933	Centro de Investigaciones, Universidad de Especialidades Espíritu Santo	Institute of Microbiology, Universidad San Francisco de Quito	Derly Andrade et al
EPI_ISL_491935	Centro de Investigaciones, Universidad de Especialidades Espíritu Santo	Institute of Microbiology, Universidad San Francisco de Quito	Derly Andrade et al
EPI_ISL_491941	Centro de Investigaciones, Universidad de Especialidades Espíritu Santo	Institute of Microbiology, Universidad San Francisco de Quito	Derly Andrade et al
EPI_ISL_527819	Centro de Investigaciones, Universidad de Especialidades Espíritu Santo	Institute of Microbiology, Universidad San Francisco de Quito	Derly Andrade et al
EPI_ISL_1381304	Omics Sciences Laboratory	Omics Sciences Laboratory	Derly Andrade Molina et al
EPI_ISL_1381303	Omics Sciences Laboratory	Omics Sciences Laboratory	Derly Andrade Molina et al
EPI_ISL_1443672	Omics Sciences Laboratory	Omics Sciences Laboratory	Derly Andrade Molina et al
EPI_ISL_1443676	Omics Sciences Laboratory	Omics Sciences Laboratory	Derly Andrade Molina et al
EPI_ISL_1381300	Omics Sciences Laboratory	Omics Sciences Laboratory	Derly Andrade Molina et al
EPI_ISL_1381301	Omics Sciences Laboratory	Omics Sciences Laboratory	Derly Andrade Molina et al
EPI_ISL_1443680	Omics Sciences Laboratory	Omics Sciences Laboratory	Derly Andrade Molina et al

EPI_ISL_1381305	Omics Sciences Laboratory	Omics Sciences Laboratory	Derly Andrade Molina et al
EPI_ISL_1381302	Omics Sciences Laboratory	Omics Sciences Laboratory	Derly Andrade Molina et al
EPI_ISL_1443681	Omics Sciences Laboratory	Omics Sciences Laboratory	Derly Andrade Molina et al
EPI_ISL_1443682	Omics Sciences Laboratory	Omics Sciences Laboratory	Derly Andrade Molina et al
EPI_ISL_1443641	Institute of Microbiology, Universidad San Francisco de Quito	Omics Sciences Laboratory	Derly Andrade Molina et al
EPI_ISL_1443642	Institute of Microbiology, Universidad San Francisco de Quito	Omics Sciences Laboratory	Derly Andrade Molina et al
EPI_ISL_1443643	Institute of Microbiology, Universidad San Francisco de Quito	Omics Sciences Laboratory	Derly Andrade Molina et al
EPI_ISL_1443650	Institute of Microbiology, Universidad San Francisco de Quito	Omics Sciences Laboratory	Derly Andrade Molina et al
EPI_ISL_1443651	Institute of Microbiology, Universidad San Francisco de Quito	Omics Sciences Laboratory	Derly Andrade Molina et al
EPI_ISL_1443652	Institute of Microbiology, Universidad San Francisco de Quito	Omics Sciences Laboratory	Derly Andrade Molina et al
EPI_ISL_1443656	Institute of Microbiology, Universidad San Francisco de Quito	Omics Sciences Laboratory	Derly Andrade Molina et al
EPI_ISL_1443658	Institute of Microbiology, Universidad San Francisco de Quito	Omics Sciences Laboratory	Derly Andrade Molina et al
EPI_ISL_1443664	Institute of Microbiology, Universidad San Francisco de Quito	Omics Sciences Laboratory	Derly Andrade Molina et al
EPI_ISL_1443665	Institute of Microbiology, Universidad San Francisco de Quito	Omics Sciences Laboratory	Derly Andrade Molina et al
EPI_ISL_861914	Genomika Einstein	LATE - Laboratório de Técnicas Especiais - Hospital Israelita Albert Einstein	Deyvid Amgarten et al
EPI_ISL_861868	LATE - Laboratório de Técnicas Especiais - Hospital Israelita Albert Einstein	LATE - Laboratório de Técnicas Especiais - Hospital Israelita Albert Einstein	Deyvid Amgarten et al
EPI_ISL_861870	LATE - Laboratório de Técnicas Especiais - Hospital Israelita Albert Einstein	LATE - Laboratório de Técnicas Especiais - Hospital Israelita Albert Einstein	Deyvid Amgarten et al
EPI_ISL_861871	LATE - Laboratório de Técnicas Especiais - Hospital Israelita Albert Einstein	LATE - Laboratório de Técnicas Especiais - Hospital Israelita Albert Einstein	Deyvid Amgarten et al
EPI_ISL_861873	LATE - Laboratório de Técnicas Especiais - Hospital Israelita Albert Einstein	LATE - Laboratório de Técnicas Especiais - Hospital Israelita Albert Einstein	Deyvid Amgarten et al
EPI_ISL_861875	LATE - Laboratório de Técnicas Especiais - Hospital Israelita Albert Einstein	LATE - Laboratório de Técnicas Especiais - Hospital Israelita Albert Einstein	Deyvid Amgarten et al
EPI_ISL_861876	LATE - Laboratório de Técnicas Especiais - Hospital Israelita Albert Einstein	LATE - Laboratório de Técnicas Especiais - Hospital Israelita Albert Einstein	Deyvid Amgarten et al
EPI_ISL_861878	LATE - Laboratório de Técnicas Especiais - Hospital Israelita Albert Einstein	LATE - Laboratório de Técnicas Especiais - Hospital Israelita Albert Einstein	Deyvid Amgarten et al
EPI_ISL_861881	LATE - Laboratório de Técnicas Especiais - Hospital Israelita Albert Einstein	LATE - Laboratório de Técnicas Especiais - Hospital Israelita Albert Einstein	Deyvid Amgarten et al
EPI_ISL_861884	LATE - Laboratório de Técnicas Especiais - Hospital Israelita Albert Einstein	LATE - Laboratório de Técnicas Especiais - Hospital Israelita Albert Einstein	Deyvid Amgarten et al
EPI_ISL_861885	LATE - Laboratório de Técnicas Especiais - Hospital Israelita Albert Einstein	LATE - Laboratório de Técnicas Especiais - Hospital Israelita Albert Einstein	Deyvid Amgarten et al
EPI_ISL_861886	LATE - Laboratório de Técnicas Especiais - Hospital Israelita Albert Einstein	LATE - Laboratório de Técnicas Especiais - Hospital Israelita Albert Einstein	Deyvid Amgarten et al

EPI_ISL_861888	LATE - Laboratório de Técnicas Especiais - Hospital Israelita Albert Einstein	LATE - Laboratório de Técnicas Especiais - Hospital Israelita Albert Einstein	Deyvid Amgarten et al
EPI_ISL_861891	LATE - Laboratório de Técnicas Especiais - Hospital Israelita Albert Einstein	LATE - Laboratório de Técnicas Especiais - Hospital Israelita Albert Einstein	Deyvid Amgarten et al
EPI_ISL_861892	LATE - Laboratório de Técnicas Especiais - Hospital Israelita Albert Einstein	LATE - Laboratório de Técnicas Especiais - Hospital Israelita Albert Einstein	Deyvid Amgarten et al
EPI_ISL_884250	LATE - Laboratório de Técnicas Especiais - Hospital Israelita Albert Einstein	LATE - Laboratório de Técnicas Especiais - Hospital Israelita Albert Einstein	Deyvid Amgarten et al
EPI_ISL_861895	LATE - Laboratório de Técnicas Especiais - Hospital Israelita Albert Einstein	LATE - Laboratório de Técnicas Especiais - Hospital Israelita Albert Einstein	Deyvid Amgarten et al
EPI_ISL_861896	LATE - Laboratório de Técnicas Especiais - Hospital Israelita Albert Einstein	LATE - Laboratório de Técnicas Especiais - Hospital Israelita Albert Einstein	Deyvid Amgarten et al
EPI_ISL_861897	LATE - Laboratório de Técnicas Especiais - Hospital Israelita Albert Einstein	LATE - Laboratório de Técnicas Especiais - Hospital Israelita Albert Einstein	Deyvid Amgarten et al
EPI_ISL_861901	LATE - Laboratório de Técnicas Especiais - Hospital Israelita Albert Einstein	LATE - Laboratório de Técnicas Especiais - Hospital Israelita Albert Einstein	Deyvid Amgarten et al
EPI_ISL_861902	LATE - Laboratório de Técnicas Especiais - Hospital Israelita Albert Einstein	LATE - Laboratório de Técnicas Especiais - Hospital Israelita Albert Einstein	Deyvid Amgarten et al
EPI_ISL_861903	LATE - Laboratório de Técnicas Especiais - Hospital Israelita Albert Einstein	LATE - Laboratório de Técnicas Especiais - Hospital Israelita Albert Einstein	Deyvid Amgarten et al
EPI_ISL_861905	LATE - Laboratório de Técnicas Especiais - Hospital Israelita Albert Einstein	LATE - Laboratório de Técnicas Especiais - Hospital Israelita Albert Einstein	Deyvid Amgarten et al
EPI_ISL_861908	LATE - Laboratório de Técnicas Especiais - Hospital Israelita Albert Einstein	LATE - Laboratório de Técnicas Especiais - Hospital Israelita Albert Einstein	Deyvid Amgarten et al
EPI_ISL_861909	LATE - Laboratório de Técnicas Especiais - Hospital Israelita Albert Einstein	LATE - Laboratório de Técnicas Especiais - Hospital Israelita Albert Einstein	Deyvid Amgarten et al
EPI_ISL_1544070	National Centre For Cell Science	National Centre For Cell Science – INSACOG	Dhiraj Paul et al
EPI_ISL_1544073	National Centre For Cell Science	National Centre For Cell Science – INSACOG	Dhiraj Paul et al
EPI_ISL_1415196	National Centre For Cell Science	National Centre For Cell Science – INSACOG	Dhiraj Paul et al
EPI_ISL_812967	University Clinical Research Center, University of Sciences	University Clinical Research Center, University of Sciences	Diarra et al
EPI_ISL_812966	University Clinical Research Center, University of Sciences	University Clinical Research Center, University of Sciences	Diarra et al
EPI_ISL_525438	Institute of Microbiology, Universidad San Francisco de Quito	Institute of Microbiology, Universidad San Francisco de Quito	Diego Egas et al
EPI_ISL_1445093	SECRETARIA MUNICIPAL DE SAUDE DE PIRACAIA	Instituto Butantan / Mendelics	Dimas Tadeu Covas et al
EPI_ISL_1445095	SECRETARIA MUNICIPAL DE SAUDE DE PIRACAIA	Instituto Butantan / Mendelics	Dimas Tadeu Covas et al
EPI_ISL_1445102	PRONTO ATENDIMENTO VILA PADRE ANCHIETA	Instituto Butantan / Mendelics	Dimas Tadeu Covas et al
EPI_ISL_1445104	PRONTO ATENDIMENTO VILA PADRE ANCHIETA	Instituto Butantan / Mendelics	Dimas Tadeu Covas et al
EPI_ISL_1445129	UNIDADE RESPIRATORIA NOVA HORTOLANDIA	Instituto Butantan / Mendelics	Dimas Tadeu Covas et al
EPI_ISL_1445136	SMS IPERO	Instituto Butantan / Mendelics	Dimas Tadeu Covas et al

EPI_ISL_1445138	SECRETARIA MUNICIPAL DE SAUDE SOROCABA	Instituto Butantan / Mendelics	Dimas Tadeu Covas et al
EPI_ISL_1445142	CS II EGIDIO BRUNHARA MORRO AGUDO	Instituto Butantan / Mendelics	Dimas Tadeu Covas et al
EPI_ISL_1445157	SECAO CENTRO DE DIAGNOSTICO SECEDI	Instituto Butantan / Mendelics	Dimas Tadeu Covas et al
EPI_ISL_1445159	SECAO CENTRO DE DIAGNOSTICO SECEDI	Instituto Butantan / Mendelics	Dimas Tadeu Covas et al
EPI_ISL_1445212	UBS IRMA AGUEDA MARIA JAIME	Instituto Butantan / Mendelics	Dimas Tadeu Covas et al
EPI_ISL_1445213	UBS OTACILIO FIRMINO LOPES	Instituto Butantan / Mendelics	Dimas Tadeu Covas et al
EPI_ISL_1445214	UBS JOSE HILARIO DOS SANTOS	Instituto Butantan / Mendelics	Dimas Tadeu Covas et al
EPI_ISL_1445217	UBS EMILIA COSME CERQUEIRA	Instituto Butantan / Mendelics	Dimas Tadeu Covas et al
EPI_ISL_1445231	VIGILANCIA EPIDEMIOLOGICA	Instituto Butantan / Mendelics	Dimas Tadeu Covas et al
EPI_ISL_1445232	VIGILANCIA EPIDEMIOLOGICA	Instituto Butantan / Mendelics	Dimas Tadeu Covas et al
EPI_ISL_1445250	VIGILANCIA EPIDEMIOLOGICA	Instituto Butantan / Mendelics	Dimas Tadeu Covas et al
EPI_ISL_1445255	VIGILANCIA EPIDEMIOLOGICA	Instituto Butantan / Mendelics	Dimas Tadeu Covas et al
EPI_ISL_1445260	VIGILANCIA EPIDEMIOLOGICA	Instituto Butantan / Mendelics	Dimas Tadeu Covas et al
EPI_ISL_1445261	VIGILANCIA EPIDEMIOLOGICA	Instituto Butantan / Mendelics	Dimas Tadeu Covas et al
EPI_ISL_1445269	SERV DE VIG SANITARIA EPIDEMIO E CTRL DE ZOONOZES GUARUJA	Instituto Butantan / Mendelics	Dimas Tadeu Covas et al
EPI_ISL_1445271	VIGILANCIA EPIDEMIOLOGICA	Instituto Butantan / Mendelics	Dimas Tadeu Covas et al
EPI_ISL_885143	Central public health laboratory	Molecular Diagnostics Department, Central public health laboratory	Dler et al
EPI_ISL_412028	Hong Kong Department of Health	School of Public Health, The University of Hon g Kong	Dominic N.C. Tsang et al
EPI_ISL_572329	Public Health Authority of the Slovak Republic, Bratislava	Faculty of Natural Sciences, Comenius University in Bratislava	Dominika Fričová et al
EPI_ISL_737200	National Reference Laboratory, Nigeria Centre for Disease Control.	National Reference Laboratory, Nigeria Centre for Disease Control, Gaduwa,Abuja, Nigeria	Dr Ndodo Nnaemeka et al
EPI_ISL_825807	Nigeria Centre For Disease Control	National reference Laboratory, NCDC, Gaduwa, Abuja	Dr Ndodo Nnaemeka et al
EPI_ISL_1073640	Nigeria Centre For Disease Control	National reference Laboratory, NCDC, Gaduwa, Abuja	Dr Ndodo Nnaemeka et al
EPI_ISL_1627088	National reference Laboratory, NCDC, Gaduwa, Abuja, Nigeria	National reference Laboratory, NCDC, Gaduwa, Abuja, Nigeria	Dr Ndodo Nnaemeka et al
EPI_ISL_1197066	Nigeria Centre For Disease Control	National reference Laboratory, NCDC, Gaduwa, Abuja	Dr Ndodo Nnaemeka et al
EPI_ISL_848196	Microbiology Lab	NBCC Sequencing Facility	Dr. Jeff Wrana et al
EPI_ISL_873164	Medical Laboratory Sciences, Arab American University	Medical Laboratory Sciences, Arab American University	Dumaidi et al
EPI_ISL_873165	Medical Laboratory Sciences, Arab American University	Medical Laboratory Sciences, Arab American University	Dumaidi et al
EPI_ISL_792516	Laboratorio Central de la Ciudad de Santa Fe	Grupo de Genómica y Bioinformática del Instituto de Investigación de la Cadena Láctea CONICET-INTA on behalf of 'Proyecto Argentino Interinstitucional de genomica de SARS-CoV-2' (PAIS Consortium)	Eberhardt et al

EPI_ISL_792517	Laboratorio Central de la Ciudad de Santa Fe	Grupo de Genómica y Bioinformática del Instituto de Investigación de la Cadena Láctea CONICET-INTA on behalf of 'Proyecto Argentino Interinstitucional de genomica de SARS-CoV-2' (PAIS Consortium)	Eberhardt et al
EPI_ISL_1395880	Laboratorio Central de la Ciudad de Santa Fe	Grupo de Genómica y Bioinformática del Instituto de Investigación de la Cadena Láctea CONICET-INTA on behalf of 'Proyecto Argentino Interinstitucional de genomica de SARS-CoV-2' (PAIS Consortium)	Eberhardt et al
EPI_ISL_1395886	Laboratorio Central de la Ciudad de Santa Fe	Grupo de Genómica y Bioinformática del Instituto de Investigación de la Cadena Láctea CONICET-INTA on behalf of 'Proyecto Argentino Interinstitucional de genomica de SARS-CoV-2' (PAIS Consortium)	Eberhardt et al
EPI_ISL_626550	Laboratorio de Biología Molecular, Facultad de Medicina, Universidad de Atacama, Copiapo, Chile/ FONDAP CRG, Universidad Andrés Bello, Santiago, Chile	Center for Mathematical Modeling and Center for Genome Regulation. Santiago, Chile	Echeverría C et al
EPI_ISL_626552	Laboratorio de Biología Molecular, Facultad de Medicina, Universidad de Atacama, Copiapo, Chile/ FONDAP CRG, Universidad Andrés Bello, Santiago, Chile	Center for Mathematical Modeling and Center for Genome Regulation. Santiago, Chile	Echeverría C et al
EPI_ISL_417030	Centre for Infectious Diseases and Microbiology Laboratory Services	NSW Health Pathology - Institute of Clinical Pathology and Medical Research; Westmead Hospital; University of Sydney	Eden J-S et al
EPI_ISL_413213	Centre for Infectious Diseases and Microbiology Laboratory Services	NSW Health Pathology - Institute of Clinical Pathology and Medical Research; Westmead Hospital; University of Sydney	Eden J-S et al
EPI_ISL_889008	RS Sentra Medika Cibinong	Eijkman Institute for Molecular Biology, Ministry of Research and Technology/National Agency for Research and Innovation	Edison Johar et al
EPI_ISL_947249	RS Bina Husada Cibinong	Eijkman Institute for Molecular Biology, Ministry of Research and Technology/National Agency for Research and Innovation	Edison Johar et al
EPI_ISL_947252	RS Bina Husada Cibinong	Eijkman Institute for Molecular Biology, Ministry of Research and Technology/National Agency for Research and Innovation	Edison Johar et al
EPI_ISL_536479	Instituto Nacional de Salud	Laboratorio de Infecciones Respiratorias Agudas	Eduardo Juscamayta Lopez et al
EPI_ISL_536506	Instituto Nacional de Salud	Laboratorio de Infecciones Respiratorias Agudas	Eduardo Juscamayta Lopez et al
EPI_ISL_536526	Instituto Nacional de Salud	Laboratorio de Infecciones Respiratorias Agudas	Eduardo Juscamayta Lopez et al
EPI_ISL_536564	Instituto Nacional de Salud	Laboratorio de Infecciones Respiratorias Agudas	Eduardo Juscamayta Lopez et al
EPI_ISL_536485	Instituto Nacional de Salud	Laboratorio de Infecciones Respiratorias Agudas	Eduardo Juscamayta Lopez et al
EPI_ISL_536489	Instituto Nacional de Salud	Laboratorio de Infecciones Respiratorias Agudas	Eduardo Juscamayta Lopez et al
EPI_ISL_536490	Instituto Nacional de Salud	Laboratorio de Infecciones Respiratorias Agudas	Eduardo Juscamayta Lopez et al
EPI_ISL_536497	Instituto Nacional de Salud	Laboratorio de Infecciones Respiratorias Agudas	Eduardo Juscamayta Lopez et al
EPI_ISL_536500	Instituto Nacional de Salud	Laboratorio de Infecciones Respiratorias Agudas	Eduardo Juscamayta Lopez et al
EPI_ISL_536510	Instituto Nacional de Salud	Laboratorio de Infecciones Respiratorias Agudas	Eduardo Juscamayta Lopez et al
EPI_ISL_536511	Instituto Nacional de Salud	Laboratorio de Infecciones Respiratorias Agudas	Eduardo Juscamayta Lopez et al
EPI_ISL_536512	Instituto Nacional de Salud	Laboratorio de Infecciones Respiratorias Agudas	Eduardo Juscamayta Lopez et al
EPI_ISL_536521	Instituto Nacional de Salud	Laboratorio de Infecciones Respiratorias Agudas	Eduardo Juscamayta Lopez et al
EPI_ISL_536535	Instituto Nacional de Salud	Laboratorio de Infecciones Respiratorias Agudas	Eduardo Juscamayta Lopez et al

EPI_ISL_536539	Instituto Nacional de Salud	Laboratorio de Infecciones Respiratorias Agudas	Eduardo Juscamayta Lopez et al
EPI_ISL_536540	Instituto Nacional de Salud	Laboratorio de Infecciones Respiratorias Agudas	Eduardo Juscamayta Lopez et al
EPI_ISL_536541	Instituto Nacional de Salud	Laboratorio de Infecciones Respiratorias Agudas	Eduardo Juscamayta Lopez et al
EPI_ISL_536561	Instituto Nacional de Salud	Laboratorio de Infecciones Respiratorias Agudas	Eduardo Juscamayta Lopez et al
EPI_ISL_794606	Central Public Health Laboratories, Egyptian Ministry of Health and Population	Central Public Health Laboratories, Egyptian Ministry of Health and Population	El-Shesheny et al
EPI_ISL_1138899	Laboratory for HIV and opportunistic infections diagnosis The Republican Research and Practical Center for Epidemiology and Microbiology (RRPCEM)	Laboratory for HIV and opportunistic infections diagnosis The Republican Research and Practical Center for Epidemiology and Microbiology (RRPCEM)	Elena Gasich et al
EPI_ISL_933533	Laboratory for HIV and opportunistic infections diagnosis The Republican Research and Practical Center for Epidemiology and Microbiology (RRPCEM)	Laboratory for HIV and opportunistic infections diagnosis The Republican Research and Practical Center for Epidemiology and Microbiology (RRPCEM)	Elena Gasich et al
EPI_ISL_754229	The Republican Research and Practical Center for Epidemiology and Microbiology (RRPCEM)	WHO National Influenza Centre Russian Federation	Elena Gasich et al
EPI_ISL_754230	The Republican Research and Practical Center for Epidemiology and Microbiology (RRPCEM)	WHO National Influenza Centre Russian Federation	Elena Gasich et al
EPI_ISL_754231	The Republican Research and Practical Center for Epidemiology and Microbiology (RRPCEM)	WHO National Influenza Centre Russian Federation	Elena Gasich et al
EPI_ISL_1209407	Laboratory for HIV and opportunistic infections diagnosis The Republican Research and Practical Center for Epidemiology and Microbiology (RRPCEM)	Laboratory for HIV and opportunistic infections diagnosis The Republican Research and Practical Center for Epidemiology and Microbiology (RRPCEM)	Elena Gasich et al
EPI_ISL_1138983	Laboratory for HIV and opportunistic infections diagnosis The Republican Research and Practical Center for Epidemiology and Microbiology (RRPCEM)	Laboratory for HIV and opportunistic infections diagnosis The Republican Research and Practical Center for Epidemiology and Microbiology (RRPCEM)	Elena Gasich et al
EPI_ISL_1508895	Laboratory for HIV and opportunistic infections diagnosis The Republican Research and Practical Center for Epidemiology and Microbiology (RRPCEM)	Laboratory for HIV and opportunistic infections diagnosis The Republican Research and Practical Center for Epidemiology and Microbiology (RRPCEM)	Elena Gasich et al
EPI_ISL_754234	The Republican Research and Practical Center for Epidemiology and Microbiology (RRPCEM)	WHO National Influenza Centre Russian Federation	Elena Gasich et al
EPI_ISL_1181779	Microbiology and Virology Unit,Azienda Ospedale Padova,Padova,Italy	Department of Molecular Medicine,Computational Medicine Group, Univeresity of Padova,Padova,Italy	Elisa Franchin et al
EPI_ISL_1181797	Microbiology and Virology Unit,Azienda Ospedale Padova,Padova,Italy	Department of Molecular Medicine,Computational Medicine Group, Univeresity of Padova,Padova,Italy	Elisa Franchin et al
EPI_ISL_1181767	Microbiology and Virology Unit,Azienda Ospedale Padova,Padova,Italy	Department of Molecular Medicine,Computational Medicine Group, Univeresity of Padova,Padova,Italy	Elisa Franchin et al
EPI_ISL_1181776	Microbiology and Virology Unit,Azienda Ospedale Padova,Padova,Italy	Department of Molecular Medicine,Computational Medicine Group, Univeresity of Padova,Padova,Italy	Elisa Franchin et al
EPI_ISL_1181804	Microbiology and Virology Unit,Azienda Ospedale Padova,Padova,Italy	Department of Molecular Medicine,Computational Medicine Group, Univeresity of Padova,Padova,Italy	Elisa Franchin et al
EPI_ISL_1064084	Microbiology and Virology Unit, Azienda Ospedale Padova, Padova, Italy	Department of Molecular Medicine, Computational Medicine Group, Univeresity of Padova, Padova, Italy	Elisa Franchin et al
EPI_ISL_1064098	Microbiology and Virology Unit, Azienda Ospedale Padova, Padova, Italy	Department of Molecular Medicine, Computational Medicine Group, Univeresity of Padova, Padova, Italy	Elisa Franchin et al

EPI_ISL_1064028	Microbiology and Virology Unit, Azienda Ospedale Padova, Padova, Italy	Department of Molecular Medicine, Computational Medicine Group, Univeresity of Padova, Padova, Italy	Elisa Franchin et al
EPI_ISL_429168	Ramathibodi Hospital	COVID-19 Network Investigations (CONI) Alliance	Elizabeth Batty et al
EPI_ISL_447004	Ramathibodi Hospital	COVID-19 Network Investigations (CONI) Alliance	Elizabeth Batty et al
EPI_ISL_445380	Ramathibodi Hospital	COVID-19 Network Investigations (CONI) Alliance	Elizabeth Batty et al
EPI_ISL_447029	Ramathibodi Hospital	COVID-19 Network Investigations (CONI) Alliance	Elizabeth Batty et al
EPI_ISL_458028	Hospital for Tropical Diseases	COVID-19 Network Investigations (CONI) Alliance	Elizabeth Batty et al
EPI_ISL_455916	Ramathibodi Hospital	COVID-19 Network Investigations (CONI) Alliance	Elizabeth Batty et al
EPI_ISL_1138829	Institute for Urban Disease Control and Prevention	COVID-19 Network Investigations (CONI) Alliance	Elizabeth Batty et al
EPI_ISL_1073954	Ramathibodi Hospital	COVID-19 Network Investigations (CONI) Alliance	Elizabeth Batty et al
EPI_ISL_1491570	Institute for Urban Disease Control and Prevention	COVID-19 Network Investigations (CONI) Alliance	Elizabeth Batty et al
EPI_ISL_1511124	Institute for Urban Disease Control and Prevention	COVID-19 Network Investigations (CONI) Alliance	Elizabeth Batty et al
EPI_ISL_1511128	Institute for Urban Disease Control and Prevention	COVID-19 Network Investigations (CONI) Alliance	Elizabeth Batty et al
EPI_ISL_1538423	Institute for Urban Disease Control and Prevention	COVID-19 Network Investigations (CONI) Alliance	Elizabeth Batty et al
EPI_ISL_1538419	Institute for Urban Disease Control and Prevention	COVID-19 Network Investigations (CONI) Alliance	Elizabeth Batty et al
EPI_ISL_1538418	Institute for Urban Disease Control and Prevention	COVID-19 Network Investigations (CONI) Alliance	Elizabeth Batty et al
EPI_ISL_1538431	Faculty of Medicine Vajira Hospital	COVID-19 Network Investigations (CONI) Alliance	Elizabeth Batty et al
EPI_ISL_1491572	Division of Epidemiology	COVID-19 Network Investigations (CONI) Alliance	Elizabeth Batty et al
EPI_ISL_1491563	Division of Epidemiology	COVID-19 Network Investigations (CONI) Alliance	Elizabeth Batty et al
EPI_ISL_1491564	Division of Epidemiology	COVID-19 Network Investigations (CONI) Alliance	Elizabeth Batty et al
EPI_ISL_1491566	Division of Epidemiology	COVID-19 Network Investigations (CONI) Alliance	Elizabeth Batty et al
EPI_ISL_1073965	Office of Diseases Prevention and Control Region 4 Saraburi	COVID-19 Network Investigations (CONI) Alliance	Elizabeth Batty et al
EPI_ISL_1491576	Faculty of Medicine Vajira Hospital	COVID-19 Network Investigations (CONI) Alliance	Elizabeth Batty et al
EPI_ISL_1491579	Faculty of Medicine Vajira Hospital	COVID-19 Network Investigations (CONI) Alliance	Elizabeth Batty et al
EPI_ISL_1491581	Faculty of Medicine Vajira Hospital	COVID-19 Network Investigations (CONI) Alliance	Elizabeth Batty et al
EPI_ISL_1538420	Faculty of Medicine Vajira Hospital	COVID-19 Network Investigations (CONI) Alliance	Elizabeth Batty et al
EPI_ISL_882780	Ramathibodi Chakri Naruebodindra Hospital	COVID-19 Network Investigations (CONI) Alliance	Elizabeth Batty et al
EPI_ISL_1073958	Ramathibodi Hospital	COVID-19 Network Investigations (CONI) Alliance	Elizabeth Batty et al
EPI_ISL_978544	Centre de Virologie des Maladies infectueuses Tropicales	Functional Genomic Platform UATRS-biology, CNRST	Elmostafa EL FAHIME et al
EPI_ISL_978549	Centre de Virologie des Maladies infectueuses Tropicales	Functional Genomic Platform UATRS-biology, CNRST	Elmostafa EL FAHIME et al
EPI_ISL_802856	Vilnius University Hospital Santaros Klinikos, Vilnius University	Institute of Biotechnology, Life Sciences Center, Vilnius University	Emilija Vasiliunaite et al

EPI_ISL_802860	Vilnius University Hospital Santaros Klinikos, Vilnius University	Institute of Biotechnology, Life Sciences Center, Vilnius University	Emilija Vasiliunaite et al
EPI_ISL_802861	Vilnius University Hospital Santaros Klinikos, Vilnius University	Institute of Biotechnology, Life Sciences Center, Vilnius University	Emilija Vasiliunaite et al
EPI_ISL_904939	Vilnius University Hospital Santaros Klinikos, Vilnius University	Institute of Biotechnology, Life Sciences Center, Vilnius University	Emilija Vasiliunaite et al
EPI_ISL_904945	Vilnius University Hospital Santaros Klinikos, Vilnius University	Institute of Biotechnology, Life Sciences Center, Vilnius University	Emilija Vasiliunaite et al
EPI_ISL_1420640	Biomedical Research Foundation of the Academy of Athens (BRFAA)	Greek Genome Center, Biomedical Research Foundation of the Academy of Athens (BRFAA)	Emmanouil Athanasiadis et al
EPI_ISL_1420691	Biomedical Research Foundation of the Academy of Athens (BRFAA)	Greek Genome Center, Biomedical Research Foundation of the Academy of Athens (BRFAA)	Emmanouil Athanasiadis et al
EPI_ISL_1420707	Biomedical Research Foundation of the Academy of Athens (BRFAA)	Greek Genome Center, Biomedical Research Foundation of the Academy of Athens (BRFAA)	Emmanouil Athanasiadis et al
EPI_ISL_1420709	Biomedical Research Foundation of the Academy of Athens (BRFAA)	Greek Genome Center, Biomedical Research Foundation of the Academy of Athens (BRFAA)	Emmanouil Athanasiadis et al
EPI_ISL_1063900	Rwanda National Reference Laboratory	Rwanda National Reference Laboratory	Enatha Mukantwari et al
EPI_ISL_1064147	Rwanda National Reference Laboratory	Rwanda National Reference Laboratory	ENatha Mukantwari et al
EPI_ISL_1064148	Rwanda National Reference Laboratory	Rwanda National Reference Laboratory	Enatha Mukantwari et al
EPI_ISL_1064168	Rwanda National Reference Laboratory	Rwanda National Reference Laboratory	Enatha Mukantwari et al
EPI_ISL_1498123	National Reference Laboratory	National Reference Laboratory	Enatha Mukantwari et al
EPI_ISL_1064170	Rwanda National Reference Laboratory	Rwanda National Reference Laboratory	Enatha Mukantwari et al
EPI_ISL_1041199	Hungarian Defence Forces Military Medical Centre	National Laboratory of Virology, Szentágothai Research Centre	Endre Gábor Tóth et al
EPI_ISL_1041202	Hungarian Defence Forces Military Medical Centre	National Laboratory of Virology, Szentágothai Research Centre	Endre Gábor Tóth et al
EPI_ISL_1041201	Hungarian Defence Forces Military Medical Centre	National Laboratory of Virology, Szentágothai Research Centre	Endre Gábor Tóth et al
EPI_ISL_1041200	Hungarian Defence Forces Military Medical Centre	National Laboratory of Virology, Szentágothai Research Centre	Endre Gábor Tóth et al
EPI_ISL_1041207	Hungarian Defence Forces Military Medical Centre	National Laboratory of Virology, Szentágothai Research Centre	Endre Gábor Tóth et al
EPI_ISL_1041208	Hungarian Defence Forces Military Medical Centre	National Laboratory of Virology, Szentágothai Research Centre	Endre Gábor Tóth et al
EPI_ISL_1041211	Hungarian Defence Forces Military Medical Centre	National Laboratory of Virology, Szentágothai Research Centre	Endre Gábor Tóth et al
EPI_ISL_1041212	Hungarian Defence Forces Military Medical Centre	National Laboratory of Virology, Szentágothai Research Centre	Endre Gábor Tóth et al
EPI_ISL_526225	Hungarian Defence Forces Military Medical Centre	National Laboratory of Virology, Szentágothai Research Centre	Endre Gábor Tóth et al
EPI_ISL_526236	Hungarian Defence Forces Military Medical Centre	National Laboratory of Virology, Szentágothai Research Centre	Endre Gábor Tóth et al
EPI_ISL_526220	Hungarian Defence Forces Military Medical Centre	National Laboratory of Virology, Szentágothai Research Centre	Endre Gábor Tóth et al
EPI_ISL_526221	Hungarian Defence Forces Military Medical Centre	National Laboratory of Virology, Szentágothai Research Centre	Endre Gábor Tóth et al
EPI_ISL_526223	Hungarian Defence Forces Military Medical Centre	National Laboratory of Virology, Szentágothai Research Centre	Endre Gábor Tóth et al
EPI_ISL_671425	University of Debrecen, Department of Medical Microbiology	National Laboratory of Virology, Szentágothai Research Centre	Endre Gábor Tóth et al
EPI_ISL_671426	University of Debrecen, Department of Medical Microbiology	National Laboratory of Virology, Szentágothai Research Centre	Endre Gábor Tóth et al

EPI_ISL_671429	University of Debrecen, Department of Medical Microbiology	National Laboratory of Virology, Szentágothai Research Centre	Endre Gábor Tóth et al
EPI_ISL_671437	University of Debrecen, Department of Medical Microbiology	National Laboratory of Virology, Szentágothai Research Centre	Endre Gábor Tóth et al
EPI_ISL_671439	University of Debrecen, Department of Medical Microbiology	National Laboratory of Virology, Szentágothai Research Centre	Endre Gábor Tóth et al
EPI_ISL_671441	University of Debrecen, Department of Medical Microbiology	National Laboratory of Virology, Szentágothai Research Centre	Endre Gábor Tóth et al
EPI_ISL_671442	University of Debrecen, Department of Medical Microbiology	National Laboratory of Virology, Szentágothai Research Centre	Endre Gábor Tóth et al
EPI_ISL_671451	University of Debrecen, Department of Medical Microbiology	National Laboratory of Virology, Szentágothai Research Centre	Endre Gábor Tóth et al
EPI_ISL_678396	University of Debrecen, Department of Medical Microbiology	National Laboratory of Virology, Szentágothai Research Centre	Endre Gábor Tóth et al
EPI_ISL_477620	University of Szeged, Institute of Clinical Microbiology	National Laboratory of Virology, Szentágothai Research Centre	Endre Gábor Tóth et al
EPI_ISL_677729	University of Szeged, Institute of Clinical Microbiology	National Laboratory of Virology, Szentágothai Research Centre	Endre Gábor Tóth et al
EPI_ISL_477622	University of Szeged, Institute of Clinical Microbiology	National Laboratory of Virology, Szentágothai Research Centre	Endre Gábor Tóth et al
EPI_ISL_677770	University of Szeged, Institute of Clinical Microbiology	National Laboratory of Virology, Szentágothai Research Centre	Endre Gábor Tóth et al
EPI_ISL_677798	University of Szeged, Institute of Clinical Microbiology	National Laboratory of Virology, Szentágothai Research Centre	Endre Gábor Tóth et al
EPI_ISL_984749	Gazi University Faculty of Medicine, Medical Virology Laboratory	Gazi University Faculty of Medicine, Medical Virology Laboratory	Erdem Şahin et al
EPI_ISL_984750	Gazi University Faculty of Medicine, Medical Virology Laboratory	Gazi University Faculty of Medicine, Medical Virology Laboratory	Erdem Şahin et al
EPI_ISL_984990	Gazi University Faculty of Medicine, Medical Virology Laboratory	Gazi University Faculty of Medicine, Medical Virology Laboratory	Erdem Şahin et al
EPI_ISL_730575	Gazi University Faculty of Medicine, Medical Virology Laboratory	Gazi University Faculty of Medicine, Medical Virology Laboratory	Erdem Şahin et al
EPI_ISL_730572	Gazi University Faculty of Medicine, Medical Virology Laboratory	Gazi University Faculty of Medicine, Medical Virology Laboratory	Erdem Şahin et al
EPI_ISL_730571	Gazi University Faculty of Medicine, Medical Virology Laboratory	Gazi University Faculty of Medicine, Medical Virology Laboratory	Erdem Şahin et al
EPI_ISL_730568	Gazi University Faculty of Medicine, Medical Virology Laboratory	Gazi University Faculty of Medicine, Medical Virology Laboratory	Erdem Şahin et al
EPI_ISL_730569	Gazi University Faculty of Medicine, Medical Virology Laboratory	Gazi University Faculty of Medicine, Medical Virology Laboratory	Erdem Şahin et al
EPI_ISL_730570	Gazi University Faculty of Medicine, Medical Virology Laboratory	Gazi University Faculty of Medicine, Medical Virology Laboratory	Erdem Şahin et al
EPI_ISL_730620	Gazi University Faculty of Medicine, Medical Virology Laboratory	Gazi University Faculty of Medicine, Medical Virology Laboratory	Erdem Şahin et al
EPI_ISL_730577	Gazi University Faculty of Medicine, Medical Virology Laboratory	Gazi University Faculty of Medicine, Medical Virology Laboratory	Erdem Şahin et al
EPI_ISL_1273090	Biochemistry and Molecular Biology Department-Faculty of Medicine, Al-Quds University	Biochemistry and Molecular Biology Department-Faculty of Medicine, Al-Quds University	Ereqat et al
EPI_ISL_1273091	Biochemistry and Molecular Biology Department-Faculty of Medicine, Al-Quds University	Biochemistry and Molecular Biology Department-Faculty of Medicine, Al-Quds University	Ereqat et al
EPI_ISL_1273092	Biochemistry and Molecular Biology Department-Faculty of Medicine, Al-Quds University	Biochemistry and Molecular Biology Department-Faculty of Medicine, Al-Quds University	Ereqat et al
EPI_ISL_1273093	Biochemistry and Molecular Biology Department-Faculty of Medicine, Al-Quds University	Biochemistry and Molecular Biology Department-Faculty of Medicine, Al-Quds University	Ereqat et al
EPI_ISL_1273099	Faculty of Medicine, Al-Quds University	Faculty of Medicine, Al-Quds University	Ereqat et al
EPI_ISL_1273102	Faculty of Medicine, Al-Quds University	Faculty of Medicine, Al-Quds University	Ereqat et al

EPI_ISL_661272	Al-Quds Nutrition and Health Research Institute, Al-Quds University	Al-Quds Nutrition and Health Research Institute, Al-Quds University	Ereqat et al
EPI_ISL_411902	Virology Unit, Institut Pasteur du Cambodge.	Virology Unit, Institut Pasteur du Cambodge (Sequencing done by: Jessica E Manning/Jennifer A Bohl at Malaria and Vector Research Research Laboratory, National Institute of Allergy and Infectious Diseases and Vida Ahyong from Chan-Zuckerberg Biohub)	Erik A Karlsson et al
EPI_ISL_516613	Instituto de Diagnostico y Referencia Epidemiologicos (INDRE)	Instituto de Diagnostico y Referencia Epidemiologicos (INDRE)	Ernesto Ramirez-Gonzalez et al
EPI_ISL_516614	Instituto de Diagnostico y Referencia Epidemiologicos (INDRE)	Instituto de Diagnostico y Referencia Epidemiologicos (INDRE)	Ernesto Ramirez-Gonzalez et al
EPI_ISL_516615	Instituto de Diagnostico y Referencia Epidemiologicos (INDRE)	Instituto de Diagnostico y Referencia Epidemiologicos (INDRE)	Ernesto Ramirez-Gonzalez et al
EPI_ISL_516616	Instituto de Diagnostico y Referencia Epidemiologicos (INDRE)	Instituto de Diagnostico y Referencia Epidemiologicos (INDRE)	Ernesto Ramirez-Gonzalez et al
EPI_ISL_1195537	Hospital Universitario 12 de Octubre	SeqCOVID-SPAIN consortium/IBV(CSIC)	Esther Viedma et al
EPI_ISL_1195547	Hospital Universitario 12 de Octubre	SeqCOVID-SPAIN consortium/IBV(CSIC)	Esther Viedma et al
EPI_ISL_1195548	Hospital Universitario 12 de Octubre	SeqCOVID-SPAIN consortium/IBV(CSIC)	Esther Viedma et al
EPI_ISL_1195557	Hospital Universitario 12 de Octubre	SeqCOVID-SPAIN consortium/IBV(CSIC)	Esther Viedma et al
EPI_ISL_1272574	Hospital Universitario 12 de Octubre	SeqCOVID-SPAIN consortium/IBV(CSIC)	Esther Viedma et al
EPI_ISL_1159375	Bahman Hospital	Laboratory of Molecular Biology and Cancer Immunology,Lebanese University Public Health England	Fadi Abdel Sater et al
EPI_ISL_1196793	Clinical Microbiology Laboratory, Faculty of Medicine, Universitas Indonesia	Faculty of Medicine, Universitas Indonesia	Fadilah et al
EPI_ISL_512922	Pathogen Genomics Lab King Abdullah University of Science and Technology (KAUST)	Pathogen Genomics Lab King Abdullah University of Science and Technology(KAUST)	Fadwa Alofi et al
EPI_ISL_512923	Pathogen Genomics Lab King Abdullah University of Science and Technology (KAUST)	Pathogen Genomics Lab King Abdullah University of Science and Technology(KAUST)	Fadwa Alofi et al
EPI_ISL_458125	Oman National Influenza Centre	Department of Microbiology and Immunology-SQUH	Fahad Zadjali et al
EPI_ISL_457984	Oman-NIC	Department of Microbiology and Immunology-SQUH	Fahad Zadjali et al
EPI_ISL_491972	Oman-NIC	Department of Microbiology and Immunology-SQUH	Fahad Zadjali et al
EPI_ISL_491985	Oman-NIC	Department of Microbiology and Immunology-SQUH	Fahad Zadjali et al
EPI_ISL_491986	Oman-NIC	Department of Microbiology and Immunology-SQUH	Fahad Zadjali et al
EPI_ISL_491989	Oman-NIC	Department of Microbiology and Immunology-SQUH	Fahad Zadjali et al
EPI_ISL_492015	Oman-NIC	Department of Microbiology and Immunology-SQUH	Fahad Zadjali et al
EPI_ISL_492016	Oman-NIC	Department of Microbiology and Immunology-SQUH	Fahad Zadjali et al
EPI_ISL_492018	Oman-NIC	Department of Microbiology and Immunology-SQUH	Fahad Zadjali et al
EPI_ISL_492020	Oman-NIC	Department of Microbiology and Immunology-SQUH	Fahad Zadjali et al
EPI_ISL_492025	Oman-NIC	Department of Microbiology and Immunology-SQUH	Fahad Zadjali et al
EPI_ISL_422424	Jaber Al Ahmad Al Sabah Hospital	Dasman diabetes Institute	Fahd Al-Mulla et al
EPI_ISL_416541	Dasman Diabetes Institute and Virology Laboratory Ministry of Health	Dasman Diabetes Institute	Fahd Al-Mulla et al

EPI_ISL_416543	Dasman Diabetes Institute	Dasman Diabetes Institute	Fahd Al-Mulla et al
EPI_ISL_463001	unknown	Clinical virology	Fares et al
EPI_ISL_463002	unknown	Clinical virology	Fares et al
EPI_ISL_477227	Institute for Stem Cell Science and Regenerative Medicine	National Centre for Biological Sciences	Farhan Ali et al
EPI_ISL_978536	Centre de Virologie des Maladies infectueuses Tropicales	Functional Genomic Platform UATRS-biology, CNRST	Farida HILALI et al
EPI_ISL_978546	Centre de Virologie des Maladies infectueuses Tropicales	Functional Genomic Platform UATRS-biology, CNRST	Farida HILALI et al
EPI_ISL_437334	Ministry of Health Turkey	Ministry of Health Turkey	Fatma Bayrakdar et al
EPI_ISL_812774	Ministry of Health Turkey	Ministry of Health Turkey	Fatma Bayrakdar et al
EPI_ISL_812765	Ministry of Health Turkey	Ministry of Health Turkey	Fatma Bayrakdar et al
EPI_ISL_812777	Ministry of Health Turkey	Ministry of Health Turkey	Fatma Bayrakdar et al
EPI_ISL_1379950	Ministry of Health Turkey	Ministry of Health Turkey	Fatma Bayrakdar et al
EPI_ISL_437319	Ministry of Health Turkey	Ministry of Health Turkey	Fatma Bayrakdar et al
EPI_ISL_437325	Ministry of Health Turkey	Ministry of Health Turkey	Fatma Bayrakdar et al
EPI_ISL_437321	Ministry of Health Turkey	Ministry of Health Turkey	Fatma Bayrakdar et al
EPI_ISL_1534486	Ministry of Health Turkey	Ministry of Health Turkey	Fatma Bayrakdar et al
EPI_ISL_1008621	Ministry of Health Turkey	Ministry of Health Turkey	Fatma Bayrakdar et al
EPI_ISL_812876	Ministry of Health Turkey	Ministry of Health Turkey	Fatma Bayrakdar et al
EPI_ISL_812877	Ministry of Health Turkey	Ministry of Health Turkey	Fatma Bayrakdar et al
EPI_ISL_814079	Ministry of Health Turkey	Ministry of Health Turkey	Fatma Bayrakdar et al
EPI_ISL_814091	Ministry of Health Turkey	Ministry of Health Turkey	Fatma Bayrakdar et al
EPI_ISL_814067	Ministry of Health Turkey	Ministry of Health Turkey	Fatma Bayrakdar et al
EPI_ISL_814065	Ministry of Health Turkey	Ministry of Health Turkey	Fatma Bayrakdar et al
EPI_ISL_814066	Ministry of Health Turkey	Ministry of Health Turkey	Fatma Bayrakdar et al
EPI_ISL_1063969	Ministry of Health Turkey	Ministry of Health Turkey	Fatma Bayrakdar et al
EPI_ISL_437307	Ministry of Health Turkey	Ministry of Health Turkey	Fatma Bayrakdar et al
EPI_ISL_1170075	Ministry of Health Turkey	Ministry of Health Turkey	Fatma Bayrakdar et al
EPI_ISL_1183638	Ministry of Health Turkey	Ministry of Health Turkey	Fatma Bayrakdar et al
EPI_ISL_1211725	Ministry of Health Turkey	Ministry of Health Turkey	Fatma Bayrakdar et al
EPI_ISL_1211766	Ministry of Health Turkey	Ministry of Health Turkey	Fatma Bayrakdar et al
EPI_ISL_1263298	Ministry of Health Turkey	Ministry of Health Turkey	Fatma Bayrakdar et al

EPI_ISL_812886	Ministry of Health Turkey	Ministry of Health Turkey	Fatma Bayrakdar et al
EPI_ISL_812897	Ministry of Health Turkey	Ministry of Health Turkey	Fatma Bayrakdar et al
EPI_ISL_812902	Ministry of Health Turkey	Ministry of Health Turkey	Fatma Bayrakdar et al
EPI_ISL_1403611	Molecular Virology Unit, Microbiology and Virology Depertment, Fondazione IRCCS Policlinico San Matteo, Pavia	Molecular Virology Unit, Microbiology and Virology Depertment, Fondazione IRCCS Policlinico San Matteo, Pavia	Federica Giardina et al
EPI_ISL_779155	Laboratório de Microbiologia Molecular - Universidade FEEVALE	Bioinformatics Laboratory / LNCC	Felipe Benites et al
EPI_ISL_779156	Laboratório de Microbiologia Molecular - Universidade FEEVALE	Bioinformatics Laboratory / LNCC	Felipe Benites et al
EPI_ISL_770555	Laboratório de Microbiologia Molecular - Universidade FEEVALE	Bioinformatics Laboratory / LNCC	Felipe Benites et al
EPI_ISL_770556	Laboratório de Microbiologia Molecular - Universidade FEEVALE	Bioinformatics Laboratory / LNCC	Felipe Benites et al
EPI_ISL_770557	Laboratório de Microbiologia Molecular - Universidade FEEVALE	Bioinformatics Laboratory / LNCC	Felipe Benites et al
EPI_ISL_770558	Laboratório de Microbiologia Molecular - Universidade FEEVALE	Bioinformatics Laboratory / LNCC	Felipe Benites et al
EPI_ISL_770559	Laboratório de Microbiologia Molecular - Universidade FEEVALE	Bioinformatics Laboratory / LNCC	Felipe Benites et al
EPI_ISL_770560	Laboratório de Microbiologia Molecular - Universidade FEEVALE	Bioinformatics Laboratory / LNCC	Felipe Benites et al
EPI_ISL_770561	Laboratório de Microbiologia Molecular - Universidade FEEVALE	Bioinformatics Laboratory / LNCC	Felipe Benites et al
EPI_ISL_770562	Laboratório de Microbiologia Molecular - Universidade FEEVALE	Bioinformatics Laboratory / LNCC	Felipe Benites et al
EPI_ISL_770563	Laboratório de Microbiologia Molecular - Universidade FEEVALE	Bioinformatics Laboratory / LNCC	Felipe Benites et al
EPI_ISL_770564	Laboratório de Microbiologia Molecular - Universidade FEEVALE	Bioinformatics Laboratory / LNCC	Felipe Benites et al
EPI_ISL_770565	Laboratório de Microbiologia Molecular - Universidade FEEVALE	Bioinformatics Laboratory / LNCC	Felipe Benites et al
EPI_ISL_770566	Laboratório de Microbiologia Molecular - Universidade FEEVALE	Bioinformatics Laboratory / LNCC	Felipe Benites et al
EPI_ISL_770567	Laboratório de Microbiologia Molecular - Universidade FEEVALE	Bioinformatics Laboratory / LNCC	Felipe Benites et al
EPI_ISL_770568	Laboratório de Microbiologia Molecular - Universidade FEEVALE	Bioinformatics Laboratory / LNCC	Felipe Benites et al
EPI_ISL_770569	Laboratório de Microbiologia Molecular - Universidade FEEVALE	Bioinformatics Laboratory / LNCC	Felipe Benites et al
EPI_ISL_770570	Laboratório de Microbiologia Molecular - Universidade FEEVALE	Bioinformatics Laboratory / LNCC	Felipe Benites et al
EPI_ISL_770571	Laboratório de Microbiologia Molecular - Universidade FEEVALE	Bioinformatics Laboratory / LNCC	Felipe Benites et al
EPI_ISL_770572	Laboratório de Microbiologia Molecular - Universidade FEEVALE	Bioinformatics Laboratory / LNCC	Felipe Benites et al
EPI_ISL_779157	Laboratório de Microbiologia Molecular - Universidade FEEVALE	Bioinformatics Laboratory / LNCC	Felipe Benites et al
EPI_ISL_770573	Laboratório de Microbiologia Molecular - Universidade FEEVALE	Bioinformatics Laboratory / LNCC	Felipe Benites et al
EPI_ISL_770574	Laboratório de Microbiologia Molecular - Universidade FEEVALE	Bioinformatics Laboratory / LNCC	Felipe Benites et al
EPI_ISL_779158	Laboratório de Microbiologia Molecular - Universidade FEEVALE	Bioinformatics Laboratory / LNCC	Felipe Benites et al
EPI_ISL_770575	Laboratório de Microbiologia Molecular - Universidade FEEVALE	Bioinformatics Laboratory / LNCC	Felipe Benites et al
EPI_ISL_770576	Laboratório de Microbiologia Molecular - Universidade FEEVALE	Bioinformatics Laboratory / LNCC	Felipe Benites et al

EPI_ISL_770577	Laboratório de Microbiologia Molecular - Universidade FEEVALE	Bioinformatics Laboratory / LNCC	Felipe Benites et al
EPI_ISL_770553	Laboratório de Microbiologia Molecular - Universidade FEEVALE	Bioinformatics Laboratory / LNCC	Felipe Benites et al
EPI_ISL_770578	Laboratório de Microbiologia Molecular - Universidade FEEVALE	Bioinformatics Laboratory / LNCC	Felipe Benites et al
EPI_ISL_770579	Laboratório de Microbiologia Molecular - Universidade FEEVALE	Bioinformatics Laboratory / LNCC	Felipe Benites et al
EPI_ISL_779159	Laboratório de Microbiologia Molecular - Universidade FEEVALE	Bioinformatics Laboratory / LNCC	Felipe Benites et al
EPI_ISL_770580	Laboratório de Microbiologia Molecular - Universidade FEEVALE	Bioinformatics Laboratory / LNCC	Felipe Benites et al
EPI_ISL_770581	Laboratório de Microbiologia Molecular - Universidade FEEVALE	Bioinformatics Laboratory / LNCC	Felipe Benites et al
EPI_ISL_770582	Laboratório de Microbiologia Molecular - Universidade FEEVALE	Bioinformatics Laboratory / LNCC	Felipe Benites et al
EPI_ISL_770583	Laboratório de Microbiologia Molecular - Universidade FEEVALE	Bioinformatics Laboratory / LNCC	Felipe Benites et al
EPI_ISL_770584	Laboratório de Microbiologia Molecular - Universidade FEEVALE	Bioinformatics Laboratory / LNCC	Felipe Benites et al
EPI_ISL_770585	Laboratório de Microbiologia Molecular - Universidade FEEVALE	Bioinformatics Laboratory / LNCC	Felipe Benites et al
EPI_ISL_770586	Laboratório de Microbiologia Molecular - Universidade FEEVALE	Bioinformatics Laboratory / LNCC	Felipe Benites et al
EPI_ISL_770587	Laboratório de Microbiologia Molecular - Universidade FEEVALE	Bioinformatics Laboratory / LNCC	Felipe Benites et al
EPI_ISL_770588	Laboratório de Microbiologia Molecular - Universidade FEEVALE	Bioinformatics Laboratory / LNCC	Felipe Benites et al
EPI_ISL_779160	Laboratório de Microbiologia Molecular - Universidade FEEVALE	Bioinformatics Laboratory / LNCC	Felipe Benites et al
EPI_ISL_770589	Laboratório de Microbiologia Molecular - Universidade FEEVALE	Bioinformatics Laboratory / LNCC	Felipe Benites et al
EPI_ISL_770590	Laboratório de Microbiologia Molecular - Universidade FEEVALE	Bioinformatics Laboratory / LNCC	Felipe Benites et al
EPI_ISL_779161	Laboratório de Microbiologia Molecular - Universidade FEEVALE	Bioinformatics Laboratory / LNCC	Felipe Benites et al
EPI_ISL_770591	Laboratório de Microbiologia Molecular - Universidade FEEVALE	Bioinformatics Laboratory / LNCC	Felipe Benites et al
EPI_ISL_770592	Laboratório de Microbiologia Molecular - Universidade FEEVALE	Bioinformatics Laboratory / LNCC	Felipe Benites et al
EPI_ISL_770593	Laboratório de Microbiologia Molecular - Universidade FEEVALE	Bioinformatics Laboratory / LNCC	Felipe Benites et al
EPI_ISL_770594	Laboratório de Microbiologia Molecular - Universidade FEEVALE	Bioinformatics Laboratory / LNCC	Felipe Benites et al
EPI_ISL_770595	Laboratório de Microbiologia Molecular - Universidade FEEVALE	Bioinformatics Laboratory / LNCC	Felipe Benites et al
EPI_ISL_770596	Laboratório de Microbiologia Molecular - Universidade FEEVALE	Bioinformatics Laboratory / LNCC	Felipe Benites et al
EPI_ISL_770551	Laboratório de Microbiologia Molecular - Universidade FEEVALE	Bioinformatics Laboratory / LNCC	Felipe Benites et al
EPI_ISL_770597	Laboratório de Microbiologia Molecular - Universidade FEEVALE	Bioinformatics Laboratory / LNCC	Felipe Benites et al
EPI_ISL_770598	Laboratório de Microbiologia Molecular - Universidade FEEVALE	Bioinformatics Laboratory / LNCC	Felipe Benites et al
EPI_ISL_770599	Laboratório de Microbiologia Molecular - Universidade FEEVALE	Bioinformatics Laboratory / LNCC	Felipe Benites et al
EPI_ISL_770552	Laboratório de Microbiologia Molecular - Universidade FEEVALE	Bioinformatics Laboratory / LNCC	Felipe Benites et al
EPI_ISL_770600	Laboratório de Microbiologia Molecular - Universidade FEEVALE	Bioinformatics Laboratory / LNCC	Felipe Benites et al

EPI_ISL_770601	Laboratório de Microbiologia Molecular - Universidade FEEVALE	Bioinformatics Laboratory / LNCC	Felipe Benites et al
EPI_ISL_779162	Laboratório de Microbiologia Molecular - Universidade FEEVALE	Bioinformatics Laboratory / LNCC	Felipe Benites et al
EPI_ISL_770602	Laboratório de Microbiologia Molecular - Universidade FEEVALE	Bioinformatics Laboratory / LNCC	Felipe Benites et al
EPI_ISL_770603	Laboratório de Microbiologia Molecular - Universidade FEEVALE	Bioinformatics Laboratory / LNCC	Felipe Benites et al
EPI_ISL_770604	Laboratório de Microbiologia Molecular - Universidade FEEVALE	Bioinformatics Laboratory / LNCC	Felipe Benites et al
EPI_ISL_770605	Laboratório de Microbiologia Molecular - Universidade FEEVALE	Bioinformatics Laboratory / LNCC	Felipe Benites et al
EPI_ISL_770606	Laboratório de Microbiologia Molecular - Universidade FEEVALE	Bioinformatics Laboratory / LNCC	Felipe Benites et al
EPI_ISL_779163	Laboratório de Microbiologia Molecular - Universidade FEEVALE	Bioinformatics Laboratory / LNCC	Felipe Benites et al
EPI_ISL_770607	Laboratório de Microbiologia Molecular - Universidade FEEVALE	Bioinformatics Laboratory / LNCC	Felipe Benites et al
EPI_ISL_770608	Laboratório de Microbiologia Molecular - Universidade FEEVALE	Bioinformatics Laboratory / LNCC	Felipe Benites et al
EPI_ISL_770609	Laboratório de Microbiologia Molecular - Universidade FEEVALE	Bioinformatics Laboratory / LNCC	Felipe Benites et al
EPI_ISL_770610	Laboratório de Microbiologia Molecular - Universidade FEEVALE	Bioinformatics Laboratory / LNCC	Felipe Benites et al
EPI_ISL_770611	Laboratório de Microbiologia Molecular - Universidade FEEVALE	Bioinformatics Laboratory / LNCC	Felipe Benites et al
EPI_ISL_770612	Laboratório de Microbiologia Molecular - Universidade FEEVALE	Bioinformatics Laboratory / LNCC	Felipe Benites et al
EPI_ISL_779164	Laboratório de Microbiologia Molecular - Universidade FEEVALE	Bioinformatics Laboratory / LNCC	Felipe Benites et al
EPI_ISL_770613	Laboratório de Microbiologia Molecular - Universidade FEEVALE	Bioinformatics Laboratory / LNCC	Felipe Benites et al
EPI_ISL_770614	Laboratório de Microbiologia Molecular - Universidade FEEVALE	Bioinformatics Laboratory / LNCC	Felipe Benites et al
EPI_ISL_770615	Laboratório de Microbiologia Molecular - Universidade FEEVALE	Bioinformatics Laboratory / LNCC	Felipe Benites et al
EPI_ISL_770616	Laboratório de Microbiologia Molecular - Universidade FEEVALE	Bioinformatics Laboratory / LNCC	Felipe Benites et al
EPI_ISL_770617	Laboratório de Microbiologia Molecular - Universidade FEEVALE	Bioinformatics Laboratory / LNCC	Felipe Benites et al
EPI_ISL_779165	Laboratório de Microbiologia Molecular - Universidade FEEVALE	Bioinformatics Laboratory / LNCC	Felipe Benites et al
EPI_ISL_779166	Laboratório de Microbiologia Molecular - Universidade FEEVALE	Bioinformatics Laboratory / LNCC	Felipe Benites et al
EPI_ISL_770618	Laboratório de Microbiologia Molecular - Universidade FEEVALE	Bioinformatics Laboratory / LNCC	Felipe Benites et al
EPI_ISL_770619	Laboratório de Microbiologia Molecular - Universidade FEEVALE	Bioinformatics Laboratory / LNCC	Felipe Benites et al
EPI_ISL_770620	Laboratório de Microbiologia Molecular - Universidade FEEVALE	Bioinformatics Laboratory / LNCC	Felipe Benites et al
EPI_ISL_770554	Laboratório de Microbiologia Molecular - Universidade FEEVALE	Bioinformatics Laboratory / LNCC	Felipe Benites et al
EPI_ISL_770621	Laboratório de Microbiologia Molecular - Universidade FEEVALE	Bioinformatics Laboratory / LNCC	Felipe Benites et al
EPI_ISL_770622	Laboratório de Microbiologia Molecular - Universidade FEEVALE	Bioinformatics Laboratory / LNCC	Felipe Benites et al
EPI_ISL_770623	Laboratório de Microbiologia Molecular - Universidade FEEVALE	Bioinformatics Laboratory / LNCC	Felipe Benites et al
EPI_ISL_770624	Laboratório de Microbiologia Molecular - Universidade FEEVALE	Bioinformatics Laboratory / LNCC	Felipe Benites et al

EPI_ISL_779167	Laboratório de Microbiologia Molecular - Universidade FEEVALE	Bioinformatics Laboratory / LNCC	Felipe Benites et al
EPI_ISL_779168	Laboratório de Microbiologia Molecular - Universidade FEEVALE	Bioinformatics Laboratory / LNCC	Felipe Benites et al
EPI_ISL_770625	Laboratório de Microbiologia Molecular - Universidade FEEVALE	Bioinformatics Laboratory / LNCC	Felipe Benites et al
EPI_ISL_770626	Laboratório de Microbiologia Molecular - Universidade FEEVALE	Bioinformatics Laboratory / LNCC	Felipe Benites et al
EPI_ISL_770627	Laboratório de Microbiologia Molecular - Universidade FEEVALE	Bioinformatics Laboratory / LNCC	Felipe Benites et al
EPI_ISL_770628	Laboratório de Microbiologia Molecular - Universidade FEEVALE	Bioinformatics Laboratory / LNCC	Felipe Benites et al
EPI_ISL_770629	Laboratório de Microbiologia Molecular - Universidade FEEVALE	Bioinformatics Laboratory / LNCC	Felipe Benites et al
EPI_ISL_770630	Laboratório de Microbiologia Molecular - Universidade FEEVALE	Bioinformatics Laboratory / LNCC	Felipe Benites et al
EPI_ISL_792528	Laboratorio Central, Ministerio de Salud Córdoba	Instituto de Patologia Vegetal (CIAP-INTA) on behalf of 'Proyecto Argentino Interinstitucional de genomica de SARS-CoV-2' (PAIS Consortium)	Fernández et al
EPI_ISL_792531	Laboratorio Central, Ministerio de Salud Córdoba	Instituto de Patologia Vegetal (CIAP-INTA) on behalf of 'Proyecto Argentino Interinstitucional de genomica de SARS-CoV-2' (PAIS Consortium)	Fernández et al
EPI_ISL_792533	Laboratorio Central, Ministerio de Salud Córdoba	Instituto de Patologia Vegetal (CIAP-INTA) on behalf of 'Proyecto Argentino Interinstitucional de genomica de SARS-CoV-2' (PAIS Consortium)	Fernández et al
EPI_ISL_792537	Laboratorio Central, Ministerio de Salud Córdoba	Instituto de Patologia Vegetal (CIAP-INTA) on behalf of 'Proyecto Argentino Interinstitucional de genomica de SARS-CoV-2' (PAIS Consortium)	Fernández et al
EPI_ISL_792540	Laboratorio Central, Ministerio de Salud Córdoba	Instituto de Patologia Vegetal (CIAP-INTA) on behalf of 'Proyecto Argentino Interinstitucional de genomica de SARS-CoV-2' (PAIS Consortium)	Fernández et al
EPI_ISL_1396016	Laboratorio Central, Ministerio de Salud Cordoba	Instituto de Patologia Vegetal (CIAP-INTA) on behalf of 'Proyecto Argentino Interinstitucional de genomica de SARS-CoV-2' (PAIS Consortium)	Fernández et al
EPI_ISL_1395973	Laboratorio Central, Ministerio de Salud Cordoba	Instituto de Patologia Vegetal (CIAP-INTA) on behalf of 'Proyecto Argentino Interinstitucional de genomica de SARS-CoV-2' (PAIS Consortium)	Fernández et al
EPI_ISL_1396019	Laboratorio Central, Ministerio de Salud Cordoba	Instituto de Patologia Vegetal (CIAP-INTA) on behalf of 'Proyecto Argentino Interinstitucional de genomica de SARS-CoV-2' (PAIS Consortium)	Fernández et al
EPI_ISL_1395975	Laboratorio Central, Ministerio de Salud Cordoba	Instituto de Patologia Vegetal (CIAP-INTA) on behalf of 'Proyecto Argentino Interinstitucional de genomica de SARS-CoV-2' (PAIS Consortium)	Fernández et al
EPI_ISL_1395979	Laboratorio Central, Ministerio de Salud Cordoba	Instituto de Patologia Vegetal (CIAP-INTA) on behalf of 'Proyecto Argentino Interinstitucional de genomica de SARS-CoV-2' (PAIS Consortium)	Fernández et al
EPI_ISL_1395980	Laboratorio Central, Ministerio de Salud Cordoba	Instituto de Patologia Vegetal (CIAP-INTA) on behalf of 'Proyecto Argentino Interinstitucional de genomica de SARS-CoV-2' (PAIS Consortium)	Fernández et al
EPI_ISL_1395982	Laboratorio Central, Ministerio de Salud Cordoba	Instituto de Patologia Vegetal (CIAP-INTA) on behalf of 'Proyecto Argentino Interinstitucional de genomica de SARS-CoV-2' (PAIS Consortium)	Fernández et al
EPI_ISL_1395971	Laboratorio Central, Ministerio de Salud Cordoba	Instituto de Patologia Vegetal (CIAP-INTA) on behalf of 'Proyecto Argentino Interinstitucional de genomica de SARS-CoV-2' (PAIS Consortium)	Fernández et al
EPI_ISL_1395988	Laboratorio Central, Ministerio de Salud Cordoba	Instituto de Patologia Vegetal (CIAP-INTA) on behalf of 'Proyecto Argentino Interinstitucional de genomica de SARS-CoV-2' (PAIS Consortium)	Fernández et al
EPI_ISL_1396040	Laboratorio Central, Ministerio de Salud Cordoba	Instituto de Patologia Vegetal (CIAP-INTA) on behalf of 'Proyecto Argentino Interinstitucional de genomica de SARS-CoV-2' (PAIS Consortium)	Fernández et al
EPI_ISL_1395991	Laboratorio Central, Ministerio de Salud Cordoba	Instituto de Patologia Vegetal (CIAP-INTA) on behalf of 'Proyecto Argentino Interinstitucional de genomica de SARS-CoV-2' (PAIS Consortium)	Fernández et al

EPI_ISL_1396047	Laboratorio Central, Ministerio de Salud Cordoba	Instituto de Patologia Vegetal (CIAP-INTA) on behalf of 'Proyecto Argentino Interinstitucional de genomica de SARS-CoV-2' (PAIS Consortium)	Fernández et al
EPI_ISL_1396000	Laboratorio Central, Ministerio de Salud Cordoba	Instituto de Patologia Vegetal (CIAP-INTA) on behalf of 'Proyecto Argentino Interinstitucional de genomica de SARS-CoV-2' (PAIS Consortium)	Fernández et al
EPI_ISL_1396041	Laboratorio Central, Ministerio de Salud Cordoba	Instituto de Patologia Vegetal (CIAP-INTA) on behalf of 'Proyecto Argentino Interinstitucional de genomica de SARS-CoV-2' (PAIS Consortium)	Fernández et al
EPI_ISL_1396002	Laboratorio Central, Ministerio de Salud Cordoba	Instituto de Patologia Vegetal (CIAP-INTA) on behalf of 'Proyecto Argentino Interinstitucional de genomica de SARS-CoV-2' (PAIS Consortium)	Fernández et al
EPI_ISL_1396003	Laboratorio Central, Ministerio de Salud Cordoba	Instituto de Patologia Vegetal (CIAP-INTA) on behalf of 'Proyecto Argentino Interinstitucional de genomica de SARS-CoV-2' (PAIS Consortium)	Fernández et al
EPI_ISL_1396060	Laboratorio Central, Ministerio de Salud Cordoba	Instituto de Patologia Vegetal (CIAP-INTA) on behalf of 'Proyecto Argentino Interinstitucional de genomica de SARS-CoV-2' (PAIS Consortium)	Fernández et al
EPI_ISL_1396004	Laboratorio Central, Ministerio de Salud Cordoba	Instituto de Patologia Vegetal (CIAP-INTA) on behalf of 'Proyecto Argentino Interinstitucional de genomica de SARS-CoV-2' (PAIS Consortium)	Fernández et al
EPI_ISL_1396023	Laboratorio Central, Ministerio de Salud Cordoba	Instituto de Patologia Vegetal (CIAP-INTA) on behalf of 'Proyecto Argentino Interinstitucional de genomica de SARS-CoV-2' (PAIS Consortium)	Fernández et al
EPI_ISL_1395968	Laboratorio Central, Ministerio de Salud Cordoba	Instituto de Patologia Vegetal (CIAP-INTA) on behalf of 'Proyecto Argentino Interinstitucional de genomica de SARS-CoV-2' (PAIS Consortium)	Fernández et al
EPI_ISL_1396076	Laboratorio de Salud Pública	Instituto de Patologia Vegetal (CIAP-INTA) on behalf of 'Proyecto Argentino Interinstitucional de genomica de SARS-CoV-2' (PAIS Consortium)	Fernández et al
EPI_ISL_1469606	Fundação Hospitalar de Sapucaia do Sul	Epiclin	Fernando Hayashi Sant'Anna et al
EPI_ISL_1469805	Fundação Hospitalar de Sapucaia do Sul	Epiclin	Fernando Hayashi Sant'Anna et al
EPI_ISL_1469703	Unidade de Pronto Atendimento de Sapucaia do Sul	Epiclin	Fernando Hayashi Sant'Anna et al
EPI_ISL_1469787	Fundação Hospitalar de Sapucaia do Sul	Epiclin	Fernando Hayashi Sant'Anna et al
EPI_ISL_1469695	Unidade de Pronto Atendimento de Sapucaia do Sul	Epiclin	Fernando Hayashi Sant'Anna et al
EPI_ISL_1469599	Hospital Montenegro	Epiclin	Fernando Hayashi Sant'Anna et al
EPI_ISL_1469799	Hospital Regina	Epiclin	Fernando Hayashi Sant'Anna et al
EPI_ISL_1469817	Fundação Hospitalar de Sapucaia do Sul	Epiclin	Fernando Hayashi Sant'Anna et al
EPI_ISL_1469551	Secretaria Municipal de Saúde de São Leopoldo	Epiclin	Fernando Hayashi Sant'Anna et al
EPI_ISL_1469727	Secretaria Municipal de Saúde de São Leopoldo	Epiclin	Fernando Hayashi Sant'Anna et al
EPI_ISL_1469600	Hospital de Portão	Epiclin	Fernando Hayashi Sant'Anna et al
EPI_ISL_1469668	Hospital Unimed Vale do Caí	Epiclin	Fernando Hayashi Sant'Anna et al
EPI_ISL_1469654	Fundação Hospitalar de Sapucaia do Sul	Epiclin	Fernando Hayashi Sant'Anna et al
EPI_ISL_1469773	Hospital Universitário	Epiclin	Fernando Hayashi Sant'Anna et al
EPI_ISL_1469729	Centro de Especialidades Triunfo	Epiclin	Fernando Hayashi Sant'Anna et al
EPI_ISL_1469831	Fundação Hospitalar de Sapucaia do Sul	Epiclin	Fernando Hayashi Sant'Anna et al
EPI_ISL_1469849	Hospital Universitário de Canoas	Epiclin	Fernando Hayashi Sant'Anna et al

EPI_ISL_1469827	Hospital de Portão	Epiclin	Fernando Hayashi Sant'Anna et al
EPI_ISL_1469752	Fundação Hospitalar de Sapucaia do Sul	Epiclin	Fernando Hayashi Sant'Anna et al
EPI_ISL_1469844	Hospital Universitário de Canoas	Epiclin	Fernando Hayashi Sant'Anna et al
EPI_ISL_1469671	Vigilância em Saúde de Sapucaia do Sul	Epiclin	Fernando Hayashi Sant'Anna et al
EPI_ISL_1469724	Fundação Hospitalar de Sapucaia do Sul	Epiclin	Fernando Hayashi Sant'Anna et al
EPI_ISL_1469621	Pronto Atendimento Campo Bom	Epiclin	Fernando Hayashi Sant'Anna et al
EPI_ISL_1469824	Hospital Regina	Epiclin	Fernando Hayashi Sant'Anna et al
EPI_ISL_1469807	Hospital São Francisco de Assis	Epiclin	Fernando Hayashi Sant'Anna et al
EPI_ISL_1469585	Fundação Hospitalar de Sapucaia do Sul	Epiclin	Fernando Hayashi Sant'Anna et al
EPI_ISL_1469842	Hospital São Francisco de Assis	Epiclin	Fernando Hayashi Sant'Anna et al
EPI_ISL_1469837	Fundação de Saúde Pública São Camilo de Esteio	Epiclin	Fernando Hayashi Sant'Anna et al
EPI_ISL_1469561	Fundação de Saúde Pública São Camilo de Esteio	Epiclin	Fernando Hayashi Sant'Anna et al
EPI_ISL_1469581	Hospital Dia e Pronto Atendimento	Epiclin	Fernando Hayashi Sant'Anna et al
EPI_ISL_1469847	Secretaria Municipal de Saúde de Três Coroas	Epiclin	Fernando Hayashi Sant'Anna et al
EPI_ISL_1469838	Secretaria Municipal de Saúde de Três Coroas	Epiclin	Fernando Hayashi Sant'Anna et al
EPI_ISL_1469666	Secretaria Municipal de Saúde de Montenegro	Epiclin	Fernando Hayashi Sant'Anna et al
EPI_ISL_1469825	Pronto Atendimento Campo Bom	Epiclin	Fernando Hayashi Sant'Anna et al
EPI_ISL_1469615	Pronto Atendimento Campo Bom	Epiclin	Fernando Hayashi Sant'Anna et al
EPI_ISL_1469670	Hospital São Francisco de Assis	Epiclin	Fernando Hayashi Sant'Anna et al
EPI_ISL_1469567	Unidade Sanitária de Igrejinha	Epiclin	Fernando Hayashi Sant'Anna et al
EPI_ISL_1469830	Hospital São Francisco de Assis	Epiclin	Fernando Hayashi Sant'Anna et al
EPI_ISL_1469552	Unidade Sanitária de Igrejinha	Epiclin	Fernando Hayashi Sant'Anna et al
EPI_ISL_1469569	Fundação de Saúde Pública São Camilo de Esteio	Epiclin	Fernando Hayashi Sant'Anna et al
EPI_ISL_1469771	Hospital de Portão	Epiclin	Fernando Hayashi Sant'Anna et al
EPI_ISL_1469667	Centro de Referência em Síndromes Gripais	Epiclin	Fernando Hayashi Sant'Anna et al
EPI_ISL_1469794	Hospital Dia e Pronto Atendimento	Epiclin	Fernando Hayashi Sant'Anna et al
EPI_ISL_1469835	Diretoria de Vigilância em Saúde	Epiclin	Fernando Hayashi Sant'Anna et al
EPI_ISL_1469609	Diretoria de Vigilância em Saúde	Epiclin	Fernando Hayashi Sant'Anna et al
EPI_ISL_1469769	Diretoria de Vigilância em Saúde	Epiclin	Fernando Hayashi Sant'Anna et al
EPI_ISL_1469560	Diretoria de Vigilância em Saúde	Epiclin	Fernando Hayashi Sant'Anna et al

EPI_ISL_1469557	Centro de Serviços Especializados Santa Rita	Epiclin	Fernando Hayashi Sant’Anna et al
EPI_ISL_1469790	Unidade de Pronto Atendimento de Sapucaia do Sul	Epiclin	Fernando Hayashi Sant’Anna et al
EPI_ISL_1469602	Pronto Atendimento Campo Bom	Epiclin	Fernando Hayashi Sant’Anna et al
EPI_ISL_1469614	Unidade Sanitária de Igrejinha	Epiclin	Fernando Hayashi Sant’Anna et al
EPI_ISL_1469562	Hospital Dia e Pronto Atendimento	Epiclin	Fernando Hayashi Sant’Anna et al
EPI_ISL_1469616	Diretoria de Vigilância em Saúde	Epiclin	Fernando Hayashi Sant’Anna et al
EPI_ISL_1469850	Fundação de Saúde Pública São Camilo de Esteio	Epiclin	Fernando Hayashi Sant’Anna et al
EPI_ISL_1469809	Fundação Hospitalar de Sapucaia do Sul	Epiclin	Fernando Hayashi Sant’Anna et al
EPI_ISL_1469845	Fundação Hospitalar de Sapucaia do Sul	Epiclin	Fernando Hayashi Sant’Anna et al
EPI_ISL_1469582	Unidade de Pronto Atendimento de Sapucaia do Sul	Epiclin	Fernando Hayashi Sant’Anna et al
EPI_ISL_1469810	Unidade de Pronto Atendimento de Sapucaia do Sul	Epiclin	Fernando Hayashi Sant’Anna et al
EPI_ISL_1469647	Secretaria Municipal de Saúde de Taquara	Epiclin	Fernando Hayashi Sant’Anna et al
EPI_ISL_1469576	Unidade Sanitária de Igrejinha	Epiclin	Fernando Hayashi Sant’Anna et al
EPI_ISL_1479120	Diretoria de Vigilância em Saúde	Epiclin	Fernando Hayashi Sant’Anna et al
EPI_ISL_1469669	Diretoria de Vigilância em Saúde	Epiclin	Fernando Hayashi Sant’Anna et al
EPI_ISL_1469623	Diretoria de Vigilância em Saúde	Epiclin	Fernando Hayashi Sant’Anna et al
EPI_ISL_1469619	Fundação Hospitalar de Sapucaia do Sul	Epiclin	Fernando Hayashi Sant’Anna et al
EPI_ISL_1469707	Hospital Bom Jesus	Epiclin	Fernando Hayashi Sant’Anna et al
EPI_ISL_1469811	Hospital Municipal Getúlio Vargas	Epiclin	Fernando Hayashi Sant’Anna et al
EPI_ISL_1469556	Secretaria Municipal de Saúde de São Leopoldo	Epiclin	Fernando Hayashi Sant’Anna et al
EPI_ISL_1469617	Fundação Hospitalar de Sapucaia do Sul	Epiclin	Fernando Hayashi Sant’Anna et al
EPI_ISL_1469618	Diretoria de Vigilância em Saúde	Epiclin	Fernando Hayashi Sant’Anna et al
EPI_ISL_1469820	Hospital Sapiranga	Epiclin	Fernando Hayashi Sant’Anna et al
EPI_ISL_1479127	Hospital Regina	Epiclin	Fernando Hayashi Sant’Anna et al
EPI_ISL_1469795	Hospital Dia e Pronto Atendimento	Epiclin	Fernando Hayashi Sant’Anna et al
EPI_ISL_1469818	Hospital São Francisco de Assis	Epiclin	Fernando Hayashi Sant’Anna et al
EPI_ISL_1469595	Fundação de Saúde Pública de Novo Hamburgo	Epiclin	Fernando Hayashi Sant’Anna et al
EPI_ISL_1469741	Centro de Referência em Síndromes Gripais	Epiclin	Fernando Hayashi Sant’Anna et al
EPI_ISL_1469800	Fundação de Saúde Pública de Novo Hamburgo	Epiclin	Fernando Hayashi Sant’Anna et al
EPI_ISL_1469563	Unidade Básica de Saúde de Riozinho	Epiclin	Fernando Hayashi Sant’Anna et al

EPI_ISL_1469797	Diretoria de Vigilância em Saúde	Epiclin	Fernando Hayashi Sant’Anna et al
EPI_ISL_1469793	Hospital Universitário de Canoas	Epiclin	Fernando Hayashi Sant’Anna et al
EPI_ISL_1469650	Diretoria de Vigilância em Saúde	Epiclin	Fernando Hayashi Sant’Anna et al
EPI_ISL_1469653	Fundação de Saúde Pública de Novo Hamburgo	Epiclin	Fernando Hayashi Sant’Anna et al
EPI_ISL_1469696	Vigilância em Saúde de Sapucaia do Sul	Epiclin	Fernando Hayashi Sant’Anna et al
EPI_ISL_1469605	Diretoria de Vigilância em Saúde	Epiclin	Fernando Hayashi Sant’Anna et al
EPI_ISL_1469713	Fundação Hospitalar de Sapucaia do Sul	Epiclin	Fernando Hayashi Sant’Anna et al
EPI_ISL_1469607	Secretaria Municipal de Saúde de Três Coroas	Epiclin	Fernando Hayashi Sant’Anna et al
EPI_ISL_1469789	Hospital São Francisco de Assis	Epiclin	Fernando Hayashi Sant’Anna et al
EPI_ISL_1469783	Diretoria de Vigilância em Saúde	Epiclin	Fernando Hayashi Sant’Anna et al
EPI_ISL_1469756	Diretoria de Vigilância em Saúde	Epiclin	Fernando Hayashi Sant’Anna et al
EPI_ISL_1469593	Hospital Sapiranga	Epiclin	Fernando Hayashi Sant’Anna et al
EPI_ISL_1469749	Centro de Referência em Síndromes Gripais	Epiclin	Fernando Hayashi Sant’Anna et al
EPI_ISL_1469770	Fundação de Saúde Pública de Novo Hamburgo	Epiclin	Fernando Hayashi Sant’Anna et al
EPI_ISL_1469711	Hospital Dia e Pronto Atendimento	Epiclin	Fernando Hayashi Sant’Anna et al
EPI_ISL_1469803	Secretaria Municipal de Saúde de Três Coroas	Epiclin	Fernando Hayashi Sant’Anna et al
EPI_ISL_1469786	Diretoria de Vigilância em Saúde	Epiclin	Fernando Hayashi Sant’Anna et al
EPI_ISL_1479129	Pronto Atendimento Campo Bom	Epiclin	Fernando Hayashi Sant’Anna et al
EPI_ISL_1469550	Vigilância em Saúde de Sapucaia do Sul	Epiclin	Fernando Hayashi Sant’Anna et al
EPI_ISL_1469777	Centro de Referência em Síndromes Gripais	Epiclin	Fernando Hayashi Sant’Anna et al
EPI_ISL_1469714	Unidade de Atendimento DST AIDS TB e Han	Epiclin	Fernando Hayashi Sant’Anna et al
EPI_ISL_1469554	Fundação de Saúde Pública São Camilo de Esteio	Epiclin	Fernando Hayashi Sant’Anna et al
EPI_ISL_1469778	Hospital Municipal Getúlio Vargas	Epiclin	Fernando Hayashi Sant’Anna et al
EPI_ISL_1469846	Hospital Nossa Senhora das Graças	Epiclin	Fernando Hayashi Sant’Anna et al
EPI_ISL_1469821	Centro de Saúde Bruno Cassel	Epiclin	Fernando Hayashi Sant’Anna et al
EPI_ISL_1469736	SECRETARIA MUNICIPAL DE SAUDE DE SAO LEOPOLDO	Epiclin	Fernando Hayashi Sant’Anna et al
EPI_ISL_1469575	HOSPITAL SAO FRANCISCO DE ASSIS	Epiclin	Fernando Hayashi Sant’Anna et al
EPI_ISL_1469663	DIRETORIA DE VIGILANCIA EM SAUDE	Epiclin	Fernando Hayashi Sant’Anna et al
EPI_ISL_1469590	DIRETORIA DE VIGILANCIA EM SAUDE	Epiclin	Fernando Hayashi Sant’Anna et al
EPI_ISL_1469681	DIRETORIA DE VIGILANCIA EM SAUDE	Epiclin	Fernando Hayashi Sant’Anna et al

EPI_ISL_1469751	UNIDADE BASICA DE SAUDE DE RIOZINHO	Epiclin	Fernando Hayashi Sant’Anna et al
EPI_ISL_1469826	Fundação de Saúde Pública de Novo Hamburgo	Epiclin	Fernando Hayashi Sant’Anna et al
EPI_ISL_1469659	DIRETORIA DE VIGILANCIA EM SAUDE	Epiclin	Fernando Hayashi Sant’Anna et al
EPI_ISL_1469767	Fundação de Saúde Pública de Novo Hamburgo	Epiclin	Fernando Hayashi Sant’Anna et al
EPI_ISL_1469558	DIRETORIA DE VIGILANCIA EM SAUDE	Epiclin	Fernando Hayashi Sant’Anna et al
EPI_ISL_1469804	UNIDADE DE PRONTO ATENDIMENTO DE SAPUCAIA DO SUL UPA	Epiclin	Fernando Hayashi Sant’Anna et al
EPI_ISL_1469583	UNIDADE SANITARIA DE IGREJINHA	Epiclin	Fernando Hayashi Sant’Anna et al
EPI_ISL_1469792	Diretoria de Vigilância em Saúde	Epiclin	Fernando Hayashi Sant’Anna et al
EPI_ISL_1469708	Diretoria de Vigilância em Saúde	Epiclin	Fernando Hayashi Sant’Anna et al
EPI_ISL_1469731	DIRETORIA DE VIGILANCIA EM SAUDE	Epiclin	Fernando Hayashi Sant’Anna et al
EPI_ISL_1469784	DIRETORIA DE VIGILANCIA EM SAUDE	Epiclin	Fernando Hayashi Sant’Anna et al
EPI_ISL_1469813	HOSPITAL BOM JESUS	Epiclin	Fernando Hayashi Sant’Anna et al
EPI_ISL_1469664	Unidade de Atendimento DST AIDS TB e Han	Epiclin	Fernando Hayashi Sant’Anna et al
EPI_ISL_1469662	CENTRO DE SAUDE DR BRUNO CASSEL	Epiclin	Fernando Hayashi Sant’Anna et al
EPI_ISL_1469594	DIRETORIA DE VIGILANCIA EM SAUDE	Epiclin	Fernando Hayashi Sant’Anna et al
EPI_ISL_1469684	DIRETORIA DE VIGILANCIA EM SAUDE	Epiclin	Fernando Hayashi Sant’Anna et al
EPI_ISL_1469620	FUNDACAO DE SAUDE PUBLICA DE NOVO HAMBURGO FSNH	Epiclin	Fernando Hayashi Sant’Anna et al
EPI_ISL_1469571	Unidade de Pronto Atendimento de Sapucaia do Sul	Epiclin	Fernando Hayashi Sant’Anna et al
EPI_ISL_1469698	CENTRO DE SERVICOS ESPECIALIZADOS SANTA RITA DE CASSIA	Epiclin	Fernando Hayashi Sant’Anna et al
EPI_ISL_1469596	CENTRO DE REFERENCIA EM SINDROMES GRIPAIS	Epiclin	Fernando Hayashi Sant’Anna et al
EPI_ISL_1469655	Centro Municipal de Saúde de Rolante	Epiclin	Fernando Hayashi Sant’Anna et al
EPI_ISL_1469734	Hospital Dia e Pronto Atendimento	Epiclin	Fernando Hayashi Sant’Anna et al
EPI_ISL_1469676	HOSPITAL SAO FRANCISCO DE ASSIS	Epiclin	Fernando Hayashi Sant’Anna et al
EPI_ISL_1469692	DIRETORIA DE VIGILANCIA EM SAUDE	Epiclin	Fernando Hayashi Sant’Anna et al
EPI_ISL_1469679	DIRETORIA DE VIGILANCIA EM SAUDE	Epiclin	Fernando Hayashi Sant’Anna et al
EPI_ISL_1469629	CENTRO MUNICIPAL DE SAUDE DE ROLANTE	Epiclin	Fernando Hayashi Sant’Anna et al
EPI_ISL_1469685	HOSPITAL SAO FRANCISCO DE ASSIS	Epiclin	Fernando Hayashi Sant’Anna et al
EPI_ISL_1469686	DIRETORIA DE VIGILANCIA EM SAUDE	Epiclin	Fernando Hayashi Sant’Anna et al
EPI_ISL_1469710	DIRETORIA DE VIGILANCIA EM SAUDE	Epiclin	Fernando Hayashi Sant’Anna et al
EPI_ISL_1469579	DIRETORIA DE VIGILANCIA EM SAUDE	Epiclin	Fernando Hayashi Sant’Anna et al

EPI_ISL_1469680	UNIDADE DE ATENDIMENTO DST AIDS TB E HAN	Epiclin	Fernando Hayashi Sant’Anna et al
EPI_ISL_1479126	UNIDADE DE ATENDIMENTO DST AIDS TB E HAN	Epiclin	Fernando Hayashi Sant’Anna et al
EPI_ISL_1469572	FUNDACAO DE SAUDE PUBLICA DE NOVO HAMBURGO FSNH	Epiclin	Fernando Hayashi Sant’Anna et al
EPI_ISL_1469754	Hospital Municipal Getúlio Vargas	Epiclin	Fernando Hayashi Sant’Anna et al
EPI_ISL_1469768	UNIDADE DE PRONTO ATENDIMENTO DE SAPUCAIA DO SUL UPA	Epiclin	Fernando Hayashi Sant’Anna et al
EPI_ISL_1469651	FUNDACAO DE SAUDE PUBLICA DE NOVO HAMBURGO FSNH	Epiclin	Fernando Hayashi Sant’Anna et al
EPI_ISL_1469566	DIRETORIA DE VIGILANCIA EM SAUDE	Epiclin	Fernando Hayashi Sant’Anna et al
EPI_ISL_1469775	Diretoria de Vigilância em Saúde	Epiclin	Fernando Hayashi Sant’Anna et al
EPI_ISL_1469791	DIRETORIA DE VIGILANCIA EM SAUDE	Epiclin	Fernando Hayashi Sant’Anna et al
EPI_ISL_1469603	Centro de Referência em Síndromes Gripais	Epiclin	Fernando Hayashi Sant’Anna et al
EPI_ISL_1469753	Diretoria de Vigilância em Saúde	Epiclin	Fernando Hayashi Sant’Anna et al
EPI_ISL_1469592	Diretoria de Vigilância em Saúde	Epiclin	Fernando Hayashi Sant’Anna et al
EPI_ISL_1469633	DIRETORIA DE VIGILANCIA EM SAUDE	Epiclin	Fernando Hayashi Sant’Anna et al
EPI_ISL_1469584	CENTRO MUNICIPAL DE SAUDE DE ROLANTE	Epiclin	Fernando Hayashi Sant’Anna et al
EPI_ISL_1469549	FUNDACAO HOSPITALAR DE SAPUCAIA DO SUL	Epiclin	Fernando Hayashi Sant’Anna et al
EPI_ISL_1469673	Fundação de Saúde Pública de Novo Hamburgo	Epiclin	Fernando Hayashi Sant’Anna et al
EPI_ISL_1469674	DIRETORIA DE VIGILANCIA EM SAUDE	Epiclin	Fernando Hayashi Sant’Anna et al
EPI_ISL_1479123	Fundação de Saúde Pública de Novo Hamburgo	Epiclin	Fernando Hayashi Sant’Anna et al
EPI_ISL_1469630	FUNDACAO DE SAUDE PUBLICA DE NOVO HAMBURGO FSNH	Epiclin	Fernando Hayashi Sant’Anna et al
EPI_ISL_1469578	HOSPITAL SAO FRANCISCO DE ASSIS	Epiclin	Fernando Hayashi Sant’Anna et al
EPI_ISL_1469742	CENTRO DE REFERENCIA EM SINDROMES GRIPAIS	Epiclin	Fernando Hayashi Sant’Anna et al
EPI_ISL_1469688	DIRETORIA DE VIGILANCIA EM SAUDE	Epiclin	Fernando Hayashi Sant’Anna et al
EPI_ISL_1469725	DIRETORIA DE VIGILANCIA EM SAUDE	Epiclin	Fernando Hayashi Sant’Anna et al
EPI_ISL_1469702	Diretoria de Vigilância em Saúde	Epiclin	Fernando Hayashi Sant’Anna et al
EPI_ISL_1469761	Diretoria de Vigilância em Saúde	Epiclin	Fernando Hayashi Sant’Anna et al
EPI_ISL_1469694	UNIDADE BASICA DE SAUDE PARECI NOVO	Epiclin	Fernando Hayashi Sant’Anna et al
EPI_ISL_1469836	Diretoria de Vigilância em Saúde	Epiclin	Fernando Hayashi Sant’Anna et al
EPI_ISL_1469705	HOSPITAL DE CAMPO BOM DR LAURO REUS	Epiclin	Fernando Hayashi Sant’Anna et al
EPI_ISL_1479133	HOSPITAL SAO FRANCISCO DE ASSIS	Epiclin	Fernando Hayashi Sant’Anna et al
EPI_ISL_1469631	HOSPITAL MUNICIPAL GETULIO VARGAS	Epiclin	Fernando Hayashi Sant’Anna et al

EPI_ISL_1469738	HOSPITAL SAO FRANCISCO DE ASSIS	Epiclin	Fernando Hayashi Sant'Anna et al
EPI_ISL_1469604	COORDENADORIA GERAL DE VIGILANCIA EM SAUDE	Epiclin	Fernando Hayashi Sant'Anna et al
EPI_ISL_1469745	UNIDADE DE PRONTO ATENDIMENTO DE SAPUCAIA DO SUL UPA	Epiclin	Fernando Hayashi Sant'Anna et al
EPI_ISL_1469687	SECRETARIA MUNICIPAL DE SAUDE DE TAQUARA	Epiclin	Fernando Hayashi Sant'Anna et al
EPI_ISL_1469781	DIRETORIA DE VIGILANCIA EM SAUDE	Epiclin	Fernando Hayashi Sant'Anna et al
EPI_ISL_1469661	FUNDACAO DE SAUDE PUBLICA DE NOVO HAMBURGO FSNH	Epiclin	Fernando Hayashi Sant'Anna et al
EPI_ISL_1469726	HOSPITAL SAPIRANGA	Epiclin	Fernando Hayashi Sant'Anna et al
EPI_ISL_1479125	CENTRO DE REFERENCIA EM SINDROMES GRIPAIS	Epiclin	Fernando Hayashi Sant'Anna et al
EPI_ISL_1469802	Diretoria de Vigilância em Saúde	Epiclin	Fernando Hayashi Sant'Anna et al
EPI_ISL_1469785	FUNDACAO DE SAUDE PUBLICA DE NOVO HAMBURGO FSNH	Epiclin	Fernando Hayashi Sant'Anna et al
EPI_ISL_1195293	SECRETARIA MUNICIPAL DE SAUDE DE TAQUARA	Epiclin	Fernando Hayashi Sant'Anna et al
EPI_ISL_1469709	SECRETARIA MUNICIPAL DE SAUDE DE TAQUARA	Epiclin	Fernando Hayashi Sant'Anna et al
EPI_ISL_1469646	UNIDADE DE PRONTO ATENDIMENTO DE SAPUCAIA DO SUL UPA	Epiclin	Fernando Hayashi Sant'Anna et al
EPI_ISL_1479128	HOSPITAL SAO FRANCISCO DE ASSIS	Epiclin	Fernando Hayashi Sant'Anna et al
EPI_ISL_1469580	Unidade de Pronto Atendimento de Sapucaia do Sul	Epiclin	Fernando Hayashi Sant'Anna et al
EPI_ISL_1469766	Coordenadoria Geral de Vigilância em Saúde · Vigilância em Saúde	Epiclin	Fernando Hayashi Sant'Anna et al
EPI_ISL_1469635	COORDENADORIA GERAL DE VIGILANCIA EM SAUDE	Epiclin	Fernando Hayashi Sant'Anna et al
EPI_ISL_1469677	DIRETORIA DE VIGILANCIA EM SAUDE	Epiclin	Fernando Hayashi Sant'Anna et al
EPI_ISL_1469589	UNIDADE DE PRONTO ATENDIMENTO DE SAPUCAIA DO SUL UPA	Epiclin	Fernando Hayashi Sant'Anna et al
EPI_ISL_1469665	FUNDACAO DE SAUDE PUBLICA SAO CAMILO DE ESTEIO	Epiclin	Fernando Hayashi Sant'Anna et al
EPI_ISL_1469814	SECRETARIA MUNICIPAL DE SAUDE DE SAO LEOPOLDO	Epiclin	Fernando Hayashi Sant'Anna et al
EPI_ISL_1469690	SECRETARIA MUNICIPAL DE SAUDE DE SAO LEOPOLDO	Epiclin	Fernando Hayashi Sant'Anna et al
EPI_ISL_1469624	HOSPITAL MUNICIPAL GETULIO VARGAS	Epiclin	Fernando Hayashi Sant'Anna et al
EPI_ISL_1469683	SECRETARIA MUNICIPAL DE SAUDE DE TAQUARA	Epiclin	Fernando Hayashi Sant'Anna et al
EPI_ISL_1469675	DIRETORIA DE VIGILANCIA EM SAUDE	Epiclin	Fernando Hayashi Sant'Anna et al
EPI_ISL_1469645	SECRETARIA MUNICIPAL DE SAUDE DE TRES COROAS	Epiclin	Fernando Hayashi Sant'Anna et al
EPI_ISL_1469637	DIRETORIA DE VIGILANCIA EM SAUDE	Epiclin	Fernando Hayashi Sant'Anna et al
EPI_ISL_1469755	Hospital São Francisco de Assis	Epiclin	Fernando Hayashi Sant'Anna et al
EPI_ISL_1469648	HOSPITAL SAO FRANCISCO DE ASSIS	Epiclin	Fernando Hayashi Sant'Anna et al
EPI_ISL_1469642	CENTRO DE REFERENCIA EM SINDROMES GRIPAIS	Epiclin	Fernando Hayashi Sant'Anna et al

EPI_ISL_1469823	Diretoria de Vigilância em Saúde	Epiclin	Fernando Hayashi Sant'Anna et al
EPI_ISL_1479130	SECRETARIA MUNICIPAL DE SAUDE DE TAQUARA	Epiclin	Fernando Hayashi Sant'Anna et al
EPI_ISL_1469628	CENTRO DE SERVICOS ESPECIALIZADOS SANTA RITA DE CASSIA	Epiclin	Fernando Hayashi Sant'Anna et al
EPI_ISL_1469573	VIGILANCIA EM SAUDE NH	Epiclin	Fernando Hayashi Sant'Anna et al
EPI_ISL_1469733	UNIDADE DE PRONTO ATENDIMENTO DE SAPUCAIA DO SUL UPA	Epiclin	Fernando Hayashi Sant'Anna et al
EPI_ISL_1469735	COORDENADORIA GERAL DE VIGILANCIA EM SAUDE	Epiclin	Fernando Hayashi Sant'Anna et al
EPI_ISL_1469715	COORDENADORIA GERAL DE VIGILANCIA EM SAUDE	Epiclin	Fernando Hayashi Sant'Anna et al
EPI_ISL_1469782	COORDENADORIA GERAL DE VIGILANCIA EM SAUDE	Epiclin	Fernando Hayashi Sant'Anna et al
EPI_ISL_1469601	SECRETARIA MUNICIPAL DE SAUDE DE TAQUARA	Epiclin	Fernando Hayashi Sant'Anna et al
EPI_ISL_1469730	CENTRO DE REFERENCIA EM SINDROMES GRIPAIS	Epiclin	Fernando Hayashi Sant'Anna et al
EPI_ISL_1469701	Hospital Sapiranga	Epiclin	Fernando Hayashi Sant'Anna et al
EPI_ISL_1469611	DIRETORIA DE VIGILANCIA EM SAUDE	Epiclin	Fernando Hayashi Sant'Anna et al
EPI_ISL_1469765	UNIDADE SANITARIA DE IGREJINHA	Epiclin	Fernando Hayashi Sant'Anna et al
EPI_ISL_1469722	UNIDADE SANITARIA DE IGREJINHA	Epiclin	Fernando Hayashi Sant'Anna et al
EPI_ISL_1469759	HOSPITAL MUNICIPAL GETULIO VARGAS	Epiclin	Fernando Hayashi Sant'Anna et al
EPI_ISL_1469691	Fundação de Saúde Pública São Camilo de Esteio	Epiclin	Fernando Hayashi Sant'Anna et al
EPI_ISL_1479121	DIRETORIA DE VIGILANCIA EM SAUDE	Epiclin	Fernando Hayashi Sant'Anna et al
EPI_ISL_1469632	Diretoria de Vigilância em Saúde	Epiclin	Fernando Hayashi Sant'Anna et al
EPI_ISL_1469586	FUNDACAO HOSPITALAR SAO JOSE	Epiclin	Fernando Hayashi Sant'Anna et al
EPI_ISL_1469643	HOSPITAL SAO FRANCISCO DE ASSIS	Epiclin	Fernando Hayashi Sant'Anna et al
EPI_ISL_1195273	FUNDACAO DE SAUDE PUBLICA SAO CAMILO DE ESTEIO	Epiclin	Fernando Hayashi Sant'Anna et al
EPI_ISL_1479131	Fundação de Saúde Pública de Novo Hamburgo	Epiclin	Fernando Hayashi Sant'Anna et al
EPI_ISL_1469721	Pronto Atendimento Cruzeiro do Sul	Epiclin	Fernando Hayashi Sant'Anna et al
EPI_ISL_1479132	Fundação de Saúde Pública de Novo Hamburgo	Epiclin	Fernando Hayashi Sant'Anna et al
EPI_ISL_1479119	DIRETORIA DE VIGILANCIA EM SAUDE	Epiclin	Fernando Hayashi Sant'Anna et al
EPI_ISL_1195279	CENTRO DE REFERENCIA EM SINDROMES GRIPAIS	Epiclin	Fernando Hayashi Sant'Anna et al
EPI_ISL_1195280	SECRETARIA MUNICIPAL DE SAUDE DE ARARICA	Epiclin	Fernando Hayashi Sant'Anna et al
EPI_ISL_1479124	UNIDADE DE SAUDE NOVA HARTZ	Epiclin	Fernando Hayashi Sant'Anna et al
EPI_ISL_1195292	SECRETARIA MUNICIPAL DE SAUDE DE TRES COROAS	Epiclin	Fernando Hayashi Sant'Anna et al
EPI_ISL_1469720	UNIDADE SANITARIA DE IGREJINHA	Epiclin	Fernando Hayashi Sant'Anna et al

EPI_ISL_1469574	CENTRO DE SERVICOS ESPECIALIZADOS SANTA RITA DE CASSIA	Epiclin	Fernando Hayashi Sant'Anna et al
EPI_ISL_1469652	DIRETORIA DE VIGILANCIA EM SAUDE	Epiclin	Fernando Hayashi Sant'Anna et al
EPI_ISL_1469612	FUNDACAO DE SAUDE PUBLICA DE NOVO HAMBURGO FSNH	Epiclin	Fernando Hayashi Sant'Anna et al
EPI_ISL_1195291	DIRETORIA DE VIGILANCIA EM SAUDE	Epiclin	Fernando Hayashi Sant'Anna et al
EPI_ISL_1469723	FUNDACAO DE SAUDE PUBLICA SAO CAMILO DE ESTEIO	Epiclin	Fernando Hayashi Sant'Anna et al
EPI_ISL_1469772	DIRETORIA DE VIGILANCIA EM SAUDE	Epiclin	Fernando Hayashi Sant'Anna et al
EPI_ISL_1469848	Diretoria de Vigilância em Saúde	Epiclin	Fernando Hayashi Sant'Anna et al
EPI_ISL_1195290	SECRETARIA MUNICIPAL DE SAUDE DE TRES COROAS	Epiclin	Fernando Hayashi Sant'Anna et al
EPI_ISL_1469798	DIRETORIA DE VIGILANCIA EM SAUDE	Epiclin	Fernando Hayashi Sant'Anna et al
EPI_ISL_1469764	DIRETORIA DE VIGILANCIA EM SAUDE	Epiclin	Fernando Hayashi Sant'Anna et al
EPI_ISL_1469760	DIRETORIA DE VIGILANCIA EM SAUDE	Epiclin	Fernando Hayashi Sant'Anna et al
EPI_ISL_1469699	HOSPITAL MUNICIPAL GETULIO VARGAS	Epiclin	Fernando Hayashi Sant'Anna et al
EPI_ISL_1469834	HOSPITAL SAO FRANCISCO DE ASSIS	Epiclin	Fernando Hayashi Sant'Anna et al
EPI_ISL_1195278	DIRETORIA DE VIGILANCIA EM SAUDE	Epiclin	Fernando Hayashi Sant'Anna et al
EPI_ISL_1469740	SECRETARIA MUNICIPAL DE SAUDE DE TRES COROAS	Epiclin	Fernando Hayashi Sant'Anna et al
EPI_ISL_1469568	SECRETARIA MUNICIPAL DE SAUDE DE SAO LEOPOLDO	Epiclin	Fernando Hayashi Sant'Anna et al
EPI_ISL_1469555	FUNDACAO DE SAUDE PUBLICA DE NOVO HAMBURGO FSNH	Epiclin	Fernando Hayashi Sant'Anna et al
EPI_ISL_1195289	SECRETARIA MUNICIPAL DE SAUDE DE SAO LEOPOLDO	Epiclin	Fernando Hayashi Sant'Anna et al
EPI_ISL_1469719	UNIDADE DE PRONTO ATENDIMENTO DE SAPUCAIA DO SUL UPA	Epiclin	Fernando Hayashi Sant'Anna et al
EPI_ISL_1469822	Hospital São Francisco de Assis	Epiclin	Fernando Hayashi Sant'Anna et al
EPI_ISL_1469839	HOSPITAL DE CAMPO BOM DR LAURO REUS	Epiclin	Fernando Hayashi Sant'Anna et al
EPI_ISL_1469704	FUNDACAO DE SAUDE PUBLICA SAO CAMILO DE ESTEIO	Epiclin	Fernando Hayashi Sant'Anna et al
EPI_ISL_1469840	Hospital Sapiranga	Epiclin	Fernando Hayashi Sant'Anna et al
EPI_ISL_1195288	DIRETORIA DE VIGILANCIA EM SAUDE	Epiclin	Fernando Hayashi Sant'Anna et al
EPI_ISL_1469638	DIRETORIA DE VIGILANCIA EM SAUDE	Epiclin	Fernando Hayashi Sant'Anna et al
EPI_ISL_1469743	DIRETORIA DE VIGILANCIA EM SAUDE	Epiclin	Fernando Hayashi Sant'Anna et al
EPI_ISL_1469832	Diretoria de Vigilância em Saúde	Epiclin	Fernando Hayashi Sant'Anna et al
EPI_ISL_1195277	Unidade de Atendimento DST AIDS TB e Han	Epiclin	Fernando Hayashi Sant'Anna et al
EPI_ISL_1469774	Hospital Universitário de Canoas	Epiclin	Fernando Hayashi Sant'Anna et al
EPI_ISL_1469553	Diretoria de Vigilância em Saúde	Epiclin	Fernando Hayashi Sant'Anna et al

EPI_ISL_1469808	Diretoria de Vigilância em Saúde	Epiclin	Fernando Hayashi Sant'Anna et al
EPI_ISL_1469689	FUNDACAO DE SAUDE PUBLICA SAO CAMILO DE ESTEIO	Epiclin	Fernando Hayashi Sant'Anna et al
EPI_ISL_1195287	SECRETARIA MUNICIPAL DE SAUDE DE SAO LEOPOLDO	Epiclin	Fernando Hayashi Sant'Anna et al
EPI_ISL_1195274	SECRETARIA MUNICIPAL DE SAUDE DE TRES COROAS	Epiclin	Fernando Hayashi Sant'Anna et al
EPI_ISL_1469658	CENTRO DE ESPECIALIDADES TRIUNFO	Epiclin	Fernando Hayashi Sant'Anna et al
EPI_ISL_1469788	SECRETARIA MUNICIPAL DE SAUDE DE SAO LEOPOLDO	Epiclin	Fernando Hayashi Sant'Anna et al
EPI_ISL_1469660	UNIDADE BASICA DE SAUDE DE RIOZINHO	Epiclin	Fernando Hayashi Sant'Anna et al
EPI_ISL_1469815	Hospital Universitário	Epiclin	Fernando Hayashi Sant'Anna et al
EPI_ISL_1469776	Centro Municipal de Saúde de Rolante	Epiclin	Fernando Hayashi Sant'Anna et al
EPI_ISL_1469634	Diretoria de Vigilância em Saúde	Epiclin	Fernando Hayashi Sant'Anna et al
EPI_ISL_1469570	Hospital Universitário	Epiclin	Fernando Hayashi Sant'Anna et al
EPI_ISL_1195284	Centro de Especialidades Triunfo	Epiclin	Fernando Hayashi Sant'Anna et al
EPI_ISL_1469706	CENTRO DE SAUDE DR BRUNO CASSEL	Epiclin	Fernando Hayashi Sant'Anna et al
EPI_ISL_1469757	Secretaria Municipal de Saúde de São Leopoldo	Epiclin	Fernando Hayashi Sant'Anna et al
EPI_ISL_1469639	SECRETARIA MUNICIPAL DE SAUDE DE SAO LEOPOLDO	Epiclin	Fernando Hayashi Sant'Anna et al
EPI_ISL_1195285	SECRETARIA MUNICIPAL DE SAUDE DE TRES COROAS	Epiclin	Fernando Hayashi Sant'Anna et al
EPI_ISL_1195286	SECRETARIA MUNICIPAL DE SAUDE DE TRES COROAS	Epiclin	Fernando Hayashi Sant'Anna et al
EPI_ISL_1469641	COORDENADORIA GERAL DE VIGILANCIA EM SAUDE	Epiclin	Fernando Hayashi Sant'Anna et al
EPI_ISL_1469636	COORDENADORIA GERAL DE VIGILANCIA EM SAUDE	Epiclin	Fernando Hayashi Sant'Anna et al
EPI_ISL_1469608	COORDENADORIA GERAL DE VIGILANCIA EM SAUDE	Epiclin	Fernando Hayashi Sant'Anna et al
EPI_ISL_1195282	UNIDADE SANITARIA DE IGREJINHA	Epiclin	Fernando Hayashi Sant'Anna et al
EPI_ISL_1195272	FUNDACAO HOSPITALAR DE SAPUCAIA DO SUL	Epiclin	Fernando Hayashi Sant'Anna et al
EPI_ISL_1469693	FUNDACAO DE SAUDE PUBLICA SAO CAMILO DE ESTEIO	Epiclin	Fernando Hayashi Sant'Anna et al
EPI_ISL_1195281	FUNDACAO DE SAUDE PUBLICA SAO CAMILO DE ESTEIO	Epiclin	Fernando Hayashi Sant'Anna et al
EPI_ISL_1469746	HOSPITAL MONTENEGRO	Epiclin	Fernando Hayashi Sant'Anna et al
EPI_ISL_1195275	CENTRO DE REFERENCIA EM SINDROMES GRIPAIS	Epiclin	Fernando Hayashi Sant'Anna et al
EPI_ISL_1195276	CENTRO DE REFERENCIA EM SINDROMES GRIPAIS	Epiclin	Fernando Hayashi Sant'Anna et al
EPI_ISL_1469737	CENTRO DE REFERENCIA EM SINDROMES GRIPAIS	Epiclin	Fernando Hayashi Sant'Anna et al
EPI_ISL_1469732	CENTRO DE REFERENCIA EM SINDROMES GRIPAIS	Epiclin	Fernando Hayashi Sant'Anna et al
EPI_ISL_1469682	CENTRO DE SERVICOS ESPECIALIZADOS SANTA RITA DE CASSIA	Epiclin	Fernando Hayashi Sant'Anna et al

EPI_ISL_1469744	HOSPITAL SAO FRANCISCO DE ASSIS	Epiclin	Fernando Hayashi Sant'Anna et al
EPI_ISL_1469678	SECRETARIA MUNICIPAL DE SAUDE DE TRES COROAS	Epiclin	Fernando Hayashi Sant'Anna et al
EPI_ISL_1195283	SECRETARIA MUNICIPAL DE SAUDE DE TRES COROAS	Epiclin	Fernando Hayashi Sant'Anna et al
EPI_ISL_1469672	Diretoria de Vigilância em Saúde	Epiclin	Fernando Hayashi Sant'Anna et al
EPI_ISL_1469819	Diretoria de Vigilância em Saúde	Epiclin	Fernando Hayashi Sant'Anna et al
EPI_ISL_1469627	Diretoria de Vigilância em Saúde	Epiclin	Fernando Hayashi Sant'Anna et al
EPI_ISL_1469828	Fundação de Saúde Pública de Novo Hamburgo	Epiclin	Fernando Hayashi Sant'Anna et al
EPI_ISL_1469758	Secretaria Municipal de Saúde de Três Coroas	Epiclin	Fernando Hayashi Sant'Anna et al
EPI_ISL_1469812	Posto de Saúde Cambará do Sul	Epiclin	Fernando Hayashi Sant'Anna et al
EPI_ISL_1469843	Hospital Sapiranga	Epiclin	Fernando Hayashi Sant'Anna et al
EPI_ISL_1469779	Centro de Especialidades Triunfo	Epiclin	Fernando Hayashi Sant'Anna et al
EPI_ISL_1469841	Diretoria de Vigilância em Saúde	Epiclin	Fernando Hayashi Sant'Anna et al
EPI_ISL_1469763	Unidade Básica de Saúde de Riozinho	Epiclin	Fernando Hayashi Sant'Anna et al
EPI_ISL_1469598	Diretoria de Vigilância em Saúde	Epiclin	Fernando Hayashi Sant'Anna et al
EPI_ISL_1469588	Secretaria Municipal de Saúde de Taquara	Epiclin	Fernando Hayashi Sant'Anna et al
EPI_ISL_1469806	Centro de Referência em Síndromes Gripais	Epiclin	Fernando Hayashi Sant'Anna et al
EPI_ISL_1469829	Diretoria de Vigilância em Saúde	Epiclin	Fernando Hayashi Sant'Anna et al
EPI_ISL_1469801	Hospital Sapiranga	Epiclin	Fernando Hayashi Sant'Anna et al
EPI_ISL_1469610	Diretoria de Vigilância em Saúde	Epiclin	Fernando Hayashi Sant'Anna et al
EPI_ISL_1469748	Diretoria de Vigilância em Saúde	Epiclin	Fernando Hayashi Sant'Anna et al
EPI_ISL_1469697	Hospital Regina	Epiclin	Fernando Hayashi Sant'Anna et al
EPI_ISL_1479122	Fundação Hospitalar São José	Epiclin	Fernando Hayashi Sant'Anna et al
EPI_ISL_1469739	Hospital São Francisco de Assis	Epiclin	Fernando Hayashi Sant'Anna et al
EPI_ISL_1469762	Hospital Nossa Senhora das Graças	Epiclin	Fernando Hayashi Sant'Anna et al
EPI_ISL_1469656	Diretoria de Vigilância em Saúde	Epiclin	Fernando Hayashi Sant'Anna et al
EPI_ISL_1469796	Diretoria de Vigilância em Saúde	Epiclin	Fernando Hayashi Sant'Anna et al
EPI_ISL_1469587	Hospital Sapiranga	Epiclin	Fernando Hayashi Sant'Anna et al
EPI_ISL_1469644	Diretoria de Vigilância em Saúde	Epiclin	Fernando Hayashi Sant'Anna et al
EPI_ISL_1469613	Fundação de Saúde Pública de Novo Hamburgo	Epiclin	Fernando Hayashi Sant'Anna et al
EPI_ISL_1469816	Diretoria de Vigilância em Saúde	Epiclin	Fernando Hayashi Sant'Anna et al

EPI_ISL_1469780	Secretaria Municipal de Saúde de Taquara	Epiclin	Fernando Hayashi Sant'Anna et al
EPI_ISL_1469649	Unidade de Atendimento DST AIDS TB e Han	Epiclin	Fernando Hayashi Sant'Anna et al
EPI_ISL_1469640	Fundação de Saúde Pública São Camilo de Esteio	Epiclin	Fernando Hayashi Sant'Anna et al
EPI_ISL_1469591	Hospital Nossa Senhora das Graças	Epiclin	Fernando Hayashi Sant'Anna et al
EPI_ISL_1469626	Fundação de Saúde Pública de Novo Hamburgo	Epiclin	Fernando Hayashi Sant'Anna et al
EPI_ISL_1469622	Fundação de Saúde Pública de Novo Hamburgo	Epiclin	Fernando Hayashi Sant'Anna et al
EPI_ISL_1469716	Unidade Sanitária de Igrejinha	Epiclin	Fernando Hayashi Sant'Anna et al
EPI_ISL_1469851	Hospital Nossa Senhora das Graças	Epiclin	Fernando Hayashi Sant'Anna et al
EPI_ISL_1469717	Hospital São Francisco de Assis	Epiclin	Fernando Hayashi Sant'Anna et al
EPI_ISL_1469700	Diretoria de Vigilância em Saúde	Epiclin	Fernando Hayashi Sant'Anna et al
EPI_ISL_1469747	Unidade de Pronto Atendimento de Sapucaia do Sul	Epiclin	Fernando Hayashi Sant'Anna et al
EPI_ISL_1469577	Unidade de Pronto Atendimento de Sapucaia do Sul	Epiclin	Fernando Hayashi Sant'Anna et al
EPI_ISL_1469657	Pronto Atendimento Campo Bom	Epiclin	Fernando Hayashi Sant'Anna et al
EPI_ISL_1469750	Hospital São Francisco de Assis	Epiclin	Fernando Hayashi Sant'Anna et al
EPI_ISL_1469712	Pronto Atendimento Campo Bom	Epiclin	Fernando Hayashi Sant'Anna et al
EPI_ISL_1469833	Hospital Nossa Senhora das Graças	Epiclin	Fernando Hayashi Sant'Anna et al
EPI_ISL_1469625	Diretoria de Vigilância em Saúde	Epiclin	Fernando Hayashi Sant'Anna et al
EPI_ISL_1469728	Diretoria de Vigilância em Saúde	Epiclin	Fernando Hayashi Sant'Anna et al
EPI_ISL_1469718	Unidade de Pronto Atendimento de Sapucaia do Sul	Epiclin	Fernando Hayashi Sant'Anna et al
EPI_ISL_1469564	Pronto Atendimento Campo Bom	Epiclin	Fernando Hayashi Sant'Anna et al
EPI_ISL_1469559	Secretaria Municipal de Saúde de Três Coroas	Epiclin	Fernando Hayashi Sant'Anna et al
EPI_ISL_1469565	Unidade de Pronto Atendimento de Sapucaia do Sul	Epiclin	Fernando Hayashi Sant'Anna et al
EPI_ISL_1469597	Fundação de Saúde Pública de Novo Hamburgo	Epiclin	Fernando Hayashi Sant'Anna et al
EPI_ISL_426580	Instituto Sabin	Laboratory of Virology	Fernando L Melo et al
EPI_ISL_1323013	Hospital Universitario La Paz (Madrid)	SeqCOVID-SPAIN consortium/IBV(CSIC)	Fernando Lázaro-Perona et al
EPI_ISL_1323010	Hospital Universitario La Paz (Madrid)	SeqCOVID-SPAIN consortium/IBV(CSIC)	Fernando Lázaro-Perona et al
EPI_ISL_455566	Institute for Public Health	Laboratory for advanced genomics	Filip Rokić et al
EPI_ISL_454605	Institute for Public Health	Laboratory for advanced genomics	Filip Rokić et al
EPI_ISL_468656	Institute for Public Health	Laboratory for advanced genomics	Filip Rokić et al
EPI_ISL_468591	Institute for Public Health	Laboratory for advanced genomics	Filip Rokić et al

EPI_ISL_454574	nstitute for Public Health	Laboratory for advanced genomics	Filip Rokić et al
EPI_ISL_417949	Laboratório Hermes Pardini	Bioinformatics Laboratory - LNCC	Filipe Romero et al
EPI_ISL_1592227	Laboratório Hermes Pardini	Laboratório de Biologia Integrativa, Instituto de Ciências Biológicas, Universidade Federal de Minas Gerais	Filipe Romero Rebello Moreira et al
EPI_ISL_1592229	Laboratório Hermes Pardini	Laboratório de Biologia Integrativa, Instituto de Ciências Biológicas, Universidade Federal de Minas Gerais	Filipe Romero Rebello Moreira et al
EPI_ISL_1133267	Laboratório Hermes Pardini	Laboratório de Biologia Integrativa, Instituto de Ciências Biológicas, Universidade Federal de Minas Gerais	Filipe Romero Rebello Moreira et al
EPI_ISL_1494967	Laboratório de Biologia Integrativa	Laboratório de Biologia Integrativa	Filipe Romero Rebello Moreira et al
EPI_ISL_1494968	Laboratório de Biologia Integrativa	Laboratório de Biologia Integrativa	Filipe Romero Rebello Moreira et al
EPI_ISL_1494969	Laboratório de Biologia Integrativa	Laboratório de Biologia Integrativa	Filipe Romero Rebello Moreira et al
EPI_ISL_1494971	Laboratório de Biologia Integrativa	Laboratório de Biologia Integrativa	Filipe Romero Rebello Moreira et al
EPI_ISL_1494972	Laboratório de Biologia Integrativa	Laboratório de Biologia Integrativa	Filipe Romero Rebello Moreira et al
EPI_ISL_1494973	Laboratório de Biologia Integrativa	Laboratório de Biologia Integrativa	Filipe Romero Rebello Moreira et al
EPI_ISL_1494974	Laboratório de Biologia Integrativa	Laboratório de Biologia Integrativa	Filipe Romero Rebello Moreira et al
EPI_ISL_1494975	Laboratório de Biologia Integrativa	Laboratório de Biologia Integrativa	Filipe Romero Rebello Moreira et al
EPI_ISL_1494977	Laboratório de Biologia Integrativa	Laboratório de Biologia Integrativa	Filipe Romero Rebello Moreira et al
EPI_ISL_1494978	Laboratório de Biologia Integrativa	Laboratório de Biologia Integrativa	Filipe Romero Rebello Moreira et al
EPI_ISL_1494982	Laboratório de Biologia Integrativa	Laboratório de Biologia Integrativa	Filipe Romero Rebello Moreira et al
EPI_ISL_1494986	Laboratório de Biologia Integrativa	Laboratório de Biologia Integrativa	Filipe Romero Rebello Moreira et al
EPI_ISL_1494987	Laboratório de Biologia Integrativa	Laboratório de Biologia Integrativa	Filipe Romero Rebello Moreira et al
EPI_ISL_1494989	Laboratório de Biologia Integrativa	Laboratório de Biologia Integrativa	Filipe Romero Rebello Moreira et al
EPI_ISL_1494994	Laboratório de Biologia Integrativa	Laboratório de Biologia Integrativa	Filipe Romero Rebello Moreira et al
EPI_ISL_1494995	Laboratório de Biologia Integrativa	Laboratório de Biologia Integrativa	Filipe Romero Rebello Moreira et al
EPI_ISL_1494997	Laboratório de Biologia Integrativa	Laboratório de Biologia Integrativa	Filipe Romero Rebello Moreira et al
EPI_ISL_1494998	Laboratório de Biologia Integrativa	Laboratório de Biologia Integrativa	Filipe Romero Rebello Moreira et al
EPI_ISL_1494999	Laboratório de Biologia Integrativa	Laboratório de Biologia Integrativa	Filipe Romero Rebello Moreira et al
EPI_ISL_1495004	Laboratório de Biologia Integrativa	Laboratório de Biologia Integrativa	Filipe Romero Rebello Moreira et al
EPI_ISL_1495005	Laboratório de Biologia Integrativa	Laboratório de Biologia Integrativa	Filipe Romero Rebello Moreira et al
EPI_ISL_1495006	Laboratório de Biologia Integrativa	Laboratório de Biologia Integrativa	Filipe Romero Rebello Moreira et al
EPI_ISL_1495007	Laboratório de Biologia Integrativa	Laboratório de Biologia Integrativa	Filipe Romero Rebello Moreira et al
EPI_ISL_1495009	Laboratório de Biologia Integrativa	Laboratório de Biologia Integrativa	Filipe Romero Rebello Moreira et al

EPI_ISL_1495010	Laboratório de Biologia Integrativa	Laboratório de Biologia Integrativa	Filipe Romero Rebello Moreira et al
EPI_ISL_1495011	Laboratório de Biologia Integrativa	Laboratório de Biologia Integrativa	Filipe Romero Rebello Moreira et al
EPI_ISL_1495012	Laboratório de Biologia Integrativa	Laboratório de Biologia Integrativa	Filipe Romero Rebello Moreira et al
EPI_ISL_1495013	Laboratório de Biologia Integrativa	Laboratório de Biologia Integrativa	Filipe Romero Rebello Moreira et al
EPI_ISL_1495014	Laboratório de Biologia Integrativa	Laboratório de Biologia Integrativa	Filipe Romero Rebello Moreira et al
EPI_ISL_1495015	Laboratório de Biologia Integrativa	Laboratório de Biologia Integrativa	Filipe Romero Rebello Moreira et al
EPI_ISL_1495016	Laboratório de Biologia Integrativa	Laboratório de Biologia Integrativa	Filipe Romero Rebello Moreira et al
EPI_ISL_1495019	Laboratório de Biologia Integrativa	Laboratório de Biologia Integrativa	Filipe Romero Rebello Moreira et al
EPI_ISL_1495020	Laboratório de Biologia Integrativa	Laboratório de Biologia Integrativa	Filipe Romero Rebello Moreira et al
EPI_ISL_1495021	Laboratório de Biologia Integrativa	Laboratório de Biologia Integrativa	Filipe Romero Rebello Moreira et al
EPI_ISL_1495022	Laboratório de Biologia Integrativa	Laboratório de Biologia Integrativa	Filipe Romero Rebello Moreira et al
EPI_ISL_1495023	Laboratório de Biologia Integrativa	Laboratório de Biologia Integrativa	Filipe Romero Rebello Moreira et al
EPI_ISL_1495024	Laboratório de Biologia Integrativa	Laboratório de Biologia Integrativa	Filipe Romero Rebello Moreira et al
EPI_ISL_1495025	Laboratório de Biologia Integrativa	Laboratório de Biologia Integrativa	Filipe Romero Rebello Moreira et al
EPI_ISL_1495026	Laboratório de Biologia Integrativa	Laboratório de Biologia Integrativa	Filipe Romero Rebello Moreira et al
EPI_ISL_1495027	Laboratório de Biologia Integrativa	Laboratório de Biologia Integrativa	Filipe Romero Rebello Moreira et al
EPI_ISL_1495030	Laboratório de Biologia Integrativa	Laboratório de Biologia Integrativa	Filipe Romero Rebello Moreira et al
EPI_ISL_1495031	Laboratório de Biologia Integrativa	Laboratório de Biologia Integrativa	Filipe Romero Rebello Moreira et al
EPI_ISL_1495032	Laboratório de Biologia Integrativa	Laboratório de Biologia Integrativa	Filipe Romero Rebello Moreira et al
EPI_ISL_1495035	Laboratório de Biologia Integrativa	Laboratório de Biologia Integrativa	Filipe Romero Rebello Moreira et al
EPI_ISL_1495036	Laboratório de Biologia Integrativa	Laboratório de Biologia Integrativa	Filipe Romero Rebello Moreira et al
EPI_ISL_1495037	Laboratório de Biologia Integrativa	Laboratório de Biologia Integrativa	Filipe Romero Rebello Moreira et al
EPI_ISL_1495038	Laboratório de Biologia Integrativa	Laboratório de Biologia Integrativa	Filipe Romero Rebello Moreira et al
EPI_ISL_1495042	Laboratório de Biologia Integrativa	Laboratório de Biologia Integrativa	Filipe Romero Rebello Moreira et al
EPI_ISL_1494960	Laboratório de Biologia Integrativa	Laboratório de Biologia Integrativa	Filipe Romero Rebello Moreira et al
EPI_ISL_1494961	Laboratório de Biologia Integrativa	Laboratório de Biologia Integrativa	Filipe Romero Rebello Moreira et al
EPI_ISL_1494962	Laboratório de Biologia Integrativa	Laboratório de Biologia Integrativa	Filipe Romero Rebello Moreira et al
EPI_ISL_1494963	Laboratório de Biologia Integrativa	Laboratório de Biologia Integrativa	Filipe Romero Rebello Moreira et al
EPI_ISL_1494964	Laboratório de Biologia Integrativa	Laboratório de Biologia Integrativa	Filipe Romero Rebello Moreira et al
EPI_ISL_1494965	Laboratório de Biologia Integrativa	Laboratório de Biologia Integrativa	Filipe Romero Rebello Moreira et al

EPI_ISL_1494966	Laboratório de Biologia Integrativa	Laboratório de Biologia Integrativa	Filipe Romero Rebello Moreira et al
EPI_ISL_1133274	Laboratório Hermes Pardini	Laboratório de Biologia Integrativa, Instituto de Ciências Biológicas, Universidade Federal de Minas Gerais	Filipe Romero Rebello Moreira et al
EPI_ISL_1133256	Laboratório Hermes Pardini	Laboratório de Biologia Integrativa, Instituto de Ciências Biológicas, Universidade Federal de Minas Gerais	Filipe Romero Rebello Moreira et al
EPI_ISL_1592230	Laboratório Hermes Pardini	Laboratório de Biologia Integrativa, Instituto de Ciências Biológicas, Universidade Federal de Minas Gerais	Filipe Romero Rebello Moreira et al
EPI_ISL_1133261	Laboratório Hermes Pardini	Laboratório de Biologia Integrativa, Instituto de Ciências Biológicas, Universidade Federal de Minas Gerais	Filipe Romero Rebello Moreira et al
EPI_ISL_1357775	National Virus Reference Laboratory	National Virus Reference Laboratory	Fiona Crispie et al
EPI_ISL_1357776	National Virus Reference Laboratory	National Virus Reference Laboratory	Fiona Crispie et al
EPI_ISL_1010714	New South Wales Health Pathology Royal Prince Alfred Hospital	Microbiology RPAH	Foster et al
EPI_ISL_1061033	New South Wales Health Pathology Royal Prince Alfred Hospital	Microbiology RPAH	Foster et al
EPI_ISL_1061037	New South Wales Health Pathology Royal Prince Alfred Hospital	Microbiology RPAH	Foster et al
EPI_ISL_1121987	New South Wales Health Pathology Royal Prince Alfred Hospital	Microbiology RPAH	Foster et al
EPI_ISL_1315067	New South Wales Health Pathology Royal Prince Alfred Hospital	Microbiology RPAH	Foster et al
EPI_ISL_1660408	New South Wales Health Pathology Royal Prince Alfred Hospital	Microbiology RPAH	Foster et al
EPI_ISL_678320	Area of Virology, Serology and Virology Division (SAViD), New South Wales Health Pathology Randwick	Virology Research Laboratory; Area of Virology, Serology and Virology Division (SAViD), New South Wales Health Pathology Randwick	Foster et al
EPI_ISL_678344	Area of Virology, Serology and Virology Division (SAViD), New South Wales Health Pathology Randwick	Virology Research Laboratory; Area of Virology, Serology and Virology Division (SAViD), New South Wales Health Pathology Randwick	Foster et al
EPI_ISL_1121976	Area of Virology, Serology and Virology Division (SAViD), New South Wales Health Pathology Randwick	Virology Research Laboratory; Area of Virology, Serology and Virology Division (SAViD), New South Wales Health Pathology Randwick	Foster et al
EPI_ISL_1406435	Area of Virology, Serology and Virology Division (SAViD), New South Wales Health Pathology Randwick	Virology Research Laboratory; Area of Virology, Serology and Virology Division (SAViD), New South Wales Health Pathology Randwick	Foster et al
EPI_ISL_1516747	Area of Virology, Serology and Virology Division (SAViD), New South Wales Health Pathology Randwick	Virology Research Laboratory; Area of Virology, Serology and Virology Division (SAViD), New South Wales Health Pathology Randwick	Foster et al
EPI_ISL_1081793	Philippine Red Cross	Philippine Genome Center	Francis A. Tablizo et al
EPI_ISL_1081795	SOUTH SUPER HI-WAY	Philippine Genome Center	Francis A. Tablizo et al
EPI_ISL_1122458	The Lord's Grace Medical and Industrial Clinic	Philippine Genome Center	Francis A. Tablizo et al
EPI_ISL_1213568	Lung Center of the Philippines (LCP)	Philippine Genome Center	Francis A. Tablizo et al
EPI_ISL_1122457	Hi-Precision Diagnostics	Philippine Genome Center	Francis A. Tablizo et al
EPI_ISL_1122455	Southern Philippines Medical Center	Philippine Genome Center	Francis A. Tablizo et al
EPI_ISL_1122456	Manila Doctors Hospital	Philippine Genome Center	Francis A. Tablizo et al
EPI_ISL_1213571	Negros Oriental Provincial Hospital	Philippine Genome Center	Francis A. Tablizo et al
EPI_ISL_1213573	Negros Oriental Provincial Hospital	Philippine Genome Center	Francis A. Tablizo et al

EPI_ISL_1213554	Cebu TB Reference Laboratory	Philippine Genome Center	Francis A. Tablizo et al
EPI_ISL_1213518	Bohol Containerized PCR Laboratory	Philippine Genome Center	Francis A. Tablizo et al
EPI_ISL_1213509	Bohol Containerized PCR Laboratory	Philippine Genome Center	Francis A. Tablizo et al
EPI_ISL_1213530	Bohol Containerized PCR Laboratory	Philippine Genome Center	Francis A. Tablizo et al
EPI_ISL_434536	Hospital San Vicente de Paul	Inciensa, Instituto Costarricense de Investigación y Enseñanza en Nutrición y Salud	Francisco Duarte et al
EPI_ISL_491451	Hospital México	Inciensa, Instituto Costarricense de Investigación y Enseñanza en Nutrición y Salud	Francisco Duarte et al
EPI_ISL_512655	Hospital De Niños Dr. Carlos Saenz Herrera [San Jose/San Jose]	Inciensa, Instituto Costarricense de Investigación y Enseñanza en Nutrición y Salud	Francisco Duarte et al
EPI_ISL_512668	Area De Salud Corredores	Inciensa, Instituto Costarricense de Investigación y Enseñanza en Nutrición y Salud	Francisco Duarte et al
EPI_ISL_512669	Area De Salud Alajuela Norte - Clinica Dr. Marcial Rodriguez	Inciensa, Instituto Costarricense de Investigación y Enseñanza en Nutrición y Salud	Francisco Duarte et al
EPI_ISL_527740	Area De Salud Alajuela Norte - Clinica Dr. Marcial Rodriguez	Inciensa, Instituto Costarricense de Investigación y Enseñanza en Nutrición y Salud	Francisco Duarte et al
EPI_ISL_527756	Area De Salud Aserri	Inciensa, Instituto Costarricense de Investigación y Enseñanza en Nutrición y Salud	Francisco Duarte et al
EPI_ISL_682253	HOSPITAL SAN JUAN DE DIOS	Inciensa, Instituto Costarricense de Investigación y Enseñanza en Nutrición y Salud	Francisco Duarte et al
EPI_ISL_682271	HOSPITAL SAN JUAN DE DIOS	Inciensa, Instituto Costarricense de Investigación y Enseñanza en Nutrición y Salud	Francisco Duarte et al
EPI_ISL_682276	LABORATORIOS LABIN	Inciensa, Instituto Costarricense de Investigación y Enseñanza en Nutrición y Salud	Francisco Duarte et al
EPI_ISL_770006	Hle - Asociacion Hogar De Ancianos De Palmar Sur De Osa	Inciensa, Instituto Costarricense de Investigación y Enseñanza en Nutrición y Salud	Francisco Duarte et al
EPI_ISL_770028	Hospital Dr. Raul Blanco Cervantes	Inciensa, Instituto Costarricense de Investigación y Enseñanza en Nutrición y Salud	Francisco Duarte et al
EPI_ISL_770007	Area De Salud San Rafael	Inciensa, Instituto Costarricense de Investigación y Enseñanza en Nutrición y Salud	Francisco Duarte et al
EPI_ISL_770000	Area De Salud La Cruz	Inciensa, Instituto Costarricense de Investigación y Enseñanza en Nutrición y Salud	Francisco Duarte et al
EPI_ISL_770001	Area De Salud La Cruz	Inciensa, Instituto Costarricense de Investigación y Enseñanza en Nutrición y Salud	Francisco Duarte et al
EPI_ISL_770002	Area De Salud La Cruz	Inciensa, Instituto Costarricense de Investigación y Enseñanza en Nutrición y Salud	Francisco Duarte et al
EPI_ISL_770025	Area De Salud San Juan-San Diego-Concepcion 2	Inciensa, Instituto Costarricense de Investigación y Enseñanza en Nutrición y Salud	Francisco Duarte et al
EPI_ISL_770017	Area De Salud Perez Zeledon	Inciensa, Instituto Costarricense de Investigación y Enseñanza en Nutrición y Salud	Francisco Duarte et al
EPI_ISL_770029	Area De Salud Corredores	Inciensa, Instituto Costarricense de Investigación y Enseñanza en Nutrición y Salud	Francisco Duarte et al

EPI_ISL_770030	Area De Salud Buenos Aires	Inciensa, Instituto Costarricense de Investigación y Enseñanza en Nutrición y Salud	Francisco Duarte et al
EPI_ISL_769989	Area De Salud San Francisco-San Antonio (Coopesana)	Inciensa, Instituto Costarricense de Investigación y Enseñanza en Nutrición y Salud	Francisco Duarte et al
EPI_ISL_769986	Area De Salud Catedral Noreste	Inciensa, Instituto Costarricense de Investigación y Enseñanza en Nutrición y Salud	Francisco Duarte et al
EPI_ISL_770022	Area De Salud Perez Zeledon	Inciensa, Instituto Costarricense de Investigación y Enseñanza en Nutrición y Salud	Francisco Duarte et al
EPI_ISL_770023	Area De Salud San Francisco-San Antonio (Coopesana)	Inciensa, Instituto Costarricense de Investigación y Enseñanza en Nutrición y Salud	Francisco Duarte et al
EPI_ISL_914800	AREA DE SALUD SAN FRANCISCO-SAN ANTONIO (COOPESANA)	Inciensa, Instituto Costarricense de Investigación y Enseñanza en Nutrición y Salud	Francisco Duarte et al
EPI_ISL_914807	HLE - ASOCIACION HOGAR DE ANCIANOS SANTIAGO CRESPO CALVO	Inciensa, Instituto Costarricense de Investigación y Enseñanza en Nutrición y Salud	Francisco Duarte et al
EPI_ISL_914811	AREA DE SALUD GOICOECHEA 2 - CLINICA DR. JIMENEZ NUÑEZ	Inciensa, Instituto Costarricense de Investigación y Enseñanza en Nutrición y Salud	Francisco Duarte et al
EPI_ISL_914824	TAMIZAJE COMUNITARIO - PASO CANOAS	Inciensa, Instituto Costarricense de Investigación y Enseñanza en Nutrición y Salud	Francisco Duarte et al
EPI_ISL_914834	HOSPITAL METROPOLITANO	Inciensa, Instituto Costarricense de Investigación y Enseñanza en Nutrición y Salud	Francisco Duarte et al
EPI_ISL_1067600	TAMIZAJE COMUNITARIO - PASO CANOAS	Inciensa, Instituto Costarricense de Investigación y Enseñanza en Nutrición y Salud	Francisco Duarte et al
EPI_ISL_1067593	LABORATORIO CLINICO LABIN	Inciensa, Instituto Costarricense de Investigación y Enseñanza en Nutrición y Salud	Francisco Duarte et al
EPI_ISL_1196438	AREA DE SALUD CORRALILLO	Inciensa, Instituto Costarricense de Investigación y Enseñanza en Nutrición y Salud	Francisco Duarte et al
EPI_ISL_1379415	AREA DE SALUD PAVAS (COOPESALUD)	Inciensa, Instituto Costarricense de Investigación y Enseñanza en Nutrición y Salud	Francisco Duarte et al
EPI_ISL_1379422	HOSPITAL SAN JUAN DE DIOS	Inciensa, Instituto Costarricense de Investigación y Enseñanza en Nutrición y Salud	Francisco Duarte et al
EPI_ISL_1576835	Universidad Industrial de Santander (Laboratorio Central de Investigaciones - Clínica Chicamocha).	Universidad Industrial de Santander.	Francisco Martinez-Perez et al
EPI_ISL_1577026	Universidad Industrial de Santander (Laboratorio Central de Investigaciones - Clínica Chicamocha).	Universidad Industrial de Santander.	Francisco Martinez-Perez et al
EPI_ISL_1629710	Universidad Industrial de Santander (Laboratorio Central de Investigaciones - Clínica Chicamocha).	Universidad Industrial de Santander.	Francisco Martinez-Perez et al
EPI_ISL_1553838	CAP SANT JULIÀ DE VILATORTA	Banc de Sang i Teixits	Francisco Vidal et al
EPI_ISL_1665222	National Platform bis UMONS/Jolimont	National Platform bis UMONS/Jolimont	François Dufrasne et al
EPI_ISL_1623659	National Platform bis UMONS/Jolimont	National Platform bis UMONS/Jolimont	François Dufrasne et al
EPI_ISL_1665233	National Platform bis UMONS/Jolimont	National Platform bis UMONS/Jolimont	François Dufrasne et al
EPI_ISL_513312	Public Health, United States Air Force School of Aerospace Medicine	Public Health, United States Air Force School of Aerospace Medicine	Fries et al
EPI_ISL_513313	Public Health, United States Air Force School of Aerospace Medicine	Public Health, United States Air Force School of Aerospace Medicine	Fries et al

EPI_ISL_766034	RS Mitra Keluarga Bintaro	Eijkman Institute for Molecular Biology, Ministry of Research and Technology/National Agency for Research and Innovation	Frilasita A Yudhaputri et al
EPI_ISL_574611	RS Premier Bintaro	Eijkman Institute for Molecular Biology, Ministry of Research and Technology/National Agency for Research and Innovation	Frilasita A Yudhaputri et al
EPI_ISL_947270	RS Mitra Keluarga Gading Serpong	Eijkman Institute for Molecular Biology, Ministry of Research and Technology/National Agency for Research and Innovation	Frilasita A Yudhaputri et al
EPI_ISL_574606	RS Siloam Purwakarta	Eijkman Institute for Molecular Biology, Ministry of Research and Technology/National Agency for Research and Innovation	Frilasita A Yudhaputri et al
EPI_ISL_574612	RS Hermina Mekarsari	Eijkman Institute for Molecular Biology, Ministry of Research and Technology/National Agency for Research and Innovation	Frilasita A Yudhaputri et al
EPI_ISL_568690	RSUP Fatmawati	Eijkman Institute for Molecular Biology, Ministry of Research and Technology/National Agency for Research and Innovation	Frilasita A Yudhaputri et al
EPI_ISL_568691	RSUP Fatmawati	Eijkman Institute for Molecular Biology, Ministry of Research and Technology/National Agency for Research and Innovation	Frilasita A Yudhaputri et al
EPI_ISL_574608	RS Mitra Keluarga Kelapa Gading	Eijkman Institute for Molecular Biology, Ministry of Research and Technology/National Agency for Research and Innovation	Frilasita A Yudhaputri et al
EPI_ISL_766043	RS Pelni	Eijkman Institute for Molecular Biology, Ministry of Research and Technology/National Agency for Research and Innovation	Frilasita A Yudhaputri et al
EPI_ISL_766046	RSUP Fatmawati	Eijkman Institute for Molecular Biology, Ministry of Research and Technology/National Agency for Research and Innovation	Frilasita A Yudhaputri et al
EPI_ISL_574614	RS Kramat 128	Eijkman Institute for Molecular Biology, Ministry of Research and Technology/National Agency for Research and Innovation	Frilasita A Yudhaputri et al
EPI_ISL_766033	RSUD Prof. DR. W. Z. Johannes Kupang	Eijkman Institute for Molecular Biology, Ministry of Research and Technology/National Agency for Research and Innovation	Frilasita A Yudhaputri et al
EPI_ISL_766039	RS Bhayangkara Tk. III	Eijkman Institute for Molecular Biology, Ministry of Research and Technology/National Agency for Research and Innovation	Frilasita A Yudhaputri et al
EPI_ISL_574604	RSUP Prof. Dr. R. Kandou Manado	Eijkman Institute for Molecular Biology, Ministry of Research and Technology/National Agency for Research and Innovation	Frilasita A Yudhaputri et al
EPI_ISL_574605	RSUP dr. Sardjito	Eijkman Institute for Molecular Biology, Ministry of Research and Technology/National Agency for Research and Innovation	Frilasita A Yudhaputri et al
EPI_ISL_902739	Hospital Universitari Germans Trias i Pujol (HUGTiP) / Fundació Lluita contra la SIDA (FLSida)	IrsiCaixa - Can Ruti CovidSeq	Fundació irsiCaixa. Hospital Universitari Ge
EPI_ISL_902747	Hospital Universitari Germans Trias i Pujol (HUGTiP) / Fundació Lluita contra la SIDA (FLSida)	IrsiCaixa - Can Ruti CovidSeq	Fundació irsiCaixa. Hospital Universitari Ge
EPI_ISL_660069	Zurita & Zurita Laboratorios	Zurita & Zurita Laboratorios	Gabriela Sevillano Camilo Zurita-Salinas Ka
EPI_ISL_660070	Zurita & Zurita Laboratorios	Zurita & Zurita Laboratorios	Gabriela Sevillano Camilo Zurita-Salinas Ka
EPI_ISL_681703	Zurita & Zurita Laboratorios	Zurita & Zurita Laboratorios	Gabriela Sevillano et al
EPI_ISL_468752	Center for Genome Regulation (CRG)	Center for Mathematical Modeling and Center for Genome Regulation. Santiago, Chile	Gaete A et al
EPI_ISL_459858	Center for Genome Regulation (CRG)	Center for Mathematical Modeling and Center for Genome Regulation. Santiago, Chile	Gaete A et al
EPI_ISL_753417	Clinical virology Laboratory, Children's Hospital Los Angeles	Center for Personalized Medicine, Children's Hospital Los Angeles	Gai et al

rmans Trias i Pujol(HUGTiP) et al

rmans Trias i Pujol(HUGTiP) et al

aren Loaiza David Ortega-Paredes Jeannete

aren Loaiza David Ortega-Paredes Jeannete

EPI_ISL_602623	AHRI-Sigal	KRISP, KZN Research Innovation and Sequencing Platform	Gazy I et al
EPI_ISL_1615655	Centre de Recherches Médicales de Lambaréné (CERMEL)	Centre de Recherches Médicales de Lambaréné (CERMEL)	Gédéon Prince Manouana et al
EPI_ISL_637110	Rafik Hariri University Hospital	Microbial Pathogenomics Lab	Georgi Merhi et al
EPI_ISL_637112	Rafik Hariri University Hospital	Microbial Pathogenomics Lab	Georgi Merhi et al
EPI_ISL_637113	Rafik Hariri University Hospital	Microbial Pathogenomics Lab	Georgi Merhi et al
EPI_ISL_1116466	Laboratoire central de Virologie	Laboratoire de Biotechnologie	Ghizlane EL Amin et al
EPI_ISL_1116555	Laboratoire central de Virologie	Laboratoire de Biotechnologie	Ghizlane EL Amin et al
EPI_ISL_467431	Molecular Diagnostics Services (MDS)	KRISP, KZN Research Innovation and Sequencing Platform	Giandhari J et al
EPI_ISL_482706	Molecular Diagnostics Services (MDS)	KRISP, KZN Research Innovation and Sequencing Platform	Giandhari J et al
EPI_ISL_482854	Molecular Diagnostics Services (MDS)	KRISP, KZN Research Innovation and Sequencing Platform	Giandhari J et al
EPI_ISL_482871	Molecular Diagnostics Services (MDS)	KRISP, KZN Research Innovation and Sequencing Platform	Giandhari J et al
EPI_ISL_482872	Molecular Diagnostics Services (MDS)	KRISP, KZN Research Innovation and Sequencing Platform	Giandhari J et al
EPI_ISL_495526	NHLS-IALCH	KRISP, KZN Research Innovation and Sequencing Platform	Giandhari J et al
EPI_ISL_495554	NHLS-IALCH	KRISP, KZN Research Innovation and Sequencing Platform	Giandhari J et al
EPI_ISL_498076	NHLS-IALCH	KRISP, KZN Research Innovation and Sequencing Platform	Giandhari J et al
EPI_ISL_455634	KRISP, KZN Research Innovation and Sequencing Platform	KRISP, KZN Research Innovation and Sequencing Platform	Giandhari J et al
EPI_ISL_455638	KRISP, KZN Research Innovation and Sequencing Platform	KRISP, KZN Research Innovation and Sequencing Platform	Giandhari J et al
EPI_ISL_535402	NHLS-IALCH	KRISP, KZN Research Innovation and Sequencing Platform	Giandhari J et al
EPI_ISL_535474	NHLS-IALCH	KRISP, KZN Research Innovation and Sequencing Platform	Giandhari J et al
EPI_ISL_535521	NHLS-IALCH	KRISP, KZN Research Innovation and Sequencing Platform	Giandhari J et al
EPI_ISL_529792	NHLS-IALCH	KRISP, KZN Research Innovation and Sequencing Platform	Giandhari J et al
EPI_ISL_529793	NHLS-IALCH	KRISP, KZN Research Innovation and Sequencing Platform	Giandhari J et al
EPI_ISL_602640	NHLS-IALCH	KRISP, KZN Research Innovation and Sequencing Platform	Giandhari J et al
EPI_ISL_602751	NHLS-IALCH	KRISP, KZN Research Innovation and Sequencing Platform	Giandhari J et al
EPI_ISL_602755	NHLS-IALCH	KRISP, KZN Research Innovation and Sequencing Platform	Giandhari J et al
EPI_ISL_1085997	Netcare/AMPATH	KRISP, KZN Research Innovation and Sequencing Platform	Giandhari J et al
EPI_ISL_1085999	Netcare/AMPATH	KRISP, KZN Research Innovation and Sequencing Platform	Giandhari J et al
EPI_ISL_1371935	National Health Laboratory Service, South Africa	KRISP, KZn Research Innovation and Sequencing Platform	Giandhari J et al
EPI_ISL_1550934	National Health Laboratory Service, South Africa	KRISP, KZN Research Innovation and Sequencing Platform	Giandhari J et al
EPI_ISL_1550952	National Health Laboratory Service, South Africa	KRISP, KZN Research Innovation and Sequencing Platform	Giandhari J et al

EPI_ISL_1289446	Department of Health Technology and Informatics, The Hong Kong Polytechnic University	Department of Health Technology and Informatics, The Hong Kong Polytechnic University	Gilman Kit-Hang Siu et al
EPI_ISL_1018118	Department of Health Technology and Informatics, The Hong Kong Polytechnic University	Department of Health Technology and Informatics, The Hong Kong Polytechnic University	Gilman Kit-Hang Siu et al
EPI_ISL_1018119	Department of Health Technology and Informatics, The Hong Kong Polytechnic University	Department of Health Technology and Informatics, The Hong Kong Polytechnic University	Gilman Kit-Hang Siu et al
EPI_ISL_1018123	Department of Health Technology and Informatics, The Hong Kong Polytechnic University	Department of Health Technology and Informatics, The Hong Kong Polytechnic University	Gilman Kit-Hang Siu et al
EPI_ISL_1018312	Department of Health Technology and Informatics, The Hong Kong Polytechnic University	Department of Health Technology and Informatics, The Hong Kong Polytechnic University	Gilman Kit-Hang Siu et al
EPI_ISL_1019642	Department of Health Technology and Informatics, The Hong Kong Polytechnic University	Department of Health Technology and Informatics, The Hong Kong Polytechnic University	Gilman Kit-Hang Siu et al
EPI_ISL_1019674	Department of Health Technology and Informatics, The Hong Kong Polytechnic University	Department of Health Technology and Informatics, The Hong Kong Polytechnic University	Gilman Kit-Hang Siu et al
EPI_ISL_1019678	Department of Health Technology and Informatics, The Hong Kong Polytechnic University	Department of Health Technology and Informatics, The Hong Kong Polytechnic University	Gilman Kit-Hang Siu et al
EPI_ISL_1019734	Department of Health Technology and Informatics, The Hong Kong Polytechnic University	Department of Health Technology and Informatics, The Hong Kong Polytechnic University	Gilman Kit-Hang Siu et al
EPI_ISL_1019764	Department of Health Technology and Informatics, The Hong Kong Polytechnic University	Department of Health Technology and Informatics, The Hong Kong Polytechnic University	Gilman Kit-Hang Siu et al
EPI_ISL_1020215	Department of Health Technology and Informatics, The Hong Kong Polytechnic University	Department of Health Technology and Informatics, The Hong Kong Polytechnic University	Gilman Kit-Hang Siu et al
EPI_ISL_763062	Department for Virology, Molecular Biology and Genome Research, R. G. Lugar Center for Public Health Research, National Center for Disease Control and Public Health (NCDC) of Georgia.	Department for Virology, Molecular Biology and Genome Research, R. G. Lugar Center for Public Health Research, National Center for Disease Control and Public Health (NCDC) of Georgia.	Giorgi Tomashvili et al
EPI_ISL_1048371	Megalab, Molecular and Cytogenetics Diagnostics	Department for Virology, Molecular Biology and Genome Research, R. G. Lugar Center for Public Health Research, National Center for Disease Control and Public Health (NCDC) of Georgia.	Giorgi Tomashvili et al
EPI_ISL_876976	1.AO Universitaria 'S. Giovanni di Dio e Ruggi D'Aragona, Scuola Medica Salernitana' Hospital / 2.UOC di Virologia e Microbiologia, Università della Campania 'L. Vanvitelli' / 3.AO Universitaria 'Federico II' Napoli Hospital / 4. AORN 'San Giuseppe Moscati' Avellino Hospital / 5.AO 'San Pio - presidio G. Rummo' Benevento Hospital / 6.AO 'Sant'Anna e San Sebastiano' Caserta Hospital / 7.PO 'Maria Santissima Addolorata' Eboli Hospital / 8.Biogem Istituto di Ricerche Genetiche	1. Genome Research Center for Health (CRGS) / 2. Laboratory of Molecular Medicine and Genomics(LMMGe) / 3. Center for Research in Pure and Applied Mathematics (CRMPA)	Giorgio Giurato (Corresponding Author) et a
EPI_ISL_1366893	1.AO Universitaria 'S. Giovanni di Dio e Ruggi D'Aragona, Scuola Medica Salernitana' Hospital / 2.UOC di Virologia e Microbiologia, Università della Campania 'L. Vanvitelli' / 3.AO Universitaria 'Federico II' Napoli Hospital / 4. AORN 'San Giuseppe Moscati' Avellino Hospital / 5.AO 'San Pio - presidio G. Rummo' Benevento Hospital / 6.AO 'Sant'Anna e San Sebastiano' Caserta Hospital / 7.PO 'Maria Santissima Addolorata' Eboli Hospital / 8.Biogem Istituto di Ricerche Genetiche	1. Genome Research Center for Health (CRGS) / 2. Laboratory of Molecular Medicine and Genomics(LMMGe) / 3. Center for Research in Pure and Applied Mathematics (CRMPA)	Giorgio Giurato (Corresponding Author) et a

EPI_ISL_1361596	1.AO Universitaria 'S. Giovanni di Dio e Ruggi D'Aragona, Scuola Medica Salernitana' Hospital / 2.UOC di Virologia e Microbiologia, Università della Campania 'L. Vanvitelli' / 3.AO Universitaria 'Federico II' Napoli Hospital / 4. AORN 'San Giuseppe Moscati' Avellino Hospital / 5.AO 'San Pio - presidio G. Rummo' Benevento Hospital / 6.AO 'Sant'Anna e San Sebastiano' Caserta Hospital / 7.PO 'Maria Santissima Addolorata' Eboli Hospital / 8.Biogem Istituto di Ricerche Genetiche / 9. U.O.C. di Genetica Medica e di Laboratorio A.O.R. N., Azienda Ospedaliera di Rilievo Nazionale Antonio Cardarelli, Napoli / 10. Centro di riferimento Oncologico della Basilicata (IRCCS-CROB), Rionero in Vulture (PZ) / 11. Presidio Ospedaliero di Agropoli, Agropoli (SA).	1. Genome Research Center for Health (CRGS) / 2. Laboratory of Molecular Medicine and Genomics(LMMGe) / 3. Center for Research in Pure and Applied Mathematics (CRMPA)	Giorgio Giurato (Corresponding Author) et a
EPI_ISL_1298510	ASL Napoli 1 Centro	AMES Centro Polidiagnostico Strumentale S.r.l.	Giovanni Savarese et al
EPI_ISL_1298614	ASL Napoli 1 Centro	AMES Centro Polidiagnostico Strumentale S.r.l.	Giovanni Savarese et al
EPI_ISL_516620	Instituto de Diagnostico y Referencia Epidemiologicos (INDRE)	Instituto de Diagnostico y Referencia Epidemiologicos (INDRE)	Gisela Barrera-Badillo et al
EPI_ISL_806623	KEMRI-Wellcome Trust Research Programme/KEMRI-CGMR-C Kilifi	KEMRI-Wellcome Trust Research Programme/KEMRI-CGMR-C Kilifi	Githinji et al
EPI_ISL_806624	KEMRI-Wellcome Trust Research Programme/KEMRI-CGMR-C Kilifi	KEMRI-Wellcome Trust Research Programme/KEMRI-CGMR-C Kilifi	Githinji et al
EPI_ISL_806681	KEMRI-Wellcome Trust Research Programme/KEMRI-CGMR-C Kilifi	KEMRI-Wellcome Trust Research Programme/KEMRI-CGMR-C Kilifi	Githinji et al
EPI_ISL_806630	KEMRI-Wellcome Trust Research Programme/KEMRI-CGMR-C Kilifi	KEMRI-Wellcome Trust Research Programme/KEMRI-CGMR-C Kilifi	Githinji et al
EPI_ISL_806632	KEMRI-Wellcome Trust Research Programme/KEMRI-CGMR-C Kilifi	KEMRI-Wellcome Trust Research Programme/KEMRI-CGMR-C Kilifi	Githinji et al
EPI_ISL_806554	KEMRI-Wellcome Trust Research Programme/KEMRI-CGMR-C Kilifi	KEMRI-Wellcome Trust Research Programme/KEMRI-CGMR-C Kilifi	Githinji et al
EPI_ISL_806558	KEMRI-Wellcome Trust Research Programme/KEMRI-CGMR-C Kilifi	KEMRI-Wellcome Trust Research Programme/KEMRI-CGMR-C Kilifi	Githinji et al
EPI_ISL_806566	KEMRI-Wellcome Trust Research Programme/KEMRI-CGMR-C Kilifi	KEMRI-Wellcome Trust Research Programme/KEMRI-CGMR-C Kilifi	Githinji et al
EPI_ISL_806570	KEMRI-Wellcome Trust Research Programme/KEMRI-CGMR-C Kilifi	KEMRI-Wellcome Trust Research Programme/KEMRI-CGMR-C Kilifi	Githinji et al
EPI_ISL_806709	KEMRI-Wellcome Trust Research Programme/KEMRI-CGMR-C Kilifi	KEMRI-Wellcome Trust Research Programme/KEMRI-CGMR-C Kilifi	Githinji et al
EPI_ISL_806710	KEMRI-Wellcome Trust Research Programme/KEMRI-CGMR-C Kilifi	KEMRI-Wellcome Trust Research Programme/KEMRI-CGMR-C Kilifi	Githinji et al
EPI_ISL_806712	KEMRI-Wellcome Trust Research Programme/KEMRI-CGMR-C Kilifi	KEMRI-Wellcome Trust Research Programme/KEMRI-CGMR-C Kilifi	Githinji et al
EPI_ISL_806715	KEMRI-Wellcome Trust Research Programme/KEMRI-CGMR-C Kilifi	KEMRI-Wellcome Trust Research Programme/KEMRI-CGMR-C Kilifi	Githinji et al
EPI_ISL_806716	KEMRI-Wellcome Trust Research Programme/KEMRI-CGMR-C Kilifi	KEMRI-Wellcome Trust Research Programme/KEMRI-CGMR-C Kilifi	Githinji et al
EPI_ISL_806611	KEMRI-Wellcome Trust Research Programme/KEMRI-CGMR-C Kilifi	KEMRI-Wellcome Trust Research Programme/KEMRI-CGMR-C Kilifi	Githinji et al
EPI_ISL_806617	KEMRI-Wellcome Trust Research Programme/KEMRI-CGMR-C Kilifi	KEMRI-Wellcome Trust Research Programme/KEMRI-CGMR-C Kilifi	Githinji et al
EPI_ISL_968881	KEMRI-Wellcome Trust Research Programme/KEMRI-CGMR-C Kilifi	KEMRI-Wellcome Trust Research Programme/KEMRI-CGMR-C Kilifi	Githinji et al
EPI_ISL_968892	KEMRI-Wellcome Trust Research Programme/KEMRI-CGMR-C Kilifi	KEMRI-Wellcome Trust Research Programme/KEMRI-CGMR-C Kilifi	Githinji et al
EPI_ISL_855536	KEMRI-Wellcome Trust Research Programme/KEMRI-CGMR-C Kilifi	KEMRI-Wellcome Trust Research Programme/KEMRI-CGMR-C Kilifi	Githinji et al
EPI_ISL_968901	KEMRI-Wellcome Trust Research Programme/KEMRI-CGMR-C Kilifi	KEMRI-Wellcome Trust Research Programme/KEMRI-CGMR-C Kilifi	Githinji et al
EPI_ISL_855533	KEMRI-Wellcome Trust Research Programme/KEMRI-CGMR-C Kilifi	KEMRI-Wellcome Trust Research Programme/KEMRI-CGMR-C Kilifi	Githinji et al

EPI_ISL_968902	KEMRI-Wellcome Trust Research Programme/KEMRI-CGMR-C Kilifi	KEMRI-Wellcome Trust Research Programme/KEMRI-CGMR-C Kilifi	Githinji et al
EPI_ISL_968903	KEMRI-Wellcome Trust Research Programme/KEMRI-CGMR-C Kilifi	KEMRI-Wellcome Trust Research Programme/KEMRI-CGMR-C Kilifi	Githinji et al
EPI_ISL_855538	KEMRI-Wellcome Trust Research Programme/KEMRI-CGMR-C Kilifi	KEMRI-Wellcome Trust Research Programme/KEMRI-CGMR-C Kilifi	Githinji et al
EPI_ISL_968911	KEMRI-Wellcome Trust Research Programme/KEMRI-CGMR-C Kilifi	KEMRI-Wellcome Trust Research Programme/KEMRI-CGMR-C Kilifi	Githinji et al
EPI_ISL_968930	KEMRI-Wellcome Trust Research Programme/KEMRI-CGMR-C Kilifi	KEMRI-Wellcome Trust Research Programme/KEMRI-CGMR-C Kilifi	Githinji et al
EPI_ISL_1039225	KEMRI-Wellcome Trust Research Programme/KEMRI-CGMR-C Kilifi	KEMRI-Wellcome Trust Research Programme/KEMRI-CGMR-C Kilifi	Githinji et al
EPI_ISL_968997	KEMRI-Wellcome Trust Research Programme/KEMRI-CGMR-C Kilifi	KEMRI-Wellcome Trust Research Programme/KEMRI-CGMR-C Kilifi	Githinji et al
EPI_ISL_855535	KEMRI-Wellcome Trust Research Programme/KEMRI-CGMR-C Kilifi	KEMRI-Wellcome Trust Research Programme/KEMRI-CGMR-C Kilifi	Githinji et al
EPI_ISL_969003	KEMRI-Wellcome Trust Research Programme/KEMRI-CGMR-C Kilifi	KEMRI-Wellcome Trust Research Programme/KEMRI-CGMR-C Kilifi	Githinji et al
EPI_ISL_969017	KEMRI-Wellcome Trust Research Programme/KEMRI-CGMR-C Kilifi	KEMRI-Wellcome Trust Research Programme/KEMRI-CGMR-C Kilifi	Githinji et al
EPI_ISL_568708	KEMRI-Wellcome Trust Research Programme/KEMRI-CGMR-C Kilifi	KEMRI-Wellcome Trust Research Programme/KEMRI-CGMR-C Kilifi	Githinji et al 2020 et al
EPI_ISL_568790	KEMRI-Wellcome Trust Research Programme/KEMRI-CGMR-C Kilifi	KEMRI-Wellcome Trust Research Programme/KEMRI-CGMR-C Kilifi	Githinji et al 2020 et al
EPI_ISL_568804	KEMRI-Wellcome Trust Research Programme/KEMRI-CGMR-C Kilifi	KEMRI-Wellcome Trust Research Programme/KEMRI-CGMR-C Kilifi	Githinji et al 2020 et al
EPI_ISL_568815	KEMRI-Wellcome Trust Research Programme/KEMRI-CGMR-C Kilifi	KEMRI-Wellcome Trust Research Programme/KEMRI-CGMR-C Kilifi	Githinji et al 2020 et al
EPI_ISL_568863	KEMRI-Wellcome Trust Research Programme/KEMRI-CGMR-C Kilifi	KEMRI-Wellcome Trust Research Programme/KEMRI-CGMR-C Kilifi	Githinji et al 2020 et al
EPI_ISL_1440078	KEMRI-Wellcome Trust Research Programme,Kilifi	KEMRI-Wellcome Trust Research Programme,Kilifi	Githinji G. et al
EPI_ISL_1440084	KEMRI-Wellcome Trust Research Programme,Kilifi	KEMRI-Wellcome Trust Research Programme,Kilifi	Githinji G. et al
EPI_ISL_1440102	KEMRI-Wellcome Trust Research Programme,Kilifi	KEMRI-Wellcome Trust Research Programme,Kilifi	Githinji G. et al
EPI_ISL_1440106	KEMRI-Wellcome Trust Research Programme,Kilifi	KEMRI-Wellcome Trust Research Programme,Kilifi	Githinji G. et al
EPI_ISL_1440096	KEMRI-Wellcome Trust Research Programme,Kilifi	KEMRI-Wellcome Trust Research Programme,Kilifi	Githinji G. et al
EPI_ISL_1440115	KEMRI-Wellcome Trust Research Programme,Kilifi	KEMRI-Wellcome Trust Research Programme,Kilifi	Githinji G. et al
EPI_ISL_1440118	KEMRI-Wellcome Trust Research Programme,Kilifi	KEMRI-Wellcome Trust Research Programme,Kilifi	Githinji G. et al
EPI_ISL_1440119	KEMRI-Wellcome Trust Research Programme,Kilifi	KEMRI-Wellcome Trust Research Programme,Kilifi	Githinji G. et al
EPI_ISL_1440136	KEMRI-Wellcome Trust Research Programme,Kilifi	KEMRI-Wellcome Trust Research Programme,Kilifi	Githinji G. et al
EPI_ISL_1440181	KEMRI-Wellcome Trust Research Programme,Kilifi	KEMRI-Wellcome Trust Research Programme,Kilifi	Githinji G. et al
EPI_ISL_1440292	KEMRI-Wellcome Trust Research Programme,Kilifi	KEMRI-Wellcome Trust Research Programme,Kilifi	Githinji G. et al
EPI_ISL_1610624	KEMRI Center for Biotechnology Research and Development	KEMRI-Wellcome Trust Research Programme,Kilifi	Githinji G. et al
EPI_ISL_1610629	KEMRI Center for Biotechnology Research and Development	KEMRI-Wellcome Trust Research Programme,Kilifi	Githinji G. et al
EPI_ISL_457874	KEMRI-CGMR-C	KEMRI-Wellcome Trust Research Programme/KEMRI-CGMR-C Kilifi	Githinji G. et al 2020 et al
EPI_ISL_457876	KEMRI-CGMR-C	KEMRI-Wellcome Trust Research Programme/KEMRI-CGMR-C Kilifi	Githinji G. et al 2020 et al

EPI_ISL_457890	KEMRI-CGMR-C	KEMRI-Wellcome Trust Research Programme/KEMRI-CGMR-C Kilifi	Githinji G. et al 2020 et al
EPI_ISL_457916	KEMRI-CGMR-C	KEMRI-Wellcome Trust Research Programme/KEMRI-CGMR-C Kilifi	Githinji G. et al 2020 et al
EPI_ISL_457836	National Public Health Laboratory	KEMRI-Wellcome Trust Research Programme/KEMRI-CGMR-C Kilifi	Githinji G. et al 2020 et al
EPI_ISL_457932	KEMRI-Centre for Virus Research	KEMRI-Wellcome Trust Research Programme/KEMRI-CGMR-C Kilifi	Githinji G. et al 2020 et al
EPI_ISL_457933	KEMRI-Centre for Virus Research	KEMRI-Wellcome Trust Research Programme/KEMRI-CGMR-C Kilifi	Githinji G. et al 2020 et al
EPI_ISL_419687	Servicio de Microbiología. Consorcio Hospital General Universitario de Valencia	Sequencing and Bioinformatics Service and Molecular Epidemiology Research Group. FISABIO-Public Health	Giuseppe D'Auria et al
EPI_ISL_512811	Kenema Government Hospital, Ministry of Health and Sanitation	Kenema Government Hospital, Ministry of Health and Sanitation	Goba et al
EPI_ISL_512812	Kenema Government Hospital, Ministry of Health and Sanitation	Kenema Government Hospital, Ministry of Health and Sanitation	Goba et al
EPI_ISL_512821	Kenema Government Hospital, Ministry of Health and Sanitation	Kenema Government Hospital, Ministry of Health and Sanitation	Goba et al
EPI_ISL_462753	University Clinical Hospital of Mostar	University of Sarajevo Veterinary Faculty	Goletic et al
EPI_ISL_1016885	University of Sarajevo, Veterinary Faculty, Laboratory for Molecular Diagnostic and Research Laboratory	University of Sarajevo, Veterinary Faculty, Laboratory for Molecular Diagnostic and Research Laboratory	Goletic S. et al
EPI_ISL_1016970	University of Sarajevo, Veterinary Faculty, Laboratory for Molecular Diagnostic and Research Laboratory	University of Sarajevo, Veterinary Faculty, Laboratory for Molecular Diagnostic and Research Laboratory	Goletic S. et al
EPI_ISL_955169	University of Sarajevo, Veterinary Faculty, Laboratory for Molecular Diagnostic and Research Laboratory	University of Sarajevo, Veterinary Faculty, Laboratory for Molecular Diagnostic and Research Laboratory	Goletić Š. et al
EPI_ISL_955171	University of Sarajevo, Veterinary Faculty, Laboratory for Molecular Diagnostic and Research Laboratory	University of Sarajevo, Veterinary Faculty, Laboratory for Molecular Diagnostic and Research Laboratory	Goletić Š. et al
EPI_ISL_955173	University of Sarajevo, Veterinary Faculty, Laboratory for Molecular Diagnostic and Research Laboratory	University of Sarajevo, Veterinary Faculty, Laboratory for Molecular Diagnostic and Research Laboratory	Goletić Š. et al
EPI_ISL_955211	University of Sarajevo, Veterinary Faculty, Laboratory for Molecular Diagnostic and Research Laboratory	University of Sarajevo, Veterinary Faculty, Laboratory for Molecular Diagnostic and Research Laboratory	Goletić Š. et al
EPI_ISL_1016690	University of Sarajevo, Veterinary Faculty, Laboratory for Molecular Diagnostic and Research Laboratory	University of Sarajevo, Veterinary Faculty, Laboratory for Molecular Diagnostic and Research Laboratory	Goletic T. et al
EPI_ISL_1016884	University of Sarajevo, Veterinary Faculty, Laboratory for Molecular Diagnostic and Research Laboratory	University of Sarajevo, Veterinary Faculty, Laboratory for Molecular Diagnostic and Research Laboratory	Goletic T. et al
EPI_ISL_955142	University of Sarajevo, Veterinary Faculty, Laboratory for Molecular Diagnostic and Research Laboratory	University of Sarajevo, Veterinary Faculty, Laboratory for Molecular Diagnostic and Research Laboratory	Goletić T. et al
EPI_ISL_955144	University of Sarajevo, Veterinary Faculty, Laboratory for Molecular Diagnostic and Research Laboratory	University of Sarajevo, Veterinary Faculty, Laboratory for Molecular Diagnostic and Research Laboratory	Goletić T. et al
EPI_ISL_955146	University of Sarajevo, Veterinary Faculty, Laboratory for Molecular Diagnostic and Research Laboratory	University of Sarajevo, Veterinary Faculty, Laboratory for Molecular Diagnostic and Research Laboratory	Goletić T. et al
EPI_ISL_955148	University of Sarajevo, Veterinary Faculty, Laboratory for Molecular Diagnostic and Research Laboratory	University of Sarajevo, Veterinary Faculty, Laboratory for Molecular Diagnostic and Research Laboratory	Goletić T. et al
EPI_ISL_955150	University of Sarajevo, Veterinary Faculty, Laboratory for Molecular Diagnostic and Research Laboratory	University of Sarajevo, Veterinary Faculty, Laboratory for Molecular Diagnostic and Research Laboratory	Goletić T. et al
EPI_ISL_955151	University of Sarajevo, Veterinary Faculty, Laboratory for Molecular Diagnostic and Research Laboratory	University of Sarajevo, Veterinary Faculty, Laboratory for Molecular Diagnostic and Research Laboratory	Goletić T. et al

EPI_ISL_955155	University of Sarajevo, Veterinary Faculty, Laboratory for Molecular Diagnostic and Research Laboratory	University of Sarajevo, Veterinary Faculty, Laboratory for Molecular Diagnostic and Research Laboratory	Goletić T. et al
EPI_ISL_955156	University of Sarajevo, Veterinary Faculty, Laboratory for Molecular Diagnostic and Research Laboratory	University of Sarajevo, Veterinary Faculty, Laboratory for Molecular Diagnostic and Research Laboratory	Goletić T. et al
EPI_ISL_955170	University of Sarajevo, Veterinary Faculty, Laboratory for Molecular Diagnostic and Research Laboratory	University of Sarajevo, Veterinary Faculty, Laboratory for Molecular Diagnostic and Research Laboratory	Goletić T. et al
EPI_ISL_1502889	Gorgas Memorial Laboratory of Health Studies	Gorgas Memorial Laboratory of Health Studies	Gonzalez Claudia et al
EPI_ISL_1502895	Gorgas Memorial Laboratory of Health Studies	Gorgas Memorial Laboratory of Health Studies	Gonzalez Claudia et al
EPI_ISL_1502903	Gorgas Memorial Laboratory of Health Studies	Gorgas Memorial Laboratory of Health Studies	Gonzalez Claudia et al
EPI_ISL_1502908	Gorgas Memorial Laboratory of Health Studies	Gorgas Memorial Laboratory of Health Studies	Gonzalez Claudia et al
EPI_ISL_1502945	Gorgas Memorial Laboratory of Health Studies	Gorgas Memorial Laboratory of Health Studies	Gonzalez Claudia et al
EPI_ISL_1502980	Gorgas Memorial Laboratory of Health Studies	Gorgas Memorial Laboratory of Health Studies	Gonzalez Claudia et al
EPI_ISL_1502982	Gorgas Memorial Laboratory of Health Studies	Gorgas Memorial Laboratory of Health Studies	Gonzalez Claudia et al
EPI_ISL_1503122	Gorgas Memorial Laboratory of Health Studies	Gorgas Memorial Laboratory of Health Studies	Gonzalez Claudia et al
EPI_ISL_1503124	Hospital Regional Anita Moreno Los Santos	Gorgas Memorial Laboratory of Health Studies	Gonzalez Claudia et al
EPI_ISL_1503134	Gorgas Memorial Laboratory of Health Studies	Gorgas Memorial Laboratory of Health Studies	Gonzalez Claudia et al
EPI_ISL_1503139	Gorgas Memorial Laboratory of Health Studies	Gorgas Memorial Laboratory of Health Studies	Gonzalez Claudia et al
EPI_ISL_1503017	Gorgas Memorial Laboratory of Health Studies	Gorgas Memorial Laboratory of Health Studies	Gonzalez Claudia et al
EPI_ISL_1503021	Gorgas Memorial Laboratory of Health Studies	Gorgas Memorial Laboratory of Health Studies	Gonzalez Claudia et al
EPI_ISL_1503024	Gorgas Memorial Laboratory of Health Studies	Gorgas Memorial Laboratory of Health Studies	Gonzalez Claudia et al
EPI_ISL_1503051	Gorgas Memorial Laboratory of Health Studies	Gorgas Memorial Laboratory of Health Studies	Gonzalez Claudia et al
EPI_ISL_1503052	Gorgas Memorial Laboratory of Health Studies	Gorgas Memorial Laboratory of Health Studies	Gonzalez Claudia et al
EPI_ISL_1503054	Gorgas Memorial Laboratory of Health Studies	Gorgas Memorial Laboratory of Health Studies	Gonzalez Claudia et al
EPI_ISL_1503065	Gorgas Memorial Laboratory of Health Studies	Gorgas Memorial Laboratory of Health Studies	Gonzalez Claudia et al
EPI_ISL_1503084	Gorgas Memorial Laboratory of Health Studies	Gorgas Memorial Laboratory of Health Studies	Gonzalez Claudia et al
EPI_ISL_1503086	Gorgas Memorial Laboratory of Health Studies	Gorgas Memorial Laboratory of Health Studies	Gonzalez Claudia et al
EPI_ISL_1502867	Hospital Regional Anita Moreno Los Santos	Gorgas Memorial Laboratory of Health Studies	Gonzalez Claudia et al
EPI_ISL_1502879	Gorgas Memorial Laboratory of Health Studies	Gorgas Memorial Laboratory of Health Studies	Gonzalez Claudia et al
EPI_ISL_1502882	Gorgas Memorial Laboratory of Health Studies	Gorgas Memorial Laboratory of Health Studies	Gonzalez Claudia et al
EPI_ISL_1502883	Gorgas Memorial Laboratory of Health Studies	Gorgas Memorial Laboratory of Health Studies	Gonzalez Claudia et al
EPI_ISL_1502884	Gorgas Memorial Laboratory of Health Studies	Gorgas Memorial Laboratory of Health Studies	Gonzalez Claudia et al
EPI_ISL_414476	MSHS Clinical Microbiology Laboratories	MSHS Pathogen Surveillance Program	Gopi Patel et al

EPI_ISL_1273391	National Reference Laboratory - Ministry of Health Maseru Lesotho	National Institute for Communicable Diseases of the National Health Laboratory Service	Gorova V et al
EPI_ISL_1273392	National Reference Laboratory - Ministry of Health Maseru Lesotho	National Institute for Communicable Diseases of the National Health Laboratory Service	Gorova V et al
EPI_ISL_1273393	National Reference Laboratory - Ministry of Health Maseru Lesotho	National Institute for Communicable Diseases of the National Health Laboratory Service	Gorova V et al
EPI_ISL_1273394	National Reference Laboratory - Ministry of Health Maseru Lesotho	National Institute for Communicable Diseases of the National Health Laboratory Service	Gorova V et al
EPI_ISL_1273395	National Reference Laboratory - Ministry of Health Maseru Lesotho	National Institute for Communicable Diseases of the National Health Laboratory Service	Gorova V et al
EPI_ISL_1273399	National Reference Laboratory - Ministry of Health Maseru Lesotho	National Institute for Communicable Diseases of the National Health Laboratory Service	Gorova V et al
EPI_ISL_1273401	National Reference Laboratory - Ministry of Health Maseru Lesotho	National Institute for Communicable Diseases of the National Health Laboratory Service	Gorova V et al
EPI_ISL_1273406	National Reference Laboratory - Ministry of Health Maseru Lesotho	National Institute for Communicable Diseases of the National Health Laboratory Service	Gorova V et al
EPI_ISL_1273407	National Reference Laboratory - Ministry of Health Maseru Lesotho	National Institute for Communicable Diseases of the National Health Laboratory Service	Gorova V et al
EPI_ISL_1273408	National Reference Laboratory - Ministry of Health Maseru Lesotho	National Institute for Communicable Diseases of the National Health Laboratory Service	Gorova V et al
EPI_ISL_427731	Centre for Infectious Diseases and Microbiology Public Health	NSW Health Pathology - Institute of Clinical Pathology and Medical Research; Westmead Hospital; University of Sydney	Gray K et al
EPI_ISL_999032	Institut Pasteur de Guinée	Institut Pasteur de Dakar	Grayo Solene et al
EPI_ISL_416485	Servicio de Microbiologia. Consorcio Hospital General Universitario de Valencia	Sequencing and Bioinformatics Service and Molecular Epidemiology Research Group. FISABIO-Public Health	Griselda De Marco et al
EPI_ISL_1055164	National Virus Reference Laboratory	National Virus Reference Laboratory	Guerrino Macori et al
EPI_ISL_426361	Instituto Nacional de Ciencias Medicas y Nutricion Salvador Zubiran	Instituto Nacional de Ciencias Medicas y Nutricion Salvador Zubiran	Guillermo M. Ruiz-Palacios et al
EPI_ISL_538142	Servicio de Microbiología y Parasitología clínica. UCEIMP. Hospital Universitario Virgen del Rocío/IBIS/CSIC/US	SeqCOVID-SPAIN consortium/IBV(CSIC)	Guillermo Martín Gutiérrez et al
EPI_ISL_610162	RSUD Dr. Tjitrowardojo	Genetics Working Group (Pokja Genetik) Faculty of Medicine, Public Health and Nursing Universitas Gadjah Mada (FK-KMK UGM); Disease Investigation Center Wates Ministry of Agriculture Indonesia; Department of Microbiology FK-KMK UGM; Laboratorium Diagnostik Yayasan Tahija World Mosquito Program (WMP) Yogyakarta Center for Tropical Medicine FK-KMK UGM; Integrated Research Center FK-KMK UGM; Department of Computer Science and Electronics FMIPA UGM; Balai Besar Teknik Kesehatan Lingkungan dan Pengendalian Penyakit (BBTKLPP) Yogyakarta	Gunadi et al
EPI_ISL_516806	Rumah Sakit PKU Gamping	Genetics Working Group (Pokja Genetik) Faculty of Medicine, Public Health and Nursing Universitas Gadjah Mada (FK-KMK UGM); Disease Investigation Center Wates Ministry of Agriculture Indonesia; Department of Microbiology FK-KMK UGM; Laboratorium Diagnostik Yayasan Tahija World Mosquito Program (WMP) Yogyakarta Center for Tropical Medicine FK-KMK UGM; Integrated Research center FK-KMK UGM; Department of Computer Science and Electronics FMIPA UGM	Gunadi et al

EPI_ISL_537558	UCLA Pathology Clinical Microbiology Lab	Kruglyak Lab	Guo et al.
EPI_ISL_509615	Servicio de Microbiología. Hospital Universitario Donostia. OSI Donostialdea. Área de Enfermedades Infecciosas, Grupo de Infección Respiratoria y Resistencia Antimicrobiana. Instituto de Investigación Sanitaria Biodonostia	SeqCOVID-SPAIN consortium/IBV(CSIC)	Gustavo Cilla et al
EPI_ISL_509610	Servicio de Microbiología. Hospital Universitario Donostia. OSI Donostialdea. Área de Enfermedades Infecciosas, Grupo de Infección Respiratoria y Resistencia Antimicrobiana. Instituto de Investigación Sanitaria Biodonostia	SeqCOVID-SPAIN consortium/IBV(CSIC)	Gustavo Cilla et al
EPI_ISL_565921	Servicio de Microbiología. Hospital Universitario Donostia. OSI Donostialdea. Área de Enfermedades Infecciosas, Grupo de Infección Respiratoria y Resistencia Antimicrobiana. Instituto de Investigación Sanitaria Biodonostia	SeqCOVID-SPAIN consortium/IBV(CSIC)	Gustavo Cilla et al
EPI_ISL_528637	LVM/UFRJ	Bioinformatics Laboratory / LNCC	Gustavo D. P. Silva et al
EPI_ISL_470877	Department for Virology, Molecular Biology and Genome Research, R. G. Lugar Center for Public Health Research, National Center for Disease Control and Public Health (NCDC) of Georgia.	Department for Virology, Molecular Biology and Genome Research, R. G. Lugar Center for Public Health Research, National Center for Disease Control and Public Health (NCDC) of Georgia.	Gvantsa Brachveli et al
EPI_ISL_1048367	Department for Virology, Molecular Biology and Genome Research, R. G. Lugar Center for Public Health Research, National Center for Disease Control and Public Health (NCDC) of Georgia.	Department for Virology, Molecular Biology and Genome Research, R. G. Lugar Center for Public Health Research, National Center for Disease Control and Public Health (NCDC) of Georgia.	Gvantsa Brachveli et al
EPI_ISL_1005231	Vilnius university hospital Santaros Klinikos, Center of Laboratory Medicine	Vilnius university hospital Santaros Klinikos, Center of Laboratory Medicine	Gytis Dudas et al
EPI_ISL_1005225	Vilnius university hospital Santaros Klinikos, Center of Laboratory Medicine	Vilnius university hospital Santaros Klinikos, Center of Laboratory Medicine	Gytis Dudas et al
EPI_ISL_1005227	Vilnius university hospital Santaros Klinikos, Center of Laboratory Medicine	Vilnius university hospital Santaros Klinikos, Center of Laboratory Medicine	Gytis Dudas et al
EPI_ISL_1005228	Vilnius university hospital Santaros Klinikos, Center of Laboratory Medicine	Vilnius university hospital Santaros Klinikos, Center of Laboratory Medicine	Gytis Dudas et al
EPI_ISL_1005230	Vilnius university hospital Santaros Klinikos, Center of Laboratory Medicine	Vilnius university hospital Santaros Klinikos, Center of Laboratory Medicine	Gytis Dudas et al
EPI_ISL_1661599	Life Sciences Center, Vilnius University	Vilnius University Hospital Santaros Klinikos, Center of Laboratory Medicine	Gytis Dudas et al
EPI_ISL_1661660	Vilnius University Hospital Santaros Klinikos	Vilnius University Hospital Santaros Klinikos, Center of Laboratory Medicine	Gytis Dudas et al
EPI_ISL_1661664	UAB Medicina practica laboratorija	Vilnius University Hospital Santaros Klinikos, Center of Laboratory Medicine	Gytis Dudas et al
EPI_ISL_1661745	Vilnius University Hospital Santaros Klinikos	Vilnius University Hospital Santaros Klinikos, Center of Laboratory Medicine	Gytis Dudas et al
EPI_ISL_1661761	Anteja laboratorija (UAB Diagnostikos laboratorija)	Vilnius University Hospital Santaros Klinikos, Center of Laboratory Medicine	Gytis Dudas et al
EPI_ISL_1133254	University of Chittagong	Central Biological Research Laboratory and Department of Biochemistry and Molecular Biology	H. M. Abdullah Al Masud et al
EPI_ISL_735500	University of Chittagong	Central Biological Research Laboratory and Department of Biochemistry and Molecular Biology	H. M. Abdullah Al Masud et al
EPI_ISL_476559	unknown	Laboratoire Sciences et Technologies de la Santé (STS) Institut Supérieur des Sciences de la Santé Université Hassan 1er, Settat, Morocco	Hajar Lemriss et al
EPI_ISL_1107189	Laboratoire central de Virologie	Laboratoire de Biotechnologie	Hakima Kabbaj et al
EPI_ISL_1116470	Laboratoire central de Virologie	Laboratoire de Biotechnologie	Hakima Kabbaj et al
EPI_ISL_1117289	Laboratoire central de Virologie	Laboratoire de Biotechnologie	Hakima Kabbaj et al
EPI_ISL_754179	Specialized Lab for COVID-19 Detection, Department of Genetic Engineering and Biotechnology	Specialized Lab for COVID-19 Detection, Department of Genetic Engineering and Biotechnology	Hammadul Hoque et al

EPI_ISL_1257859	KKP Soekarno Hatta	National Institute of Health Research and Development	Hana Apsari Pawestri et al
EPI_ISL_458286	unknown	Bundeswehr Institute of Microbiology	Handrick et al
EPI_ISL_833336	Bureau of Quarantine	Research Institute for Tropical Medicine	Hannah Leah Morito et al
EPI_ISL_833338	Siniloan Rural Health Unit	Research Institute for Tropical Medicine	Hannah Leah Morito et al
EPI_ISL_833334	Batangas City Health Office	Research Institute for Tropical Medicine	Hannah Leah Morito et al
EPI_ISL_833333	Batangas City Health Office	Research Institute for Tropical Medicine	Hannah Leah Morito et al
EPI_ISL_1069188	Lumban Rural Health Unit	Research Institute for Tropical Medicine	Hannah Leah Morito et al
EPI_ISL_1332747	Lighthouse Lab in Glasgow	Wellcome Sanger Institute for the COVID-19 Genomics UK (COG-UK) Consortium	Harper VanSteenhouse et al
EPI_ISL_532970	Lighthouse Lab in Glasgow	Wellcome Sanger Institute for the COVID-19 Genomics UK (COG-UK) consortium	Harper VanSteenhouse et al
EPI_ISL_549568	Lighthouse Lab in Glasgow	Wellcome Sanger Institute for the COVID-19 Genomics UK (COG-UK) consortium	Harper VanSteenhouse et al
EPI_ISL_567404	Lighthouse Lab in Glasgow	Wellcome Sanger Institute for the COVID-19 Genomics UK (COG-UK) consortium	Harper VanSteenhouse et al
EPI_ISL_567449	Lighthouse Lab in Glasgow	Wellcome Sanger Institute for the COVID-19 Genomics UK (COG-UK) consortium	Harper VanSteenhouse et al
EPI_ISL_567526	Lighthouse Lab in Glasgow	Wellcome Sanger Institute for the COVID-19 Genomics UK (COG-UK) consortium	Harper VanSteenhouse et al
EPI_ISL_599760	Lighthouse Lab in Glasgow	Wellcome Sanger Institute for the COVID-19 Genomics UK (COG-UK) consortium	Harper VanSteenhouse et al
EPI_ISL_587521	Lighthouse Lab in Glasgow	Wellcome Sanger Institute for the COVID-19 Genomics UK (COG-UK) consortium	Harper VanSteenhouse et al
EPI_ISL_641980	Lighthouse Lab in Glasgow	Wellcome Sanger Institute for the COVID-19 Genomics UK (COG-UK) Consortium	Harper VanSteenhouse et al
EPI_ISL_760740	Lighthouse Lab in Glasgow	Wellcome Sanger Institute for the COVID-19 Genomics UK (COG-UK) Consortium	Harper VanSteenhouse et al
EPI_ISL_1507943	Lighthouse Lab in Glasgow	Wellcome Sanger Institute for the COVID-19 Genomics UK (COG-UK) Consortium	Harper VanSteenhouse et al
EPI_ISL_1634819	Lighthouse Lab in Glasgow	Wellcome Sanger Institute for the COVID-19 Genomics UK (COG-UK) Consortium	Harper VanSteenhouse et al
EPI_ISL_1634918	Lighthouse Lab in Glasgow	Wellcome Sanger Institute for the COVID-19 Genomics UK (COG-UK) Consortium	Harper VanSteenhouse et al
EPI_ISL_415578	BCCDC Public Health Laboratory	BCCDC Public Health Laboratory	Harrigan et al
EPI_ISL_415577	BCCDC Public Health Laboratory	BCCDC Public Health Laboratory	Harrigan et al
EPI_ISL_1469256	RS Khusus Paru Karawang	National Institute of Health Research and Development	Hartanti Dian Ikawati et al
EPI_ISL_1257851	BBTKLPP Banjarbaru	National Institute of Health Research and Development	Hartanti Dian Ikawati et al
EPI_ISL_539573	Centre de Recherches Medicales de Lambarene (CERMEL)	Department of Emerging Infectious Diseases, Institute of Tropical Medicine, Nagasaki University	Haruka Abe et al

EPI_ISL_539574	Centre de Recherches Medicales de Lambarene (CERMEL)	Department of Emerging Infectious Diseases, Institute of Tropical Medicine, Nagasaki University	Haruka Abe et al
EPI_ISL_539575	Centre de Recherches Medicales de Lambarene (CERMEL)	Department of Emerging Infectious Diseases, Institute of Tropical Medicine, Nagasaki University	Haruka Abe et al
EPI_ISL_539576	Centre de Recherches Medicales de Lambarene (CERMEL)	Department of Emerging Infectious Diseases, Institute of Tropical Medicine, Nagasaki University	Haruka Abe et al
EPI_ISL_1498152	Institute of Epidemiology, Disease Control and Research (IEDCR)	Institute for Developing Science and Health Initiatives (ideSHi)	Hassan Afrad et al
EPI_ISL_1498142	Institute of Epidemiology, Disease Control and Research (IEDCR)	Institute for Developing Science and Health Initiatives (ideSHi)	Hassan Afrad et al
EPI_ISL_1498146	Institute of Epidemiology, Disease Control and Research (IEDCR)	Institute for Developing Science and Health Initiatives (ideSHi)	Hassan Afrad et al
EPI_ISL_1582394	Institute of Epidemiology, Disease Control and Research (IEDCR)	Institute for Developing Science and Health Initiatives (ideSHi)	Hassan Afrad et al
EPI_ISL_1582397	Institute of Epidemiology, Disease Control and Research (IEDCR)	Institute for Developing Science and Health Initiatives (ideSHi)	Hassan Afrad et al
EPI_ISL_1582393	Institute of Epidemiology, Disease Control and Research (IEDCR)	Institute for Developing Science and Health Initiatives (ideSHi)	Hassan Afrad et al
EPI_ISL_1587441	Institute for Developing Science and Health Initiatives (ideSHi)	Institute for Developing Science and Health Initiatives (ideSHi)	Hassan Afrad et al
EPI_ISL_1587432	Institute for Developing Science and Health Initiatives (ideSHi)	Institute for Developing Science and Health Initiatives (ideSHi)	Hassan Afrad et al
EPI_ISL_746318	Genome Center	Genome Center	Hassan M. Al-Emran et al
EPI_ISL_812793	Genomics Program, Children Cancer Hospital	Genomics Program, Children Cancer Hospital	Hatem et al
EPI_ISL_812800	Genomics Program, Children Cancer Hospital	Genomics Program, Children Cancer Hospital	Hatem et al
EPI_ISL_812812	Genomics Program, Children Cancer Hospital	Genomics Program, Children Cancer Hospital	Hatem et al
EPI_ISL_812815	Genomics Program, Children Cancer Hospital	Genomics Program, Children Cancer Hospital	Hatem et al
EPI_ISL_812820	Genomics Program, Children Cancer Hospital	Genomics Program, Children Cancer Hospital	Hatem et al
EPI_ISL_812821	Genomics Program, Children Cancer Hospital	Genomics Program, Children Cancer Hospital	Hatem et al
EPI_ISL_812824	Genomics Program, Children Cancer Hospital	Genomics Program, Children Cancer Hospital	Hatem et al
EPI_ISL_812829	Genomics Program, Children Cancer Hospital	Genomics Program, Children Cancer Hospital	Hatem et al
EPI_ISL_812837	Genomics Program, Children Cancer Hospital	Genomics Program, Children Cancer Hospital	Hatem et al
EPI_ISL_812842	Genomics Program, Children Cancer Hospital	Genomics Program, Children Cancer Hospital	Hatem et al
EPI_ISL_812846	Genomics Program, Children Cancer Hospital	Genomics Program, Children Cancer Hospital	Hatem et al
EPI_ISL_812864	Genomics Program, Children Cancer Hospital	Genomics Program, Children Cancer Hospital	Hatem et al
EPI_ISL_812872	Genomics Program, Children Cancer Hospital	Genomics Program, Children Cancer Hospital	Hatem et al
EPI_ISL_1647215	Dept. of Microbiology and Infection Control, Akershus University Hospital HF	Dept. of Microbiology and Infection Control, Akershus University Hospital HF	Hege Vangstein Aamot et al
EPI_ISL_1647217	Dept. of Microbiology and Infection Control, Akershus University Hospital HF	Dept. of Microbiology and Infection Control, Akershus University Hospital HF	Hege Vangstein Aamot et al
EPI_ISL_1647256	Dept. of Microbiology and Infection Control, Akershus University Hospital HF	Dept. of Microbiology and Infection Control, Akershus University Hospital HF	Hege Vangstein Aamot et al

EPI_ISL_1647260	Dept. of Microbiology and Infection Control, Akershus University Hospital HF	Dept. of Microbiology and Infection Control, Akershus University Hospital HF	Hege Vangstein Aamot et al
EPI_ISL_665258	Dept. of Microbiology and Infection Control, Akershus University Hospital HF	Dept. of Microbiology and Infection Control, Akershus University Hospital HF	Hege Vangstein Aamot et al
EPI_ISL_665259	Dept. of Microbiology and Infection Control, Akershus University Hospital HF	Dept. of Microbiology and Infection Control, Akershus University Hospital HF	Hege Vangstein Aamot et al
EPI_ISL_665260	Dept. of Microbiology and Infection Control, Akershus University Hospital HF	Dept. of Microbiology and Infection Control, Akershus University Hospital HF	Hege Vangstein Aamot et al
EPI_ISL_666596	Dept. of Microbiology and Infection Control, Akershus University Hospital HF	Dept. of Microbiology and Infection Control, Akershus University Hospital HF	Hege Vangstein Aamot et al
EPI_ISL_666599	Dept. of Microbiology and Infection Control, Akershus University Hospital HF	Dept. of Microbiology and Infection Control, Akershus University Hospital HF	Hege Vangstein Aamot et al
EPI_ISL_666601	Dept. of Microbiology and Infection Control, Akershus University Hospital HF	Dept. of Microbiology and Infection Control, Akershus University Hospital HF	Hege Vangstein Aamot et al
EPI_ISL_666603	Dept. of Microbiology and Infection Control, Akershus University Hospital HF	Dept. of Microbiology and Infection Control, Akershus University Hospital HF	Hege Vangstein Aamot et al
EPI_ISL_666604	Dept. of Microbiology and Infection Control, Akershus University Hospital HF	Dept. of Microbiology and Infection Control, Akershus University Hospital HF	Hege Vangstein Aamot et al
EPI_ISL_445240	Ulltuna Vardcentral	The Public Health Agency of Sweden	Heidi Lindback et al
EPI_ISL_450824	Ulltuna Vardcentral	The Public Health Agency of Sweden	Heidi Lindback et al
EPI_ISL_1492070	National Institute of Public Health	National Institute of Public Health	Helena Jirincova et al
EPI_ISL_1492117	National Institute of Public Health	National Institute of Public Health	Helena Jirincova et al
EPI_ISL_1588586	National Institute of Public Health	National Institute of Public Health	Helena Jirincova et al
EPI_ISL_1588589	National Institute of Public Health	National Institute of Public Health	Helena Jirincova et al
EPI_ISL_895763	Molecular biology division, Institute of Clinical Biochemistry and Diagnostics, Charles University, Faculty of Medicine in Hradec Králové and University Hospital Hradec Králové	Molecular biology division, Institute of Clinical Biochemistry and Diagnostics, Charles University, Faculty of Medicine in Hradec Králové and University Hospital Hradec Králové	Helena Kovaříková et al
EPI_ISL_895751	Molecular biology division, Institute of Clinical Biochemistry and Diagnostics, Charles University, Faculty of Medicine in Hradec Králové and University Hospital Hradec Králové	Molecular biology division, Institute of Clinical Biochemistry and Diagnostics, Charles University, Faculty of Medicine in Hradec Králové and University Hospital Hradec Králové	Helena Kovaříková et al
EPI_ISL_895781	Molecular biology division, Institute of Clinical Biochemistry and Diagnostics, Charles University, Faculty of Medicine in Hradec Králové and University Hospital Hradec Králové	Molecular biology division, Institute of Clinical Biochemistry and Diagnostics, Charles University, Faculty of Medicine in Hradec Králové and University Hospital Hradec Králové	Helena Kovaříková et al
EPI_ISL_895784	Molecular biology division, Institute of Clinical Biochemistry and Diagnostics, Charles University, Faculty of Medicine in Hradec Králové and University Hospital Hradec Králové	Molecular biology division, Institute of Clinical Biochemistry and Diagnostics, Charles University, Faculty of Medicine in Hradec Králové and University Hospital Hradec Králové	Helena Kovaříková et al
EPI_ISL_895827	Molecular biology division, Institute of Clinical Biochemistry and Diagnostics, Charles University, Faculty of Medicine in Hradec Králové and University Hospital Hradec Králové	Molecular biology division, Institute of Clinical Biochemistry and Diagnostics, Charles University, Faculty of Medicine in Hradec Králové and University Hospital Hradec Králové	Helena Kovaříková et al
EPI_ISL_895737	Molecular biology division, Institute of Clinical Biochemistry and Diagnostics, Charles University, Faculty of Medicine in Hradec Králové and University Hospital Hradec Králové	Molecular biology division, Institute of Clinical Biochemistry and Diagnostics, Charles University, Faculty of Medicine in Hradec Králové and University Hospital Hradec Králové	Helena Kovaříková et al

EPI_ISL_895740	Molecular biology division, Institute of Clinical Biochemistry and Diagnostics, Charles University, Faculty of Medicine in Hradec Králové and University Hospital Hradec Králové	Molecular biology division, Institute of Clinical Biochemistry and Diagnostics, Charles University, Faculty of Medicine in Hradec Králové and University Hospital Hradec Králové	Helena Kovaříková et al
EPI_ISL_895787	Molecular biology division, Institute of Clinical Biochemistry and Diagnostics, Charles University, Faculty of Medicine in Hradec Králové and University Hospital Hradec Králové	Molecular biology division, Institute of Clinical Biochemistry and Diagnostics, Charles University, Faculty of Medicine in Hradec Králové and University Hospital Hradec Králové	Helena Kovaříková et al
EPI_ISL_895789	Molecular biology division, Institute of Clinical Biochemistry and Diagnostics, Charles University, Faculty of Medicine in Hradec Králové and University Hospital Hradec Králové	Molecular biology division, Institute of Clinical Biochemistry and Diagnostics, Charles University, Faculty of Medicine in Hradec Králové and University Hospital Hradec Králové	Helena Kovaříková et al
EPI_ISL_895819	Molecular biology division, Institute of Clinical Biochemistry and Diagnostics, Charles University, Faculty of Medicine in Hradec Králové and University Hospital Hradec Králové	Molecular biology division, Institute of Clinical Biochemistry and Diagnostics, Charles University, Faculty of Medicine in Hradec Králové and University Hospital Hradec Králové	Helena Kovaříková et al
EPI_ISL_895729	Molecular biology division, Institute of Clinical Biochemistry and Diagnostics, Charles University, Faculty of Medicine in Hradec Králové and University Hospital Hradec Králové	Molecular biology division, Institute of Clinical Biochemistry and Diagnostics, Charles University, Faculty of Medicine in Hradec Králové and University Hospital Hradec Králové	Helena Kovaříková et al
EPI_ISL_895730	Molecular biology division, Institute of Clinical Biochemistry and Diagnostics, Charles University, Faculty of Medicine in Hradec Králové and University Hospital Hradec Králové	Molecular biology division, Institute of Clinical Biochemistry and Diagnostics, Charles University, Faculty of Medicine in Hradec Králové and University Hospital Hradec Králové	Helena Kovaříková et al
EPI_ISL_895813	Molecular biology division, Institute of Clinical Biochemistry and Diagnostics, Charles University, Faculty of Medicine in Hradec Králové and University Hospital Hradec Králové	Molecular biology division, Institute of Clinical Biochemistry and Diagnostics, Charles University, Faculty of Medicine in Hradec Králové and University Hospital Hradec Králové	Helena Kovaříková et al
EPI_ISL_895814	Molecular biology division, Institute of Clinical Biochemistry and Diagnostics, Charles University, Faculty of Medicine in Hradec Králové and University Hospital Hradec Králové	Molecular biology division, Institute of Clinical Biochemistry and Diagnostics, Charles University, Faculty of Medicine in Hradec Králové and University Hospital Hradec Králové	Helena Kovaříková et al
EPI_ISL_895792	Molecular biology division, Institute of Clinical Biochemistry and Diagnostics, Charles University, Faculty of Medicine in Hradec Králové and University Hospital Hradec Králové	Molecular biology division, Institute of Clinical Biochemistry and Diagnostics, Charles University, Faculty of Medicine in Hradec Králové and University Hospital Hradec Králové	Helena Kovaříková et al
EPI_ISL_895793	Molecular biology division, Institute of Clinical Biochemistry and Diagnostics, Charles University, Faculty of Medicine in Hradec Králové and University Hospital Hradec Králové	Molecular biology division, Institute of Clinical Biochemistry and Diagnostics, Charles University, Faculty of Medicine in Hradec Králové and University Hospital Hradec Králové	Helena Kovaříková et al
EPI_ISL_895834	Molecular biology division, Institute of Clinical Biochemistry and Diagnostics, Charles University, Faculty of Medicine in Hradec Králové and University Hospital Hradec Králové	Molecular biology division, Institute of Clinical Biochemistry and Diagnostics, Charles University, Faculty of Medicine in Hradec Králové and University Hospital Hradec Králové	Helena Kovaříková et al
EPI_ISL_895824	Molecular biology division, Institute of Clinical Biochemistry and Diagnostics, Charles University, Faculty of Medicine in Hradec Králové and University Hospital Hradec Králové	Molecular biology division, Institute of Clinical Biochemistry and Diagnostics, Charles University, Faculty of Medicine in Hradec Králové and University Hospital Hradec Králové	Helena Kovaříková et al
EPI_ISL_895815	Molecular biology division, Institute of Clinical Biochemistry and Diagnostics, Charles University, Faculty of Medicine in Hradec Králové and University Hospital Hradec Králové	Molecular biology division, Institute of Clinical Biochemistry and Diagnostics, Charles University, Faculty of Medicine in Hradec Králové and University Hospital Hradec Králové	Helena Kovaříková et al
EPI_ISL_895761	Molecular biology division, Institute of Clinical Biochemistry and Diagnostics, Charles University, Faculty of Medicine in Hradec Králové and University Hospital Hradec Králové	Molecular biology division, Institute of Clinical Biochemistry and Diagnostics, Charles University, Faculty of Medicine in Hradec Králové and University Hospital Hradec Králové	Helena Kovaříková et al
EPI_ISL_700167	Hematopathology Laboratory, ACTREC, TMC	Hematopathology Laboratory, ACTREC, TMC	Hematopathology Laboratory et al
EPI_ISL_978550	Centre de Virologie des Maladies infectueuses Tropicales	Functional Genomic Platform UATRS-biology, CNRST	Hemlali Mouhssine et al
EPI_ISL_1542694	Pandemic Response Lab - NYC	Pandemic Response Lab, R&D	Henry Lee et al

EPI_ISL_1543367	Pandemic Response Lab - NYC	Pandemic Response Lab, R&D	Henry Lee et al
EPI_ISL_1633869	Pandemic Response Lab - NYC	Pandemic Response Lab, R&D	Henry Lee et al
EPI_ISL_1633841	Pandemic Response Lab - NYC	Pandemic Response Lab, R&D	Henry Lee et al
EPI_ISL_1633723	Pandemic Response Lab - NYC	Pandemic Response Lab, R&D	Henry Lee et al
EPI_ISL_483035	Medical Ain Shams Research Institute (MASRI), Ain Shams University	Medical Ain Shams Research Institute (MASRI), Ain Shams University	Hesham Elghazaly et al
EPI_ISL_482765	Medical Ain Shams Research Institute (MASRI), Ain Shams University	Medical Ain Shams Research Institute (MASRI), Ain Shams University	Hesham Elghazaly et al
EPI_ISL_483036	Medical Ain Shams Research Institute (MASRI), Ain Shams University	Medical Ain Shams Research Institute (MASRI), Ain Shams University	Hesham Elghazaly et al
EPI_ISL_1109485	Medical Ain Shams Research Institute (MASRI), Ain Shams University	Medical Ain Shams Research Institute (MASRI), Ain Shams University	Hesham Elghazaly et al
EPI_ISL_471456	Centre de Virologie des Maladies Tropicales	Functional Genomic Platform/Service Analyses Biologique/UATRS/ Centre National Pour la Recherche Scientifique Et Technique (CNRST)	Hicham EL ANNAZ et al
EPI_ISL_471458	Centre de Virologie des Maladies Tropicales	Functional Genomic Platform/Service Analyses Biologique/UATRS/ Centre National Pour la Recherche Scientifique Et Technique (CNRST)	Hicham EL ANNAZ et al
EPI_ISL_471460	Centre de Virologie des Maladies Tropicales	Functional Genomic Platform/Service Analyses Biologique/UATRS/ Centre National Pour la Recherche Scientifique Et Technique (CNRST)	Hicham EL ANNAZ et al
EPI_ISL_1591658	Instituto Nacional de Medicina Genomica	Instituto Nacional de Medicina Genomica	Hidalgo-Miranda A et al
EPI_ISL_1585919	Instituto Nacional de Medicina Genomica	Instituto Nacional de Medicina Genomica	Hidalgo-Miranda A et al
EPI_ISL_1585916	Instituto Nacional de Medicina Genomica	Instituto Nacional de Medicina Genomica	Hidalgo-Miranda A et al
EPI_ISL_1585918	Instituto Nacional de Medicina Genomica	Instituto Nacional de Medicina Genomica	Hidalgo-Miranda A et al
EPI_ISL_933525	Instituto Nacional de Medicina Genomica	Instituto Nacional de Medicina Genomica	Hidalgo-Miranda A et al
EPI_ISL_955192	Instituto Nacional de Medicina Genomica	Instituto Nacional de Medicina Genomica	Hidalgo-Miranda A et al
EPI_ISL_955207	Instituto Nacional de Medicina Genomica	Instituto Nacional de Medicina Genomica	Hidalgo-Miranda A et al
EPI_ISL_1005563	Instituto Nacional de Medicina Genomica	Instituto Nacional de Medicina Genomica	Hidalgo-Miranda A et al
EPI_ISL_522942	Instituto Nacional de Medicina Genómica	Instituto Nacional de Medicina Genómica	Hidalgo-Miranda A et al
EPI_ISL_1628603	InDRE	Instituto Nacional de Medicina Genomica	Hidalgo-Miranda A et al
EPI_ISL_574623	BTKLPP Kelas I Manado	Eijkman Institute for Molecular Biology, Ministry of Research and Technology/National Agency for Research and Innovation	Hidayat Trimarsanto et al
EPI_ISL_418275	University Hospital Basel, Clinical Virology	University Hospital Basel, Clinical Bacteriology	Hirsch et al
EPI_ISL_418273	University Hospital Basel, Clinical Virology	University Hospital Basel, Labormedizin	Hirsch et al
EPI_ISL_1390768	Ohio Department of Health Laboratory	Ohio Department of Health Laboratory	Holmes et al
EPI_ISL_824389	Ohio Department of Health Laboratory	Ohio Department of Health Laboratory	Holmes et al
EPI_ISL_909772	National Institute of Health Research and Development	National Institute of Health Research and Development	Holy Arif Wibowo et al

EPI_ISL_1096141	Departamento de Cuidados Intensivos-Unidad de Investigación Médica en Inmunoquímica, UMAE Hospital de Especialidades “Bernardo Sepúlveda Gutiérrez”, Centro Médico Nacional Siglo XXI, Instituto Mexicano del Seguro Social (IMSS), México.	Laboratorio de Secuenciación-Centro de Instrumentos, IMSS México /Unidad de Investigación Médica en Inmunoquímica, UMAE Hospital de Especialidades “Bernardo Sepúlveda Gutiérrez”, Centro Médico Nacional Siglo XXI, Instituto Mexicano del Seguro Social (IMSS), México.	Horacio Zamudio-Meza et al
EPI_ISL_591270	National Institute for Viral Disease Control and Prevention, China CDC	National Institute for Viral Disease Control and Prevention, China CDC	Huilai Ma et al
EPI_ISL_591271	National Institute for Viral Disease Control and Prevention, China CDC	National Institute for Viral Disease Control and Prevention, China CDC	Huilai Ma et al
EPI_ISL_539496	Hospital Nostra Senyora de Meritxell	Instituto de Salud Carlos III	Iglesias-Caballero et al
EPI_ISL_455314	Hospital Virgen del Rocio	Instituto de Salud Carlos III	Iglesias-Caballero et al
EPI_ISL_802730	Hospital Clínic de Barcelona	Instituto de Salud Carlos III	Iglesias-Caballero et al
EPI_ISL_539558	Hospital San Pedro de Alcántara	Instituto de Salud Carlos III	Iglesias-Caballero et al
EPI_ISL_804972	Hospital Comarcal de Melilla	Instituto de Salud Carlos III	Iglesias-Caballero et al
EPI_ISL_455336	Hospital San Pedro	Instituto de Salud Carlos III	Iglesias-Caballero et al
EPI_ISL_794738	1-Laboratory of Microbiology, National Reference Lab, Charles Nicolle Hospital; 2-University of Tunis ElManar, Faculty of Medicine of Tunis, LR99ES09, Tunis, Tunisia	1-Clinical and Experimental Pharmacology Lab, LR16SP02, National Center of Pharmacovigilance, University of Tunis El Manar, Tunis, Tunisia. 2-Neurodegenerative diseases and psychiatric troubles, LR18SP03, Razi Hospital, University of Tunis El Manar, Tunis, Tunisia. 3- Ministry of Health, National Observatory of New and Emerging Diseases, 1006, Tunis, Tunisia	Ilhem Boutiba-Ben Boubaker et al
EPI_ISL_794736	1-Laboratory of Microbiology, National Reference Lab, Charles Nicolle Hospital; 2-University of Tunis ElManar, Faculty of Medicine of Tunis, LR99ES09, Tunis, Tunisia	1-Clinical and Experimental Pharmacology Lab, LR16SP02, National Center of Pharmacovigilance, University of Tunis El Manar, Tunis, Tunisia. 2-Neurodegenerative diseases and psychiatric troubles, LR18SP03, Razi Hospital, University of Tunis El Manar, Tunis, Tunisia. 3- Ministry of Health, National Observatory of New and Emerging Diseases, 1006, Tunis, Tunisia	Ilhem Boutiba-Ben Boubaker et al
EPI_ISL_733500	1-Laboratory of Microbiology, National Reference Lab, Charles Nicolle Hospital; 2-University of Tunis ElManar, Faculty of Medicine of Tunis, LR99ES09, Tunis, Tunisia	1-Clinical and Experimental Pharmacology Lab, LR16SP02, National Center of Pharmacovigilance, University of Tunis El Manar, Tunis, Tunisia. 2-Neurodegenerative diseases and psychiatric troubles, LR18SP03, Razi Hospital, University of Tunis El Manar, Tunis, Tunisia. 3- Ministry of Health, National Observatory of New and Emerging Diseases, 1006, Tunis, Tunisia	Ilhem Boutiba-Ben Boubaker et al
EPI_ISL_654020	Laboratory of Microbiology, National Reference Lab, Charles Nicolle Hospital; 2-University of Tunis ElManar, Faculty of Medicine of Tunis, LR99ES09, Tunis, Tunisia	1-Clinical and Experimental Pharmacology Lab, LR16SP02, National Center of Pharmacovigilance, University of Tunis El Manar, Tunis, Tunisia. 2-Neurodegenerative diseases and psychiatric troubles, LR18SP03, Razi Hospital, University of Tunis El Manar, Tunis, Tunisia. 3- Ministry of Health, National Observatory of New and Emerging Diseases, 1006, Tunis, Tunisia	Ilhem Boutiba-Ben Boubaker et al
EPI_ISL_632310	1-Laboratory of Microbiology, National Reference Lab, Charles Nicolle Hospital; 2-University of Tunis ElManar, Faculty of Medicine of Tunis, LR99ES09, Tunis, Tunisia	1-Clinical and Experimental Pharmacology Lab, LR16SP02, National Center of Pharmacovigilance, University of Tunis El Manar, Tunis, Tunisia. 2-Neurodegenerative diseases and psychiatric troubles, LR18SP03, Razi Hospital, University of Tunis El Manar, Tunis, Tunisia. 3- Ministry of Health, National Observatory of New and Emerging Diseases, 1006, Tunis, Tunisia	Ilhem Boutiba-Ben Boubaker et al
EPI_ISL_634977	1-Laboratory of Microbiology, National Reference Lab, Charles Nicolle Hospital; 2-University of Tunis ElManar, Faculty of Medicine of Tunis, LR99ES09, Tunis, Tunisia	1-Clinical and Experimental Pharmacology Lab, LR16SP02, National Center of Pharmacovigilance, University of Tunis El Manar, Tunis, Tunisia. 2-Neurodegenerative diseases and psychiatric troubles, LR18SP03, Razi Hospital, University of Tunis El Manar, Tunis, Tunisia. 3- Ministry of Health, National Observatory of New and Emerging Diseases, 1006, Tunis, Tunisia	Ilhem Boutiba-Ben Boubaker et al

EPI_ISL_635062	1-Laboratory of Microbiology, National Reference Lab, Charles Nicolle Hospital; 2-University of Tunis ElManar, Faculty of Medicine of Tunis, LR99ES09, Tunis, Tunisia	1-Clinical and Experimental Pharmacology Lab, LR16SP02, National Center of Pharmacovigilance, University of Tunis El Manar, Tunis, Tunisia. 2-Neurodegenerative diseases and psychiatric troubles, LR18SP03, Razi Hospital, University of Tunis El Manar, Tunis, Tunisia. 3- Ministry of Health, National Observatory of New and Emerging Diseases, 1006, Tunis, Tunisia	Ilhem Boutiba-Ben Boubaker et al
EPI_ISL_707700	1-Laboratory of Microbiology, National Reference Lab, Charles Nicolle Hospital; 2-University of Tunis ElManar, Faculty of Medicine of Tunis, LR99ES09, Tunis, Tunisia	1-Clinical and Experimental Pharmacology Lab, LR16SP02, National Center of Pharmacovigilance, University of Tunis El Manar, Tunis, Tunisia. 2-Neurodegenerative diseases and psychiatric troubles, LR18SP03, Razi Hospital, University of Tunis El Manar, Tunis, Tunisia. 3- Ministry of Health, National Observatory of New and Emerging Diseases, 1006, Tunis, Tunisia	Ilhem Boutiba-Ben Boubaker et al
EPI_ISL_733499	1-Laboratory of Microbiology, National Reference Lab, Charles Nicolle Hospital; 2-University of Tunis ElManar, Faculty of Medicine of Tunis, LR99ES09, Tunis, Tunisia	1-Clinical and Experimental Pharmacology Lab, LR16SP02, National Center of Pharmacovigilance, University of Tunis El Manar, Tunis, Tunisia. 2-Neurodegenerative diseases and psychiatric troubles, LR18SP03, Razi Hospital, University of Tunis El Manar, Tunis, Tunisia. 3- Ministry of Health, National Observatory of New and Emerging Diseases, 1006, Tunis, Tunisia	Ilhem Boutiba-Ben Boubaker et al
EPI_ISL_707792	1-Laboratory of Microbiology, National Reference Lab, Charles Nicolle Hospital; 2-University of Tunis ElManar, Faculty of Medicine of Tunis, LR99ES09, Tunis, Tunisia	1-Clinical and Experimental Pharmacology Lab, LR16SP02, National Center of Pharmacovigilance, University of Tunis El Manar, Tunis, Tunisia. 2-Neurodegenerative diseases and psychiatric troubles, LR18SP03, Razi Hospital, University of Tunis El Manar, Tunis, Tunisia. 3- Ministry of Health, National Observatory of New and Emerging Diseases, 1006, Tunis, Tunisia	Ilhem Boutiba-Ben Boubaker et al
EPI_ISL_763065	1-Laboratory of Microbiology, National Reference Lab, Charles Nicolle Hospital; 2-University of Tunis ElManar, Faculty of Medicine of Tunis, LR99ES09, Tunis, Tunisia	1-Clinical and Experimental Pharmacology Lab, LR16SP02, National Center of Pharmacovigilance, University of Tunis El Manar, Tunis, Tunisia. 2-Neurodegenerative diseases and psychiatric troubles, LR18SP03, Razi Hospital, University of Tunis El Manar, Tunis, Tunisia. 3- Ministry of Health, National Observatory of New and Emerging Diseases, 1006, Tunis, Tunisia	Ilhem Boutiba-Ben Boubaker et al
EPI_ISL_796782	Laboratory of Microbiology, National Reference Lab, Charles Nicolle Hospital; 2-University of Tunis ElManar, Faculty of Medicine of Tunis, LR99ES09, Tunis, Tunisia	Clinical and Experimental Pharmacology Lab, LR16SP02, National Center of Pharmacovigilance, University of Tunis El Manar, Tunis, Tunisia. 2-Neurodegenerative diseases and psychiatric troubles, LR18SP03, Razi Hospital, University of Tunis El Manar, Tunis, Tunisia. 3- Ministry of Health, National Observatory of New and Emerging Diseases, 1006, Tunis, Tunisia	Ilhem Boutiba-Ben Boubaker et al
EPI_ISL_763067	1-Laboratory of Microbiology, National Reference Lab, Charles Nicolle Hospital; 2-University of Tunis ElManar, Faculty of Medicine of Tunis, LR99ES09, Tunis, Tunisia	1-Clinical and Experimental Pharmacology Lab, LR16SP02, National Center of Pharmacovigilance, University of Tunis El Manar, Tunis, Tunisia. 2-Neurodegenerative diseases and psychiatric troubles, LR18SP03, Razi Hospital, University of Tunis El Manar, Tunis, Tunisia. 3- Ministry of Health, National Observatory of New and Emerging Diseases, 1006, Tunis, Tunisia	Ilhem Boutiba-Ben Boubaker et al
EPI_ISL_480246	Genomic Laboratory (GLAB) (Conjoint lab of Health Directorate of Istanbul and Istanbul Technical University)	Genomic Laboratory (GLAB), Istanbul Technical University	Ilker Karacan et al
EPI_ISL_735272	Genomic Laboratory (GLAB) (Conjoint lab of Health Directorate of Istanbul and Istanbul Technical University)	Genomic Laboratory (GLAB), Istanbul Technical University	Ilker Karacan et al
EPI_ISL_735275	Genomic Laboratory (GLAB) (Conjoint lab of Health Directorate of Istanbul and Istanbul Technical University)	Genomic Laboratory (GLAB), Istanbul Technical University	Ilker Karacan et al
EPI_ISL_735277	Genomic Laboratory (GLAB) (Conjoint lab of Health Directorate of Istanbul and Istanbul Technical University)	Genomic Laboratory (GLAB), Istanbul Technical University	Ilker Karacan et al
EPI_ISL_480256	Genomic Laboratory (GLAB) (Conjoint lab of Health Directorate of Istanbul and Istanbul Technical University)	Genomic Laboratory (GLAB), Istanbul Technical University	Ilker Karacan et al
EPI_ISL_480271	Genomic Laboratory (GLAB) (Conjoint lab of Health Directorate of Istanbul and Istanbul Technical University)	Genomic Laboratory (GLAB), Istanbul Technical University	Ilker Karacan et al

EPI_ISL_480276	Genomic Laboratory (GLAB) (Conjoint lab of Health Directorate of Istanbul and Istanbul Technical University)	Genomic Laboratory (GLAB), Istanbul Technical University	Ilker Karacan et al
EPI_ISL_735328	Genomic Laboratory (GLAB) (Conjoint lab of Health Directorate of Istanbul and Istanbul Technical University)	Genomic Laboratory (GLAB), Istanbul Technical University	Ilker Karacan et al
EPI_ISL_735344	Genomic Laboratory (GLAB) (Conjoint lab of Health Directorate of Istanbul and Istanbul Technical University)	Genomic Laboratory (GLAB), Istanbul Technical University	Ilker Karacan et al
EPI_ISL_735352	Genomic Laboratory (GLAB) (Conjoint lab of Health Directorate of Istanbul and Istanbul Technical University)	Genomic Laboratory (GLAB), Istanbul Technical University	Ilker Karacan et al
EPI_ISL_735360	Genomic Laboratory (GLAB) (Conjoint lab of Health Directorate of Istanbul and Istanbul Technical University)	Genomic Laboratory (GLAB), Istanbul Technical University	Ilker Karacan et al
EPI_ISL_955154	Genomic Laboratory (GLAB) (Conjoint lab of Health Directorate of Istanbul and Istanbul Technical University)	Genomic Laboratory (GLAB), Istanbul Technical University	Ilker Karacan et al
EPI_ISL_419211	Central Virology Laboratory	Israel Institute for Biological Research	Inbar Cohen-Gihon et al
EPI_ISL_933929	Vilnius university hospital Santaros Klinikos, Center of Laboratory Medicine	Vilnius university hospital Santaros Klinikos, Center of Laboratory Medicine	Ingrida Olendraite et al
EPI_ISL_933939	Vilnius university hospital Santaros Klinikos, Center of Laboratory Medicine	Vilnius university hospital Santaros Klinikos, Center of Laboratory Medicine	Ingrida Olendraite et al
EPI_ISL_933949	Vilnius university hospital Santaros Klinikos, Center of Laboratory Medicine	Vilnius university hospital Santaros Klinikos, Center of Laboratory Medicine	Ingrida Olendraite et al
EPI_ISL_933959	Vilnius university hospital Santaros Klinikos, Center of Laboratory Medicine	Vilnius university hospital Santaros Klinikos, Center of Laboratory Medicine	Ingrida Olendraite et al
EPI_ISL_934211	Vilnius university hospital Santaros Klinikos, Center of Laboratory Medicine	Vilnius university hospital Santaros Klinikos, Center of Laboratory Medicine	Ingrida Olendraite et al
EPI_ISL_934272	Vilnius university hospital Santaros Klinikos, Center of Laboratory Medicine	Vilnius university hospital Santaros Klinikos, Center of Laboratory Medicine	Ingrida Olendraite et al
EPI_ISL_914881	Vilnius university hospital Santaros Klinikos, Center of Laboratory Medicine	Vilnius University Hospital Santaros Klinikos	Ingrida Olendraite et al
EPI_ISL_914882	Vilnius university hospital Santaros Klinikos, Center of Laboratory Medicine	Vilnius University Hospital Santaros Klinikos	Ingrida Olendraite et al
EPI_ISL_914884	Vilnius university hospital Santaros Klinikos, Center of Laboratory Medicine	Vilnius University Hospital Santaros Klinikos	Ingrida Olendraite et al
EPI_ISL_914886	Vilnius university hospital Santaros Klinikos, Center of Laboratory Medicine	Vilnius University Hospital Santaros Klinikos	Ingrida Olendraite et al
EPI_ISL_1591041	Servicio de Microbiología. HRU de Málaga. Servicio Andaluz de Salud	SeqCOVID-SPAIN consortium/IBV(CSIC)	Inmaculada de Toro Peinado et al
EPI_ISL_1591046	Servicio de Microbiología. HRU de Málaga. Servicio Andaluz de Salud	SeqCOVID-SPAIN consortium/IBV(CSIC)	Inmaculada de Toro Peinado et al
EPI_ISL_1591050	Servicio de Microbiología. HRU de Málaga. Servicio Andaluz de Salud	SeqCOVID-SPAIN consortium/IBV(CSIC)	Inmaculada de Toro Peinado et al
EPI_ISL_1591051	Servicio de Microbiología. HRU de Málaga. Servicio Andaluz de Salud	SeqCOVID-SPAIN consortium/IBV(CSIC)	Inmaculada de Toro Peinado et al
EPI_ISL_510063	Servicio de Microbiología. HRU de Málaga. Servicio Andaluz de Salud	SeqCOVID-SPAIN consortium/IBV(CSIC)	Inmaculada de Toro Peinado. MªConcepció
EPI_ISL_1532286	Oman-National Influenza Center	Biotechnology & OMICs Laboratory	Intisar Al-Shukri et al
EPI_ISL_1532287	Oman-National Influenza Center	Biotechnology & OMICs Laboratory	Intisar Al-Shukri et al
EPI_ISL_1532288	Oman-National Influenza Center	Biotechnology & OMICs Laboratory	Intisar Al-Shukri et al
EPI_ISL_1532289	Oman-National Influenza Center	Biotechnology & OMICs Laboratory	Intisar Al-Shukri et al
EPI_ISL_1499505	Institute of Public Health of Međimurje county	Croatian Institute of Public Health	Irena Tabain et al
EPI_ISL_1499515	Institute of Public Health of Karlovac County	Croatian Institute of Public Health	Irena Tabain et al

n Mediavilla Gradolph. Begoña Palop Borrás

EPI_ISL_1499519	Institute of Public Health of Dubrovnik-Neretva County	Croatian Institute of Public Health	Irena Tabain et al
EPI_ISL_1578483	Uversity hospital Dubrava	Croatian Institute of Public Health	Irena Tabain et al
EPI_ISL_1578501	University Hospital Centre Zagreb	Croatian Institute of Public Health	Irena Tabain et al
EPI_ISL_1591279	Institute for Public Health of Šibenik-Knin County	Croatian Institute of Public Health	Irena Tabain et al
EPI_ISL_1591311	Teaching Institute for Public Health	Croatian Institute of Public Health	Irena Tabain et al
EPI_ISL_454602	Croatian Institute of Public Health	University of Zagreb, Centre for research and knowledge transfer in biotechnology	Irena Tabain et al
EPI_ISL_1272020	University Hospital Centre Zagreb	Croatian Institute of Public Health	Irena Tabain et al
EPI_ISL_1287400	Croatian Institute of Public Health	Croatian Institute of Public Health	Irena Tabain et al
EPI_ISL_1435902	Institute of Public Health of Varazdin county	Croatian Institute of Public Health	Irena Tabain et al
EPI_ISL_1195672	Croatian Institute of Public Health	Croatian Institute of Public Health	Irena Tabain et al
EPI_ISL_1195728	Public Health Institute of Šibenik-Knin County	Croatian Institute of Public Health	Irena Tabain et al
EPI_ISL_1091196	Croatian Institute of Public Health	Croatian Institute of Public Health	Irena Tabain et al
EPI_ISL_1300659	Clinical Center, University of Sarajevo; Unit for Clinical Microbiology	Clinical Center, University of Sarajevo; Unit for Clinical Microbiology	Irma Salimović-Bešić et al
EPI_ISL_1300645	Clinical Center, University of Sarajevo; Unit for Clinical Microbiology	Clinical Center, University of Sarajevo; Unit for Clinical Microbiology	Irma Salimović-Bešić et al
EPI_ISL_1300658	Clinical Center, University of Sarajevo; Unit for Clinical Microbiology	Clinical Center, University of Sarajevo; Unit for Clinical Microbiology	Irma Salimović-Bešić et al
EPI_ISL_1300651	Clinical Center, University of Sarajevo; Unit for Clinical Microbiology	Clinical Center, University of Sarajevo; Unit for Clinical Microbiology	Irma Salimović-Bešić et al
EPI_ISL_1020189	RS Trimitra, Bogor, West java	Biosafety Level-3 Laboratory, Indonesian Institute of Sciences (LIPI)	Isa Nuryana et al
EPI_ISL_1395798	Laboratorio del Hospital Interzonal General de Agudos “Evita”	Área de Secuenciación del Laboratorio de Virología del Hospital de Niños Dr. Ricardo Gutierrez on behalf of 'Proyecto Argentino Interinstitucional de genomica de SARS-CoV-2' (PAIS Consortium)	Isabel Desimone et al
EPI_ISL_1395800	Laboratorio del Hospital Interzonal General de Agudos “Evita”	Área de Secuenciación del Laboratorio de Virología del Hospital de Niños Dr. Ricardo Gutierrez on behalf of 'Proyecto Argentino Interinstitucional de genomica de SARS-CoV-2' (PAIS Consortium)	Isabel Desimone et al
EPI_ISL_1443196	Hospital Aliança	Hospital São Rafael - IDOR	Isadora Cristina de Siqueira et al
EPI_ISL_1443197	Hospital Aliança	Hospital São Rafael - IDOR	Isadora Cristina de Siqueira et al
EPI_ISL_1443198	Hospital Aliança	Hospital São Rafael - IDOR	Isadora Cristina de Siqueira et al
EPI_ISL_636979	HP Pemba	KRISP, KZN Research Innovation and Sequencing Platform	Ismael N et al
EPI_ISL_636975	HP Pemba	KRISP, KZN Research Innovation and Sequencing Platform	Ismael N et al
EPI_ISL_636981	City of Chimoio	KRISP, KZN Research Innovation and Sequencing Platform	Ismael N et al
EPI_ISL_636977	HP Pemba	KRISP, KZN Research Innovation and Sequencing Platform	Ismael N et al
EPI_ISL_636976	HP Pemba	KRISP, KZN Research Innovation and Sequencing Platform	Ismael N et al
EPI_ISL_635778	Biolab Diagnostic Laboratories	Andersen lab at Scripps Research	Issa Abu-Dayyeh et al

EPI_ISL_635782	Biolab Diagnostic Laboratories	Andersen lab at Scripps Research	Issa Abu-Dayyeh et al
EPI_ISL_730240	Biolab Diagnostic Laboratories	Andersen lab at Scripps Research	Issa Abu-Dayyeh et al
EPI_ISL_730266	Biolab Diagnostic Laboratories	Andersen lab at Scripps Research	Issa Abu-Dayyeh et al
EPI_ISL_730378	Biolab Diagnostic Laboratories	Andersen lab at Scripps Research	Issa Abu-Dayyeh et al
EPI_ISL_730385	Biolab Diagnostic Laboratories	Andersen lab at Scripps Research	Issa Abu-Dayyeh et al
EPI_ISL_730398	Biolab Diagnostic Laboratories	Andersen lab at Scripps Research	Issa Abu-Dayyeh et al
EPI_ISL_730520	Biolab Diagnostic Laboratories	Andersen lab at Scripps Research	Issa Abu-Dayyeh et al
EPI_ISL_730521	Biolab Diagnostic Laboratories	Andersen lab at Scripps Research	Issa Abu-Dayyeh et al
EPI_ISL_730524	Biolab Diagnostic Laboratories	Andersen lab at Scripps Research	Issa Abu-Dayyeh et al
EPI_ISL_730544	Biolab Diagnostic Laboratories	Andersen lab at Scripps Research	Issa Abu-Dayyeh et al
EPI_ISL_730560	Biolab Diagnostic Laboratories	Andersen lab at Scripps Research	Issa Abu-Dayyeh et al
EPI_ISL_878498	Biolab Diagnostic Laboratories	Andersen lab at Scripps Research	Issa Abu-Dayyeh et al
EPI_ISL_878571	Biolab Diagnostic Laboratories	Andersen lab at Scripps Research	Issa Abu-Dayyeh et al
EPI_ISL_878574	Biolab Diagnostic Laboratories	Andersen lab at Scripps Research	Issa Abu-Dayyeh et al
EPI_ISL_1406151	Biolab Diagnostic Laboratories	Biolab Diagnostic Laboratories	Issa Abu-Dayyeh et al
EPI_ISL_1406179	Biolab Diagnostic Laboratories	Biolab Diagnostic Laboratories	Issa Abu-Dayyeh et al
EPI_ISL_1406183	Biolab Diagnostic Laboratories	Biolab Diagnostic Laboratories	Issa Abu-Dayyeh et al
EPI_ISL_1406189	Biolab Diagnostic Laboratories	Biolab Diagnostic Laboratories	Issa Abu-Dayyeh et al
EPI_ISL_1406195	Biolab Diagnostic Laboratories	Biolab Diagnostic Laboratories	Issa Abu-Dayyeh et al
EPI_ISL_1406239	Biolab Diagnostic Laboratories	Biolab Diagnostic Laboratories	Issa Abu-Dayyeh et al
EPI_ISL_977602	Biolab Diagnostic Laboratories	Biolab Diagnostic Laboratories	Issa Abu-Dayyeh et al
EPI_ISL_635777	Biolab Diagnostic Laboratories	Andersen lab at Scripps Research	Issa Abu-Dayyeh et al
EPI_ISL_755236	Biolab Diagnostic Laboratories	Andersen lab at Scripps Research	Issa Abu-Dayyeh et al
EPI_ISL_755237	Biolab Diagnostic Laboratories	Andersen lab at Scripps Research	Issa Abu-Dayyeh et al
EPI_ISL_755238	Biolab Diagnostic Laboratories	Andersen lab at Scripps Research	Issa Abu-Dayyeh et al
EPI_ISL_755128	Biolab Diagnostic Laboratories	Andersen lab at Scripps Research	Issa Abu-Dayyeh et al
EPI_ISL_755124	Biolab Diagnostic Laboratories	Andersen lab at Scripps Research	Issa Abu-Dayyeh et al
EPI_ISL_755253	Biolab Diagnostic Laboratories	Andersen lab at Scripps Research	Issa Abu-Dayyeh et al
EPI_ISL_755257	Biolab Diagnostic Laboratories	Andersen lab at Scripps Research	Issa Abu-Dayyeh et al
EPI_ISL_755145	Biolab Diagnostic Laboratories	Andersen lab at Scripps Research	Issa Abu-Dayyeh et al

EPI_ISL_755263	Biolab Diagnostic Laboratories	Andersen lab at Scripps Research	Issa Abu-Dayyeh et al
EPI_ISL_450188	Biolab Diagnostic Laboratories	Andersen lab at Scripps Research	Issa Abu-Dayyeh et al
EPI_ISL_450189	Biolab Diagnostic Laboratories	Andersen lab at Scripps Research	Issa Abu-Dayyeh et al
EPI_ISL_429995	Biolab Diagnostic Laboratories	Andersen lab at Scripps Research	Issa Abu-Dayyeh et al
EPI_ISL_429998	Biolab Diagnostic Laboratories	Andersen lab at Scripps Research	Issa Abu-Dayyeh et al
EPI_ISL_430000	Biolab Diagnostic Laboratories	Andersen lab at Scripps Research	Issa Abu-Dayyeh et al
EPI_ISL_430008	Biolab Diagnostic Laboratories	Andersen lab at Scripps Research	Issa Abu-Dayyeh et al
EPI_ISL_430011	Biolab Diagnostic Laboratories	Andersen lab at Scripps Research	Issa Abu-Dayyeh et al
EPI_ISL_430015	Biolab Diagnostic Laboratories	Andersen lab at Scripps Research	Issa Abu-Dayyeh et al
EPI_ISL_480297	National Reference Laboratory Influenza and acute respiratory diseases	NRL-HIV	Ivan Ivanov et al
EPI_ISL_480298	National Reference Laboratory Influenza and acute respiratory diseases	NRL-HIV	Ivan Ivanov et al
EPI_ISL_480300	National Reference Laboratory Influenza and acute respiratory diseases	NRL-HIV	Ivan Ivanov et al
EPI_ISL_480302	National Reference Laboratory Influenza and acute respiratory diseases	NRL-HIV	Ivan Ivanov et al
EPI_ISL_480303	National Reference Laboratory Influenza and acute respiratory diseases	NRL-HIV	Ivan Ivanov et al
EPI_ISL_480305	National Reference Laboratory Influenza and acute respiratory diseases	NRL-HIV	Ivan Ivanov et al
EPI_ISL_480307	National Reference Laboratory Influenza and acute respiratory diseases	NRL-HIV	Ivan Ivanov et al
EPI_ISL_480308	National Reference Laboratory Influenza and acute respiratory diseases	NRL-HIV	Ivan Ivanov et al
EPI_ISL_480310	National Reference Laboratory Influenza and acute respiratory diseases	NRL-HIV	Ivan Ivanov et al
EPI_ISL_454581	University Hospital for Infectious Diseases Dr. Fran Mihaljević, Research Unit	University of Zagreb, Centre for research and knowledge transfer in biotechnology	Ivan-Christian Kurolt et al
EPI_ISL_454588	University Hospital for Infectious Diseases Dr. Fran Mihaljević, Research Unit	University of Zagreb, Centre for research and knowledge transfer in biotechnology	Ivan-Christian Kurolt et al
EPI_ISL_454592	University Hospital for Infectious Diseases Dr. Fran Mihaljević, Research Unit	University of Zagreb, Centre for research and knowledge transfer in biotechnology	Ivan-Christian Kurolt et al
EPI_ISL_450518	E. Gulbja Laboratorija	Latvian Biomedical Research and Study Centre	Ivars Silamiķelis et al
EPI_ISL_450519	E. Gulbja Laboratorija	Latvian Biomedical Research and Study Centre	Ivars Silamiķelis et al
EPI_ISL_421654	E. Gulbja Laboratorija	Latvian Biomedical Research and Study Centre	Ivars Silamiķelis et al
EPI_ISL_450522	Centrālā Laboratorija	Latvian Biomedical Research and Study Centre	Ivars Silamiķelis et al
EPI_ISL_450523	Centrālā Laboratorija	Latvian Biomedical Research and Study Centre	Ivars Silamiķelis et al
EPI_ISL_450524	Centrālā Laboratorija	Latvian Biomedical Research and Study Centre	Ivars Silamiķelis et al
EPI_ISL_486390	Centrālā laboratorija	Latvian Biomedical Research and Study Centre	Ivars Silamiķelis et al
EPI_ISL_486410	Centrala laboratorija	Latvian Biomedical Research and Study Centre	Ivars Silamiķelis et al

EPI_ISL_486412	Centrālā laboratorija	Latvian Biomedical Research and Study Centre	Ivars Silamiķelis et al
EPI_ISL_421655	E. Gulbja Laboratorija	Latvian Biomedical Research and Study Centre	Ivars Silamiķelis et al
EPI_ISL_486414	Centrālā laboratorija	Latvian Biomedical Research and Study Centre	Ivars Silamiķelis et al
EPI_ISL_486415	Centrālā laboratorija	Latvian Biomedical Research and Study Centre	Ivars Silamiķelis et al
EPI_ISL_486416	Centrālā laboratorija	Latvian Biomedical Research and Study Centre	Ivars Silamiķelis et al
EPI_ISL_421656	E. Gulbja Laboratorija	Latvian Biomedical Research and Study Centre	Ivars Silamiķelis et al
EPI_ISL_486423	Latvijas Infektoloģijas centrs	Latvian Biomedical Research and Study Centre	Ivars Silamiķelis et al
EPI_ISL_492988	Centrālā laboratorija	Latvian Biomedical Research and Study Centre	Ivars Silamiķelis et al
EPI_ISL_486426	Latvijas Infektoloģijas centrs	Latvian Biomedical Research and Study Centre	Ivars Silamiķelis et al
EPI_ISL_486430	Latvijas Infektoloģijas centrs	Latvian Biomedical Research and Study Centre	Ivars Silamiķelis et al
EPI_ISL_426285	E. Gulbja Laboratorija	Latvian Biomedical Research and Study Centre	Ivars Silamiķelis et al
EPI_ISL_501286	Centrālā laboratorija	Latvian Biomedical Research and Study Centre	Ivars Silamiķelis et al
EPI_ISL_501829	E. Gulbja Laboratorija	Latvian Biomedical Research and Study Centre	Ivars Silamiķelis et al
EPI_ISL_501849	E. Gulbja Laboratorija	Latvian Biomedical Research and Study Centre	Ivars Silamiķelis et al
EPI_ISL_501915	Centrālā laboratorija	Latvian Biomedical Research and Study Centre	Ivars Silamiķelis et al
EPI_ISL_501922	Centrālā laboratorija	Latvian Biomedical Research and Study Centre	Ivars Silamiķelis et al
EPI_ISL_512313	E. Gulbja Laboratorija	Latvian Biomedical Research and Study Centre	Ivars Silamiķelis et al
EPI_ISL_426289	E. Gulbja Laboratorija	Latvian Biomedical Research and Study Centre	Ivars Silamiķelis et al
EPI_ISL_512647	Latvijas Infektoloģijas centrs	Latvian Biomedical Research and Study Centre	Ivars Silamiķelis et al
EPI_ISL_512649	Latvijas Infektoloģijas centrs	Latvian Biomedical Research and Study Centre	Ivars Silamiķelis et al
EPI_ISL_515185	Latvijas Infektoloģijas centrs	Latvian Biomedical Research and Study Centre	Ivars Silamiķelis et al
EPI_ISL_515186	Latvijas Infektoloģijas centrs	Latvian Biomedical Research and Study Centre	Ivars Silamiķelis et al
EPI_ISL_639637	E. Gulbja Laboratorija	Latvian Biomedical Research and Study Centre	Ivars Silamiķelis et al
EPI_ISL_639658	E. Gulbja Laboratorija	Latvian Biomedical Research and Study Centre	Ivars Silamiķelis et al
EPI_ISL_856707	MFD Veselības centrs ''Dziedniecība''	Latvian Biomedical Research and Study Centre	Ivars Silamiķelis et al
EPI_ISL_856709	MFD Veselības centrs ''Dziedniecība''	Latvian Biomedical Research and Study Centre	Ivars Silamiķelis et al
EPI_ISL_1014678	National Influenza Center, Virology Department	National Influenza Center	J Yavarian et al
EPI_ISL_959282	National Influenza Center, Virology Department	National Influenza Center	J Yavarian et al
EPI_ISL_862080	National Influenza Center, Virology Department	National Influenza Center	J Yavarian et al
EPI_ISL_1014687	National Influenza Center, Virology Department	National Influenza Center	J Yavarian et al

EPI_ISL_862077	National Influenza Center, Virology Department	National Influenza Center	J Yavarian et al
EPI_ISL_1014683	National Influenza Center, Virology Department	National Influenza Center	J Yavarian et al
EPI_ISL_886066	Lighthouse Lab in Alderley Park	Wellcome Sanger Institute for the COVID-19 Genomics UK (COG-UK) Consortium	Jacquelyn Wynn et al
EPI_ISL_886148	Lighthouse Lab in Alderley Park	Wellcome Sanger Institute for the COVID-19 Genomics UK (COG-UK) Consortium	Jacquelyn Wynn et al
EPI_ISL_1584044	Lighthouse Lab in Alderley Park	Wellcome Sanger Institute for the COVID-19 Genomics UK (COG-UK) Consortium	Jacquelyn Wynn et al
EPI_ISL_567767	Lighthouse Lab in Alderley Park	Wellcome Sanger Institute for the COVID-19 Genomics UK (COG-UK) consortium	Jacquelyn Wynn et al
EPI_ISL_674292	Lighthouse Lab in Alderley Park	Wellcome Sanger Institute for the COVID-19 Genomics UK (COG-UK) Consortium	Jacquelyn Wynn et al
EPI_ISL_703236	Lighthouse Lab in Alderley Park	Wellcome Sanger Institute for the COVID-19 Genomics UK (COG-UK) Consortium	Jacquelyn Wynn et al
EPI_ISL_833971	Lighthouse Lab in Alderley Park	Wellcome Sanger Institute for the COVID-19 Genomics UK (COG-UK) Consortium	Jacquelyn Wynn et al
EPI_ISL_463741	Department of Molecular Virology, Cyprus Institute of Neurology and Genetics	Department of Molecular Virology, Cyprus Institute of Neurology and Genetics	Jan Richter et al
EPI_ISL_463742	Department of Molecular Virology, Cyprus Institute of Neurology and Genetics	Department of Molecular Virology, Cyprus Institute of Neurology and Genetics	Jan Richter et al
EPI_ISL_463743	Department of Molecular Virology, Cyprus Institute of Neurology and Genetics	Department of Molecular Virology, Cyprus Institute of Neurology and Genetics	Jan Richter et al
EPI_ISL_463744	Department of Molecular Virology, Cyprus Institute of Neurology and Genetics	Department of Molecular Virology, Cyprus Institute of Neurology and Genetics	Jan Richter et al
EPI_ISL_463745	Department of Molecular Virology, Cyprus Institute of Neurology and Genetics	Department of Molecular Virology, Cyprus Institute of Neurology and Genetics	Jan Richter et al
EPI_ISL_463747	Department of Molecular Virology, Cyprus Institute of Neurology and Genetics	Department of Molecular Virology, Cyprus Institute of Neurology and Genetics	Jan Richter et al
EPI_ISL_463748	Department of Molecular Virology, Cyprus Institute of Neurology and Genetics	Department of Molecular Virology, Cyprus Institute of Neurology and Genetics	Jan Richter et al
EPI_ISL_1164626	Department of Molecular Virology, Cyprus Institute of Neurology and Genetics	Department of Molecular Virology, Cyprus Institute of Neurology and Genetics	Jan Richter et al
EPI_ISL_1164627	Department of Molecular Virology, Cyprus Institute of Neurology and Genetics	Department of Molecular Virology, Cyprus Institute of Neurology and Genetics	Jan Richter et al
EPI_ISL_1164628	Department of Molecular Virology, Cyprus Institute of Neurology and Genetics	Department of Molecular Virology, Cyprus Institute of Neurology and Genetics	Jan Richter et al
EPI_ISL_1164629	Department of Molecular Virology, Cyprus Institute of Neurology and Genetics	Department of Molecular Virology, Cyprus Institute of Neurology and Genetics	Jan Richter et al
EPI_ISL_1164630	Department of Molecular Virology, Cyprus Institute of Neurology and Genetics	Department of Molecular Virology, Cyprus Institute of Neurology and Genetics	Jan Richter et al
EPI_ISL_1164631	Department of Molecular Virology, Cyprus Institute of Neurology and Genetics	Department of Molecular Virology, Cyprus Institute of Neurology and Genetics	Jan Richter et al

EPI_ISL_1164632	Department of Molecular Virology, Cyprus Institute of Neurology and Genetics	Department of Molecular Virology, Cyprus Institute of Neurology and Genetics	Jan Richter et al
EPI_ISL_1164633	Department of Molecular Virology, Cyprus Institute of Neurology and Genetics	Department of Molecular Virology, Cyprus Institute of Neurology and Genetics	Jan Richter et al
EPI_ISL_1164634	Department of Molecular Virology, Cyprus Institute of Neurology and Genetics	Department of Molecular Virology, Cyprus Institute of Neurology and Genetics	Jan Richter et al
EPI_ISL_1164635	Department of Molecular Virology, Cyprus Institute of Neurology and Genetics	Department of Molecular Virology, Cyprus Institute of Neurology and Genetics	Jan Richter et al
EPI_ISL_1164636	Department of Molecular Virology, Cyprus Institute of Neurology and Genetics	Department of Molecular Virology, Cyprus Institute of Neurology and Genetics	Jan Richter et al
EPI_ISL_1164637	Department of Molecular Virology, Cyprus Institute of Neurology and Genetics	Department of Molecular Virology, Cyprus Institute of Neurology and Genetics	Jan Richter et al
EPI_ISL_1164641	Department of Molecular Virology, Cyprus Institute of Neurology and Genetics	Department of Molecular Virology, Cyprus Institute of Neurology and Genetics	Jan Richter et al
EPI_ISL_1164642	Department of Molecular Virology, Cyprus Institute of Neurology and Genetics	Department of Molecular Virology, Cyprus Institute of Neurology and Genetics	Jan Richter et al
EPI_ISL_1164643	Department of Molecular Virology, Cyprus Institute of Neurology and Genetics	Department of Molecular Virology, Cyprus Institute of Neurology and Genetics	Jan Richter et al
EPI_ISL_1164646	Department of Molecular Virology, Cyprus Institute of Neurology and Genetics	Department of Molecular Virology, Cyprus Institute of Neurology and Genetics	Jan Richter et al
EPI_ISL_1164647	Department of Molecular Virology, Cyprus Institute of Neurology and Genetics	Department of Molecular Virology, Cyprus Institute of Neurology and Genetics	Jan Richter et al
EPI_ISL_1164648	Department of Molecular Virology, Cyprus Institute of Neurology and Genetics	Department of Molecular Virology, Cyprus Institute of Neurology and Genetics	Jan Richter et al
EPI_ISL_1164651	Department of Molecular Virology, Cyprus Institute of Neurology and Genetics	Department of Molecular Virology, Cyprus Institute of Neurology and Genetics	Jan Richter et al
EPI_ISL_1164652	Department of Molecular Virology, Cyprus Institute of Neurology and Genetics	Department of Molecular Virology, Cyprus Institute of Neurology and Genetics	Jan Richter et al
EPI_ISL_1164654	Department of Molecular Virology, Cyprus Institute of Neurology and Genetics	Department of Molecular Virology, Cyprus Institute of Neurology and Genetics	Jan Richter et al
EPI_ISL_1164658	Department of Molecular Virology, Cyprus Institute of Neurology and Genetics	Department of Molecular Virology, Cyprus Institute of Neurology and Genetics	Jan Richter et al
EPI_ISL_1164675	Department of Molecular Virology, Cyprus Institute of Neurology and Genetics	Department of Molecular Virology, Cyprus Institute of Neurology and Genetics	Jan Richter et al
EPI_ISL_1164678	Department of Molecular Virology, Cyprus Institute of Neurology and Genetics	Department of Molecular Virology, Cyprus Institute of Neurology and Genetics	Jan Richter et al
EPI_ISL_1164685	Department of Molecular Virology, Cyprus Institute of Neurology and Genetics	Department of Molecular Virology, Cyprus Institute of Neurology and Genetics	Jan Richter et al
EPI_ISL_1164687	Department of Molecular Virology, Cyprus Institute of Neurology and Genetics	Department of Molecular Virology, Cyprus Institute of Neurology and Genetics	Jan Richter et al
EPI_ISL_1164689	Department of Molecular Virology, Cyprus Institute of Neurology and Genetics	Department of Molecular Virology, Cyprus Institute of Neurology and Genetics	Jan Richter et al
EPI_ISL_1164690	Department of Molecular Virology, Cyprus Institute of Neurology and Genetics	Department of Molecular Virology, Cyprus Institute of Neurology and Genetics	Jan Richter et al

EPI_ISL_1164691	Department of Molecular Virology, Cyprus Institute of Neurology and Genetics	Department of Molecular Virology, Cyprus Institute of Neurology and Genetics	Jan Richter et al
EPI_ISL_1164694	Department of Molecular Virology, Cyprus Institute of Neurology and Genetics	Department of Molecular Virology, Cyprus Institute of Neurology and Genetics	Jan Richter et al
EPI_ISL_1164695	Department of Molecular Virology, Cyprus Institute of Neurology and Genetics	Department of Molecular Virology, Cyprus Institute of Neurology and Genetics	Jan Richter et al
EPI_ISL_1164696	Department of Molecular Virology, Cyprus Institute of Neurology and Genetics	Department of Molecular Virology, Cyprus Institute of Neurology and Genetics	Jan Richter et al
EPI_ISL_1164698	Department of Molecular Virology, Cyprus Institute of Neurology and Genetics	Department of Molecular Virology, Cyprus Institute of Neurology and Genetics	Jan Richter et al
EPI_ISL_1164708	Department of Molecular Virology, Cyprus Institute of Neurology and Genetics	Department of Molecular Virology, Cyprus Institute of Neurology and Genetics	Jan Richter et al
EPI_ISL_1164711	Department of Molecular Virology, Cyprus Institute of Neurology and Genetics	Department of Molecular Virology, Cyprus Institute of Neurology and Genetics	Jan Richter et al
EPI_ISL_1164712	Department of Molecular Virology, Cyprus Institute of Neurology and Genetics	Department of Molecular Virology, Cyprus Institute of Neurology and Genetics	Jan Richter et al
EPI_ISL_1164715	Department of Molecular Virology, Cyprus Institute of Neurology and Genetics	Department of Molecular Virology, Cyprus Institute of Neurology and Genetics	Jan Richter et al
EPI_ISL_1164731	Department of Molecular Virology, Cyprus Institute of Neurology and Genetics	Department of Molecular Virology, Cyprus Institute of Neurology and Genetics	Jan Richter et al
EPI_ISL_1164744	Department of Molecular Virology, Cyprus Institute of Neurology and Genetics	Department of Molecular Virology, Cyprus Institute of Neurology and Genetics	Jan Richter et al
EPI_ISL_1164746	Department of Molecular Virology, Cyprus Institute of Neurology and Genetics	Department of Molecular Virology, Cyprus Institute of Neurology and Genetics	Jan Richter et al
EPI_ISL_483150	Robert Koch Institute, ZBS1 Highly Pathogenic Viruses, Berlin, Germany	Robert Koch Institute, Bioinformatics MF1, Berlin, Germany	Janine Michel et al
EPI_ISL_1312555	Centrala laboratorija	Latvian Biomedical Research and Study Centre	Janis Pjalkovskis et al
EPI_ISL_1312639	E. Gulbja laboratorija	Latvian Biomedical Research and Study Centre	Janis Pjalkovskis et al
EPI_ISL_1312655	E. Gulbja laboratorija	Latvian Biomedical Research and Study Centre	Janis Pjalkovskis et al
EPI_ISL_1312683	E. Gulbja laboratorija	Latvian Biomedical Research and Study Centre	Janis Pjalkovskis et al
EPI_ISL_1312689	E. Gulbja laboratorija	Latvian Biomedical Research and Study Centre	Janis Pjalkovskis et al
EPI_ISL_1312690	E. Gulbja laboratorija	Latvian Biomedical Research and Study Centre	Janis Pjalkovskis et al
EPI_ISL_1312724	E. Gulbja laboratorija	Latvian Biomedical Research and Study Centre	Janis Pjalkovskis et al
EPI_ISL_1321930	E. Gulbja laboratorija	Latvian Biomedical Research and Study Centre	Janis Pjalkovskis et al
EPI_ISL_1613124	LIC	Latvian Biomedical Research and Study Centre	Janis Pjalkovskis et al
EPI_ISL_1613125	LIC	Latvian Biomedical Research and Study Centre	Janis Pjalkovskis et al
EPI_ISL_1613127	LIC	Latvian Biomedical Research and Study Centre	Janis Pjalkovskis et al
EPI_ISL_1613776	E. Gulbja laboratorija	Latvian Biomedical Research and Study Centre	Janis Pjalkovskis et al
EPI_ISL_961801	E. Gulbja laboratorija	Latvian Biomedical Research and Study Centre	Janis Pjalkovskis et al

EPI_ISL_1219217	E. Gulbja laboratorija	Latvian Biomedical Research and Study Centre	Janis Pjalkovskis et al
EPI_ISL_1219423	BIOR	Latvian Biomedical Research and Study Centre	Janis Pjalkovskis et al
EPI_ISL_1219558	E. Gulbja laboratorija	Latvian Biomedical Research and Study Centre	Janis Pjalkovskis et al
EPI_ISL_1219603	E. Gulbja laboratorija	Latvian Biomedical Research and Study Centre	Janis Pjalkovskis et al
EPI_ISL_1219663	E. Gulbja laboratorija	Latvian Biomedical Research and Study Centre	Janis Pjalkovskis et al
EPI_ISL_1312403	E. Gulbja laboratorija	Latvian Biomedical Research and Study Centre	Janis Pjalkovskis et al
EPI_ISL_1312417	E. Gulbja laboratorija	Latvian Biomedical Research and Study Centre	Janis Pjalkovskis et al
EPI_ISL_1312455	E. Gulbja laboratorija	Latvian Biomedical Research and Study Centre	Janis Pjalkovskis et al
EPI_ISL_1312476	E. Gulbja laboratorija	Latvian Biomedical Research and Study Centre	Janis Pjalkovskis et al
EPI_ISL_1312496	E. Gulbja laboratorija	Latvian Biomedical Research and Study Centre	Janis Pjalkovskis et al
EPI_ISL_1312499	LIC	Latvian Biomedical Research and Study Centre	Janis Pjalkovskis et al
EPI_ISL_1544184	Government Medical College (GMC), Surat	Gujarat Biotechnology Research Centre	Janvi Raval et al
EPI_ISL_412964	Hospital Israelita Albert Einstein	Instituto Adolfo Lutz Interdisciplinary Procedures Center Strategic Laboratory	Jaqueline Goes de Jesus et al
EPI_ISL_413016	Hospital Israelita Albert Einstein	Instituto Adolfo Lutz, Interdisciplinary Procedures Center, Strategic Laboratory	Jaqueline Goes de Jesus et al
EPI_ISL_994658	Dr. Andrija Stampar“ Teaching Institute of Public Health, Department of Clinical Microbiology	Institute of Applied Genomics	Jasmina Vranes et al
EPI_ISL_994659	Dr. Andrija Stampar“ Teaching Institute of Public Health, Department of Clinical Microbiology	Institute of Applied Genomics	Jasmina Vranes et al
EPI_ISL_994667	Dr. Andrija Stampar“ Teaching Institute of Public Health, Department of Clinical Microbiology	Institute of Applied Genomics	Jasmina Vranes et al
EPI_ISL_994661	Dr. Andrija Stampar“ Teaching Institute of Public Health, Department of Clinical Microbiology	Institute of Applied Genomics	Jasmina Vranes et al
EPI_ISL_994740	Dr. Andrija Stampar“ Teaching Institute of Public Health, Department of Clinical Microbiology	Institute of Applied Genomics	Jasmina Vranes et al
EPI_ISL_996004	Dr. Andrija Stampar“ Teaching Institute of Public Health, Department of Clinical Microbiology	Institute of Applied Genomics	Jasmina Vranes et al
EPI_ISL_994739	Dr. Andrija Stampar“ Teaching Institute of Public Health, Department of Clinical Microbiology	Institute of Applied Genomics	Jasmina Vranes et al
EPI_ISL_1167040	Dr. Andrija Stampar Teaching Institute of Public Health, Department of Clinical Microbiology	Istituto di Genomica Applicata	Jasmina Vranes et al
EPI_ISL_1167044	Dr. Andrija Stampar Teaching Institute of Public Health, Department of Clinical Microbiology	Istituto di Genomica Applicata	Jasmina Vranes et al
EPI_ISL_1167045	Dr. Andrija Stampar Teaching Institute of Public Health, Department of Clinical Microbiology	Istituto di Genomica Applicata	Jasmina Vranes et al
EPI_ISL_1167046	Dr. Andrija Stampar Teaching Institute of Public Health, Department of Clinical Microbiology	Istituto di Genomica Applicata	Jasmina Vranes et al

EPI_ISL_1167049	Dr. Andrija Stampar Teaching Institute of Public Health, Department of Clinical Microbiology	Istituto di Genomica Applicata	Jasmina Vranes et al
EPI_ISL_1167053	Dr. Andrija Stampar Teaching Institute of Public Health, Department of Clinical Microbiology	Istituto di Genomica Applicata	Jasmina Vranes et al
EPI_ISL_1167054	Dr. Andrija Stampar Teaching Institute of Public Health, Department of Clinical Microbiology	Istituto di Genomica Applicata	Jasmina Vranes et al
EPI_ISL_1167057	Dr. Andrija Stampar Teaching Institute of Public Health, Department of Clinical Microbiology	Istituto di Genomica Applicata	Jasmina Vranes et al
EPI_ISL_1167067	Dr. Andrija Stampar Teaching Institute of Public Health, Department of Clinical Microbiology	Istituto di Genomica Applicata	Jasmina Vranes et al
EPI_ISL_1167082	Dr. Andrija Stampar Teaching Institute of Public Health, Department of Clinical Microbiology	Istituto di Genomica Applicata	Jasmina Vranes et al
EPI_ISL_1167083	Dr. Andrija Stampar Teaching Institute of Public Health, Department of Clinical Microbiology	Istituto di Genomica Applicata	Jasmina Vranes et al
EPI_ISL_1167084	Dr. Andrija Stampar Teaching Institute of Public Health, Department of Clinical Microbiology	Istituto di Genomica Applicata	Jasmina Vranes et al
EPI_ISL_1167085	Dr. Andrija Stampar Teaching Institute of Public Health, Department of Clinical Microbiology	Istituto di Genomica Applicata	Jasmina Vranes et al
EPI_ISL_1167028	Dr. Andrija Stampar Teaching Institute of Public Health, Department of Clinical Microbiology	Istituto di Genomica Applicata	Jasmina Vranes et al
EPI_ISL_1661222	Platform BIS UZA/UAntwerpen	Labo Klinische Biologie, UZA	Jasmine Coppens et al
EPI_ISL_1661211	Platform BIS UZA/UAntwerpen	Labo Klinische Biologie, UZA	Jasmine Coppens et al
EPI_ISL_417444	Department of Healthcare Biotechnology, National University of Sciences and Technology (NUST)	Department of Healthcare Biotechnology, National University of Sciences and Technology (NUST)	Javed et al
EPI_ISL_1300508	Genetica Molecular and Subdepartamento de Virologia ISP Chile	Instituto de Salud Publica de Chile	Javier Tognarelli et al
EPI_ISL_1167794	Genetica Molecular and Subdepartamento de Virologia ISP Chile	Instituto de Salud Publica de Chile	Javier Tognarelli et al
EPI_ISL_1541058	Genetica Molecular and Subdepartamento de Virologia ISP Chile	Instituto de Salud Publica de Chile	Javier Tognarelli et al
EPI_ISL_746499	Genetica Molecular and Subdepartamento de Virologia ISP Chile	Instituto de Salud Publica de Chile	Javier Tognarelli et al
EPI_ISL_746503	Genetica Molecular and Subdepartamento de Virologia ISP Chile	Instituto de Salud Publica de Chile	Javier Tognarelli et al
EPI_ISL_1167844	Genetica Molecular and Subdepartamento de Virologia ISP Chile	Instituto de Salud Publica de Chile	Javier Tognarelli et al
EPI_ISL_1167835	Genetica Molecular and Subdepartamento de Virologia ISP Chile	Instituto de Salud Publica de Chile	Javier Tognarelli et al
EPI_ISL_1300511	Genetica Molecular and Subdepartamento de Virologia ISP Chile	Instituto de Salud Publica de Chile	Javier Tognarelli et al
EPI_ISL_1321495	Genetica Molecular and Subdepartamento de Virologia ISP Chile	Instituto de Salud Publica de Chile	Javier Tognarelli et al
EPI_ISL_1321498	Genetica Molecular and Subdepartamento de Virologia ISP Chile	Instituto de Salud Publica de Chile	Javier Tognarelli et al
EPI_ISL_1167875	Genetica Molecular and Subdepartamento de Virologia ISP Chile	Instituto de Salud Publica de Chile	Javier Tognarelli et al
EPI_ISL_1167871	Genetica Molecular and Subdepartamento de Virologia ISP Chile	Instituto de Salud Publica de Chile	Javier Tognarelli et al
EPI_ISL_1633549	Genetica Molecular and Subdepartamento de Virologia ISP Chile	Instituto de Salud Publica de Chile	Javier Tognarelli et al

EPI_ISL_1167804	Genetica Molecular and Subdepartamento de Virologia ISP Chile	Instituto de Salud Publica de Chile	Javier Tognarelli et al
EPI_ISL_746509	Genetica Molecular and Subdepartamento de Virologia ISP Chile	Instituto de Salud Publica de Chile	Javier Tognarelli et al
EPI_ISL_746518	Genetica Molecular and Subdepartamento de Virologia ISP Chile	Instituto de Salud Publica de Chile	Javier Tognarelli et al
EPI_ISL_1167810	Genetica Molecular and Subdepartamento de Virologia ISP Chile	Instituto de Salud Publica de Chile	Javier Tognarelli et al
EPI_ISL_1300502	Genetica Molecular and Subdepartamento de Virologia ISP Chile	Instituto de Salud Publica de Chile	Javier Tognarelli et al
EPI_ISL_1470478	Genetica Molecular and Subdepartamento de Virologia ISP Chile	Instituto de Salud Publica de Chile	Javier Tognarelli et al
EPI_ISL_1167907	Genetica Molecular and Subdepartamento de Virologia ISP Chile	Instituto de Salud Publica de Chile	Javier Tognarelli et al
EPI_ISL_1321578	Genetica Molecular and Subdepartamento de Virologia ISP Chile	Instituto de Salud Publica de Chile	Javier Tognarelli et al
EPI_ISL_1321468	Genetica Molecular and Subdepartamento de Virologia ISP Chile	Instituto de Salud Publica de Chile	Javier Tognarelli et al
EPI_ISL_1167796	Genetica Molecular and Subdepartamento de Virologia ISP Chile	Instituto de Salud Publica de Chile	Javier Tognarelli et al
EPI_ISL_1167861	Genetica Molecular and Subdepartamento de Virologia ISP Chile	Instituto de Salud Publica de Chile	Javier Tognarelli et al
EPI_ISL_1321469	Genetica Molecular and Subdepartamento de Virologia ISP Chile	Instituto de Salud Publica de Chile	Javier Tognarelli et al
EPI_ISL_1321505	Genetica Molecular and Subdepartamento de Virologia ISP Chile	Instituto de Salud Publica de Chile	Javier Tognarelli et al
EPI_ISL_1321470	Genetica Molecular and Subdepartamento de Virologia ISP Chile	Instituto de Salud Publica de Chile	Javier Tognarelli et al
EPI_ISL_1470531	Genetica Molecular and Subdepartamento de Virologia ISP Chile	Instituto de Salud Publica de Chile	Javier Tognarelli et al
EPI_ISL_1541032	Genetica Molecular and Subdepartamento de Virologia ISP Chile	Instituto de Salud Publica de Chile	Javier Tognarelli et al
EPI_ISL_1541033	Genetica Molecular and Subdepartamento de Virologia ISP Chile	Instituto de Salud Publica de Chile	Javier Tognarelli et al
EPI_ISL_1167918	Genetica Molecular and Subdepartamento de Virologia ISP Chile	Instituto de Salud Publica de Chile	Javier Tognarelli et al
EPI_ISL_1167668	Genetica Molecular and Subdepartamento de Virologia ISP Chile	Instituto de Salud Publica de Chile	Javier Tognarelli et al
EPI_ISL_746545	Genetica Molecular and Subdepartamento de Virologia ISP Chile	Instituto de Salud Publica de Chile	Javier Tognarelli et al
EPI_ISL_746559	Genetica Molecular and Subdepartamento de Virologia ISP Chile	Instituto de Salud Publica de Chile	Javier Tognarelli et al
EPI_ISL_1167842	Genetica Molecular and Subdepartamento de Virologia ISP Chile	Instituto de Salud Publica de Chile	Javier Tognarelli et al
EPI_ISL_746570	Genetica Molecular and Subdepartamento de Virologia ISP Chile	Instituto de Salud Publica de Chile	Javier Tognarelli et al
EPI_ISL_746589	Genetica Molecular and Subdepartamento de Virologia ISP Chile	Instituto de Salud Publica de Chile	Javier Tognarelli et al
EPI_ISL_746590	Genetica Molecular and Subdepartamento de Virologia ISP Chile	Instituto de Salud Publica de Chile	Javier Tognarelli et al
EPI_ISL_746622	Genetica Molecular and Subdepartamento de Virologia ISP Chile	Instituto de Salud Publica de Chile	Javier Tognarelli et al
EPI_ISL_746624	Genetica Molecular and Subdepartamento de Virologia ISP Chile	Instituto de Salud Publica de Chile	Javier Tognarelli et al
EPI_ISL_746628	Genetica Molecular and Subdepartamento de Virologia ISP Chile	Instituto de Salud Publica de Chile	Javier Tognarelli et al
EPI_ISL_746635	Genetica Molecular and Subdepartamento de Virologia ISP Chile	Instituto de Salud Publica de Chile	Javier Tognarelli et al
EPI_ISL_746640	Genetica Molecular and Subdepartamento de Virologia ISP Chile	Instituto de Salud Publica de Chile	Javier Tognarelli et al

EPI_ISL_746644	Genetica Molecular and Subdepartamento de Virologia ISP Chile	Instituto de Salud Publica de Chile	Javier Tognarelli et al
EPI_ISL_746660	Genetica Molecular and Subdepartamento de Virologia ISP Chile	Instituto de Salud Publica de Chile	Javier Tognarelli et al
EPI_ISL_746666	Genetica Molecular and Subdepartamento de Virologia ISP Chile	Instituto de Salud Publica de Chile	Javier Tognarelli et al
EPI_ISL_746673	Genetica Molecular and Subdepartamento de Virologia ISP Chile	Instituto de Salud Publica de Chile	Javier Tognarelli et al
EPI_ISL_746674	Genetica Molecular and Subdepartamento de Virologia ISP Chile	Instituto de Salud Publica de Chile	Javier Tognarelli et al
EPI_ISL_746675	Genetica Molecular and Subdepartamento de Virologia ISP Chile	Instituto de Salud Publica de Chile	Javier Tognarelli et al
EPI_ISL_746682	Genetica Molecular and Subdepartamento de Virologia ISP Chile	Instituto de Salud Publica de Chile	Javier Tognarelli et al
EPI_ISL_746685	Genetica Molecular and Subdepartamento de Virologia ISP Chile	Instituto de Salud Publica de Chile	Javier Tognarelli et al
EPI_ISL_746686	Genetica Molecular and Subdepartamento de Virologia ISP Chile	Instituto de Salud Publica de Chile	Javier Tognarelli et al
EPI_ISL_746687	Genetica Molecular and Subdepartamento de Virologia ISP Chile	Instituto de Salud Publica de Chile	Javier Tognarelli et al
EPI_ISL_746706	Genetica Molecular and Subdepartamento de Virologia ISP Chile	Instituto de Salud Publica de Chile	Javier Tognarelli et al
EPI_ISL_746723	Genetica Molecular and Subdepartamento de Virologia ISP Chile	Instituto de Salud Publica de Chile	Javier Tognarelli et al
EPI_ISL_746736	Genetica Molecular and Subdepartamento de Virologia ISP Chile	Instituto de Salud Publica de Chile	Javier Tognarelli et al
EPI_ISL_746743	Genetica Molecular and Subdepartamento de Virologia ISP Chile	Instituto de Salud Publica de Chile	Javier Tognarelli et al
EPI_ISL_746750	Genetica Molecular and Subdepartamento de Virologia ISP Chile	Instituto de Salud Publica de Chile	Javier Tognarelli et al
EPI_ISL_746754	Genetica Molecular and Subdepartamento de Virologia ISP Chile	Instituto de Salud Publica de Chile	Javier Tognarelli et al
EPI_ISL_1300459	Genetica Molecular and Subdepartamento de Virologia ISP Chile	Instituto de Salud Publica de Chile	Javier Tognarelli et al
EPI_ISL_746759	Genetica Molecular and Subdepartamento de Virologia ISP Chile	Instituto de Salud Publica de Chile	Javier Tognarelli et al
EPI_ISL_746761	Genetica Molecular and Subdepartamento de Virologia ISP Chile	Instituto de Salud Publica de Chile	Javier Tognarelli et al
EPI_ISL_746767	Genetica Molecular and Subdepartamento de Virologia ISP Chile	Instituto de Salud Publica de Chile	Javier Tognarelli et al
EPI_ISL_1167696	Genetica Molecular and Subdepartamento de Virologia ISP Chile	Instituto de Salud Publica de Chile	Javier Tognarelli et al
EPI_ISL_1167705	Genetica Molecular and Subdepartamento de Virologia ISP Chile	Instituto de Salud Publica de Chile	Javier Tognarelli et al
EPI_ISL_1167707	Genetica Molecular and Subdepartamento de Virologia ISP Chile	Instituto de Salud Publica de Chile	Javier Tognarelli et al
EPI_ISL_1167710	Genetica Molecular and Subdepartamento de Virologia ISP Chile	Instituto de Salud Publica de Chile	Javier Tognarelli et al
EPI_ISL_1167711	Genetica Molecular and Subdepartamento de Virologia ISP Chile	Instituto de Salud Publica de Chile	Javier Tognarelli et al
EPI_ISL_1167716	Genetica Molecular and Subdepartamento de Virologia ISP Chile	Instituto de Salud Publica de Chile	Javier Tognarelli et al
EPI_ISL_1167722	Genetica Molecular and Subdepartamento de Virologia ISP Chile	Instituto de Salud Publica de Chile	Javier Tognarelli et al
EPI_ISL_1167724	Genetica Molecular and Subdepartamento de Virologia ISP Chile	Instituto de Salud Publica de Chile	Javier Tognarelli et al
EPI_ISL_1167736	Genetica Molecular and Subdepartamento de Virologia ISP Chile	Instituto de Salud Publica de Chile	Javier Tognarelli et al
EPI_ISL_1167847	Genetica Molecular and Subdepartamento de Virologia ISP Chile	Instituto de Salud Publica de Chile	Javier Tognarelli et al

EPI_ISL_1167877	Genetica Molecular and Subdepartamento de Virologia ISP Chile	Instituto de Salud Publica de Chile	Javier Tognarelli et al
EPI_ISL_1167883	Genetica Molecular and Subdepartamento de Virologia ISP Chile	Instituto de Salud Publica de Chile	Javier Tognarelli et al
EPI_ISL_1321466	Genetica Molecular and Subdepartamento de Virologia ISP Chile	Instituto de Salud Publica de Chile	Javier Tognarelli et al
EPI_ISL_1321560	Genetica Molecular and Subdepartamento de Virologia ISP Chile	Instituto de Salud Publica de Chile	Javier Tognarelli et al
EPI_ISL_1321512	Genetica Molecular and Subdepartamento de Virologia ISP Chile	Instituto de Salud Publica de Chile	Javier Tognarelli et al
EPI_ISL_1321515	Genetica Molecular and Subdepartamento de Virologia ISP Chile	Instituto de Salud Publica de Chile	Javier Tognarelli et al
EPI_ISL_1167815	Genetica Molecular and Subdepartamento de Virologia ISP Chile	Instituto de Salud Publica de Chile	Javier Tognarelli et al
EPI_ISL_1470501	Genetica Molecular and Subdepartamento de Virologia ISP Chile	Instituto de Salud Publica de Chile	Javier Tognarelli et al
EPI_ISL_1470440	Genetica Molecular and Subdepartamento de Virologia ISP Chile	Instituto de Salud Publica de Chile	Javier Tognarelli et al
EPI_ISL_1470457	Genetica Molecular and Subdepartamento de Virologia ISP Chile	Instituto de Salud Publica de Chile	Javier Tognarelli et al
EPI_ISL_1534590	Genetica Molecular and Subdepartamento de Virologia ISP Chile	Instituto de Salud Publica de Chile	Javier Tognarelli et al
EPI_ISL_1633493	Genetica Molecular and Subdepartamento de Virologia ISP Chile	Instituto de Salud Publica de Chile	Javier Tognarelli et al
EPI_ISL_1541038	Genetica Molecular and Subdepartamento de Virologia ISP Chile	Instituto de Salud Publica de Chile	Javier Tognarelli et al
EPI_ISL_1167771	Genetica Molecular and Subdepartamento de Virologia ISP Chile	Instituto de Salud Publica de Chile	Javier Tognarelli et al
EPI_ISL_1541064	Genetica Molecular and Subdepartamento de Virologia ISP Chile	Instituto de Salud Publica de Chile	Javier Tognarelli et al
EPI_ISL_1633360	Genetica Molecular and Subdepartamento de Virologia ISP Chile	Instituto de Salud Publica de Chile	Javier Tognarelli et al
EPI_ISL_1633362	Genetica Molecular and Subdepartamento de Virologia ISP Chile	Instituto de Salud Publica de Chile	Javier Tognarelli et al
EPI_ISL_1167772	Genetica Molecular and Subdepartamento de Virologia ISP Chile	Instituto de Salud Publica de Chile	Javier Tognarelli et al
EPI_ISL_746807	Genetica Molecular and Subdepartamento de Virologia ISP Chile	Instituto de Salud Publica de Chile	Javier Tognarelli et al
EPI_ISL_746484	Genetica Molecular and Subdepartamento de Virologia ISP Chile	Instituto de Salud Publica de Chile	Javier Tognarelli et al
EPI_ISL_1300499	Genetica Molecular and Subdepartamento de Virologia ISP Chile	Instituto de Salud Publica de Chile	Javier Tognarelli et al
EPI_ISL_1321471	Genetica Molecular and Subdepartamento de Virologia ISP Chile	Instituto de Salud Publica de Chile	Javier Tognarelli et al
EPI_ISL_1534607	Genetica Molecular and Subdepartamento de Virologia ISP Chile	Instituto de Salud Publica de Chile	Javier Tognarelli et al
EPI_ISL_826830	INSPI-CRN DE INFLUENZA Y OTROS VIRUS RESPIRATORIOS	Instituto de Salud Publica de Chile	Javier Tognarelli et al
EPI_ISL_826832	INSPI-CRN DE INFLUENZA Y OTROS VIRUS RESPIRATORIOS	Instituto de Salud Publica de Chile	Javier Tognarelli et al
EPI_ISL_826834	INSPI-CRN DE INFLUENZA Y OTROS VIRUS RESPIRATORIOS	Instituto de Salud Publica de Chile	Javier Tognarelli et al
EPI_ISL_826800	INSPI-CRN DE INFLUENZA Y OTROS VIRUS RESPIRATORIOS	Instituto de Salud Publica de Chile	Javier Tognarelli et al
EPI_ISL_826818	INSPI-CRN DE INFLUENZA Y OTROS VIRUS RESPIRATORIOS	Instituto de Salud Publica de Chile	Javier Tognarelli et al
EPI_ISL_826836	INSPI-CRN DE INFLUENZA Y OTROS VIRUS RESPIRATORIOS	Instituto de Salud Publica de Chile	Javier Tognarelli et al
EPI_ISL_831020	INSPI-CRN DE INFLUENZA Y OTROS VIRUS RESPIRATORIOS	Instituto de Salud Publica de Chile	Javier Tognarelli et al

EPI_ISL_826820	INSPI-CRN DE INFLUENZA Y OTROS VIRUS RESPIRATORIOS	Instituto de Salud Publica de Chile	Javier Tognarelli et al
EPI_ISL_826822	INSPI-CRN DE INFLUENZA Y OTROS VIRUS RESPIRATORIOS	Instituto de Salud Publica de Chile	Javier Tognarelli et al
EPI_ISL_826825	INSPI-CRN DE INFLUENZA Y OTROS VIRUS RESPIRATORIOS	Instituto de Salud Publica de Chile	Javier Tognarelli et al
EPI_ISL_636987	Virology Lab, National Institute for Biomedical Research (INRB)	Project group Epidemology of Highly Pathogenic Microorganisms, Robert Koch-Institute	Jean-Jacques Muyembe Tamfum et al
EPI_ISL_1014102	Zurita & Zurita Laboratorios	Zurita & Zurita Laboratorios	Jeannete Zurita et al
EPI_ISL_1040028	Original detection - Virology Unit, Institut Pasteur du Cambodge; Sequencing - US National Institute of Allergy and Infectious Diseases Cambodia	Virology Unit, Institut Pasteur du Cambodge	Jennifer Bohl et al
EPI_ISL_412869	Division of Viral Diseases, Center for Laboratory Control of Infectious Diseases, Korea Centers for Diseases Control and Prevention	Division of Viral Diseases, Center for Laboratory Control of Infectious Diseases, Korea Centers for Diseases Control and Prevention	Jeong-Min Kim et al
EPI_ISL_412871	Division of Viral Diseases, Center for Laboratory Control of Infectious Diseases, Korea Centers for Diseases Control and Prevention	Division of Viral Diseases, Center for Laboratory Control of Infectious Diseases, Korea Centers for Diseases Control and Prevention	Jeong-Min Kim et al
EPI_ISL_425118	Division of Viral Diseases, Center for Laboratory Control of Infectious Diseases, Korea Centers for Diseases Control and Prevention	Division of Viral Diseases, Center for Laboratory Control of Infectious Diseases, Korea Centers for Diseases Control and Prevention	Jeong-Min Kim et al
EPI_ISL_426163	Division of Viral Diseases, Center for Laboratory Control of Infectious Diseases, Korea Centers for Diseases Control and Prevention	Division of Viral Diseases, Center for Laboratory Control of Infectious Diseases, Korea Centers for Diseases Control and Prevention	Jeong-Min Kim et al
EPI_ISL_426187	Division of Viral Diseases, Center for Laboratory Control of Infectious Diseases, Korea Centers for Diseases Control and Prevention	Division of Viral Diseases, Center for Laboratory Control of Infectious Diseases, Korea Centers for Diseases Control and Prevention	Jeong-Min Kim et al
EPI_ISL_497962	Division of Viral Diseases, Center for Laboratory Control of Infectious Diseases, Korea Centers for Diseases Control and Prevention	Division of Viral Diseases, Center for Laboratory Control of Infectious Diseases, Korea Centers for Diseases Control and Prevention	Jeong-Min Kim et al
EPI_ISL_497966	Division of Viral Diseases, Center for Laboratory Control of Infectious Diseases, Korea Centers for Diseases Control and Prevention	Division of Viral Diseases, Center for Laboratory Control of Infectious Diseases, Korea Centers for Diseases Control and Prevention	Jeong-Min Kim et al
EPI_ISL_497970	Division of Viral Diseases, Center for Laboratory Control of Infectious Diseases, Korea Centers for Diseases Control and Prevention	Division of Viral Diseases, Center for Laboratory Control of Infectious Diseases, Korea Centers for Diseases Control and Prevention	Jeong-Min Kim et al
EPI_ISL_497974	Division of Viral Diseases, Center for Laboratory Control of Infectious Diseases, Korea Centers for Diseases Control and Prevention	Division of Viral Diseases, Center for Laboratory Control of Infectious Diseases, Korea Centers for Diseases Control and Prevention	Jeong-Min Kim et al
EPI_ISL_498037	Division of Viral Diseases, Center for Laboratory Control of Infectious Diseases, Korea Centers for Diseases Control and Prevention	Division of Viral Diseases, Center for Laboratory Control of Infectious Diseases, Korea Centers for Diseases Control and Prevention	Jeong-Min Kim et al
EPI_ISL_498049	Division of Viral Diseases, Center for Laboratory Control of Infectious Diseases, Korea Centers for Diseases Control and Prevention	Division of Viral Diseases, Center for Laboratory Control of Infectious Diseases, Korea Centers for Diseases Control and Prevention	Jeong-Min Kim et al
EPI_ISL_506982	Division of Viral Diseases, Center for Laboratory Control of Infectious Diseases, Korea Centers for Diseases Control and Prevention	Division of Viral Diseases, Center for Laboratory Control of Infectious Diseases, Korea Centers for Diseases Control and Prevention	Jeong-Min Kim et al
EPI_ISL_506987	Division of Viral Diseases, Center for Laboratory Control of Infectious Diseases, Korea Centers for Diseases Control and Prevention	Division of Viral Diseases, Center for Laboratory Control of Infectious Diseases, Korea Centers for Diseases Control and Prevention	Jeong-Min Kim et al
EPI_ISL_506991	Division of Viral Diseases, Center for Laboratory Control of Infectious Diseases, Korea Centers for Diseases Control and Prevention	Division of Viral Diseases, Center for Laboratory Control of Infectious Diseases, Korea Centers for Diseases Control and Prevention	Jeong-Min Kim et al
EPI_ISL_510627	Division of Viral Diseases, Center for Laboratory Control of Infectious Diseases, Korea Centers for Diseases Control and Prevention	Division of Viral Diseases, Center for Laboratory Control of Infectious Diseases, Korea Centers for Diseases Control and Prevention	Jeong-Min Kim et al
EPI_ISL_510636	Division of Viral Diseases, Center for Laboratory Control of Infectious Diseases, Korea Centers for Diseases Control and Prevention	Division of Viral Diseases, Center for Laboratory Control of Infectious Diseases, Korea Centers for Diseases Control and Prevention	Jeong-Min Kim et al
EPI_ISL_514830	Division of Viral Diseases, Center for Laboratory Control of Infectious Diseases, Korea Centers for Diseases Control and Prevention	Division of Viral Diseases, Center for Laboratory Control of Infectious Diseases, Korea Centers for Diseases Control and Prevention	Jeong-Min Kim et al

EPI_ISL_514884	Division of Viral Diseases, Center for Laboratory Control of Infectious Diseases, Korea Centers for Diseases Control and Prevention	Division of Viral Diseases, Center for Laboratory Control of Infectious Diseases, Korea Centers for Diseases Control and Prevention	Jeong-Min Kim et al
EPI_ISL_522490	Division of Viral Diseases, Center for Laboratory Control of Infectious Diseases, Korea Centers for Diseases Control and Prevention	Division of Viral Diseases, Center for Laboratory Control of Infectious Diseases, Korea Centers for Diseases Control and Prevention	Jeong-Min Kim et al
EPI_ISL_1039541	LSUHS Emerging Viral Threat Laboratory	Microbial Genome Sequencing Center	Jeremy P. Kamil et al
EPI_ISL_913073	Center for Virology	Center for Virology	Jeremy V. Camp et al
EPI_ISL_913064	Center for Virology	Center for Virology	Jeremy V. Camp et al
EPI_ISL_913091	Center for Virology	Center for Virology	Jeremy V. Camp et al
EPI_ISL_913063	Labor Mustafa Wien	Center for Virology	Jeremy V. Camp et al
EPI_ISL_913079	Center for Virology	Center for Virology	Jeremy V. Camp et al
EPI_ISL_1056465	National Laboratory for Health, Environment and Food	CISLD (Clinical Institute of Special Laboratory Diagnostics), University Children's Hospital, University Medical Center Ljubljana	Jernej Kovač et al
EPI_ISL_1056460	National Laboratory for Health, Environment and Food	CISLD (Clinical Institute of Special Laboratory Diagnostics), University Children's Hospital, University Medical Center Ljubljana	Jernej Kovač et al
EPI_ISL_1240606	National Laboratory for Health, Environment and Food, OMM, Maribor	CISLD (Clinical Institute of Special Laboratory Diagnostics), University Children's Hospital, University Medical Center Ljubljana	Jernej Kovač et al
EPI_ISL_1267014	National Laboratory for Health, Environment and Food, OMM, Maribor	CISLD (Clinical Institute of Special Laboratory Diagnostics), University Children's Hospital, University Medical Center Ljubljana	Jernej Kovač et al
EPI_ISL_1266463	National Laboratory for Health, Environment and Food, OMM, Kranj	CISLD (Clinical Institute of Special Laboratory Diagnostics), University Children's Hospital, University Medical Center Ljubljana	Jernej Kovač et al
EPI_ISL_1335466	National Laboratory for Health, Environment and Food, OMM, Celje	CISLD (Clinical Institute of Special Laboratory Diagnostics), University Children's Hospital, University Medical Center Ljubljana	Jernej Kovač et al
EPI_ISL_1335712	National Laboratory for Health, Environment and Food, OMM, Celje	CISLD (Clinical Institute of Special Laboratory Diagnostics), University Children's Hospital, University Medical Center Ljubljana	Jernej Kovač et al
EPI_ISL_1056256	National Laboratory for Health, Environment and Food	CISLD (Clinical Institute of Special Laboratory Diagnostics), University Children's Hospital, University Medical Center Ljubljana	Jernej Kovač et al
EPI_ISL_1056454	National Laboratory for Health, Environment and Food	CISLD (Clinical Institute of Special Laboratory Diagnostics), University Children's Hospital, University Medical Center Ljubljana	Jernej Kovač et al
EPI_ISL_1055853	National Laboratory for Health, Environment and Food	CISLD (Clinical Institute of Special Laboratory Diagnostics), University Children's Hospital, University Medical Center Ljubljana	Jernej Kovač et al
EPI_ISL_458081	RSUD Bangil Pasuruan	Institute of Tropical Disease, Universitas Airlangga	Jezzy R Dewantari et al
EPI_ISL_759961	Universitas Airlangga Hospital	Institute of Tropical Disease, Universitas Airlangga	Jezzy R Dewantari et al
EPI_ISL_406031	Centers for Disease Control, R.O.C. (Taiwan)	Centers for Disease Control, R.O.C. (Taiwan)	Ji-Rong Yang et al
EPI_ISL_411926	Taiwan Centers for Disease Control	Taiwan Centers for Disease Control	Ji-Rong Yang et al
EPI_ISL_428489	Centers for Disease Control, R.O.C. (Taiwan)	Centers for Disease Control, R.O.C. (Taiwan)	Ji-Rong Yang et al
EPI_ISL_428488	Centers for Disease Control, R.O.C. (Taiwan)	Centers for Disease Control, R.O.C. (Taiwan)	Ji-Rong Yang et al
EPI_ISL_413868	Guangdong Provincial Institution of Public Health, Guangdong Provinical Center for Disease Control and Prevention	Guangdong Provincial Institution of Public Health	Jing Lu et al

EPI_ISL_413873	Guangdong Provincial Institution of Public Health, Guangdong Provinical Center for Disease Control and Prevention	Guangdong Provincial Institution of Public Health	Jing Lu et al
EPI_ISL_509711	Belize Ministry of Health	Pathogen Discovery, Respiratory Viruses Branch, Division of Viral Diseases, Centers for Disease Control and Prevention	Jing Zhang et al
EPI_ISL_509712	Belize Ministry of Health	Pathogen Discovery, Respiratory Viruses Branch, Division of Viral Diseases, Centers for Disease Control and Prevention	Jing Zhang et al
EPI_ISL_509713	Belize Ministry of Health	Pathogen Discovery, Respiratory Viruses Branch, Division of Viral Diseases, Centers for Disease Control and Prevention	Jing Zhang et al
EPI_ISL_509714	Belize Ministry of Health	Pathogen Discovery, Respiratory Viruses Branch, Division of Viral Diseases, Centers for Disease Control and Prevention	Jing Zhang et al
EPI_ISL_463902	Shaoxing Center for Disease Control and Prevention	Department of Pathology and Laboratory Medicine, University of California Los Angeles	Jinkun Chen et al
EPI_ISL_1346626	Center of Excellence in Clinical Virology	Research Unit of Systems Microbiology	Jiratchaya Puenpa et al
EPI_ISL_420412	KU Leuven, Clinical and Epidemiological Virology	KU Leuven, Clinical and Epidemiological Virology	Joan Marti-Carreras et al
EPI_ISL_960776	Germano de sousa	Instituto Gulbenkian de Ciencia	João Costa et al
EPI_ISL_491234	Instituto Gulbenkian de Ciência	Instituto Gulbenkian de Ciência	Joao Sobral et al
EPI_ISL_1396423	Centro de Tecnología en Salud Pública de la Universidad Nacional de Rosario	Laboratorio Mixto de Biotecnología Acuática (LMBA) on behalf of 'Proyecto Argentino Interinstitucional de genomica de SARS-CoV-2' (PAIS Consortium)	Joaquín Ezpeleta et al
EPI_ISL_1396459	Centro de Tecnología en Salud Pública de la Universidad Nacional de Rosario	Laboratorio Mixto de Biotecnología Acuática (LMBA) on behalf of 'Proyecto Argentino Interinstitucional de genomica de SARS-CoV-2' (PAIS Consortium)	Joaquín Ezpeleta et al
EPI_ISL_1396460	Centro de Tecnología en Salud Pública de la Universidad Nacional de Rosario	Laboratorio Mixto de Biotecnología Acuática (LMBA) on behalf of 'Proyecto Argentino Interinstitucional de genomica de SARS-CoV-2' (PAIS Consortium)	Joaquín Ezpeleta et al
EPI_ISL_648209	INBIRS-UBA	Laboratorio Mixto de Biotecnología Acuática (LMBA)	Joaquín Ezpeleta et al
EPI_ISL_648214	INBIRS-UBA	Laboratorio Mixto de Biotecnología Acuática (LMBA)	Joaquín Ezpeleta et al
EPI_ISL_424627	Instituto Nacional de Enfermedades Respiratorias	Instituto Nacional de Enfermedades Respiratorias	Joel Armando Vázquez Pérez et al
EPI_ISL_695611	AZ SPHL, Arizona Department of Health Services	TGen North	Jolene Bowers et al
EPI_ISL_1289905	Department of Public Health Microbiology Ljubljana, National Laboratory for Health, Environment and Food	Department of Public Health Microbiology Ljubljana, National Laboratory for Health, Environment and Food	José Gonçalves et al
EPI_ISL_1289911	Department of Public Health Microbiology Ljubljana, National Laboratory for Health, Environment and Food	Department of Public Health Microbiology Ljubljana, National Laboratory for Health, Environment and Food	José Gonçalves et al
EPI_ISL_1289912	Department of Public Health Microbiology Ljubljana, National Laboratory for Health, Environment and Food	Department of Public Health Microbiology Ljubljana, National Laboratory for Health, Environment and Food	José Gonçalves et al
EPI_ISL_1289913	Department of Public Health Microbiology Ljubljana, National Laboratory for Health, Environment and Food	Department of Public Health Microbiology Ljubljana, National Laboratory for Health, Environment and Food	José Gonçalves et al
EPI_ISL_1289915	Department of Public Health Microbiology Ljubljana, National Laboratory for Health, Environment and Food	Department of Public Health Microbiology Ljubljana, National Laboratory for Health, Environment and Food	José Gonçalves et al
EPI_ISL_1289916	Department of Public Health Microbiology Ljubljana, National Laboratory for Health, Environment and Food	Department of Public Health Microbiology Ljubljana, National Laboratory for Health, Environment and Food	José Gonçalves et al

EPI_ISL_1289917	Department of Public Health Microbiology Ljubljana, National Laboratory for Health, Environment and Food	Department of Public Health Microbiology Ljubljana, National Laboratory for Health, Environment and Food	José Gonçalves et al
EPI_ISL_1289919	Department of Public Health Microbiology Ljubljana, National Laboratory for Health, Environment and Food	Department of Public Health Microbiology Ljubljana, National Laboratory for Health, Environment and Food	José Gonçalves et al
EPI_ISL_1289925	Department of Public Health Microbiology Ljubljana, National Laboratory for Health, Environment and Food	Department of Public Health Microbiology Ljubljana, National Laboratory for Health, Environment and Food	José Gonçalves et al
EPI_ISL_1289927	Department of Public Health Microbiology Ljubljana, National Laboratory for Health, Environment and Food	Department of Public Health Microbiology Ljubljana, National Laboratory for Health, Environment and Food	José Gonçalves et al
EPI_ISL_1289875	Department of Public Health Microbiology Ljubljana, National Laboratory for Health, Environment and Food	Department of Public Health Microbiology Ljubljana, National Laboratory for Health, Environment and Food	José Gonçalves et al
EPI_ISL_1289876	Department of Public Health Microbiology Ljubljana, National Laboratory for Health, Environment and Food	Department of Public Health Microbiology Ljubljana, National Laboratory for Health, Environment and Food	José Gonçalves et al
EPI_ISL_1289892	Department of Public Health Microbiology Ljubljana, National Laboratory for Health, Environment and Food	Department of Public Health Microbiology Ljubljana, National Laboratory for Health, Environment and Food	José Gonçalves et al
EPI_ISL_541038	Hospital Clínico Universitario de Santiago de Compostela	SeqCOVID-SPAIN consortium/Institute of Biomedicine of Valencia, IBV-CSIC	José Javier Costa Alcalde et al
EPI_ISL_541066	Hospital Clínico Universitario de Santiago de Compostela	SeqCOVID-SPAIN consortium/Institute of Biomedicine of Valencia, IBV-CSIC	José Javier Costa Alcalde et al
EPI_ISL_476161	Laboratório de Patologia Clínica - UNICAMP	Laboratório de Estudos de Vírus Emergentes - UNICAMP	José Luiz Proença-Modena et al
EPI_ISL_1620750	National Virus Reference Laboratory	Irish Coronavirus Sequencing Consortium - Teagasc Moorepark	Jose Sanchez-Morgado et al
EPI_ISL_1620748	National Virus Reference Laboratory	Irish Coronavirus Sequencing Consortium - Teagasc Moorepark	Jose Sanchez-Morgado et al
EPI_ISL_1620704	National Virus Reference Laboratory	Irish Coronavirus Sequencing Consortium - Teagasc Moorepark	Jose Sanchez-Morgado et al
EPI_ISL_1620740	National Virus Reference Laboratory	Irish Coronavirus Sequencing Consortium - Teagasc Moorepark	Jose Sanchez-Morgado et al
EPI_ISL_1620712	National Virus Reference Laboratory	Irish Coronavirus Sequencing Consortium - Teagasc Moorepark	Jose Sanchez-Morgado et al
EPI_ISL_1378833	Instituto de Medicina Tropical & Salud Global Universidad Iberoamericana	Grubaugh Lab - Yale School of Public Health	Joseph Fauver et al
EPI_ISL_1378834	Instituto de Medicina Tropical & Salud Global Universidad Iberoamericana	Grubaugh Lab - Yale School of Public Health	Joseph Fauver et al
EPI_ISL_1378837	Instituto de Medicina Tropical & Salud Global Universidad Iberoamericana	Grubaugh Lab - Yale School of Public Health	Joseph Fauver et al
EPI_ISL_1378839	Instituto de Medicina Tropical & Salud Global Universidad Iberoamericana	Grubaugh Lab - Yale School of Public Health	Joseph Fauver et al
EPI_ISL_1378842	Instituto de Medicina Tropical & Salud Global Universidad Iberoamericana	Grubaugh Lab - Yale School of Public Health	Joseph Fauver et al
EPI_ISL_1378844	Instituto de Medicina Tropical & Salud Global Universidad Iberoamericana	Grubaugh Lab - Yale School of Public Health	Joseph Fauver et al
EPI_ISL_729754	Connecticut Department of Health	Grubaugh Lab - Yale School of Public Health	Joseph Fauver et al
EPI_ISL_729744	Connecticut Department of Health	Grubaugh Lab - Yale School of Public Health	Joseph Fauver et al
EPI_ISL_729748	Connecticut Department of Health	Grubaugh Lab - Yale School of Public Health	Joseph Fauver et al
EPI_ISL_422397	NMIMR, Department of Virology	WACCBIP, University of Ghana	Joyce M. Ngoi et al
EPI_ISL_422402	NMIMR, Department of Virology	WACCBIP, University of Ghana	Joyce M. Ngoi et al
EPI_ISL_422403	NMIMR, Department of Virology	WACCBIP, University of Ghana	Joyce M. Ngoi et al

EPI_ISL_1120498	Dutch COVID-19 response team	Medical Microbiology, Maastricht University Medical Centre	Jozef Dingemans* et al
EPI_ISL_965656	Dutch COVID-19 response team	Medical Microbiology, Maastricht University Medical Centre	Jozef Dingemans* et al
EPI_ISL_1120515	Dutch COVID-19 response team	Medical Microbiology, Maastricht University Medical Centre	Jozef Dingemans* et al
EPI_ISL_965567	Dutch COVID-19 response team	Medical Microbiology, Maastricht University Medical Centre	Jozef Dingemans* et al
EPI_ISL_447767	Instituto Nacional de Salud, Bogotá, Colombia	Grupo de Investigaciones Microbiológicas-UR (GIMUR), Departamento de Biología, Facultad de Ciencias Naturales, Universidad del Rosario, Bogotá, Colombia Instituto Nacional de Salud, Bogotá, Colombia Icahn School of Medicine at Mount Sinai, New York, USA	Juan David Ramírez et al
EPI_ISL_447734	Grupo de Investigaciones Microbiológicas-UR (GIMUR), Departamento de Biología, Facultad de Ciencias Naturales, Universidad del Rosario, Bogotá, Colombia	Grupo de Investigaciones Microbiológicas-UR (GIMUR), Departamento de Biología, Facultad de Ciencias Naturales, Universidad del Rosario, Bogotá, Colombia Instituto Nacional de Salud, Bogotá, Colombia Icahn School of Medicine at Mount Sinai, New York, USA	Juan David Ramírez et al
EPI_ISL_447751	Grupo de Investigaciones Microbiológicas-UR (GIMUR), Departamento de Biología, Facultad de Ciencias Naturales, Universidad del Rosario, Bogotá, Colombia	Grupo de Investigaciones Microbiológicas-UR (GIMUR), Departamento de Biología, Facultad de Ciencias Naturales, Universidad del Rosario, Bogotá, Colombia Instituto Nacional de Salud, Bogotá, Colombia Icahn School of Medicine at Mount Sinai, New York, USA	Juan David Ramírez et al
EPI_ISL_1040924	Grupo de Investigación en Enfermedades Tropicales del Ejército (GINETEJ), Laboratorio de Referencia e Investigación, Dirección de Sanidad Ejército, Bogotá, Colombia	Centro de Investigaciones en Microbiología y Biotecnología-UR (CIMBIUR), Facultad de Ciencias Naturales, Universidad del Rosario, Bogotá, Colombia	Juan David Ramírez et al
EPI_ISL_447757	Instituto Nacional de Salud, Bogotá, Colombia	Grupo de Investigaciones Microbiológicas-UR (GIMUR), Departamento de Biología, Facultad de Ciencias Naturales, Universidad del Rosario, Bogotá, Colombia Instituto Nacional de Salud, Bogotá, Colombia Icahn School of Medicine at Mount Sinai, New York, USA	Juan David Ramírez et al
EPI_ISL_447784	Instituto Nacional de Salud, Bogotá, Colombia	Grupo de Investigaciones Microbiológicas-UR (GIMUR), Departamento de Biología, Facultad de Ciencias Naturales, Universidad del Rosario, Bogotá, Colombia Instituto Nacional de Salud, Bogotá, Colombia Icahn School of Medicine at Mount Sinai, New York, USA	Juan David Ramírez et al
EPI_ISL_477016	Institute of Microbiology, Universidad San Francisco de Quito	Institute of Microbiology, Universidad San Francisco de Quito	Juan José Guadalupe et al
EPI_ISL_516648	Institute of Microbiology, Universidad San Francisco de Quito	Institute of Microbiology, Universidad San Francisco de Quito	Juan José Guadalupe et al
EPI_ISL_516649	Institute of Microbiology, Universidad San Francisco de Quito	Institute of Microbiology, Universidad San Francisco de Quito	Juan José Guadalupe et al
EPI_ISL_525430	Institute of Microbiology, Universidad San Francisco de Quito	Institute of Microbiology, Universidad San Francisco de Quito	Juan José Guadalupe et al
EPI_ISL_525432	Institute of Microbiology, Universidad San Francisco de Quito	Institute of Microbiology, Universidad San Francisco de Quito	Juan José Guadalupe et al
EPI_ISL_525433	Institute of Microbiology, Universidad San Francisco de Quito	Institute of Microbiology, Universidad San Francisco de Quito	Juan José Guadalupe et al
EPI_ISL_525436	Institute of Microbiology, Universidad San Francisco de Quito	Institute of Microbiology, Universidad San Francisco de Quito	Juan José Guadalupe et al
EPI_ISL_513514	Programa de Oncovirologia, Instituto Nacional de Câncer	Programa de Oncovirologia, Instituto Nacional de Câncer	Juliana D. Siqueira et al
EPI_ISL_513519	Programa de Oncovirologia, Instituto Nacional de Câncer	Programa de Oncovirologia, Instituto Nacional de Câncer	Juliana D. Siqueira et al
EPI_ISL_513532	Programa de Oncovirologia, Instituto Nacional de Câncer	Programa de Oncovirologia, Instituto Nacional de Câncer	Juliana D. Siqueira et al
EPI_ISL_513557	Programa de Oncovirologia, Instituto Nacional de Câncer	Programa de Oncovirologia, Instituto Nacional de Câncer	Juliana D. Siqueira et al
EPI_ISL_513566	Programa de Oncovirologia, Instituto Nacional de Câncer	Programa de Oncovirologia, Instituto Nacional de Câncer	Juliana D. Siqueira et al

EPI_ISL_513573	Programa de Oncovirologia, Instituto Nacional de Câncer	Programa de Oncovirologia, Instituto Nacional de Câncer	Juliana D. Siqueira et al
EPI_ISL_513577	Programa de Oncovirologia, Instituto Nacional de Câncer	Programa de Oncovirologia, Instituto Nacional de Câncer	Juliana D. Siqueira et al
EPI_ISL_513578	Programa de Oncovirologia, Instituto Nacional de Câncer	Programa de Oncovirologia, Instituto Nacional de Câncer	Juliana D. Siqueira et al
EPI_ISL_516783	Center for Laboratory Control of Infectious Diseases, Korea Centers for Diseases Control and Prevention	Center for Laboratory Control of Infectious Diseases, Korea Centers for Diseases Control and Prevention	Junyoung Kim et al
EPI_ISL_522491	Center for Laboratory Control of Infectious Diseases, Korea Centers for Diseases Control and Prevention	Center for Laboratory Control of Infectious Diseases, Korea Centers for Diseases Control and Prevention	Junyoung Kim et al
EPI_ISL_522502	Center for Laboratory Control of Infectious Diseases, Korea Centers for Diseases Control and Prevention	Center for Laboratory Control of Infectious Diseases, Korea Centers for Diseases Control and Prevention	Junyoung Kim et al
EPI_ISL_560391	Vilnius University Hospital Santaros Klinikos, Vilnius University	Institute of Biotechnology, Life Sciences Center, Vilnius University and Thermo Fisher Scientific	Justinas Slikas et al
EPI_ISL_560393	Vilnius University Hospital Santaros Klinikos, Vilnius University	Institute of Biotechnology, Life Sciences Center, Vilnius University and Thermo Fisher Scientific	Justinas Slikas et al
EPI_ISL_560395	Vilnius University Hospital Santaros Klinikos, Vilnius University	Institute of Biotechnology, Life Sciences Center, Vilnius University and Thermo Fisher Scientific	Justinas Slikas et al
EPI_ISL_560396	Vilnius University Hospital Santaros Klinikos, Vilnius University	Institute of Biotechnology, Life Sciences Center, Vilnius University and Thermo Fisher Scientific	Justinas Slikas et al
EPI_ISL_560398	Vilnius University Hospital Santaros Klinikos, Vilnius University	Institute of Biotechnology, Life Sciences Center, Vilnius University and Thermo Fisher Scientific	Justinas Slikas et al
EPI_ISL_560399	Vilnius University Hospital Santaros Klinikos, Vilnius University	Institute of Biotechnology, Life Sciences Center, Vilnius University and Thermo Fisher Scientific	Justinas Slikas et al
EPI_ISL_560405	Vilnius University Hospital Santaros Klinikos, Vilnius University	Institute of Biotechnology, Life Sciences Center, Vilnius University and Thermo Fisher Scientific	Justinas Slikas et al
EPI_ISL_1014680	National Influenza Center, Virology Department	National Influenza Center	K Sadeghi et al
EPI_ISL_1014685	National Influenza Center, Virology Department	National Influenza Center	K Sadeghi et al
EPI_ISL_862079	National Influenza Center, Virology Department	National Influenza Center	K Sadeghi et al
EPI_ISL_959279	National Influenza Center, Virology Department	National Influenza Center	K Sadeghi et al
EPI_ISL_779169	Laboratorio de Infectología, Servicio de Infectología, Hospital Universitario Dr. José Eleuterio González - Universidad Autónoma de Nuevo León	Laboratorio de Infectología Molecular, Departamento de Bioquímica y Medicina Molecular,Facultad de Medicina - Universidad Autónoma de Nuevo León	Kame A. Galán-Huerta et al
EPI_ISL_779176	Laboratorio de Infectología, Servicio de Infectología, Hospital Universitario Dr. José Eleuterio González - Universidad Autónoma de Nuevo León	Laboratorio de Infectología Molecular, Departamento de Bioquímica y Medicina Molecular,Facultad de Medicina - Universidad Autónoma de Nuevo León	Kame A. Galán-Huerta et al
EPI_ISL_779188	Laboratorio Estatal de Salud Pública de Nuevo León	Laboratorio de Infectología Molecular, Departamento de Bioquímica y Medicina Molecular,Facultad de Medicina - Universidad Autónoma de Nuevo León	Kame A. Galán-Huerta et al
EPI_ISL_979331	Laboratorio Estatal de Salud Pública de Nuevo León	Laboratorio de Infectología Molecular, Departamento de Bioquímica y Medicina Molecular,Facultad de Medicina - Universidad Autónoma de Nuevo León	Kame A. Galán-Huerta et al
EPI_ISL_961794	Laboratorio de Infectología, Servicio de Infectología, Hospital Universitario Dr. José Eleuterio González - Universidad Autónoma de Nuevo León	Laboratorio de Infectología Molecular, Departamento de Bioquímica y Medicina Molecular,Facultad de Medicina - Universidad Autónoma de Nuevo León	Kame A. Galán-Huerta et al

EPI_ISL_961791	Laboratorio de Infectología, Servicio de Infectología, Hospital Universitario Dr. José Eleuterio González - Universidad Autónoma de Nuevo León	Laboratorio de Infectología Molecular, Departamento de Bioquímica y Medicina Molecular,Facultad de Medicina - Universidad Autónoma de Nuevo León	Kame A. Galán-Huerta et al
EPI_ISL_961793	Laboratorio de Infectología, Servicio de Infectología, Hospital Universitario Dr. José Eleuterio González - Universidad Autónoma de Nuevo León	Laboratorio de Infectología Molecular, Departamento de Bioquímica y Medicina Molecular,Facultad de Medicina - Universidad Autónoma de Nuevo León	Kame A. Galán-Huerta et al
EPI_ISL_961775	Laboratorio de Infectología, Servicio de Infectología, Hospital Universitario Dr. José Eleuterio González - Universidad Autónoma de Nuevo León	Laboratorio de Infectología Molecular, Departamento de Bioquímica y Medicina Molecular,Facultad de Medicina - Universidad Autónoma de Nuevo León	Kame A. Galán-Huerta et al
EPI_ISL_961780	Laboratorio de Infectología, Servicio de Infectología, Hospital Universitario Dr. José Eleuterio González - Universidad Autónoma de Nuevo León	Laboratorio de Infectología Molecular, Departamento de Bioquímica y Medicina Molecular,Facultad de Medicina - Universidad Autónoma de Nuevo León	Kame A. Galán-Huerta et al
EPI_ISL_961773	Laboratorio de Infectología, Servicio de Infectología, Hospital Universitario Dr. José Eleuterio González - Universidad Autónoma de Nuevo León	Laboratorio de Infectología Molecular, Departamento de Bioquímica y Medicina Molecular,Facultad de Medicina - Universidad Autónoma de Nuevo León	Kame A. Galán-Huerta et al
EPI_ISL_961766	Laboratorio de Infectología, Servicio de Infectología, Hospital Universitario Dr. José Eleuterio González - Universidad Autónoma de Nuevo León	Laboratorio de Infectología Molecular, Departamento de Bioquímica y Medicina Molecular,Facultad de Medicina - Universidad Autónoma de Nuevo León	Kame A. Galán-Huerta et al
EPI_ISL_1091266	Laboratorio PGM	Laboratorio de Infectología Molecular, Departamento de Bioquímica y Medicina Molecular,Facultad de Medicina - Universidad Autónoma de Nuevo León	Kame A. Galán-Huerta et al
EPI_ISL_1091259	Laboratorio PGM	Laboratorio de Infectología Molecular, Departamento de Bioquímica y Medicina Molecular,Facultad de Medicina - Universidad Autónoma de Nuevo León	Kame A. Galán-Huerta et al
EPI_ISL_728561	Institute for Urban Disease Control and Prevention	COVID-19 Network Investigations (CONI) Alliance	Kamolthip Atsawawaranunt et al
EPI_ISL_882773	Institute for Urban Disease Control and Prevention	COVID-19 Network Investigations (CONI) Alliance	Kamolthip Atsawawaranunt et al
EPI_ISL_733552	Institute for Urban Disease Control and Prevention	COVID-19 Network Investigations (CONI) Alliance	Kamolthip Atsawawaranunt et al
EPI_ISL_812941	Institute for Urban Disease Control and Prevention	COVID-19 Network Investigations (CONI) Alliance	Kamolthip Atsawawaranunt et al
EPI_ISL_561338	Civil Hospital, Panchkula	CSIR-Institute of Microbial Technology	Kanika Bansal et al
EPI_ISL_493137	Center for Research and Innovation, Faculty of Medical Technology, Mahidol University	Center for Research and Innovation, Faculty of Medical Technology, Mahidol University	Kantima Sangsiriwut et al
EPI_ISL_437300	Diagnostic- and Research Institute of Pathology, Medical University of Graz	Diagnostic- and Research Institute of Pathology, Medical University of Graz	Karl Kashofer et al
EPI_ISL_1169051	National Institute of Health Research and Development	National Institute of Health Research and Development	Kartika Dewi Puspa et al
EPI_ISL_1257844	RSUP Fatmawati	National Institute of Health Research and Development	Kartika Dewi Puspa et al
EPI_ISL_437895	Laboratory of Microbiology, Medical School, National and Kapodistrian University of Athens	Laboratory of Biology, Department of Medicine, Democritus University of Thrace	Kassela K. et al
EPI_ISL_437908	Laboratory of Microbiology, Medical School, National and Kapodistrian University of Athens	Laboratory of Biology, Department of Medicine, Democritus University of Thrace	Kassela K. et al
EPI_ISL_418809	University of Wisconsin - Madison: Influenza Research Institute	University of Wisconsin Madison, AIDS Vaccine Research Laboratories	Katarina Braun et al
EPI_ISL_960308	NLZOH, Laboratory for Virology	NLZOH, Laboratory for Virology	Katarina Prosenc (Laboratory for Virology) e
EPI_ISL_960306	NLZOH, Laboratory for Virology	NLZOH, Laboratory for Virology	Katarina Prosenc (Laboratory for Virology) e

et al
et al

EPI_ISL_960304	NLZOH, Laboratory for Virology	NLZOH, Laboratory for Virology	Katarina Prosenc (Laboratory for Virology) e
EPI_ISL_455441	1. ViroGenetics - BSL3 Laboratory of Virology, Małopolska Centre of Biotechnology, Jagiellonian University; 2. II Department of Internal Medicine, Faculty of Medicine, Jagiellonian University Medical College; 3. Narodowy Instytut Zdrowia Publicznego – Państwowy Zakład Higieny (NIZP-PZH)	1. ViroGenetics - BSL3 Laboratory of Virology, Małopolska Centre of Biotechnology, Jagiellonian University; 2. II Department of Internal Medicine, Faculty of Medicine, Jagiellonian University Medical College; 3. Narodowy Instytut Zdrowia Publicznego – Państwowy Zakład Higieny (NIZP-PZH).	Katarzyna Pancer et al
EPI_ISL_455443	1. ViroGenetics - BSL3 Laboratory of Virology, Małopolska Centre of Biotechnology, Jagiellonian University; 2. II Department of Internal Medicine, Faculty of Medicine, Jagiellonian University Medical College; 3. Narodowy Instytut Zdrowia Publicznego – Państwowy Zakład Higieny (NIZP-PZH)	1. ViroGenetics - BSL3 Laboratory of Virology, Małopolska Centre of Biotechnology, Jagiellonian University; 2. II Department of Internal Medicine, Faculty of Medicine, Jagiellonian University Medical College; 3. Narodowy Instytut Zdrowia Publicznego – Państwowy Zakład Higieny (NIZP-PZH).	Katarzyna Pancer et al
EPI_ISL_455448	1. ViroGenetics - BSL3 Laboratory of Virology, Małopolska Centre of Biotechnology, Jagiellonian University; 2. II Department of Internal Medicine, Faculty of Medicine, Jagiellonian University Medical College; 3. Narodowy Instytut Zdrowia Publicznego – Państwowy Zakład Higieny (NIZP-PZH)	1. ViroGenetics - BSL3 Laboratory of Virology, Małopolska Centre of Biotechnology, Jagiellonian University; 2. II Department of Internal Medicine, Faculty of Medicine, Jagiellonian University Medical College; 3. Narodowy Instytut Zdrowia Publicznego – Państwowy Zakład Higieny (NIZP-PZH).	Katarzyna Pancer et al
EPI_ISL_848146	National Virus Reference Laboratory	Irish Coronavirus Sequencing Consortium - National University of Ireland Galway	Kate Reddington et al
EPI_ISL_848084	National Virus Reference Laboratory	Irish Coronavirus Sequencing Consortium - National University of Ireland Galway	Kate Reddington et al
EPI_ISL_848101	National Virus Reference Laboratory	Irish Coronavirus Sequencing Consortium - National University of Ireland Galway	Kate Reddington et al
EPI_ISL_848113	National Virus Reference Laboratory	Irish Coronavirus Sequencing Consortium - National University of Ireland Galway	Kate Reddington et al
EPI_ISL_1137618	Dirección de Sanidad Policia Nacional	Instituto Nacional de Salud- Dirección de Investigación en Salud Pública	Katherine Laiton-Donato et al
EPI_ISL_1137619	Laboratorio de Salud Publica de Cauca	Instituto Nacional de Salud- Dirección de Investigación en Salud Pública	Katherine Laiton-Donato et al
EPI_ISL_1137615	Laboratorio de Salud Publica de Cauca	Instituto Nacional de Salud- Dirección de Investigación en Salud Pública	Katherine Laiton-Donato et al
EPI_ISL_1303374	CLINICA COLSANITAS CENTRAL DE REFERENCIA	Instituto Nacional de Salud- Dirección de Investigación en Salud Pública	Katherine Laiton-Donato et al
EPI_ISL_1303373	Dirección de Sanidad Policia Nacional	Instituto Nacional de Salud- Dirección de Investigación en Salud Pública	Katherine Laiton-Donato et al
EPI_ISL_1303372	Dirección de Sanidad Policia Nacional	Instituto Nacional de Salud- Dirección de Investigación en Salud Pública	Katherine Laiton-Donato et al
EPI_ISL_1303371	Dirección de Sanidad Policia Nacional	Instituto Nacional de Salud- Dirección de Investigación en Salud Pública	Katherine Laiton-Donato et al
EPI_ISL_1303366	Instituto Nacional de Salud- Dirección de Redes de Laboratorios de Salud Pública	Instituto Nacional de Salud- Dirección de Investigación en Salud Pública	Katherine Laiton-Donato et al
EPI_ISL_1303367	Instituto Nacional de Salud- Dirección de Redes de Laboratorios de Salud Pública	Instituto Nacional de Salud- Dirección de Investigación en Salud Pública	Katherine Laiton-Donato et al
EPI_ISL_1303377	Instituto Nacional de Salud- Dirección de Redes de Laboratorios de Salud Pública	Instituto Nacional de Salud- Dirección de Investigación en Salud Pública	Katherine Laiton-Donato et al
EPI_ISL_1632511	LDSP Amazonas	Instituto Nacional de Salud- Dirección de Investigación en Salud Pública	Katherine Laiton-Donato et al
EPI_ISL_1652041	LDSP BOGOTA	Instituto Nacional de Salud- Dirección de Investigación en Salud Pública	Katherine Laiton-Donato et al
EPI_ISL_956297	Instituto Nacional de Salud- Dirección de Redes de Laboratorios de Salud Pública	Instituto Nacional de Salud- Dirección de Investigación en Salud Pública	Katherine Laiton-Donato et al

et al

EPI_ISL_956288	Instituto Nacional de Salud- Dirección de Redes de Laboratorios de Salud Pública	Instituto Nacional de Salud- Dirección de Investigación en Salud Pública	Katherine Laiton-Donato et al
EPI_ISL_956289	Instituto Nacional de Salud- Dirección de Redes de Laboratorios de Salud Pública	Instituto Nacional de Salud- Dirección de Investigación en Salud Pública	Katherine Laiton-Donato et al
EPI_ISL_956291	Instituto Nacional de Salud- Dirección de Redes de Laboratorios de Salud Pública	Instituto Nacional de Salud- Dirección de Investigación en Salud Pública	Katherine Laiton-Donato et al
EPI_ISL_956292	Instituto Nacional de Salud- Dirección de Redes de Laboratorios de Salud Pública	Instituto Nacional de Salud- Dirección de Investigación en Salud Pública	Katherine Laiton-Donato et al
EPI_ISL_956293	Instituto Nacional de Salud- Dirección de Redes de Laboratorios de Salud Pública	Instituto Nacional de Salud- Dirección de Investigación en Salud Pública	Katherine Laiton-Donato et al
EPI_ISL_956295	Instituto Nacional de Salud- Dirección de Redes de Laboratorios de Salud Pública	Instituto Nacional de Salud- Dirección de Investigación en Salud Pública	Katherine Laiton-Donato et al
EPI_ISL_653756	Instituto Nacional de Salud, Bogotá, Colombia	Instituto Nacional de Salud, Bogotá, Colombia	Katherine Laiton-Donato et al
EPI_ISL_739680	Instituto Nacional de Salud, Bogotá, Colombia	Instituto Nacional de Salud, Bogotá, Colombia	Katherine Laiton-Donato et al
EPI_ISL_739675	Instituto Nacional de Salud, Bogotá, Colombia	Instituto Nacional de Salud, Bogotá, Colombia	Katherine Laiton-Donato et al
EPI_ISL_739674	Instituto Nacional de Salud, Bogotá, Colombia	Instituto Nacional de Salud, Bogotá, Colombia	Katherine Laiton-Donato et al
EPI_ISL_739677	Instituto Nacional de Salud, Bogotá, Colombia	Instituto Nacional de Salud, Bogotá, Colombia	Katherine Laiton-Donato et al
EPI_ISL_739676	Instituto Nacional de Salud, Bogotá, Colombia	Instituto Nacional de Salud, Bogotá, Colombia	Katherine Laiton-Donato et al
EPI_ISL_653758	Instituto Nacional de Salud, Bogotá, Colombia	Instituto Nacional de Salud, Bogotá, Colombia	Katherine Laiton-Donato et al
EPI_ISL_1424059	Instituto Nacional de Salud- Dirección de Investigación en Salud Pública	Instituto Nacional de Salud- Dirección de Investigación en Salud Pública	Katherine Laiton-Donato et al
EPI_ISL_1424060	Instituto Nacional de Salud- Dirección de Investigación en Salud Pública	Instituto Nacional de Salud- Dirección de Investigación en Salud Pública	Katherine Laiton-Donato et al
EPI_ISL_1424061	Instituto Nacional de Salud- Dirección de Investigación en Salud Pública	Instituto Nacional de Salud- Dirección de Investigación en Salud Pública	Katherine Laiton-Donato et al
EPI_ISL_956300	Laboratorio de salud publica Arauca	Instituto Nacional de Salud- Dirección de Investigación en Salud Pública	Katherine Laiton-Donato et al
EPI_ISL_653761	Instituto Nacional de Salud, Bogotá, Colombia	Instituto Nacional de Salud, Bogotá, Colombia	Katherine Laiton-Donato et al
EPI_ISL_653760	Instituto Nacional de Salud, Bogotá, Colombia	Instituto Nacional de Salud, Bogotá, Colombia	Katherine Laiton-Donato et al
EPI_ISL_653762	Instituto Nacional de Salud, Bogotá, Colombia	Instituto Nacional de Salud, Bogotá, Colombia	Katherine Laiton-Donato et al
EPI_ISL_1220050	Laboratorio Clinico Clinica General del Norte	Instituto Nacional de Salud- Dirección de Investigación en Salud Pública	Katherine Laiton-Donato et al
EPI_ISL_845620	compensar calle 63	Instituto Nacional de Salud - Dirección de Investigación en Salud Pública	Katherine Laiton-Donato et al
EPI_ISL_845621	Dirección de Sanidad Ejército	Instituto Nacional de Salud - Dirección de Investigación en Salud Pública	Katherine Laiton-Donato et al
EPI_ISL_1632531	UNIVERSIDAD DEL SINU	Instituto Nacional de Salud- Dirección de Investigación en Salud Pública	Katherine Laiton-Donato et al
EPI_ISL_498158	Instituto Nacional de Salud, Bogotá, Colombia	Instituto Nacional de Salud, Bogotá, Colombia	Katherine Laiton-Donato et al
EPI_ISL_456123	Instituto Nacional de Salud - Unidad de Secuenciación y Análisis Genómico	Instituto Nacional de Salud, Universidad Cooperativa de Colombia, Instituto Alexander von Humboldt, Imperial College-London, London School of Hygiene & Tropical Medicine	Katherine Laiton-Donato et al
EPI_ISL_498168	Instituto Nacional de Salud, Bogotá, Colombia	Instituto Nacional de Salud, Bogotá, Colombia	Katherine Laiton-Donato et al

EPI_ISL_498170	Instituto Nacional de Salud, Bogotá, Colombia	Instituto Nacional de Salud, Bogotá, Colombia	Katherine Laiton-Donato et al
EPI_ISL_526969	Instituto Nacional de Salud, Bogotá, Colombia	Instituto Nacional de Salud, Bogotá, Colombia	Katherine Laiton-Donato et al
EPI_ISL_526974	Instituto Nacional de Salud, Bogotá, Colombia	Instituto Nacional de Salud, Bogotá, Colombia	Katherine Laiton-Donato et al
EPI_ISL_526971	Instituto Nacional de Salud, Bogotá, Colombia	Instituto Nacional de Salud, Bogotá, Colombia	Katherine Laiton-Donato et al
EPI_ISL_653745	Instituto Nacional de Salud, Bogotá, Colombia	Instituto Nacional de Salud, Bogotá, Colombia	Katherine Laiton-Donato et al
EPI_ISL_653746	Instituto Nacional de Salud, Bogotá, Colombia	Instituto Nacional de Salud, Bogotá, Colombia	Katherine Laiton-Donato et al
EPI_ISL_653747	Instituto Nacional de Salud, Bogotá, Colombia	Instituto Nacional de Salud, Bogotá, Colombia	Katherine Laiton-Donato et al
EPI_ISL_653754	Instituto Nacional de Salud, Bogotá, Colombia	Instituto Nacional de Salud, Bogotá, Colombia	Katherine Laiton-Donato et al
EPI_ISL_739673	Instituto Nacional de Salud, Bogotá, Colombia	Instituto Nacional de Salud, Bogotá, Colombia	Katherine Laiton-Donato et al
EPI_ISL_791086	Instituto Nacional de Salud - Unidad de Secuenciación y Análisis Genómico	Instituto Nacional de Salud - Dirección de Investigación en Salud Pública	Katherine Laiton-Donato et al
EPI_ISL_791084	Instituto Nacional de Salud - Unidad de Secuenciación y Análisis Genómico	Instituto Nacional de Salud - Dirección de Investigación en Salud Pública	Katherine Laiton-Donato et al
EPI_ISL_1092008	Laboratorio de Salud Publica de Bogota	Instituto Nacional de Salud- Dirección de Investigación en Salud Pública	Katherine Laiton-Donato et al
EPI_ISL_1494944	HOSPITAL DEPARTAMENTAL SAN VICENTE DE PAUL	Instituto Nacional de Salud- Dirección de Investigación en Salud Pública	Katherine Laiton-Donato et al
EPI_ISL_526959	Instituto Nacional de Salud, Bogotá, Colombia	Instituto Nacional de Salud, Bogotá, Colombia	Katherine Laiton-Donato et al
EPI_ISL_526958	Instituto Nacional de Salud, Bogotá, Colombia	Instituto Nacional de Salud, Bogotá, Colombia	Katherine Laiton-Donato et al
EPI_ISL_1582978	E.S.E. HOSPITAL SAN JOSE DE MAICAO	Instituto Nacional de Salud- Dirección de Investigación en Salud Pública	Katherine Laiton-Donato et al
EPI_ISL_1582979	E.S.E. HOSPITAL SAN JOSE DE MAICAO	Instituto Nacional de Salud- Dirección de Investigación en Salud Pública	Katherine Laiton-Donato et al
EPI_ISL_1582980	E.S.E. HOSPITAL SAN JOSE DE MAICAO	Instituto Nacional de Salud- Dirección de Investigación en Salud Pública	Katherine Laiton-Donato et al
EPI_ISL_1582989	Laboratorio de Biología Molecular – Universidad del Magdalena	Instituto Nacional de Salud- Dirección de Investigación en Salud Pública	Katherine Laiton-Donato et al
EPI_ISL_1582993	Universidad del Atlántico Laboratorio de Investigación en Biología Molecular	Instituto Nacional de Salud- Dirección de Investigación en Salud Pública	Katherine Laiton-Donato et al
EPI_ISL_1582983	HOSPITAL DEPARTAMENTAL DE VILLAVICENCIO	Instituto Nacional de Salud- Dirección de Investigación en Salud Pública	Katherine Laiton-Donato et al
EPI_ISL_498162	Instituto Nacional de Salud, Bogotá, Colombia	Instituto Nacional de Salud, Bogotá, Colombia	Katherine Laiton-Donato et al
EPI_ISL_956306	Laboratorio Gencore- Universidad de los Andes	Instituto Nacional de Salud- Dirección de Investigación en Salud Pública, Universidad de los Andes- Gencore	Katherine Laiton-Donato et al
EPI_ISL_1017707	Hospital Universitario Hernando Moncaleano Perdomo	Instituto Nacional de Salud- Dirección de Investigación en Salud Pública	Katherine Laiton-Donato et al
EPI_ISL_456128	Instituto Nacional de Salud - Unidad de Secuenciación y Análisis Genómico	Instituto Nacional de Salud, Universidad Cooperativa de Colombia, Instituto Alexander von Humboldt, Imperial College-London, London School of Hygiene & Tropical Medicine	Katherine Laiton-Donato et al
EPI_ISL_739663	Instituto Nacional de Salud, Bogotá, Colombia	Instituto Nacional de Salud, Bogotá, Colombia	Katherine Laiton-Donato et al
EPI_ISL_1494950	Laboratorio de Salud Publica de Santander	Instituto Nacional de Salud- Dirección de Investigación en Salud Pública	Katherine Laiton-Donato et al
EPI_ISL_1220065	Investigaciones Biomédicas - Universidad de Sucre	Instituto Nacional de Salud- Dirección de Investigación en Salud Pública	Katherine Laiton-Donato et al
EPI_ISL_1494940	Investigaciones Biomédicas - Universidad de Sucre	Instituto Nacional de Salud- Dirección de Investigación en Salud Pública	Katherine Laiton-Donato et al

EPI_ISL_653749	Instituto Nacional de Salud, Bogotá, Colombia	Instituto Nacional de Salud, Bogotá, Colombia	Katherine Laiton-Donato et al
EPI_ISL_456152	Instituto Nacional de Salud - Unidad de Secuenciación y Análisis Genómico	Instituto Nacional de Salud, Universidad Cooperativa de Colombia, Instituto Alexander von Humboldt, Imperial College-London, London School of Hygiene & Tropical Medicine	Katherine Laiton-Donato et al
EPI_ISL_707947	Virology, Universitätsklinikum des Saarlandes	Epigenetics, Saarland University	Kathrin Kattler et al
EPI_ISL_707948	Virology, Universitätsklinikum des Saarlandes	Epigenetics, Saarland University	Kathrin Kattler et al
EPI_ISL_708025	Virology, Universitätsklinikum des Saarlandes	Epigenetics, Saarland University	Kathrin Kattler et al
EPI_ISL_860285	Department of Medical Microbiology, St. Olavs hospital	Norwegian Institute of Public Health, Department of Virology	Kathrine Stene-Johansen et al
EPI_ISL_860287	Department of Medical Microbiology, St. Olavs hospital	Norwegian Institute of Public Health, Department of Virology	Kathrine Stene-Johansen et al
EPI_ISL_1034356	Department of Medical Microbiology, St. Olavs hospital	Norwegian Institute of Public Health, Department of Virology	Kathrine Stene-Johansen et al
EPI_ISL_1034357	Department of Medical Microbiology, St. Olavs hospital	Norwegian Institute of Public Health, Department of Virology	Kathrine Stene-Johansen et al
EPI_ISL_1034361	Department of Medical Microbiology, St. Olavs hospital	Norwegian Institute of Public Health, Department of Virology	Kathrine Stene-Johansen et al
EPI_ISL_417483	Oslo University Hospital, Department of Medical Microbiology	Norwegian Institute of Public Health	Kathrine Stene-Johansen et al
EPI_ISL_417484	Oslo University Hospital, Department of Medical Microbiology	Norwegian Institute of Public Health, Department of Virology	Kathrine Stene-Johansen et al
EPI_ISL_417490	Oslo University Hospital, Department of Medical Microbiology	Norwegian Institute of Public Health, Department of Virology	Kathrine Stene-Johansen et al
EPI_ISL_420135	Oslo University Hospital, Department of Medical Microbiology	Norwegian Institute of Public Health, Department of Virology	Kathrine Stene-Johansen et al
EPI_ISL_417488	Oslo University Hospital, Department of Medical Microbiology	Norwegian Institute of Public Health, Department of Virology	Kathrine Stene-Johansen et al
EPI_ISL_417489	Oslo University Hospital, Department of Medical Microbiology	Norwegian Institute of Public Health, Department of Virology	Kathrine Stene-Johansen et al
EPI_ISL_420134	Akershus University Hospital, Department for Microbiology and Infectious Disease Control	Norwegian Institute of Public Health, Department of Virology	Kathrine Stene-Johansen et al
EPI_ISL_417486	Hospital of Southern Norway - Kristiansand, Department of Medical Microbiology	Norwegian Institute of Public Health, Department of Virology	Kathrine Stene-Johansen et al
EPI_ISL_1118497	Department of Medical Microbiology, St. Olavs hospital	Norwegian Institute of Public Health, Department of Virology	Kathrine Stene-Johansen et al
EPI_ISL_420136	Akershus University Hospital, Department for Microbiology and Infectious Disease Control	Norwegian Institute of Public Health, Department of Virology	Kathrine Stene-Johansen et al
EPI_ISL_590915	Hospital of Southern Norway - Kristiansand, Department of Medical Microbiology	Norwegian Institute of Public Health, Department of Virology	Kathrine Stene-Johansen et al
EPI_ISL_420148	Ostfold Hospital Trust -Kalnes Centre for Laboratory Medicine Section for gene technology and infection serology	Norwegian Institute of Public Health, Department of Virology	Kathrine Stene-Johansen et al
EPI_ISL_420151	Nordland Hospital - Bodo, Laboratory Department, Molecular Biology Unit	Norwegian Institute of Public Health, Department of Virology	Kathrine Stene-Johansen et al
EPI_ISL_449788	Furst Medical Laboratory	Norwegian Institute of Public Health, Department of Virology	Kathrine Stene-Johansen et al
EPI_ISL_1317842	Furst Medical Laboratory	Norwegian Institute of Public Health, Department of Virology	Kathrine Stene-Johansen et al
EPI_ISL_481210	Ostfold Hospital Trust - Kalnes, Centre for Laboratory Medicine, Section for gene technology and infection serology	Norwegian Institute of Public Health, Department of Virology	Kathrine Stene-Johansen et al
EPI_ISL_481211	Ostfold Hospital Trust - Kalnes, Centre for Laboratory Medicine, Section for gene technology and infection serology	Norwegian Institute of Public Health, Department of Virology	Kathrine Stene-Johansen et al

EPI_ISL_481212	Ostfold Hospital Trust - Kalnes, Centre for Laboratory Medicine, Section for gene technology and infection serology	Norwegian Institute of Public Health, Department of Virology	Kathrine Stene-Johansen et al
EPI_ISL_481215	Oslo University Hospital, Department of Medical Microbiology	Norwegian Institute of Public Health, Department of Virology	Kathrine Stene-Johansen et al
EPI_ISL_1251957	Department of Medical Microbiology, St. Olavs hospital	Norwegian Institute of Public Health, Department of Virology	Kathrine Stene-Johansen et al
EPI_ISL_493355	Oslo University Hospital, Department of Medical Microbiology	Norwegian Institute of Public Health, Department of Virology	Kathrine Stene-Johansen et al
EPI_ISL_493358	Furst Medical Laboratory	Norwegian Institute of Public Health, Department of Virology	Kathrine Stene-Johansen et al
EPI_ISL_493369	Furst Medical Laboratory	Norwegian Institute of Public Health, Department of Virology	Kathrine Stene-Johansen et al
EPI_ISL_493380	Furst Medical Laboratory	Norwegian Institute of Public Health, Department of Virology	Kathrine Stene-Johansen et al
EPI_ISL_493382	Medical Microbiology Unit, Department for Laboratory Medicine, Drammen Hospital, Vestre Viken Health Trust,	Norwegian Institute of Public Health, Department of Virology	Kathrine Stene-Johansen et al
EPI_ISL_549038	Furst Medical Laboratory	Norwegian Institute of Public Health, Department of Virology	Kathrine Stene-Johansen et al
EPI_ISL_549068	Furst Medical Laboratory	Norwegian Institute of Public Health, Department of Virology	Kathrine Stene-Johansen et al
EPI_ISL_549056	Furst Medical Laboratory	Norwegian Institute of Public Health, Department of Virology	Kathrine Stene-Johansen et al
EPI_ISL_549057	Furst Medical Laboratory	Norwegian Institute of Public Health, Department of Virology	Kathrine Stene-Johansen et al
EPI_ISL_549084	Akershus University Hospital, Department for Microbiology and Infectious Disease Control	Norwegian Institute of Public Health, Department of Virology	Kathrine Stene-Johansen et al
EPI_ISL_1317969	Furst Medical Laboratory	Norwegian Institute of Public Health, Department of Virology	Kathrine Stene-Johansen et al
EPI_ISL_1192300	Department of Medical Microbiology, St. Olavs hospital	Norwegian Institute of Public Health, Department of Virology	Kathrine Stene-Johansen et al
EPI_ISL_1318016	Furst Medical Laboratory	Norwegian Institute of Public Health, Department of Virology	Kathrine Stene-Johansen et al
EPI_ISL_1490489	Furst Medical Laboratory	Norwegian Institute of Public Health, Department of Virology	Kathrine Stene-Johansen et al
EPI_ISL_635161	Hospital of Southern Norway - Kristiansand, Department of Medical Microbiology	Norwegian Institute of Public Health, Department of Virology	Kathrine Stene-Johansen et al
EPI_ISL_668394	Hospital of Southern Norway - Kristiansand, Department of Medical Microbiology	Norwegian Institute of Public Health, Department of Virology	Kathrine Stene-Johansen et al
EPI_ISL_1252548	Furst Medical Laboratory	Norwegian Institute of Public Health, Department of Virology	Kathrine Stene-Johansen et al
EPI_ISL_708059	Furst Medical Laboratory	Norwegian Institute of Public Health, Department of Virology	Kathrine Stene-Johansen et al
EPI_ISL_1547525	Furst Medical Laboratory	Norwegian Institute of Public Health, Department of Virology	Kathrine Stene-Johansen et al
EPI_ISL_1579172	Furst Medical Laboratory	Norwegian Institute of Public Health, Department of Virology	Kathrine Stene-Johansen et al
EPI_ISL_1547553	Furst Medical Laboratory	Norwegian Institute of Public Health, Department of Virology	Kathrine Stene-Johansen et al
EPI_ISL_775398	Nordland Hospital - Bodo, Laboratory Department, Molecular Biology Unit	Norwegian Institute of Public Health, Department of Virology	Kathrine Stene-Johansen et al
EPI_ISL_1547634	Ostfold Hospital Trust - Kalnes, Centre for Laboratory Medicine, Section for gene technology and infection serology	Norwegian Institute of Public Health, Department of Virology	Kathrine Stene-Johansen et al
EPI_ISL_775528	Akershus University Hospital, Department for Microbiology and Infectious Disease Control	Norwegian Institute of Public Health, Department of Virology	Kathrine Stene-Johansen et al

EPI_ISL_775529	Akershus University Hospital, Department for Microbiology and Infectious Disease Control	Norwegian Institute of Public Health, Department of Virology	Kathrine Stene-Johansen et al
EPI_ISL_775530	Akershus University Hospital, Department for Microbiology and Infectious Disease Control	Norwegian Institute of Public Health, Department of Virology	Kathrine Stene-Johansen et al
EPI_ISL_1251304	Akershus University Hospital, Department for Microbiology and Infectious Disease Control	Norwegian Institute of Public Health, Department of Virology	Kathrine Stene-Johansen et al
EPI_ISL_796703	Oslo University Hospital, Department of Medical Microbiology	Norwegian Institute of Public Health, Department of Virology	Kathrine Stene-Johansen et al
EPI_ISL_1251378	University Hospital of Northern Norway, Department for Microbiology and Infectious Disease Control	Norwegian Institute of Public Health, Department of Virology	Kathrine Stene-Johansen et al
EPI_ISL_860233	Norwegian Institute of Public Health, Department of Virology	Norwegian Institute of Public Health, Department of Virology	Kathrine Stene-Johansen et al
EPI_ISL_1599419	Akershus University Hospital, Department for Microbiology and Infectious Disease Control	Norwegian Institute of Public Health, Department of Virology	Kathrine Stene-Johansen et al
EPI_ISL_794602	Central Laboratories, Egyptian Ministry of Health and Population	Central Laboratories, Egyptian Ministry of Health and Population	Kayed et al
EPI_ISL_794600	Central Laboratories, Egyptian Ministry of Health and Population	Central Laboratories, Egyptian Ministry of Health and Population	Kayed et al
EPI_ISL_1335899	Department of Microbiology, Faculty of Medicine, Universitas Sumatera Utara	Faculty of Medicine, Universitas Sumatera Utara; Institute of Tropical Disease, Universitas Airlangga	Kazufumi Shimizu et al
EPI_ISL_770472	National Health laboratory	Botswana Institute for Technology Research and Innovation	Kefentse Arnold Tumedi et al
EPI_ISL_770475	National health Laboratory	Botswana Institute for Technology Research and Innovation	Kefentse Arnold Tumedi et al
EPI_ISL_770470	National Health laboratory	Botswana Institute for Technology Research and Innovation	Kefentse Arnold Tumedi et al
EPI_ISL_560386	National Health Laboratory	Botswana Institute for Technology Research and innovation	Kefentse Arnold Tumedi et al
EPI_ISL_560390	National Health Laboratory	Botswana Institute for Technology Research and Innovation	Kefentse Arnold Tumedi et al
EPI_ISL_560385	National Health Laboratory	Botswana Institute for Technology Research and Innovation	Kefentse Arnold Tumedi et al
EPI_ISL_560387	National Health Laboratory	Botswana Institute for Technology Research and Innovation	Kefentse Arnold Tumedi et al
EPI_ISL_560389	National Health Laboratory	Botswana Institute for Technology Research and Innovation	Kefentse Arnold Tumedi et al
EPI_ISL_560388	National Health Laboratory	Botswana Institute for Technology Research and Innovation	Kefentse Arnold Tumedi et al
EPI_ISL_770474	National Health laboratory	Botswana Institute for Technology Research and Innovation	Kefentse Arnold Tumedi et al
EPI_ISL_427389	Department of Clinical Microbiology	GIGA Medical Genomics	Keith Durkin et al
EPI_ISL_455975	Department of Clinical Microbiology	GIGA Medical Genomics	Keith Durkin et al
EPI_ISL_484708	Department of Clinical Microbiology	GIGA Medical Genomics	Keith Durkin et al
EPI_ISL_540451	University of Liège COVID-19 testing center	GIGA Medical Genomics	Keith Durkin et al
EPI_ISL_540466	University of Liège COVID-19 testing center	GIGA Medical Genomics	Keith Durkin et al
EPI_ISL_581596	Department of Clinical Microbiology	GIGA Medical Genomics	Keith Durkin et al
EPI_ISL_581622	Department of Clinical Microbiology	GIGA Medical Genomics	Keith Durkin et al
EPI_ISL_626279	Department of Clinical Microbiology	GIGA Medical Genomics	Keith Durkin et al

EPI_ISL_626236	Department of Clinical Microbiology	GIGA Medical Genomics	Keith Durkin et al
EPI_ISL_626232	Department of Clinical Microbiology	GIGA Medical Genomics	Keith Durkin et al
EPI_ISL_641597	Department of Clinical Microbiology	GIGA Medical Genomics	Keith Durkin et al
EPI_ISL_661243	Department of Clinical Microbiology	GIGA Medical Genomics	Keith Durkin et al
EPI_ISL_678432	Department of Clinical Microbiology	GIGA Medical Genomics	Keith Durkin et al
EPI_ISL_678452	Department of Clinical Microbiology	GIGA Medical Genomics	Keith Durkin et al
EPI_ISL_707735	Department of Clinical Microbiology	GIGA Medical Genomics	Keith Durkin et al
EPI_ISL_707722	Department of Clinical Microbiology	GIGA Medical Genomics	Keith Durkin et al
EPI_ISL_708455	Department of Clinical Microbiology	GIGA Medical Genomics	Keith Durkin et al
EPI_ISL_708456	Department of Clinical Microbiology	GIGA Medical Genomics	Keith Durkin et al
EPI_ISL_737330	Department of Clinical Microbiology	GIGA Medical Genomics	Keith Durkin et al
EPI_ISL_737372	Department of Clinical Microbiology	GIGA Medical Genomics	Keith Durkin et al
EPI_ISL_737383	Department of Clinical Microbiology	GIGA Medical Genomics	Keith Durkin et al
EPI_ISL_925421	Department of Clinical Microbiology	GIGA Medical Genomics	Keith Durkin et al
EPI_ISL_925470	Department of Clinical Microbiology	GIGA Medical Genomics	Keith Durkin et al
EPI_ISL_925472	Department of Clinical Microbiology	GIGA Medical Genomics	Keith Durkin et al
EPI_ISL_1660775	University of Liège COVID-19 testing center	GIGA Medical Genomics	Keith Durkin et al
EPI_ISL_1660782	University of Liège COVID-19 testing center	GIGA Medical Genomics	Keith Durkin et al
EPI_ISL_848344	Illinois Department of Public Health	Gagnon Lab, Southern Illinois University	Keith Gagnon et al
EPI_ISL_497768	Department of Microbiology, The University of Hong Kong	Department of Microbiology, The University of Hong Kong	Kelvin K.W. To et al
EPI_ISL_497770	Department of Microbiology, The University of Hong Kong	Department of Microbiology, The University of Hong Kong	Kelvin K.W. To et al
EPI_ISL_497789	Department of Microbiology, The University of Hong Kong	Department of Microbiology, The University of Hong Kong	Kelvin K.W. To et al
EPI_ISL_497818	Department of Microbiology, The University of Hong Kong	Department of Microbiology, The University of Hong Kong	Kelvin K.W. To et al
EPI_ISL_497823	Department of Microbiology, The University of Hong Kong	Department of Microbiology, The University of Hong Kong	Kelvin K.W. To et al
EPI_ISL_497851	Department of Microbiology, The University of Hong Kong	Department of Microbiology, The University of Hong Kong	Kelvin K.W. To et al
EPI_ISL_497865	Department of Microbiology, The University of Hong Kong	Department of Microbiology, The University of Hong Kong	Kelvin K.W. To et al
EPI_ISL_498270	Department of Microbiology, The University of Hong Kong	Department of Microbiology, The University of Hong Kong	Kelvin K.W. To et al
EPI_ISL_498271	Department of Microbiology, The University of Hong Kong	Department of Microbiology, The University of Hong Kong	Kelvin K.W. To et al
EPI_ISL_510540	Department of Microbiology, The University of Hong Kong	Department of Microbiology, The University of Hong Kong	Kelvin K.W. To et al
EPI_ISL_1034641	Department of Microbiology, The University of Hong Kong	Department of Microbiology, The University of Hong Kong	Kelvin K.W. To et al

EPI_ISL_1034642	Department of Microbiology, The University of Hong Kong	Department of Microbiology, The University of Hong Kong	Kelvin K.W. To et al
EPI_ISL_1034656	Department of Microbiology, The University of Hong Kong	Department of Microbiology, The University of Hong Kong	Kelvin K.W. To et al
EPI_ISL_1034382	Department of Microbiology, The University of Hong Kong	Department of Microbiology, The University of Hong Kong	Kelvin K.W. To et al
EPI_ISL_1034592	Department of Microbiology, The University of Hong Kong	Department of Microbiology, The University of Hong Kong	Kelvin K.W. To et al
EPI_ISL_1034373	Department of Microbiology, The University of Hong Kong	Department of Microbiology, The University of Hong Kong	Kelvin K.W. To et al
EPI_ISL_1034600	Department of Microbiology, The University of Hong Kong	Department of Microbiology, The University of Hong Kong	Kelvin K.W. To et al
EPI_ISL_1034601	Department of Microbiology, The University of Hong Kong	Department of Microbiology, The University of Hong Kong	Kelvin K.W. To et al
EPI_ISL_1034603	Department of Microbiology, The University of Hong Kong	Department of Microbiology, The University of Hong Kong	Kelvin K.W. To et al
EPI_ISL_1034368	Department of Microbiology, The University of Hong Kong	Department of Microbiology, The University of Hong Kong	Kelvin K.W. To et al
EPI_ISL_1034668	Department of Microbiology, The University of Hong Kong	Department of Microbiology, The University of Hong Kong	Kelvin K.W. To et al
EPI_ISL_1197081	Department of Microbiology, The University of Hong Kong	Department of Microbiology, The University of Hong Kong	Kelvin K.W. To et al
EPI_ISL_1197114	Department of Microbiology, The University of Hong Kong	Department of Microbiology, The University of Hong Kong	Kelvin K.W. To et al
EPI_ISL_1205467	Department of Microbiology, The University of Hong Kong	Department of Microbiology, The University of Hong Kong	Kelvin K.W. To et al
EPI_ISL_1197083	Department of Microbiology, The University of Hong Kong	Department of Microbiology, The University of Hong Kong	Kelvin K.W. To et al
EPI_ISL_1197084	Department of Microbiology, The University of Hong Kong	Department of Microbiology, The University of Hong Kong	Kelvin K.W. To et al
EPI_ISL_1197109	Department of Microbiology, The University of Hong Kong	Department of Microbiology, The University of Hong Kong	Kelvin K.W. To et al
EPI_ISL_1660475	Department of Microbiology, The University of Hong Kong	The University of Hong Kong Department of Microbiology	Kelvin K.W. To et al
EPI_ISL_469291	Keio University Hospital	Keio University Hospital	Kenjiro Kosaki et al
EPI_ISL_469292	Keio University Hospital	Keio University Hospital	Kenjiro Kosaki et al
EPI_ISL_469295	Keio University Hospital	Keio University Hospital	Kenjiro Kosaki et al
EPI_ISL_417179	Department of Pathology, Princess Margaret Hospital	Department of Health Technology and Informatics, Faculty of Health and Social Science, The Hong Kong Polytechnic University	Kenneth Siu-Sing LEUNG et al
EPI_ISL_417183	Department of Clinical Pathology, Pamela Youde Nethersole Eastern Hospital	Department of Health Technology and Informatics, Faculty of Health and Social Science, The Hong Kong Polytechnic University	Kenneth Siu-Sing LEUNG et al
EPI_ISL_417177	Department of Pathology, Princess Margaret Hospital	Department of Health Technology and Informatics, Faculty of Health and Social Science, The Hong Kong Polytechnic University	Kenneth Siu-Sing LEUNG et al
EPI_ISL_419244	Department of Clinical Pathology, Pamela Youde Nethersole Eastern Hospital	Department of Health Technology and Informatics, Faculty of Health and Social Science, The Hong Kong Polytechnic University	Kenneth Siu-Sing LEUNG et al
EPI_ISL_419245	Department of Clinical Pathology, Pamela Youde Nethersole Eastern Hospital	Department of Health Technology and Informatics, Faculty of Health and Social Science, The Hong Kong Polytechnic University	Kenneth Siu-Sing LEUNG et al
EPI_ISL_419246	Department of Clinical Pathology, Pamela Youde Nethersole Eastern Hospital	Department of Health Technology and Informatics, Faculty of Health and Social Science, The Hong Kong Polytechnic University	Kenneth Siu-Sing LEUNG et al
EPI_ISL_419248	Department of Clinical Pathology, Pamela Youde Nethersole Eastern Hospital	Department of Health Technology and Informatics, Faculty of Health and Social Science, The Hong Kong Polytechnic University	Kenneth Siu-Sing LEUNG et al

EPI_ISL_419249	Department of Clinical Pathology, Pamela Youde Nethersole Eastern Hospital	Department of Health Technology and Informatics, Faculty of Health and Social Science, The Hong Kong Polytechnic University	Kenneth Siu-Sing LEUNG et al
EPI_ISL_419251	Department of Clinical Pathology, Pamela Youde Nethersole Eastern Hospital	Department of Health Technology and Informatics, Faculty of Health and Social Science, The Hong Kong Polytechnic University	Kenneth Siu-Sing LEUNG et al
EPI_ISL_419252	Department of Clinical Pathology, Pamela Youde Nethersole Eastern Hospital	Department of Health Technology and Informatics, Faculty of Health and Social Science, The Hong Kong Polytechnic University	Kenneth Siu-Sing LEUNG et al
EPI_ISL_419253	Department of Pathology, United Christian Hospital	Department of Health Technology and Informatics, Faculty of Health and Social Science, The Hong Kong Polytechnic University	Kenneth Siu-Sing LEUNG et al
EPI_ISL_605907	NGS Lab, DNA SOLUTION LTD.	NGS Lab, DNA SOLUTION LTD.	Khan et al
EPI_ISL_605908	NGS Lab, DNA SOLUTION LTD.	NGS Lab, DNA SOLUTION LTD.	Khan et al
EPI_ISL_513925	Microbiology & Bioinformatics and Biostatistics, Kohat University of Science and Technology (Pakistan) & Shanghai Jiao Tong University (China)	Microbiology & Bioinformatics and Biostatistics, Kohat University of Science and Technology (Pakistan) & Shanghai Jiao Tong University (China)	Khan et al
EPI_ISL_896284	SUNY UPSTATE MEDICAL UNIVERSITY	Wadsworth Center, New York State Department of Health	Kirsten St. George et al
EPI_ISL_1228374	WESTCHESTER MEDICAL CENTER	Wadsworth Center, New York State Department of Health	Kirsten St. George et al
EPI_ISL_754411	Wadsworth Center, New York State Department.of Health	Wadsworth Center, New York State Department.of Health	Kirsten St. George et al
EPI_ISL_1265406	1. Wojewódzka Stacja Sanitarno - Epidemiologiczna w Katowicach (WSSE Katowice); 2. Wojewódzka Stacja Sanitarno - Epidemiologiczna w Kielcach (WSSE Kielce); 3. Wojewódzka Stacja Sanitarno - Epidemiologiczna w Szczecinie (WSSE Szczecin); 4. Wojewódzka Stacja Sanitarno - Epidemiologiczna w Lublinie (WSSE Lublin); 5. Wojewódzka Stacja Sanitarno - Epidemiologiczna w Gorzowie Wielkopolskim (WSSE Gorzów Wielkopolski); 6. Wojewódzka Stacja Sanitarno-Epidemiologiczna w Gdańsku (WSSE Gdańsk); 7. Wojewódzka Stacja Sanitarno-Epidemiologiczna w Poznaniu (WSSE Poznań); 8. Wojewódzka Stacja Sanitarno-Epidemiologiczna w Opolu (WSSE Opole); 9. Wojewódzka Stacja Sanitarno-Epidemiologiczna we Wrocławiu (WSSE Wrocław); 10. Wojewódzka Stacja Sanitarno-Epidemiologiczna w Krakowie (WSSE Kraków); 11. Wojewódzka Stacja Sanitarno-Epidemiologiczna w Olsztynie (WSSE Olsztyn); 12. Wojewódzka Stacja Sanitarno-Epidemiologiczna w Bydgoszczy (WSSE Bydgoszcz); 13. Wojewódzka Stacja Sanitarno-Epidemiologiczna w Rzeszowie (WSSE Rzeszów); 14. Wojewódzka Stacja Sanitarno-Epidemiologiczna w Białymstoku (WSSE Białystok);	1. ViroGenetics - BSL3 Laboratory of Virology, Małopolska Centre of Biotechnology, Jagiellonian University; 2. Human Genome Variation Research Group, Malopolska Centre of Biotechnology, Jagiellonian University;	Klajmon et al
EPI_ISL_1265440	1. Wojewódzka Stacja Sanitarno - Epidemiologiczna w Katowicach (WSSE Katowice); 2. Wojewódzka Stacja Sanitarno - Epidemiologiczna w Kielcach (WSSE Kielce); 3. Wojewódzka Stacja Sanitarno - Epidemiologiczna w Szczecinie (WSSE Szczecin); 4. Wojewódzka Stacja Sanitarno - Epidemiologiczna w Lublinie (WSSE Lublin); 5. Wojewódzka Stacja Sanitarno - Epidemiologiczna w Gorzowie Wielkopolskim (WSSE Gorzów Wielkopolski); 6. Wojewódzka Stacja Sanitarno-Epidemiologiczna w Gdańsku (WSSE Gdańsk); 7. Wojewódzka Stacja Sanitarno-Epidemiologiczna w Poznaniu (WSSE Poznań); 8. Wojewódzka Stacja Sanitarno-Epidemiologiczna w Opolu (WSSE Opole); 9. Wojewódzka Stacja Sanitarno-Epidemiologiczna we Wrocławiu (WSSE Wrocław); 10. Wojewódzka Stacja Sanitarno-Epidemiologiczna w Krakowie (WSSE Kraków); 11. Wojewódzka Stacja Sanitarno-Epidemiologiczna w Olsztynie (WSSE Olsztyn); 12. Wojewódzka Stacja Sanitarno-Epidemiologiczna w Bydgoszczy (WSSE Bydgoszcz); 13. Wojewódzka Stacja Sanitarno-Epidemiologiczna w Rzeszowie (WSSE Rzeszów); 14. Wojewódzka Stacja Sanitarno-Epidemiologiczna w Białymstoku (WSSE Białystok);	1. ViroGenetics - BSL3 Laboratory of Virology, Małopolska Centre of Biotechnology, Jagiellonian University; 2. Human Genome Variation Research Group, Malopolska Centre of Biotechnology, Jagiellonian University;	Klajmon et al

EPI_ISL_1654841	Laboratory Batajnica	Institute of microbiology and Immunology, Faculty of Medicine, University of Belgrade	Knezevic et al
EPI_ISL_1017680	Institute of Virology, Vaccines and Sera "Torlak"	Institute of microbiology and Immunology, Faculty of Medicine, University of Belgrade	Knezevic et al
EPI_ISL_1654811	Institute of Virology, Vaccines and Sera Torlak	Institute of microbiology and Immunology, Faculty of Medicine, University of Belgrade	Knezevic et al
EPI_ISL_1654815	Institute of Virology, Vaccines and Sera Torlak	Institute of microbiology and Immunology, Faculty of Medicine, University of Belgrade	Knezevic et al
EPI_ISL_1017691	Institute of Virology, Vaccines and Sera "Torlak"	Institute of microbiology and Immunology, Faculty of Medicine, University of Belgrade	Knezevic et al
EPI_ISL_1017679	Institute of Virology, Vaccines and Sera "Torlak"	Institute of microbiology and Immunology, Faculty of Medicine, University of Belgrade	Knezevic et al
EPI_ISL_1017688	Institute of Virology, Vaccines and Sera "Torlak"	Institute of microbiology and Immunology, Faculty of Medicine, University of Belgrade	Knezevic et al
EPI_ISL_1017689	Institute of Virology, Vaccines and Sera "Torlak"	Institute of microbiology and Immunology, Faculty of Medicine, University of Belgrade	Knezevic et al
EPI_ISL_1017681	Institute of Virology, Vaccines and Sera "Torlak"	Institute of microbiology and Immunology, Faculty of Medicine, University of Belgrade	Knezevic et al
EPI_ISL_1017699	Institute of Virology, Vaccines and Sera "Torlak"	Institute of microbiology and Immunology, Faculty of Medicine, University of Belgrade	Knezevic et al
EPI_ISL_1017700	Institute of Virology, Vaccines and Sera "Torlak"	Institute of microbiology and Immunology, Faculty of Medicine, University of Belgrade	Knezevic et al
EPI_ISL_1654817	Institute of Virology, Vaccines and Sera Torlak	Institute of microbiology and Immunology, Faculty of Medicine, University of Belgrade	Knezevic et al
EPI_ISL_1654818	Institute of Virology, Vaccines and Sera Torlak	Institute of microbiology and Immunology, Faculty of Medicine, University of Belgrade	Knezevic et al
EPI_ISL_1654809	Institute of Virology, Vaccines and Sera Torlak	Institute of microbiology and Immunology, Faculty of Medicine, University of Belgrade	Knezevic et al
EPI_ISL_1654813	Institute of Virology, Vaccines and Sera Torlak	Institute of microbiology and Immunology, Faculty of Medicine, University of Belgrade	Knezevic et al
EPI_ISL_1654837	Institute of Virology, Vaccines and Sera Torlak	Institute of microbiology and Immunology, Faculty of Medicine, University of Belgrade	Knezevic et al
EPI_ISL_1654835	Institute of Virology, Vaccines and Sera Torlak	Institute of microbiology and Immunology, Faculty of Medicine, University of Belgrade	Knezevic et al
EPI_ISL_955160	Institute for Biocides and Medical Ecology	Institute of microbiology and Immunology, Faculty of Medicine, University of Belgrade	Knezevic et al
EPI_ISL_955166	Institute for Biocides and Medical Ecology	Institute of microbiology and Immunology, Faculty of Medicine, University of Belgrade	Knezevic et al
EPI_ISL_955167	Institute for Biocides and Medical Ecology	Institute of microbiology and Immunology, Faculty of Medicine, University of Belgrade	Knezevic et al
EPI_ISL_1654842	Laboratory for COVID19 diagnostics, Clinical Centre of Serbia	Institute of microbiology and Immunology, Faculty of Medicine, University of Belgrade	Knezevic et al
EPI_ISL_586542	Government General Hospital, Jam Khambhaliya, Devbhoomi Dwarka	Gujarat Biotechnology Research Centre	Komal Patel et al

EPI_ISL_487446	CICM-Mali	Bundeswehr Institut of Microbiology	Kouriba et al
EPI_ISL_487449	CICM-Mali	Bundeswehr Institut of Microbiology	Kouriba et al
EPI_ISL_487450	CICM-Mali	Bundeswehr Institut of Microbiology	Kouriba et al
EPI_ISL_487452	CICM-Mali	Bundeswehr Institut of Microbiology	Kouriba et al
EPI_ISL_487454	CICM-Mali	Bundeswehr Institut of Microbiology	Kouriba et al
EPI_ISL_487455	CICM-Mali	Bundeswehr Institut of Microbiology	Kouriba et al
EPI_ISL_487461	CICM-Mali	Bundeswehr Institut of Microbiology	Kouriba et al
EPI_ISL_487463	CICM-Mali	Bundeswehr Institut of Microbiology	Kouriba et al
EPI_ISL_645011	Human Genome Variation Research Group, Malopolska Centre of Biotechnology	Human Genome Variation Research Group, Malopolska Centre of Biotechnology	Kowalski et al
EPI_ISL_645030	Human Genome Variation Research Group, Malopolska Centre of Biotechnology	Human Genome Variation Research Group, Malopolska Centre of Biotechnology	Kowalski et al
EPI_ISL_645049	Human Genome Variation Research Group, Malopolska Centre of Biotechnology	Human Genome Variation Research Group, Malopolska Centre of Biotechnology	Kowalski et al
EPI_ISL_645056	Human Genome Variation Research Group, Malopolska Centre of Biotechnology	Human Genome Variation Research Group, Malopolska Centre of Biotechnology	Kowalski et al
EPI_ISL_645057	Human Genome Variation Research Group, Malopolska Centre of Biotechnology	Human Genome Variation Research Group, Malopolska Centre of Biotechnology	Kowalski et al
EPI_ISL_645060	Human Genome Variation Research Group, Malopolska Centre of Biotechnology	Human Genome Variation Research Group, Malopolska Centre of Biotechnology	Kowalski et al
EPI_ISL_645061	Human Genome Variation Research Group, Malopolska Centre of Biotechnology	Human Genome Variation Research Group, Malopolska Centre of Biotechnology	Kowalski et al
EPI_ISL_645062	Human Genome Variation Research Group, Malopolska Centre of Biotechnology	Human Genome Variation Research Group, Malopolska Centre of Biotechnology	Kowalski et al
EPI_ISL_645063	Human Genome Variation Research Group, Malopolska Centre of Biotechnology	Human Genome Variation Research Group, Malopolska Centre of Biotechnology	Kowalski et al
EPI_ISL_645064	Human Genome Variation Research Group, Malopolska Centre of Biotechnology	Human Genome Variation Research Group, Malopolska Centre of Biotechnology	Kowalski et al
EPI_ISL_645065	Human Genome Variation Research Group, Malopolska Centre of Biotechnology	Human Genome Variation Research Group, Malopolska Centre of Biotechnology	Kowalski et al
EPI_ISL_645068	Human Genome Variation Research Group, Malopolska Centre of Biotechnology	Human Genome Variation Research Group, Malopolska Centre of Biotechnology	Kowalski et al
EPI_ISL_645084	Human Genome Variation Research Group, Malopolska Centre of Biotechnology	Human Genome Variation Research Group, Malopolska Centre of Biotechnology	Kowalski et al
EPI_ISL_645085	Human Genome Variation Research Group, Malopolska Centre of Biotechnology	Human Genome Variation Research Group, Malopolska Centre of Biotechnology	Kowalski et al
EPI_ISL_678279	Mikrobiologie, RARI	Mikrobiologie, RARI	Krasnov et al
EPI_ISL_678288	Mikrobiologie, RARI	Mikrobiologie, RARI	Krasnov et al
EPI_ISL_678273	Mikrobiologie, RARI	Mikrobiologie, RARI	Krasnov et al

EPI_ISL_877227	Thai Red Cross Emerging Infectious Diseases Health Science Centre, Chulalongkorn Hospital, Faculty of Medicine, Chulalongkorn University	Thai Red Cross Emerging Infectious Diseases Center and Faculty of Medicine, Chulalongkorn University	Krirk Asavametha et al
EPI_ISL_759959	Premier Hospital Surabaya	Institute of Tropical Disease, Universitas Airlangga	Krisnoadi Rahardjo et al
EPI_ISL_1072986	Department of Microbiology, Faculty of Medicine, Universitas Sumatera Utara	Institute of Tropical Disease, Universitas Airlangga; Faculty of Medicine, Universitas Sumatera Utara	Krisnoadi Rahardjo et al
EPI_ISL_1273049	Guam Public Health Laboratory	Centers for Disease Control and Prevention Division of Viral Diseases, Pathogen Discovery	Krista Queen et al
EPI_ISL_1273073	Guam Public Health Laboratory	Centers for Disease Control and Prevention Division of Viral Diseases, Pathogen Discovery	Krista Queen et al
EPI_ISL_1273057	Guam Public Health Laboratory	Centers for Disease Control and Prevention Division of Viral Diseases, Pathogen Discovery	Krista Queen et al
EPI_ISL_1273069	Guam Public Health Laboratory	Centers for Disease Control and Prevention Division of Viral Diseases, Pathogen Discovery	Krista Queen et al
EPI_ISL_1273052	Guam Public Health Laboratory	Centers for Disease Control and Prevention Division of Viral Diseases, Pathogen Discovery	Krista Queen et al
EPI_ISL_1273072	Guam Public Health Laboratory	Centers for Disease Control and Prevention Division of Viral Diseases, Pathogen Discovery	Krista Queen et al
EPI_ISL_1273050	Guam Public Health Laboratory	Centers for Disease Control and Prevention Division of Viral Diseases, Pathogen Discovery	Krista Queen et al
EPI_ISL_1273062	Guam Public Health Laboratory	Centers for Disease Control and Prevention Division of Viral Diseases, Pathogen Discovery	Krista Queen et al
EPI_ISL_1273059	Guam Public Health Laboratory	Centers for Disease Control and Prevention Division of Viral Diseases, Pathogen Discovery	Krista Queen et al
EPI_ISL_1273067	Guam Public Health Laboratory	Centers for Disease Control and Prevention Division of Viral Diseases, Pathogen Discovery	Krista Queen et al
EPI_ISL_1273068	Guam Public Health Laboratory	Centers for Disease Control and Prevention Division of Viral Diseases, Pathogen Discovery	Krista Queen et al
EPI_ISL_1273053	Guam Public Health Laboratory	Centers for Disease Control and Prevention Division of Viral Diseases, Pathogen Discovery	Krista Queen et al
EPI_ISL_1273048	Guam Public Health Laboratory	Centers for Disease Control and Prevention Division of Viral Diseases, Pathogen Discovery	Krista Queen et al
EPI_ISL_1273064	Guam Public Health Laboratory	Centers for Disease Control and Prevention Division of Viral Diseases, Pathogen Discovery	Krista Queen et al
EPI_ISL_1273054	Guam Public Health Laboratory	Centers for Disease Control and Prevention Division of Viral Diseases, Pathogen Discovery	Krista Queen et al
EPI_ISL_1273071	Guam Public Health Laboratory	Centers for Disease Control and Prevention Division of Viral Diseases, Pathogen Discovery	Krista Queen et al
EPI_ISL_1273063	Guam Public Health Laboratory	Centers for Disease Control and Prevention Division of Viral Diseases, Pathogen Discovery	Krista Queen et al
EPI_ISL_1273056	Guam Public Health Laboratory	Centers for Disease Control and Prevention Division of Viral Diseases, Pathogen Discovery	Krista Queen et al
EPI_ISL_1273061	Guam Public Health Laboratory	Centers for Disease Control and Prevention Division of Viral Diseases, Pathogen Discovery	Krista Queen et al

EPI_ISL_450792	Jamaica Ministry of Health and Wellness	Pathogen Discovery, Respiratory Viruses Branch, Division of Viral Diseases, Centers for Disease Control and Prevention	Krista Queen et al
EPI_ISL_450798	Jamaica Ministry of Health and Wellness	Pathogen Discovery, Respiratory Viruses Branch, Division of Viral Diseases, Centers for Disease Control and Prevention	Krista Queen et al
EPI_ISL_408008	California Department of Health	Pathogen Discovery, Respiratory Viruses Branch, Division of Viral Diseases, Centers for Disease Control and Prevention	Krista Queen et al
EPI_ISL_408009	California Department of Health	Pathogen Discovery, Respiratory Viruses Branch, Division of Viral Diseases, Centers for Disease Control and Prevention	Krista Queen et al
EPI_ISL_411954	California Department of Public Health	Pathogen Discovery, Respiratory Viruses Branch, Division of Viral Diseases, Centers for Disease Control and Prevention	Krista Queen et al
EPI_ISL_959618	Institute of Virology, Biomedical Research Center of the Slovak Academy of Sciences, Bratislava	Faculty of Natural Sciences, Comenius University, Bratislava	Kristína Boršová et al
EPI_ISL_1239407	Institute of Virology, Biomedical Research Center of the Slovak Academy of Sciences, Bratislava	Faculty of Natural Sciences, Comenius University, Bratislava	Kristína Boršová et al
EPI_ISL_577737	Institute of Virology, Biomedical Research Center of the Slovak Academy of Sciences, Bratislava	Faculty of Natural Sciences, Comenius University, Bratislava	Kristína Boršová et al
EPI_ISL_577739	Institute of Virology, Biomedical Research Center of the Slovak Academy of Sciences, Bratislava	Faculty of Natural Sciences, Comenius University, Bratislava	Kristína Boršová et al
EPI_ISL_718250	Institute of Virology, Biomedical Research Center of the Slovak Academy of Sciences, Bratislava	Faculty of Natural Sciences, Comenius University, Bratislava	Kristína Boršová et al
EPI_ISL_485389	University of Ulsan College of Medicine and Asan Medical Center	University of Ulsan College of Medicine and Asan Medical Center	Kuenyoul Park et al
EPI_ISL_485390	University of Ulsan College of Medicine and Asan Medical Center	University of Ulsan College of Medicine and Asan Medical Center	Kuenyoul Park et al
EPI_ISL_485391	University of Ulsan College of Medicine and Asan Medical Center	University of Ulsan College of Medicine and Asan Medical Center	Kuenyoul Park et al
EPI_ISL_485393	University of Ulsan College of Medicine and Asan Medical Center	University of Ulsan College of Medicine and Asan Medical Center	Kuenyoul Park et al
EPI_ISL_485395	University of Ulsan College of Medicine and Asan Medical Center	University of Ulsan College of Medicine and Asan Medical Center	Kuenyoul Park et al
EPI_ISL_485396	University of Ulsan College of Medicine and Asan Medical Center	University of Ulsan College of Medicine and Asan Medical Center	Kuenyoul Park et al
EPI_ISL_930843	Virology, ICAR-National Research Centre on Equines	Virology, ICAR-National Research Centre on Equines	Kumar et al
EPI_ISL_760059	Laboratory of Genetics and Personalized Medicine, Zan Mitrev Clinic	Laboratory of Genetics and Personalized Medicine, Zan Mitrev Clinic	Kungulovski et al.
EPI_ISL_760246	Laboratory of Genetics and Personalized Medicine, Zan Mitrev Clinic	Laboratory of Genetics and Personalized Medicine, Zan Mitrev Clinic	Kungulovski et al.
EPI_ISL_1201428	Laboratory of Genetics and Personalized Medicine, Zan Mitrev Clinic	Laboratory of Genetics and Personalized Medicine, Zan Mitrev Clinic	Kungulovski G. et al.
EPI_ISL_1201429	Laboratory of Genetics and Personalized Medicine, Zan Mitrev Clinic	Laboratory of Genetics and Personalized Medicine, Zan Mitrev Clinic	Kungulovski G. et al.
EPI_ISL_1201433	Laboratory of Genetics and Personalized Medicine, Zan Mitrev Clinic	Laboratory of Genetics and Personalized Medicine, Zan Mitrev Clinic	Kungulovski G. et al.
EPI_ISL_1096341	Laboratory of Genetics and Personalized Medicine, Zan Mitrev Clinic	Laboratory of Genetics and Personalized Medicine, Zan Mitrev Clinic	Kungulovski G. et al.
EPI_ISL_415741	Laboratory Medicine	Department of Laboratory Medicine, Lin-Kou Chang Gung Memorial Hospital, Taoyuan, Taiwan	Kuo-Chien Tsao et al
EPI_ISL_415742	Laboratory Medicine	Department of Laboratory Medicine, Lin-Kou Chang Gung Memorial Hospital, Taoyuan, Taiwan	Kuo-Chien Tsao et al

EPI_ISL_415743	Laboratory Medicine	Department of Laboratory Medicine, Lin-Kou Chang Gung Memorial Hospital, Taoyuan, Taiwan	Kuo-Chien Tsao et al
EPI_ISL_444274	Laboratory Medicine	Department of Laboratory Medicine, Lin-Kou Chang Gung Memorial Hospital, Taoyuan, Taiwan	Kuo-Chien Tsao et al
EPI_ISL_452178	Laboratory Medicine	Department of Laboratory Medicine, Lin-Kou Chang Gung Memorial Hospital, Taoyuan, Taiwan	Kuo-Chien Tsao et al
EPI_ISL_452179	Laboratory Medicine	Department of Laboratory Medicine, Lin-Kou Chang Gung Memorial Hospital, Taoyuan, Taiwan	Kuo-Chien Tsao et al
EPI_ISL_464093	Laboratory Medicine	Department of Laboratory Medicine, Lin-Kou Chang Gung Memorial Hospital, Taoyuan, Taiwan	Kuo-Chien Tsao et al
EPI_ISL_660543	Laboratory Medicine	Department of Laboratory Medicine, Lin-Kou Chang Gung Memorial Hospital, Taoyuan, Taiwan	Kuo-Chien Tsao et al
EPI_ISL_660544	Laboratory Medicine	Department of Laboratory Medicine, Lin-Kou Chang Gung Memorial Hospital, Taoyuan, Taiwan	Kuo-Chien Tsao et al
EPI_ISL_660545	Laboratory Medicine	Department of Laboratory Medicine, Lin-Kou Chang Gung Memorial Hospital, Taoyuan, Taiwan	Kuo-Chien Tsao et al
EPI_ISL_667809	Laboratory Medicine	Department of Laboratory Medicine, Lin-Kou Chang Gung Memorial Hospital, Taoyuan, Taiwan	Kuo-Chien Tsao et al
EPI_ISL_956317	Laboratory Medicine	Department of Laboratory Medicine, Lin-Kou Chang Gung Memorial Hospital, Taoyuan, Taiwan	Kuo-Chien Tsao et al
EPI_ISL_956318	Laboratory Medicine	Department of Laboratory Medicine, Lin-Kou Chang Gung Memorial Hospital, Taoyuan, Taiwan	Kuo-Chien Tsao et al
EPI_ISL_956320	Laboratory Medicine	Department of Laboratory Medicine, Lin-Kou Chang Gung Memorial Hospital, Taoyuan, Taiwan	Kuo-Chien Tsao et al
EPI_ISL_956322	Laboratory Medicine	Department of Laboratory Medicine, Lin-Kou Chang Gung Memorial Hospital, Taoyuan, Taiwan	Kuo-Chien Tsao et al
EPI_ISL_956323	Laboratory Medicine	Department of Laboratory Medicine, Lin-Kou Chang Gung Memorial Hospital, Taoyuan, Taiwan	Kuo-Chien Tsao et al
EPI_ISL_956324	Laboratory Medicine	Department of Laboratory Medicine, Lin-Kou Chang Gung Memorial Hospital, Taoyuan, Taiwan	Kuo-Chien Tsao et al
EPI_ISL_956325	Laboratory Medicine	Department of Laboratory Medicine, Lin-Kou Chang Gung Memorial Hospital, Taoyuan, Taiwan	Kuo-Chien Tsao et al
EPI_ISL_956326	Laboratory Medicine	Department of Laboratory Medicine, Lin-Kou Chang Gung Memorial Hospital, Taoyuan, Taiwan	Kuo-Chien Tsao et al
EPI_ISL_956331	Laboratory Medicine	Department of Laboratory Medicine, Lin-Kou Chang Gung Memorial Hospital, Taoyuan, Taiwan	Kuo-Chien Tsao et al
EPI_ISL_1669972	Laboratory of virology and molecular diagnostics, Institute of Public Health	Laboratory of virology and molecular diagnostics, Institute of Public Health	Kuzmanovska M et al
EPI_ISL_1669992	Laboratory of virology and molecular diagnostics, Institute of Public Health	Laboratory of virology and molecular diagnostics, Institute of Public Health	Kuzmanovska M et al
EPI_ISL_1669993	Laboratory of virology and molecular diagnostics, Institute of Public Health	Laboratory of virology and molecular diagnostics, Institute of Public Health	Kuzmanovska M et al
EPI_ISL_1191759	Laboratory of virology and molecular diagnostics	Laboratory of virology and molecular diagnostics	Kuzmanovska Maja et al
EPI_ISL_1180157	Laboratory of virology and molecular diagnostics	Laboratory of virology and molecular diagnostics	Kuzmanovska Maja et al
EPI_ISL_1180159	Laboratory of virology and molecular diagnostics	Laboratory of virology and molecular diagnostics	Kuzmanovska Maja et al

EPI_ISL_1180153	Laboratory of virology and molecular diagnostics	Laboratory of virology and molecular diagnostics	Kuzmanovska Maja et al
EPI_ISL_648124	UHAS COVID-19 Lab	UHAS COVID-19 Lab	Kwabena O. Duedu et al
EPI_ISL_648125	UHAS COVID-19 Lab	UHAS COVID-19 Lab	Kwabena O. Duedu et al
EPI_ISL_628761	UHAS COVID-19 Lab	UHAS COVID-19 Lab	Kwabena O. Duedu et al
EPI_ISL_1503159	National Public Health Organization	National Public Health Organization	Kyriaki Tryfinopoulou et al
EPI_ISL_1503160	National Public Health Organization	National Public Health Organization	Kyriaki Tryfinopoulou et al
EPI_ISL_1630201	Central Public Health Lab, National Public Health Organization	Central Public Health Lab, National Public Health Organization	Kyriaki Tryfinopoulou et al
EPI_ISL_1497476	National Public Health Organization	National Public Health Organization	Kyriaki Tryfinopoulou et al
EPI_ISL_1497478	National Public Health Organization	National Public Health Organization	Kyriaki Tryfinopoulou et al
EPI_ISL_1497327	National Public Health Organization	National Public Health Organization	Kyriaki Tryfinopoulou et al
EPI_ISL_1497491	National Public Health Organization	National Public Health Organization	Kyriaki Tryfinopoulou et al
EPI_ISL_1497492	National Public Health Organization	National Public Health Organization	Kyriaki Tryfinopoulou et al
EPI_ISL_1497493	National Public Health Organization	National Public Health Organization	Kyriaki Tryfinopoulou et al
EPI_ISL_1497494	National Public Health Organization	National Public Health Organization	Kyriaki Tryfinopoulou et al
EPI_ISL_1497495	National Public Health Organization	National Public Health Organization	Kyriaki Tryfinopoulou et al
EPI_ISL_1497496	National Public Health Organization	National Public Health Organization	Kyriaki Tryfinopoulou et al
EPI_ISL_1497497	National Public Health Organization	National Public Health Organization	Kyriaki Tryfinopoulou et al
EPI_ISL_1497498	National Public Health Organization	National Public Health Organization	Kyriaki Tryfinopoulou et al
EPI_ISL_1497477	National Public Health Organization	National Public Health Organization	Kyriaki Tryfinopoulou et al
EPI_ISL_1497290	National Public Health Organization	National Public Health Organization	Kyriaki Tryfinopoulou et al
EPI_ISL_1048453	National Health Laboratory Service, South Africa	KRISP, KZn Research Innovation and Sequencing Platform	Laguda-Akingba O et al
EPI_ISL_1366838	National Health Laboratory Service, South Africa	KRISP, KZN Research Innovation and Sequencing Platform	Laguda-Akingba O et al
EPI_ISL_528833	CSIR-Centre for Cellular and Molecular Biology	CSIR-Centre for Cellular and Molecular Biology	Lamuk Zaveri et al
EPI_ISL_751486	CHU Purpan - Laboratoire de Virologie - Institut Fédératif de Biologie	CHU Purpan - Laboratoire de Virologie - Institut Fédératif de Biologie	Latour J. et al
EPI_ISL_415457	University Hospitals of Geneva Laboratory of Virology	University Hospitals of Geneva Laboratory of Virology	Laubscher F. et al
EPI_ISL_415700	University Hospitals of Geneva Laboratory of Virology	University Hospitals of Geneva Laboratory of Virology	Laubscher F. et al
EPI_ISL_415454	University Hospitals of Geneva Laboratory of Virology	University Hospitals of Geneva Laboratory of Virology	Laubscher F. et al
EPI_ISL_429203	University Hospitals of Geneva Laboratory of Virology	University Hospitals of Geneva Laboratory of Virology	Laubscher F. et al
EPI_ISL_413999	Laboratoire de Virologie, HUG	Swiss National Reference Centre for Influenza	LAUBSCHER Florian et al.
EPI_ISL_414019	Laboratoire de Virologie, HUG	Swiss National Reference Centre for Influenza	LAUBSCHER Florian et al.

EPI_ISL_414020	Laboratoire de Virologie, HUG	Swiss National Reference Centre for Influenza	LAUBSCHER Florian et al.
EPI_ISL_413996	Laboratoire de Virologie, HUG	Swiss National Reference Centre for Influenza	LAUBSCHER Florian et al.
EPI_ISL_510116	Hospital General Universitario Gregorio Marañón	SeqCOVID-SPAIN consortium/IBV(CSIC)	Laura Pérez-Lago et al
EPI_ISL_600492	Institute of Epidemiology Disease Control And Research	Institute for Developing Science and Health Initiatives	Lauren Cowley et al
EPI_ISL_600498	Institute of Epidemiology Disease Control And Research	Institute for Developing Science and Health Initiatives	Lauren Cowley et al
EPI_ISL_600512	Institute of Epidemiology Disease Control And Research	Institute for Developing Science and Health Initiatives	Lauren Cowley et al
EPI_ISL_600562	Institute of Epidemiology Disease Control And Research	Institute for Developing Science and Health Initiatives	Lauren Cowley et al
EPI_ISL_451172	Lab voor klinische biologie	Onderzoeksgroep Virologie	Laurens Lambrechts et al
EPI_ISL_940821	INSPI-CRN de Influenza y otros virus respiratorios	INSPI-Centro de Investigación Multidisciplinaria de la DTIDI	Leandro Patiño et al
EPI_ISL_940775	INSPI-CRN de Influenza y otros virus respiratorios	INSPI-Centro de Investigación Multidisciplinaria de la DTIDI	Leandro Patiño et al
EPI_ISL_940769	INSPI-CRN de Influenza y otros virus respiratorios	INSPI-Centro de Investigación Multidisciplinaria de la DTIDI	Leandro Patiño et al
EPI_ISL_806544	INSPI Instituto Nacional de Investigación en Salud Pública	Av. Julián Coronel 905 entre Esmeraldas y José Mascote Av. Juan Tanca Marengo No. 100 y Av. de las Américas	Leandro Patiño et al
EPI_ISL_574292	Instituto Nacional de Investigación en Salud Pública	Instituto Nacional de Investigación en Salud Pública	Leandro Patino Patino et al
EPI_ISL_574294	Instituto Nacional de Investigacion en Salud Pública	Instituto Nacional de Investigación en Salud Pública	Leandro Patino Patino et al
EPI_ISL_574293	Instituto Nacional de Investigación en Salud Pública	Instituto Nacional de Investigación en Salud Publica	Leandro Patino Patino et al
EPI_ISL_1663230	Fimlab Laboratories	Fimlab Laboratories	Leena Huhti et al
EPI_ISL_1663231	Fimlab Laboratories	Fimlab Laboratories	Leena Huhti et al
EPI_ISL_1663233	Fimlab Laboratories	Fimlab Laboratories	Leena Huhti et al
EPI_ISL_875545	Instituto de Biotecnologia - UNESP-Botucatu-SP	Instituto de Biotecnologia - UNESP-Botucatu-SP	Leila Sabrina Ullmann et al
EPI_ISL_875542	Instituto de Biotecnologia - UNESP-Botucatu-SP	Instituto de Biotecnologia - UNESP-Botucatu-SP	Leila Sabrina Ullmann et al
EPI_ISL_875547	Instituto de Biotecnologia - UNESP-Botucatu-SP	Instituto de Biotecnologia - UNESP-Botucatu-SP	Leila Sabrina Ullmann et al
EPI_ISL_861242	Instituto de Biotecnologia - UNESP-Botucatu-SP	Instituto de Biotecnologia - UNESP-Botucatu-SP	Leila Sabrina Ullmann et al
EPI_ISL_765890	Massachusetts General Hospital	Infectious Disease Program, Broad Institute of Harvard and MIT	Lemieux et al
EPI_ISL_1629785	Laboratorio de Referencia Nacional de Virus Respiratorios, Instituto Nacional de Salud Peru	Laboratorio de Genómica Microbiana, Universidad Peruana Cayetano Heredia	Lenin Maturrano et al
EPI_ISL_1629787	Laboratorio de Referencia Nacional de Virus Respiratorios, Instituto Nacional de Salud Peru	Laboratorio de Genómica Microbiana, Universidad Peruana Cayetano Heredia	Lenin Maturrano et al
EPI_ISL_1629792	Instituto de Medicina Tropical Alexander Von Humboldt, Universidad Peruana Cayetano Heredia	Laboratorio de Genómica Microbiana, Universidad Peruana Cayetano Heredia	Lenin Maturrano et al
EPI_ISL_1629793	Instituto de Medicina Tropical Alexander Von Humboldt, Universidad Peruana Cayetano Heredia	Laboratorio de Genómica Microbiana, Universidad Peruana Cayetano Heredia	Lenin Maturrano et al
EPI_ISL_1629794	Instituto de Medicina Tropical Alexander Von Humboldt, Universidad Peruana Cayetano Heredia	Laboratorio de Genómica Microbiana, Universidad Peruana Cayetano Heredia	Lenin Maturrano et al

EPI_ISL_1629795	Instituto de Medicina Tropical Alexander Von Humboldt, Universidad Peruana Cayetano Heredia	Laboratorio de Genómica Microbiana, Universidad Peruana Cayetano Heredia	Lenin Maturrano et al
EPI_ISL_1629796	Instituto de Medicina Tropical Alexander Von Humboldt, Universidad Peruana Cayetano Heredia	Laboratorio de Genómica Microbiana, Universidad Peruana Cayetano Heredia	Lenin Maturrano et al
EPI_ISL_1629798	Instituto de Medicina Tropical Alexander Von Humboldt, Universidad Peruana Cayetano Heredia	Laboratorio de Genómica Microbiana, Universidad Peruana Cayetano Heredia	Lenin Maturrano et al
EPI_ISL_1629799	Instituto de Medicina Tropical Alexander Von Humboldt, Universidad Peruana Cayetano Heredia	Laboratorio de Genómica Microbiana, Universidad Peruana Cayetano Heredia	Lenin Maturrano et al
EPI_ISL_1629800	Instituto de Medicina Tropical Alexander Von Humboldt, Universidad Peruana Cayetano Heredia	Laboratorio de Genómica Microbiana, Universidad Peruana Cayetano Heredia	Lenin Maturrano et al
EPI_ISL_1629801	Instituto de Medicina Tropical Alexander Von Humboldt, Universidad Peruana Cayetano Heredia	Laboratorio de Genómica Microbiana, Universidad Peruana Cayetano Heredia	Lenin Maturrano et al
EPI_ISL_1629802	Instituto de Medicina Tropical Alexander Von Humboldt, Universidad Peruana Cayetano Heredia	Laboratorio de Genómica Microbiana, Universidad Peruana Cayetano Heredia	Lenin Maturrano et al
EPI_ISL_1629803	Instituto de Medicina Tropical Alexander Von Humboldt, Universidad Peruana Cayetano Heredia	Laboratorio de Genómica Microbiana, Universidad Peruana Cayetano Heredia	Lenin Maturrano et al
EPI_ISL_1629804	Instituto de Medicina Tropical Alexander Von Humboldt, Universidad Peruana Cayetano Heredia	Laboratorio de Genómica Microbiana, Universidad Peruana Cayetano Heredia	Lenin Maturrano et al
EPI_ISL_1629805	Instituto de Medicina Tropical Alexander Von Humboldt, Universidad Peruana Cayetano Heredia	Laboratorio de Genómica Microbiana, Universidad Peruana Cayetano Heredia	Lenin Maturrano et al
EPI_ISL_1629806	Instituto de Medicina Tropical Alexander Von Humboldt, Universidad Peruana Cayetano Heredia	Laboratorio de Genómica Microbiana, Universidad Peruana Cayetano Heredia	Lenin Maturrano et al
EPI_ISL_1629807	Instituto de Medicina Tropical Alexander Von Humboldt, Universidad Peruana Cayetano Heredia	Laboratorio de Genómica Microbiana, Universidad Peruana Cayetano Heredia	Lenin Maturrano et al
EPI_ISL_801559	Laboratory of Molecular Virology, Pontificia Universidad Católica de Chile	MSHS Pathogen Surveillance Program	Leonardo I. Almonacid et al
EPI_ISL_801566	Laboratory of Molecular Virology, Pontificia Universidad Católica de Chile	MSHS Pathogen Surveillance Program	Leonardo I. Almonacid et al
EPI_ISL_801579	Laboratory of Molecular Virology, Pontificia Universidad Católica de Chile	MSHS Pathogen Surveillance Program	Leonardo I. Almonacid et al
EPI_ISL_801549	Laboratory of Molecular Virology, Pontificia Universidad Católica de Chile	MSHS Pathogen Surveillance Program	Leonardo I. Almonacid et al
EPI_ISL_801606	Laboratory of Molecular Virology, Pontificia Universidad Católica de Chile	MSHS Pathogen Surveillance Program	Leonardo I. Almonacid et al
EPI_ISL_801607	Laboratory of Molecular Virology, Pontificia Universidad Católica de Chile	MSHS Pathogen Surveillance Program	Leonardo I. Almonacid et al
EPI_ISL_801612	Laboratory of Molecular Virology, Pontificia Universidad Católica de Chile	MSHS Pathogen Surveillance Program	Leonardo I. Almonacid et al
EPI_ISL_801613	Laboratory of Molecular Virology, Pontificia Universidad Católica de Chile	MSHS Pathogen Surveillance Program	Leonardo I. Almonacid et al
EPI_ISL_801647	Laboratory of Molecular Virology, Pontificia Universidad Católica de Chile	MSHS Pathogen Surveillance Program	Leonardo I. Almonacid et al
EPI_ISL_801663	Laboratory of Molecular Virology, Pontificia Universidad Católica de Chile	MSHS Pathogen Surveillance Program	Leonardo I. Almonacid et al
EPI_ISL_801731	Laboratory of Molecular Virology, Pontificia Universidad Católica de Chile	MSHS Pathogen Surveillance Program	Leonardo I. Almonacid et al
EPI_ISL_801735	Laboratory of Molecular Virology, Pontificia Universidad Católica de Chile	MSHS Pathogen Surveillance Program	Leonardo I. Almonacid et al
EPI_ISL_801816	Laboratory of Molecular Virology, Pontificia Universidad Católica de Chile	MSHS Pathogen Surveillance Program	Leonardo I. Almonacid et al
EPI_ISL_801848	Laboratory of Molecular Virology, Pontificia Universidad Católica de Chile	MSHS Pathogen Surveillance Program	Leonardo I. Almonacid et al

EPI_ISL_801853	Laboratory of Molecular Virology, Pontificia Universidad Católica de Chile	MSHS Pathogen Surveillance Program	Leonardo I. Almonacid et al
EPI_ISL_801593	Laboratory of Molecular Virology, Pontificia Universidad Católica de Chile	MSHS Pathogen Surveillance Program	Leonardo I. Almonacid et al
EPI_ISL_985395	National Institute of Infectious Diseases-Prof. Dr. Matei Bals Molecular Diagnostics Laboratory	National Institute of Infectious Diseases-Prof. Dr. Matei Bals Molecular Diagnostics Laboratory	Leontina Banica et al
EPI_ISL_979544	National Institute of Infectious Diseases-Prof. Dr. Matei Bals Molecular Diagnostics Laboratory	National Institute of Infectious Diseases-Prof. Dr. Matei Bals Molecular Diagnostics Laboratory	Leontina Banica et al
EPI_ISL_885154	National Institute of Infectious Diseases-Prof. Dr. Matei Bals Molecular Diagnostics Laboratory	National Institute of Infectious Diseases-Prof. Dr. Matei Bals Molecular Diagnostics Laboratory	Leontina Banica et al
EPI_ISL_979959	National Institute of Infectious Diseases-Prof. Dr. Matei Bals Molecular Diagnostics Laboratory	National Institute of Infectious Diseases-Prof. Dr. Matei Bals Molecular Diagnostics Laboratory	Leontina Banica et al
EPI_ISL_1116445	National Institute of Infectious Diseases-Prof. Dr. Matei Bals Molecular Diagnostics Laboratory	National Institute of Infectious Diseases-Prof. Dr. Matei Bals Molecular Diagnostics Laboratory	Leontina Banica et al
EPI_ISL_1116446	National Institute of Infectious Diseases-Prof. Dr. Matei Bals Molecular Diagnostics Laboratory	National Institute of Infectious Diseases-Prof. Dr. Matei Bals Molecular Diagnostics Laboratory	Leontina Banica et al
EPI_ISL_1184825	National Institute of Infectious Diseases-Prof. Dr. Matei Bals Molecular Diagnostics Laboratory	National Institute of Infectious Diseases-Prof. Dr. Matei Bals Molecular Diagnostics Laboratory	Leontina Banica et al
EPI_ISL_1184826	National Institute of Infectious Diseases-Prof. Dr. Matei Bals Molecular Diagnostics Laboratory	National Institute of Infectious Diseases-Prof. Dr. Matei Bals Molecular Diagnostics Laboratory	Leontina Banica et al
EPI_ISL_1279947	National Institute of Infectious Diseases-Prof. Dr. Matei Bals Molecular Diagnostics Laboratory	National Institute of Infectious Diseases-Prof. Dr. Matei Bals Molecular Diagnostics Laboratory	Leontina Banica et al
EPI_ISL_1279949	National Institute of Infectious Diseases-Prof. Dr. Matei Bals Molecular Diagnostics Laboratory	National Institute of Infectious Diseases-Prof. Dr. Matei Bals Molecular Diagnostics Laboratory	Leontina Banica et al
EPI_ISL_1279953	National Institute of Infectious Diseases-Prof. Dr. Matei Bals Molecular Diagnostics Laboratory	National Institute of Infectious Diseases-Prof. Dr. Matei Bals Molecular Diagnostics Laboratory	Leontina Banica et al
EPI_ISL_1279956	National Institute of Infectious Diseases-Prof. Dr. Matei Bals Molecular Diagnostics Laboratory	National Institute of Infectious Diseases-Prof. Dr. Matei Bals Molecular Diagnostics Laboratory	Leontina Banica et al
EPI_ISL_1279959	National Institute of Infectious Diseases-Prof. Dr. Matei Bals Molecular Diagnostics Laboratory	National Institute of Infectious Diseases-Prof. Dr. Matei Bals Molecular Diagnostics Laboratory	Leontina Banica et al
EPI_ISL_1279965	National Institute of Infectious Diseases-Prof. Dr. Matei Bals Molecular Diagnostics Laboratory	National Institute of Infectious Diseases-Prof. Dr. Matei Bals Molecular Diagnostics Laboratory	Leontina Banica et al
EPI_ISL_1279966	National Institute of Infectious Diseases-Prof. Dr. Matei Bals Molecular Diagnostics Laboratory	National Institute of Infectious Diseases-Prof. Dr. Matei Bals Molecular Diagnostics Laboratory	Leontina Banica et al
EPI_ISL_468136	[Romania, Bucharest] National Institute for Infectious Diseases “Prof. Dr. Matei Balș”	[Romania, Bucharest] National Institute for Infectious Diseases “Prof. Dr. Matei Balș”	Leontina Banica et al
EPI_ISL_1279960	National Institute of Infectious Diseases-Prof. Dr. Matei Bals Molecular Diagnostics Laboratory	National Institute of Infectious Diseases-Prof. Dr. Matei Bals Molecular Diagnostics Laboratory	Leontina Banica et al
EPI_ISL_1116447	National Institute of Infectious Diseases-Prof. Dr. Matei Bals Molecular Diagnostics Laboratory	National Institute of Infectious Diseases-Prof. Dr. Matei Bals Molecular Diagnostics Laboratory	Leontina Banica et al
EPI_ISL_862836	National Institute of Infectious Diseases-Prof. Dr. Matei Bals Molecular Diagnostics Laboratory	National Institute of Infectious Diseases-Prof. Dr. Matei Bals Molecular Diagnostics Laboratory	Leontina Banica et al
EPI_ISL_468137	[Romania, Bucharest] National Institute for Infectious Diseases “Prof. Dr. Matei Balș”	[Romania, Bucharest] National Institute for Infectious Diseases “Prof. Dr. Matei Balș”	Leontina Banica et al

EPI_ISL_468139	[Romania, Bucharest] National Institute for Infectious Diseases “Prof. Dr. Matei Balș”	[Romania, Bucharest] National Institute for Infectious Diseases “Prof. Dr. Matei Balș”	Leontina Banica et al
EPI_ISL_468141	[Romania, Bucharest] National Institute for Infectious Diseases “Prof. Dr. Matei Balș”	[Romania, Bucharest] National Institute for Infectious Diseases “Prof. Dr. Matei Balș”	Leontina Banica et al
EPI_ISL_468143	[Romania, Bucharest] National Institute for Infectious Diseases “Prof. Dr. Matei Balș”	[Romania, Bucharest] National Institute for Infectious Diseases “Prof. Dr. Matei Balș”	Leontina Banica et al
EPI_ISL_682298	Respiratory virus Laboratory, Chinese Academy of Medical Science	Respiratory virus Laboratory, Chinese Academy of Medical Science	Li et al
EPI_ISL_417420	Jiangxi province Center for Disease Control and Prevention	Jiangxi province Center for Disease Control and Prevention	Li jian Xiong et al
EPI_ISL_498226	LIC	LIC	LIC et al
EPI_ISL_1098895	Laboratory of Communicable Diseasea	Laboratory of Communicable Diseasea	Liidia Dotsenko et al
EPI_ISL_1138533	Laboratory of Communicable Diseases	1. Laboratory of Communicable Diseases (Estonia); 2. Eurofins Genomics Europe Sequencing GmbH	Liidia Dotsenko et al
EPI_ISL_1138531	Laboratory of Communicable Diseases	1. Laboratory of Communicable Diseases (Estonia); 2. Eurofins Genomics Europe Sequencing GmbH	Liidia Dotsenko et al
EPI_ISL_1138512	SA Pärnu Hospital Laboratory	1. Laboratory of Communicable Diseases (Estonia); 2. Eurofins Genomics Europe Sequencing GmbH	Liidia Dotsenko et al
EPI_ISL_1138513	SA Pärnu Hospital Laboratory	1. Laboratory of Communicable Diseases (Estonia); 2. Eurofins Genomics Europe Sequencing GmbH	Liidia Dotsenko et al
EPI_ISL_1138514	SA Pärnu Hospital Laboratory	1. Laboratory of Communicable Diseases (Estonia); 2. Eurofins Genomics Europe Sequencing GmbH	Liidia Dotsenko et al
EPI_ISL_1138515	SA Pärnu Hospital Laboratory	1. Laboratory of Communicable Diseases (Estonia); 2. Eurofins Genomics Europe Sequencing GmbH	Liidia Dotsenko et al
EPI_ISL_1259062	Synlab Eesti OÜ	1. Laboratory of Communicable Diseases (Estonia); 2. Eurofins Genomics Europe Sequencing GmbH	Liidia Dotsenko et al.
EPI_ISL_1470225	Synlab Eesti OÜ	1. Laboratory of Communicable Diseases (Estonia); 2. Eurofins Genomics Europe Sequencing GmbH	Liidia Dotsenko et al.
EPI_ISL_1470151	Synlab Eesti OÜ	1. Laboratory of Communicable Diseases (Estonia); 2. Eurofins Genomics Europe Sequencing GmbH	Liidia Dotsenko et al.
EPI_ISL_1319299	Synlab Eesti OÜ	1. Laboratory of Communicable Diseases (Estonia); 2. Eurofins Genomics Europe Sequencing GmbH	Liidia Dotsenko et al.
EPI_ISL_1259086	Synlab Eesti OÜ	1. Laboratory of Communicable Diseases (Estonia); 2. Eurofins Genomics Europe Sequencing GmbH	Liidia Dotsenko et al.
EPI_ISL_1470026	Synlab Eesti OÜ	1. Laboratory of Communicable Diseases (Estonia); 2. Eurofins Genomics Europe Sequencing GmbH	Liidia Dotsenko et al.
EPI_ISL_1319321	Synlab Eesti OÜ	1. Laboratory of Communicable Diseases (Estonia); 2. Eurofins Genomics Europe Sequencing GmbH	Liidia Dotsenko et al.
EPI_ISL_1470138	Synlab Eesti OÜ	1. Laboratory of Communicable Diseases (Estonia); 2. Eurofins Genomics Europe Sequencing GmbH	Liidia Dotsenko et al.
EPI_ISL_1470134	North Estonia Medical Centre laboratory	1. Laboratory of Communicable Diseases (Estonia); 2. Eurofins Genomics Europe Sequencing GmbH	Liidia Dotsenko et al.
EPI_ISL_1470176	Synlab Eesti OÜ	1. Laboratory of Communicable Diseases (Estonia); 2. Eurofins Genomics Europe Sequencing GmbH	Liidia Dotsenko et al.

EPI_ISL_1470177	Synlab Eesti OÜ	1. Laboratory of Communicable Diseases (Estonia); 2. Eurofins Genomics Europe Sequencing GmbH	Liidia Dotsenko et al.
EPI_ISL_1470189	Synlab Eesti OÜ	1. Laboratory of Communicable Diseases (Estonia); 2. Eurofins Genomics Europe Sequencing GmbH	Liidia Dotsenko et al.
EPI_ISL_1470197	Synlab Eesti OÜ	1. Laboratory of Communicable Diseases (Estonia); 2. Eurofins Genomics Europe Sequencing GmbH	Liidia Dotsenko et al.
EPI_ISL_1470287	Synlab Eesti OÜ	1. Laboratory of Communicable Diseases (Estonia); 2. Eurofins Genomics Europe Sequencing GmbH	Liidia Dotsenko et al.
EPI_ISL_1259026	North Estonia Medical Centre laboratory	1. Laboratory of Communicable Diseases (Estonia); 2. Eurofins Genomics Europe Sequencing GmbH	Liidia Dotsenko et al.
EPI_ISL_431180	Fujian Center for Disease Control and Prevention	Fujian Center for Disease Control and Prevention	Lin Qi et al
EPI_ISL_431240	Fujian Center for Disease Control and Prevention	Fujian Center for Disease Control and Prevention	Lin Qi et al
EPI_ISL_452236	VC Sorgenfrimottagningen	The Public Health Agency of Sweden	Lisa Esbjornsson Klemendz et al
EPI_ISL_450816	orestadsklinikens VC	The Public Health Agency of Sweden	Lisa Kjellberg / Laura Plavitu et al
EPI_ISL_445241	Ã–restadsklinikens VC	The Public Health Agency of Sweden	Lisa Kjellberg / Laura Plavitu et al
EPI_ISL_678397	Suceava County Emergency Hospital	Stefan cel Mare University Metagenomics Lab	Lobiuc Andrei et al
EPI_ISL_486834	Suceava County Energency Hospital Sf. Ioan cel Nou	SMU Metagenomics lab	Lobiuc Andrei et al
EPI_ISL_491036	Suceava County Emergency Hospital	Stefan cel Mare University Metagenomics Lab	Lobiuc Andrei et al
EPI_ISL_491038	Suceava County Emergency Hospital	Stefan cel Mare University Metagenomics Lab	Lobiuc Andrei et al
EPI_ISL_491059	Suceava County Emergency Hospital	Stefan cel Mare University Metagenomics Lab	Lobiuc Andrei et al
EPI_ISL_896374	University of Medicine and Pharmacy of Craiova	Stefan cel Mare University Metagenomics Lab	Lobiuc Andrei et al
EPI_ISL_896375	University of Medicine and Pharmacy of Craiova	Stefan cel Mare University Metagenomics Lab	Lobiuc Andrei et al
EPI_ISL_891227	The Oncology Institute Prof. Dr. Ion Chiricuta Cluj Napoca	Stefan cel Mare University Metagenomics Lab	Lobiuc Andrei et al
EPI_ISL_891230	The Oncology Institute Prof. Dr. Ion Chiricuta Cluj Napoca	Stefan cel Mare University Metagenomics Lab	Lobiuc Andrei et al
EPI_ISL_891224	The Oncology Institute Prof. Dr. Ion Chiricuta Cluj Napoca	Stefan cel Mare University Metagenomics Lab	Lobiuc Andrei et al
EPI_ISL_891226	The Oncology Institute Prof. Dr. Ion Chiricuta Cluj Napoca	Stefan cel Mare University Metagenomics Lab	Lobiuc Andrei et al
EPI_ISL_891225	The Oncology Institute Prof. Dr. Ion Chiricuta Cluj Napoca	Stefan cel Mare University Metagenomics Lab	Lobiuc Andrei et al
EPI_ISL_486855	Emergency county Hospital Suceava	Stefan cel Mare University Metagenomics Lab	Lobiuc Andrei et al.
EPI_ISL_491039	Suceava County Emergency Hospital	Stefan cel Mare University Metagenomics Lab	Lobiuc Andrei et al.
EPI_ISL_491048	Suceava County Emergency Hospital	Stefan cel Mare University Metagenomics Lab	Lobiuc Andrei et al.
EPI_ISL_491051	Suceava County Emergency Hospital	Stefan cel Mare University Metagenomics Lab	Lobiuc Andrei et al.
EPI_ISL_491052	Suceava County Emergency Hospital	Stefan cel Mare University Metagenomics Lab	Lobiuc Andrei et al.
EPI_ISL_491055	Suceava County Emergency Hospital	Stefan cel Mare University Metagenomics Lab	Lobiuc Andrei et al.

EPI_ISL_491074	Suceava County Emergency Hospital	Stefan cel Mare University Metagenomics Lab	Lobiuc Andrei et al.
EPI_ISL_491089	Suceava County Emergency Hospital	Stefan cel Mare University Metagenomics Lab	Lobiuc Andrei et al.
EPI_ISL_444998	Naval Health Research Center	Naval Medical Research Center Biological Defense Research Directorate	Logan Voegtly et al
EPI_ISL_444999	Naval Health Research Center	Naval Medical Research Center Biological Defense Research Directorate	Logan Voegtly et al
EPI_ISL_445000	Naval Health Research Center	Naval Medical Research Center Biological Defense Research Directorate	Logan Voegtly et al
EPI_ISL_849656	Servizio Igiene Epidemiologia e Sanità Pubblica (SIESP)-L'Aquila	Istitituto Zooprofilattico Sperimentale dell'Abruzzo e Molise G.Caporale	Lorusso A et al
EPI_ISL_849657	Servizio Igiene Epidemiologia e Sanità Pubblica (SIESP)-L'Aquila	Istitituto Zooprofilattico Sperimentale dell'Abruzzo e Molise G.Caporale	Lorusso A et al
EPI_ISL_849658	Servizio Igiene Epidemiologia e Sanità Pubblica (SIESP)-L'Aquila	Istitituto Zooprofilattico Sperimentale dell'Abruzzo e Molise G.Caporale	Lorusso A et al
EPI_ISL_849659	Servizio Igiene Epidemiologia e Sanità Pubblica (SIESP)-L'Aquila	Istitituto Zooprofilattico Sperimentale dell'Abruzzo e Molise G.Caporale	Lorusso A et al
EPI_ISL_849660	Servizio Igiene Epidemiologia e Sanità Pubblica (SIESP)-L'Aquila	Istitituto Zooprofilattico Sperimentale dell'Abruzzo e Molise G.Caporale	Lorusso A et al
EPI_ISL_875566	SIESP L'AQUILA	Istituto Zooprofilattico Sperimentale dell’Abruzzo e Molise “G. Caporale”	Lorusso A et al
EPI_ISL_875567	SIESP L'AQUILA	Istituto Zooprofilattico Sperimentale dell’Abruzzo e Molise “G. Caporale”	Lorusso A et al
EPI_ISL_875568	SIESP L'AQUILA	Istituto Zooprofilattico Sperimentale dell’Abruzzo e Molise “G. Caporale”	Lorusso A et al
EPI_ISL_528934	Agenzia di Tutela della Salute di Bergamo	Istitituto Zooprofilattico Sperimentale dell'Abruzzo e Molise G.Caporale	Lorusso A et al
EPI_ISL_528942	Agenzia di Tutela della Salute di Bergamo	Istitituto Zooprofilattico Sperimentale dell'Abruzzo e Molise G.Caporale	Lorusso A et al
EPI_ISL_528945	Agenzia di Tutela della Salute di Bergamo	Istitituto Zooprofilattico Sperimentale dell'Abruzzo e Molise G.Caporale	Lorusso A et al
EPI_ISL_528946	Agenzia di Tutela della Salute di Bergamo	Istitituto Zooprofilattico Sperimentale dell'Abruzzo e Molise G.Caporale	Lorusso A et al
EPI_ISL_514131	Rondônia Central Public Health Laboratory (LACEN/RO), vinculated to State Health Secretariat of Rondônia (SESAU/RO)	Molecular Virology Laboratory of Oswaldo Cruz Foundation of Rondônia	Luan Felipo Botelho-Souza et al
EPI_ISL_514132	Rondônia Central Public Health Laboratory (LACEN/RO), vinculated to State Health Secretariat of Rondônia (SESAU/RO)	Molecular Virology Laboratory of Oswaldo Cruz Foundation of Rondônia	Luan Felipo Botelho-Souza et al
EPI_ISL_514133	Rondônia Central Public Health Laboratory (LACEN/RO), vinculated to State Health Secretariat of Rondônia (SESAU/RO)	Molecular Virology Laboratory of Oswaldo Cruz Foundation of Rondônia	Luan Felipo Botelho-Souza et al
EPI_ISL_514134	Rondônia Central Public Health Laboratory (LACEN/RO), vinculated to State Health Secretariat of Rondônia (SESAU/RO)	Molecular Virology Laboratory of Oswaldo Cruz Foundation of Rondônia	Luan Felipo Botelho-Souza et al
EPI_ISL_514135	Rondônia Central Public Health Laboratory (LACEN/RO), vinculated to State Health Secretariat of Rondônia (SESAU/RO)	Molecular Virology Laboratory of Oswaldo Cruz Foundation of Rondônia	Luan Felipo Botelho-Souza et al
EPI_ISL_514136	Rondônia Central Public Health Laboratory (LACEN/RO), vinculated to State Health Secretariat of Rondônia (SESAU/RO)	Molecular Virology Laboratory of Oswaldo Cruz Foundation of Rondônia	Luan Felipo Botelho-Souza et al
EPI_ISL_514137	Rondônia Central Public Health Laboratory (LACEN/RO), vinculated to State Health Secretariat of Rondônia (SESAU/RO)	Molecular Virology Laboratory of Oswaldo Cruz Foundation of Rondônia	Luan Felipo Botelho-Souza et al
EPI_ISL_514138	Rondônia Central Public Health Laboratory (LACEN/RO), vinculated to State Health Secretariat of Rondônia (SESAU/RO)	Molecular Virology Laboratory of Oswaldo Cruz Foundation of Rondônia	Luan Felipo Botelho-Souza et al
EPI_ISL_1626618	Laboratorio de Salud Pública Bogota	Gencore - Universidad de los Andes	Luisa Sacristan et al
EPI_ISL_1626621	Laboratorio de Salud Pública Bogota	Gencore - Universidad de los Andes	Luisa Sacristan et al

EPI_ISL_1626619	Laboratorio de Salud Pública Bogota	Gencore - Universidad de los Andes	Luisa Sacristan et al
EPI_ISL_1626615	Gencore - Universidad de los Andes	Gencore - Universidad de los Andes	Luisa Sacristan et al
EPI_ISL_1626613	Gencore - Universidad de los Andes	Gencore - Universidad de los Andes	Luisa Sacristan et al
EPI_ISL_1626616	Gencore - Universidad de los Andes	Gencore - Universidad de los Andes	Luisa Sacristan et al
EPI_ISL_1626611	Gencore - Universidad de los Andes	Gencore - Universidad de los Andes	Luisa Sacristan et al
EPI_ISL_1664115	Laboratorio Central Noel Nutels	Bioinformatics Laboratory / LNCC	Luiz G P de Almeida et al
EPI_ISL_1664116	Laboratorio Central Noel Nutels	Bioinformatics Laboratory / LNCC	Luiz G P de Almeida et al
EPI_ISL_1664121	Laboratorio Central Noel Nutels	Bioinformatics Laboratory / LNCC	Luiz G P de Almeida et al
EPI_ISL_1664123	Laboratorio Central Noel Nutels	Bioinformatics Laboratory / LNCC	Luiz G P de Almeida et al
EPI_ISL_1664156	Laboratorio Central Noel Nutels	Bioinformatics Laboratory / LNCC	Luiz G P de Almeida et al
EPI_ISL_1664157	Laboratorio Central Noel Nutels	Bioinformatics Laboratory / LNCC	Luiz G P de Almeida et al
EPI_ISL_1664160	Laboratorio Central Noel Nutels	Bioinformatics Laboratory / LNCC	Luiz G P de Almeida et al
EPI_ISL_1664164	Laboratorio Central Noel Nutels	Bioinformatics Laboratory / LNCC	Luiz G P de Almeida et al
EPI_ISL_1664165	Laboratorio Central Noel Nutels	Bioinformatics Laboratory / LNCC	Luiz G P de Almeida et al
EPI_ISL_1664166	Laboratorio Central Noel Nutels	Bioinformatics Laboratory / LNCC	Luiz G P de Almeida et al
EPI_ISL_1664167	Laboratorio Central Noel Nutels	Bioinformatics Laboratory / LNCC	Luiz G P de Almeida et al
EPI_ISL_1664168	Laboratorio Central Noel Nutels	Bioinformatics Laboratory / LNCC	Luiz G P de Almeida et al
EPI_ISL_1664169	Laboratorio Central Noel Nutels	Bioinformatics Laboratory / LNCC	Luiz G P de Almeida et al
EPI_ISL_1664170	Laboratorio Central Noel Nutels	Bioinformatics Laboratory / LNCC	Luiz G P de Almeida et al
EPI_ISL_1664171	Laboratorio Central Noel Nutels	Bioinformatics Laboratory / LNCC	Luiz G P de Almeida et al
EPI_ISL_1664172	Laboratorio Central Noel Nutels	Bioinformatics Laboratory / LNCC	Luiz G P de Almeida et al
EPI_ISL_1664173	Laboratorio Central Noel Nutels	Bioinformatics Laboratory / LNCC	Luiz G P de Almeida et al
EPI_ISL_1664180	Laboratorio Central Noel Nutels	Bioinformatics Laboratory / LNCC	Luiz G P de Almeida et al
EPI_ISL_1664191	Laboratorio Central Noel Nutels	Bioinformatics Laboratory / LNCC	Luiz G P de Almeida et al
EPI_ISL_1664199	Laboratorio Central Noel Nutels	Bioinformatics Laboratory / LNCC	Luiz G P de Almeida et al
EPI_ISL_910339	Laboratory for Respiratory Viruses, Cantacuzino National Military-Medical Institute for Research and Development	Cantacuzino Institute Virology	Luiza Ustea et al
EPI_ISL_853819	Department of Pathology, Landeskrankenhaus Graz II, Medical University Graz	Bergthaler laboratory, CeMM Research Center for Molecular Medicine of the Austrian Academy of Sciences	Lukas Endler et al
EPI_ISL_853886	Center for Virology, Medical University of Vienna	Bergthaler laboratory, CeMM Research Center for Molecular Medicine of the Austrian Academy of Sciences	Lukas Endler et al
EPI_ISL_853836	Center for Virology, Medical University of Vienna	Bergthaler laboratory, CeMM Research Center for Molecular Medicine of the Austrian Academy of Sciences	Lukas Endler et al

EPI_ISL_853837	Center for Virology, Medical University of Vienna	Bergthaler laboratory, CeMM Research Center for Molecular Medicine of the Austrian Academy of Sciences	Lukas Endler et al
EPI_ISL_853840	Center for Virology, Medical University of Vienna	Bergthaler laboratory, CeMM Research Center for Molecular Medicine of the Austrian Academy of Sciences	Lukas Endler et al
EPI_ISL_853751	Center for Virology, Medical University of Vienna	Bergthaler laboratory, CeMM Research Center for Molecular Medicine of the Austrian Academy of Sciences	Lukas Endler et al
EPI_ISL_853892	Center for Virology, Medical University of Vienna	Bergthaler laboratory, CeMM Research Center for Molecular Medicine of the Austrian Academy of Sciences	Lukas Endler et al
EPI_ISL_853893	Center for Virology, Medical University of Vienna	Bergthaler laboratory, CeMM Research Center for Molecular Medicine of the Austrian Academy of Sciences	Lukas Endler et al
EPI_ISL_853897	Center for Virology, Medical University of Vienna	Bergthaler laboratory, CeMM Research Center for Molecular Medicine of the Austrian Academy of Sciences	Lukas Endler et al
EPI_ISL_853899	Center for Virology, Medical University of Vienna	Bergthaler laboratory, CeMM Research Center for Molecular Medicine of the Austrian Academy of Sciences	Lukas Endler et al
EPI_ISL_853849	Center for Virology, Medical University of Vienna	Bergthaler laboratory, CeMM Research Center for Molecular Medicine of the Austrian Academy of Sciences	Lukas Endler et al
EPI_ISL_853911	Center for Virology, Medical University of Vienna	Bergthaler laboratory, CeMM Research Center for Molecular Medicine of the Austrian Academy of Sciences	Lukas Endler et al
EPI_ISL_853979	Austrian Agency for Health and Food Safety (AGES)	Bergthaler laboratory, CeMM Research Center for Molecular Medicine of the Austrian Academy of Sciences	Lukas Endler et al
EPI_ISL_854277	Austrian Agency for Health and Food Safety (AGES)	Bergthaler laboratory, CeMM Research Center for Molecular Medicine of the Austrian Academy of Sciences	Lukas Endler et al
EPI_ISL_853977	Austrian Agency for Health and Food Safety (AGES)	Bergthaler laboratory, CeMM Research Center for Molecular Medicine of the Austrian Academy of Sciences	Lukas Endler et al
EPI_ISL_1495157	Department of Laboratory Medicine, Division of Clinical Virology, University of Medicine, Vienna	Bergthaler laboratory, CeMM Research Center for Molecular Medicine of the Austrian Academy of Sciences	Lukas Endler et al
EPI_ISL_1495159	Department of Laboratory Medicine, Division of Clinical Virology, University of Medicine, Vienna	Bergthaler laboratory, CeMM Research Center for Molecular Medicine of the Austrian Academy of Sciences	Lukas Endler et al
EPI_ISL_1495160	Department of Laboratory Medicine, Division of Clinical Virology, University of Medicine, Vienna	Bergthaler laboratory, CeMM Research Center for Molecular Medicine of the Austrian Academy of Sciences	Lukas Endler et al
EPI_ISL_1495161	Department of Laboratory Medicine, Division of Clinical Virology, University of Medicine, Vienna	Bergthaler laboratory, CeMM Research Center for Molecular Medicine of the Austrian Academy of Sciences	Lukas Endler et al
EPI_ISL_1495162	Department of Laboratory Medicine, Division of Clinical Virology, University of Medicine, Vienna	Bergthaler laboratory, CeMM Research Center for Molecular Medicine of the Austrian Academy of Sciences	Lukas Endler et al
EPI_ISL_1495164	Department of Laboratory Medicine, Division of Clinical Virology, University of Medicine, Vienna	Bergthaler laboratory, CeMM Research Center for Molecular Medicine of the Austrian Academy of Sciences	Lukas Endler et al
EPI_ISL_1008372	Department of Laboratory Medicine, Division of Clinical Virology, University of Medicine, Vienna	Bergthaler laboratory, CeMM Research Center for Molecular Medicine of the Austrian Academy of Sciences	Lukas Endler et al
EPI_ISL_1008373	Department of Laboratory Medicine, Division of Clinical Virology, University of Medicine, Vienna	Bergthaler laboratory, CeMM Research Center for Molecular Medicine of the Austrian Academy of Sciences	Lukas Endler et al
EPI_ISL_934434	Austrian Agency for Health and Food Safety (AGES)	Bergthaler laboratory, CeMM Research Center for Molecular Medicine of the Austrian Academy of Sciences	Lukas Endler et al
EPI_ISL_1008182	Austrian Agency for Health and Food Safety (AGES)	Bergthaler laboratory, CeMM Research Center for Molecular Medicine of the Austrian Academy of Sciences	Lukas Endler et al

EPI_ISL_1008185	Austrian Agency for Health and Food Safety (AGES)	Bergthaler laboratory, CeMM Research Center for Molecular Medicine of the Austrian Academy of Sciences	Lukas Endler et al
EPI_ISL_934667	Department of Laboratory Medicine, Division of Clinical Virology, University of Medicine, Vienna	Bergthaler laboratory, CeMM Research Center for Molecular Medicine of the Austrian Academy of Sciences	Lukas Endler et al
EPI_ISL_934669	Department of Laboratory Medicine, Division of Clinical Virology, University of Medicine, Vienna	Bergthaler laboratory, CeMM Research Center for Molecular Medicine of the Austrian Academy of Sciences	Lukas Endler et al
EPI_ISL_1008235	Department of Laboratory Medicine, Division of Clinical Virology, University of Medicine, Vienna	Bergthaler laboratory, CeMM Research Center for Molecular Medicine of the Austrian Academy of Sciences	Lukas Endler et al
EPI_ISL_1008258	Department of Laboratory Medicine, Division of Clinical Virology, University of Medicine, Vienna	Bergthaler laboratory, CeMM Research Center for Molecular Medicine of the Austrian Academy of Sciences	Lukas Endler et al
EPI_ISL_1008343	Department of Laboratory Medicine, Division of Clinical Virology, University of Medicine, Vienna	Bergthaler laboratory, CeMM Research Center for Molecular Medicine of the Austrian Academy of Sciences	Lukas Endler et al
EPI_ISL_1180828	Austrian Agency for Health and Food Safety (AGES)	Bergthaler laboratory, CeMM Research Center for Molecular Medicine of the Austrian Academy of Sciences	Lukas Endler et al
EPI_ISL_1118072	Elling group, Institute of Molecular Biotechnology (IMBA)	Bergthaler laboratory, CeMM Research Center for Molecular Medicine of the Austrian Academy of Sciences	Lukas Endler et al
EPI_ISL_1117865	Austrian Agency for Health and Food Safety (AGES)	Bergthaler laboratory, CeMM Research Center for Molecular Medicine of the Austrian Academy of Sciences	Lukas Endler et al
EPI_ISL_1180860	Department of Laboratory Medicine, Division of Clinical Virology, University of Medicine, Vienna	Bergthaler laboratory, CeMM Research Center for Molecular Medicine of the Austrian Academy of Sciences	Lukas Endler et al
EPI_ISL_1180878	Department of Laboratory Medicine, Division of Clinical Virology, University of Medicine, Vienna	Bergthaler laboratory, CeMM Research Center for Molecular Medicine of the Austrian Academy of Sciences	Lukas Endler et al
EPI_ISL_1495283	Institute for Laboratory Diagnostics and Microbiology, Klinikum Klagenfurt am Wörthersee	Bergthaler laboratory, CeMM Research Center for Molecular Medicine of the Austrian Academy of Sciences	Lukas Endler et al
EPI_ISL_1495300	Department of Laboratory Medicine, Division of Clinical Virology, University of Medicine, Vienna	Bergthaler laboratory, CeMM Research Center for Molecular Medicine of the Austrian Academy of Sciences	Lukas Endler et al
EPI_ISL_1495576	Vienna COVID-19 Detection Initiative (VCDI)	Bergthaler laboratory, CeMM Research Center for Molecular Medicine of the Austrian Academy of Sciences	Lukas Endler et al
EPI_ISL_1495706	Austrian Agency for Health and Food Safety (AGES)	Bergthaler laboratory, CeMM Research Center for Molecular Medicine of the Austrian Academy of Sciences	Lukas Endler et al
EPI_ISL_1583371	Austrian Agency for Health and Food Safety (AGES)	Bergthaler laboratory, CeMM Research Center for Molecular Medicine of the Austrian Academy of Sciences	Lukas Endler et al
EPI_ISL_1495815	Department of Laboratory Medicine, Division of Clinical Virology, University of Medicine, Vienna	Bergthaler laboratory, CeMM Research Center for Molecular Medicine of the Austrian Academy of Sciences	Lukas Endler et al
EPI_ISL_1583534	Austrian Agency for Health and Food Safety (AGES)	Bergthaler laboratory, CeMM Research Center for Molecular Medicine of the Austrian Academy of Sciences	Lukas Endler et al
EPI_ISL_1671064	Austrian Agency for Health and Food Safety (AGES)	Bergthaler laboratory, CeMM Research Center for Molecular Medicine of the Austrian Academy of Sciences	Lukas Endler et al
EPI_ISL_1671014	Austrian Agency for Health and Food Safety (AGES)	Bergthaler laboratory, CeMM Research Center for Molecular Medicine of the Austrian Academy of Sciences	Lukas Endler et al
EPI_ISL_1670958	Austrian Agency for Health and Food Safety (AGES)	Bergthaler laboratory, CeMM Research Center for Molecular Medicine of the Austrian Academy of Sciences	Lukas Endler et al
EPI_ISL_1671037	Austrian Agency for Health and Food Safety (AGES)	Bergthaler laboratory, CeMM Research Center for Molecular Medicine of the Austrian Academy of Sciences	Lukas Endler et al

EPI_ISL_1671010	Austrian Agency for Health and Food Safety (AGES)	Bergthaler laboratory, CeMM Research Center for Molecular Medicine of the Austrian Academy of Sciences	Lukas Endler et al
EPI_ISL_1671056	Austrian Agency for Health and Food Safety (AGES)	Bergthaler laboratory, CeMM Research Center for Molecular Medicine of the Austrian Academy of Sciences	Lukas Endler et al
EPI_ISL_1670969	Austrian Agency for Health and Food Safety (AGES)	Bergthaler laboratory, CeMM Research Center for Molecular Medicine of the Austrian Academy of Sciences	Lukas Endler et al
EPI_ISL_1671115	Austrian Agency for Health and Food Safety (AGES)	Bergthaler laboratory, CeMM Research Center for Molecular Medicine of the Austrian Academy of Sciences	Lukas Endler et al
EPI_ISL_1671060	Austrian Agency for Health and Food Safety (AGES)	Bergthaler laboratory, CeMM Research Center for Molecular Medicine of the Austrian Academy of Sciences	Lukas Endler et al
EPI_ISL_1671017	Austrian Agency for Health and Food Safety (AGES)	Bergthaler laboratory, CeMM Research Center for Molecular Medicine of the Austrian Academy of Sciences	Lukas Endler et al
EPI_ISL_1180680	Public Health Authority of the Slovak Republic	Bergthaler laboratory, CeMM Research Center for Molecular Medicine of the Austrian Academy of Sciences	Lukas Endler et al
EPI_ISL_1180691	Public Health Authority of the Slovak Republic	Bergthaler laboratory, CeMM Research Center for Molecular Medicine of the Austrian Academy of Sciences	Lukas Endler et al
EPI_ISL_1180692	Public Health Authority of the Slovak Republic	Bergthaler laboratory, CeMM Research Center for Molecular Medicine of the Austrian Academy of Sciences	Lukas Endler et al
EPI_ISL_1209258	Public Health Authority of the Slovak Republic	Bergthaler laboratory, CeMM Research Center for Molecular Medicine of the Austrian Academy of Sciences	Lukas Endler et al
EPI_ISL_1209260	Public Health Authority of the Slovak Republic	Bergthaler laboratory, CeMM Research Center for Molecular Medicine of the Austrian Academy of Sciences	Lukas Endler et al
EPI_ISL_603118	Lithuanian University of Health Sciences Hospital, Department of Laboratory Medicine	Lithuanian University of Health Sciences, Molecular cardiology lab.	Lukas Zemaitis et al
EPI_ISL_603088	Lithuanian University of Health Sciences Hospital, Department of Laboratory Medicine	Lithuanian University of Health Sciences, Molecular cardiology lab.	Lukas Zemaitis et al
EPI_ISL_1657243	NVSPL	National Public Health Surveillance Laboratory	Lukas Zemaitis et al
EPI_ISL_1657152	NMVRVI	National Public Health Surveillance Laboratory	Lukas Zemaitis et al
EPI_ISL_1655018	NMVRVI	National Public Health Surveillance Laboratory	Lukas Zemaitis et al
EPI_ISL_1655991	NMVRVI	National Public Health Surveillance Laboratory	Lukas Zemaitis et al
EPI_ISL_1580062	NMVRVI	National Public Health Surveillance Laboratory	Lukas Zemaitis et al
EPI_ISL_1580272	NMVRVI	National Public Health Surveillance Laboratory	Lukas Zemaitis et al
EPI_ISL_1656121	NMVRVI	National Public Health Surveillance Laboratory	Lukas Zemaitis et al
EPI_ISL_1580281	NMVRVI	National Public Health Surveillance Laboratory	Lukas Zemaitis et al
EPI_ISL_1580331	NMVRVI	National Public Health Surveillance Laboratory	Lukas Zemaitis et al
EPI_ISL_1579767	Rezus LT	National Public Health Surveillance Laboratory	Lukas Zemaitis et al
EPI_ISL_636604	Lithuanian University of Health Sciences Hospital, Department of Laboratory Medicine	Lithuanian University of Health Sciences, Molecular cardiology lab.	Lukas Zemaitis et al
EPI_ISL_636839	Lithuanian University of Health Sciences Hospital, Department of Laboratory Medicine	Lithuanian University of Health Sciences, Molecular cardiology lab.	Lukas Zemaitis et al

EPI_ISL_636840	Lithuanian University of Health Sciences Hospital, Department of Laboratory Medicine	Lithuanian University of Health Sciences, Molecular cardiology lab.	Lukas Zemaitis et al
EPI_ISL_636841	Lithuanian University of Health Sciences Hospital, Department of Laboratory Medicine	Lithuanian University of Health Sciences, Molecular cardiology lab.	Lukas Zemaitis et al
EPI_ISL_636842	Lithuanian University of Health Sciences Hospital, Department of Laboratory Medicine	Lithuanian University of Health Sciences, Molecular cardiology lab.	Lukas Zemaitis et al
EPI_ISL_636843	Lithuanian University of Health Sciences Hospital, Department of Laboratory Medicine	Lithuanian University of Health Sciences, Molecular cardiology lab.	Lukas Zemaitis et al
EPI_ISL_636844	Lithuanian University of Health Sciences Hospital, Department of Laboratory Medicine	Lithuanian University of Health Sciences, Molecular cardiology lab.	Lukas Zemaitis et al
EPI_ISL_603119	Lithuanian University of Health Sciences Hospital, Department of Laboratory Medicine	Lithuanian University of Health Sciences, Molecular cardiology lab.	Lukas Zemaitis et al
EPI_ISL_636903	Lithuanian University of Health Sciences Hospital, Department of Laboratory Medicine	Lithuanian University of Health Sciences, Molecular cardiology lab.	Lukas Zemaitis et al
EPI_ISL_636906	Lithuanian University of Health Sciences Hospital, Department of Laboratory Medicine	Lithuanian University of Health Sciences, Molecular cardiology lab.	Lukas Zemaitis et al
EPI_ISL_770499	Lithuanian University of Health Sciences Hospital, Department of Laboratory Medicine	Lithuanian University of Health Sciences, Molecular cardiology lab.	Lukas Zemaitis et al
EPI_ISL_541873	Lithuanian University of Health Sciences Hospital, Department of Laboratory Medicine	Lithuanian University of Health Sciences, Laboratory of Molecular Cardiology	Lukas Zemaitis et al
EPI_ISL_1084608	Hospital for Infectious Diseases, Molecular Diagnostics Laboratory, Warsaw, Poland	43. Laboratory of Recombinant Vaccines, Intercollegiate Faculty of Biotechnology University of Gdansk and Medical University of Gdansk, 2. ViroGenetics - BSL3 Laboratory of Virology, Małopolska Centre of Biotechnology, Jagiellonian University	Lukasz Rabalski et al
EPI_ISL_906711	Hematology Laboratory, Section of Molecular Diagnostics, University Clinical Centre, Medical University of Gdansk	Laboratory of Recombinant Vaccines	Lukasz Rabalski et al
EPI_ISL_906732	Hematology Laboratory, Section of Molecular Diagnostics, University Clinical Centre, Medical University of Gdansk	Laboratory of Recombinant Vaccines	Lukasz Rabalski et al
EPI_ISL_1229508	Hematology Laboratory, Section of Molecular Diagnostics, University Clinical Centre, Medical University of Gdansk	1. Laboratory of Recombinant Vaccines 2. Department of Biology and Medical Genetics 3. Laboratory of Clinical Genetics	Lukasz Rabalski et al
EPI_ISL_1229410	Hematology Laboratory, Section of Molecular Diagnostics, University Clinical Centre, Medical University of Gdansk	Laboratory of Recombinant Vaccines	Lukasz Rabalski et al
EPI_ISL_906752	Hematology Laboratory, Section of Molecular Diagnostics, University Clinical Centre, Medical University of Gdansk	Laboratory of Recombinant Vaccines	Lukasz Rabalski et al
EPI_ISL_440499	Department of Pathology, University of Cambridge	Wellcome Sanger Institute for the COVID-19 Genomics UK (COG-UK) consortium	Luke W Meredith et al
EPI_ISL_941944	Centro de Investigaciones en Microbiología y Biotecnología-UR (CIMBIUR), Facultad de Ciencias Naturales, Universidad del Rosario, Bogotá, Colombia	Centro de Investigaciones en Microbiología y Biotecnología-UR (CIMBIUR), Facultad de Ciencias Naturales, Universidad del Rosario, Bogotá, Colombia Instituto Nacional de Salud, Bogotá, Colombia Icahn School of Medicine at Mount Sinai, New York, USA	Luz Helena Patiño et al
EPI_ISL_942004	Centro de Investigaciones en Microbiología y Biotecnología-UR (CIMBIUR), Facultad de Ciencias Naturales, Universidad del Rosario, Bogotá, Colombia	Centro de Investigaciones en Microbiología y Biotecnología-UR (CIMBIUR), Facultad de Ciencias Naturales, Universidad del Rosario, Bogotá, Colombia Instituto Nacional de Salud, Bogotá, Colombia Icahn School of Medicine at Mount Sinai, New York, USA	Luz Helena Patiño et al

EPI_ISL_941997	Centro de Investigaciones en Microbiología y Biotecnología-UR (CIMBIUR), Facultad de Ciencias Naturales, Universidad del Rosario, Bogotá, Colombia	Centro de Investigaciones en Microbiología y Biotecnología-UR (CIMBIUR), Facultad de Ciencias Naturales, Universidad del Rosario, Bogotá, Colombia Instituto Nacional de Salud, Bogotá, Colombia Icahn School of Medicine at Mount Sinai, New York, USA	Luz Helena Patiño et al
EPI_ISL_941998	Centro de Investigaciones en Microbiología y Biotecnología-UR (CIMBIUR), Facultad de Ciencias Naturales, Universidad del Rosario, Bogotá, Colombia	Centro de Investigaciones en Microbiología y Biotecnología-UR (CIMBIUR), Facultad de Ciencias Naturales, Universidad del Rosario, Bogotá, Colombia Instituto Nacional de Salud, Bogotá, Colombia Icahn School of Medicine at Mount Sinai, New York, USA	Luz Helena Patiño et al
EPI_ISL_941947	Centro de Investigaciones en Microbiología y Biotecnología-UR (CIMBIUR), Facultad de Ciencias Naturales, Universidad del Rosario, Bogotá, Colombia	Centro de Investigaciones en Microbiología y Biotecnología-UR (CIMBIUR), Facultad de Ciencias Naturales, Universidad del Rosario, Bogotá, Colombia Instituto Nacional de Salud, Bogotá, Colombia Icahn School of Medicine at Mount Sinai, New York, USA	Luz Helena Patiño et al
EPI_ISL_941942	Instituto Nacional de Salud, Bogotá, Colombia	Centro de Investigaciones en Microbiología y Biotecnología-UR (CIMBIUR), Facultad de Ciencias Naturales, Universidad del Rosario, Bogotá, Colombia Instituto Nacional de Salud, Bogotá, Colombia Icahn School of Medicine at Mount Sinai, New York, USA	Luz Helena Patiño et al
EPI_ISL_941943	Instituto Nacional de Salud, Bogotá, Colombia	Centro de Investigaciones en Microbiología y Biotecnología-UR (CIMBIUR), Facultad de Ciencias Naturales, Universidad del Rosario, Bogotá, Colombia Instituto Nacional de Salud, Bogotá, Colombia Icahn School of Medicine at Mount Sinai, New York, USA	Luz Helena Patiño et al
EPI_ISL_941994	Instituto Nacional de Salud, Bogotá, Colombia	Centro de Investigaciones en Microbiología y Biotecnología-UR (CIMBIUR), Facultad de Ciencias Naturales, Universidad del Rosario, Bogotá, Colombia Instituto Nacional de Salud, Bogotá, Colombia Icahn School of Medicine at Mount Sinai, New York, USA	Luz Helena Patiño et al
EPI_ISL_941955	Instituto Nacional de Salud, Bogotá, Colombia	Centro de Investigaciones en Microbiología y Biotecnología-UR (CIMBIUR), Facultad de Ciencias Naturales, Universidad del Rosario, Bogotá, Colombia Instituto Nacional de Salud, Bogotá, Colombia Icahn School of Medicine at Mount Sinai, New York, USA	Luz Helena Patiño et al
EPI_ISL_964903	Laboratorio Central Mg. Luis Alfredo Pianciola on behalf of 'Proyecto Argentino Interinstitucional de genomica de SARS-CoV-2' (PAIS Consortium)	Laboratorio Central Mg. Luis Alfredo Pianciola on behalf of 'Proyecto Argentino Interinstitucional de genomica de SARS-CoV-2' (PAIS Consortium)	M Mazzeo et al
EPI_ISL_528835	CSIR-Centre for Cellular and Molecular Biology	CSIR-Centre for Cellular and Molecular Biology	M Soujanya Reddy et al
EPI_ISL_1000972	Hospital Universitario de Gran Canaria Dr. Negrín	SeqCOVID-SPAIN consortium/IBV(CSIC)	M. Carmen Pérez González et al
EPI_ISL_1000974	Hospital Universitario de Gran Canaria Dr. Negrín	SeqCOVID-SPAIN consortium/IBV(CSIC)	M. Carmen Pérez González et al
EPI_ISL_962942	Hospital Universitario de Gran Canaria Dr. Negrín	SeqCOVID-SPAIN consortium/IBV(CSIC)	M. Carmen Pérez González et al
EPI_ISL_962973	Hospital Universitario de Gran Canaria Dr. Negrín	SeqCOVID-SPAIN consortium/IBV(CSIC)	M. Carmen Pérez González et al
EPI_ISL_455472	Laboratory for Respiratory Viruses, Cantacuzino National Military-Medical Institute for Research and Development	Cantacuzino Institute	M.Lazar et al
EPI_ISL_491474	Research Institute for Tropical Medicine	Research Institute for Tropical Medicine	Ma. Angelica Tujan et al
EPI_ISL_512301	Hematology Laboratory, Section of Molecular Diagnostics, University Clinical Centre, Medical University of Gdansk	Department of Virology, Faculty of Medicine, University of Helsinki, Helsinki, Finland	Maciej Grzybek et al
EPI_ISL_512303	Hematology Laboratory, Section of Molecular Diagnostics, University Clinical Centre, Medical University of Gdansk	Department of Virology, Faculty of Medicine, University of Helsinki, Helsinki, Finland	Maciej Grzybek et al
EPI_ISL_1008733	genXone SA, Molecular Diagnostics Laboratory / NZOZ	genXone SA, Research & Development Laboratory	Maciej Sykulski et al

EPI_ISL_876001	Laboratory of Molecular Biology, Diagnostyka sp. z o.o.	genXone SA, Research & Development Laboratory	Maciej Sykulski et al
EPI_ISL_876010	Laboratory of Molecular Biology, Diagnostyka sp. z o.o.	genXone SA, Research & Development Laboratory	Maciej Sykulski et al
EPI_ISL_925041	Laboratory of Molecular Biology, Diagnostyka sp. z o.o.	genXone SA, Research & Development Laboratory	Maciej Sykulski et al
EPI_ISL_876015	Laboratory of Molecular Biology, Diagnostyka sp. z o.o.	genXone SA, Research & Development Laboratory	Maciej Sykulski et al
EPI_ISL_1295181	Laboratory Diagnostics and Clinical Immunology of Developmental Age, Medical University of Warsaw	genXone SA, Research & Development Laboratory; The Faculty of Mathematics, Informatics and Mechanics of the University of Warsaw	Maciej Sykulski et al
EPI_ISL_754904	Laboratory Diagnostics and Clinical Immunology of Developmental Age, Medical University of Warsaw	genXone SA, Research & Development Laboratory; The Faculty of Mathematics, Informatics and Mechanics of the University of Warsaw	Maciej Sykulski et al
EPI_ISL_754906	Laboratory Diagnostics and Clinical Immunology of Developmental Age, Medical University of Warsaw	genXone SA, Research & Development Laboratory; The Faculty of Mathematics, Informatics and Mechanics of the University of Warsaw	Maciej Sykulski et al
EPI_ISL_754907	Laboratory Diagnostics and Clinical Immunology of Developmental Age, Medical University of Warsaw	genXone SA, Research & Development Laboratory; The Faculty of Mathematics, Informatics and Mechanics of the University of Warsaw	Maciej Sykulski et al
EPI_ISL_754913	Laboratory Diagnostics and Clinical Immunology of Developmental Age, Medical University of Warsaw	genXone SA, Research & Development Laboratory; The Faculty of Mathematics, Informatics and Mechanics of the University of Warsaw	Maciej Sykulski et al
EPI_ISL_754909	Laboratory Diagnostics and Clinical Immunology of Developmental Age, Medical University of Warsaw	genXone SA, Research & Development Laboratory; The Faculty of Mathematics, Informatics and Mechanics of the University of Warsaw	Maciej Sykulski et al
EPI_ISL_754910	Laboratory Diagnostics and Clinical Immunology of Developmental Age, Medical University of Warsaw	genXone SA, Research & Development Laboratory; The Faculty of Mathematics, Informatics and Mechanics of the University of Warsaw	Maciej Sykulski et al
EPI_ISL_754902	Laboratory Diagnostics and Clinical Immunology of Developmental Age, Medical University of Warsaw	genXone SA, Research & Development Laboratory; The Faculty of Mathematics, Informatics and Mechanics of the University of Warsaw	Maciej Sykulski et al
EPI_ISL_754908	Laboratory Diagnostics and Clinical Immunology of Developmental Age, Medical University of Warsaw	genXone SA, Research & Development Laboratory; The Faculty of Mathematics, Informatics and Mechanics of the University of Warsaw	Maciej Sykulski et al
EPI_ISL_527918	University Hospital Basel, Clinical Virology	University Hospital Basel, Clinical Bacteriology	Madlen Stange et al
EPI_ISL_527919	University Hospital Basel, Clinical Virology	University Hospital Basel, Clinical Bacteriology	Madlen Stange et al
EPI_ISL_528291	University Hospital Basel, Clinical Virology	University Hospital Basel, Clinical Bacteriology	Madlen Stange et al
EPI_ISL_463749	Pasteur Institute of Iran	Rapid Response Team	Mahboobeh Rafigh et al
EPI_ISL_636765	National Centre for Disease control (NCDC)	NCDC/CSIR-IGIB	Mahesh S. Dhar1* et al
EPI_ISL_949180	Laboratory of Virology and Molecular Diagnostics	Institute of Public Health of Republic of North Macedonia Laboratory of Virology and Molecular Diagnostics	Maja Kuzmanovska et al
EPI_ISL_418993	National Public Health Laboratory, National Centre for Infectious Diseases	National Public Health Laboratory, National Centre for Infectious Diseases	Mak TM et al
EPI_ISL_462318	National Public Health Laboratory, National Centre for Infectious Diseases	National Public Health Laboratory, National Centre for Infectious Diseases	Mak TM et al
EPI_ISL_462342	National Public Health Laboratory, National Centre for Infectious Diseases	National Public Health Laboratory, National Centre for Infectious Diseases	Mak TM et al
EPI_ISL_462346	National Public Health Laboratory, National Centre for Infectious Diseases	National Public Health Laboratory, National Centre for Infectious Diseases	Mak TM et al
EPI_ISL_469122	National Public Health Laboratory, National Centre for Infectious Diseases	National Public Health Laboratory, National Centre for Infectious Diseases	Mak TM et al
EPI_ISL_475938	National Public Health Laboratory, National Centre for Infectious Diseases	National Public Health Laboratory, National Centre for Infectious Diseases	Mak TM et al
EPI_ISL_475940	National Public Health Laboratory, National Centre for Infectious Diseases	National Public Health Laboratory, National Centre for Infectious Diseases	Mak TM et al
EPI_ISL_475948	National Public Health Laboratory, National Centre for Infectious Diseases	National Public Health Laboratory, National Centre for Infectious Diseases	Mak TM et al

EPI_ISL_475952	National Public Health Laboratory, National Centre for Infectious Diseases	National Public Health Laboratory, National Centre for Infectious Diseases	Mak TM et al
EPI_ISL_475997	National Public Health Laboratory, National Centre for Infectious Diseases	National Public Health Laboratory, National Centre for Infectious Diseases	Mak TM et al
EPI_ISL_475999	National Public Health Laboratory, National Centre for Infectious Diseases	National Public Health Laboratory, National Centre for Infectious Diseases	Mak TM et al
EPI_ISL_479591	National Public Health Laboratory, National Centre for Infectious Diseases	National Public Health Laboratory, National Centre for Infectious Diseases	Mak TM et al
EPI_ISL_490067	National Public Health Laboratory, National Centre for Infectious Diseases	National Public Health Laboratory, National Centre for Infectious Diseases	Mak TM et al
EPI_ISL_493390	National Public Health Laboratory, National Centre for Infectious Diseases	National Public Health Laboratory, National Centre for Infectious Diseases	Mak TM et al
EPI_ISL_498599	National Public Health Laboratory, National Centre for Infectious Diseases	National Public Health Laboratory, National Centre for Infectious Diseases	Mak TM et al
EPI_ISL_516815	National Public Health Laboratory, National Centre for Infectious Diseases	National Public Health Laboratory, National Centre for Infectious Diseases	Mak TM et al
EPI_ISL_516819	National Public Health Laboratory, National Centre for Infectious Diseases	National Public Health Laboratory, National Centre for Infectious Diseases	Mak TM et al
EPI_ISL_516824	National Public Health Laboratory, National Centre for Infectious Diseases	National Public Health Laboratory, National Centre for Infectious Diseases	Mak TM et al
EPI_ISL_536431	National Public Health Laboratory, National Centre for Infectious Diseases	National Public Health Laboratory, National Centre for Infectious Diseases	Mak TM et al
EPI_ISL_536435	National Public Health Laboratory, National Centre for Infectious Diseases	National Public Health Laboratory, National Centre for Infectious Diseases	Mak TM et al
EPI_ISL_536445	National Public Health Laboratory, National Centre for Infectious Diseases	National Public Health Laboratory, National Centre for Infectious Diseases	Mak TM et al
EPI_ISL_536447	National Public Health Laboratory, National Centre for Infectious Diseases	National Public Health Laboratory, National Centre for Infectious Diseases	Mak TM et al
EPI_ISL_536451	National Public Health Laboratory, National Centre for Infectious Diseases	National Public Health Laboratory, National Centre for Infectious Diseases	Mak TM et al
EPI_ISL_443187	National Virology Reference Laboratory	National Public Health Laboratory, National Centre for Infectious Diseases	Mak Tze Minn et al
EPI_ISL_435674	National Virology Reference Laboratory	National Public Health Laboratory, National Centre for Infectious Diseases	Mak Tze Minn et al
EPI_ISL_435677	National Virology Reference Laboratory	National Public Health Laboratory, National Centre for Infectious Diseases	Mak Tze Minn et al
EPI_ISL_435686	National Public Health Laboratory, National Centre for Infectious Diseases	National Public Health Laboratory, National Centre for Infectious Diseases	Mak Tze Minn et al
EPI_ISL_1196017	Manzini RRT	National Institute for Communicable Diseases of the National Health Laboratory Service	Maphalala GP et al
EPI_ISL_1196002	Women correctional	National Institute for Communicable Diseases of the National Health Laboratory Service	Maphalala GP et al
EPI_ISL_1196003	Homecare	National Institute for Communicable Diseases of the National Health Laboratory Service	Maphalala GP et al
EPI_ISL_1196004	Mbabane PHU	National Institute for Communicable Diseases of the National Health Laboratory Service	Maphalala GP et al
EPI_ISL_1196005	RSP Clinic	National Institute for Communicable Diseases of the National Health Laboratory Service	Maphalala GP et al
EPI_ISL_1196006	TLC Clinic	National Institute for Communicable Diseases of the National Health Laboratory Service	Maphalala GP et al
EPI_ISL_1196008	Nhlangano Health Centre	National Institute for Communicable Diseases of the National Health Laboratory Service	Maphalala GP et al
EPI_ISL_1196009	Nhlangano Health Centre	National Institute for Communicable Diseases of the National Health Laboratory Service	Maphalala GP et al

EPI_ISL_1196010	Nhlangano Health Centre	National Institute for Communicable Diseases of the National Health Laboratory Service	Maphalala GP et al
EPI_ISL_1196011	Homecare	National Institute for Communicable Diseases of the National Health Laboratory Service	Maphalala GP et al
EPI_ISL_1196012	Homecare	National Institute for Communicable Diseases of the National Health Laboratory Service	Maphalala GP et al
EPI_ISL_1196013	Hlathikhulu Government Hospital	National Institute for Communicable Diseases of the National Health Laboratory Service	Maphalala GP et al
EPI_ISL_1196014	Homecare	National Institute for Communicable Diseases of the National Health Laboratory Service	Maphalala GP et al
EPI_ISL_1595918	NSTU COVID-19 Diagnostic Center	NSU Genome Research Institute (NGRI)	Maqsud Hossain et al
EPI_ISL_1626485	NSTU COVID-19 Diagnostic Center	NSU Genome Research Institute (NGRI)	Maqsud Hossain et al
EPI_ISL_1626488	NSTU COVID-19 Diagnostic Center	NSU Genome Research Institute (NGRI)	Maqsud Hossain et al
EPI_ISL_1626489	NSTU COVID-19 Diagnostic Center	NSU Genome Research Institute (NGRI)	Maqsud Hossain et al
EPI_ISL_1626490	NSTU COVID-19 Diagnostic Center	NSU Genome Research Institute (NGRI)	Maqsud Hossain et al
EPI_ISL_1626495	NSTU COVID-19 Diagnostic Center	NSU Genome Research Institute (NGRI)	Maqsud Hossain et al
EPI_ISL_1626502	NSTU COVID-19 Diagnostic Center	NSU Genome Research Institute (NGRI)	Maqsud Hossain et al
EPI_ISL_1626509	NSTU COVID-19 Diagnostic Center	NSU Genome Research Institute (NGRI)	Maqsud Hossain et al
EPI_ISL_1626514	NSTU COVID-19 Diagnostic Center	NSU Genome Research Institute (NGRI)	Maqsud Hossain et al
EPI_ISL_1626517	NSTU COVID-19 Diagnostic Center	NSU Genome Research Institute (NGRI)	Maqsud Hossain et al
EPI_ISL_1626519	NSTU COVID-19 Diagnostic Center	NSU Genome Research Institute (NGRI)	Maqsud Hossain et al
EPI_ISL_1626524	NSTU COVID-19 Diagnostic Center	NSU Genome Research Institute (NGRI)	Maqsud Hossain et al
EPI_ISL_1208770	Microbiology Department, Laboratori Clínic Metropolitana Nord. Hospital Universitari Germans Trias i Pujol.	Can Ruti SARS-CoV-2 Sequencing Hub (HUGTiP/IrsiCaixa/IGTP)	Marc Noguera-Julian et al
EPI_ISL_1583556	Microbiology Department, Laboratori Clínic Metropolitana Nord. Hospital Universitari Germans Trias i Pujol.	Can Ruti SARS-CoV-2 Sequencing Hub (HUGTiP/IrsiCaixa/IGTP)	Marc Noguera-Julian et al
EPI_ISL_1583601	Microbiology Department, Laboratori Clínic Metropolitana Nord. Hospital Universitari Germans Trias i Pujol.	Can Ruti SARS-CoV-2 Sequencing Hub (HUGTiP/IrsiCaixa/IGTP)	Marc Noguera-Julian et al
EPI_ISL_1583612	Microbiology Department, Laboratori Clínic Metropolitana Nord. Hospital Universitari Germans Trias i Pujol.	Can Ruti SARS-CoV-2 Sequencing Hub (HUGTiP/IrsiCaixa/IGTP)	Marc Noguera-Julian et al
EPI_ISL_1661933	Microbiology Department, Laboratori Clínic Metropolitana Nord. Hospital Universitari Germans Trias i Pujol.	Can Ruti SARS-CoV-2 Sequencing Hub (HUGTiP/IrsiCaixa/IGTP)	Marc Noguera-Julian et al
EPI_ISL_1661943	Microbiology Department, Laboratori Clínic Metropolitana Nord. Hospital Universitari Germans Trias i Pujol.	Can Ruti SARS-CoV-2 Sequencing Hub (HUGTiP/IrsiCaixa/IGTP)	Marc Noguera-Julian et al
EPI_ISL_1661956	Microbiology Department, Laboratori Clínic Metropolitana Nord. Hospital Universitari Germans Trias i Pujol.	Can Ruti SARS-CoV-2 Sequencing Hub (HUGTiP/IrsiCaixa/IGTP)	Marc Noguera-Julian et al
EPI_ISL_1547769	Microbiology Department, Laboratori Clínic Metropolitana Nord. Hospital Universitari Germans Trias i Pujol.	Can Ruti SARS-CoV-2 Sequencing Hub (HUGTiP/IrsiCaixa/IGTP)	Marc Noguera-Julian et al

EPI_ISL_1636455	Microbiology Department, Laboratori Clínic Metropolitana Nord. Hospital Universitari Germans Trias i Pujol.	Can Ruti SARS-CoV-2 Sequencing Hub (HUGTiP/IrsiCaixa/IGTP)	Marc Noguera-Julian et al
EPI_ISL_832103	Hospital Universitari Germans Trias i Pujol(HUGTiP)/Fundació Lluita contra la SIDA (FLSida)	IrsiCaixa AIDS Research Lab	Marc Noguera-Julian et al
EPI_ISL_1239012	Laboratório Central de Saúde Pública do Estado de Pernambuco (LACEN-PE)	WallauLab, Aggeu Magalhaes Institute	Marcelo Henrique dos Santos Paiva et al
EPI_ISL_1239013	Laboratório Central de Saúde Pública do Estado de Pernambuco (LACEN-PE)	WallauLab, Aggeu Magalhaes Institute	Marcelo Henrique dos Santos Paiva et al
EPI_ISL_1239015	Laboratório Central de Saúde Pública do Estado de Pernambuco (LACEN-PE)	WallauLab, Aggeu Magalhaes Institute	Marcelo Henrique dos Santos Paiva et al
EPI_ISL_572373	Laboratório Central de Saúde Pública do Estado de Pernambuco (LACEN-PE)	WallauLab, Aggeu Magalhaes Institute	Marcelo Henrique Santos Paiva et al
EPI_ISL_572374	Laboratório Central de Saúde Pública do Estado de Pernambuco (LACEN-PE)	WallauLab, Aggeu Magalhaes Institute	Marcelo Henrique Santos Paiva et al
EPI_ISL_572375	Laboratório Central de Saúde Pública do Estado de Pernambuco (LACEN-PE)	WallauLab, Aggeu Magalhaes Institute	Marcelo Henrique Santos Paiva et al
EPI_ISL_572379	Laboratório Central de Saúde Pública do Estado de Pernambuco (LACEN-PE)	WallauLab, Aggeu Magalhaes Institute	Marcelo Henrique Santos Paiva et al
EPI_ISL_572380	Laboratório Central de Saúde Pública do Estado de Pernambuco (LACEN-PE)	WallauLab, Aggeu Magalhaes Institute	Marcelo Henrique Santos Paiva et al
EPI_ISL_572381	Laboratório Central de Saúde Pública do Estado de Pernambuco (LACEN-PE)	WallauLab, Aggeu Magalhaes Institute	Marcelo Henrique Santos Paiva et al
EPI_ISL_572382	Laboratório Central de Saúde Pública do Estado de Pernambuco (LACEN-PE)	WallauLab, Aggeu Magalhaes Institute	Marcelo Henrique Santos Paiva et al
EPI_ISL_572384	Laboratório Central de Saúde Pública do Estado de Pernambuco (LACEN-PE)	WallauLab, Aggeu Magalhaes Institute	Marcelo Henrique Santos Paiva et al
EPI_ISL_572385	Laboratório Central de Saúde Pública do Estado de Pernambuco (LACEN-PE)	WallauLab, Aggeu Magalhaes Institute	Marcelo Henrique Santos Paiva et al
EPI_ISL_572386	Laboratório Central de Saúde Pública do Estado de Pernambuco (LACEN-PE)	WallauLab, Aggeu Magalhaes Institute	Marcelo Henrique Santos Paiva et al
EPI_ISL_572388	Laboratório Central de Saúde Pública do Estado de Pernambuco (LACEN-PE)	WallauLab, Aggeu Magalhaes Institute	Marcelo Henrique Santos Paiva et al
EPI_ISL_500463	Laboratório Central de Saúde Pública do Estado de Pernambuco (LACEN-PE)	WallauLab, Aggeu Magalhaes Institute	Marcelo Henrique Santos Paiva et al
EPI_ISL_572394	Laboratório Central de Saúde Pública do Estado de Pernambuco (LACEN-PE)	WallauLab, Aggeu Magalhaes Institute	Marcelo Henrique Santos Paiva et al
EPI_ISL_572396	Laboratório Central de Saúde Pública do Estado de Pernambuco (LACEN-PE)	WallauLab, Aggeu Magalhaes Institute	Marcelo Henrique Santos Paiva et al
EPI_ISL_500465	Laboratório Central de Saúde Pública do Estado de Pernambuco (LACEN-PE)	WallauLab, Aggeu Magalhaes Institute	Marcelo Henrique Santos Paiva et al
EPI_ISL_500867	Laboratório Central de Saúde Pública do Estado de Pernambuco (LACEN-PE)	WallauLab, Aggeu Magalhaes Institute	Marcelo Henrique Santos Paiva et al
EPI_ISL_500467	Laboratório Central de Saúde Pública do Estado de Pernambuco (LACEN-PE)	WallauLab, Aggeu Magalhaes Institute	Marcelo Henrique Santos Paiva et al
EPI_ISL_500873	Laboratório Central de Saúde Pública do Estado de Pernambuco (LACEN-PE)	WallauLab, Aggeu Magalhaes Institute	Marcelo Henrique Santos Paiva et al
EPI_ISL_500479	Laboratório Central de Saúde Pública do Estado de Pernambuco (LACEN-PE)	WallauLab, Aggeu Magalhaes Institute	Marcelo Henrique Santos Paiva et al
EPI_ISL_500875	Laboratório Central de Saúde Pública do Estado de Pernambuco (LACEN-PE)	WallauLab, Aggeu Magalhaes Institute	Marcelo Henrique Santos Paiva et al
EPI_ISL_572336	Laboratório Central de Saúde Pública do Estado de Pernambuco (LACEN-PE)	WallauLab, Aggeu Magalhaes Institute	Marcelo Henrique Santos Paiva et al
EPI_ISL_500481	Laboratório Central de Saúde Pública do Estado de Pernambuco (LACEN-PE)	WallauLab, Aggeu Magalhaes Institute	Marcelo Henrique Santos Paiva et al
EPI_ISL_572337	Laboratório Central de Saúde Pública do Estado de Pernambuco (LACEN-PE)	WallauLab, Aggeu Magalhaes Institute	Marcelo Henrique Santos Paiva et al
EPI_ISL_572338	Laboratório Central de Saúde Pública do Estado de Pernambuco (LACEN-PE)	WallauLab, Aggeu Magalhaes Institute	Marcelo Henrique Santos Paiva et al
EPI_ISL_572349	Laboratório Central de Saúde Pública do Estado de Pernambuco (LACEN-PE)	WallauLab, Aggeu Magalhaes Institute	Marcelo Henrique Santos Paiva et al

EPI_ISL_500483	Laboratório Central de Saúde Pública do Estado de Pernambuco (LACEN-PE)	WallauLab, Aggeu Magalhaes Institute	Marcelo Henrique Santos Paiva et al
EPI_ISL_572351	Laboratório Central de Saúde Pública do Estado de Pernambuco (LACEN-PE)	WallauLab, Aggeu Magalhaes Institute	Marcelo Henrique Santos Paiva et al
EPI_ISL_572358	Laboratório Central de Saúde Pública do Estado de Pernambuco (LACEN-PE)	WallauLab, Aggeu Magalhaes Institute	Marcelo Henrique Santos Paiva et al
EPI_ISL_572359	Laboratório Central de Saúde Pública do Estado de Pernambuco (LACEN-PE)	WallauLab, Aggeu Magalhaes Institute	Marcelo Henrique Santos Paiva et al
EPI_ISL_572360	Laboratório Central de Saúde Pública do Estado de Pernambuco (LACEN-PE)	WallauLab, Aggeu Magalhaes Institute	Marcelo Henrique Santos Paiva et al
EPI_ISL_572361	Laboratório Central de Saúde Pública do Estado de Pernambuco (LACEN-PE)	WallauLab, Aggeu Magalhaes Institute	Marcelo Henrique Santos Paiva et al
EPI_ISL_572362	Laboratório Central de Saúde Pública do Estado de Pernambuco (LACEN-PE)	WallauLab, Aggeu Magalhaes Institute	Marcelo Henrique Santos Paiva et al
EPI_ISL_572363	Laboratório Central de Saúde Pública do Estado de Pernambuco (LACEN-PE)	WallauLab, Aggeu Magalhaes Institute	Marcelo Henrique Santos Paiva et al
EPI_ISL_572366	Laboratório Central de Saúde Pública do Estado de Pernambuco (LACEN-PE)	WallauLab, Aggeu Magalhaes Institute	Marcelo Henrique Santos Paiva et al
EPI_ISL_572367	Laboratório Central de Saúde Pública do Estado de Pernambuco (LACEN-PE)	WallauLab, Aggeu Magalhaes Institute	Marcelo Henrique Santos Paiva et al
EPI_ISL_572371	Laboratório Central de Saúde Pública do Estado de Pernambuco (LACEN-PE)	WallauLab, Aggeu Magalhaes Institute	Marcelo Henrique Santos Paiva et al
EPI_ISL_572372	Laboratório Central de Saúde Pública do Estado de Pernambuco (LACEN-PE)	WallauLab, Aggeu Magalhaes Institute	Marcelo Henrique Santos Paiva et al
EPI_ISL_451971	1. ViroGenetics - BSL3 Laboratory of Virology, Małopolska Centre of Biotechnology, Jagiellonian University; 2. II Department of Internal Medicine, Faculty of Medicine, Jagiellonian University Medical College; 3. DIAGNOSTYKA Ltd.	1. ViroGenetics - BSL3 Laboratory of Virology, Małopolska Centre of Biotechnology, Jagiellonian University; 2. II Department of Internal Medicine, Faculty of Medicine, Jagiellonian University Medical College.	Marek Sanak et al
EPI_ISL_451973	1. ViroGenetics - BSL3 Laboratory of Virology, Małopolska Centre of Biotechnology, Jagiellonian University; 2. II Department of Internal Medicine, Faculty of Medicine, Jagiellonian University Medical College; 3. DIAGNOSTYKA Ltd.	1. ViroGenetics - BSL3 Laboratory of Virology, Małopolska Centre of Biotechnology, Jagiellonian University; 2. II Department of Internal Medicine, Faculty of Medicine, Jagiellonian University Medical College.	Marek Sanak et al
EPI_ISL_451975	1. ViroGenetics - BSL3 Laboratory of Virology, Małopolska Centre of Biotechnology, Jagiellonian University; 2. II Department of Internal Medicine, Faculty of Medicine, Jagiellonian University Medical College; 3. DIAGNOSTYKA Ltd.	1. ViroGenetics - BSL3 Laboratory of Virology, Małopolska Centre of Biotechnology, Jagiellonian University; 2. II Department of Internal Medicine, Faculty of Medicine, Jagiellonian University Medical College.	Marek Sanak et al
EPI_ISL_451980	1. ViroGenetics - BSL3 Laboratory of Virology, Małopolska Centre of Biotechnology, Jagiellonian University; 2. II Department of Internal Medicine, Faculty of Medicine, Jagiellonian University Medical College; 3. DIAGNOSTYKA Ltd.	1. ViroGenetics - BSL3 Laboratory of Virology, Małopolska Centre of Biotechnology, Jagiellonian University; 2. II Department of Internal Medicine, Faculty of Medicine, Jagiellonian University Medical College.	Marek Sanak et al
EPI_ISL_451981	1. ViroGenetics - BSL3 Laboratory of Virology, Małopolska Centre of Biotechnology, Jagiellonian University; 2. II Department of Internal Medicine, Faculty of Medicine, Jagiellonian University Medical College; 3. DIAGNOSTYKA Ltd.	1. ViroGenetics - BSL3 Laboratory of Virology, Małopolska Centre of Biotechnology, Jagiellonian University; 2. II Department of Internal Medicine, Faculty of Medicine, Jagiellonian University Medical College.	Marek Sanak et al
EPI_ISL_451982	1. ViroGenetics - BSL3 Laboratory of Virology, Małopolska Centre of Biotechnology, Jagiellonian University; 2. II Department of Internal Medicine, Faculty of Medicine, Jagiellonian University Medical College; 3. DIAGNOSTYKA Ltd.	1. ViroGenetics - BSL3 Laboratory of Virology, Małopolska Centre of Biotechnology, Jagiellonian University; 2. II Department of Internal Medicine, Faculty of Medicine, Jagiellonian University Medical College.	Marek Sanak et al
EPI_ISL_451984	1. ViroGenetics - BSL3 Laboratory of Virology, Małopolska Centre of Biotechnology, Jagiellonian University; 2. II Department of Internal Medicine, Faculty of Medicine, Jagiellonian University Medical College; 3. DIAGNOSTYKA Ltd.	1. ViroGenetics - BSL3 Laboratory of Virology, Małopolska Centre of Biotechnology, Jagiellonian University; 2. II Department of Internal Medicine, Faculty of Medicine, Jagiellonian University Medical College.	Marek Sanak et al

EPI_ISL_451985	1. ViroGenetics - BSL3 Laboratory of Virology, Małopolska Centre of Biotechnology, Jagiellonian University; 2. II Department of Internal Medicine, Faculty of Medicine, Jagiellonian University Medical College; 3. DIAGNOSTYKA Ltd.	1. ViroGenetics - BSL3 Laboratory of Virology, Małopolska Centre of Biotechnology, Jagiellonian University; 2. II Department of Internal Medicine, Faculty of Medicine, Jagiellonian University Medical College.	Marek Sanak et al
EPI_ISL_451986	1. ViroGenetics - BSL3 Laboratory of Virology, Małopolska Centre of Biotechnology, Jagiellonian University; 2. II Department of Internal Medicine, Faculty of Medicine, Jagiellonian University Medical College; 3. DIAGNOSTYKA Ltd.	1. ViroGenetics - BSL3 Laboratory of Virology, Małopolska Centre of Biotechnology, Jagiellonian University; 2. II Department of Internal Medicine, Faculty of Medicine, Jagiellonian University Medical College.	Marek Sanak et al
EPI_ISL_430339	NYU Langone Health	Departments of Pathology and Medicine, New York University School of Medicine	Maria Aguero-Rosenfeld et al
EPI_ISL_476838	National Influenza Centre for Northern Greece	National Influenza Centre for Northern Greece	Maria Christoforidi et al
EPI_ISL_1550388	Instituto Oswaldo Cruz	Laboratorio de Virologia, Faculdade de Medicina, Universidade Federal de Mato Grosso, campus Cuiabá	María de Fátima Ferreira et al
EPI_ISL_1395830	Inmunología del Hospital Perrando e Instituto de Medicina Regional de la UNNE	Grupo de Genómica y Bioinformática del Instituto de Investigación de la Cadena Láctea CONICET-INTA on behalf of 'Proyecto Argentino Interinstitucional de genomica de SARS-CoV-2' (PAIS Consortium)	María Delia Foussal et al
EPI_ISL_1335887	North Sumatera Health Office	Faculty of Medicine, Universitas Sumatera Utara; Institute of Tropical Disease, Universitas Airlangga	Maria I Lusida et al
EPI_ISL_614301	Faroese National Reference Laboratory for Fish and Animal Diseases	Faroese National Reference Laboratory for Fish and Animal Diseases	Maria Marjunardóttir Dahl et al
EPI_ISL_614302	Faroese National Reference Laboratory for Fish and Animal Diseases	Faroese National Reference Laboratory for Fish and Animal Diseases	Maria Marjunardóttir Dahl et al
EPI_ISL_614305	Faroese National Reference Laboratory for Fish and Animal Diseases	Faroese National Reference Laboratory for Fish and Animal Diseases	Maria Marjunardóttir Dahl et al
EPI_ISL_614306	Faroese National Reference Laboratory for Fish and Animal Diseases	Faroese National Reference Laboratory for Fish and Animal Diseases	Maria Marjunardóttir Dahl et al
EPI_ISL_854594	Faroese National Reference Laboratory for Fish and Animal Diseases	Faroese National Reference Laboratory for Fish and Animal Diseases	Maria Marjunardóttir Dahl et al
EPI_ISL_455107	Narhalsan Mellerud	The Public Health Agency of Sweden	Maria Nykvist et al
EPI_ISL_410546	INMI Lazzaro Spallanzani IRCCS	Laboratory of Virology, INMI Lazzaro Spallanzani IRCCS	Maria R. Capobianchi et al
EPI_ISL_410545	INMI Lazzaro Spallanzani IRCCS	Laboratory of Virology, INMI Lazzaro Spallanzani IRCCS	Maria R. Capobianchi et al
EPI_ISL_983221	University Health Network/Mount Sinai Hospital Department of Microbiology	Ontario Institute for Cancer Research	Marie-Ming Aynaud et al
EPI_ISL_1511399	Laboratorio de Patologia Clinica - UNICAMP	Laboratorio de Estudos de Virus Emergentes - UNICAMP	Mariene R. Amorim et al
EPI_ISL_1404193	Lab voor klinische biologie	Lab voor klinische biologie	Marija Janevska et al
EPI_ISL_420142	Department for Virology, Molecular Biology and Genome Research, R. G. Lugar Center for Public Health Research, National Center for Disease Control and Public Health (NCDC) of Georgia.	Department for Virology, Molecular Biology and Genome Research, R. G. Lugar Center for Public Health Research, National Center for Disease Control and Public Health (NCDC) of Georgia.	Marine Murtskhvaladze et al
EPI_ISL_416477	R. G. Lugar Center for Public Health Research, National Center for Disease Control and Public Health (NCDC) of Georgia.	R. G. Lugar Center for Public Health Research, National Center for Disease Control and Public Health (NCDC) of Georgia.	Marine Murtskhvaladze et al
EPI_ISL_1583049	Hospital	National Reference Center for Viruses of Respiratory Infections, Institut Pasteur, Paris	Marion Barbet et al
EPI_ISL_832144	Hospital	National Reference Center for Viruses of Respiratory Infections, Institut Pasteur, Paris	Marion Barbet et al
EPI_ISL_1594450	Labo Analyses Med	National Reference Center for Viruses of Respiratory Infections, Institut Pasteur, Paris	Marion Barbet et al

EPI_ISL_890353	Labo Analyses Med	National Reference Center for Viruses of Respiratory Infections, Institut Pasteur, Paris	Marion Barbet et al
EPI_ISL_1060587	Outre mer	National Reference Center for Viruses of Respiratory Infections, Institut Pasteur, Paris	Marion Barbet et al
EPI_ISL_1060591	Outre mer	National Reference Center for Viruses of Respiratory Infections, Institut Pasteur, Paris	Marion Barbet et al
EPI_ISL_1096262	Outre mer	National Reference Center for Viruses of Respiratory Infections, Institut Pasteur, Paris	Marion Barbet et al
EPI_ISL_1201532	Outre Mer	National Reference Center for Viruses of Respiratory Infections, Institut Pasteur, Paris	Marion Barbet et al
EPI_ISL_1381215	Hospital	National Reference Center for Viruses of Respiratory Infections, Institut Pasteur, Paris	Marion Barbet et al
EPI_ISL_613420	Institut Pasteur de la Guadeloupe	Institut Pasteur de la Guadeloupe	Marion Barbet et al
EPI_ISL_613422	Institut Pasteur de la Guadeloupe	Institut Pasteur de la Guadeloupe	Marion Barbet et al
EPI_ISL_613425	Institut Pasteur de la Guadeloupe	Institut Pasteur de la Guadeloupe	Marion Barbet et al
EPI_ISL_613436	Institut Pasteur de la Guadeloupe	Institut Pasteur de la Guadeloupe	Marion Barbet et al
EPI_ISL_613441	Institut Pasteur de la Guadeloupe	Institut Pasteur de la Guadeloupe	Marion Barbet et al
EPI_ISL_613442	Institut Pasteur de la Guadeloupe	Institut Pasteur de la Guadeloupe	Marion Barbet et al
EPI_ISL_613445	Institut Pasteur de la Guadeloupe	Institut Pasteur de la Guadeloupe	Marion Barbet et al
EPI_ISL_1013418	Institut Pasteur de Guadeloupe	National Reference Center for Viruses of Respiratory Infections, Institut Pasteur, Paris	Marion Barbet et al
EPI_ISL_1013425	Institut Pasteur de Guadeloupe	National Reference Center for Viruses of Respiratory Infections, Institut Pasteur, Paris	Marion Barbet et al
EPI_ISL_1013426	Institut Pasteur de Guadeloupe	National Reference Center for Viruses of Respiratory Infections, Institut Pasteur, Paris	Marion Barbet et al
EPI_ISL_1443892	Outre mer	National Reference Center for Viruses of Respiratory Infections, Institut Pasteur, Paris	Marion Barbet et al
EPI_ISL_1416969	Outre mer	National Reference Center for Viruses of Respiratory Infections, Institut Pasteur, Paris	Marion Barbet et al
EPI_ISL_1416970	Outre mer	National Reference Center for Viruses of Respiratory Infections, Institut Pasteur, Paris	Marion Barbet et al
EPI_ISL_1416971	Outre mer	National Reference Center for Viruses of Respiratory Infections, Institut Pasteur, Paris	Marion Barbet et al
EPI_ISL_1443894	Outre mer	National Reference Center for Viruses of Respiratory Infections, Institut Pasteur, Paris	Marion Barbet et al
EPI_ISL_1416978	Outre mer	National Reference Center for Viruses of Respiratory Infections, Institut Pasteur, Paris	Marion Barbet et al
EPI_ISL_1416979	Outre mer	National Reference Center for Viruses of Respiratory Infections, Institut Pasteur, Paris	Marion Barbet et al
EPI_ISL_1416983	Outre mer	National Reference Center for Viruses of Respiratory Infections, Institut Pasteur, Paris	Marion Barbet et al

EPI_ISL_1416985	Outre mer	National Reference Center for Viruses of Respiratory Infections, Institut Pasteur, Paris	Marion Barbet et al
EPI_ISL_1416988	Outre mer	National Reference Center for Viruses of Respiratory Infections, Institut Pasteur, Paris	Marion Barbet et al
EPI_ISL_1416990	Outre mer	National Reference Center for Viruses of Respiratory Infections, Institut Pasteur, Paris	Marion Barbet et al
EPI_ISL_1660296	Virology Unit, Institut Pasteur de Madagascar	Virology Unit, Institut Pasteur de Madagascar	Marion Barbet et al
EPI_ISL_1660305	Virology Unit, Institut Pasteur de Madagascar	Virology Unit, Institut Pasteur de Madagascar	Marion Barbet et al
EPI_ISL_1660310	Virology Unit, Institut Pasteur de Madagascar	Virology Unit, Institut Pasteur de Madagascar	Marion Barbet et al
EPI_ISL_1660312	Virology Unit, Institut Pasteur de Madagascar	Virology Unit, Institut Pasteur de Madagascar	Marion Barbet et al
EPI_ISL_1660313	Virology Unit, Institut Pasteur de Madagascar	Virology Unit, Institut Pasteur de Madagascar	Marion Barbet et al
EPI_ISL_1660315	Virology Unit, Institut Pasteur de Madagascar	Virology Unit, Institut Pasteur de Madagascar	Marion Barbet et al
EPI_ISL_1660317	Virology Unit, Institut Pasteur de Madagascar	Virology Unit, Institut Pasteur de Madagascar	Marion Barbet et al
EPI_ISL_1660318	Virology Unit, Institut Pasteur de Madagascar	Virology Unit, Institut Pasteur de Madagascar	Marion Barbet et al
EPI_ISL_1660322	Virology Unit, Institut Pasteur de Madagascar	Virology Unit, Institut Pasteur de Madagascar	Marion Barbet et al
EPI_ISL_1660323	Virology Unit, Institut Pasteur de Madagascar	Virology Unit, Institut Pasteur de Madagascar	Marion Barbet et al
EPI_ISL_1660329	Virology Unit, Institut Pasteur de Madagascar	Virology Unit, Institut Pasteur de Madagascar	Marion Barbet et al
EPI_ISL_1660331	Virology Unit, Institut Pasteur de Madagascar	Virology Unit, Institut Pasteur de Madagascar	Marion Barbet et al
EPI_ISL_1201507	Hopital	National Reference Center for Viruses of Respiratory Infections, Institut Pasteur, Paris	Marion Barbet et al
EPI_ISL_1219962	Labo Analyses Med	National Reference Center for Viruses of Respiratory Infections, Institut Pasteur, Paris	Marion Barbet et al
EPI_ISL_1219949	Labo Analyses Med	National Reference Center for Viruses of Respiratory Infections, Institut Pasteur, Paris	Marion Barbet et al
EPI_ISL_1219963	Labo Analyses Med	National Reference Center for Viruses of Respiratory Infections, Institut Pasteur, Paris	Marion Barbet et al
EPI_ISL_459970	Institut Pasteur du Maroc	Institut Pasteur du Maroc	Marion Barbet et al
EPI_ISL_459971	Institut Pasteur du Maroc	Institut Pasteur du Maroc	Marion Barbet et al
EPI_ISL_459972	Institut Pasteur du Maroc	Institut Pasteur du Maroc	Marion Barbet et al
EPI_ISL_459974	Institut Pasteur du Maroc	Institut Pasteur du Maroc	Marion Barbet et al
EPI_ISL_459976	Institut Pasteur du Maroc	Institut Pasteur du Maroc	Marion Barbet et al
EPI_ISL_459981	Institut Pasteur du Maroc	Institut Pasteur du Maroc	Marion Barbet et al
EPI_ISL_459982	Institut Pasteur du Maroc	Institut Pasteur du Maroc	Marion Barbet et al
EPI_ISL_613429	Institut Pasteur de la Guadeloupe	Institut Pasteur de la Guadeloupe	Marion Barbet et al
EPI_ISL_613446	Institut Pasteur de la Guadeloupe	Institut Pasteur de la Guadeloupe	Marion Barbet et al

EPI_ISL_613447	Institut Pasteur de la Guadeloupe	Institut Pasteur de la Guadeloupe	Marion Barbet et al
EPI_ISL_613448	Institut Pasteur de la Guadeloupe	Institut Pasteur de la Guadeloupe	Marion Barbet et al
EPI_ISL_613450	Institut Pasteur de la Guadeloupe	Institut Pasteur de la Guadeloupe	Marion Barbet et al
EPI_ISL_613451	Institut Pasteur de la Guadeloupe	Institut Pasteur de la Guadeloupe	Marion Barbet et al
EPI_ISL_613452	Institut Pasteur de la Guadeloupe	Institut Pasteur de la Guadeloupe	Marion Barbet et al
EPI_ISL_613453	Institut Pasteur de la Guadeloupe	Institut Pasteur de la Guadeloupe	Marion Barbet et al
EPI_ISL_613454	Institut Pasteur de la Guadeloupe	Institut Pasteur de la Guadeloupe	Marion Barbet et al
EPI_ISL_613455	Institut Pasteur de la Guadeloupe	Institut Pasteur de la Guadeloupe	Marion Barbet et al
EPI_ISL_613456	Institut Pasteur de la Guadeloupe	Institut Pasteur de la Guadeloupe	Marion Barbet et al
EPI_ISL_1034760	Bundeswehr Institute of Microbiology	Bundeswehr Institute of Microbiology	Markus Antwerpen et al
EPI_ISL_1000998	Bundeswehr Institute of Microbiology	Bundeswehr Institute of Microbiology	Markus Antwerpen et al
EPI_ISL_1000999	Bundeswehr Institute of Microbiology	Bundeswehr Institute of Microbiology	Markus Antwerpen et al
EPI_ISL_1001000	Bundeswehr Institute of Microbiology	Bundeswehr Institute of Microbiology	Markus Antwerpen et al
EPI_ISL_1001001	Bundeswehr Institute of Microbiology	Bundeswehr Institute of Microbiology	Markus Antwerpen et al
EPI_ISL_1001002	Bundeswehr Institute of Microbiology	Bundeswehr Institute of Microbiology	Markus Antwerpen et al
EPI_ISL_1001003	Bundeswehr Institute of Microbiology	Bundeswehr Institute of Microbiology	Markus Antwerpen et al
EPI_ISL_1001004	Bundeswehr Institute of Microbiology	Bundeswehr Institute of Microbiology	Markus Antwerpen et al
EPI_ISL_1034755	Bundeswehr Institute of Microbiology	Bundeswehr Institute of Microbiology	Markus Antwerpen et al
EPI_ISL_1034757	Bundeswehr Institute of Microbiology	Bundeswehr Institute of Microbiology	Markus Antwerpen et al
EPI_ISL_732530	Bundeswehr Institute of Microbiology	Bundeswehr Institute of Microbiology	Markus Antwerpen et al
EPI_ISL_732526	Bundeswehr Institute of Microbiology	Bundeswehr Institute of Microbiology	Markus Antwerpen et al
EPI_ISL_754239	Santosa Hospital Bandung Central	School of Life Sciences and Technology & School of Pharmacy-Institut Teknologi Bandung; Molecular Genetics Laboratory-Faculty of Medicine-Universitas Padjadjaran; Laboratorium Kesehatan Provinsi Jawa Barat	Marselina Irasonia Tan et al
EPI_ISL_1492637	Laboratorio Central de Salud Publica de Paraguay	Laboratorio Central de Salud Publica de Paraguay	Marta Giovanetti et al
EPI_ISL_1492646	Laboratorio Central de Salud Publica de Paraguay	Laboratorio Central de Salud Publica de Paraguay	Marta Giovanetti et al
EPI_ISL_1492647	Laboratorio Central de Salud Publica de Paraguay	Laboratorio Central de Salud Publica de Paraguay	Marta Giovanetti et al
EPI_ISL_1492648	Laboratorio Central de Salud Publica de Paraguay	Laboratorio Central de Salud Publica de Paraguay	Marta Giovanetti et al
EPI_ISL_1492649	Laboratorio Central de Salud Publica de Paraguay	Laboratorio Central de Salud Publica de Paraguay	Marta Giovanetti et al
EPI_ISL_1492650	Laboratorio Central de Salud Publica de Paraguay	Laboratorio Central de Salud Publica de Paraguay	Marta Giovanetti et al
EPI_ISL_1492651	Laboratorio Central de Salud Publica de Paraguay	Laboratorio Central de Salud Publica de Paraguay	Marta Giovanetti et al

EPI_ISL_1492652	Laboratorio Central de Salud Publica de Paraguay	Laboratorio Central de Salud Publica de Paraguay	Marta Giovanetti et al
EPI_ISL_1492654	Laboratorio Central de Salud Publica de Paraguay	Laboratorio Central de Salud Publica de Paraguay	Marta Giovanetti et al
EPI_ISL_1492657	Laboratorio Central de Salud Publica de Paraguay	Laboratorio Central de Salud Publica de Paraguay	Marta Giovanetti et al
EPI_ISL_1492658	Laboratorio Central de Salud Publica de Paraguay	Laboratorio Central de Salud Publica de Paraguay	Marta Giovanetti et al
EPI_ISL_1492659	Laboratorio Central de Salud Publica de Paraguay	Laboratorio Central de Salud Publica de Paraguay	Marta Giovanetti et al
EPI_ISL_1492660	Laboratorio Central de Salud Publica de Paraguay	Laboratorio Central de Salud Publica de Paraguay	Marta Giovanetti et al
EPI_ISL_1492661	Laboratorio Central de Salud Publica de Paraguay	Laboratorio Central de Salud Publica de Paraguay	Marta Giovanetti et al
EPI_ISL_1492662	Laboratorio Central de Salud Publica de Paraguay	Laboratorio Central de Salud Publica de Paraguay	Marta Giovanetti et al
EPI_ISL_1492639	Laboratorio Central de Salud Publica de Paraguay	Laboratorio Central de Salud Publica de Paraguay	Marta Giovanetti et al
EPI_ISL_1492670	Laboratorio Central de Salud Publica de Paraguay	Laboratorio Central de Salud Publica de Paraguay	Marta Giovanetti et al
EPI_ISL_1492640	Laboratorio Central de Salud Publica de Paraguay	Laboratorio Central de Salud Publica de Paraguay	Marta Giovanetti et al
EPI_ISL_1492676	Laboratorio Central de Salud Publica de Paraguay	Laboratorio Central de Salud Publica de Paraguay	Marta Giovanetti et al
EPI_ISL_1492677	Laboratorio Central de Salud Publica de Paraguay	Laboratorio Central de Salud Publica de Paraguay	Marta Giovanetti et al
EPI_ISL_1492641	Laboratorio Central de Salud Publica de Paraguay	Laboratorio Central de Salud Publica de Paraguay	Marta Giovanetti et al
EPI_ISL_1492642	Laboratorio Central de Salud Publica de Paraguay	Laboratorio Central de Salud Publica de Paraguay	Marta Giovanetti et al
EPI_ISL_1492643	Laboratorio Central de Salud Publica de Paraguay	Laboratorio Central de Salud Publica de Paraguay	Marta Giovanetti et al
EPI_ISL_1492644	Laboratorio Central de Salud Publica de Paraguay	Laboratorio Central de Salud Publica de Paraguay	Marta Giovanetti et al
EPI_ISL_1492645	Laboratorio Central de Salud Publica de Paraguay	Laboratorio Central de Salud Publica de Paraguay	Marta Giovanetti et al
EPI_ISL_436322	Servicio de Microbiologia. Hospital Clinico Universitario de Valencia	Sequencing and Bioinformatics Service and Molecular Epidemiology Research Group. FISABIO-Public Health	Marta Pla Diaz et al
EPI_ISL_1585338	Laboratorio di Microbiologia	Laboratorio di Microbiologia	Martinetti Lucchini Gladys et al
EPI_ISL_1163532	LABCOVID_HCPA	LABRESIS_HCPA	Martins AF et al
EPI_ISL_1163530	LABCOVID_HCPA	LABRESIS_HCPA	Martins AF et al
EPI_ISL_1163736	LABCOVID_HCPA	LABCOVID_HCPA	Martins AF et al
EPI_ISL_1163737	LABCOVID_HCPA	LABRESIS_HCPA	Martins AF et al
EPI_ISL_1133128	LABCOVID_HCPA	LABRESIS_HCPA	Martins AF et al
EPI_ISL_1163694	LABCOVID_HCPA	LABRESIS_HCPA	Martins AF et al
EPI_ISL_1133129	LABCOVID_HCPA	LABRESIS_HCPA	Martins AF et al
EPI_ISL_1163695	LABCOVID_HCPA	LABRESIS_HCPA	Martins AF et al
EPI_ISL_1133130	LABCOVID_HCPA	LABRESIS_HCPA	Martins AF et al
EPI_ISL_1163696	LABCOVID_HCPA	LABRESIS_HCPA	Martins AF et al

EPI_ISL_1133131	LABCOVID_HCPA	LABRESIS_HCPA	Martins AF et al
EPI_ISL_1133132	LABCOVID_HCPA	LABRESIS_HCPA	Martins AF et al
EPI_ISL_1163697	LABCOVID_HCPA	LABRESIS_HCPA	Martins AF et al
EPI_ISL_1161401	LABCOVID_HCPA	LABRESIS_HCPA	Martins AF et al
EPI_ISL_1163698	LABCOVID_HCPA	LABRESIS_HCPA	Martins AF et al
EPI_ISL_1163699	LABCOVID_HCPA	LABRESIS_HCPA	Martins AF et al
EPI_ISL_1133133	LABCOVID_HCPA	LABRESIS_HCPA	Martins AF et al
EPI_ISL_1161402	LABCOVID_HCPA	LABRESIS_HCPA	Martins AF et al
EPI_ISL_1163700	LABCOVID_HCPA	LABRESIS_HCPA	Martins AF et al
EPI_ISL_1161403	LABCOVID_HCPA	LABRESIS_HCPA	Martins AF et al
EPI_ISL_1163701	LABCOVID_HCPA	LABRESIS_HCPA	Martins AF et al
EPI_ISL_1133120	LABCOVID_HCPA	LABRESIS_HCPA	Martins AF et al
EPI_ISL_1163702	LABCOVID_HCPA	LABRESIS_HCPA	Martins AF et al
EPI_ISL_1163703	LABCOVID_HCPA	LABRESIS_HCPA	Martins AF et al
EPI_ISL_1163704	LABCOVID_HCPA	LABRESIS_HCPA	Martins AF et al
EPI_ISL_1163705	LABCOVID_HCPA	LABRESIS_HCPA	Martins AF et al
EPI_ISL_1163706	LABCOVID_HCPA	LABRESIS_HCPA	Martins AF et al
EPI_ISL_1163707	LABCOVID_HCPA	LABRESIS_HCPA	Martins AF et al
EPI_ISL_1133134	LABCOVID_HCPA	LABRESIS_HCPA	Martins AF et al
EPI_ISL_1163708	LABCOVID_HCPA	LABRESIS_HCPA	Martins AF et al
EPI_ISL_1163709	LABCOVID_HCPA	LABRESIS_HCPA	Martins AF et al
EPI_ISL_1163710	LABCOVID_HCPA	LABRESIS_HCPA	Martins AF et al
EPI_ISL_1163711	LABCOVID_HCPA	LABRESIS_HCPA	Martins AF et al
EPI_ISL_1133135	LABCOVID_HCPA	LABRESIS_HCPA	Martins AF et al
EPI_ISL_1161413	LABCOVID_HCPA	LABRESIS_HCPA	Martins AF et al
EPI_ISL_1163712	LABCOVID_HCPA	LABRESIS_HCPA	Martins AF et al
EPI_ISL_1161414	LABCOVID_HCPA	LABRESIS_HCPA	Martins AF et al
EPI_ISL_1163713	LABCOVID_HCPA	LABRESIS_HCPA	Martins AF et al
EPI_ISL_1133136	LABCOVID_HCPA	LABRESIS_HCPA	Martins AF et al
EPI_ISL_1133137	LABCOVID_HCPA	LABRESIS_HCPA	Martins AF et al

EPI_ISL_1133138	LABCOVID_HCPA	LABRESIS_HCPA	Martins AF et al
EPI_ISL_1163714	LABCOVID_HCPA	LABRESIS_HCPA	Martins AF et al
EPI_ISL_1133139	LABCOVID_HCPA	LABRESIS_HCPA	Martins AF et al
EPI_ISL_1163715	LABCOVID_HCPA	LABRESIS_HCPA	Martins AF et al
EPI_ISL_1163716	LABCOVID_HCPA	LABRESIS_HCPA	Martins AF et al
EPI_ISL_1133140	LABCOVID_HCPA	LABRESIS_HCPA	Martins AF et al
EPI_ISL_1133121	LABCOVID_HCPA	LABRESIS_HCPA	Martins AF et al
EPI_ISL_1161415	LABCOVID_HCPA	LABRESIS_HCPA	Martins AF et al
EPI_ISL_1133141	LABCOVID_HCPA	LABRESIS_HCPA	Martins AF et al
EPI_ISL_1133142	LABCOVID_HCPA	LABRESIS_HCPA	Martins AF et al
EPI_ISL_1133143	LABCOVID_HCPA	LABRESIS_HCPA	Martins AF et al
EPI_ISL_1133144	LABCOVID_HCPA	LABRESIS_HCPA	Martins AF et al
EPI_ISL_1133122	LABCOVID_HCPA	LABRESIS_HCPA	Martins AF et al
EPI_ISL_1133123	LABCOVID_HCPA	LABRESIS_HCPA	Martins AF et al
EPI_ISL_1133124	LABCOVID_HCPA	LABRESIS_HCPA	Martins AF et al
EPI_ISL_1133125	LABCOVID_HCPA	LABRESIS_HCPA	Martins AF et al
EPI_ISL_1133126	LABCOVID_HCPA	LABRESIS_HCPA	Martins AF et al
EPI_ISL_1163693	LABCOVID_HCPA	LABRESIS_HCPA	Martins AF et al
EPI_ISL_1133127	LABCOVID_HCPA	LABRESIS_HCPA	Martins AF et al
EPI_ISL_596672	St.Vincent's University Hospital	St.Vincent's University Hospital	Mary Lucey et al
EPI_ISL_757285	Department of Virology, Public Health Laboratories Division	Department of Virology, Public Health Laboratories Division	Massab Umair et al
EPI_ISL_757286	Department of Virology, Public Health Laboratories Division	Department of Virology, Public Health Laboratories Division	Massab Umair et al
EPI_ISL_468159	unknown	Department of Virology, Public Health Laboratories Division, National Institute of Health	Massab Umair et al
EPI_ISL_1385784	Department of Virology	Department of Virology	Massab Umair et al
EPI_ISL_1385785	Department of Virology	Department of Virology	Massab Umair et al
EPI_ISL_1385786	Department of Virology	Department of Virology	Massab Umair et al
EPI_ISL_1385789	Department of Virology	Department of Virology	Massab Umair et al
EPI_ISL_1397958	Department of Virology	Department of Virology	Massab Umair et al
EPI_ISL_1398039	Department of Virology	Department of Virology	Massab Umair et al
EPI_ISL_1406391	Department of Virology	Department of Virology	Massab Umair et al

EPI_ISL_1406393	Department of Virology	Department of Virology	Massab Umair et al
EPI_ISL_1406394	Department of Virology	Department of Virology	Massab Umair et al
EPI_ISL_1385791	Department of Virology	Department of Virology	Massab Umair et al
EPI_ISL_1385794	Department of Virology	Department of Virology	Massab Umair et al
EPI_ISL_1406399	Department of Virology	Department of Virology	Massab Umair et al
EPI_ISL_1385800	Department of Virology	Department of Virology	Massab Umair et al
EPI_ISL_1385810	Department of Virology	Department of Virology	Massab Umair et al
EPI_ISL_1385812	Department of Virology	Department of Virology	Massab Umair et al
EPI_ISL_468163	Department of Virology, Public Health Laboratories Division, National Institute of Health	Department of Virology, Public Health Laboratories Division, National Institute of Health	Massab Umair et al
EPI_ISL_1372711	Maine Health and Environmental Testing Laboratory	Tewhey Lab, The Jackson Laboratory	Matluk et al
EPI_ISL_1372713	Maine Health and Environmental Testing Laboratory	Tewhey Lab, The Jackson Laboratory	Matluk et al
EPI_ISL_1372731	Maine Health and Environmental Testing Laboratory	Tewhey Lab, The Jackson Laboratory	Matluk et al
EPI_ISL_1372863	Maine Health and Environmental Testing Laboratory	Tewhey Lab, The Jackson Laboratory	Matluk et al
EPI_ISL_417479	Minnesota Department of Health, Public Health Laboratory	Minnesota Department of Health, Public Health Laboratory	Matt Plumb et al
EPI_ISL_417502	Minnesota Department of Health, Public Health Laboratory	Minnesota Department of Health, Public Health Laboratory	Matt Plumb et al
EPI_ISL_413490	Auckland Hospital	Institute of Environmental Science and Research (ESR)	Matt Storey et al
EPI_ISL_456288	Southern Community Labs Dunedin	Institute of Environmental Science and Research (ESR)	Matt Storey et al
EPI_ISL_456291	Southern Community Labs Dunedin	Institute of Environmental Science and Research (ESR)	Matt Storey et al
EPI_ISL_456293	Southern Community Labs Dunedin	Institute of Environmental Science and Research (ESR)	Matt Storey et al
EPI_ISL_456303	Southern Community Labs Dunedin	Institute of Environmental Science and Research (ESR)	Matt Storey et al
EPI_ISL_456313	Southern Community Labs Dunedin	Institute of Environmental Science and Research (ESR)	Matt Storey et al
EPI_ISL_456315	Southern Community Labs Dunedin	Institute of Environmental Science and Research (ESR)	Matt Storey et al
EPI_ISL_456341	Wellington SCL	Institute of Environmental Science and Research (ESR)	Matt Storey et al
EPI_ISL_456354	Canterbury Health Laboratories	Institute of Environmental Science and Research (ESR)	Matt Storey et al
EPI_ISL_871801	AIID	Irish Coronavirus Sequencing Consortium-Teagasc Grange	Matthew McCabe et al
EPI_ISL_871802	AIID	Irish Coronavirus Sequencing Consortium-Teagasc Grange	Matthew McCabe et al
EPI_ISL_602539	Center of Medical Microbiology, Virology, and Hospital Hygiene, University of Duesseldorf	Center of Medical Microbiology, Virology, and Hospital Hygiene, University of Duesseldorf	Maximilian Damagnez et al
EPI_ISL_602515	Center of Medical Microbiology, Virology, and Hospital Hygiene, University of Duesseldorf	Center of Medical Microbiology, Virology, and Hospital Hygiene, University of Duesseldorf	Maximilian Damagnez et al
EPI_ISL_1346939	Center of Medical Microbiology, Virology, and Hospital Hygiene, University of Duesseldorf	Center of Medical Microbiology, Virology, and Hospital Hygiene, University of Duesseldorf	Maximilian Damagnez et al

EPI_ISL_1659701	Swedish national genomic surveillance program of SARS-CoV-2	The Public Health Agency of Sweden	Maximilian Riess et al
EPI_ISL_1659714	Swedish national genomic surveillance program of SARS-CoV-2	The Public Health Agency of Sweden	Maximilian Riess et al
EPI_ISL_1659866	Swedish national genomic surveillance program of SARS-CoV-2	The Public Health Agency of Sweden	Maximilian Riess et al
EPI_ISL_1660117	Swedish national genomic surveillance program of SARS-CoV-2	The Public Health Agency of Sweden	Maximilian Riess et al
EPI_ISL_1499123	1. Glowny Inspektorat Sanitarny. 2. Diagnostyka. Laboratoria Medyczne.	1. ViroGenetics - BSL3 Laboratory of Virology, Małopolska Centre of Biotechnology, Jagiellonian University; 2. genXone SA, Research & Development Laboratory	Mazur-Panasiuk et al
EPI_ISL_1402488	1. Glowny Inspektorat Sanitarny. 2. Diagnostyka. Laboratoria Medyczne.	1. ViroGenetics - BSL3 Laboratory of Virology, Małopolska Centre of Biotechnology, Jagiellonian University; 2. genXone SA, Research & Development Laboratory	Mazur-Panasiuk et al
EPI_ISL_1402519	1. Glowny Inspektorat Sanitarny. 2. Diagnostyka. Laboratoria Medyczne.	1. ViroGenetics - BSL3 Laboratory of Virology, Małopolska Centre of Biotechnology, Jagiellonian University; 2. genXone SA, Research & Development Laboratory	Mazur-Panasiuk et al
EPI_ISL_1402524	1. Glowny Inspektorat Sanitarny. 2. Diagnostyka. Laboratoria Medyczne.	1. ViroGenetics - BSL3 Laboratory of Virology, Małopolska Centre of Biotechnology, Jagiellonian University; 2. genXone SA, Research & Development Laboratory	Mazur-Panasiuk et al
EPI_ISL_1402537	1. Glowny Inspektorat Sanitarny. 2. Diagnostyka. Laboratoria Medyczne.	1. ViroGenetics - BSL3 Laboratory of Virology, Małopolska Centre of Biotechnology, Jagiellonian University; 2. genXone SA, Research & Development Laboratory	Mazur-Panasiuk et al
EPI_ISL_1406337	1. Glowny Inspektorat Sanitarny. 2. Diagnostyka. Laboratoria Medyczne.	1. ViroGenetics - BSL3 Laboratory of Virology, Małopolska Centre of Biotechnology, Jagiellonian University; 2. genXone SA, Research & Development Laboratory	Mazur-Panasiuk et al
EPI_ISL_573249	Virology Department, Royal Infirmary of Edinburgh, NHS Lothian / School of Biological Sciences, University of Edinburgh / Institute of Genetics and Molecular Medicine, University of Edinburgh	COVID-19 Genomics UK (COG-UK) Consortium	McHugh M et al
EPI_ISL_1104214	Virology Department, Royal Infirmary of Edinburgh, NHS Lothian / School of Biological Sciences, University of Edinburgh	COVID-19 Genomics UK (COG-UK) Consortium	McHugh M et al
EPI_ISL_1104216	Virology Department, Royal Infirmary of Edinburgh, NHS Lothian / School of Biological Sciences, University of Edinburgh	COVID-19 Genomics UK (COG-UK) Consortium	McHugh M et al
EPI_ISL_1636703	Virology Department, Royal Infirmary of Edinburgh, NHS Lothian / School of Biological Sciences, University of Edinburgh	COVID-19 Genomics UK (COG-UK) Consortium	McHugh M et al
EPI_ISL_478249	Virology Department, Royal Infirmary of Edinburgh, NHS Lothian / School of Biological Sciences, University of Edinburgh / Institute of Genetics and Molecular Medicine, University of Edinburgh	COVID-19 Genomics UK (COG-UK) Consortium	McHugh M et al
EPI_ISL_1494726	Centro de Investigación en Ciencias de la Salud y Biomedicina, U.A.S.LP.	Centro de Investigación en Ciencias de la Salud y Biomedicina, U.A.S.LP.	MD PhD Sofía Bernal Silva et al
EPI_ISL_943566	National Institute of Laboratory Medicine and Referral Center	Genomic Research Lab, BCSIR	Md. Ahashan Habib et al
EPI_ISL_747283	DNA SOLUTION LTD.	DNA SOLUTION LTD.	Md. Imran Khan et al
EPI_ISL_774897	Designated Reference Institute for Chemical Measurements (DRICM)	DNA SOLUTION LTD.	Md. Imran Khan et al
EPI_ISL_774894	Designated Reference Institute for Chemical Measurements (DRICM)	DNA SOLUTION LTD.	Md. Imran Khan et al
EPI_ISL_774898	Designated Reference Institute for Chemical Measurements (DRICM)	DNA SOLUTION LTD.	Md. Imran Khan et al
EPI_ISL_775000	Designated Reference Institute for Chemical Measurements (DRICM)	DNA SOLUTION LTD.	Md. Imran Khan et al

EPI_ISL_774979	Designated Reference Institute for Chemical Measurements (DRICM)	DNA SOLUTION LTD.	Md. Imran Khan et al
EPI_ISL_735496	Chattogram Veterinary and Animal Sciences University	Central Biological Research Laboratory and Department of Biochemistry and Molecular Biology	Md. Imranul Hoq et al
EPI_ISL_959309	National Institute of Laboratory Medicine and Referral Center	Genomic Research Lab, BCSIR	Md. Maruf Ahmed Molla et al
EPI_ISL_447904	National Institute of Biotechnology	National Institute of Biotechnology	Md. Moniruzzaman et al
EPI_ISL_1585269	Armed Forces Institute of Pathology (AFIP), Dhaka Cantonment	Genomic Research Lab, BCSIR	Md. Murshed Hasan Sarkar et al
EPI_ISL_1636521	DNA Solution Ltd.	Genomic Research Lab, BCSIR	Md. Murshed Hasan Sarkar et al
EPI_ISL_1636522	DNA Solution Ltd.	Genomic Research Lab, BCSIR	Md. Murshed Hasan Sarkar et al
EPI_ISL_1493059	National Institute of Laboratory Medicine and Referral Center	Bangladesh Council of Scientific and Industrial Research	Md. Murshed Hasan Sarkar et al
EPI_ISL_1033158	National Institute of Laboratory Medicine and Referral Center	Genomic Research Lab, BCSIR	Md. Murshed Hasan Sarkar et al
EPI_ISL_1508950	National Institute of Laboratory Medicine and Referral Center	Genomic Research Lab, BCSIR	Md. Murshed Hasan Sarkar et al
EPI_ISL_1542073	Department of Genetic Engineering and Biotechnology, Shahjalal University of Science and Technology	Genomic Research Lab, BCSIR	Md. Murshed Hasan Sarkar et al
EPI_ISL_1563631	Department of Genetic Engineering and Biotechnology, Shahjalal University of Science and Technology	Genomic Research Lab, BCSIR	Md. Shamsul Haque Prodhan et al
EPI_ISL_430456	Rizal Medical Center	Research Institute for Tropical Medicine	Medado et al
EPI_ISL_430845	Pasig City General Hospital	Research Institute for Tropical Medicine	Medado et al
EPI_ISL_610245	Virology, National Institute for Biological Standards and Control	Virology, National Institute for Biological Standards and Control	Mee et al
EPI_ISL_455719	T.C. Sağlık Bakanlığı Adıyaman İl Sağlık Müdürlüğü Adıyaman Eğitim Ve Araştırma Hastanesi	VETAL Animal Health Products Company, BSL3+ Production Laboratuary /Turkey	Mehmet Turgut et al
EPI_ISL_418241	NIC Viral Respiratory Unit - Institut Pasteur of Algeria	National Reference Center for Viruses of Respiratory Infections, Institut Pasteur, Paris	Mélanie Albert et al
EPI_ISL_418242	NIC Viral Respiratory Unit - Institut Pasteur of Algeria	National Reference Center for Viruses of Respiratory Infections, Institut Pasteur, Paris	Mélanie Albert et al
EPI_ISL_420037	NIC Viral Respiratory Unit - Institut Pasteur of Algeria	National Reference Center for Viruses of Respiratory Infections, Institut Pasteur, Paris	Mélanie Albert et al
EPI_ISL_766864	NIC Viral Respiratory Unit - Institut Pasteur of Algeria	National Reference Center for Viruses of Respiratory Infections, Institut Pasteur, Paris	Mélanie Albert et al
EPI_ISL_766867	NIC Viral Respiratory Unit - Institut Pasteur of Algeria	National Reference Center for Viruses of Respiratory Infections, Institut Pasteur, Paris	Mélanie Albert et al
EPI_ISL_766874	NIC Viral Respiratory Unit - Institut Pasteur of Algeria	National Reference Center for Viruses of Respiratory Infections, Institut Pasteur, Paris	Mélanie Albert et al
EPI_ISL_406597	Department of Infectious and Tropical Diseases, Bichat Claude Bernard Hospital, Paris	National Reference Center for Viruses of Respiratory Infections, Institut Pasteur, Paris	Mélanie Albert et al
EPI_ISL_410984	Department of Infectious and Tropical Diseases, Bichat Claude Bernard Hospital, Paris	National Reference Center for Viruses of Respiratory Infections, Institut Pasteur, Paris	Mélanie Albert et al
EPI_ISL_408430	Department of Infectious and Tropical Diseases, Bichat Claude Bernard Hospital, Paris	National Reference Center for Viruses of Respiratory Infections, Institut Pasteur, Paris	Mélanie Albert et al

EPI_ISL_414623	Laboratoire de Virologie Institut de Virologie - INSERM U 1109 Hôpitaux Universitaires de Strasbourg	National Reference Center for Viruses of Respiratory Infections, Institut Pasteur, Paris	Mélnie Albert et al
EPI_ISL_414600	Laboratoire de Virologie Institut de Virologie - INSERM U 1109 Hôpitaux Universitaires de Strasbourg	National Reference Center for Viruses of Respiratory Infections, Institut Pasteur, Paris	Mélnie Albert et al
EPI_ISL_1296252	Università degli Studi di Perugia	Istituto Zooprofilattico Sperimentale dell'Abruzzo e Molise "G. Caporale"	Mencacci A et al
EPI_ISL_1670647	Università degli Studi di Perugia	Istituto Zooprofilattico Sperimentale dell'Abruzzo e Molise "G. Caporale"	Mencacci A et al
EPI_ISL_500168	Hospital Universitario Virgen de las Nieves de Granada-SAS	SeqCOVID-SPAIN consortium/IBV(CSIC)	Mercedes Pérez Ruiz et al
EPI_ISL_471529	Department for Virology, Molecular Biology and Genome Research, R. G. Lugar Center for Public Health Research, National Center for Disease Control and Public Health (NCDC) of Georgia.	Department for Virology, Molecular Biology and Genome Research, R. G. Lugar Center for Public Health Research, National Center for Disease Control and Public Health (NCDC) of Georgia.	Meri Pantsulaia et al
EPI_ISL_754181	Department for Virology, Molecular Biology and Genome Research, R. G. Lugar Center for Public Health Research, National Center for Disease Control and Public Health (NCDC) of Georgia.	Department for Virology, Molecular Biology and Genome Research, R. G. Lugar Center for Public Health Research, National Center for Disease Control and Public Health (NCDC) of Georgia.	Meri Pantsulaia et al
EPI_ISL_469051	LNR National Reference Laboratory, Mohammed VI University of Health Sciences	Medical Biotechnology Laboratory, Rabat Medical and Pharmacy School, Mohammed The Vth University in Rabat	Meriem LAAMARTI et al
EPI_ISL_482739	LNR National Reference Laboratory, Mohammed VI University of Health Sciences	Medical Biotechnology Laboratory, Rabat Medical and Pharmacy School, Mohammed The Vth University in Rabat	Meriem LAAMARTI et al
EPI_ISL_482740	LNR National Reference Laboratory, Mohammed VI University of Health Sciences	Medical Biotechnology Laboratory, Rabat Medical and Pharmacy School, Mohammed The Vth University in Rabat	Meriem LAAMARTI et al
EPI_ISL_1061413	Istanbul University-Cerrahpasa, Cerrahpasa School of Medicine, COVID-19 Laboratory	Istanbul University-Cerrahpasa, Cerrahpasa School of Medicine, COVID-19 Laboratory	Mert Ahmet Kuskucu et al
EPI_ISL_1662234	IU-Cerrahpasa, Cerrahpasa School of Medicine, COVID-19 Lab	IU-Cerrahpasa, Cerrahpasa School of Medicine, COVID-19 Lab	Mert Kuskucu et al
EPI_ISL_1662593	IU-Cerrahpasa, Cerrahpasa School of Medicine, COVID-19 Lab	IU-Cerrahpasa, Cerrahpasa School of Medicine, COVID-19 Lab	Mert Kuskucu et al
EPI_ISL_1663062	IU-Cerrahpasa, Cerrahpasa School of Medicine, COVID-19 Lab	Illumina Miseq	Mert Kuskucu et al
EPI_ISL_1663581	IU-Cerrahpasa, Cerrahpasa School of Medicine, COVID-19 Lab	IU-Cerrahpasa, Cerrahpasa School of Medicine, COVID-19 Lab	Mert Kuskucu et al
EPI_ISL_959706	National Virus Reference Laboratory	National Virus Reference Laboratory	Michael Carr et al
EPI_ISL_644338	CEPHR / Vincent's Hospital	Irish Coronavirus Sequencing Consortium - National Virus Reference Laboratory	Michael Carr et al
EPI_ISL_528470	National Virus Reference Laboratory	National Virus Reference Laboratory	Michael Carr et al
EPI_ISL_732464	National Virus Reference Laboratory	National Virus Reference Laboratory	Michael Carr et al
EPI_ISL_501261	National Virus Reference Laboratory	National Virus Reference Laboratory	Michael Carr et al
EPI_ISL_500592	National Virus Reference Laboratory	National Virus Reference Laboratory	Michael Carr et al
EPI_ISL_528465	National Virus Reference Laboratory	National Virus Reference Laboratory	Michael Carr et al
EPI_ISL_837362	National Virus Reference Laboratory	National Virus Reference Laboratory	Michael Carr et al
EPI_ISL_875351	National Virus Reference Laboratory	National Virus Reference Laboratory	Michael Carr et al
EPI_ISL_644214	CEPHR / Vincent's Hospital	Irish Coronavirus Sequencing Consortium - National Virus Reference Laboratory	Michael Carr et al

EPI_ISL_671903	National Virus Reference Laboratory	National Virus Reference Laboratory	Michael Carr et al
EPI_ISL_512106	National Virus Reference Laboratory	National Virus Reference Laboratory	Michael Carr et al
EPI_ISL_525363	National Virus Reference Laboratory	National Virus Reference Laboratory	Michael Carr et al
EPI_ISL_644213	CEPHR / Vincent's Hospital	Irish Coronavirus Sequencing Consortium - National Virus Reference Laboratory	Michael Carr et al
EPI_ISL_501260	National Virus Reference Laboratory	National Virus Reference Laboratory	Michael Carr et al
EPI_ISL_671921	National Virus Reference Laboratory	National Virus Reference Laboratory	Michael Carr et al
EPI_ISL_639900	National Virus Reference Laboratory	National Virus Reference Laboratory	Michael Carr et al
EPI_ISL_639899	National Virus Reference Laboratory	National Virus Reference Laboratory	Michael Carr et al
EPI_ISL_639898	National Virus Reference Laboratory	National Virus Reference Laboratory	Michael Carr et al
EPI_ISL_639906	National Virus Reference Laboratory	National Virus Reference Laboratory	Michael Carr et al
EPI_ISL_639892	National Virus Reference Laboratory	National Virus Reference Laboratory	Michael Carr et al
EPI_ISL_639903	National Virus Reference Laboratory	National Virus Reference Laboratory	Michael Carr et al
EPI_ISL_639831	National Virus Reference Laboratory	National Virus Reference Laboratory	Michael Carr et al
EPI_ISL_525354	National Virus Reference Laboratory	National Virus Reference Laboratory	Michael Carr et al
EPI_ISL_528453	National Virus Reference Laboratory	National Virus Reference Laboratory	Michael Carr et al
EPI_ISL_578235	National Virus Reference Laboratory	National Virus Reference Laboratory	Michael Carr et al
EPI_ISL_752592	National Virus Reference Laboratory	National Virus Reference Laboratory	Michael Carr et al
EPI_ISL_644224	CEPHR / Vincent's Hospital	Irish Coronavirus Sequencing Consortium - National Virus Reference Laboratory	Michael Carr et al
EPI_ISL_1301441	Department of Infectious and Tropical Diseases, Bichat Claude Bernard Hospital, Paris	NBACC	Michael Schuit et al
EPI_ISL_708512	National Virus Reference Laboratory	Irish Coronavirus Sequencing Consortium - Teagasc Oakpark	Michele Della Bartola et al
EPI_ISL_708519	National Virus Reference Laboratory	Irish Coronavirus Sequencing Consortium - Teagasc Oakpark	Michele Della Bartola et al
EPI_ISL_861712	Laboratory for Respiratory Viruses, Cantacuzino National Military-Medical Institute for Research and Development	Cantacuzino Institute Virology	Mihaela Lazar et al
EPI_ISL_861713	Laboratory for Respiratory Viruses, Cantacuzino National Military-Medical Institute for Research and Development	Cantacuzino Institute Virology	Mihaela Lazar et al
EPI_ISL_861711	Laboratory for Respiratory Viruses, Cantacuzino National Military-Medical Institute for Research and Development	Cantacuzino Institute Virology	Mihaela Lazar et al
EPI_ISL_1663578	Fimlab Laboratories	Fimlab Laboratories	Minna Paloniemi et al
EPI_ISL_1663568	Fimlab Laboratories	Fimlab Laboratories	Minna Paloniemi et al
EPI_ISL_1663575	Fimlab Laboratories	Fimlab Laboratories	Minna Paloniemi et al
EPI_ISL_1072985	University of Balamand	Microbial Genomics Lab, Lebanese American University, Byblos	Mira El Chaar et al

EPI_ISL_978545	Centre de Virologie des Maladies infectueuses Tropicales	Functional Genomic Platform UATRS-biology, CNRST	Mly Abdelaziz ELALAOUI et al
EPI_ISL_430819	Center of Scientific Excellence for Influenza Viruses,National Research Centre (NRC), Egypt.	Center of Scientific Excellence for Influenza Viruses,National Research Centre (NRC), Egypt.	Mohamed Ahmed Ali et al
EPI_ISL_442044	Kawsar Human Genetic Research Center	Kawsar Human Genetic Research Center	Mohammad Ali Khosravi et al
EPI_ISL_904015	Molecular Biology and Virology lab, Faculty of Veterinary Medicine, Jordan University of Science and Technology	Molecular Biology and Virology lab, Faculty of Veterinary Medicine, Jordan University of Science and Technology	Mohammad Hussien Alboom et al
EPI_ISL_904016	Molecular Biology and Virology lab, Faculty of Veterinary Medicine, Jordan University of Science and Technology	Molecular Biology and Virology lab, Faculty of Veterinary Medicine, Jordan University of Science and Technology	Mohammad Hussien Alboom et al
EPI_ISL_1593857	Armed Forces Institute of Pathology (AFIP), Dhaka Cantonment	Genomic Research Lab, BCSIR	Mohammad Mizanur Rahman et al
EPI_ISL_1133253	University of Chittagong	Central Biological Research Laboratory and Department of Biochemistry and Molecular Biology	Mohammad Omar Faruque et al
EPI_ISL_977585	Department of Medical Microbiology, Hospital Pengajar Universiti Putra Malaysia	Malaysia Genome Institute	Mohd Noor Mat Isa et al
EPI_ISL_861725	Department of Medical Microbiology, Hospital Pengajar Universiti Putra Malaysia	Malaysia Genome Institute	Mohd Noor Mat Isa et al
EPI_ISL_861726	Department of Medical Microbiology, Hospital Pengajar Universiti Putra Malaysia	Malaysia Genome Institute	Mohd Noor Mat Isa et al
EPI_ISL_455793	Institute for Medical Research, Infectious Disease Research Centre, National Institutes of Health, Ministry of Health Malaysia	Malaysia Genome Institute	Mohd Noor Mat Isa et al
EPI_ISL_455790	Institute for Medical Research, Infectious Disease Research Centre, National Institutes of Health, Ministry of Health Malaysia	Malaysia Genome Institute	Mohd Noor Mat Isa et al
EPI_ISL_455791	Institute for Medical Research, Infectious Disease Research Centre, National Institutes of Health, Ministry of Health Malaysia	Malaysia Genome Institute	Mohd Noor Mat Isa et al
EPI_ISL_455792	Institute for Medical Research, Infectious Disease Research Centre, National Institutes of Health, Ministry of Health Malaysia	Malaysia Genome Institute	Mohd Noor Mat Isa et al
EPI_ISL_582124	Malaysia Genome Institute	Malaysia Genome Institute	Mohd Noor Mat Isa et al
EPI_ISL_528743	Malaysia Genome Institute	Malaysia Genome Institute	Mohd Noor Mat Isa et al
EPI_ISL_528744	Malaysia Genome Institute	Malaysia Genome Institute	Mohd Noor Mat Isa et al
EPI_ISL_416829	National Public Health Laboratory	Malaysia Genome Institute	Mohd Noor Mat Isa et al
EPI_ISL_416866	National Public Health Laboratory	Malaysia Genome Institute	Mohd Noor Mat Isa et al
EPI_ISL_416885	National Public Health Laboratory	Malaysia Genome Institute	Mohd Noor Mat Isa et al
EPI_ISL_416886	National Public Health Laboratory	Malaysia Genome Institute	Mohd Noor Mat Isa et al
EPI_ISL_416907	National Public Health Laboratory	Malaysia Genome Institute	Mohd Noor Mat Isa et al
EPI_ISL_807153	Makmal Kesihatan Awam Kebangsaan, Kementerian Kesihatan Malaysia	Integrative Pharmacogenomics Institute (iPROMISE)	Mohd Zaki Salleh et al
EPI_ISL_807152	Makmal Kesihatan Awam Kebangsaan, Kementerian Kesihatan Malaysia	Integrative Pharmacogenomics Institute (iPROMISE)	Mohd Zaki Salleh et al
EPI_ISL_407071	Respiratory Virus Unit, Microbiology Services Colindale, Public Health England	Respiratory Virus Unit, Microbiology Services Colindale, Public Health England	Monica Galiano et al

EPI_ISL_407073	Respiratory Virus Unit, Microbiology Services Colindale, Public Health England	Respiratory Virus Unit, Microbiology Services Colindale, Public Health England	Monica Galiano et al
EPI_ISL_414522	Respiratory Virus Unit, Microbiology Services Colindale, Public Health England	Respiratory Virus Unit, Microbiology Services Colindale, Public Health England	Monica Galiano et al
EPI_ISL_424154	Respiratory Virus Unit, Microbiology Services Colindale, Public Health England	Respiratory Virus Unit, Microbiology Services Colindale, Public Health England	Monica Galiano et al
EPI_ISL_1589576	Hospital Universitario Marqués de Valdecilla - IDIVAL (Santander, Cantabria)	SeqCOVID-SPAIN consortium/IBV(CSIC)	Mónica Gozalo Margüello et al
EPI_ISL_1133186	Molecular Virology Unit, Microbiology and Virology Depertment, Fondazione IRCCS Policlinico San Matteo, Pavia	Molecular Virology Unit, Microbiology and Virology Depertment, Fondazione IRCCS Policlinico San Matteo, Pavia	Monica Tallarita et al
EPI_ISL_1133197	Molecular Virology Unit, Microbiology and Virology Depertment, Fondazione IRCCS Policlinico San Matteo, Pavia	Molecular Virology Unit, Microbiology and Virology Depertment, Fondazione IRCCS Policlinico San Matteo, Pavia	Monica Tallarita et al
EPI_ISL_1133201	Molecular Virology Unit, Microbiology and Virology Depertment, Fondazione IRCCS Policlinico San Matteo, Pavia	Molecular Virology Unit, Microbiology and Virology Depertment, Fondazione IRCCS Policlinico San Matteo, Pavia	Monica Tallarita et al
EPI_ISL_1133202	Molecular Virology Unit, Microbiology and Virology Depertment, Fondazione IRCCS Policlinico San Matteo, Pavia	Molecular Virology Unit, Microbiology and Virology Depertment, Fondazione IRCCS Policlinico San Matteo, Pavia	Monica Tallarita et al
EPI_ISL_417877	Institute of Virology, Biomedical Research Center of the Slovak Academy of Sciences, Bratislava; Public Health Authority of the Slovak Republic, Bratislava	Institute of Virology, Biomedical Research Center of the Slovak Academy of Sciences, Bratislava; Comenius University Science Park, Bratislava	Monika Slávikova et al
EPI_ISL_417878	Institute of Virology, Biomedical Research Center of the Slovak Academy of Sciences, Bratislava; Public Health Authority of the Slovak Republic, Bratislava	Institute of Virology, Biomedical Research Center of the Slovak Academy of Sciences, Bratislava; Comenius University Science Park, Bratislava	Monika Slávikova et al
EPI_ISL_417879	Institute of Virology, Biomedical Research Center of the Slovak Academy of Sciences, Bratislava; Public Health Authority of the Slovak Republic, Bratislava	Institute of Virology, Biomedical Research Center of the Slovak Academy of Sciences, Bratislava; Comenius University Science Park, Bratislava	Monika Slávikova et al
EPI_ISL_417880	Institute of Virology, Biomedical Research Center of the Slovak Academy of Sciences, Bratislava; Public Health Authority of the Slovak Republic, Bratislava	Institute of Virology, Biomedical Research Center of the Slovak Academy of Sciences, Bratislava; Comenius University Science Park, Bratislava	Monika Slávikova et al
EPI_ISL_1347884	Instituto Nacional de Investigação em Saúde	KRISP, KZN Research Innovation and Sequencing Platform	Morais J et al
EPI_ISL_1347887	Instituto Nacional de Investigação em Saúde	KRISP, KZN Research Innovation and Sequencing Platform	Morais J et al
EPI_ISL_1347896	Instituto Nacional de Investigação em Saúde	KRISP, KZN Research Innovation and Sequencing Platform	Morais J et al
EPI_ISL_1347897	Instituto Nacional de Investigação em Saúde	KRISP, KZN Research Innovation and Sequencing Platform	Morais J et al
EPI_ISL_1347898	Instituto Nacional de Investigação em Saúde	KRISP, KZN Research Innovation and Sequencing Platform	Morais J et al
EPI_ISL_1347910	Instituto Nacional de Investigação em Saúde	KRISP, KZN Research Innovation and Sequencing Platform	Morais J et al
EPI_ISL_1347935	Instituto Nacional de Investigação em Saúde	KRISP, KZN Research Innovation and Sequencing Platform	Morais J et al
EPI_ISL_1347937	Instituto Nacional de Investigação em Saúde	KRISP, KZN Research Innovation and Sequencing Platform	Morais J et al
EPI_ISL_1347940	Instituto Nacional de Investigação em Saúde	KRISP, KZN Research Innovation and Sequencing Platform	Morais J et al
EPI_ISL_1347941	Instituto Nacional de Investigação em Saúde	KRISP, KZN Research Innovation and Sequencing Platform	Morais J et al
EPI_ISL_1347942	Instituto Nacional de Investigação em Saúde	KRISP, KZN Research Innovation and Sequencing Platform	Morais J et al
EPI_ISL_1545290	Instituto Nacional de Investigação em Saúde	KRISP, KZN Research Innovation and Sequencing Platform	Morais J et al
EPI_ISL_1545304	Instituto Nacional de Investigação em Saúde	KRISP, KZN Research Innovation and Sequencing Platform	Morais J et al
EPI_ISL_1545307	Instituto Nacional de Investigação em Saúde	KRISP, KZN Research Innovation and Sequencing Platform	Morais J et al

EPI_ISL_1545308	Instituto Nacional de Investigação em Saúde	KRISP, KZN Research Innovation and Sequencing Platform	Morais J et al
EPI_ISL_1545309	Instituto Nacional de Investigação em Saúde	KRISP, KZN Research Innovation and Sequencing Platform	Morais J et al
EPI_ISL_1545313	Instituto Nacional de Investigação em Saúde	KRISP, KZN Research Innovation and Sequencing Platform	Morais J et al
EPI_ISL_1545321	Instituto Nacional de Investigação em Saúde	KRISP, KZN Research Innovation and Sequencing Platform	Morais J et al
EPI_ISL_1545323	Instituto Nacional de Investigação em Saúde	KRISP, KZN Research Innovation and Sequencing Platform	Morais J et al
EPI_ISL_1545325	Instituto Nacional de Investigação em Saúde	KRISP, KZN Research Innovation and Sequencing Platform	Morais J et al
EPI_ISL_1545326	Instituto Nacional de Investigação em Saúde	KRISP, KZN Research Innovation and Sequencing Platform	Morais J et al
EPI_ISL_1545353	Instituto Nacional de Investigação em Saúde	KRISP, KZN Research Innovation and Sequencing Platform	Morais J et al
EPI_ISL_1545370	Instituto Nacional de Investigação em Saúde	KRISP, KZN Research Innovation and Sequencing Platform	Morais J et al
EPI_ISL_1545372	Instituto Nacional de Investigação em Saúde	KRISP, KZN Research Innovation and Sequencing Platform	Morais J et al
EPI_ISL_944663	Department of Biochemistry, Cell and Molecular Biology, West African Centre for Cell Biology of Infectious Pathogens (WACCBIP), University of Ghana	Department of Biochemistry, Cell and Molecular Biology, West African Centre for Cell Biology of Infectious Pathogens (WACCBIP), University of Ghana	Morang'a et al
EPI_ISL_944662	Department of Biochemistry, Cell and Molecular Biology, West African Centre for Cell Biology of Infectious Pathogens (WACCBIP), University of Ghana	Department of Biochemistry, Cell and Molecular Biology, West African Centre for Cell Biology of Infectious Pathogens (WACCBIP), University of Ghana	Morang'a et al
EPI_ISL_944735	Department of Biochemistry, Cell and Molecular Biology, West African Centre for Cell Biology of Infectious Pathogens (WACCBIP), University of Ghana	Department of Biochemistry, Cell and Molecular Biology, West African Centre for Cell Biology of Infectious Pathogens (WACCBIP), University of Ghana	Morang'a et al
EPI_ISL_416142	Department of Virus and Microbiological Special diagnostics, Statens Serum Institut, Copenhagen, Denmark.	Statens Serum Institute	Morten Rasmussen et al
EPI_ISL_416143	Department of Virus and Microbiological Special diagnostics, Statens Serum Institut, Copenhagen, Denmark.	ViFU	Morten Rasmussen et al
EPI_ISL_769863	Laboratoire Biolife	Laboratoire de Biotechnologie	Mouna Ouadghiri et al
EPI_ISL_775221	Laboratoire Biolife	Laboratoire de Biotechnologie	Mouna Ouadghiri et al
EPI_ISL_775256	Laboratoire Biolife	Laboratoire de Biotechnologie	Mouna Ouadghiri et al
EPI_ISL_775258	Laboratoire Biolife	Laboratoire de Biotechnologie	Mouna Ouadghiri et al
EPI_ISL_775263	Laboratoire Biolife	Laboratoire de Biotechnologie	Mouna Ouadghiri et al
EPI_ISL_1415293	Institut National d'hygiène	Unité Mixte Internationale TransVIHMI (UMI 233 IRD – U1175 INSERM - Université de Montpellier) IRD (Institut de recherche pour le développement)	Mounerou SALOU et al
EPI_ISL_1440879	Institut National d'Hygiène	Unité Mixte Internationale TransVIHMI (UMI 233 IRD – U1175 INSERM - Université de Montpellier) IRD (Institut de recherche pour le développement)	Mounerou SALOU et al
EPI_ISL_1442993	Institut National d'hygiène	Unité Mixte Internationale TransVIHMI (UMI 233 IRD – U1175 INSERM - Université de Montpellier) IRD (Institut de recherche pour le développement)	Mounerou SALOU et al

EPI_ISL_1416192	Institut National d'hygiène	Unité Mixte Internationale TransVIHMI (UMI 233 IRD – U1175 INSERM - Université de Montpellier) IRD (Institut de recherche pour le développement)	Mounerou SALOU et al
EPI_ISL_1416222	Institut National d'hygiène	Unité Mixte Internationale TransVIHMI (UMI 233 IRD – U1175 INSERM - Université de Montpellier) IRD (Institut de recherche pour le développement)	Mounerou SALOU et al
EPI_ISL_1433132	Institut National d'Hygiène	Unité Mixte Internationale TransVIHMI (UMI 233 IRD – U1175 INSERM - Université de Montpellier) IRD (Institut de recherche pour le développement)	Mounerou SALOU et al
EPI_ISL_1434443	Institut National d'Hygiène	Unité Mixte Internationale TransVIHMI (UMI 233 IRD – U1175 INSERM - Université de Montpellier) IRD (Institut de recherche pour le développement)	Mounerou SALOU et al
EPI_ISL_1435763	Institut National d'Hygiène	Unité Mixte Internationale TransVIHMI (UMI 233 IRD – U1175 INSERM - Université de Montpellier) IRD (Institut de recherche pour le développement)	Mounerou SALOU et al
EPI_ISL_1436817	Institut National d'Hygiène	Unité Mixte Internationale TransVIHMI (UMI 233 IRD – U1175 INSERM - Université de Montpellier) IRD (Institut de recherche pour le développement)	Mounerou SALOU et al
EPI_ISL_1303031	Institut National d’Hygiène (INH)	Unité Mixte Internationale TransVIHMI (UMI 233 IRD – U1175 INSERM - Université de Montpellier)IRD (Institut de recherche pour le développement)	Mounerou SALOU et al
EPI_ISL_1443001	Institut National d'hygiène	Unité Mixte Internationale TransVIHMI (UMI 233 IRD – U1175 INSERM - Université de Montpellier) IRD (Institut de recherche pour le développement)	Mounerou SALOU et al
EPI_ISL_1437749	Institut National d'Hygiène	Unité Mixte Internationale TransVIHMI (UMI 233 IRD – U1175 INSERM - Université de Montpellier) IRD (Institut de recherche pour le développement)	Mounerou SALOU et al
EPI_ISL_1439647	Institut National d'Hygiène	Unité Mixte Internationale TransVIHMI (UMI 233 IRD – U1175 INSERM - Université de Montpellier) IRD (Institut de recherche pour le développement)	Mounerou SALOU et al
EPI_ISL_1443002	Institut National d'hygiène	Unité Mixte Internationale TransVIHMI (UMI 233 IRD – U1175 INSERM - Université de Montpellier) IRD (Institut de recherche pour le développement)	Mounerou SALOU et al
EPI_ISL_1443003	Institut National d'hygiène	Unité Mixte Internationale TransVIHMI (UMI 233 IRD – U1175 INSERM - Université de Montpellier) IRD (Institut de recherche pour le développement)	Mounerou SALOU et al
EPI_ISL_1445275	Institut National d’Hygiène (INH)	Unité Mixte Internationale TransVIHMI (UMI 233 IRD – U1175 INSERM - Université de Montpellier)IRD (Institut de recherche pour le développement)	Mounerou SALOU et al
EPI_ISL_1438551	Institut National d'Hygiène	Unité Mixte Internationale TransVIHMI (UMI 233 IRD – U1175 INSERM - Université de Montpellier) IRD (Institut de recherche pour le développement)	Mounerou SALOU et al
EPI_ISL_1321604	Institut National d’Hygiène (INH)	Unité Mixte Internationale TransVIHMI (UMI 233 IRD – U1175 INSERM - Université de Montpellier)IRD (Institut de recherche pour le développement)	Mounerou SALOU et al
EPI_ISL_1404615	Institut National d'Hygiène	Unité Mixte Internationale TransVIHMI (UMI 233 IRD – U1175 INSERM - Université de Montpellier) IRD (Institut de recherche pour le développement)	Mounerou SALOU et al

EPI_ISL_1406144	Institut National d’Hygiène (INH)	Unité Mixte Internationale TransVIHMI (UMI 233 IRD – U1175 INSERM - Université de Montpellier)IRD (Institut de recherche pour le développement)	Mounerou SALOU et al
EPI_ISL_1293351	Institut National d’Hygiène (INH)	Unité Mixte Internationale TransVIHMI (UMI 233 IRD – U1175 INSERM - Université de Montpellier)IRD (Institut de recherche pour le développement)	Mounerou SALOU et al
EPI_ISL_1587449	Laboratoire Biolim/FSS/UL	Unité Mixte Internationale TransVIHMI (UMI 233 IRD – U1175 INSERM - Université de Montpellier) IRD (Institut de recherche pour le développement)	Mounerou SALOU et al
EPI_ISL_1654210	Laboratoire Biolim/FSS/UL	Unité Mixte Internationale TransVIHMI (UMI 233 IRD – U1175 INSERM - Université de Montpellier) IRD (Institut de recherche pour le développement)	Mounerou SALOU et al
EPI_ISL_1575297	Laboratoire Biolim/FSS/UL	Unité Mixte Internationale TransVIHMI (UMI 233 IRD – U1175 INSERM - Université de Montpellier) IRD (Institut de recherche pour le développement)	Mounerou SALOU et al
EPI_ISL_1575310	Laboratoire Biolim/FSS/UL	Unité Mixte Internationale TransVIHMI (UMI 233 IRD – U1175 INSERM - Université de Montpellier) IRD (Institut de recherche pour le développement)	Mounerou SALOU et al
EPI_ISL_1575436	Laboratoire Biolim/FSS/UL	Unité Mixte Internationale TransVIHMI (UMI 233 IRD – U1175 INSERM - Université de Montpellier) IRD (Institut de recherche pour le développement)	Mounerou SALOU et al
EPI_ISL_1575441	Laboratoire Biolim/FSS/UL	Unité Mixte Internationale TransVIHMI (UMI 233 IRD – U1175 INSERM - Université de Montpellier) IRD (Institut de recherche pour le développement)	Mounerou SALOU et al
EPI_ISL_1508956	Laboratoire Biolim/FSS/UL	Unité Mixte Internationale TransVIHMI (UMI 233 IRD – U1175 INSERM - Université de Montpellier) IRD (Institut de recherche pour le développement)	Mounerou SALOU et al
EPI_ISL_1500948	Institut National d’Hygiène (INH)	Unité Mixte Internationale TransVIHMI (UMI 233 IRD – U1175 INSERM - Université de Montpellier)IRD (Institut de recherche pour le développement)	Mounerou SALOU et al
EPI_ISL_1508849	Laboratoire Biolim/FSS/UL	Unité Mixte Internationale TransVIHMI (UMI 233 IRD – U1175 INSERM - Université de Montpellier) IRD (Institut de recherche pour le développement)	Mounerou SALOU et al
EPI_ISL_1508890	Laboratoire Biolim/FSS/UL	Unité Mixte Internationale TransVIHMI (UMI 233 IRD – U1175 INSERM - Université de Montpellier) IRD (Institut de recherche pour le développement)	Mounerou SALOU et al
EPI_ISL_1585941	Laboratoire Biolim/FSS/UL	Unité Mixte Internationale TransVIHMI (UMI 233 IRD – U1175 INSERM - Université de Montpellier) IRD (Institut de recherche pour le développement)	Mounerou SALOU et al
EPI_ISL_1508894	Laboratoire Biolim/FSS/UL	Unité Mixte Internationale TransVIHMI (UMI 233 IRD – U1175 INSERM - Université de Montpellier) IRD (Institut de recherche pour le développement)	Mounerou SALOU et al
EPI_ISL_1508916	Laboratoire Biolim/FSS/UL	Unité Mixte Internationale TransVIHMI (UMI 233 IRD – U1175 INSERM - Université de Montpellier) IRD (Institut de recherche pour le développement)	Mounerou SALOU et al
EPI_ISL_1508945	Laboratoire Biolim/FSS/UL	Unité Mixte Internationale TransVIHMI (UMI 233 IRD – U1175 INSERM - Université de Montpellier) IRD (Institut de recherche pour le développement)	Mounerou SALOU et al

EPI_ISL_1508959	Laboratoire Biolim/FSS/UL	Unité Mixte Internationale TransVIHMI (UMI 233 IRD – U1175 INSERM - Université de Montpellier) IRD (Institut de recherche pour le développement)	Mounerou SALOU et al
EPI_ISL_1509102	Laboratoire Biolim/FSS/UL	Unité Mixte Internationale TransVIHMI (UMI 233 IRD – U1175 INSERM - Université de Montpellier) IRD (Institut de recherche pour le développement)	Mounerou SALOU et al
EPI_ISL_1524717	Laboratoire Biolim/FSS/UL	Unité Mixte Internationale TransVIHMI (UMI 233 IRD – U1175 INSERM - Université de Montpellier) IRD (Institut de recherche pour le développement)	Mounerou SALOU et al
EPI_ISL_1508949	Laboratoire Biolim/FSS/UL	Unité Mixte Internationale TransVIHMI (UMI 233 IRD – U1175 INSERM - Université de Montpellier) IRD (Institut de recherche pour le développement)	Mounerou SALOU et al
EPI_ISL_1508953	Institut National d'hygiène	Unité Mixte Internationale TransVIHMI (UMI 233 IRD – U1175 INSERM - Université de Montpellier) IRD (Institut de recherche pour le développement)	Mounerou SALOU et al
EPI_ISL_1508958	Institut National d'hygiène	Unité Mixte Internationale TransVIHMI (UMI 233 IRD – U1175 INSERM - Université de Montpellier) IRD (Institut de recherche pour le développement)	Mounerou SALOU et al
EPI_ISL_1622433	RSJ Dr. Soeharto Heerdjan	Eijkman Institute for Molecular Biology, Ministry of Research and Technology/National Agency for Research and Innovation	Muhammad Rezki Rasyak et al
EPI_ISL_678211	Pathogen Genomics Lab King Abdullah University of Science and Technology (KAUST)	Pathogen Genomics Lab King Abdullah University of Science and Technology(KAUST)	Muhammad Shuaib et al
EPI_ISL_678238	Pathogen Genomics Lab King Abdullah University of Science and Technology (KAUST)	Pathogen Genomics Lab King Abdullah University of Science and Technology(KAUST)	Muhammad Shuaib et al
EPI_ISL_495423	Kafkas University, Faculty of Medicine, Department of Medical Microbiology	Kafkas University, Faculty of Medicine, Department of Medical Microbiology	Murat Karamese et al
EPI_ISL_1080932	National Health Laboratory Services	National Health Laboratory Services	Mushal Ali et al
EPI_ISL_1407113	National HIV Reference Laboratory, Ministry of Health, Public Health Institute of Malawi	KRISP, KZN Research Innovation and Sequencing Platform	Mvula B et al
EPI_ISL_1407115	National HIV Reference Laboratory, Ministry of Health, Public Health Institute of Malawi	KRISP, KZN Research Innovation and Sequencing Platform	Mvula B et al
EPI_ISL_1407116	National HIV Reference Laboratory, Ministry of Health, Public Health Institute of Malawi	KRISP, KZN Research Innovation and Sequencing Platform	Mvula B et al
EPI_ISL_1407131	National HIV Reference Laboratory, Ministry of Health, Public Health Institute of Malawi	KRISP, KZN Research Innovation and Sequencing Platform	Mvula B et al
EPI_ISL_1407148	National HIV Reference Laboratory, Ministry of Health, Public Health Institute of Malawi	KRISP, KZN Research Innovation and Sequencing Platform	Mvula B et al
EPI_ISL_1407151	National HIV Reference Laboratory, Ministry of Health, Public Health Institute of Malawi	KRISP, KZN Research Innovation and Sequencing Platform	Mvula B et al
EPI_ISL_1407158	National HIV Reference Laboratory, Ministry of Health, Public Health Institute of Malawi	KRISP, KZN Research Innovation and Sequencing Platform	Mvula B et al
EPI_ISL_1407201	National HIV Reference Laboratory, Ministry of Health, Public Health Institute of Malawi	KRISP, KZN Research Innovation and Sequencing Platform	Mvula B et al
EPI_ISL_1407212	National HIV Reference Laboratory, Ministry of Health, Public Health Institute of Malawi	KRISP, KZN Research Innovation and Sequencing Platform	Mvula B et al

EPI_ISL_1407253	National HIV Reference Laboratory, Ministry of Health, Public Health Institute of Malawi	KRISP, KZN Research Innovation and Sequencing Platform	Mvula B et al
EPI_ISL_512844	Department of Medical Research	DMR_Myanmar	Myat Htut Nyunt et al
EPI_ISL_1103569	Laboratoire central de Virologie, Centre hospitalo-universitaire Ibn Sina, Hopital des spécialités, Rabat	Laboratoire de Biotechnologie	Myriam Seffar et al
EPI_ISL_1103576	Laboratoire central de Virologie	Laboratoire de Biotechnologie	Myriam Seffar et al
EPI_ISL_1103579	Laboratoire central de Virologie, Centre hospitalo-universitaire Ibn Sina, Hôpital des spécialités	Laboratoire de Biotechnologie	Myriam Seffar et al
EPI_ISL_793013	Laboratorio de Virología del Hospital de Niños Dr. Ricardo Gutierrez	Área de Secuenciación del Laboratorio de Virología del Hospital de Niños Dr. Ricardo Gutierrez on behalf of 'Proyecto Argentino Interinstitucional de genomica de SARS-CoV-2' (PAIS Consortium)	Nabaes Jodar et al
EPI_ISL_430815	Laboratorio de Virología del Hospital de Niños Dr. Ricardo Gutierrez	Área de Secuenciación del Laboratorio de Virología del Hospital de Niños Dr. Ricardo Gutierrez on behalf of 'Proyecto Argentino Interinstitucional de genomica de SARS-CoV-2' (PAIS Consortium)	Nabaes Jodar et al
EPI_ISL_430817	Laboratorio de Virología del Hospital de Niños Dr. Ricardo Gutierrez	Área de Secuenciación del Laboratorio de Virología del Hospital de Niños Dr. Ricardo Gutierrez on behalf of 'Proyecto Argentino Interinstitucional de genomica de SARS-CoV-2' (PAIS Consortium)	Nabaes Jodar et al
EPI_ISL_792223	Laboratorio de Virología del Hospital de Niños Dr. Ricardo Gutierrez	Área de Secuenciación del Laboratorio de Virología del Hospital de Niños Dr. Ricardo Gutierrez on behalf of 'Proyecto Argentino Interinstitucional de genomica de SARS-CoV-2' (PAIS Consortium)	Nabaes Jodar et al
EPI_ISL_792235	Laboratorio de Virología del Hospital de Niños Dr. Ricardo Gutierrez	Área de Secuenciación del Laboratorio de Virología del Hospital de Niños Dr. Ricardo Gutierrez on behalf of 'Proyecto Argentino Interinstitucional de genomica de SARS-CoV-2' (PAIS Consortium)	Nabaes Jodar et al
EPI_ISL_792302	Unidad de Virología, Centro de Educación Médica en Investigaciones Clínicas CEMIC	Área de Secuenciación del Laboratorio de Virología del Hospital de Niños Dr. Ricardo Gutierrez on behalf of 'Proyecto Argentino Interinstitucional de genomica de SARS-CoV-2' (PAIS Consortium)	Nabaes Jodar et al
EPI_ISL_792303	Unidad de Virología, Centro de Educación Médica en Investigaciones Clínicas CEMIC	Área de Secuenciación del Laboratorio de Virología del Hospital de Niños Dr. Ricardo Gutierrez on behalf of 'Proyecto Argentino Interinstitucional de genomica de SARS-CoV-2' (PAIS Consortium)	Nabaes Jodar et al
EPI_ISL_792317	Laboratorio del Hospital El Cruce Dr. Néstor C. Kirchner	Área de Secuenciación del Laboratorio de Virología del Hospital de Niños Dr. Ricardo Gutierrez on behalf of 'Proyecto Argentino Interinstitucional de genomica de SARS-CoV-2' (PAIS Consortium)	Nabaes Jodar et al
EPI_ISL_792335	Laboratorio del Hospital Interzonal General de Agudos Evita	Área de Secuenciación del Laboratorio de Virología del Hospital de Niños Dr. Ricardo Gutierrez on behalf of 'Proyecto Argentino Interinstitucional de genomica de SARS-CoV-2' (PAIS Consortium)	Nabaes Jodar et al
EPI_ISL_792336	Laboratorio del Hospital Interzonal General de Agudos Evita	Área de Secuenciación del Laboratorio de Virología del Hospital de Niños Dr. Ricardo Gutierrez on behalf of 'Proyecto Argentino Interinstitucional de genomica de SARS-CoV-2' (PAIS Consortium)	Nabaes Jodar et al
EPI_ISL_792346	Laboratorio del Hospital Interzonal General de Agudos Evita	Área de Secuenciación del Laboratorio de Virología del Hospital de Niños Dr. Ricardo Gutierrez on behalf of 'Proyecto Argentino Interinstitucional de genomica de SARS-CoV-2' (PAIS Consortium)	Nabaes Jodar et al
EPI_ISL_792376	Laboratorio de salud pública, Facultad de Ciencias Exactas, Universidad Nacional de La Plata	Área de Secuenciación del Laboratorio de Virología del Hospital de Niños Dr. Ricardo Gutierrez on behalf of 'Proyecto Argentino Interinstitucional de genomica de SARS-CoV-2' (PAIS Consortium)	Nabaes Jodar et al

EPI_ISL_792377	Laboratorio de salud pública, Facultad de Ciencias Exactas, Universidad Nacional de La Plata	Área de Secuenciación del Laboratorio de Virología del Hospital de Niños Dr. Ricardo Gutierrez on behalf of 'Proyecto Argentino Interinstitucional de genomica de SARS-CoV-2' (PAIS Consortium)	Nabaes Jodar et al
EPI_ISL_792380	Laboratorio de salud pública, Facultad de Ciencias Exactas, Universidad Nacional de La Plata	Área de Secuenciación del Laboratorio de Virología del Hospital de Niños Dr. Ricardo Gutierrez on behalf of 'Proyecto Argentino Interinstitucional de genomica de SARS-CoV-2' (PAIS Consortium)	Nabaes Jodar et al
EPI_ISL_792383	Laboratorio de salud pública, Facultad de Ciencias Exactas, Universidad Nacional de La Plata	Área de Secuenciación del Laboratorio de Virología del Hospital de Niños Dr. Ricardo Gutierrez on behalf of 'Proyecto Argentino Interinstitucional de genomica de SARS-CoV-2' (PAIS Consortium)	Nabaes Jodar et al
EPI_ISL_792385	Laboratorio de salud pública, Facultad de Ciencias Exactas, Universidad Nacional de La Plata	Área de Secuenciación del Laboratorio de Virología del Hospital de Niños Dr. Ricardo Gutierrez on behalf of 'Proyecto Argentino Interinstitucional de genomica de SARS-CoV-2' (PAIS Consortium)	Nabaes Jodar et al
EPI_ISL_792395	Plataforma de Servicios Biotecnólogicos: UTTIPP/PSB , Universidad Nacional de Quilmes.	Área de Secuenciación del Laboratorio de Virología del Hospital de Niños Dr. Ricardo Gutierrez on behalf of 'Proyecto Argentino Interinstitucional de genomica de SARS-CoV-2' (PAIS Consortium)	Nabaes Jodar et al
EPI_ISL_971451	Cell culture Unit at CV-MIT belonging to HIMMV	Functional Genomic Platform UATRS-biology, CNRST	Nadia Touil et al
EPI_ISL_851051	Gandhi Medical College and Hospital	CSIR-Centre for Cellular and Molecular Biology	Nagamani Kammili et al
EPI_ISL_850514	Gandhi Medical College and Hospital	MDRU-DHR,Gandhi Medical College	Nagamani Kammili et al
EPI_ISL_660557	The National Institute of Public Health	State Veterinary Institute Prague	Nagy et al
EPI_ISL_693664	The National Institute of Public Health	State Veterinary Institute Prague	Nagy et al
EPI_ISL_693672	The National Institute of Public Health	State Veterinary Institute Prague	Nagy et al
EPI_ISL_693663	The National Institute of Public Health	State Veterinary Institute Prague	Nagy et al
EPI_ISL_1510357	National Institute of Public Health	State Veterinary Institute Prague	Nagy et al
EPI_ISL_584080	The National Institute of Public Health	State Veterinary Institute Prague	Nagy et al
EPI_ISL_584078	The National Institute of Public Health	State Veterinary Institute Prague	Nagy et al
EPI_ISL_737014	The National Institute of Public Health	State Veterinary Institute Prague	Nagy et al
EPI_ISL_737022	The National Institute of Public Health	State Veterinary Institute Prague	Nagy et al
EPI_ISL_792690	The National Institute of Public Health	State Veterinary Institute Prague	Nagy et al
EPI_ISL_792707	The National Institute of Public Health	State Veterinary Institute Prague	Nagy et al
EPI_ISL_792697	The National Institute of Public Health	State Veterinary Institute Prague	Nagy et al
EPI_ISL_960414	The National Institute of Public Health	State Veterinary Institute Prague	Nagy et al
EPI_ISL_960423	The National Institute of Public Health	State Veterinary Institute Prague	Nagy et al
EPI_ISL_960438	The National Institute of Public Health	State Veterinary Institute Prague	Nagy et al
EPI_ISL_960424	The National Institute of Public Health	State Veterinary Institute Prague	Nagy et al
EPI_ISL_882953	The National Institute of Public Health	State Veterinary Institute Prague	Nagy et al
EPI_ISL_577626	The National Institute of Public Health	State Veterinary Institute Prague	Nagy et al

EPI_ISL_577638	The National Institute of Public Health	State Veterinary Institute Prague	Nagy et al
EPI_ISL_887455	Instituto Nacional de Saude (INS), Mozambique	KRISP, KZN Research Innovation and Sequencing Platform	Nalia Ismael et al
EPI_ISL_887456	Instituto Nacional de Saude (INS), Mozambique	KRISP, KZN Research Innovation and Sequencing Platform	Nalia Ismael et al
EPI_ISL_887458	Instituto Nacional de Saude (INS), Mozambique	KRISP, KZN Research Innovation and Sequencing Platform	Nalia Ismael et al
EPI_ISL_887459	Instituto Nacional de Saude (INS), Mozambique	KRISP, KZN Research Innovation and Sequencing Platform	Nalia Ismael et al
EPI_ISL_887460	Instituto Nacional de Saude (INS), Mozambique	KRISP, KZN Research Innovation and Sequencing Platform	Nalia Ismael et al
EPI_ISL_887461	Instituto Nacional de Saude (INS), Mozambique	KRISP, KZN Research Innovation and Sequencing Platform	Nalia Ismael et al
EPI_ISL_887464	Instituto Nacional de Saude (INS), Mozambique	KRISP, KZN Research Innovation and Sequencing Platform	Nalia Ismael et al
EPI_ISL_887474	Instituto Nacional de Saude (INS), Mozambique	KRISP, KZN Research Innovation and Sequencing Platform	Nalia Ismael et al
EPI_ISL_887428	Instituto Nacional de Saude (INS), Mozambique	KRISP, KZN Research Innovation and Sequencing Platform	Nalia Ismael et al
EPI_ISL_887444	Instituto Nacional de Saude (INS), Mozambique	KRISP, KZN Research Innovation and Sequencing Platform	Nalia Ismael et al
EPI_ISL_887445	Instituto Nacional de Saude (INS), Mozambique	KRISP, KZN Research Innovation and Sequencing Platform	Nalia Ismael et al
EPI_ISL_887447	Instituto Nacional de Saude (INS), Mozambique	KRISP, KZN Research Innovation and Sequencing Platform	Nalia Ismael et al
EPI_ISL_887420	Instituto Nacional de Saude (INS), Mozambique	KRISP, KZN Research Innovation and Sequencing Platform	Nalia Ismael et al
EPI_ISL_887500	Instituto Nacional de Saude (INS), Mozambique	KRISP, KZN Research Innovation and Sequencing Platform	Nalia Ismael et al
EPI_ISL_887501	Instituto Nacional de Saude (INS), Mozambique	KRISP, KZN Research Innovation and Sequencing Platform	Nalia Ismael et al
EPI_ISL_887421	Instituto Nacional de Saude (INS), Mozambique	KRISP, KZN Research Innovation and Sequencing Platform	Nalia Ismael et al
EPI_ISL_887434	Instituto Nacional de Saude (INS), Mozambique	KRISP, KZN Research Innovation and Sequencing Platform	Nalia Ismael et al
EPI_ISL_887431	Instituto Nacional de Saude (INS), Mozambique	KRISP, KZN Research Innovation and Sequencing Platform	Nalia Ismael et al
EPI_ISL_887426	Instituto Nacional de Saude (INS), Mozambique	KRISP, KZN Research Innovation and Sequencing Platform	Nalia Ismael et al
EPI_ISL_887502	Instituto Nacional de Saude (INS), Mozambique	KRISP, KZN Research Innovation and Sequencing Platform	Nalia Ismael et al
EPI_ISL_887503	Instituto Nacional de Saude (INS), Mozambique	KRISP, KZN Research Innovation and Sequencing Platform	Nalia Ismael et al
EPI_ISL_887429	Instituto Nacional de Saude (INS), Mozambique	KRISP, KZN Research Innovation and Sequencing Platform	Nalia Ismael et al
EPI_ISL_887433	Instituto Nacional de Saude (INS), Mozambique	KRISP, KZN Research Innovation and Sequencing Platform	Nalia Ismael et al
EPI_ISL_887430	Instituto Nacional de Saude (INS), Mozambique	KRISP, KZN Research Innovation and Sequencing Platform	Nalia Ismael et al
EPI_ISL_964923	Instituto Nacional de Saude (INS), Mozambique	KRISP, KZN Research Innovation and Sequencing Platform	Nalia Ismael et al
EPI_ISL_1132824	Instituto Nacional de Saude (INS), Mozambique	KRISP, KZN Research Innovation and Sequencing Platform	Nalia Ismael et al
EPI_ISL_1132836	Instituto Nacional de Saude (INS), Mozambique	KRISP, KZN Research Innovation and Sequencing Platform	Nalia Ismael et al
EPI_ISL_1132843	Instituto Nacional de Saude (INS), Mozambique	KRISP, KZN Research Innovation and Sequencing Platform	Nalia Ismael et al
EPI_ISL_626569	National Centre for Communicable Disease (NCCD) National Influenza Center	National Centre for Communicable Disease (NCCD) National Influenza Center	Naranzul Ts et al

EPI_ISL_626566	National Centre for Communication Disease (NCCD) National Influenza Center	National Centre for Communication Disease (NCCD) National Influenza Center	Naranzul Ts et al
EPI_ISL_626626	National Centre for Communicable Disease (NCCD) National Influenza Center	National Centre for Communicable Disease (NCCD) National Influenza Center	Naranzul Ts et al
EPI_ISL_626627	National Centre for Communicable Disease (NCCD) National Influenza Center	National Centre for Communicable Disease (NCCD) National Influenza Center	Naranzul Ts et al
EPI_ISL_467666	Virology lab, NIC, NCCD, Ulaanbaatar, Mongolia	National Centre for Communicable Deseases (NCCD)	Naranzul Ts et al
EPI_ISL_1502568	Biochemistry and Molecular Biology Department-Faculty of Medicine, Al-Quds University	Biochemistry and Molecular Biology Department-Faculty of Medicine, Al-Quds University	Nasereddin et al
EPI_ISL_1502570	Biochemistry and Molecular Biology Department-Faculty of Medicine, Al-Quds University	Biochemistry and Molecular Biology Department-Faculty of Medicine, Al-Quds University	Nasereddin et al
EPI_ISL_1502574	Biochemistry and Molecular Biology Department-Faculty of Medicine, Al-Quds University	Biochemistry and Molecular Biology Department-Faculty of Medicine, Al-Quds University	Nasereddin et al
EPI_ISL_1502578	Biochemistry and Molecular Biology Department-Faculty of Medicine, Al-Quds University	Biochemistry and Molecular Biology Department-Faculty of Medicine, Al-Quds University	Nasereddin et al
EPI_ISL_1395939	Laboratorio Central de Salud Pública	Grupo de Genómica y Bioinformática del Instituto de Investigación de la Cadena Láctea CONICET-INTA on behalf of 'Proyecto Argentino Interinstitucional de genomica de SARS-CoV-2' (PAIS Consortium)	Natalia Andrea Ayala y María Verónica Góm
EPI_ISL_941111	Centro de Investigaciones en Microbiología y Biotecnología-UR (CIMBIUR), Facultad de Ciencias Naturales, Universidad del Rosario, Bogotá, Colombia	Centro de Investigaciones en Microbiología y Biotecnología-UR (CIMBIUR), Facultad de Ciencias Naturales, Universidad del Rosario, Bogotá, Colombia Icahn School of Medicine at Mount Sinai, New York, USA	Nathalia Ballesteros et al
EPI_ISL_941114	Centro de Investigaciones en Microbiología y Biotecnología-UR (CIMBIUR), Facultad de Ciencias Naturales, Universidad del Rosario, Bogotá, Colombia	Centro de Investigaciones en Microbiología y Biotecnología-UR (CIMBIUR), Facultad de Ciencias Naturales, Universidad del Rosario, Bogotá, Colombia Icahn School of Medicine at Mount Sinai, New York, USA	Nathalia Ballesteros et al
EPI_ISL_941123	Centro de Investigaciones en Microbiología y Biotecnología-UR (CIMBIUR), Facultad de Ciencias Naturales, Universidad del Rosario, Bogotá, Colombia	Centro de Investigaciones en Microbiología y Biotecnología-UR (CIMBIUR), Facultad de Ciencias Naturales, Universidad del Rosario, Bogotá, Colombia Icahn School of Medicine at Mount Sinai, New York, USA	Nathalia Ballesteros et al
EPI_ISL_941139	Centro de Investigaciones en Microbiología y Biotecnología-UR (CIMBIUR), Facultad de Ciencias Naturales, Universidad del Rosario, Bogotá, Colombia	Centro de Investigaciones en Microbiología y Biotecnología-UR (CIMBIUR), Facultad de Ciencias Naturales, Universidad del Rosario, Bogotá, Colombia Icahn School of Medicine at Mount Sinai, New York, USA	Nathalia Ballesteros et al
EPI_ISL_941140	Centro de Investigaciones en Microbiología y Biotecnología-UR (CIMBIUR), Facultad de Ciencias Naturales, Universidad del Rosario, Bogotá, Colombia	Centro de Investigaciones en Microbiología y Biotecnología-UR (CIMBIUR), Facultad de Ciencias Naturales, Universidad del Rosario, Bogotá, Colombia Icahn School of Medicine at Mount Sinai, New York, USA	Nathalia Ballesteros et al
EPI_ISL_941149	Centro de Investigaciones en Microbiología y Biotecnología-UR (CIMBIUR), Facultad de Ciencias Naturales, Universidad del Rosario, Bogotá, Colombia	Centro de Investigaciones en Microbiología y Biotecnología-UR (CIMBIUR), Facultad de Ciencias Naturales, Universidad del Rosario, Bogotá, Colombia Icahn School of Medicine at Mount Sinai, New York, USA	Nathalia Ballesteros et al
EPI_ISL_941154	Centro de Investigaciones en Microbiología y Biotecnología-UR (CIMBIUR), Facultad de Ciencias Naturales, Universidad del Rosario, Bogotá, Colombia	Centro de Investigaciones en Microbiología y Biotecnología-UR (CIMBIUR), Facultad de Ciencias Naturales, Universidad del Rosario, Bogotá, Colombia Icahn School of Medicine at Mount Sinai, New York, USA	Nathalia Ballesteros et al
EPI_ISL_415643	R. G. Lugar Center for Public Health Research, National Center for Disease Control and Public Health (NCDC) of Georgia.	R. G. Lugar Center for Public Health Research, National Center for Disease Control and Public Health (NCDC) of Georgia.	Nato Kotaria et al
EPI_ISL_415641	R. G. Lugar Center for Public Health Research, National Center for Disease Control and Public Health (NCDC) of Georgia.	R. G. Lugar Center for Public Health Research, National Center for Disease Control and Public Health (NCDC) of Georgia.	Nato Kotaria et al
EPI_ISL_415644	R. G. Lugar Center for Public Health Research, National Center for Disease Control and Public Health (NCDC) of Georgia.	R. G. Lugar Center for Public Health Research, National Center for Disease Control and Public Health (NCDC) of Georgia.	Nato Kotaria et al

ıez et al

EPI_ISL_418206	Institut Pasteur Dakar	Institut Pasteur de Dakar	Ndongo Dia et al
EPI_ISL_418216	Institut Pasteur Dakar	Institut Pasteur de Dakar	Ndongo Dia et al
EPI_ISL_480789	Institut Pasteur Dakar	Institut Pasteur de Dakar	Ndongo Dia et al
EPI_ISL_481220	Institut Pasteur Dakar	Institut Pasteur de Dakar	Ndongo Dia et al
EPI_ISL_481234	Institut Pasteur Dakar	Institut Pasteur de Dakar	Ndongo Dia et al
EPI_ISL_498238	Institut Pasteur de Dakar	Institut Pasteur de Dakar	Ndongo Dia et al
EPI_ISL_498239	Institut Pasteur de Dakar	Institut Pasteur de Dakar	Ndongo Dia et al
EPI_ISL_498241	Institut Pasteur de Dakar	Institut Pasteur de Dakar	Ndongo Dia et al
EPI_ISL_420078	Institut Pasteur Dakar	Institut Pasteur de Dakar	Ndongo Dia et al
EPI_ISL_498246	Institut Pasteur de Dakar	Institut Pasteur de Dakar	Ndongo Dia et al
EPI_ISL_476560	Institut Pasteur Dakar	Institut Pasteur de Dakar	Ndongo Dia et al
EPI_ISL_476570	Institut Pasteur Dakar	Institut Pasteur de Dakar	Ndongo Dia et al
EPI_ISL_486869	Institut Pasteur Dakar	Institut Pasteur de Dakar	Ndongo Dia et al
EPI_ISL_498235	Institut Pasteur de Dakar	Institut Pasteur de Dakar	Ndongo Dia et al
EPI_ISL_498251	Institut Pasteur de Dakar	Institut Pasteur de Dakar	Ndongo Dia et al
EPI_ISL_1209667	Israel Central Virology laboratory	Israel National Consortium for SARS-CoV-2 sequencing	Neta Zuckerman et al
EPI_ISL_889051	Israel Central Virology laboratory	Israel National Consortium for SARS-CoV-2 sequencing	Neta Zuckerman et al
EPI_ISL_1209489	Israel Central Virology laboratory	Israel National Consortium for SARS-CoV-2 sequencing	Neta Zuckerman et al
EPI_ISL_1210310	Israel Central Virology laboratory	Israel National Consortium for SARS-CoV-2 sequencing	Neta Zuckerman et al
EPI_ISL_1358495	Israel Central Virology laboratory	Israel National Consortium for SARS-CoV-2 sequencing	Neta Zuckerman et al
EPI_ISL_1209816	Israel Central Virology laboratory	Israel National Consortium for SARS-CoV-2 sequencing	Neta Zuckerman et al
EPI_ISL_804112	Israel Central Virology laboratory	Israel National Consortium for SARS-CoV-2 sequencing	Neta Zuckerman et al
EPI_ISL_1240654	Israel Central Virology laboratory	Israel National Consortium for SARS-CoV-2 sequencing	Neta Zuckerman et al
EPI_ISL_889025	Israel Central Virology laboratory	Israel National Consortium for SARS-CoV-2 sequencing	Neta Zuckerman et al
EPI_ISL_889141	Israel Central Virology laboratory	Israel National Consortium for SARS-CoV-2 sequencing	Neta Zuckerman et al
EPI_ISL_889144	Israel Central Virology laboratory	Israel National Consortium for SARS-CoV-2 sequencing	Neta Zuckerman et al
EPI_ISL_1073156	Israel Central Virology laboratory	Israel National Consortium for SARS-CoV-2 sequencing	Neta Zuckerman et al
EPI_ISL_889270	Israel Central Virology laboratory	Israel National Consortium for SARS-CoV-2 sequencing	Neta Zuckerman et al
EPI_ISL_514308	Israel Central Virology laboratory	Israel Central Virology laboratory	Neta Zuckerman et al
EPI_ISL_1240645	Israel Central Virology Laboratory	Israel National Consortium for SARS-CoV-2 sequencing	Neta Zuckerman et al

EPI_ISL_1240647	Israel Central Virology Laboratory	Israel National Consortium for SARS-CoV-2 sequencing	Neta Zuckerman et al
EPI_ISL_1240648	Israel Central Virology Laboratory	Israel National Consortium for SARS-CoV-2 sequencing	Neta Zuckerman et al
EPI_ISL_1240649	Israel Central Virology Laboratory	Israel National Consortium for SARS-CoV-2 sequencing	Neta Zuckerman et al
EPI_ISL_1240650	Israel Central Virology Laboratory	Israel National Consortium for SARS-CoV-2 sequencing	Neta Zuckerman et al
EPI_ISL_1240651	Israel Central Virology laboratory	Israel National Consortium for SARS-CoV-2 sequencing	Neta Zuckerman et al
EPI_ISL_516900	Israel Central Virology laboratory	Israel Central Virology laboratory	Neta Zuckerman et al
EPI_ISL_514278	Israel Central Virology laboratory	Israel Central Virology laboratory	Neta Zuckerman et al
EPI_ISL_514282	Israel Central Virology laboratory	Israel Central Virology laboratory	Neta Zuckerman et al
EPI_ISL_514298	Israel Central Virology laboratory	Israel Central Virology laboratory	Neta Zuckerman et al
EPI_ISL_649094	Israel Central Virology laboratory	Israel Central Virology laboratory	Neta Zuckerman et al
EPI_ISL_575332	Israel Central Virology laboratory	Israel Central Virology laboratory	Neta Zuckerman et al
EPI_ISL_575334	Israel Central Virology laboratory	Israel Central Virology laboratory	Neta Zuckerman et al
EPI_ISL_516904	Israel Central Virology laboratory	Israel Central Virology laboratory	Neta Zuckerman et al
EPI_ISL_516915	Israel Central Virology laboratory	Israel Central Virology laboratory	Neta Zuckerman et al
EPI_ISL_483708	Israel Central Virology laboratory	Israel Central Virology laboratory	Neta Zuckerman et al
EPI_ISL_483721	Israel Central Virology laboratory	Israel Central Virology laboratory	Neta Zuckerman et al
EPI_ISL_515082	Department of Biochemistry, Cell and Molecular Biology	WACCBIP, University of Ghana	Ngoi et al
EPI_ISL_515085	Department of Biochemistry, Cell and Molecular Biology	WACCBIP, University of Ghana	Ngoi et al
EPI_ISL_515087	Department of Biochemistry, Cell and Molecular Biology	WACCBIP, University of Ghana	Ngoi et al
EPI_ISL_515096	Department of Biochemistry, Cell and Molecular Biology	WACCBIP, University of Ghana	Ngoi et al
EPI_ISL_515098	Department of Biochemistry, Cell and Molecular Biology	WACCBIP, University of Ghana	Ngoi et al
EPI_ISL_515103	Department of Biochemistry, Cell and Molecular Biology	WACCBIP, University of Ghana	Ngoi et al
EPI_ISL_515106	Department of Biochemistry, Cell and Molecular Biology	WACCBIP, University of Ghana	Ngoi et al
EPI_ISL_884843	Department of Biochemistry, Cell and Molecular Biology, West African Centre for Cell Biology of Infectious Pathogens (WACCBIP), University of Ghana	Department of Biochemistry, Cell and Molecular Biology, West African Centre for Cell Biology of Infectious Pathogens (WACCBIP), University of Ghana	Ngoi et al
EPI_ISL_884849	Department of Biochemistry, Cell and Molecular Biology, West African Centre for Cell Biology of Infectious Pathogens (WACCBIP), University of Ghana	Department of Biochemistry, Cell and Molecular Biology, West African Centre for Cell Biology of Infectious Pathogens (WACCBIP), University of Ghana	Ngoi et al
EPI_ISL_884851	Department of Biochemistry, Cell and Molecular Biology, West African Centre for Cell Biology of Infectious Pathogens (WACCBIP), University of Ghana	Department of Biochemistry, Cell and Molecular Biology, West African Centre for Cell Biology of Infectious Pathogens (WACCBIP), University of Ghana	Ngoi et al
EPI_ISL_481230	Lab voor klinische biologie	Onderzoeksgroep Virologie	Nick Vereecke et al
EPI_ISL_717588	Lab voor klinische biologie	Onderzoeksgroep Virologie	Nick Vereecke et al

EPI_ISL_1524330	Biology Department, College of Science, Al Muthanna University and Public Health Laboratory, Al-Muthanna Health Directorate	Department of Virology, Faculty of Medicine, University of Helsinki, Helsinki, Finland generated and submitted to GISAID	Nihad Al-Rashedi et al
EPI_ISL_1524344	Biology Department, College of Science, Al Muthanna University and Public Health Laboratory, Al-Muthanna Health Directorate	Department of Virology, Faculty of Medicine, University of Helsinki, Helsinki, Finland generated and submitted to GISAID	Nihad Al-Rashedi et al
EPI_ISL_1524347	Biology Department, College of Science, Al Muthanna University and Public Health Laboratory, Al-Muthanna Health Directorate	Department of Virology, Faculty of Medicine, University of Helsinki, Helsinki, Finland generated and submitted to GISAID	Nihad Al-Rashedi et al
EPI_ISL_1524352	Biology Department, College of Science, Al Muthanna University and Public Health Laboratory, Al-Muthanna Health Directorate	Department of Virology, Faculty of Medicine, University of Helsinki, Helsinki, Finland generated and submitted to GISAID	Nihad Al-Rashedi et al
EPI_ISL_1524356	Biology Department, College of Science, Al Muthanna University and Public Health Laboratory, Al-Muthanna Health Directorate	Department of Virology, Faculty of Medicine, University of Helsinki, Helsinki, Finland generated and submitted to GISAID	Nihad Al-Rashedi et al
EPI_ISL_1524332	Biology Department, College of Science, Al Muthanna University and Public Health Laboratory, Al-Muthanna Health Directorate	Department of Virology, Faculty of Medicine, University of Helsinki, Helsinki, Finland generated and submitted to GISAID	Nihad Al-Rashedi et al
EPI_ISL_1524378	Biology Department, College of Science, Al Muthanna University and Public Health Laboratory, Al-Muthanna Health Directorate	Department of Virology, Faculty of Medicine, University of Helsinki, Helsinki, Finland generated and submitted to GISAID	Nihad Al-Rashedi et al
EPI_ISL_1524381	Biology Department, College of Science, Al Muthanna University and Public Health Laboratory, Al-Muthanna Health Directorate	Department of Virology, Faculty of Medicine, University of Helsinki, Helsinki, Finland generated and submitted to GISAID	Nihad Al-Rashedi et al
EPI_ISL_1524337	Biology Department, College of Science, Al Muthanna University and Public Health Laboratory, Al-Muthanna Health Directorate	Department of Virology, Faculty of Medicine, University of Helsinki, Helsinki, Finland generated and submitted to GISAID	Nihad Al-Rashedi et al
EPI_ISL_977655	Caribbean Public Health Agency	Carrington Lab, Department of PreClinical Sciences, Building 36, First Floor Biochemistry Unit, Faculty of Medical Sciences, The University of the West Indies	Nikita S. D. Sahadeo et al
EPI_ISL_977656	Caribbean Public Health Agency	Carrington Lab, Department of PreClinical Sciences, Building 36, First Floor Biochemistry Unit, Faculty of Medical Sciences, The University of the West Indies	Nikita S. D. Sahadeo et al
EPI_ISL_977658	Caribbean Public Health Agency	Carrington Lab, Department of PreClinical Sciences, Building 36, First Floor Biochemistry Unit, Faculty of Medical Sciences, The University of the West Indies	Nikita S. D. Sahadeo et al
EPI_ISL_977659	Caribbean Public Health Agency	Carrington Lab, Department of PreClinical Sciences, Building 36, First Floor Biochemistry Unit, Faculty of Medical Sciences, The University of the West Indies	Nikita S. D. Sahadeo et al
EPI_ISL_756367	The Caribbean Public Health Agency	Carrington Lab, Department of PreClinical Sciences, Faculty of Medical Sciences, The University of the West Indies	Nikita S. D. Sahadeo et al
EPI_ISL_872193	Caribbean Public Health Agency	Carrington Lab, Department ofBuilding 36, First Floor Biochemistry Unit, Faculty of Medical Sciences, The University of the West Indies	Nikita S. D. Sahadeo et al
EPI_ISL_872194	Caribbean Public Health Agency	Carrington Lab, Department of PreClinical Sciences, Faculty of Medical Sciences, The University of the West Indies	Nikita S. D. Sahadeo et al
EPI_ISL_872195	Caribbean Public Health Agency	Carrington Lab, Department of PreClinical Sciences, Faculty of Medical Sciences, The University of the West Indies	Nikita S. D. Sahadeo et al
EPI_ISL_977583	Caribbean Public Health Agency	Carrington Lab, Department of PreClinical Sciences	Nikita S. D. Sahadeo et al
EPI_ISL_977538	Caribbean Public Health Agency	Carrington Lab, Department of PreClinical Sciences	Nikita S. D. Sahadeo et al
EPI_ISL_1498262	The Caribbean Public Health Agency	Carrington Lab, Department of PreClinical Sciences, Faculty of Medical Sciences, The University of the West Indies	Nikita S. D. Sahadeo et al
EPI_ISL_1498379	The Caribbean Public Health Agency	Carrington Lab, Department of PreClinical Sciences, Faculty of Medical Sciences, The University of the West Indies	Nikita S. D. Sahadeo et al

EPI_ISL_1499115	Trinidad Public Health Laboratory	Carrington Lab, Department of PreClinical Sciences, Faculty of Medical Sciences, The University of the West Indies	Nikita S. D. Sahadeo et al
EPI_ISL_1490252	The Caribbean Public Health Agency	Carrington Lab, Department of PreClinical Sciences, Faculty of Medical Sciences, The University of the West Indies	Nikita S. D. Sahadeo et al
EPI_ISL_1490228	The Caribbean Public Health Agency	Carrington Lab, Department of PreClinical Sciences, Faculty of Medical Sciences, The University of the West Indies	Nikita S. D. Sahadeo et al
EPI_ISL_1490226	The Caribbean Public Health Agency	Carrington Lab, Department of PreClinical Sciences, Faculty of Medical Sciences, The University of the West Indies	Nikita S. D. Sahadeo et al
EPI_ISL_1591351	Caribbean Public Health Agency	Carrington Lab, Department of PreClinical Sciences, Faculty of Medical Sciences, The University of the West Indies	Nikita S. D. Sahadeo et al
EPI_ISL_1591250	Caribbean Public Health Agency	Carrington Lab, Department of PreClinical Sciences, Faculty of Medical Sciences, The University of the West Indies	Nikita S. D. Sahadeo et al
EPI_ISL_1591265	Caribbean Public Health Agency	Carrington Lab, Department of PreClinical Sciences, Faculty of Medical Sciences, The University of the West Indies	Nikita S. D. Sahadeo et al
EPI_ISL_1490666	The Caribbean Public Health Agency	Carrington Lab, Department of PreClinical Sciences, Faculty of Medical Sciences, The University of the West Indies	Nikita S. D. Sahadeo et al
EPI_ISL_1498095	The Caribbean Public Health Agency	Carrington Lab, Department of PreClinical Sciences, Faculty of Medical Sciences, The University of the West Indies	Nikita S. D. Sahadeo et al
EPI_ISL_1588899	Caribbean Public Health Agency	Carrington Lab, Department of PreClinical Sciences, Faculty of Medical Sciences, The University of the West Indies	Nikita S. D. Sahadeo et al
EPI_ISL_1588904	Caribbean Public Health Agency	Carrington Lab, Department of PreClinical Sciences, Faculty of Medical Sciences, The University of the West Indies	Nikita S. D. Sahadeo et al
EPI_ISL_1589003	Caribbean Public Health Agency	Carrington Lab, Department of PreClinical Sciences, Faculty of Medical Sciences, The University of the West Indies	Nikita S. D. Sahadeo et al
EPI_ISL_1589004	Caribbean Public Health Agency	Carrington Lab, Department of PreClinical Sciences, Faculty of Medical Sciences, The University of the West Indies	Nikita S. D. Sahadeo et al
EPI_ISL_1589115	Caribbean Public Health Agency	Carrington Lab, Department of PreClinical Sciences, Faculty of Medical Sciences, The University of the West Indies	Nikita S. D. Sahadeo et al
EPI_ISL_1591458	Caribbean Public Health Agency	Carrington Lab, Department of PreClinical Sciences, Faculty of Medical Sciences, The University of the West Indies	Nikita S. D. Sahadeo et al
EPI_ISL_1591459	Caribbean Public Health Agency	Carrington Lab, Department of PreClinical Sciences, Faculty of Medical Sciences, The University of the West Indies	Nikita S. D. Sahadeo et al
EPI_ISL_1591718	Caribbean Public Health Agency	Carrington Lab, Department of PreClinical Sciences, Faculty of Medical Sciences, The University of the West Indies	Nikita S. D. Sahadeo et al
EPI_ISL_1591518	Caribbean Public Health Agency	Carrington Lab, Department of PreClinical Sciences, Faculty of Medical Sciences, The University of the West Indies	Nikita S. D. Sahadeo et al
EPI_ISL_1591719	Caribbean Public Health Agency	Carrington Lab, Department of PreClinical Sciences, Faculty of Medical Sciences, The University of the West Indies	Nikita S. D. Sahadeo et al
EPI_ISL_1591720	Caribbean Public Health Laboratory	Carrington Lab, Department of PreClinical Sciences, Faculty of Medical Sciences, The University of the West Indies	Nikita S. D. Sahadeo et al
EPI_ISL_1591721	Caribbean Public Health Agency	Carrington Lab, Department of PreClinical Sciences, Faculty of Medical Sciences, The University of the West Indies	Nikita S. D. Sahadeo et al
EPI_ISL_717694	Trinidad Public Health Laboratory	Carrington Lab, Department of PreClinical Sciences, Faculty of Medical Sciences, The University of the West Indies	Nikita S. D. Sahadeo et al

EPI_ISL_756362	Trinidad Public Health Laboratory	Carrington Lab, Department of PreClinical Sciences, Faculty of Medical Sciences, The University of the West Indies	Nikita S. D. Sahadeo et al
EPI_ISL_717695	Trinidad Public Health Laboratory	Carrington Lab, Department of PreClinical Sciences, Faculty of Medical Sciences, The University of the West Indies	Nikita S. D. Sahadeo et al
EPI_ISL_717699	Trinidad Public Health Laboratory	Carrington Lab, Department of PreClinical Sciences, Faculty of Medical Sciences, The University of the West Indies	Nikita S. D. Sahadeo et al
EPI_ISL_756311	The Caribbean Public Health Agency	Carrington Lab, Department of PreClinical Sciences, Faculty of Medical Sciences, The University of the West Indies	Nikita S. D. Sahadeo et al
EPI_ISL_756357	The Caribbean Public Health Agency	Carrington Lab, Department of PreClinical Sciences, Faculty of Medical Sciences, The University of the West Indies	Nikita S. D. Sahadeo et al
EPI_ISL_717697	Trinidad Public Health Laboratory	Carrington Lab, Department of PreClinical Sciences, Faculty of Medical Sciences, The University of the West Indies	Nikita S. D. Sahadeo et al
EPI_ISL_756363	Trinidad Public Health Laboratory	Carrington Lab, Department of PreClinical Sciences, Faculty of Medical Sciences, The University of the West Indies	Nikita S. D. Sahadeo et al
EPI_ISL_717692	Trinidad Public Health Laboratory	Carrington Lab, Department of PreClinical Sciences, Faculty of Medical Sciences, The University of the West Indies	Nikita S. D. Sahadeo et al
EPI_ISL_717696	Trinidad Public Health Laboratory	Carrington Lab, Department of PreClinical Sciences, Faculty of Medical Sciences, The University of the West Indies	Nikita S. D. Sahadeo et al
EPI_ISL_717700	Trinidad Public Health Laboratory	Carrington Lab, Department of PreClinical Sciences, Faculty of Medical Sciences, The University of the West Indies	Nikita S. D. Sahadeo et al
EPI_ISL_717698	Trinidad Public Health Laboratory	Carrington Lab, Department of PreClinical Sciences, Faculty of Medical Sciences, The University of the West Indies	Nikita S. D. Sahadeo et al
EPI_ISL_481483	Department for Virology, Molecular Biology and Genome Research, R. G. Lugar Center for Public Health Research, National Center for Disease Control and Public Health (NCDC) of Georgia.	Department for Virology, Molecular Biology and Genome Research, R. G. Lugar Center for Public Health Research, National Center for Disease Control and Public Health (NCDC) of Georgia.	Nino Berishvili et al
EPI_ISL_596509	Palestinian Ministry of Health	Molecular Genetics Lab	Nouar Qutob et al
EPI_ISL_596522	Palestinian Ministry of Health	Molecular Genetics Lab	Nouar Qutob et al
EPI_ISL_596517	Palestinian Ministry of Health	Molecular Genetics Lab	Nouar Qutob et al
EPI_ISL_596519	Palestinian Ministry of Health	Molecular Genetics Lab	Nouar Qutob et al
EPI_ISL_596521	Palestinian Ministry of Health	Molecular Genetics Lab	Nouar Qutob et al
EPI_ISL_596525	Palestinian Ministry of Health	Molecular Genetics Lab	Nouar Qutob et al
EPI_ISL_596530	Palestinian Ministry of Health	Molecular Genetics Lab	Nouar Qutob et al
EPI_ISL_596531	Palestinian Ministry of Health	Molecular Genetics Lab	Nouar Qutob et al
EPI_ISL_596532	Palestinian Ministry of Health	Molecular Genetics Lab	Nouar Qutob et al
EPI_ISL_596534	Palestinian Ministry of Health	Molecular Genetics Lab	Nouar Qutob et al
EPI_ISL_596536	Palestinian Ministry of Health	Molecular Genetics Lab	Nouar Qutob et al
EPI_ISL_596542	Palestinian Ministry of Health	Molecular Genetics Lab	Nouar Qutob et al
EPI_ISL_596548	Palestinian Ministry of Health	Molecular Genetics Lab	Nouar Qutob et al

EPI_ISL_596550	Palestinian Ministry of Health	Molecular Genetics Lab	Nouar Qutob et al
EPI_ISL_596551	Palestinian Ministry of Health	Molecular Genetics Lab	Nouar Qutob et al
EPI_ISL_596552	Palestinian Ministry of Health	Molecular Genetics Lab	Nouar Qutob et al
EPI_ISL_596553	Palestinian Ministry of Health	Molecular Genetics Lab	Nouar Qutob et al
EPI_ISL_596554	Palestinian Ministry of Health	Molecular Genetics Lab	Nouar Qutob et al
EPI_ISL_596558	Palestinian Ministry of Health	Molecular Genetics Lab	Nouar Qutob et al
EPI_ISL_596560	Palestinian Ministry of Health	Molecular Genetics Lab	Nouar Qutob et al
EPI_ISL_596561	Palestinian Ministry of Health	Molecular Genetics Lab	Nouar Qutob et al
EPI_ISL_596567	Palestinian Ministry of Health	Molecular Genetics Lab	Nouar Qutob et al
EPI_ISL_804815	DB Diagnosticos do Brasil	Laboratório de Parasitologia Médica - Instituto de Medicina Tropical - Universidade de São Paulo	Nuno Faria et al
EPI_ISL_804824	DB Diagnosticos do Brasil	Laboratório de Parasitologia Médica - Instituto de Medicina Tropical - Universidade de São Paulo	Nuno Faria et al
EPI_ISL_804832	DB Diagnosticos do Brasil	Laboratório de Parasitologia Médica - Instituto de Medicina Tropical - Universidade de São Paulo	Nuno Faria et al
EPI_ISL_804835	DB Diagnosticos do Brasil	Laboratório de Parasitologia Médica - Instituto de Medicina Tropical - Universidade de São Paulo	Nuno Faria et al
EPI_ISL_804829	DB Diagnosticos do Brasil	Laboratório de Parasitologia Médica - Instituto de Medicina Tropical - Universidade de São Paulo	Nuno Faria et al
EPI_ISL_804833	DB Diagnosticos do Brasil	Laboratório de Parasitologia Médica - Instituto de Medicina Tropical - Universidade de São Paulo	Nuno Faria et al
EPI_ISL_583897	Singapore General Hospital	Department of Microbiology	Nurdyana Abdul Rahman et al
EPI_ISL_583900	Singapore General Hospital	Department of Microbiology	Nurdyana Abdul Rahman et al
EPI_ISL_482679	Singapore General Hospital	Department of Microbiology	Nurdyana Abdul Rahman et al
EPI_ISL_500570	Singapore General Hospital	Department of Microbiology	Nurdyana Abdul Rahman et al
EPI_ISL_518003	Singapore General Hospital	Department of Microbiology	Nurdyana Abdul Rahman et al
EPI_ISL_1014682	National Influenza Center, Virology Department	National Influenza Center	NZ Shafiei Jandaghi et al
EPI_ISL_862081	National Influenza Center, Virology Department	National Influenza Center	NZ Shafiei Jandaghi et al
EPI_ISL_1014686	National Influenza Center, Virology Department	National Influenza Center	NZ Shafiei Jandaghi et al
EPI_ISL_539259	Hospital Universitario de La Ribera (Alzira, València)	SeqCOVID-SPAIN consortium/IBV(CSIC)	Olalla Martínez Macias et al
EPI_ISL_1093432	Nigerian Centre for Disease Control (NCDC)	African Centre of Excellence for Genomics of Infectious Diseases (ACEGID), Redeemer’s University	Olawoye et al
EPI_ISL_1093450	Nigerian Centre for Disease Control (NCDC)	African Centre of Excellence for Genomics of Infectious Diseases (ACEGID), Redeemer’s University	Olawoye et al
EPI_ISL_1173210	Nigeria Centre for Disease Control (NCDC)	African Centre for Excellence for Genomics of Infectious Diseases (ACEGID), Redeemer’s University	Olawoye et al

EPI_ISL_1173243	Nigeria Centre for Disease Control (NCDC)	African Centre for Excellence for Genomics of Infectious Diseases (ACEGID), Redeemer’s University	Olawoye et al
EPI_ISL_1235644	Nigerian Centre for Disease Control (NCDC)	African Centre of Excellence for Genomics of Infectious Diseases (ACEGID), Redeemer’s University	Olawoye et al
EPI_ISL_1235666	Nigerian Centre for Disease Control (NCDC)	African Centre of Excellence for Genomics of Infectious Diseases (ACEGID), Redeemer’s University	Olawoye et al
EPI_ISL_428916	State Research Center of Virology and Biotechnology VECTOR, Department of Collection of Microorganisms	State Research Center of Virology and Biotechnology VECTOR, Department of Collection of Microorganisms	Oleg V. Pyankov et al
EPI_ISL_428893	State Research Center of Virology and Biotechnology VECTOR, Department of Collection of Microorganisms	State Research Center of Virology and Biotechnology VECTOR, Department of Collection of Microorganisms	Oleg V. Pyankov et al
EPI_ISL_428883	State Research Center of Virology and Biotechnology VECTOR, Department of Collection of Microorganisms	State Research Center of Virology and Biotechnology VECTOR, Department of Collection of Microorganisms	Oleg V. Pyankov et al
EPI_ISL_845548	National Public Health Laboratory, Cameroon	African Centre of Excellence for Genomics of Infectious Diseases (ACEGID), Redeemer's University	Oluniyi P.E. et al
EPI_ISL_845546	National Public Health Laboratory, Cameroon	African Centre of Excellence for Genomics of Infectious Diseases (ACEGID), Redeemer's University	Oluniyi P.E. et al
EPI_ISL_845549	National Public Health Laboratory, Cameroon	African Centre of Excellence for Genomics of Infectious Diseases (ACEGID), Redeemer's University	Oluniyi P.E. et al
EPI_ISL_845551	National Public Health Laboratory, Cameroon	African Centre of Excellence for Genomics of Infectious Diseases (ACEGID), Redeemer's University	Oluniyi P.E. et al
EPI_ISL_845550	National Public Health Laboratory, Cameroon	African Centre of Excellence for Genomics of Infectious Diseases (ACEGID), Redeemer's University	Oluniyi P.E. et al
EPI_ISL_845553	National Public Health Laboratory, Cameroon	African Centre of Excellence for Genomics of Infectious Diseases (ACEGID), Redeemer's University	Oluniyi P.E. et al
EPI_ISL_845552	National Public Health Laboratory, Cameroon	African Centre of Excellence for Genomics of Infectious Diseases (ACEGID), Redeemer's University	Oluniyi P.E. et al
EPI_ISL_845554	National Public Health Laboratory, Cameroon	African Centre of Excellence for Genomics of Infectious Diseases (ACEGID), Redeemer's University	Oluniyi P.E. et al
EPI_ISL_845564	National Public Health Laboratory, Cameroon	African Centre of Excellence for Genomics of Infectious Diseases (ACEGID), Redeemer's University	Oluniyi P.E. et al
EPI_ISL_845563	National Public Health Laboratory, Cameroon	African Centre of Excellence for Genomics of Infectious Diseases (ACEGID), Redeemer's University	Oluniyi P.E. et al
EPI_ISL_845565	National Public Health Laboratory, Cameroon	African Centre of Excellence for Genomics of Infectious Diseases (ACEGID), Redeemer's University	Oluniyi P.E. et al
EPI_ISL_845562	National Public Health Laboratory, Cameroon	African Centre of Excellence for Genomics of Infectious Diseases (ACEGID), Redeemer's University	Oluniyi P.E. et al
EPI_ISL_845560	National Public Health Laboratory, Cameroon	African Centre of Excellence for Genomics of Infectious Diseases (ACEGID), Redeemer's University	Oluniyi P.E. et al
EPI_ISL_845561	National Public Health Laboratory, Cameroon	African Centre of Excellence for Genomics of Infectious Diseases (ACEGID), Redeemer's University	Oluniyi P.E. et al
EPI_ISL_845558	National Public Health Laboratory, Cameroon	African Centre of Excellence for Genomics of Infectious Diseases (ACEGID), Redeemer's University	Oluniyi P.E. et al
EPI_ISL_729993	Nigeria Centre for Disease Control (NCDC)	African Centre of Excellence for Genomics of Infectious Diseases (ACEGID), Redeemer's University, Ede, Osun State, Nigeria	Oluniyi P.E. et al

EPI_ISL_729959	Nigeria Centre for Disease Control (NCDC)	African Centre of Excellence for Genomics of Infectious Diseases (ACEGID), Redeemer's University, Ede, Osun State, Nigeria	Oluniyi P.E. et al
EPI_ISL_729995	Nigeria Centre for Disease Control (NCDC)	African Centre of Excellence for Genomics of Infectious Diseases (ACEGID), Redeemer's University, Ede, Osun State, Nigeria	Oluniyi P.E. et al
EPI_ISL_729997	Nigeria Centre for Disease Control (NCDC)	African Centre of Excellence for Genomics of Infectious Diseases (ACEGID), Redeemer's University, Ede, Osun State, Nigeria	Oluniyi P.E. et al
EPI_ISL_729999	Nigeria Centre for Disease Control (NCDC)	African Centre of Excellence for Genomics of Infectious Diseases (ACEGID), Redeemer's University, Ede, Osun State, Nigeria	Oluniyi P.E. et al
EPI_ISL_730000	Nigeria Centre for Disease Control (NCDC)	African Centre of Excellence for Genomics of Infectious Diseases (ACEGID), Redeemer's University, Ede, Osun State, Nigeria	Oluniyi P.E. et al
EPI_ISL_730001	Nigeria Centre for Disease Control (NCDC)	African Centre of Excellence for Genomics of Infectious Diseases (ACEGID), Redeemer's University, Ede, Osun State, Nigeria	Oluniyi P.E. et al
EPI_ISL_730003	Nigeria Centre for Disease Control (NCDC)	African Centre of Excellence for Genomics of Infectious Diseases (ACEGID), Redeemer's University, Ede, Osun State, Nigeria	Oluniyi P.E. et al
EPI_ISL_730004	Nigeria Centre for Disease Control (NCDC)	African Centre of Excellence for Genomics of Infectious Diseases (ACEGID), Redeemer's University, Ede, Osun State, Nigeria	Oluniyi P.E. et al
EPI_ISL_730010	Nigeria Centre for Disease Control (NCDC)	African Centre of Excellence for Genomics of Infectious Diseases (ACEGID), Redeemer's University, Ede, Osun State, Nigeria	Oluniyi P.E. et al
EPI_ISL_730013	Nigeria Centre for Disease Control (NCDC)	African Centre of Excellence for Genomics of Infectious Diseases (ACEGID), Redeemer's University, Ede, Osun State, Nigeria	Oluniyi P.E. et al
EPI_ISL_730014	Nigeria Centre for Disease Control (NCDC)	African Centre of Excellence for Genomics of Infectious Diseases (ACEGID), Redeemer's University, Ede, Osun State, Nigeria	Oluniyi P.E. et al
EPI_ISL_906298	Nigeria Centre for Disease Control (NCDC)	African Centre of Excellence for Genomics of Infectious Diseases (ACEGID), Redeemer's University	Oluniyi P.E. et al
EPI_ISL_941277	Nigeria Centre for Disease Control (NCDC)	African Centre of Excellence for Genomics of Infectious Diseases (ACEGID), Redeemer's University	Oluniyi P.E. et al
EPI_ISL_941280	Nigeria Centre for Disease Control (NCDC)	African Centre of Excellence for Genomics of Infectious Diseases (ACEGID), Redeemer's University	Oluniyi P.E. et al
EPI_ISL_941281	Nigeria Centre for Disease Control (NCDC)	African Centre of Excellence for Genomics of Infectious Diseases (ACEGID), Redeemer's University	Oluniyi P.E. et al
EPI_ISL_941287	Nigeria Centre for Disease Control (NCDC)	African Centre of Excellence for Genomics of Infectious Diseases (ACEGID), Redeemer's University	Oluniyi P.E. et al
EPI_ISL_1242014	Nigeria Centre for Disease Control (NCDC)	African Centre of Excellence for Genomics of Infectious Diseases (ACEGID), Redeemer's University	Oluniyi P.E. et al
EPI_ISL_527875	Nigeria Centre for Disease Control (NCDC)	African Centre of Excellence for Genomics of Infectious Diseases (ACEGID), Redeemer's University, Ede, Osun State, Nigeria	Oluniyi P.E. et al
EPI_ISL_729921	Nigeria Centre for Disease Control (NCDC)	African Centre of Excellence for Genomics of Infectious Diseases (ACEGID), Redeemer's University, Ede, Osun State, Nigeria	Oluniyi P.E. et al
EPI_ISL_729924	Nigeria Centre for Disease Control (NCDC)	African Centre of Excellence for Genomics of Infectious Diseases (ACEGID), Redeemer's University, Ede, Osun State, Nigeria	Oluniyi P.E. et al
EPI_ISL_729931	Nigeria Centre for Disease Control (NCDC)	African Centre of Excellence for Genomics of Infectious Diseases (ACEGID), Redeemer's University, Ede, Osun State, Nigeria	Oluniyi P.E. et al
EPI_ISL_729933	Nigeria Centre for Disease Control (NCDC)	African Centre of Excellence for Genomics of Infectious Diseases (ACEGID), Redeemer's University, Ede, Osun State, Nigeria	Oluniyi P.E. et al

EPI_ISL_872603	Nigeria Centre for Disease Control (NCDC)	African Centre of Excellence for Genomics of Infectious Diseases (ACEGID), Redeemer's University	Oluniyi P.E. et al
EPI_ISL_872604	Nigeria Centre for Disease Control (NCDC)	African Centre of Excellence for Genomics of Infectious Diseases (ACEGID), Redeemer's University	Oluniyi P.E. et al
EPI_ISL_872606	Nigeria Centre for Disease Control (NCDC)	African Centre of Excellence for Genomics of Infectious Diseases (ACEGID), Redeemer's University	Oluniyi P.E. et al
EPI_ISL_872609	Nigeria Centre for Disease Control (NCDC)	African Centre of Excellence for Genomics of Infectious Diseases (ACEGID), Redeemer's University	Oluniyi P.E. et al
EPI_ISL_872611	Nigeria Centre for Disease Control (NCDC)	African Centre of Excellence for Genomics of Infectious Diseases (ACEGID), Redeemer's University	Oluniyi P.E. et al
EPI_ISL_872622	Nigeria Centre for Disease Control (NCDC)	African Centre of Excellence for Genomics of Infectious Diseases (ACEGID), Redeemer's University	Oluniyi P.E. et al
EPI_ISL_872624	Nigeria Centre for Disease Control (NCDC)	African Centre of Excellence for Genomics of Infectious Diseases (ACEGID), Redeemer's University	Oluniyi P.E. et al
EPI_ISL_730023	Nigeria Centre for Disease Control (NCDC)	African Centre of Excellence for Genomics of Infectious Diseases (ACEGID), Redeemer's University, Ede, Osun State, Nigeria	Oluniyi P.E. et al
EPI_ISL_730047	Nigeria Centre for Disease Control (NCDC)	African Centre of Excellence for Genomics of Infectious Diseases (ACEGID), Redeemer's University, Ede, Osun State, Nigeria	Oluniyi P.E. et al
EPI_ISL_730048	Nigeria Centre for Disease Control (NCDC)	African Centre of Excellence for Genomics of Infectious Diseases (ACEGID), Redeemer's University, Ede, Osun State, Nigeria	Oluniyi P.E. et al
EPI_ISL_729946	Nigeria Centre for Disease Control (NCDC)	African Centre of Excellence for Genomics of Infectious Diseases (ACEGID), Redeemer's University, Ede, Osun State, Nigeria	Oluniyi P.E. et al
EPI_ISL_729947	Nigeria Centre for Disease Control (NCDC)	African Centre of Excellence for Genomics of Infectious Diseases (ACEGID), Redeemer's University, Ede, Osun State, Nigeria	Oluniyi P.E. et al
EPI_ISL_730042	Nigeria Centre for Disease Control (NCDC)	African Centre of Excellence for Genomics of Infectious Diseases (ACEGID), Redeemer's University, Ede, Osun State, Nigeria	Oluniyi P.E. et al
EPI_ISL_413550	Centre for Human and Zoonotic Virology (CHAZVY), College of Medicine University of Lagos/Lagos University Teaching Hospital (LUTH), part of the Laboratory Network of the Nigeria Centre for Disease Control (NCDC)	African Centre of Excellence for Genomics of Infectious Diseases (ACEGID), Redeemer's University, Ede, Osun State, Nigeria	Oluniyi P.E. et al
EPI_ISL_729967	Nigeria Centre for Disease Control (NCDC)	African Centre of Excellence for Genomics of Infectious Diseases (ACEGID), Redeemer's University, Ede, Osun State, Nigeria	Oluniyi P.E. et al
EPI_ISL_729978	Nigeria Centre for Disease Control (NCDC)	African Centre of Excellence for Genomics of Infectious Diseases (ACEGID), Redeemer's University, Ede, Osun State, Nigeria	Oluniyi P.E. et al
EPI_ISL_729979	Nigeria Centre for Disease Control (NCDC)	African Centre of Excellence for Genomics of Infectious Diseases (ACEGID), Redeemer's University, Ede, Osun State, Nigeria	Oluniyi P.E. et al
EPI_ISL_729980	Nigeria Centre for Disease Control (NCDC)	African Centre of Excellence for Genomics of Infectious Diseases (ACEGID), Redeemer's University, Ede, Osun State, Nigeria	Oluniyi P.E. et al
EPI_ISL_729981	Nigeria Centre for Disease Control (NCDC)	African Centre of Excellence for Genomics of Infectious Diseases (ACEGID), Redeemer's University, Ede, Osun State, Nigeria	Oluniyi P.E. et al
EPI_ISL_729982	Nigeria Centre for Disease Control (NCDC)	African Centre of Excellence for Genomics of Infectious Diseases (ACEGID), Redeemer's University, Ede, Osun State, Nigeria	Oluniyi P.E. et al
EPI_ISL_729984	Nigeria Centre for Disease Control (NCDC)	African Centre of Excellence for Genomics of Infectious Diseases (ACEGID), Redeemer's University, Ede, Osun State, Nigeria	Oluniyi P.E. et al

EPI_ISL_729985	Nigeria Centre for Disease Control (NCDC)	African Centre of Excellence for Genomics of Infectious Diseases (ACEGID), Redeemer's University, Ede, Osun State, Nigeria	Oluniyi P.E. et al
EPI_ISL_455415	Nigeria Centre for Disease Control (NCDC)	African Centre of Excellence for Genomics of Infectious Diseases (ACEGID), Redeemer's University, Ede, Osun State, Nigeria	Oluniyi P.E. et al
EPI_ISL_455418	Nigeria Centre for Disease Control (NCDC)	African Centre of Excellence for Genomics of Infectious Diseases (ACEGID), Redeemer's University, Ede, Osun State, Nigeria	Oluniyi P.E. et al
EPI_ISL_455422	Nigeria Centre for Disease Control	African Centre of Excellence for Genomics of Infectious Diseases (ACEGID), Redeemer's University, Ede, Osun State, Nigeria	Oluniyi P.E. et al
EPI_ISL_455423	Nigeria Centre for Disease Control (NCDC)	African Centre of Excellence for Genomics of Infectious Diseases (ACEGID), Redeemer's University, Ede, Osun State, Nigeria	Oluniyi P.E. et al
EPI_ISL_455427	Nigeria Centre for Disease Control (NCDC)	African Centre of Excellence for Genomics of Infectious Diseases (ACEGID), Redeemer's University, Ede, Osun State, Nigeria	Oluniyi P.E. et al
EPI_ISL_527895	Nigeria Centre for Disease Control (NCDC)	African Centre of Excellence for Genomics of Infectious Diseases (ACEGID), Redeemer's University, Ede, Osun State, Nigeria	Oluniyi P.E. et al
EPI_ISL_527898	Nigeria Centre for Disease Control (NCDC)	African Centre of Excellence for Genomics of Infectious Diseases (ACEGID), Redeemer's University, Ede, Osun State, Nigeria	Oluniyi P.E. et al
EPI_ISL_527899	Nigeria Centre for Disease Control (NCDC)	African Centre of Excellence for Genomics of Infectious Diseases (ACEGID), Redeemer's University, Ede, Osun State, Nigeria	Oluniyi P.E. et al
EPI_ISL_527902	Nigeria Centre for Disease Control (NCDC)	African Centre of Excellence for Genomics of Infectious Diseases (ACEGID), Redeemer's University, Ede, Osun State, Nigeria	Oluniyi P.E. et al
EPI_ISL_872605	Nigeria Centre for Disease Control (NCDC)	African Centre of Excellence for Genomics of Infectious Diseases (ACEGID), Redeemer's University	Oluniyi P.E. et al
EPI_ISL_626228	Institute for Virology, University Hospital Essen	Center of Medical Microbiology, Virology, and Hospital Hygiene, University of Duesseldorf	Olympia E. Anastasiou et al
EPI_ISL_1385804	Alfa Diagnostica, Republic of Moldova	ONCOGENE LLC	ONCOGENE LLC et al
EPI_ISL_1385807	Alfa Diagnostica LLC	ONCOGENE LLC	ONCOGENE LLC et al
EPI_ISL_1385811	Alfa Diagnostica LLC	ONCOGENE LLC	ONCOGENE LLC et al
EPI_ISL_707938	Pamukkale University Hospital	Pamukkale University Department of Medical Genetics	Onur TOKGUN et al.
EPI_ISL_707939	Pamukkale University Hospital	Pamukkale University Department of Medical Genetics	Onur TOKGUN et al.
EPI_ISL_708185	Pamukkale University Hospital	Pamukkale University Department of Medical Genetics	Onur TOKGUN et al.
EPI_ISL_707937	Pamukkale University Hospital	Pamukkale University Department of Medical Genetics	Onur TOKGUN et al.
EPI_ISL_708190	Pamukkale University Hospital	Pamukkale University Department of Medical Genetics	Onur TOKGUN et al.
EPI_ISL_708191	Pamukkale University Hospital	Pamukkale University Department of Medical Genetics	Onur TOKGUN et al.
EPI_ISL_708193	Pamukkale University Hospital	Pamukkale University Department of Medical Genetics	Onur TOKGUN et al.
EPI_ISL_849738	Special Operations Medical Research Division, Defence Services Medical Research Centre	Special Operations Medical Research Division, Defence Services Medical Research Centre	Oo et al
EPI_ISL_849739	Special Operations Medical Research Division, Defence Services Medical Research Centre	Special Operations Medical Research Division, Defence Services Medical Research Centre	Oo et al
EPI_ISL_849740	Special Operations Medical Research Division, Defence Services Medical Research Centre	Special Operations Medical Research Division, Defence Services Medical Research Centre	Oo et al

EPI_ISL_849741	Special Operations Medical Research Division, Defence Services Medical Research Centre	Special Operations Medical Research Division, Defence Services Medical Research Centre	Oo et al
EPI_ISL_849742	Special Operations Medical Research Division, Defence Services Medical Research Centre	Special Operations Medical Research Division, Defence Services Medical Research Centre	Oo et al
EPI_ISL_849743	Special Operations Medical Research Division, Defence Services Medical Research Centre	Special Operations Medical Research Division, Defence Services Medical Research Centre	Oo et al
EPI_ISL_413488	Center of Medical Microbiology, Virology, and Hospital Hygiene, University of Duesseldorf	Center of Medical Microbiology, Virology, and Hospital Hygiene, University of Duesseldorf	Ortwin Adams et al
EPI_ISL_414506	Center of Medical Microbiology, Virology, and Hospital Hygiene, University of Duesseldorf	Center of Medical Microbiology, Virology, and Hospital Hygiene, University of Duesseldorf	Ortwin Adams et al
EPI_ISL_414508	Center of Medical Microbiology, Virology, and Hospital Hygiene, University of Duesseldorf	Center of Medical Microbiology, Virology, and Hospital Hygiene, University of Duesseldorf	Ortwin Adams et al
EPI_ISL_414574	Center of Medical Microbiology, Virology, and Hospital Hygiene, University of Duesseldorf	Center of Medical Microbiology, Virology, and Hospital Hygiene, University of Duesseldorf	Ortwin Adams et al
EPI_ISL_1250842	Clin & Gen Lab	Molecular Genetics Laboratory, Instituto de Investigaciones Químicas, Universidad Mayor de San Andrés	Oscar M. Rollano-Peñaloza et al
EPI_ISL_1278275	Clin & Gen Lab	Molecular Genetics Laboratory, Instituto de Investigaciones Químicas, Universidad Mayor de San Andrés	Oscar M. Rollano-Peñaloza et al
EPI_ISL_1278276	Clin & Gen Lab	Molecular Genetics Laboratory, Instituto de Investigaciones Químicas, Universidad Mayor de San Andrés	Oscar M. Rollano-Peñaloza et al
EPI_ISL_1363787	Clin & Gen Lab	Microbiologia Molecular, Instituto SELADIS, Universidad Mayor de San Andrés	Oscar M. Rollano-Peñaloza et al
EPI_ISL_1278281	Laboratorio de Biologia Molecular, SEDES-Potosi	Molecular Genetics Laboratory, Instituto de Investigaciones Químicas, Universidad Mayor de San Andrés	Oscar M. Rollano-Peñaloza et al
EPI_ISL_1278282	Laboratorio de Biologia Molecular, SEDES-Potosi	Molecular Genetics Laboratory, Instituto de Investigaciones Químicas, Universidad Mayor de San Andrés	Oscar M. Rollano-Peñaloza et al
EPI_ISL_1278283	Laboratorio de Biologia Molecular, SEDES-Potosi	Molecular Genetics Laboratory, Instituto de Investigaciones Químicas, Universidad Mayor de San Andrés	Oscar M. Rollano-Peñaloza et al
EPI_ISL_1278284	Laboratorio de Biologia Molecular, SEDES-Potosi	Molecular Genetics Laboratory, Instituto de Investigaciones Químicas, Universidad Mayor de San Andrés	Oscar M. Rollano-Peñaloza et al
EPI_ISL_1278277	Laboratorio de Biologia Molecular, Hospital San Pedro Claver	Molecular Genetics Laboratory, Instituto de Investigaciones Químicas, Universidad Mayor de San Andrés	Oscar M. Rollano-Peñaloza et al
EPI_ISL_1278278	Laboratorio de Biologia Molecular, Hospital San Pedro Claver	Molecular Genetics Laboratory, Instituto de Investigaciones Químicas, Universidad Mayor de San Andrés	Oscar M. Rollano-Peñaloza et al
EPI_ISL_476139	Folkhalsomyndigheten	The Public Health Agency of Sweden	Oskar Karlsson Lindsjo et al
EPI_ISL_475544	Karolinska Universitetslaboratoriet	The Public Health Agency of Sweden	Oskar Karlsson Lindsjo et al
EPI_ISL_475545	Karolinska Universitetslaboratoriet	The Public Health Agency of Sweden	Oskar Karlsson Lindsjo et al
EPI_ISL_475546	Karolinska Universitetslaboratoriet	The Public Health Agency of Sweden	Oskar Karlsson Lindsjo et al
EPI_ISL_475547	Karolinska Universitetslaboratoriet	The Public Health Agency of Sweden	Oskar Karlsson Lindsjo et al
EPI_ISL_475557	Halmstad klinisk mikrobiologi	The Public Health Agency of Sweden	Oskar Karlsson Lindsjo et al
EPI_ISL_475511	Orestadsklinikens VC	The Public Health Agency of Sweden	Oskar Karlsson Lindsjo et al

EPI_ISL_469060	Narhalsan Sjobo vardcentral	The Public Health Agency of Sweden	Oskar Karlsson Lindsjo et al
EPI_ISL_469061	Narhalsan Sjobo vardcentral	The Public Health Agency of Sweden	Oskar Karlsson Lindsjo et al
EPI_ISL_475559	Karolinska Universitetslaboratoriet	The Public Health Agency of Sweden	Oskar Karlsson Lindsjo et al
EPI_ISL_510831	Halmstad klinisk mikrobiologi	The Public Health Agency of Sweden	Oskar Karlsson Lindsjo et al
EPI_ISL_510842	Klinisk mikrobiologi NAL Trollhattan	The Public Health Agency of Sweden	Oskar Karlsson Lindsjo et al
EPI_ISL_510850	Skovde/Unilabs	The Public Health Agency of Sweden	Oskar Karlsson Lindsjo et al
EPI_ISL_509413	Acibadem Labcell Cellular Therapy Laboratory	Acibadem Mehmet Ali Aydinlar University School of Medicine, Medical Genetics Department	Ozden Hatirnaz Ng et al
EPI_ISL_949630	NHLS Universitas Academic	UFS Virology	PA Bester et al
EPI_ISL_912467	NHLS Universitas Academic	UFS Virology	PA Bester et al
EPI_ISL_729906	Instituto de Medicina Tropical, Universidad Nacional Toribio Rodríguez de Mendoza de Amazonas	Laboratorio de Genómica Microbiana, Universidad Peruana Cayetano Heredia	Pablo Tsukayama et al
EPI_ISL_729907	Instituto de Medicina Tropical, Universidad Nacional Toribio Rodríguez de Mendoza de Amazonas	Laboratorio de Genómica Microbiana, Universidad Peruana Cayetano Heredia	Pablo Tsukayama et al
EPI_ISL_729908	Instituto de Medicina Tropical, Universidad Nacional Toribio Rodríguez de Mendoza de Amazonas	Laboratorio de Genómica Microbiana, Universidad Peruana Cayetano Heredia	Pablo Tsukayama et al
EPI_ISL_729909	Instituto de Medicina Tropical, Universidad Nacional Toribio Rodríguez de Mendoza de Amazonas	Laboratorio de Genómica Microbiana, Universidad Peruana Cayetano Heredia	Pablo Tsukayama et al
EPI_ISL_729912	Instituto de Medicina Tropical, Universidad Nacional Toribio Rodríguez de Mendoza de Amazonas	Laboratorio de Genómica Microbiana, Universidad Peruana Cayetano Heredia	Pablo Tsukayama et al
EPI_ISL_729915	Instituto de Medicina Tropical, Universidad Nacional Toribio Rodríguez de Mendoza de Amazonas	Laboratorio de Genómica Microbiana, Universidad Peruana Cayetano Heredia	Pablo Tsukayama et al
EPI_ISL_729916	Instituto de Medicina Tropical, Universidad Nacional Toribio Rodríguez de Mendoza de Amazonas	Laboratorio de Genómica Microbiana, Universidad Peruana Cayetano Heredia	Pablo Tsukayama et al
EPI_ISL_729871	Instituto de Medicina Tropical, Universidad Nacional Toribio Rodríguez de Mendoza de Amazonas	Laboratorio de Genómica Microbiana, Universidad Peruana Cayetano Heredia	Pablo Tsukayama et al
EPI_ISL_529067	Laboratorio de Referencia Nacional de Virus Respiratorios, Instituto Nacional de Salud Peru	Laboratorio de Genómica Microbiana, Universidad Peruana Cayetano Heredia	Pablo Tsukayama et al
EPI_ISL_540926	Laboratorio de Referencia Nacional de Virus Respiratorios, Instituto Nacional de Salud Peru	Laboratorio de Genómica Microbiana, Universidad Peruana Cayetano Heredia	Pablo Tsukayama et al
EPI_ISL_540934	Laboratorio de Referencia Nacional de Virus Respiratorios, Instituto Nacional de Salud Peru	Laboratorio de Genómica Microbiana, Universidad Peruana Cayetano Heredia	Pablo Tsukayama et al
EPI_ISL_529075	Laboratorio de Referencia Nacional de Virus Respiratorios, Instituto Nacional de Salud Peru	Laboratorio de Genómica Microbiana, Universidad Peruana Cayetano Heredia	Pablo Tsukayama et al
EPI_ISL_540945	Laboratorio de Referencia Nacional de Virus Respiratorios, Instituto Nacional de Salud Peru	Laboratorio de Genómica Microbiana, Universidad Peruana Cayetano Heredia	Pablo Tsukayama et al
EPI_ISL_540952	Laboratorio de Referencia Nacional de Virus Respiratorios, Instituto Nacional de Salud Peru	Laboratorio de Genómica Microbiana, Universidad Peruana Cayetano Heredia	Pablo Tsukayama et al
EPI_ISL_540959	Laboratorio de Referencia Nacional de Virus Respiratorios, Instituto Nacional de Salud Peru	Laboratorio de Genómica Microbiana, Universidad Peruana Cayetano Heredia	Pablo Tsukayama et al

EPI_ISL_540992	Laboratorio de Referencia Nacional de Virus Respiratorios, Instituto Nacional de Salud Peru	Laboratorio de Genómica Microbiana, Universidad Peruana Cayetano Heredia	Pablo Tsukayama et al
EPI_ISL_568517	Laboratorio de Referencia Nacional de Virus Respiratorios, Instituto Nacional de Salud Peru	Laboratorio de Genómica Microbiana, Universidad Peruana Cayetano Heredia	Pablo Tsukayama et al
EPI_ISL_568527	Laboratorio de Referencia Nacional de Virus Respiratorios, Instituto Nacional de Salud Peru	Laboratorio de Genómica Microbiana, Universidad Peruana Cayetano Heredia	Pablo Tsukayama et al
EPI_ISL_568536	Laboratorio de Referencia Nacional de Virus Respiratorios, Instituto Nacional de Salud Peru	Laboratorio de Genómica Microbiana, Universidad Peruana Cayetano Heredia	Pablo Tsukayama et al
EPI_ISL_729872	Laboratorio de Referencia Nacional de Virus Respiratorios, Instituto Nacional de Salud Peru	Laboratorio de Genómica Microbiana, Universidad Peruana Cayetano Heredia	Pablo Tsukayama et al
EPI_ISL_729875	Laboratorio de Referencia Nacional de Virus Respiratorios, Instituto Nacional de Salud Peru	Laboratorio de Genómica Microbiana, Universidad Peruana Cayetano Heredia	Pablo Tsukayama et al
EPI_ISL_729876	Laboratorio de Referencia Nacional de Virus Respiratorios, Instituto Nacional de Salud Peru	Laboratorio de Genómica Microbiana, Universidad Peruana Cayetano Heredia	Pablo Tsukayama et al
EPI_ISL_729896	Laboratorio de Referencia Nacional de Virus Respiratorios, Instituto Nacional de Salud Peru	Laboratorio de Genómica Microbiana, Universidad Peruana Cayetano Heredia	Pablo Tsukayama et al
EPI_ISL_729897	Laboratorio de Referencia Nacional de Virus Respiratorios, Instituto Nacional de Salud Peru	Laboratorio de Genómica Microbiana, Universidad Peruana Cayetano Heredia	Pablo Tsukayama et al
EPI_ISL_862476	Kurnool Medical College (KMC)	CSIR Institute of Genomics and Integrative Biology	Pallavali Roja Rani et al
EPI_ISL_862479	Kurnool Medical College (KMC)	CSIR Institute of Genomics and Integrative Biology	Pallavali Roja Rani et al
EPI_ISL_1424519	Queensland Medical Laboratories	Victorian Infectious Diseases Reference Laboratory (VIDRL) and the Melbourne Diagnostic Unit Public Health Laboratory (MDU-PHL)	Palou et al
EPI_ISL_1424521	Queensland Medical Laboratories	Victorian Infectious Diseases Reference Laboratory (VIDRL) and the Melbourne Diagnostic Unit Public Health Laboratory (MDU-PHL)	Palou et al
EPI_ISL_1424523	Queensland Medical Laboratories	Victorian Infectious Diseases Reference Laboratory (VIDRL) and the Melbourne Diagnostic Unit Public Health Laboratory (MDU-PHL)	Palou et al
EPI_ISL_1424526	Queensland Medical Laboratories	Victorian Infectious Diseases Reference Laboratory (VIDRL) and the Melbourne Diagnostic Unit Public Health Laboratory (MDU-PHL)	Palou et al
EPI_ISL_1424540	Queensland Medical Laboratories	Victorian Infectious Diseases Reference Laboratory (VIDRL) and the Melbourne Diagnostic Unit Public Health Laboratory (MDU-PHL)	Palou et al
EPI_ISL_1424544	Queensland Medical Laboratories	Victorian Infectious Diseases Reference Laboratory (VIDRL) and the Melbourne Diagnostic Unit Public Health Laboratory (MDU-PHL)	Palou et al
EPI_ISL_1424584	Queensland Medical Laboratories	Victorian Infectious Diseases Reference Laboratory (VIDRL) and the Melbourne Diagnostic Unit Public Health Laboratory (MDU-PHL)	Palou et al
EPI_ISL_1424591	Queensland Medical Laboratories	Victorian Infectious Diseases Reference Laboratory (VIDRL) and the Melbourne Diagnostic Unit Public Health Laboratory (MDU-PHL)	Palou et al
EPI_ISL_1424592	Queensland Medical Laboratories	Victorian Infectious Diseases Reference Laboratory (VIDRL) and the Melbourne Diagnostic Unit Public Health Laboratory (MDU-PHL)	Palou et al
EPI_ISL_1424615	Queensland Medical Laboratories	Victorian Infectious Diseases Reference Laboratory (VIDRL) and the Melbourne Diagnostic Unit Public Health Laboratory (MDU-PHL)	Palou et al
EPI_ISL_1424616	Queensland Medical Laboratories	Victorian Infectious Diseases Reference Laboratory (VIDRL) and the Melbourne Diagnostic Unit Public Health Laboratory (MDU-PHL)	Palou et al

EPI_ISL_1424618	Queensland Medical Laboratories	Victorian Infectious Diseases Reference Laboratory (VIDRL) and the Melbourne Diagnostic Unit Public Health Laboratory (MDU-PHL)	Palou et al
EPI_ISL_1424625	Queensland Medical Laboratories	Victorian Infectious Diseases Reference Laboratory (VIDRL) and the Melbourne Diagnostic Unit Public Health Laboratory (MDU-PHL)	Palou et al
EPI_ISL_1424635	Queensland Medical Laboratories	Victorian Infectious Diseases Reference Laboratory (VIDRL) and the Melbourne Diagnostic Unit Public Health Laboratory (MDU-PHL)	Palou et al
EPI_ISL_1424680	Queensland Medical Laboratories	Melbourne Diagnostic Unit Public Health Laboratory (MDU-PHL)	Palou et al
EPI_ISL_1296263	Azienda Ospedaliera Terni	Istituto Zooprofilattico Sperimentale dell'Abruzzo e Molise "G. Caporale"	Palumbo M et al
EPI_ISL_1299525	Azienda Ospedaliera Terni	Istituto Zooprofilattico Sperimentale dell'Abruzzo e Molise "G. Caporale"	Palumbo m et al
EPI_ISL_1415419	Reference Laboratory of the Ministry of Health	Laboratory of Respiratory Viruses and Measles, Oswaldo Cruz Institute, FIOCRUZ	Paola Resende et al
EPI_ISL_1415418	Reference Laboratory of the Ministry of Health	Laboratory of Respiratory Viruses and Measles, Oswaldo Cruz Institute, FIOCRUZ	Paola Resende et al
EPI_ISL_1415417	Reference Laboratory of the Ministry of Health	Laboratory of Respiratory Viruses and Measles, Oswaldo Cruz Institute, FIOCRUZ	Paola Resende et al
EPI_ISL_1415416	Reference Laboratory of the Ministry of Health	Laboratory of Respiratory Viruses and Measles, Oswaldo Cruz Institute, FIOCRUZ	Paola Resende et al
EPI_ISL_1415415	Reference Laboratory of the Ministry of Health	Laboratory of Respiratory Viruses and Measles, Oswaldo Cruz Institute, FIOCRUZ	Paola Resende et al
EPI_ISL_1415414	Reference Laboratory of the Ministry of Health	Laboratory of Respiratory Viruses and Measles, Oswaldo Cruz Institute, FIOCRUZ	Paola Resende et al
EPI_ISL_1415413	Reference Laboratory of the Ministry of Health	Laboratory of Respiratory Viruses and Measles, Oswaldo Cruz Institute, FIOCRUZ	Paola Resende et al
EPI_ISL_1415412	Reference Laboratory of the Ministry of Health	Laboratory of Respiratory Viruses and Measles, Oswaldo Cruz Institute, FIOCRUZ	Paola Resende et al
EPI_ISL_1415411	Reference Laboratory of the Ministry of Health	Laboratory of Respiratory Viruses and Measles, Oswaldo Cruz Institute, FIOCRUZ	Paola Resende et al
EPI_ISL_1415410	Reference Laboratory of the Ministry of Health	Laboratory of Respiratory Viruses and Measles, Oswaldo Cruz Institute, FIOCRUZ	Paola Resende et al
EPI_ISL_1415409	Reference Laboratory of the Ministry of Health	Laboratory of Respiratory Viruses and Measles, Oswaldo Cruz Institute, FIOCRUZ	Paola Resende et al
EPI_ISL_1415408	Reference Laboratory of the Ministry of Health	Laboratory of Respiratory Viruses and Measles, Oswaldo Cruz Institute, FIOCRUZ	Paola Resende et al
EPI_ISL_1415407	Reference Laboratory of the Ministry of Health	Laboratory of Respiratory Viruses and Measles, Oswaldo Cruz Institute, FIOCRUZ	Paola Resende et al
EPI_ISL_1415406	Reference Laboratory of the Ministry of Health	Laboratory of Respiratory Viruses and Measles, Oswaldo Cruz Institute, FIOCRUZ	Paola Resende et al
EPI_ISL_1415404	Reference Laboratory of the Ministry of Health	Laboratory of Respiratory Viruses and Measles, Oswaldo Cruz Institute, FIOCRUZ	Paola Resende et al
EPI_ISL_1415403	Reference Laboratory of the Ministry of Health	Laboratory of Respiratory Viruses and Measles, Oswaldo Cruz Institute, FIOCRUZ	Paola Resende et al
EPI_ISL_1415399	Reference Laboratory of the Ministry of Health	Laboratory of Respiratory Viruses and Measles, Oswaldo Cruz Institute, FIOCRUZ	Paola Resende et al

EPI_ISL_1415392	Reference Laboratory of the Ministry of Health	Laboratory of Respiratory Viruses and Measles, Oswaldo Cruz Institute, FIOCRUZ	Paola Resende et al
EPI_ISL_837555	Centro Nacional de Enfermedades Tropicales (CENETROP)	Laboratory of Respiratory Viruses and Measles, Oswaldo Cruz Institute, FIOCRUZ	Paola Resende et al
EPI_ISL_837556	Centro Nacional de Enfermedades Tropicales (CENETROP)	Laboratory of Respiratory Viruses and Measles, Oswaldo Cruz Institute, FIOCRUZ	Paola Resende et al
EPI_ISL_837557	Centro Nacional de Enfermedades Tropicales (CENETROP)	Laboratory of Respiratory Viruses and Measles, Oswaldo Cruz Institute, FIOCRUZ	Paola Resende et al
EPI_ISL_837558	Centro Nacional de Enfermedades Tropicales (CENETROP)	Laboratory of Respiratory Viruses and Measles, Oswaldo Cruz Institute, FIOCRUZ	Paola Resende et al
EPI_ISL_837554	Centro Nacional de Enfermedades Tropicales (CENETROP)	Laboratory of Respiratory Viruses and Measles, Oswaldo Cruz Institute, FIOCRUZ	Paola Resende et al
EPI_ISL_837559	Centro Nacional de Enfermedades Tropicales (CENETROP)	Laboratory of Respiratory Viruses and Measles, Oswaldo Cruz Institute, FIOCRUZ	Paola Resende et al
EPI_ISL_837560	Centro Nacional de Enfermedades Tropicales (CENETROP)	Laboratory of Respiratory Viruses and Measles, Oswaldo Cruz Institute, FIOCRUZ	Paola Resende et al
EPI_ISL_837561	Centro Nacional de Enfermedades Tropicales (CENETROP)	Laboratory of Respiratory Viruses and Measles, Oswaldo Cruz Institute, FIOCRUZ	Paola Resende et al
EPI_ISL_837562	Centro Nacional de Enfermedades Tropicales (CENETROP)	Laboratory of Respiratory Viruses and Measles, Oswaldo Cruz Institute, FIOCRUZ	Paola Resende et al
EPI_ISL_837563	Centro Nacional de Enfermedades Tropicales (CENETROP)	Laboratory of Respiratory Viruses and Measles, Oswaldo Cruz Institute, FIOCRUZ	Paola Resende et al
EPI_ISL_837564	Centro Nacional de Enfermedades Tropicales (CENETROP)	Laboratory of Respiratory Viruses and Measles, Oswaldo Cruz Institute, FIOCRUZ	Paola Resende et al
EPI_ISL_837565	Centro Nacional de Enfermedades Tropicales (CENETROP)	Laboratory of Respiratory Viruses and Measles, Oswaldo Cruz Institute, FIOCRUZ	Paola Resende et al
EPI_ISL_837553	Centro Nacional de Enfermedades Tropicales (CENETROP)	Laboratory of Respiratory Viruses and Measles, Oswaldo Cruz Institute, FIOCRUZ	Paola Resende et al
EPI_ISL_837568	Centro Nacional de Enfermedades Tropicales (CENETROP)	Laboratory of Respiratory Viruses and Measles, Oswaldo Cruz Institute, FIOCRUZ	Paola Resende et al
EPI_ISL_837569	Centro Nacional de Enfermedades Tropicales (CENETROP)	Laboratory of Respiratory Viruses and Measles, Oswaldo Cruz Institute, FIOCRUZ	Paola Resende et al
EPI_ISL_837570	Centro Nacional de Enfermedades Tropicales (CENETROP)	Laboratory of Respiratory Viruses and Measles, Oswaldo Cruz Institute, FIOCRUZ	Paola Resende et al
EPI_ISL_837571	Centro Nacional de Enfermedades Tropicales (CENETROP)	Laboratory of Respiratory Viruses and Measles, Oswaldo Cruz Institute, FIOCRUZ	Paola Resende et al
EPI_ISL_837550	Centro Nacional de Enfermedades Tropicales (CENETROP)	Laboratory of Respiratory Viruses and Measles, Oswaldo Cruz Institute, FIOCRUZ	Paola Resende et al
EPI_ISL_837572	Centro Nacional de Enfermedades Tropicales (CENETROP)	Laboratory of Respiratory Viruses and Measles, Oswaldo Cruz Institute, FIOCRUZ	Paola Resende et al
EPI_ISL_837573	Centro Nacional de Enfermedades Tropicales (CENETROP)	Laboratory of Respiratory Viruses and Measles, Oswaldo Cruz Institute, FIOCRUZ	Paola Resende et al
EPI_ISL_837575	Centro Nacional de Enfermedades Tropicales (CENETROP)	Laboratory of Respiratory Viruses and Measles, Oswaldo Cruz Institute, FIOCRUZ	Paola Resende et al

EPI_ISL_837576	Centro Nacional de Enfermedades Tropicales (CENETROP)	Laboratory of Respiratory Viruses and Measles, Oswaldo Cruz Institute, FIOCRUZ	Paola Resende et al
EPI_ISL_837577	Centro Nacional de Enfermedades Tropicales (CENETROP)	Laboratory of Respiratory Viruses and Measles, Oswaldo Cruz Institute, FIOCRUZ	Paola Resende et al
EPI_ISL_837578	Centro Nacional de Enfermedades Tropicales (CENETROP)	Laboratory of Respiratory Viruses and Measles, Oswaldo Cruz Institute, FIOCRUZ	Paola Resende et al
EPI_ISL_792639	Laboratório Central de Saúde Pública do Estado de Alagoas (LACEN-AL)	Laboratory of Respiratory Viruses and Measles, Oswaldo Cruz Institute, FIOCRUZ	Paola Resende et al
EPI_ISL_792640	Laboratório Central de Saúde Pública do Estado de Alagoas (LACEN-AL)	Laboratory of Respiratory Viruses and Measles, Oswaldo Cruz Institute, FIOCRUZ	Paola Resende et al
EPI_ISL_792641	Laboratório Central de Saúde Pública do Estado de Alagoas (LACEN-AL)	Laboratory of Respiratory Viruses and Measles, Oswaldo Cruz Institute, FIOCRUZ	Paola Resende et al
EPI_ISL_792642	Laboratório Central de Saúde Pública do Estado de Alagoas (LACEN-AL)	Laboratory of Respiratory Viruses and Measles, Oswaldo Cruz Institute, FIOCRUZ	Paola Resende et al
EPI_ISL_792643	Laboratório Central de Saúde Pública do Estado de Alagoas (LACEN-AL)	Laboratory of Respiratory Viruses and Measles, Oswaldo Cruz Institute, FIOCRUZ	Paola Resende et al
EPI_ISL_792644	Laboratório Central de Saúde Pública do Estado de Alagoas (LACEN-AL)	Laboratory of Respiratory Viruses and Measles, Oswaldo Cruz Institute, FIOCRUZ	Paola Resende et al
EPI_ISL_1181592	Laboratorio Central de Saude Publica do Estado do Alagoas (LACEN-AL)	Laboratory of Respiratory Viruses and Measles, Oswaldo Cruz Institute, FIOCRUZ	Paola Resende et al
EPI_ISL_1181396	Laboratorio Central de Saude Publica do Estado do Alagoas (LACEN-AL)	Laboratory of Respiratory Viruses and Measles, Oswaldo Cruz Institute, FIOCRUZ	Paola Resende et al
EPI_ISL_1181365	Laboratorio Central de Saude Publica do Estado do Alagoas (LACEN-AL)	Laboratory of Respiratory Viruses and Measles, Oswaldo Cruz Institute, FIOCRUZ	Paola Resende et al
EPI_ISL_1219135	Laboratorio Central de Saude Publica do Estado do Alagoas (LACEN-AL)	Laboratory of Respiratory Viruses and Measles, Oswaldo Cruz Institute, FIOCRUZ	Paola Resende et al
EPI_ISL_1219134	Laboratorio Central de Saude Publica do Estado do Alagoas (LACEN-AL)	Laboratory of Respiratory Viruses and Measles, Oswaldo Cruz Institute, FIOCRUZ	Paola Resende et al
EPI_ISL_427292	Laboratório Central de Saúde Pública do Estado de Alagoas (LACEN-AL)	Laboratory of Respiratory Viruses and Measles, Oswaldo Cruz Institute, FIOCRUZ	Paola Resende et al
EPI_ISL_1181354	Gonçalo Moniz Institute, FIOCRUZ, Bahia	Laboratory of Respiratory Viruses and Measles, Oswaldo Cruz Institute, FIOCRUZ	Paola Resende et al
EPI_ISL_1181484	Gonçalo Moniz Institute, FIOCRUZ, Bahia	Laboratory of Respiratory Viruses and Measles, Oswaldo Cruz Institute, FIOCRUZ	Paola Resende et al
EPI_ISL_1181485	Gonçalo Moniz Institute, FIOCRUZ, Bahia	Laboratory of Respiratory Viruses and Measles, Oswaldo Cruz Institute, FIOCRUZ	Paola Resende et al
EPI_ISL_1181435	Federal University of Southern Bahia (UFSB - Universidade Federal do Sul da Bahia)	Laboratory of Respiratory Viruses and Measles, Oswaldo Cruz Institute, FIOCRUZ	Paola Resende et al
EPI_ISL_1181436	Federal University of Southern Bahia (UFSB - Universidade Federal do Sul da Bahia)	Laboratory of Respiratory Viruses and Measles, Oswaldo Cruz Institute, FIOCRUZ	Paola Resende et al
EPI_ISL_1181364	Federal University of Southern Bahia (UFSB - Universidade Federal do Sul da Bahia)	Laboratory of Respiratory Viruses and Measles, Oswaldo Cruz Institute, FIOCRUZ	Paola Resende et al
EPI_ISL_1181443	Federal University of Southern Bahia (UFSB - Universidade Federal do Sul da Bahia)	Laboratory of Respiratory Viruses and Measles, Oswaldo Cruz Institute, FIOCRUZ	Paola Resende et al

EPI_ISL_1181450	Federal University of Southern Bahia (UFSB - Universidade Federal do Sul da Bahia)	Laboratory of Respiratory Viruses and Measles, Oswaldo Cruz Institute, FIOCRUZ	Paola Resende et al
EPI_ISL_1181449	Gonçalo Moniz Institute, FIOCRUZ, Bahia	Laboratory of Respiratory Viruses and Measles, Oswaldo Cruz Institute, FIOCRUZ	Paola Resende et al
EPI_ISL_1181444	Gonçalo Moniz Institute, FIOCRUZ, Bahia	Laboratory of Respiratory Viruses and Measles, Oswaldo Cruz Institute, FIOCRUZ	Paola Resende et al
EPI_ISL_1181445	Gonçalo Moniz Institute, FIOCRUZ, Bahia	Laboratory of Respiratory Viruses and Measles, Oswaldo Cruz Institute, FIOCRUZ	Paola Resende et al
EPI_ISL_1181441	Gonçalo Moniz Institute, FIOCRUZ, Bahia	Laboratory of Respiratory Viruses and Measles, Oswaldo Cruz Institute, FIOCRUZ	Paola Resende et al
EPI_ISL_1181440	Gonçalo Moniz Institute, FIOCRUZ, Bahia	Laboratory of Respiratory Viruses and Measles, Oswaldo Cruz Institute, FIOCRUZ	Paola Resende et al
EPI_ISL_1181355	Gonçalo Moniz Institute, FIOCRUZ, Bahia	Laboratory of Respiratory Viruses and Measles, Oswaldo Cruz Institute, FIOCRUZ	Paola Resende et al
EPI_ISL_1181429	Gonçalo Moniz Institute, FIOCRUZ, Bahia	Laboratory of Respiratory Viruses and Measles, Oswaldo Cruz Institute, FIOCRUZ	Paola Resende et al
EPI_ISL_1181379	Gonçalo Moniz Institute, FIOCRUZ, Bahia	Laboratory of Respiratory Viruses and Measles, Oswaldo Cruz Institute, FIOCRUZ	Paola Resende et al
EPI_ISL_1181424	Gonçalo Moniz Institute, FIOCRUZ, Bahia	Laboratory of Respiratory Viruses and Measles, Oswaldo Cruz Institute, FIOCRUZ	Paola Resende et al
EPI_ISL_1181419	Gonçalo Moniz Institute, FIOCRUZ, Bahia	Laboratory of Respiratory Viruses and Measles, Oswaldo Cruz Institute, FIOCRUZ	Paola Resende et al
EPI_ISL_1181408	Gonçalo Moniz Institute, FIOCRUZ, Bahia	Laboratory of Respiratory Viruses and Measles, Oswaldo Cruz Institute, FIOCRUZ	Paola Resende et al
EPI_ISL_1219136	Gonçalo Moniz Institute, FIOCRUZ, Bahia	Laboratory of Respiratory Viruses and Measles, Oswaldo Cruz Institute, FIOCRUZ	Paola Resende et al
EPI_ISL_1181356	Oswaldo Cruz Foundation, FIOCRUZ - Ceara (Fiocruz-CE)	Laboratory of Respiratory Viruses and Measles, Oswaldo Cruz Institute, FIOCRUZ	Paola Resende et al
EPI_ISL_1181520	Oswaldo Cruz Foundation, FIOCRUZ - Ceara (Fiocruz-CE)	Laboratory of Respiratory Viruses and Measles, Oswaldo Cruz Institute, FIOCRUZ	Paola Resende et al
EPI_ISL_1181522	Oswaldo Cruz Foundation, FIOCRUZ - Ceara (Fiocruz-CE)	Laboratory of Respiratory Viruses and Measles, Oswaldo Cruz Institute, FIOCRUZ	Paola Resende et al
EPI_ISL_1181575	Oswaldo Cruz Foundation, FIOCRUZ - Ceara (Fiocruz-CE)	Laboratory of Respiratory Viruses and Measles, Oswaldo Cruz Institute, FIOCRUZ	Paola Resende et al
EPI_ISL_1181577	Oswaldo Cruz Foundation, FIOCRUZ - Ceara (Fiocruz-CE)	Laboratory of Respiratory Viruses and Measles, Oswaldo Cruz Institute, FIOCRUZ	Paola Resende et al
EPI_ISL_1181586	Oswaldo Cruz Foundation, FIOCRUZ - Ceara (Fiocruz-CE)	Laboratory of Respiratory Viruses and Measles, Oswaldo Cruz Institute, FIOCRUZ	Paola Resende et al
EPI_ISL_1181588	Oswaldo Cruz Foundation, FIOCRUZ - Ceara (Fiocruz-CE)	Laboratory of Respiratory Viruses and Measles, Oswaldo Cruz Institute, FIOCRUZ	Paola Resende et al
EPI_ISL_1181591	Oswaldo Cruz Foundation, FIOCRUZ - Ceara (Fiocruz-CE)	Laboratory of Respiratory Viruses and Measles, Oswaldo Cruz Institute, FIOCRUZ	Paola Resende et al
EPI_ISL_1181593	Oswaldo Cruz Foundation, FIOCRUZ - Ceara (Fiocruz-CE)	Laboratory of Respiratory Viruses and Measles, Oswaldo Cruz Institute, FIOCRUZ	Paola Resende et al

EPI_ISL_1181596	Oswaldo Cruz Foundation, FIOCRUZ - Ceara (Fiocruz-CE)	Laboratory of Respiratory Viruses and Measles, Oswaldo Cruz Institute, FIOCRUZ	Paola Resende et al
EPI_ISL_1181598	Oswaldo Cruz Foundation, FIOCRUZ - Ceara (Fiocruz-CE)	Laboratory of Respiratory Viruses and Measles, Oswaldo Cruz Institute, FIOCRUZ	Paola Resende et al
EPI_ISL_1181604	Oswaldo Cruz Foundation, FIOCRUZ - Ceara (Fiocruz-CE)	Laboratory of Respiratory Viruses and Measles, Oswaldo Cruz Institute, FIOCRUZ	Paola Resende et al
EPI_ISL_1181621	Oswaldo Cruz Foundation, FIOCRUZ - Ceara (Fiocruz-CE)	Laboratory of Respiratory Viruses and Measles, Oswaldo Cruz Institute, FIOCRUZ	Paola Resende et al
EPI_ISL_1181622	Oswaldo Cruz Foundation, FIOCRUZ - Ceara (Fiocruz-CE)	Laboratory of Respiratory Viruses and Measles, Oswaldo Cruz Institute, FIOCRUZ	Paola Resende et al
EPI_ISL_427294	Laboratory of Respiratory Viruses and Measles, Oswaldo Cruz Institute, FIOCRUZ	Laboratory of Respiratory Viruses and Measles, Oswaldo Cruz Institute, FIOCRUZ	Paola Resende et al
EPI_ISL_427295	Laboratory of Respiratory Viruses and Measles, Oswaldo Cruz Institute, FIOCRUZ	Laboratory of Respiratory Viruses and Measles, Oswaldo Cruz Institute, FIOCRUZ	Paola Resende et al
EPI_ISL_427296	Laboratory of Respiratory Viruses and Measles, Oswaldo Cruz Institute, FIOCRUZ	Laboratory of Respiratory Viruses and Measles, Oswaldo Cruz Institute, FIOCRUZ	Paola Resende et al
EPI_ISL_427297	Laboratory of Respiratory Viruses and Measles, Oswaldo Cruz Institute, FIOCRUZ	Laboratory of Respiratory Viruses and Measles, Oswaldo Cruz Institute, FIOCRUZ	Paola Resende et al
EPI_ISL_427298	Laboratory of Respiratory Viruses and Measles, Oswaldo Cruz Institute, FIOCRUZ	Laboratory of Respiratory Viruses and Measles, Oswaldo Cruz Institute, FIOCRUZ	Paola Resende et al
EPI_ISL_415128	Laboratório Central de Saúde Pública do Estado do Espírito Santo (LACEN-ES)	Laboratory of Respiratory Viruses and Measles, Oswaldo Cruz Institute, FIOCRUZ	Paola Resende et al
EPI_ISL_1181432	Laboratorio Central de Saude Publica do Estado do Espitito Santo (LACEN-ES)	Laboratory of Respiratory Viruses and Measles, Oswaldo Cruz Institute, FIOCRUZ	Paola Resende et al
EPI_ISL_1181418	Laboratorio Central de Saude Publica do Estado do Espitito Santo (LACEN-ES)	Laboratory of Respiratory Viruses and Measles, Oswaldo Cruz Institute, FIOCRUZ	Paola Resende et al
EPI_ISL_1181420	Laboratorio Central de Saude Publica do Estado do Espitito Santo (LACEN-ES)	Laboratory of Respiratory Viruses and Measles, Oswaldo Cruz Institute, FIOCRUZ	Paola Resende et al
EPI_ISL_1181421	Laboratorio Central de Saude Publica do Estado do Espitito Santo (LACEN-ES)	Laboratory of Respiratory Viruses and Measles, Oswaldo Cruz Institute, FIOCRUZ	Paola Resende et al
EPI_ISL_1181423	Laboratorio Central de Saude Publica do Estado do Espitito Santo (LACEN-ES)	Laboratory of Respiratory Viruses and Measles, Oswaldo Cruz Institute, FIOCRUZ	Paola Resende et al
EPI_ISL_1181397	Laboratorio Central de Saude Publica do Estado do Espitito Santo (LACEN-ES)	Laboratory of Respiratory Viruses and Measles, Oswaldo Cruz Institute, FIOCRUZ	Paola Resende et al
EPI_ISL_1181395	Laboratorio Central de Saude Publica do Estado do Espitito Santo (LACEN-ES)	Laboratory of Respiratory Viruses and Measles, Oswaldo Cruz Institute, FIOCRUZ	Paola Resende et al
EPI_ISL_1181583	Laboratorio Central de Saude Publica do Estado do Espitito Santo (LACEN-ES)	Laboratory of Respiratory Viruses and Measles, Oswaldo Cruz Institute, FIOCRUZ	Paola Resende et al
EPI_ISL_1181585	Laboratorio Central de Saude Publica do Estado do Espitito Santo (LACEN-ES)	Laboratory of Respiratory Viruses and Measles, Oswaldo Cruz Institute, FIOCRUZ	Paola Resende et al
EPI_ISL_1181582	Laboratorio Central de Saude Publica do Estado do Espitito Santo (LACEN-ES)	Laboratory of Respiratory Viruses and Measles, Oswaldo Cruz Institute, FIOCRUZ	Paola Resende et al
EPI_ISL_1181480	Laboratorio de Imunologia de Transplantes de Goias LTDA (HLAGYN)	Laboratory of Respiratory Viruses and Measles, Oswaldo Cruz Institute, FIOCRUZ	Paola Resende et al

EPI_ISL_1181478	Laboratorio de Imunologia de Transplantes de Goias LTDA (HLAGYN)	Laboratory of Respiratory Viruses and Measles, Oswaldo Cruz Institute, FIOCRUZ	Paola Resende et al
EPI_ISL_1181479	Laboratorio de Imunologia de Transplantes de Goias LTDA (HLAGYN)	Laboratory of Respiratory Viruses and Measles, Oswaldo Cruz Institute, FIOCRUZ	Paola Resende et al
EPI_ISL_1181546	Laboratorio de Imunologia de Transplantes de Goias LTDA (HLAGYN)	Laboratory of Respiratory Viruses and Measles, Oswaldo Cruz Institute, FIOCRUZ	Paola Resende et al
EPI_ISL_1181547	Laboratorio de Imunologia de Transplantes de Goias LTDA (HLAGYN)	Laboratory of Respiratory Viruses and Measles, Oswaldo Cruz Institute, FIOCRUZ	Paola Resende et al
EPI_ISL_1181548	Laboratorio de Imunologia de Transplantes de Goias LTDA (HLAGYN)	Laboratory of Respiratory Viruses and Measles, Oswaldo Cruz Institute, FIOCRUZ	Paola Resende et al
EPI_ISL_1181549	Laboratorio de Imunologia de Transplantes de Goias LTDA (HLAGYN)	Laboratory of Respiratory Viruses and Measles, Oswaldo Cruz Institute, FIOCRUZ	Paola Resende et al
EPI_ISL_1181550	Laboratorio de Imunologia de Transplantes de Goias LTDA (HLAGYN)	Laboratory of Respiratory Viruses and Measles, Oswaldo Cruz Institute, FIOCRUZ	Paola Resende et al
EPI_ISL_1181471	Laboratorio de Imunologia de Transplantes de Goias LTDA (HLAGYN)	Laboratory of Respiratory Viruses and Measles, Oswaldo Cruz Institute, FIOCRUZ	Paola Resende et al
EPI_ISL_1181472	Laboratorio de Imunologia de Transplantes de Goias LTDA (HLAGYN)	Laboratory of Respiratory Viruses and Measles, Oswaldo Cruz Institute, FIOCRUZ	Paola Resende et al
EPI_ISL_1181473	Laboratorio de Imunologia de Transplantes de Goias LTDA (HLAGYN)	Laboratory of Respiratory Viruses and Measles, Oswaldo Cruz Institute, FIOCRUZ	Paola Resende et al
EPI_ISL_1181487	Laboratorio de Imunologia de Transplantes de Goias LTDA (HLAGYN)	Laboratory of Respiratory Viruses and Measles, Oswaldo Cruz Institute, FIOCRUZ	Paola Resende et al
EPI_ISL_1181492	Laboratorio de Imunologia de Transplantes de Goias LTDA (HLAGYN)	Laboratory of Respiratory Viruses and Measles, Oswaldo Cruz Institute, FIOCRUZ	Paola Resende et al
EPI_ISL_1181525	Laboratorio de Imunologia de Transplantes de Goias LTDA (HLAGYN)	Laboratory of Respiratory Viruses and Measles, Oswaldo Cruz Institute, FIOCRUZ	Paola Resende et al
EPI_ISL_1181535	Laboratorio de Imunologia de Transplantes de Goias LTDA (HLAGYN)	Laboratory of Respiratory Viruses and Measles, Oswaldo Cruz Institute, FIOCRUZ	Paola Resende et al
EPI_ISL_1181532	Laboratorio de Imunologia de Transplantes de Goias LTDA (HLAGYN)	Laboratory of Respiratory Viruses and Measles, Oswaldo Cruz Institute, FIOCRUZ	Paola Resende et al
EPI_ISL_1181516	Laboratorio de Imunologia de Transplantes de Goias LTDA (HLAGYN)	Laboratory of Respiratory Viruses and Measles, Oswaldo Cruz Institute, FIOCRUZ	Paola Resende et al
EPI_ISL_1181569	Laboratorio de Imunologia de Transplantes de Goias LTDA (HLAGYN)	Laboratory of Respiratory Viruses and Measles, Oswaldo Cruz Institute, FIOCRUZ	Paola Resende et al
EPI_ISL_1181452	Laboratorio de Imunologia de Transplantes de Goias LTDA (HLAGYN)	Laboratory of Respiratory Viruses and Measles, Oswaldo Cruz Institute, FIOCRUZ	Paola Resende et al
EPI_ISL_1181378	Laboratorio de Imunologia de Transplantes de Goias LTDA (HLAGYN)	Laboratory of Respiratory Viruses and Measles, Oswaldo Cruz Institute, FIOCRUZ	Paola Resende et al
EPI_ISL_1465194	Laboratorio Central de Saude Publica do Estado do Maranhao (LACEN-MA)	Laboratory of Respiratory Viruses and Measles, Oswaldo Cruz Institute, FIOCRUZ	Paola Resende et al
EPI_ISL_1465217	Laboratorio Central de Saude Publica do Estado do Maranhao (LACEN-MA)	Laboratory of Respiratory Viruses and Measles, Oswaldo Cruz Institute, FIOCRUZ	Paola Resende et al
EPI_ISL_1465215	Laboratorio Central de Saude Publica do Estado do Maranhao (LACEN-MA)	Laboratory of Respiratory Viruses and Measles, Oswaldo Cruz Institute, FIOCRUZ	Paola Resende et al

EPI_ISL_1465203	Laboratorio Central de Saude Publica do Estado do Maranhao (LACEN-MA)	Laboratory of Respiratory Viruses and Measles, Oswaldo Cruz Institute, FIOCRUZ	Paola Resende et al
EPI_ISL_1465206	Laboratorio Central de Saude Publica do Estado do Maranhao (LACEN-MA)	Laboratory of Respiratory Viruses and Measles, Oswaldo Cruz Institute, FIOCRUZ	Paola Resende et al
EPI_ISL_1465213	Laboratorio Central de Saude Publica do Estado do Maranhao (LACEN-MA)	Laboratory of Respiratory Viruses and Measles, Oswaldo Cruz Institute, FIOCRUZ	Paola Resende et al
EPI_ISL_1465212	Laboratorio Central de Saude Publica do Estado do Maranhao (LACEN-MA)	Laboratory of Respiratory Viruses and Measles, Oswaldo Cruz Institute, FIOCRUZ	Paola Resende et al
EPI_ISL_1465208	Laboratorio Central de Saude Publica do Estado do Maranhao (LACEN-MA)	Laboratory of Respiratory Viruses and Measles, Oswaldo Cruz Institute, FIOCRUZ	Paola Resende et al
EPI_ISL_1465209	Laboratorio Central de Saude Publica do Estado do Maranhao (LACEN-MA)	Laboratory of Respiratory Viruses and Measles, Oswaldo Cruz Institute, FIOCRUZ	Paola Resende et al
EPI_ISL_1465210	Laboratorio Central de Saude Publica do Estado do Maranhao (LACEN-MA)	Laboratory of Respiratory Viruses and Measles, Oswaldo Cruz Institute, FIOCRUZ	Paola Resende et al
EPI_ISL_1465199	Laboratorio Central de Saude Publica do Estado do Maranhao (LACEN-MA)	Laboratory of Respiratory Viruses and Measles, Oswaldo Cruz Institute, FIOCRUZ	Paola Resende et al
EPI_ISL_1465201	Laboratorio Central de Saude Publica do Estado do Maranhao (LACEN-MA)	Laboratory of Respiratory Viruses and Measles, Oswaldo Cruz Institute, FIOCRUZ	Paola Resende et al
EPI_ISL_1465202	Laboratorio Central de Saude Publica do Estado do Maranhao (LACEN-MA)	Laboratory of Respiratory Viruses and Measles, Oswaldo Cruz Institute, FIOCRUZ	Paola Resende et al
EPI_ISL_1465192	Laboratorio Central de Saude Publica do Estado do Maranhao (LACEN-MA)	Laboratory of Respiratory Viruses and Measles, Oswaldo Cruz Institute, FIOCRUZ	Paola Resende et al
EPI_ISL_1465189	Laboratorio Central de Saude Publica do Estado do Maranhao (LACEN-MA)	Laboratory of Respiratory Viruses and Measles, Oswaldo Cruz Institute, FIOCRUZ	Paola Resende et al
EPI_ISL_1465225	Laboratorio Central de Saude Publica do Estado do Maranhao (LACEN-MA)	Laboratory of Respiratory Viruses and Measles, Oswaldo Cruz Institute, FIOCRUZ	Paola Resende et al
EPI_ISL_1465195	Laboratorio Central de Saude Publica do Estado do Maranhao (LACEN-MA)	Laboratory of Respiratory Viruses and Measles, Oswaldo Cruz Institute, FIOCRUZ	Paola Resende et al
EPI_ISL_1465205	Laboratorio Central de Saude Publica do Estado do Maranhao (LACEN-MA)	Laboratory of Respiratory Viruses and Measles, Oswaldo Cruz Institute, FIOCRUZ	Paola Resende et al
EPI_ISL_1465196	Laboratorio Central de Saude Publica do Estado do Maranhao (LACEN-MA)	Laboratory of Respiratory Viruses and Measles, Oswaldo Cruz Institute, FIOCRUZ	Paola Resende et al
EPI_ISL_1465191	Laboratorio Central de Saude Publica do Estado do Maranhao (LACEN-MA)	Laboratory of Respiratory Viruses and Measles, Oswaldo Cruz Institute, FIOCRUZ	Paola Resende et al
EPI_ISL_1465216	Laboratorio Central de Saude Publica do Estado do Maranhao (LACEN-MA)	Laboratory of Respiratory Viruses and Measles, Oswaldo Cruz Institute, FIOCRUZ	Paola Resende et al
EPI_ISL_1465221	Laboratorio Central de Saude Publica do Estado do Maranhao (LACEN-MA)	Laboratory of Respiratory Viruses and Measles, Oswaldo Cruz Institute, FIOCRUZ	Paola Resende et al
EPI_ISL_1465219	Laboratorio Central de Saude Publica do Estado do Maranhao (LACEN-MA)	Laboratory of Respiratory Viruses and Measles, Oswaldo Cruz Institute, FIOCRUZ	Paola Resende et al
EPI_ISL_1465220	Laboratorio Central de Saude Publica do Estado do Maranhao (LACEN-MA)	Laboratory of Respiratory Viruses and Measles, Oswaldo Cruz Institute, FIOCRUZ	Paola Resende et al
EPI_ISL_1465261	Laboratorio Central de Saude Publica do Estado do Maranhao (LACEN-MA)	Laboratory of Respiratory Viruses and Measles, Oswaldo Cruz Institute, FIOCRUZ	Paola Resende et al

EPI_ISL_1465246	Laboratorio Central de Saude Publica do Estado do Maranhao (LACEN-MA)	Laboratory of Respiratory Viruses and Measles, Oswaldo Cruz Institute, FIOCRUZ	Paola Resende et al
EPI_ISL_1465275	Laboratorio Central de Saude Publica do Estado do Maranhao (LACEN-MA)	Laboratory of Respiratory Viruses and Measles, Oswaldo Cruz Institute, FIOCRUZ	Paola Resende et al
EPI_ISL_1465277	Laboratorio Central de Saude Publica do Estado do Maranhao (LACEN-MA)	Laboratory of Respiratory Viruses and Measles, Oswaldo Cruz Institute, FIOCRUZ	Paola Resende et al
EPI_ISL_1465271	Laboratorio Central de Saude Publica do Estado do Maranhao (LACEN-MA)	Laboratory of Respiratory Viruses and Measles, Oswaldo Cruz Institute, FIOCRUZ	Paola Resende et al
EPI_ISL_1465222	Laboratorio Central de Saude Publica do Estado do Maranhao (LACEN-MA)	Laboratory of Respiratory Viruses and Measles, Oswaldo Cruz Institute, FIOCRUZ	Paola Resende et al
EPI_ISL_1465268	Laboratorio Central de Saude Publica do Estado do Maranhao (LACEN-MA)	Laboratory of Respiratory Viruses and Measles, Oswaldo Cruz Institute, FIOCRUZ	Paola Resende et al
EPI_ISL_1465262	Laboratorio Central de Saude Publica do Estado do Maranhao (LACEN-MA)	Laboratory of Respiratory Viruses and Measles, Oswaldo Cruz Institute, FIOCRUZ	Paola Resende et al
EPI_ISL_1465267	Laboratorio Central de Saude Publica do Estado do Maranhao (LACEN-MA)	Laboratory of Respiratory Viruses and Measles, Oswaldo Cruz Institute, FIOCRUZ	Paola Resende et al
EPI_ISL_1465270	Laboratorio Central de Saude Publica do Estado do Maranhao (LACEN-MA)	Laboratory of Respiratory Viruses and Measles, Oswaldo Cruz Institute, FIOCRUZ	Paola Resende et al
EPI_ISL_1465278	Laboratorio Central de Saude Publica do Estado do Maranhao (LACEN-MA)	Laboratory of Respiratory Viruses and Measles, Oswaldo Cruz Institute, FIOCRUZ	Paola Resende et al
EPI_ISL_1181414	Laboratorio Central de Saude Publica do Estado Maranhao (LACEN-MA)	Laboratory of Respiratory Viruses and Measles, Oswaldo Cruz Institute, FIOCRUZ	Paola Resende et al
EPI_ISL_1181400	Laboratorio Central de Saude Publica do Estado Maranhao (LACEN-MA)	Laboratory of Respiratory Viruses and Measles, Oswaldo Cruz Institute, FIOCRUZ	Paola Resende et al
EPI_ISL_1181426	Laboratorio Central de Saude Publica do Estado de Minas Gerais (LACEN-MG)	Laboratory of Respiratory Viruses and Measles, Oswaldo Cruz Institute, FIOCRUZ	Paola Resende et al
EPI_ISL_1181427	Laboratorio Central de Saude Publica do Estado de Minas Gerais (LACEN-MG)	Laboratory of Respiratory Viruses and Measles, Oswaldo Cruz Institute, FIOCRUZ	Paola Resende et al
EPI_ISL_1181428	Laboratorio Central de Saude Publica do Estado de Minas Gerais (LACEN-MG)	Laboratory of Respiratory Viruses and Measles, Oswaldo Cruz Institute, FIOCRUZ	Paola Resende et al
EPI_ISL_1181399	Laboratorio Central de Saude Publica do Estado de Minas Gerais (LACEN-MG)	Laboratory of Respiratory Viruses and Measles, Oswaldo Cruz Institute, FIOCRUZ	Paola Resende et al
EPI_ISL_1219137	Laboratorio Central de Saude Publica do Estado de Minas Gerais (LACEN-MG)	Laboratory of Respiratory Viruses and Measles, Oswaldo Cruz Institute, FIOCRUZ	Paola Resende et al
EPI_ISL_1181469	Federal University of Mato Grosso (UFMT)	Laboratory of Respiratory Viruses and Measles, Oswaldo Cruz Institute, FIOCRUZ	Paola Resende et al
EPI_ISL_1219138	Federal University of Mato Grosso (UFMT)	Laboratory of Respiratory Viruses and Measles, Oswaldo Cruz Institute, FIOCRUZ	Paola Resende et al
EPI_ISL_792563	Laboratório Central de Saúde Pública do Estado da Paraíba (LACEN-PB)	Laboratory of Respiratory Viruses and Measles, Oswaldo Cruz Institute, FIOCRUZ	Paola Resende et al
EPI_ISL_792564	Laboratório Central de Saúde Pública do Estado da Paraíba (LACEN-PB)	Laboratory of Respiratory Viruses and Measles, Oswaldo Cruz Institute, FIOCRUZ	Paola Resende et al
EPI_ISL_792565	Laboratório Central de Saúde Pública do Estado da Paraíba (LACEN-PB)	Laboratory of Respiratory Viruses and Measles, Oswaldo Cruz Institute, FIOCRUZ	Paola Resende et al

EPI_ISL_792566	Laboratório Central de Saúde Pública do Estado da Paraíba (LACEN-PB)	Laboratory of Respiratory Viruses and Measles, Oswaldo Cruz Institute, FIOCRUZ	Paola Resende et al
EPI_ISL_792568	Laboratório Central de Saúde Pública do Estado da Paraíba (LACEN-PB)	Laboratory of Respiratory Viruses and Measles, Oswaldo Cruz Institute, FIOCRUZ	Paola Resende et al
EPI_ISL_792569	Laboratório Central de Saúde Pública do Estado da Paraíba (LACEN-PB)	Laboratory of Respiratory Viruses and Measles, Oswaldo Cruz Institute, FIOCRUZ	Paola Resende et al
EPI_ISL_792570	Laboratório Central de Saúde Pública do Estado da Paraíba (LACEN-PB)	Laboratory of Respiratory Viruses and Measles, Oswaldo Cruz Institute, FIOCRUZ	Paola Resende et al
EPI_ISL_792571	Laboratório Central de Saúde Pública do Estado da Paraíba (LACEN-PB)	Laboratory of Respiratory Viruses and Measles, Oswaldo Cruz Institute, FIOCRUZ	Paola Resende et al
EPI_ISL_792572	Laboratório Central de Saúde Pública do Estado da Paraíba (LACEN-PB)	Laboratory of Respiratory Viruses and Measles, Oswaldo Cruz Institute, FIOCRUZ	Paola Resende et al
EPI_ISL_792573	Laboratório Central de Saúde Pública do Estado da Paraíba (LACEN-PB)	Laboratory of Respiratory Viruses and Measles, Oswaldo Cruz Institute, FIOCRUZ	Paola Resende et al
EPI_ISL_792574	Laboratório Central de Saúde Pública do Estado da Paraíba (LACEN-PB)	Laboratory of Respiratory Viruses and Measles, Oswaldo Cruz Institute, FIOCRUZ	Paola Resende et al
EPI_ISL_792575	Laboratório Central de Saúde Pública do Estado da Paraíba (LACEN-PB)	Laboratory of Respiratory Viruses and Measles, Oswaldo Cruz Institute, FIOCRUZ	Paola Resende et al
EPI_ISL_792576	Laboratório Central de Saúde Pública do Estado da Paraíba (LACEN-PB)	Laboratory of Respiratory Viruses and Measles, Oswaldo Cruz Institute, FIOCRUZ	Paola Resende et al
EPI_ISL_792577	Laboratório Central de Saúde Pública do Estado da Paraíba (LACEN-PB)	Laboratory of Respiratory Viruses and Measles, Oswaldo Cruz Institute, FIOCRUZ	Paola Resende et al
EPI_ISL_792578	Laboratório Central de Saúde Pública do Estado da Paraíba (LACEN-PB)	Laboratory of Respiratory Viruses and Measles, Oswaldo Cruz Institute, FIOCRUZ	Paola Resende et al
EPI_ISL_792579	Laboratório Central de Saúde Pública do Estado da Paraíba (LACEN-PB)	Laboratory of Respiratory Viruses and Measles, Oswaldo Cruz Institute, FIOCRUZ	Paola Resende et al
EPI_ISL_792583	Laboratório Central de Saúde Pública do Estado da Paraíba (LACEN-PB)	Laboratory of Respiratory Viruses and Measles, Oswaldo Cruz Institute, FIOCRUZ	Paola Resende et al
EPI_ISL_792585	Laboratório Central de Saúde Pública do Estado da Paraíba (LACEN-PB)	Laboratory of Respiratory Viruses and Measles, Oswaldo Cruz Institute, FIOCRUZ	Paola Resende et al
EPI_ISL_792587	Laboratório Central de Saúde Pública do Estado da Paraíba (LACEN-PB)	Laboratory of Respiratory Viruses and Measles, Oswaldo Cruz Institute, FIOCRUZ	Paola Resende et al
EPI_ISL_792588	Laboratório Central de Saúde Pública do Estado da Paraíba (LACEN-PB)	Laboratory of Respiratory Viruses and Measles, Oswaldo Cruz Institute, FIOCRUZ	Paola Resende et al
EPI_ISL_792589	Laboratório Central de Saúde Pública do Estado da Paraíba (LACEN-PB)	Laboratory of Respiratory Viruses and Measles, Oswaldo Cruz Institute, FIOCRUZ	Paola Resende et al
EPI_ISL_792590	Laboratório Central de Saúde Pública do Estado da Paraíba (LACEN-PB)	Laboratory of Respiratory Viruses and Measles, Oswaldo Cruz Institute, FIOCRUZ	Paola Resende et al
EPI_ISL_792593	Laboratório Central de Saúde Pública do Estado da Paraíba (LACEN-PB)	Laboratory of Respiratory Viruses and Measles, Oswaldo Cruz Institute, FIOCRUZ	Paola Resende et al
EPI_ISL_792594	Laboratório Central de Saúde Pública do Estado da Paraíba (LACEN-PB)	Laboratory of Respiratory Viruses and Measles, Oswaldo Cruz Institute, FIOCRUZ	Paola Resende et al
EPI_ISL_792595	Laboratório Central de Saúde Pública do Estado da Paraíba (LACEN-PB)	Laboratory of Respiratory Viruses and Measles, Oswaldo Cruz Institute, FIOCRUZ	Paola Resende et al

EPI_ISL_792596	Laboratório Central de Saúde Pública do Estado da Paraíba (LACEN-PB)	Laboratory of Respiratory Viruses and Measles, Oswaldo Cruz Institute, FIOCRUZ	Paola Resende et al
EPI_ISL_792597	Laboratório Central de Saúde Pública do Estado da Paraíba (LACEN-PB)	Laboratory of Respiratory Viruses and Measles, Oswaldo Cruz Institute, FIOCRUZ	Paola Resende et al
EPI_ISL_792598	Laboratório Central de Saúde Pública do Estado da Paraíba (LACEN-PB)	Laboratory of Respiratory Viruses and Measles, Oswaldo Cruz Institute, FIOCRUZ	Paola Resende et al
EPI_ISL_792599	Laboratório Central de Saúde Pública do Estado da Paraíba (LACEN-PB)	Laboratory of Respiratory Viruses and Measles, Oswaldo Cruz Institute, FIOCRUZ	Paola Resende et al
EPI_ISL_792561	Laboratório Central de Saúde Pública do Estado da Paraíba (LACEN-PB)	Laboratory of Respiratory Viruses and Measles, Oswaldo Cruz Institute, FIOCRUZ	Paola Resende et al
EPI_ISL_792562	Laboratório Central de Saúde Pública do Estado da Paraíba (LACEN-PB)	Laboratory of Respiratory Viruses and Measles, Oswaldo Cruz Institute, FIOCRUZ	Paola Resende et al
EPI_ISL_792600	Laboratório Central de Saúde Pública do Estado da Paraíba (LACEN-PB)	Laboratory of Respiratory Viruses and Measles, Oswaldo Cruz Institute, FIOCRUZ	Paola Resende et al
EPI_ISL_792601	Laboratório Central de Saúde Pública do Estado da Paraíba (LACEN-PB)	Laboratory of Respiratory Viruses and Measles, Oswaldo Cruz Institute, FIOCRUZ	Paola Resende et al
EPI_ISL_792602	Laboratório Central de Saúde Pública do Estado da Paraíba (LACEN-PB)	Laboratory of Respiratory Viruses and Measles, Oswaldo Cruz Institute, FIOCRUZ	Paola Resende et al
EPI_ISL_792603	Laboratório Central de Saúde Pública do Estado da Paraíba (LACEN-PB)	Laboratory of Respiratory Viruses and Measles, Oswaldo Cruz Institute, FIOCRUZ	Paola Resende et al
EPI_ISL_792604	Laboratório Central de Saúde Pública do Estado da Paraíba (LACEN-PB)	Laboratory of Respiratory Viruses and Measles, Oswaldo Cruz Institute, FIOCRUZ	Paola Resende et al
EPI_ISL_792605	Laboratório Central de Saúde Pública do Estado da Paraíba (LACEN-PB)	Laboratory of Respiratory Viruses and Measles, Oswaldo Cruz Institute, FIOCRUZ	Paola Resende et al
EPI_ISL_792606	Laboratório Central de Saúde Pública do Estado da Paraíba (LACEN-PB)	Laboratory of Respiratory Viruses and Measles, Oswaldo Cruz Institute, FIOCRUZ	Paola Resende et al
EPI_ISL_792607	Laboratório Central de Saúde Pública do Estado da Paraíba (LACEN-PB)	Laboratory of Respiratory Viruses and Measles, Oswaldo Cruz Institute, FIOCRUZ	Paola Resende et al
EPI_ISL_792608	Laboratório Central de Saúde Pública do Estado da Paraíba (LACEN-PB)	Laboratory of Respiratory Viruses and Measles, Oswaldo Cruz Institute, FIOCRUZ	Paola Resende et al
EPI_ISL_792609	Laboratório Central de Saúde Pública do Estado da Paraíba (LACEN-PB)	Laboratory of Respiratory Viruses and Measles, Oswaldo Cruz Institute, FIOCRUZ	Paola Resende et al
EPI_ISL_792610	Laboratório Central de Saúde Pública do Estado da Paraíba (LACEN-PB)	Laboratory of Respiratory Viruses and Measles, Oswaldo Cruz Institute, FIOCRUZ	Paola Resende et al
EPI_ISL_792611	Laboratório Central de Saúde Pública do Estado da Paraíba (LACEN-PB)	Laboratory of Respiratory Viruses and Measles, Oswaldo Cruz Institute, FIOCRUZ	Paola Resende et al
EPI_ISL_792612	Laboratório Central de Saúde Pública do Estado da Paraíba (LACEN-PB)	Laboratory of Respiratory Viruses and Measles, Oswaldo Cruz Institute, FIOCRUZ	Paola Resende et al
EPI_ISL_792613	Laboratório Central de Saúde Pública do Estado da Paraíba (LACEN-PB)	Laboratory of Respiratory Viruses and Measles, Oswaldo Cruz Institute, FIOCRUZ	Paola Resende et al
EPI_ISL_792614	Laboratório Central de Saúde Pública do Estado da Paraíba (LACEN-PB)	Laboratory of Respiratory Viruses and Measles, Oswaldo Cruz Institute, FIOCRUZ	Paola Resende et al
EPI_ISL_792615	Laboratório Central de Saúde Pública do Estado da Paraíba (LACEN-PB)	Laboratory of Respiratory Viruses and Measles, Oswaldo Cruz Institute, FIOCRUZ	Paola Resende et al

EPI_ISL_792616	Laboratório Central de Saúde Pública do Estado da Paraíba (LACEN-PB)	Laboratory of Respiratory Viruses and Measles, Oswaldo Cruz Institute, FIOCRUZ	Paola Resende et al
EPI_ISL_792617	Laboratório Central de Saúde Pública do Estado da Paraíba (LACEN-PB)	Laboratory of Respiratory Viruses and Measles, Oswaldo Cruz Institute, FIOCRUZ	Paola Resende et al
EPI_ISL_792618	Laboratório Central de Saúde Pública do Estado da Paraíba (LACEN-PB)	Laboratory of Respiratory Viruses and Measles, Oswaldo Cruz Institute, FIOCRUZ	Paola Resende et al
EPI_ISL_792619	Laboratório Central de Saúde Pública do Estado da Paraíba (LACEN-PB)	Laboratory of Respiratory Viruses and Measles, Oswaldo Cruz Institute, FIOCRUZ	Paola Resende et al
EPI_ISL_792620	Laboratório Central de Saúde Pública do Estado da Paraíba (LACEN-PB)	Laboratory of Respiratory Viruses and Measles, Oswaldo Cruz Institute, FIOCRUZ	Paola Resende et al
EPI_ISL_792621	Laboratório Central de Saúde Pública do Estado da Paraíba (LACEN-PB)	Laboratory of Respiratory Viruses and Measles, Oswaldo Cruz Institute, FIOCRUZ	Paola Resende et al
EPI_ISL_792622	Laboratório Central de Saúde Pública do Estado da Paraíba (LACEN-PB)	Laboratory of Respiratory Viruses and Measles, Oswaldo Cruz Institute, FIOCRUZ	Paola Resende et al
EPI_ISL_792623	Laboratório Central de Saúde Pública do Estado da Paraíba (LACEN-PB)	Laboratory of Respiratory Viruses and Measles, Oswaldo Cruz Institute, FIOCRUZ	Paola Resende et al
EPI_ISL_792624	Laboratório Central de Saúde Pública do Estado da Paraíba (LACEN-PB)	Laboratory of Respiratory Viruses and Measles, Oswaldo Cruz Institute, FIOCRUZ	Paola Resende et al
EPI_ISL_792625	Laboratório Central de Saúde Pública do Estado da Paraíba (LACEN-PB)	Laboratory of Respiratory Viruses and Measles, Oswaldo Cruz Institute, FIOCRUZ	Paola Resende et al
EPI_ISL_792626	Laboratório Central de Saúde Pública do Estado da Paraíba (LACEN-PB)	Laboratory of Respiratory Viruses and Measles, Oswaldo Cruz Institute, FIOCRUZ	Paola Resende et al
EPI_ISL_792627	Laboratório Central de Saúde Pública do Estado da Paraíba (LACEN-PB)	Laboratory of Respiratory Viruses and Measles, Oswaldo Cruz Institute, FIOCRUZ	Paola Resende et al
EPI_ISL_792628	Laboratório Central de Saúde Pública do Estado da Paraíba (LACEN-PB)	Laboratory of Respiratory Viruses and Measles, Oswaldo Cruz Institute, FIOCRUZ	Paola Resende et al
EPI_ISL_792629	Laboratório Central de Saúde Pública do Estado da Paraíba (LACEN-PB)	Laboratory of Respiratory Viruses and Measles, Oswaldo Cruz Institute, FIOCRUZ	Paola Resende et al
EPI_ISL_792630	Laboratório Central de Saúde Pública do Estado da Paraíba (LACEN-PB)	Laboratory of Respiratory Viruses and Measles, Oswaldo Cruz Institute, FIOCRUZ	Paola Resende et al
EPI_ISL_792631	Laboratório Central de Saúde Pública do Estado da Paraíba (LACEN-PB)	Laboratory of Respiratory Viruses and Measles, Oswaldo Cruz Institute, FIOCRUZ	Paola Resende et al
EPI_ISL_792632	Laboratório Central de Saúde Pública do Estado da Paraíba (LACEN-PB)	Laboratory of Respiratory Viruses and Measles, Oswaldo Cruz Institute, FIOCRUZ	Paola Resende et al
EPI_ISL_792633	Laboratório Central de Saúde Pública do Estado da Paraíba (LACEN-PB)	Laboratory of Respiratory Viruses and Measles, Oswaldo Cruz Institute, FIOCRUZ	Paola Resende et al
EPI_ISL_792634	Laboratório Central de Saúde Pública do Estado da Paraíba (LACEN-PB)	Laboratory of Respiratory Viruses and Measles, Oswaldo Cruz Institute, FIOCRUZ	Paola Resende et al
EPI_ISL_792635	Laboratório Central de Saúde Pública do Estado da Paraíba (LACEN-PB)	Laboratory of Respiratory Viruses and Measles, Oswaldo Cruz Institute, FIOCRUZ	Paola Resende et al
EPI_ISL_792636	Laboratório Central de Saúde Pública do Estado da Paraíba (LACEN-PB)	Laboratory of Respiratory Viruses and Measles, Oswaldo Cruz Institute, FIOCRUZ	Paola Resende et al
EPI_ISL_792637	Laboratório Central de Saúde Pública do Estado da Paraíba (LACEN-PB)	Laboratory of Respiratory Viruses and Measles, Oswaldo Cruz Institute, FIOCRUZ	Paola Resende et al

EPI_ISL_792638	Laboratório Central de Saúde Pública do Estado da Paraíba (LACEN-PB)	Laboratory of Respiratory Viruses and Measles, Oswaldo Cruz Institute, FIOCRUZ	Paola Resende et al
EPI_ISL_1402429	Laboratory of Respiratory Viruses and Measles, Oswaldo Cruz Institute, FIOCRUZ	Laboratory of Respiratory Viruses and Measles, Oswaldo Cruz Institute, FIOCRUZ	Paola Resende et al
EPI_ISL_1181416	Laboratorio Central de Saude Publica do Estado da Paraiba (LACEN-PB)	Laboratory of Respiratory Viruses and Measles, Oswaldo Cruz Institute, FIOCRUZ	Paola Resende et al
EPI_ISL_1181422	Laboratorio Central de Saude Publica do Estado da Paraiba (LACEN-PB)	Laboratory of Respiratory Viruses and Measles, Oswaldo Cruz Institute, FIOCRUZ	Paola Resende et al
EPI_ISL_1181362	Instituto Aggeu Magalhães - Oswaldo Cruz Foundation, FIOCRUZ (FIOCRUZ-PE)	Laboratory of Respiratory Viruses and Measles, Oswaldo Cruz Institute, FIOCRUZ	Paola Resende et al
EPI_ISL_1181526	Laboratorio Central de Saude Publica do Estado do Parana (LACEN-PR)	Laboratory of Respiratory Viruses and Measles, Oswaldo Cruz Institute, FIOCRUZ	Paola Resende et al
EPI_ISL_1181527	Laboratorio Central de Saude Publica do Estado do Parana (LACEN-PR)	Laboratory of Respiratory Viruses and Measles, Oswaldo Cruz Institute, FIOCRUZ	Paola Resende et al
EPI_ISL_1181528	Laboratorio Central de Saude Publica do Estado do Parana (LACEN-PR)	Laboratory of Respiratory Viruses and Measles, Oswaldo Cruz Institute, FIOCRUZ	Paola Resende et al
EPI_ISL_1181563	Laboratorio Central de Saude Publica do Estado do Parana (LACEN-PR)	Laboratory of Respiratory Viruses and Measles, Oswaldo Cruz Institute, FIOCRUZ	Paola Resende et al
EPI_ISL_1181551	Laboratorio Central de Saude Publica do Estado do Parana (LACEN-PR)	Laboratory of Respiratory Viruses and Measles, Oswaldo Cruz Institute, FIOCRUZ	Paola Resende et al
EPI_ISL_1181552	Laboratorio Central de Saude Publica do Estado do Parana (LACEN-PR)	Laboratory of Respiratory Viruses and Measles, Oswaldo Cruz Institute, FIOCRUZ	Paola Resende et al
EPI_ISL_1181553	Laboratorio Central de Saude Publica do Estado do Parana (LACEN-PR)	Laboratory of Respiratory Viruses and Measles, Oswaldo Cruz Institute, FIOCRUZ	Paola Resende et al
EPI_ISL_1181543	Laboratorio Central de Saude Publica do Estado do Parana (LACEN-PR)	Laboratory of Respiratory Viruses and Measles, Oswaldo Cruz Institute, FIOCRUZ	Paola Resende et al
EPI_ISL_1181537	Laboratorio Central de Saude Publica do Estado do Parana (LACEN-PR)	Laboratory of Respiratory Viruses and Measles, Oswaldo Cruz Institute, FIOCRUZ	Paola Resende et al
EPI_ISL_1181533	Laboratorio Central de Saude Publica do Estado do Parana (LACEN-PR)	Laboratory of Respiratory Viruses and Measles, Oswaldo Cruz Institute, FIOCRUZ	Paola Resende et al
EPI_ISL_1181534	Laboratorio Central de Saude Publica do Estado do Parana (LACEN-PR)	Laboratory of Respiratory Viruses and Measles, Oswaldo Cruz Institute, FIOCRUZ	Paola Resende et al
EPI_ISL_1181538	Laboratorio Central de Saude Publica do Estado do Parana (LACEN-PR)	Laboratory of Respiratory Viruses and Measles, Oswaldo Cruz Institute, FIOCRUZ	Paola Resende et al
EPI_ISL_1181539	Laboratorio Central de Saude Publica do Estado do Parana (LACEN-PR)	Laboratory of Respiratory Viruses and Measles, Oswaldo Cruz Institute, FIOCRUZ	Paola Resende et al
EPI_ISL_1181392	Laboratorio Central de Saude Publica do Estado do Parana (LACEN-PR)	Laboratory of Respiratory Viruses and Measles, Oswaldo Cruz Institute, FIOCRUZ	Paola Resende et al
EPI_ISL_1181545	Laboratorio Central de Saude Publica do Estado do Parana (LACEN-PR)	Laboratory of Respiratory Viruses and Measles, Oswaldo Cruz Institute, FIOCRUZ	Paola Resende et al
EPI_ISL_1181540	Laboratorio Central de Saude Publica do Estado do Parana (LACEN-PR)	Laboratory of Respiratory Viruses and Measles, Oswaldo Cruz Institute, FIOCRUZ	Paola Resende et al
EPI_ISL_1181544	Laboratorio Central de Saude Publica do Estado do Parana (LACEN-PR)	Laboratory of Respiratory Viruses and Measles, Oswaldo Cruz Institute, FIOCRUZ	Paola Resende et al

EPI_ISL_1181541	Laboratorio Central de Saude Publica do Estado do Parana (LACEN-PR)	Laboratory of Respiratory Viruses and Measles, Oswaldo Cruz Institute, FIOCRUZ	Paola Resende et al
EPI_ISL_1181542	Laboratorio Central de Saude Publica do Estado do Parana (LACEN-PR)	Laboratory of Respiratory Viruses and Measles, Oswaldo Cruz Institute, FIOCRUZ	Paola Resende et al
EPI_ISL_1181605	Laboratorio Central de Saude Publica do Estado do Parana (LACEN-PR)	Laboratory of Respiratory Viruses and Measles, Oswaldo Cruz Institute, FIOCRUZ	Paola Resende et al
EPI_ISL_1181584	Laboratorio Central de Saude Publica do Estado do Parana (LACEN-PR)	Laboratory of Respiratory Viruses and Measles, Oswaldo Cruz Institute, FIOCRUZ	Paola Resende et al
EPI_ISL_1181581	Laboratorio Central de Saude Publica do Estado do Parana (LACEN-PR)	Laboratory of Respiratory Viruses and Measles, Oswaldo Cruz Institute, FIOCRUZ	Paola Resende et al
EPI_ISL_1181580	Laboratorio Central de Saude Publica do Estado do Parana (LACEN-PR)	Laboratory of Respiratory Viruses and Measles, Oswaldo Cruz Institute, FIOCRUZ	Paola Resende et al
EPI_ISL_1181576	Laboratorio Central de Saude Publica do Estado do Parana (LACEN-PR)	Laboratory of Respiratory Viruses and Measles, Oswaldo Cruz Institute, FIOCRUZ	Paola Resende et al
EPI_ISL_1181536	Laboratorio Central de Saude Publica do Estado do Parana (LACEN-PR)	Laboratory of Respiratory Viruses and Measles, Oswaldo Cruz Institute, FIOCRUZ	Paola Resende et al
EPI_ISL_1181530	Laboratorio Central de Saude Publica do Estado do Parana (LACEN-PR)	Laboratory of Respiratory Viruses and Measles, Oswaldo Cruz Institute, FIOCRUZ	Paola Resende et al
EPI_ISL_1181517	Laboratorio Central de Saude Publica do Estado do Parana (LACEN-PR)	Laboratory of Respiratory Viruses and Measles, Oswaldo Cruz Institute, FIOCRUZ	Paola Resende et al
EPI_ISL_1181498	Laboratorio Central de Saude Publica do Estado do Parana (LACEN-PR)	Laboratory of Respiratory Viruses and Measles, Oswaldo Cruz Institute, FIOCRUZ	Paola Resende et al
EPI_ISL_1534000	Laboratorio Central de Saude Publica do Estado do Parana (LACEN-PR)	Laboratory of Respiratory Viruses and Measles, Oswaldo Cruz Institute, FIOCRUZ	Paola Resende et al
EPI_ISL_1534001	Laboratorio Central de Saude Publica do Estado do Parana (LACEN-PR)	Laboratory of Respiratory Viruses and Measles, Oswaldo Cruz Institute, FIOCRUZ	Paola Resende et al
EPI_ISL_1533999	Laboratorio Central de Saude Publica do Estado do Parana (LACEN-PR)	Laboratory of Respiratory Viruses and Measles, Oswaldo Cruz Institute, FIOCRUZ	Paola Resende et al
EPI_ISL_1533978	Laboratorio Central de Saude Publica do Estado do Parana (LACEN-PR)	Laboratory of Respiratory Viruses and Measles, Oswaldo Cruz Institute, FIOCRUZ	Paola Resende et al
EPI_ISL_1181474	Laboratorio Central de Saude Publica do Estado do Parana (LACEN-PR)	Laboratory of Respiratory Viruses and Measles, Oswaldo Cruz Institute, FIOCRUZ	Paola Resende et al
EPI_ISL_792645	Laboratório Central de Saúde Pública do Estado do Paraná (LACEN-PR)	Laboratory of Respiratory Viruses and Measles, Oswaldo Cruz Institute, FIOCRUZ	Paola Resende et al
EPI_ISL_792646	Laboratório Central de Saúde Pública do Estado do Paraná (LACEN-PR)	Laboratory of Respiratory Viruses and Measles, Oswaldo Cruz Institute, FIOCRUZ	Paola Resende et al
EPI_ISL_792647	Laboratório Central de Saúde Pública do Estado do Paraná (LACEN-PR)	Laboratory of Respiratory Viruses and Measles, Oswaldo Cruz Institute, FIOCRUZ	Paola Resende et al
EPI_ISL_792648	Laboratório Central de Saúde Pública do Estado do Paraná (LACEN-PR)	Laboratory of Respiratory Viruses and Measles, Oswaldo Cruz Institute, FIOCRUZ	Paola Resende et al
EPI_ISL_792649	Laboratório Central de Saúde Pública do Estado do Paraná (LACEN-PR)	Laboratory of Respiratory Viruses and Measles, Oswaldo Cruz Institute, FIOCRUZ	Paola Resende et al
EPI_ISL_792650	Laboratório Central de Saúde Pública do Estado do Paraná (LACEN-PR)	Laboratory of Respiratory Viruses and Measles, Oswaldo Cruz Institute, FIOCRUZ	Paola Resende et al

EPI_ISL_792651	Laboratório Central de Saúde Pública do Estado do Paraná (LACEN-PR)	Laboratory of Respiratory Viruses and Measles, Oswaldo Cruz Institute, FIOCRUZ	Paola Resende et al
EPI_ISL_792652	Laboratório Central de Saúde Pública do Estado do Paraná (LACEN-PR)	Laboratory of Respiratory Viruses and Measles, Oswaldo Cruz Institute, FIOCRUZ	Paola Resende et al
EPI_ISL_792653	Laboratório Central de Saúde Pública do Estado do Paraná (LACEN-PR)	Laboratory of Respiratory Viruses and Measles, Oswaldo Cruz Institute, FIOCRUZ	Paola Resende et al
EPI_ISL_792654	Laboratório Central de Saúde Pública do Estado do Paraná (LACEN-PR)	Laboratory of Respiratory Viruses and Measles, Oswaldo Cruz Institute, FIOCRUZ	Paola Resende et al
EPI_ISL_1181617	Laboratorio Central de Saude Publica do Estado do Parana (LACEN-PR)	Laboratory of Respiratory Viruses and Measles, Oswaldo Cruz Institute, FIOCRUZ	Paola Resende et al
EPI_ISL_1181618	Laboratorio Central de Saude Publica do Estado do Parana (LACEN-PR)	Laboratory of Respiratory Viruses and Measles, Oswaldo Cruz Institute, FIOCRUZ	Paola Resende et al
EPI_ISL_1181619	Laboratorio Central de Saude Publica do Estado do Parana (LACEN-PR)	Laboratory of Respiratory Viruses and Measles, Oswaldo Cruz Institute, FIOCRUZ	Paola Resende et al
EPI_ISL_1181614	Laboratorio Central de Saude Publica do Estado do Parana (LACEN-PR)	Laboratory of Respiratory Viruses and Measles, Oswaldo Cruz Institute, FIOCRUZ	Paola Resende et al
EPI_ISL_1181610	Laboratorio Central de Saude Publica do Estado do Parana (LACEN-PR)	Laboratory of Respiratory Viruses and Measles, Oswaldo Cruz Institute, FIOCRUZ	Paola Resende et al
EPI_ISL_1181615	Laboratorio Central de Saude Publica do Estado do Parana (LACEN-PR)	Laboratory of Respiratory Viruses and Measles, Oswaldo Cruz Institute, FIOCRUZ	Paola Resende et al
EPI_ISL_1181609	Laboratorio Central de Saude Publica do Estado do Parana (LACEN-PR)	Laboratory of Respiratory Viruses and Measles, Oswaldo Cruz Institute, FIOCRUZ	Paola Resende et al
EPI_ISL_1181616	Laboratorio Central de Saude Publica do Estado do Parana (LACEN-PR)	Laboratory of Respiratory Viruses and Measles, Oswaldo Cruz Institute, FIOCRUZ	Paola Resende et al
EPI_ISL_1219132	Laboratorio Central de Saude Publica do Estado do Parana (LACEN-PR)	Laboratory of Respiratory Viruses and Measles, Oswaldo Cruz Institute, FIOCRUZ	Paola Resende et al
EPI_ISL_1181394	Laboratorio Central de Saude Publica do Estado do Parana (LACEN-PR)	Laboratory of Respiratory Viruses and Measles, Oswaldo Cruz Institute, FIOCRUZ	Paola Resende et al
EPI_ISL_1219133	Laboratorio Central de Saude Publica do Estado do Parana (LACEN-PR)	Laboratory of Respiratory Viruses and Measles, Oswaldo Cruz Institute, FIOCRUZ	Paola Resende et al
EPI_ISL_541340	Laboratório Central de Saúde Pública do Estado do Paraná (LACEN-PR)	Laboratory of Respiratory Viruses and Measles, Oswaldo Cruz Institute, FIOCRUZ	Paola Resende et al
EPI_ISL_541341	Laboratório Central de Saúde Pública do Estado do Paraná (LACEN-PR)	Laboratory of Respiratory Viruses and Measles, Oswaldo Cruz Institute, FIOCRUZ	Paola Resende et al
EPI_ISL_541342	Laboratório Central de Saúde Pública do Estado do Paraná (LACEN-PR)	Laboratory of Respiratory Viruses and Measles, Oswaldo Cruz Institute, FIOCRUZ	Paola Resende et al
EPI_ISL_541343	Laboratório Central de Saúde Pública do Estado do Paraná (LACEN-PR)	Laboratory of Respiratory Viruses and Measles, Oswaldo Cruz Institute, FIOCRUZ	Paola Resende et al
EPI_ISL_541344	Laboratório Central de Saúde Pública do Estado do Paraná (LACEN-PR)	Laboratory of Respiratory Viruses and Measles, Oswaldo Cruz Institute, FIOCRUZ	Paola Resende et al
EPI_ISL_541345	Laboratório Central de Saúde Pública do Estado do Paraná (LACEN-PR)	Laboratory of Respiratory Viruses and Measles, Oswaldo Cruz Institute, FIOCRUZ	Paola Resende et al
EPI_ISL_541346	Laboratório Central de Saúde Pública do Estado do Paraná (LACEN-PR)	Laboratory of Respiratory Viruses and Measles, Oswaldo Cruz Institute, FIOCRUZ	Paola Resende et al

EPI_ISL_1181566	Laboratorio Central de Saude Publica do Estado do Parana (LACEN-PR)	Laboratory of Respiratory Viruses and Measles, Oswaldo Cruz Institute, FIOCRUZ	Paola Resende et al
EPI_ISL_1181568	Laboratorio Central de Saude Publica do Estado do Parana (LACEN-PR)	Laboratory of Respiratory Viruses and Measles, Oswaldo Cruz Institute, FIOCRUZ	Paola Resende et al
EPI_ISL_1181570	Laboratorio Central de Saude Publica do Estado do Parana (LACEN-PR)	Laboratory of Respiratory Viruses and Measles, Oswaldo Cruz Institute, FIOCRUZ	Paola Resende et al
EPI_ISL_1181564	Laboratorio Central de Saude Publica do Estado do Parana (LACEN-PR)	Laboratory of Respiratory Viruses and Measles, Oswaldo Cruz Institute, FIOCRUZ	Paola Resende et al
EPI_ISL_1181555	Laboratorio Central de Saude Publica do Estado do Parana (LACEN-PR)	Laboratory of Respiratory Viruses and Measles, Oswaldo Cruz Institute, FIOCRUZ	Paola Resende et al
EPI_ISL_1181496	Laboratory of Respiratory Viruses and Measles, Oswaldo Cruz Institute, FIOCRUZ	Laboratory of Respiratory Viruses and Measles, Oswaldo Cruz Institute, FIOCRUZ	Paola Resende et al
EPI_ISL_1181495	Laboratory of Respiratory Viruses and Measles, Oswaldo Cruz Institute, FIOCRUZ	Laboratory of Respiratory Viruses and Measles, Oswaldo Cruz Institute, FIOCRUZ	Paola Resende et al
EPI_ISL_456076	Laboratório Central de Saúde Pública Noel Nutels (LACEN-RJ)	Laboratory of Respiratory Viruses and Measles, Oswaldo Cruz Institute, FIOCRUZ	Paola Resende et al
EPI_ISL_1181489	Laboratory of Respiratory Viruses and Measles, Oswaldo Cruz Institute, FIOCRUZ	Laboratory of Respiratory Viruses and Measles, Oswaldo Cruz Institute, FIOCRUZ	Paola Resende et al
EPI_ISL_1181361	Laboratory of Respiratory Viruses and Measles, Oswaldo Cruz Institute, FIOCRUZ	Laboratory of Respiratory Viruses and Measles, Oswaldo Cruz Institute, FIOCRUZ	Paola Resende et al
EPI_ISL_1181488	Laboratory of Respiratory Viruses and Measles, Oswaldo Cruz Institute, FIOCRUZ	Laboratory of Respiratory Viruses and Measles, Oswaldo Cruz Institute, FIOCRUZ	Paola Resende et al
EPI_ISL_1181486	Laboratory of Respiratory Viruses and Measles, Oswaldo Cruz Institute, FIOCRUZ	Laboratory of Respiratory Viruses and Measles, Oswaldo Cruz Institute, FIOCRUZ	Paola Resende et al
EPI_ISL_1181483	Laboratory of Respiratory Viruses and Measles, Oswaldo Cruz Institute, FIOCRUZ	Laboratory of Respiratory Viruses and Measles, Oswaldo Cruz Institute, FIOCRUZ	Paola Resende et al
EPI_ISL_1181388	Laboratory of Respiratory Viruses and Measles, Oswaldo Cruz Institute, FIOCRUZ	Laboratory of Respiratory Viruses and Measles, Oswaldo Cruz Institute, FIOCRUZ	Paola Resende et al
EPI_ISL_1181613	Laboratorio Central de Saude Publica do Estado do Rio de Janeiro (LACEN-RJ)	Laboratory of Respiratory Viruses and Measles, Oswaldo Cruz Institute, FIOCRUZ	Paola Resende et al
EPI_ISL_1181477	Laboratory of Respiratory Viruses and Measles, Oswaldo Cruz Institute, FIOCRUZ	Laboratory of Respiratory Viruses and Measles, Oswaldo Cruz Institute, FIOCRUZ	Paola Resende et al
EPI_ISL_1181476	Laboratory of Respiratory Viruses and Measles, Oswaldo Cruz Institute, FIOCRUZ	Laboratory of Respiratory Viruses and Measles, Oswaldo Cruz Institute, FIOCRUZ	Paola Resende et al
EPI_ISL_1181475	Laboratory of Respiratory Viruses and Measles, Oswaldo Cruz Institute, FIOCRUZ	Laboratory of Respiratory Viruses and Measles, Oswaldo Cruz Institute, FIOCRUZ	Paola Resende et al
EPI_ISL_1181470	Laboratory of Respiratory Viruses and Measles, Oswaldo Cruz Institute, FIOCRUZ	Laboratory of Respiratory Viruses and Measles, Oswaldo Cruz Institute, FIOCRUZ	Paola Resende et al
EPI_ISL_1534016	Laboratory of Respiratory Viruses and Measles, Oswaldo Cruz Institute, FIOCRUZ	Laboratory of Respiratory Viruses and Measles, Oswaldo Cruz Institute, FIOCRUZ	Paola Resende et al
EPI_ISL_1181466	Laboratory of Respiratory Viruses and Measles, Oswaldo Cruz Institute, FIOCRUZ	Laboratory of Respiratory Viruses and Measles, Oswaldo Cruz Institute, FIOCRUZ	Paola Resende et al
EPI_ISL_1181467	Laboratory of Respiratory Viruses and Measles, Oswaldo Cruz Institute, FIOCRUZ	Laboratory of Respiratory Viruses and Measles, Oswaldo Cruz Institute, FIOCRUZ	Paola Resende et al

EPI_ISL_1181465	Laboratory of Respiratory Viruses and Measles, Oswaldo Cruz Institute, FIOCRUZ	Laboratory of Respiratory Viruses and Measles, Oswaldo Cruz Institute, FIOCRUZ	Paola Resende et al
EPI_ISL_1533991	Laboratorio de Virologia Molecular / UFRJ	Laboratory of Respiratory Viruses and Measles, Oswaldo Cruz Institute, FIOCRUZ	Paola Resende et al
EPI_ISL_1533995	Laboratorio de Virologia Molecular / UFRJ	Laboratory of Respiratory Viruses and Measles, Oswaldo Cruz Institute, FIOCRUZ	Paola Resende et al
EPI_ISL_1533998	Laboratorio de Virologia Molecular / UFRJ	Laboratory of Respiratory Viruses and Measles, Oswaldo Cruz Institute, FIOCRUZ	Paola Resende et al
EPI_ISL_1181366	Laboratory of Respiratory Viruses and Measles, Oswaldo Cruz Institute, FIOCRUZ	Laboratory of Respiratory Viruses and Measles, Oswaldo Cruz Institute, FIOCRUZ	Paola Resende et al
EPI_ISL_1181464	Laboratory of Respiratory Viruses and Measles, Oswaldo Cruz Institute, FIOCRUZ	Laboratory of Respiratory Viruses and Measles, Oswaldo Cruz Institute, FIOCRUZ	Paola Resende et al
EPI_ISL_1181463	Laboratory of Respiratory Viruses and Measles, Oswaldo Cruz Institute, FIOCRUZ	Laboratory of Respiratory Viruses and Measles, Oswaldo Cruz Institute, FIOCRUZ	Paola Resende et al
EPI_ISL_1181374	Laboratory of Respiratory Viruses and Measles, Oswaldo Cruz Institute, FIOCRUZ	Laboratory of Respiratory Viruses and Measles, Oswaldo Cruz Institute, FIOCRUZ	Paola Resende et al
EPI_ISL_1181461	Laboratory of Respiratory Viruses and Measles, Oswaldo Cruz Institute, FIOCRUZ	Laboratory of Respiratory Viruses and Measles, Oswaldo Cruz Institute, FIOCRUZ	Paola Resende et al
EPI_ISL_1181457	Laboratory of Respiratory Viruses and Measles, Oswaldo Cruz Institute, FIOCRUZ	Laboratory of Respiratory Viruses and Measles, Oswaldo Cruz Institute, FIOCRUZ	Paola Resende et al
EPI_ISL_1181458	Laboratory of Respiratory Viruses and Measles, Oswaldo Cruz Institute, FIOCRUZ	Laboratory of Respiratory Viruses and Measles, Oswaldo Cruz Institute, FIOCRUZ	Paola Resende et al
EPI_ISL_1181462	Laboratory of Respiratory Viruses and Measles, Oswaldo Cruz Institute, FIOCRUZ	Laboratory of Respiratory Viruses and Measles, Oswaldo Cruz Institute, FIOCRUZ	Paola Resende et al
EPI_ISL_1181367	Laboratory of Respiratory Viruses and Measles, Oswaldo Cruz Institute, FIOCRUZ	Laboratory of Respiratory Viruses and Measles, Oswaldo Cruz Institute, FIOCRUZ	Paola Resende et al
EPI_ISL_1181377	Laboratory of Respiratory Viruses and Measles, Oswaldo Cruz Institute, FIOCRUZ	Laboratory of Respiratory Viruses and Measles, Oswaldo Cruz Institute, FIOCRUZ	Paola Resende et al
EPI_ISL_1181455	Laboratory of Respiratory Viruses and Measles, Oswaldo Cruz Institute, FIOCRUZ	Laboratory of Respiratory Viruses and Measles, Oswaldo Cruz Institute, FIOCRUZ	Paola Resende et al
EPI_ISL_1181360	Laboratory of Respiratory Viruses and Measles, Oswaldo Cruz Institute, FIOCRUZ	Laboratory of Respiratory Viruses and Measles, Oswaldo Cruz Institute, FIOCRUZ	Paola Resende et al
EPI_ISL_1181456	Laboratory of Respiratory Viruses and Measles, Oswaldo Cruz Institute, FIOCRUZ	Laboratory of Respiratory Viruses and Measles, Oswaldo Cruz Institute, FIOCRUZ	Paola Resende et al
EPI_ISL_1181454	Laboratory of Respiratory Viruses and Measles, Oswaldo Cruz Institute, FIOCRUZ	Laboratory of Respiratory Viruses and Measles, Oswaldo Cruz Institute, FIOCRUZ	Paola Resende et al
EPI_ISL_1181382	Laboratory of Respiratory Viruses and Measles, Oswaldo Cruz Institute, FIOCRUZ	Laboratory of Respiratory Viruses and Measles, Oswaldo Cruz Institute, FIOCRUZ	Paola Resende et al
EPI_ISL_1181448	Laboratory of Respiratory Viruses and Measles, Oswaldo Cruz Institute, FIOCRUZ	Laboratory of Respiratory Viruses and Measles, Oswaldo Cruz Institute, FIOCRUZ	Paola Resende et al
EPI_ISL_1181446	Laboratory of Respiratory Viruses and Measles, Oswaldo Cruz Institute, FIOCRUZ	Laboratory of Respiratory Viruses and Measles, Oswaldo Cruz Institute, FIOCRUZ	Paola Resende et al
EPI_ISL_1181447	Laboratory of Respiratory Viruses and Measles, Oswaldo Cruz Institute, FIOCRUZ	Laboratory of Respiratory Viruses and Measles, Oswaldo Cruz Institute, FIOCRUZ	Paola Resende et al

EPI_ISL_1181381	Laboratory of Respiratory Viruses and Measles, Oswaldo Cruz Institute, FIOCRUZ	Laboratory of Respiratory Viruses and Measles, Oswaldo Cruz Institute, FIOCRUZ	Paola Resende et al
EPI_ISL_1181383	Laboratory of Respiratory Viruses and Measles, Oswaldo Cruz Institute, FIOCRUZ	Laboratory of Respiratory Viruses and Measles, Oswaldo Cruz Institute, FIOCRUZ	Paola Resende et al
EPI_ISL_1181442	Laboratory of Respiratory Viruses and Measles, Oswaldo Cruz Institute, FIOCRUZ	Laboratory of Respiratory Viruses and Measles, Oswaldo Cruz Institute, FIOCRUZ	Paola Resende et al
EPI_ISL_456088	Laboratório Central de Saúde Pública Noel Nutels (LACEN-RJ)	Laboratory of Respiratory Viruses and Measles, Oswaldo Cruz Institute, FIOCRUZ	Paola Resende et al
EPI_ISL_1181437	Laboratory of Respiratory Viruses and Measles, Oswaldo Cruz Institute, FIOCRUZ	Laboratory of Respiratory Viruses and Measles, Oswaldo Cruz Institute, FIOCRUZ	Paola Resende et al
EPI_ISL_1181438	Laboratory of Respiratory Viruses and Measles, Oswaldo Cruz Institute, FIOCRUZ	Laboratory of Respiratory Viruses and Measles, Oswaldo Cruz Institute, FIOCRUZ	Paola Resende et al
EPI_ISL_1181439	Laboratory of Respiratory Viruses and Measles, Oswaldo Cruz Institute, FIOCRUZ	Laboratory of Respiratory Viruses and Measles, Oswaldo Cruz Institute, FIOCRUZ	Paola Resende et al
EPI_ISL_456102	Laboratory of Respiratory Viruses and Measles, Oswaldo Cruz Institute, FIOCRUZ	Laboratory of Respiratory Viruses and Measles, Oswaldo Cruz Institute, FIOCRUZ	Paola Resende et al
EPI_ISL_541354	Laboratory of Respiratory Viruses and Measles, Oswaldo Cruz Institute, FIOCRUZ	Laboratory of Respiratory Viruses and Measles, Oswaldo Cruz Institute, FIOCRUZ	Paola Resende et al
EPI_ISL_541355	Laboratory of Respiratory Viruses and Measles, Oswaldo Cruz Institute, FIOCRUZ	Laboratory of Respiratory Viruses and Measles, Oswaldo Cruz Institute, FIOCRUZ	Paola Resende et al
EPI_ISL_1181353	Laboratorio Central de Saude Publica do Estado do Rio de Janeiro (LACEN-RJ)	Laboratory of Respiratory Viruses and Measles, Oswaldo Cruz Institute, FIOCRUZ	Paola Resende et al
EPI_ISL_1181372	Laboratorio Central de Saude Publica do Estado do Rio de Janeiro (LACEN-RJ)	Laboratory of Respiratory Viruses and Measles, Oswaldo Cruz Institute, FIOCRUZ	Paola Resende et al
EPI_ISL_1181431	Laboratorio Central de Saude Publica do Estado do Rio de Janeiro (LACEN-RJ)	Laboratory of Respiratory Viruses and Measles, Oswaldo Cruz Institute, FIOCRUZ	Paola Resende et al
EPI_ISL_541359	Laboratory of Respiratory Viruses and Measles, Oswaldo Cruz Institute, FIOCRUZ	Laboratory of Respiratory Viruses and Measles, Oswaldo Cruz Institute, FIOCRUZ	Paola Resende et al
EPI_ISL_1181398	Laboratory of Respiratory Viruses and Measles, Oswaldo Cruz Institute, FIOCRUZ	Laboratory of Respiratory Viruses and Measles, Oswaldo Cruz Institute, FIOCRUZ	Paola Resende et al
EPI_ISL_467356	Laboratory of Respiratory Viruses and Measles, Oswaldo Cruz Institute, FIOCRUZ	Laboratory of Respiratory Viruses and Measles, Oswaldo Cruz Institute, FIOCRUZ	Paola Resende et al
EPI_ISL_467359	Laboratory of Respiratory Viruses and Measles, Oswaldo Cruz Institute, FIOCRUZ	Laboratory of Respiratory Viruses and Measles, Oswaldo Cruz Institute, FIOCRUZ	Paola Resende et al
EPI_ISL_467366	Laboratory of Respiratory Viruses and Measles, Oswaldo Cruz Institute, FIOCRUZ	Laboratory of Respiratory Viruses and Measles, Oswaldo Cruz Institute, FIOCRUZ	Paola Resende et al
EPI_ISL_1181425	Laboratorio Central de Saude Publica do Estado do Rio de Janeiro (LACEN-RJ)	Laboratory of Respiratory Viruses and Measles, Oswaldo Cruz Institute, FIOCRUZ	Paola Resende et al
EPI_ISL_1181410	Laboratory of Respiratory Viruses and Measles, Oswaldo Cruz Institute, FIOCRUZ	Laboratory of Respiratory Viruses and Measles, Oswaldo Cruz Institute, FIOCRUZ	Paola Resende et al
EPI_ISL_1181415	Laboratory of Respiratory Viruses and Measles, Oswaldo Cruz Institute, FIOCRUZ	Laboratory of Respiratory Viruses and Measles, Oswaldo Cruz Institute, FIOCRUZ	Paola Resende et al
EPI_ISL_1181578	Laboratory of Respiratory Viruses and Measles, Oswaldo Cruz Institute, FIOCRUZ	Laboratory of Respiratory Viruses and Measles, Oswaldo Cruz Institute, FIOCRUZ	Paola Resende et al

EPI_ISL_1181384	Laboratory of Respiratory Viruses and Measles, Oswaldo Cruz Institute, FIOCRUZ	Laboratory of Respiratory Viruses and Measles, Oswaldo Cruz Institute, FIOCRUZ	Paola Resende et al
EPI_ISL_1181574	Laboratory of Respiratory Viruses and Measles, Oswaldo Cruz Institute, FIOCRUZ	Laboratory of Respiratory Viruses and Measles, Oswaldo Cruz Institute, FIOCRUZ	Paola Resende et al
EPI_ISL_1181359	Laboratory of Respiratory Viruses and Measles, Oswaldo Cruz Institute, FIOCRUZ	Laboratory of Respiratory Viruses and Measles, Oswaldo Cruz Institute, FIOCRUZ	Paola Resende et al
EPI_ISL_1181531	Laboratory of Respiratory Viruses and Measles, Oswaldo Cruz Institute, FIOCRUZ	Laboratory of Respiratory Viruses and Measles, Oswaldo Cruz Institute, FIOCRUZ	Paola Resende et al
EPI_ISL_1181529	Laboratory of Respiratory Viruses and Measles, Oswaldo Cruz Institute, FIOCRUZ	Laboratory of Respiratory Viruses and Measles, Oswaldo Cruz Institute, FIOCRUZ	Paola Resende et al
EPI_ISL_1181523	Laboratory of Respiratory Viruses and Measles, Oswaldo Cruz Institute, FIOCRUZ	Laboratory of Respiratory Viruses and Measles, Oswaldo Cruz Institute, FIOCRUZ	Paola Resende et al
EPI_ISL_1181369	Laboratory of Respiratory Viruses and Measles, Oswaldo Cruz Institute, FIOCRUZ	Laboratory of Respiratory Viruses and Measles, Oswaldo Cruz Institute, FIOCRUZ	Paola Resende et al
EPI_ISL_1181521	Laboratory of Respiratory Viruses and Measles, Oswaldo Cruz Institute, FIOCRUZ	Laboratory of Respiratory Viruses and Measles, Oswaldo Cruz Institute, FIOCRUZ	Paola Resende et al
EPI_ISL_1181519	Laboratory of Respiratory Viruses and Measles, Oswaldo Cruz Institute, FIOCRUZ	Laboratory of Respiratory Viruses and Measles, Oswaldo Cruz Institute, FIOCRUZ	Paola Resende et al
EPI_ISL_1181357	Laboratory of Respiratory Viruses and Measles, Oswaldo Cruz Institute, FIOCRUZ	Laboratory of Respiratory Viruses and Measles, Oswaldo Cruz Institute, FIOCRUZ	Paola Resende et al
EPI_ISL_1181514	Laboratory of Respiratory Viruses and Measles, Oswaldo Cruz Institute, FIOCRUZ	Laboratory of Respiratory Viruses and Measles, Oswaldo Cruz Institute, FIOCRUZ	Paola Resende et al
EPI_ISL_1181515	Laboratory of Respiratory Viruses and Measles, Oswaldo Cruz Institute, FIOCRUZ	Laboratory of Respiratory Viruses and Measles, Oswaldo Cruz Institute, FIOCRUZ	Paola Resende et al
EPI_ISL_1181511	Laboratory of Respiratory Viruses and Measles, Oswaldo Cruz Institute, FIOCRUZ	Laboratory of Respiratory Viruses and Measles, Oswaldo Cruz Institute, FIOCRUZ	Paola Resende et al
EPI_ISL_427302	Laboratory of Respiratory Viruses and Measles, Oswaldo Cruz Institute, FIOCRUZ	Laboratory of Respiratory Viruses and Measles, Oswaldo Cruz Institute, FIOCRUZ	Paola Resende et al
EPI_ISL_1534005	Laboratory of Respiratory Viruses and Measles, Oswaldo Cruz Institute, FIOCRUZ	Laboratory of Respiratory Viruses and Measles, Oswaldo Cruz Institute, FIOCRUZ	Paola Resende et al
EPI_ISL_427303	Laboratory of Respiratory Viruses and Measles, Oswaldo Cruz Institute, FIOCRUZ	Laboratory of Respiratory Viruses and Measles, Oswaldo Cruz Institute, FIOCRUZ	Paola Resende et al
EPI_ISL_427304	Laboratory of Respiratory Viruses and Measles, Oswaldo Cruz Institute, FIOCRUZ	Laboratory of Respiratory Viruses and Measles, Oswaldo Cruz Institute, FIOCRUZ	Paola Resende et al
EPI_ISL_456071	Laboratory of Respiratory Viruses and Measles, Oswaldo Cruz Institute, FIOCRUZ	Laboratory of Respiratory Viruses and Measles, Oswaldo Cruz Institute, FIOCRUZ	Paola Resende et al
EPI_ISL_1181608	Laboratory of Respiratory Viruses and Measles, Oswaldo Cruz Institute, FIOCRUZ	Laboratory of Respiratory Viruses and Measles, Oswaldo Cruz Institute, FIOCRUZ	Paola Resende et al
EPI_ISL_1181499	Laboratory of Respiratory Viruses and Measles, Oswaldo Cruz Institute, FIOCRUZ	Laboratory of Respiratory Viruses and Measles, Oswaldo Cruz Institute, FIOCRUZ	Paola Resende et al
EPI_ISL_1534013	Laboratorio Central de Saude Publica do Estado do Rio Grande do Sul (LACEN-RS)	Laboratory of Respiratory Viruses and Measles, Oswaldo Cruz Institute, FIOCRUZ	Paola Resende et al
EPI_ISL_729801	Laboratório Central de Saúde Pública do Estado do Rio Grande do Sul (LACEN-RS)	Laboratory of Respiratory Viruses and Measles, Oswaldo Cruz Institute, FIOCRUZ	Paola Resende et al

EPI_ISL_729844	Laboratório Central de Saúde Pública do Estado do Rio Grande do Sul (LACEN-RS)	Laboratory of Respiratory Viruses and Measles, Oswaldo Cruz Institute, FIOCRUZ	Paola Resende et al
EPI_ISL_729850	Laboratório Central de Saúde Pública do Estado do Rio Grande do Sul (LACEN-RS)	Laboratory of Respiratory Viruses and Measles, Oswaldo Cruz Institute, FIOCRUZ	Paola Resende et al
EPI_ISL_729856	Laboratório Central de Saúde Pública do Estado do Rio Grande do Sul (LACEN-RS)	Laboratory of Respiratory Viruses and Measles, Oswaldo Cruz Institute, FIOCRUZ	Paola Resende et al
EPI_ISL_729857	Laboratório Central de Saúde Pública do Estado do Rio Grande do Sul (LACEN-RS)	Laboratory of Respiratory Viruses and Measles, Oswaldo Cruz Institute, FIOCRUZ	Paola Resende et al
EPI_ISL_729848	Laboratório Central de Saúde Pública do Estado do Rio Grande do Sul (LACEN-RS)	Laboratory of Respiratory Viruses and Measles, Oswaldo Cruz Institute, FIOCRUZ	Paola Resende et al
EPI_ISL_729845	Laboratório Central de Saúde Pública do Estado do Rio Grande do Sul (LACEN-RS)	Laboratory of Respiratory Viruses and Measles, Oswaldo Cruz Institute, FIOCRUZ	Paola Resende et al
EPI_ISL_729851	Laboratório Central de Saúde Pública do Estado do Rio Grande do Sul (LACEN-RS)	Laboratory of Respiratory Viruses and Measles, Oswaldo Cruz Institute, FIOCRUZ	Paola Resende et al
EPI_ISL_729852	Laboratório Central de Saúde Pública do Estado do Rio Grande do Sul (LACEN-RS)	Laboratory of Respiratory Viruses and Measles, Oswaldo Cruz Institute, FIOCRUZ	Paola Resende et al
EPI_ISL_729858	Laboratório Central de Saúde Pública do Estado do Rio Grande do Sul (LACEN-RS)	Laboratory of Respiratory Viruses and Measles, Oswaldo Cruz Institute, FIOCRUZ	Paola Resende et al
EPI_ISL_729802	Laboratório Central de Saúde Pública do Estado do Rio Grande do Sul (LACEN-RS)	Laboratory of Respiratory Viruses and Measles, Oswaldo Cruz Institute, FIOCRUZ	Paola Resende et al
EPI_ISL_729853	Laboratório Central de Saúde Pública do Estado do Rio Grande do Sul (LACEN-RS)	Laboratory of Respiratory Viruses and Measles, Oswaldo Cruz Institute, FIOCRUZ	Paola Resende et al
EPI_ISL_729859	Laboratório Central de Saúde Pública do Estado do Rio Grande do Sul (LACEN-RS)	Laboratory of Respiratory Viruses and Measles, Oswaldo Cruz Institute, FIOCRUZ	Paola Resende et al
EPI_ISL_729803	Laboratório Central de Saúde Pública do Estado do Rio Grande do Sul (LACEN-RS)	Laboratory of Respiratory Viruses and Measles, Oswaldo Cruz Institute, FIOCRUZ	Paola Resende et al
EPI_ISL_729860	Laboratório Central de Saúde Pública do Estado do Rio Grande do Sul (LACEN-RS)	Laboratory of Respiratory Viruses and Measles, Oswaldo Cruz Institute, FIOCRUZ	Paola Resende et al
EPI_ISL_729849	Laboratório Central de Saúde Pública do Estado do Rio Grande do Sul (LACEN-RS)	Laboratory of Respiratory Viruses and Measles, Oswaldo Cruz Institute, FIOCRUZ	Paola Resende et al
EPI_ISL_729854	Laboratório Central de Saúde Pública do Estado do Rio Grande do Sul (LACEN-RS)	Laboratory of Respiratory Viruses and Measles, Oswaldo Cruz Institute, FIOCRUZ	Paola Resende et al
EPI_ISL_729799	Laboratório Central de Saúde Pública do Estado do Rio Grande do Sul (LACEN-RS)	Laboratory of Respiratory Viruses and Measles, Oswaldo Cruz Institute, FIOCRUZ	Paola Resende et al
EPI_ISL_729855	Laboratório Central de Saúde Pública do Estado do Rio Grande do Sul (LACEN-RS)	Laboratory of Respiratory Viruses and Measles, Oswaldo Cruz Institute, FIOCRUZ	Paola Resende et al
EPI_ISL_729861	Laboratório Central de Saúde Pública do Estado do Rio Grande do Sul (LACEN-RS)	Laboratory of Respiratory Viruses and Measles, Oswaldo Cruz Institute, FIOCRUZ	Paola Resende et al
EPI_ISL_729804	Laboratório Central de Saúde Pública do Estado do Rio Grande do Sul (LACEN-RS)	Laboratory of Respiratory Viruses and Measles, Oswaldo Cruz Institute, FIOCRUZ	Paola Resende et al
EPI_ISL_729805	Laboratório Central de Saúde Pública do Estado do Rio Grande do Sul (LACEN-RS)	Laboratory of Respiratory Viruses and Measles, Oswaldo Cruz Institute, FIOCRUZ	Paola Resende et al
EPI_ISL_729806	Laboratório Central de Saúde Pública do Estado do Rio Grande do Sul (LACEN-RS)	Laboratory of Respiratory Viruses and Measles, Oswaldo Cruz Institute, FIOCRUZ	Paola Resende et al

EPI_ISL_729807	Laboratório Central de Saúde Pública do Estado do Rio Grande do Sul (LACEN-RS)	Laboratory of Respiratory Viruses and Measles, Oswaldo Cruz Institute, FIOCRUZ	Paola Resende et al
EPI_ISL_729808	Laboratório Central de Saúde Pública do Estado do Rio Grande do Sul (LACEN-RS)	Laboratory of Respiratory Viruses and Measles, Oswaldo Cruz Institute, FIOCRUZ	Paola Resende et al
EPI_ISL_729809	Laboratório Central de Saúde Pública do Estado do Rio Grande do Sul (LACEN-RS)	Laboratory of Respiratory Viruses and Measles, Oswaldo Cruz Institute, FIOCRUZ	Paola Resende et al
EPI_ISL_1181468	Laboratorio Central de Saude Publica do Estado do Rio Grande do Sul (LACEN-RS)	Laboratory of Respiratory Viruses and Measles, Oswaldo Cruz Institute, FIOCRUZ	Paola Resende et al
EPI_ISL_729810	Laboratório Central de Saúde Pública do Estado do Rio Grande do Sul (LACEN-RS)	Laboratory of Respiratory Viruses and Measles, Oswaldo Cruz Institute, FIOCRUZ	Paola Resende et al
EPI_ISL_1181451	Laboratorio Central de Saude Publica do Estado do Rio Grande do Sul (LACEN-RS)	Laboratory of Respiratory Viruses and Measles, Oswaldo Cruz Institute, FIOCRUZ	Paola Resende et al
EPI_ISL_1181434	Laboratorio Central de Saude Publica do Estado do Rio Grande do Sul (LACEN-RS)	Laboratory of Respiratory Viruses and Measles, Oswaldo Cruz Institute, FIOCRUZ	Paola Resende et al
EPI_ISL_1181433	Laboratorio Central de Saude Publica do Estado do Rio Grande do Sul (LACEN-RS)	Laboratory of Respiratory Viruses and Measles, Oswaldo Cruz Institute, FIOCRUZ	Paola Resende et al
EPI_ISL_1181620	Laboratorio Central de Saude Publica do Estado do Rio Grande do Sul (LACEN-RS)	Laboratory of Respiratory Viruses and Measles, Oswaldo Cruz Institute, FIOCRUZ	Paola Resende et al
EPI_ISL_729812	Laboratório Central de Saúde Pública do Estado do Rio Grande do Sul (LACEN-RS)	Laboratory of Respiratory Viruses and Measles, Oswaldo Cruz Institute, FIOCRUZ	Paola Resende et al
EPI_ISL_729813	Laboratório Central de Saúde Pública do Estado do Rio Grande do Sul (LACEN-RS)	Laboratory of Respiratory Viruses and Measles, Oswaldo Cruz Institute, FIOCRUZ	Paola Resende et al
EPI_ISL_729814	Laboratório Central de Saúde Pública do Estado do Rio Grande do Sul (LACEN-RS)	Laboratory of Respiratory Viruses and Measles, Oswaldo Cruz Institute, FIOCRUZ	Paola Resende et al
EPI_ISL_729815	Laboratório Central de Saúde Pública do Estado do Rio Grande do Sul (LACEN-RS)	Laboratory of Respiratory Viruses and Measles, Oswaldo Cruz Institute, FIOCRUZ	Paola Resende et al
EPI_ISL_729816	Laboratório Central de Saúde Pública do Estado do Rio Grande do Sul (LACEN-RS)	Laboratory of Respiratory Viruses and Measles, Oswaldo Cruz Institute, FIOCRUZ	Paola Resende et al
EPI_ISL_729817	Laboratório Central de Saúde Pública do Estado do Rio Grande do Sul (LACEN-RS)	Laboratory of Respiratory Viruses and Measles, Oswaldo Cruz Institute, FIOCRUZ	Paola Resende et al
EPI_ISL_729818	Laboratório Central de Saúde Pública do Estado do Rio Grande do Sul (LACEN-RS)	Laboratory of Respiratory Viruses and Measles, Oswaldo Cruz Institute, FIOCRUZ	Paola Resende et al
EPI_ISL_1533979	Laboratorio Central de Saude Publica do Estado do Rio Grande do Sul (LACEN-RS)	Laboratory of Respiratory Viruses and Measles, Oswaldo Cruz Institute, FIOCRUZ	Paola Resende et al
EPI_ISL_1181524	Laboratorio Central de Saude Publica do Estado do Rio Grande do Sul (LACEN-RS)	Laboratory of Respiratory Viruses and Measles, Oswaldo Cruz Institute, FIOCRUZ	Paola Resende et al
EPI_ISL_1181368	Laboratorio Central de Saude Publica do Estado do Rio Grande do Sul (LACEN-RS)	Laboratory of Respiratory Viruses and Measles, Oswaldo Cruz Institute, FIOCRUZ	Paola Resende et al
EPI_ISL_1181411	Laboratorio Central de Saude Publica do Estado do Rio Grande do Sul (LACEN-RS)	Laboratory of Respiratory Viruses and Measles, Oswaldo Cruz Institute, FIOCRUZ	Paola Resende et al
EPI_ISL_729819	Laboratório Central de Saúde Pública do Estado do Rio Grande do Sul (LACEN-RS)	Laboratory of Respiratory Viruses and Measles, Oswaldo Cruz Institute, FIOCRUZ	Paola Resende et al
EPI_ISL_729820	Laboratório Central de Saúde Pública do Estado do Rio Grande do Sul (LACEN-RS)	Laboratory of Respiratory Viruses and Measles, Oswaldo Cruz Institute, FIOCRUZ	Paola Resende et al

EPI_ISL_729821	Laboratório Central de Saúde Pública do Estado do Rio Grande do Sul (LACEN-RS)	Laboratory of Respiratory Viruses and Measles, Oswaldo Cruz Institute, FIOCRUZ	Paola Resende et al
EPI_ISL_729797	Laboratório Central de Saúde Pública do Estado do Rio Grande do Sul (LACEN-RS)	Laboratory of Respiratory Viruses and Measles, Oswaldo Cruz Institute, FIOCRUZ	Paola Resende et al
EPI_ISL_729822	Laboratório Central de Saúde Pública do Estado do Rio Grande do Sul (LACEN-RS)	Laboratory of Respiratory Viruses and Measles, Oswaldo Cruz Institute, FIOCRUZ	Paola Resende et al
EPI_ISL_729823	Laboratório Central de Saúde Pública do Estado do Rio Grande do Sul (LACEN-RS)	Laboratory of Respiratory Viruses and Measles, Oswaldo Cruz Institute, FIOCRUZ	Paola Resende et al
EPI_ISL_729798	Laboratório Central de Saúde Pública do Estado do Rio Grande do Sul (LACEN-RS)	Laboratory of Respiratory Viruses and Measles, Oswaldo Cruz Institute, FIOCRUZ	Paola Resende et al
EPI_ISL_729824	Laboratório Central de Saúde Pública do Estado do Rio Grande do Sul (LACEN-RS)	Laboratory of Respiratory Viruses and Measles, Oswaldo Cruz Institute, FIOCRUZ	Paola Resende et al
EPI_ISL_729825	Laboratório Central de Saúde Pública do Estado do Rio Grande do Sul (LACEN-RS)	Laboratory of Respiratory Viruses and Measles, Oswaldo Cruz Institute, FIOCRUZ	Paola Resende et al
EPI_ISL_729826	Laboratório Central de Saúde Pública do Estado do Rio Grande do Sul (LACEN-RS)	Laboratory of Respiratory Viruses and Measles, Oswaldo Cruz Institute, FIOCRUZ	Paola Resende et al
EPI_ISL_729827	Laboratório Central de Saúde Pública do Estado do Rio Grande do Sul (LACEN-RS)	Laboratory of Respiratory Viruses and Measles, Oswaldo Cruz Institute, FIOCRUZ	Paola Resende et al
EPI_ISL_729794	Laboratório Central de Saúde Pública do Estado do Rio Grande do Sul (LACEN-RS)	Laboratory of Respiratory Viruses and Measles, Oswaldo Cruz Institute, FIOCRUZ	Paola Resende et al
EPI_ISL_729828	Laboratório Central de Saúde Pública do Estado do Rio Grande do Sul (LACEN-RS)	Laboratory of Respiratory Viruses and Measles, Oswaldo Cruz Institute, FIOCRUZ	Paola Resende et al
EPI_ISL_729829	Laboratório Central de Saúde Pública do Estado do Rio Grande do Sul (LACEN-RS)	Laboratory of Respiratory Viruses and Measles, Oswaldo Cruz Institute, FIOCRUZ	Paola Resende et al
EPI_ISL_729830	Laboratório Central de Saúde Pública do Estado do Rio Grande do Sul (LACEN-RS)	Laboratory of Respiratory Viruses and Measles, Oswaldo Cruz Institute, FIOCRUZ	Paola Resende et al
EPI_ISL_729831	Laboratório Central de Saúde Pública do Estado do Rio Grande do Sul (LACEN-RS)	Laboratory of Respiratory Viruses and Measles, Oswaldo Cruz Institute, FIOCRUZ	Paola Resende et al
EPI_ISL_729832	Laboratório Central de Saúde Pública do Estado do Rio Grande do Sul (LACEN-RS)	Laboratory of Respiratory Viruses and Measles, Oswaldo Cruz Institute, FIOCRUZ	Paola Resende et al
EPI_ISL_729833	Laboratório Central de Saúde Pública do Estado do Rio Grande do Sul (LACEN-RS)	Laboratory of Respiratory Viruses and Measles, Oswaldo Cruz Institute, FIOCRUZ	Paola Resende et al
EPI_ISL_729834	Laboratório Central de Saúde Pública do Estado do Rio Grande do Sul (LACEN-RS)	Laboratory of Respiratory Viruses and Measles, Oswaldo Cruz Institute, FIOCRUZ	Paola Resende et al
EPI_ISL_729835	Laboratório Central de Saúde Pública do Estado do Rio Grande do Sul (LACEN-RS)	Laboratory of Respiratory Viruses and Measles, Oswaldo Cruz Institute, FIOCRUZ	Paola Resende et al
EPI_ISL_729836	Laboratório Central de Saúde Pública do Estado do Rio Grande do Sul (LACEN-RS)	Laboratory of Respiratory Viruses and Measles, Oswaldo Cruz Institute, FIOCRUZ	Paola Resende et al
EPI_ISL_729795	Laboratório Central de Saúde Pública do Estado do Rio Grande do Sul (LACEN-RS)	Laboratory of Respiratory Viruses and Measles, Oswaldo Cruz Institute, FIOCRUZ	Paola Resende et al
EPI_ISL_729796	Laboratório Central de Saúde Pública do Estado do Rio Grande do Sul (LACEN-RS)	Laboratory of Respiratory Viruses and Measles, Oswaldo Cruz Institute, FIOCRUZ	Paola Resende et al
EPI_ISL_729837	Laboratório Central de Saúde Pública do Estado do Rio Grande do Sul (LACEN-RS)	Laboratory of Respiratory Viruses and Measles, Oswaldo Cruz Institute, FIOCRUZ	Paola Resende et al

EPI_ISL_729846	Laboratório Central de Saúde Pública do Estado do Rio Grande do Sul (LACEN-RS)	Laboratory of Respiratory Viruses and Measles, Oswaldo Cruz Institute, FIOCRUZ	Paola Resende et al
EPI_ISL_729838	Laboratório Central de Saúde Pública do Estado do Rio Grande do Sul (LACEN-RS)	Laboratory of Respiratory Viruses and Measles, Oswaldo Cruz Institute, FIOCRUZ	Paola Resende et al
EPI_ISL_729800	Laboratório Central de Saúde Pública do Estado do Rio Grande do Sul (LACEN-RS)	Laboratory of Respiratory Viruses and Measles, Oswaldo Cruz Institute, FIOCRUZ	Paola Resende et al
EPI_ISL_729839	Laboratório Central de Saúde Pública do Estado do Rio Grande do Sul (LACEN-RS)	Laboratory of Respiratory Viruses and Measles, Oswaldo Cruz Institute, FIOCRUZ	Paola Resende et al
EPI_ISL_729840	Laboratório Central de Saúde Pública do Estado do Rio Grande do Sul (LACEN-RS)	Laboratory of Respiratory Viruses and Measles, Oswaldo Cruz Institute, FIOCRUZ	Paola Resende et al
EPI_ISL_1181567	Laboratorio Central de Saude Publica do Estado do Rio Grande do Sul (LACEN-RS)	Laboratory of Respiratory Viruses and Measles, Oswaldo Cruz Institute, FIOCRUZ	Paola Resende et al
EPI_ISL_729841	Laboratório Central de Saúde Pública do Estado do Rio Grande do Sul (LACEN-RS)	Laboratory of Respiratory Viruses and Measles, Oswaldo Cruz Institute, FIOCRUZ	Paola Resende et al
EPI_ISL_729842	Laboratório Central de Saúde Pública do Estado do Rio Grande do Sul (LACEN-RS)	Laboratory of Respiratory Viruses and Measles, Oswaldo Cruz Institute, FIOCRUZ	Paola Resende et al
EPI_ISL_729843	Laboratório Central de Saúde Pública do Estado do Rio Grande do Sul (LACEN-RS)	Laboratory of Respiratory Viruses and Measles, Oswaldo Cruz Institute, FIOCRUZ	Paola Resende et al
EPI_ISL_729847	Laboratório Central de Saúde Pública do Estado do Rio Grande do Sul (LACEN-RS)	Laboratory of Respiratory Viruses and Measles, Oswaldo Cruz Institute, FIOCRUZ	Paola Resende et al
EPI_ISL_1181403	Laboratorio Central de Saude Publica do Estado de Santa Catarina (LACEN-SC)	Laboratory of Respiratory Viruses and Measles, Oswaldo Cruz Institute, FIOCRUZ	Paola Resende et al
EPI_ISL_1534007	Laboratorio Central de Saude Publica do Estado de Santa Catarina (LACEN-SC)	Laboratory of Respiratory Viruses and Measles, Oswaldo Cruz Institute, FIOCRUZ	Paola Resende et al
EPI_ISL_1534003	Laboratorio Central de Saude Publica do Estado de Santa Catarina (LACEN-SC)	Laboratory of Respiratory Viruses and Measles, Oswaldo Cruz Institute, FIOCRUZ	Paola Resende et al
EPI_ISL_1534006	Laboratorio Central de Saude Publica do Estado de Santa Catarina (LACEN-SC)	Laboratory of Respiratory Viruses and Measles, Oswaldo Cruz Institute, FIOCRUZ	Paola Resende et al
EPI_ISL_1534010	Laboratorio Central de Saude Publica do Estado de Santa Catarina (LACEN-SC)	Laboratory of Respiratory Viruses and Measles, Oswaldo Cruz Institute, FIOCRUZ	Paola Resende et al
EPI_ISL_1534008	Laboratorio Central de Saude Publica do Estado de Santa Catarina (LACEN-SC)	Laboratory of Respiratory Viruses and Measles, Oswaldo Cruz Institute, FIOCRUZ	Paola Resende et al
EPI_ISL_1534009	Laboratorio Central de Saude Publica do Estado de Santa Catarina (LACEN-SC)	Laboratory of Respiratory Viruses and Measles, Oswaldo Cruz Institute, FIOCRUZ	Paola Resende et al
EPI_ISL_1534002	Laboratorio Central de Saude Publica do Estado de Santa Catarina (LACEN-SC)	Laboratory of Respiratory Viruses and Measles, Oswaldo Cruz Institute, FIOCRUZ	Paola Resende et al
EPI_ISL_1533994	Laboratorio Central de Saude Publica do Estado de Santa Catarina (LACEN-SC)	Laboratory of Respiratory Viruses and Measles, Oswaldo Cruz Institute, FIOCRUZ	Paola Resende et al
EPI_ISL_1533997	Laboratorio Central de Saude Publica do Estado de Santa Catarina (LACEN-SC)	Laboratory of Respiratory Viruses and Measles, Oswaldo Cruz Institute, FIOCRUZ	Paola Resende et al
EPI_ISL_1533992	Laboratorio Central de Saude Publica do Estado de Santa Catarina (LACEN-SC)	Laboratory of Respiratory Viruses and Measles, Oswaldo Cruz Institute, FIOCRUZ	Paola Resende et al
EPI_ISL_1533990	Laboratorio Central de Saude Publica do Estado de Santa Catarina (LACEN-SC)	Laboratory of Respiratory Viruses and Measles, Oswaldo Cruz Institute, FIOCRUZ	Paola Resende et al

EPI_ISL_1533996	Laboratorio Central de Saude Publica do Estado de Santa Catarina (LACEN-SC)	Laboratory of Respiratory Viruses and Measles, Oswaldo Cruz Institute, FIOCRUZ	Paola Resende et al
EPI_ISL_1533993	Laboratorio Central de Saude Publica do Estado de Santa Catarina (LACEN-SC)	Laboratory of Respiratory Viruses and Measles, Oswaldo Cruz Institute, FIOCRUZ	Paola Resende et al
EPI_ISL_1181402	Laboratory of Respiratory Viruses and Measles, Oswaldo Cruz Institute, FIOCRUZ	Laboratory of Respiratory Viruses and Measles, Oswaldo Cruz Institute, FIOCRUZ	Paola Resende et al
EPI_ISL_1181412	Laboratorio Central de Saude Publica do Estado de Santa Catarina (LACEN-SC)	Laboratory of Respiratory Viruses and Measles, Oswaldo Cruz Institute, FIOCRUZ	Paola Resende et al
EPI_ISL_1181404	Laboratorio Central de Saude Publica do Estado de Santa Catarina (LACEN-SC)	Laboratory of Respiratory Viruses and Measles, Oswaldo Cruz Institute, FIOCRUZ	Paola Resende et al
EPI_ISL_1181453	Laboratorio Central de Saude Publica do Estado de Santa Catarina (LACEN-SC)	Laboratory of Respiratory Viruses and Measles, Oswaldo Cruz Institute, FIOCRUZ	Paola Resende et al
EPI_ISL_427305	Laboratório Central de Saúde Pública do Estado de Santa Catarina (LACEN-SC)	Laboratory of Respiratory Viruses and Measles, Oswaldo Cruz Institute, FIOCRUZ	Paola Resende et al
EPI_ISL_427306	Laboratório Central de Saúde Pública do Estado de Santa Catarina (LACEN-SC)	Laboratory of Respiratory Viruses and Measles, Oswaldo Cruz Institute, FIOCRUZ	Paola Resende et al
EPI_ISL_541370	Laboratório Central de Saúde Pública do Estado de Santa Catarina (LACEN-SC)	Laboratory of Respiratory Viruses and Measles, Oswaldo Cruz Institute, FIOCRUZ	Paola Resende et al
EPI_ISL_541371	Laboratório Central de Saúde Pública do Estado de Santa Catarina (LACEN-SC)	Laboratory of Respiratory Viruses and Measles, Oswaldo Cruz Institute, FIOCRUZ	Paola Resende et al
EPI_ISL_1534004	Laboratorio Central de Saude Publica do Estado de Sergipe (LACEN-SE)	Laboratory of Respiratory Viruses and Measles, Oswaldo Cruz Institute, FIOCRUZ	Paola Resende et al
EPI_ISL_1181405	Laboratorio Central de Saude Publica do Estado de Sergipe (LACEN-SE)	Laboratory of Respiratory Viruses and Measles, Oswaldo Cruz Institute, FIOCRUZ	Paola Resende et al
EPI_ISL_1181409	Laboratorio Central de Saude Publica do Estado de Sergipe (LACEN-SE)	Laboratory of Respiratory Viruses and Measles, Oswaldo Cruz Institute, FIOCRUZ	Paola Resende et al
EPI_ISL_1181406	Laboratorio Central de Saude Publica do Estado de Sergipe (LACEN-SE)	Laboratory of Respiratory Viruses and Measles, Oswaldo Cruz Institute, FIOCRUZ	Paola Resende et al
EPI_ISL_1181407	Laboratorio Central de Saude Publica do Estado de Sergipe (LACEN-SE)	Laboratory of Respiratory Viruses and Measles, Oswaldo Cruz Institute, FIOCRUZ	Paola Resende et al
EPI_ISL_541372	Laboratório Central de Saúde Pública do Estado de Sergipe (LACEN-SE)	Laboratory of Respiratory Viruses and Measles, Oswaldo Cruz Institute, FIOCRUZ	Paola Resende et al
EPI_ISL_541373	Laboratório Central de Saúde Pública do Estado de Sergipe (LACEN-SE)	Laboratory of Respiratory Viruses and Measles, Oswaldo Cruz Institute, FIOCRUZ	Paola Resende et al
EPI_ISL_541374	Laboratório Central de Saúde Pública do Estado de Sergipe (LACEN-SE)	Laboratory of Respiratory Viruses and Measles, Oswaldo Cruz Institute, FIOCRUZ	Paola Resende et al
EPI_ISL_541375	Laboratório Central de Saúde Pública do Estado de Sergipe (LACEN-SE)	Laboratory of Respiratory Viruses and Measles, Oswaldo Cruz Institute, FIOCRUZ	Paola Resende et al
EPI_ISL_541376	Laboratório Central de Saúde Pública do Estado de Sergipe (LACEN-SE)	Laboratory of Respiratory Viruses and Measles, Oswaldo Cruz Institute, FIOCRUZ	Paola Resende et al
EPI_ISL_541377	Laboratório Central de Saúde Pública do Estado de Sergipe (LACEN-SE)	Laboratory of Respiratory Viruses and Measles, Oswaldo Cruz Institute, FIOCRUZ	Paola Resende et al
EPI_ISL_541378	Laboratório Central de Saúde Pública do Estado de Sergipe (LACEN-SE)	Laboratory of Respiratory Viruses and Measles, Oswaldo Cruz Institute, FIOCRUZ	Paola Resende et al

EPI_ISL_541379	Laboratório Central de Saúde Pública do Estado de Sergipe (LACEN-SE)	Laboratory of Respiratory Viruses and Measles, Oswaldo Cruz Institute, FIOCRUZ	Paola Resende et al
EPI_ISL_541380	Laboratório Central de Saúde Pública do Estado de Sergipe (LACEN-SE)	Laboratory of Respiratory Viruses and Measles, Oswaldo Cruz Institute, FIOCRUZ	Paola Resende et al
EPI_ISL_1181590	Laboratorio Central de Saude Publica do Estado de Sergipe (LACEN-SE)	Laboratory of Respiratory Viruses and Measles, Oswaldo Cruz Institute, FIOCRUZ	Paola Resende et al
EPI_ISL_541381	Laboratório Central de Saúde Pública do Estado de Sergipe (LACEN-SE)	Laboratory of Respiratory Viruses and Measles, Oswaldo Cruz Institute, FIOCRUZ	Paola Resende et al
EPI_ISL_541382	Laboratório Central de Saúde Pública do Estado de Sergipe (LACEN-SE)	Laboratory of Respiratory Viruses and Measles, Oswaldo Cruz Institute, FIOCRUZ	Paola Resende et al
EPI_ISL_541383	Laboratório Central de Saúde Pública do Estado de Sergipe (LACEN-SE)	Laboratory of Respiratory Viruses and Measles, Oswaldo Cruz Institute, FIOCRUZ	Paola Resende et al
EPI_ISL_541384	Laboratório Central de Saúde Pública do Estado de Sergipe (LACEN-SE)	Laboratory of Respiratory Viruses and Measles, Oswaldo Cruz Institute, FIOCRUZ	Paola Resende et al
EPI_ISL_541385	Laboratório Central de Saúde Pública do Estado de Sergipe (LACEN-SE)	Laboratory of Respiratory Viruses and Measles, Oswaldo Cruz Institute, FIOCRUZ	Paola Resende et al
EPI_ISL_541386	Laboratório Central de Saúde Pública do Estado de Sergipe (LACEN-SE)	Laboratory of Respiratory Viruses and Measles, Oswaldo Cruz Institute, FIOCRUZ	Paola Resende et al
EPI_ISL_541387	Laboratório Central de Saúde Pública do Estado de Sergipe (LACEN-SE)	Laboratory of Respiratory Viruses and Measles, Oswaldo Cruz Institute, FIOCRUZ	Paola Resende et al
EPI_ISL_541388	Laboratório Central de Saúde Pública do Estado de Sergipe (LACEN-SE)	Laboratory of Respiratory Viruses and Measles, Oswaldo Cruz Institute, FIOCRUZ	Paola Resende et al
EPI_ISL_541389	Laboratório Central de Saúde Pública do Estado de Sergipe (LACEN-SE)	Laboratory of Respiratory Viruses and Measles, Oswaldo Cruz Institute, FIOCRUZ	Paola Resende et al
EPI_ISL_541390	Laboratório Central de Saúde Pública do Estado de Sergipe (LACEN-SE)	Laboratory of Respiratory Viruses and Measles, Oswaldo Cruz Institute, FIOCRUZ	Paola Resende et al
EPI_ISL_541391	Laboratório Central de Saúde Pública do Estado de Sergipe (LACEN-SE)	Laboratory of Respiratory Viruses and Measles, Oswaldo Cruz Institute, FIOCRUZ	Paola Resende et al
EPI_ISL_541392	Laboratório Central de Saúde Pública do Estado de Sergipe (LACEN-SE)	Laboratory of Respiratory Viruses and Measles, Oswaldo Cruz Institute, FIOCRUZ	Paola Resende et al
EPI_ISL_1181595	Laboratorio Central de Saude Publica do Estado de Sergipe (LACEN-SE)	Laboratory of Respiratory Viruses and Measles, Oswaldo Cruz Institute, FIOCRUZ	Paola Resende et al
EPI_ISL_541393	Laboratório Central de Saúde Pública do Estado de Sergipe (LACEN-SE)	Laboratory of Respiratory Viruses and Measles, Oswaldo Cruz Institute, FIOCRUZ	Paola Resende et al
EPI_ISL_541394	Laboratório Central de Saúde Pública do Estado de Sergipe (LACEN-SE)	Laboratory of Respiratory Viruses and Measles, Oswaldo Cruz Institute, FIOCRUZ	Paola Resende et al
EPI_ISL_541395	Laboratório Central de Saúde Pública do Estado de Sergipe (LACEN-SE)	Laboratory of Respiratory Viruses and Measles, Oswaldo Cruz Institute, FIOCRUZ	Paola Resende et al
EPI_ISL_541396	Laboratório Central de Saúde Pública do Estado de Sergipe (LACEN-SE)	Laboratory of Respiratory Viruses and Measles, Oswaldo Cruz Institute, FIOCRUZ	Paola Resende et al
EPI_ISL_1533988	Laboratorio Nacional de Salud	Laboratory of Respiratory Viruses and Measles, Oswaldo Cruz Institute, FIOCRUZ	Paola Resende et al
EPI_ISL_1533989	Laboratorio Nacional de Salud	Laboratory of Respiratory Viruses and Measles, Oswaldo Cruz Institute, FIOCRUZ	Paola Resende et al

EPI_ISL_837579	Laboratorio Nacional de Salud	Laboratory of Respiratory Viruses and Measles, Oswaldo Cruz Institute, FIOCRUZ	Paola Resende et al
EPI_ISL_837580	Laboratorio Nacional de Salud	Laboratory of Respiratory Viruses and Measles, Oswaldo Cruz Institute, FIOCRUZ	Paola Resende et al
EPI_ISL_837581	Laboratorio Nacional de Salud	Laboratory of Respiratory Viruses and Measles, Oswaldo Cruz Institute, FIOCRUZ	Paola Resende et al
EPI_ISL_837582	Laboratorio Nacional de Salud	Laboratory of Respiratory Viruses and Measles, Oswaldo Cruz Institute, FIOCRUZ	Paola Resende et al
EPI_ISL_837583	Laboratorio Nacional de Salud	Laboratory of Respiratory Viruses and Measles, Oswaldo Cruz Institute, FIOCRUZ	Paola Resende et al
EPI_ISL_837551	Laboratorio Nacional de Salud	Laboratory of Respiratory Viruses and Measles, Oswaldo Cruz Institute, FIOCRUZ	Paola Resende et al
EPI_ISL_837584	Laboratorio Nacional de Salud	Laboratory of Respiratory Viruses and Measles, Oswaldo Cruz Institute, FIOCRUZ	Paola Resende et al
EPI_ISL_837586	Laboratorio Nacional de Salud	Laboratory of Respiratory Viruses and Measles, Oswaldo Cruz Institute, FIOCRUZ	Paola Resende et al
EPI_ISL_837587	Laboratorio Nacional de Salud	Laboratory of Respiratory Viruses and Measles, Oswaldo Cruz Institute, FIOCRUZ	Paola Resende et al
EPI_ISL_837588	Laboratorio Nacional de Salud	Laboratory of Respiratory Viruses and Measles, Oswaldo Cruz Institute, FIOCRUZ	Paola Resende et al
EPI_ISL_837589	Laboratorio Nacional de Salud	Laboratory of Respiratory Viruses and Measles, Oswaldo Cruz Institute, FIOCRUZ	Paola Resende et al
EPI_ISL_837592	Laboratorio Nacional de Salud	Laboratory of Respiratory Viruses and Measles, Oswaldo Cruz Institute, FIOCRUZ	Paola Resende et al
EPI_ISL_837593	Laboratorio Nacional de Salud	Laboratory of Respiratory Viruses and Measles, Oswaldo Cruz Institute, FIOCRUZ	Paola Resende et al
EPI_ISL_837595	Laboratorio Nacional de Salud	Laboratory of Respiratory Viruses and Measles, Oswaldo Cruz Institute, FIOCRUZ	Paola Resende et al
EPI_ISL_837596	Laboratorio Nacional de Salud	Laboratory of Respiratory Viruses and Measles, Oswaldo Cruz Institute, FIOCRUZ	Paola Resende et al
EPI_ISL_837552	Laboratorio Nacional de Salud	Laboratory of Respiratory Viruses and Measles, Oswaldo Cruz Institute, FIOCRUZ	Paola Resende et al
EPI_ISL_1533981	Laboratorio Nacional de Salud	Laboratory of Respiratory Viruses and Measles, Oswaldo Cruz Institute, FIOCRUZ	Paola Resende et al
EPI_ISL_1533982	Laboratorio Nacional de Salud	Laboratory of Respiratory Viruses and Measles, Oswaldo Cruz Institute, FIOCRUZ	Paola Resende et al
EPI_ISL_1533984	Laboratorio Nacional de Salud	Laboratory of Respiratory Viruses and Measles, Oswaldo Cruz Institute, FIOCRUZ	Paola Resende et al
EPI_ISL_1533985	Laboratorio Nacional de Salud	Laboratory of Respiratory Viruses and Measles, Oswaldo Cruz Institute, FIOCRUZ	Paola Resende et al
EPI_ISL_1533987	Laboratorio Nacional de Salud	Laboratory of Respiratory Viruses and Measles, Oswaldo Cruz Institute, FIOCRUZ	Paola Resende et al
EPI_ISL_1340751	Departamento de Virologia, Laboratorio Central de Salud Pública, Avenida Venezuela y Teniente Escurra, Asunción, Paraguay	Laboratory of Respiratory Viruses and Measles, Oswaldo Cruz Institute, FIOCRUZ	Paola Resende et al

EPI_ISL_1340750	Departamento de Virologia, Laboratorio Central de Salud Pública, Avenida Venezuela y Teniente Escurra, Asunción, Paraguay	Laboratory of Respiratory Viruses and Measles, Oswaldo Cruz Institute, FIOCRUZ	Paola Resende et al
EPI_ISL_1340764	Departamento de Virologia, Laboratorio Central de Salud Pública, Avenida Venezuela y Teniente Escurra, Asunción, Paraguay	Laboratory of Respiratory Viruses and Measles, Oswaldo Cruz Institute, FIOCRUZ	Paola Resende et al
EPI_ISL_1340762	Departamento de Virologia, Laboratorio Central de Salud Pública, Avenida Venezuela y Teniente Escurra, Asunción, Paraguay	Laboratory of Respiratory Viruses and Measles, Oswaldo Cruz Institute, FIOCRUZ	Paola Resende et al
EPI_ISL_1340763	Departamento de Virologia, Laboratorio Central de Salud Pública, Avenida Venezuela y Teniente Escurra, Asunción, Paraguay	Laboratory of Respiratory Viruses and Measles, Oswaldo Cruz Institute, FIOCRUZ	Paola Resende et al
EPI_ISL_1340761	Departamento de Virologia, Laboratorio Central de Salud Pública, Avenida Venezuela y Teniente Escurra, Asunción, Paraguay	Laboratory of Respiratory Viruses and Measles, Oswaldo Cruz Institute, FIOCRUZ	Paola Resende et al
EPI_ISL_1340760	Departamento de Virologia, Laboratorio Central de Salud Pública, Avenida Venezuela y Teniente Escurra, Asunción, Paraguay	Laboratory of Respiratory Viruses and Measles, Oswaldo Cruz Institute, FIOCRUZ	Paola Resende et al
EPI_ISL_1340759	Departamento de Virologia, Laboratorio Central de Salud Pública, Avenida Venezuela y Teniente Escurra, Asunción, Paraguay	Laboratory of Respiratory Viruses and Measles, Oswaldo Cruz Institute, FIOCRUZ	Paola Resende et al
EPI_ISL_1340758	Departamento de Virologia, Laboratorio Central de Salud Pública, Avenida Venezuela y Teniente Escurra, Asunción, Paraguay	Laboratory of Respiratory Viruses and Measles, Oswaldo Cruz Institute, FIOCRUZ	Paola Resende et al
EPI_ISL_1340756	Departamento de Virologia, Laboratorio Central de Salud Pública, Avenida Venezuela y Teniente Escurra, Asunción, Paraguay	Laboratory of Respiratory Viruses and Measles, Oswaldo Cruz Institute, FIOCRUZ	Paola Resende et al
EPI_ISL_1340753	Departamento de Virologia, Laboratorio Central de Salud Pública, Avenida Venezuela y Teniente Escurra, Asunción, Paraguay	Laboratory of Respiratory Viruses and Measles, Oswaldo Cruz Institute, FIOCRUZ	Paola Resende et al
EPI_ISL_1340754	Departamento de Virologia, Laboratorio Central de Salud Pública, Avenida Venezuela y Teniente Escurra, Asunción, Paraguay	Laboratory of Respiratory Viruses and Measles, Oswaldo Cruz Institute, FIOCRUZ	Paola Resende et al
EPI_ISL_1340755	Departamento de Virologia, Laboratorio Central de Salud Pública, Avenida Venezuela y Teniente Escurra, Asunción, Paraguay	Laboratory of Respiratory Viruses and Measles, Oswaldo Cruz Institute, FIOCRUZ	Paola Resende et al
EPI_ISL_1340752	Departamento de Virologia, Laboratorio Central de Salud Pública, Avenida Venezuela y Teniente Escurra, Asunción, Paraguay	Laboratory of Respiratory Viruses and Measles, Oswaldo Cruz Institute, FIOCRUZ	Paola Resende et al
EPI_ISL_875687	Department of Infectious Diseases, Istituto Superiore di Sanità, Rome, Italy; UCO Igiene e Sanità Pubblica, ASUGI, Trieste, Italy; Laboratorio di Genomica ed Epigenomica sistema Argo, Area SciencePark, Trieste, Italy; Laboratorio di Virologia Molecolare, ICGEB, Trieste, Italy	Istituto Superiore di Sanità (ISS)	Paola Stefanelli et al
EPI_ISL_412974	Department of Infectious Diseases, Istituto Superiore di Sanità, Rome, Italy	Virology Laboratory, Scientific Department, Army Medical Center	Paola Stefanelli et al
EPI_ISL_1443188	CHWAPI - SITE NOTRE DAME	Institut de Pathologie et Genetique (IPG)	Pascale Hilbert et al
EPI_ISL_1451467	CHWAPI - SITE NOTRE DAME	Institut de Pathologie et Genetique (IPG)	Pascale Hilbert et al
EPI_ISL_1451473	CHWAPI - SITE NOTRE DAME	Institut de Pathologie et Genetique (IPG)	Pascale Hilbert et al
EPI_ISL_1299241	CHWAPI - SITE NOTRE DAME	Institut de Pathologie et Genetique (IPG)	Pascale Hilbert et al
EPI_ISL_596709	PathWest Laboratory Medicine WA	PathWest Laboratory Medicine WA Microbial Surveillance Unit	PathWest Laboratory Medicine WA Microbia
EPI_ISL_596764	PathWest Laboratory Medicine WA	PathWest Laboratory Medicine WA Microbial Surveillance Unit	PathWest Laboratory Medicine WA Microbia
EPI_ISL_596766	PathWest Laboratory Medicine WA	PathWest Laboratory Medicine WA Microbial Surveillance Unit	PathWest Laboratory Medicine WA Microbia
EPI_ISL_596777	PathWest Laboratory Medicine WA	PathWest Laboratory Medicine WA Microbial Surveillance Unit	PathWest Laboratory Medicine WA Microbia
EPI_ISL_605879	PathWest Laboratory Medicine WA	PathWest Laboratory Medicine WA Microbial Surveillance Unit	PathWest Laboratory Medicine WA Microbia

al Surveillance Unit et al
al Surveillance Unit et al
al Surveillance Unit et al
al Surveillance Unit et al
al Surveillance Unit et al

EPI_ISL_672653	PathWest Laboratory Medicine WA	PathWest Laboratory Medicine WA Microbial Surveillance Unit	PathWest Laboratory Medicine WA Microbia
EPI_ISL_708745	PathWest Laboratory Medicine WA	PathWest Laboratory Medicine WA Microbial Surveillance Unit	PathWest Laboratory Medicine WA Microbia
EPI_ISL_794715	PathWest Laboratory Medicine WA	PathWest Laboratory Medicine WA Microbial Surveillance Unit	PathWest Laboratory Medicine WA Microbia
EPI_ISL_794726	PathWest Laboratory Medicine WA	PathWest Laboratory Medicine WA Microbial Surveillance Unit	PathWest Laboratory Medicine WA Microbia
EPI_ISL_794672	PathWest Laboratory Medicine WA	PathWest Laboratory Medicine WA Microbial Surveillance Unit	PathWest Laboratory Medicine WA Microbia
EPI_ISL_933795	PathWest Laboratory Medicine WA	PathWest Laboratory Medicine WA Microbial Surveillance Unit	PathWest Laboratory Medicine WA Microbia
EPI_ISL_1184507	PathWest Laboratory Medicine WA	PathWest Laboratory Medicine WA Microbial Surveillance Unit	PathWest Laboratory Medicine WA Microbia
EPI_ISL_1416322	PathWest Laboratory Medicine WA	PathWest Laboratory Medicine WA Microbial Surveillance Unit	PathWest Laboratory Medicine WA Microbia
EPI_ISL_1508996	PathWest Laboratory Medicine WA	PathWest Laboratory Medicine WA Microbial Surveillance Unit	PathWest Laboratory Medicine WA Microbia
EPI_ISL_1620802	PathWest Laboratory Medicine WA	PathWest Laboratory Medicine WA Microbial Surveillance Unit	PathWest Laboratory Medicine WA Microbia
EPI_ISL_1633680	PathWest Laboratory Medicine WA	PathWest Laboratory Medicine WA Microbial Surveillance Unit	PathWest Laboratory Medicine WA Microbia
EPI_ISL_1669122	PathWest Laboratory Medicine WA	PathWest Laboratory Medicine WA Microbial Surveillance Unit	PathWest Laboratory Medicine WA Microbia
EPI_ISL_1580504	Laboratório de Biologia Molecular do Hospital das Clínicas da Faculdade de Medicina de Botucatu/SP	Laboratórios de Genômica Funcional (FCA/UNESP) e Biologia Molecular (FMB-HC/UNESP) - Rede de Vigilância Genômica (Vigenômica)/UNESP	Patrícia Akemi Assato et al
EPI_ISL_1580502	Laboratório de Biologia Molecular do Hospital das Clínicas da Faculdade de Medicina de Botucatu/SP	Laboratórios de Genômica Funcional (FCA/UNESP) e Biologia Molecular (FMB-HC/UNESP) - Rede de Vigilância Genômica (Vigenômica)/UNESP	Patrícia Akemi Assato et al
EPI_ISL_681887	National Virus Reference Laboratory	Irish Coronavirus Sequencing Consortium - Teagasc Moorepark	Paul Cotter et al
EPI_ISL_1324139	UW Virology Lab	UW Virology Lab	Pavitra Roychoudhury et al
EPI_ISL_1324149	UW Virology Lab	UW Virology Lab	Pavitra Roychoudhury et al
EPI_ISL_1324142	UW Virology Lab	UW Virology Lab	Pavitra Roychoudhury et al
EPI_ISL_1324145	UW Virology Lab	UW Virology Lab	Pavitra Roychoudhury et al
EPI_ISL_1324137	UW Virology Lab	UW Virology Lab	Pavitra Roychoudhury et al
EPI_ISL_1324140	UW Virology Lab	UW Virology Lab	Pavitra Roychoudhury et al
EPI_ISL_1324138	UW Virology Lab	UW Virology Lab	Pavitra Roychoudhury et al
EPI_ISL_1324135	UW Virology Lab	UW Virology Lab	Pavitra Roychoudhury et al
EPI_ISL_1324144	UW Virology Lab	UW Virology Lab	Pavitra Roychoudhury et al
EPI_ISL_538512	Balai Penelitian dan Pengembangan Biomedis Papua	National Institute of Health Research and Development	Pawestri et al
EPI_ISL_910075	CSIR-Centre for Cellular and Molecular Biology	CSIR-Centre for Cellular and Molecular Biology	Payel Mukherjee et al
EPI_ISL_910082	CSIR-Centre for Cellular and Molecular Biology	CSIR-Centre for Cellular and Molecular Biology	Payel Mukherjee et al
EPI_ISL_910184	CSIR-Centre for Cellular and Molecular Biology	CSIR-Centre for Cellular and Molecular Biology	Payel Mukherjee et al
EPI_ISL_910186	CSIR-Centre for Cellular and Molecular Biology	CSIR-Centre for Cellular and Molecular Biology	Payel Mukherjee et al
EPI_ISL_910273	CSIR-Centre for Cellular and Molecular Biology	CSIR-Centre for Cellular and Molecular Biology	Payel Mukherjee et al

al Surveillance Unit et al
al Surveillance Unit et al
al Surveillance Unit et al
al Surveillance Unit et al
al Surveillance Unit et al
al Surveillance Unit et al
al Surveillance Unit et al
al Surveillance Unit et al
al Surveillance Unit et al
al Surveillance Unit et al
al Surveillance Unit et al
al Surveillance Unit et al

EPI_ISL_910276	CSIR-Centre for Cellular and Molecular Biology	CSIR-Centre for Cellular and Molecular Biology	Payel Mukherjee et al
EPI_ISL_1380090	CSIR-Centre for Cellular and Molecular Biology	CSIR-Centre for Cellular and Molecular Biology	Payel Mukherjee et al
EPI_ISL_1380102	CSIR-Centre for Cellular and Molecular Biology	CSIR-Centre for Cellular and Molecular Biology	Payel Mukherjee et al
EPI_ISL_1380150	CSIR-Centre for Cellular and Molecular Biology	CSIR-Centre for Cellular and Molecular Biology	Payel Mukherjee et al
EPI_ISL_1380167	CSIR-Centre for Cellular and Molecular Biology	CSIR-Centre for Cellular and Molecular Biology	Payel Mukherjee et al
EPI_ISL_1380196	CSIR-Centre for Cellular and Molecular Biology	CSIR-Centre for Cellular and Molecular Biology	Payel Mukherjee et al
EPI_ISL_1380299	CSIR-Centre for Cellular and Molecular Biology	CSIR-Centre for Cellular and Molecular Biology	Payel Mukherjee et al
EPI_ISL_1380470	CSIR-Centre for Cellular and Molecular Biology	CSIR-Centre for Cellular and Molecular Biology	Payel Mukherjee et al
EPI_ISL_539720	CSIR-Centre for Cellular and Molecular Biology	CSIR-Centre for Cellular and Molecular Biology	Payel Mukherjee et al
EPI_ISL_1260835	BSL-3 Lab, National Institute for Viral Disease Control and Prevention, Chinese Center for Disease Control and Prevention	BSL-3 Lab, National Institute for Viral Disease Control and Prevention, Chinese Center for Disease Control and Prevention	Peipei Liu et al
EPI_ISL_430737	Chinese PLA Institute for Disease Control and Prevention	Chinese PLA Institute for Disease Control and Prevention	Peng Li et al
EPI_ISL_815257	Centogene	Centogene	Peter Bauer et al
EPI_ISL_815398	Centogene	Centogene	Peter Bauer et al
EPI_ISL_815256	Centogene	Centogene	Peter Bauer et al
EPI_ISL_815255	Centogene	Centogene	Peter Bauer et al
EPI_ISL_815390	Centogene	Centogene	Peter Bauer et al
EPI_ISL_435410	Virological Research Group, Szentágothai Research Centre	Bioinformatics Research Group, Szentágothai Research Centre	Péter Urbán et al
EPI_ISL_435416	Virological Research Group, Szentágothai Research Centre	Bioinformatics Research Group, Szentágothai Research Centre	Péter Urbán et al
EPI_ISL_435412	Virological Research Group, Szentágothai Research Centre	Bioinformatics Research Group, Szentágothai Research Centre	Péter Urbán et al
EPI_ISL_435415	Virological Research Group, Szentágothai Research Centre	Bioinformatics Research Group, Szentágothai Research Centre	Péter Urbán et al
EPI_ISL_1027843	Laboratory Corporation of America	Respiratory Viruses Branch, Division of Viral Diseases, Centers for Disease Control and Prevention	Peter W. Cook et al
EPI_ISL_1252981	Quest Diagnostics Incorporated	Centers for Disease Control and Prevention Division of Viral Diseases, Pathogen Discovery	Peter W. Cook et al
EPI_ISL_1268990	Helix/Illumina	Centers for Disease Control and Prevention Division of Viral Diseases, Pathogen Discovery	Peter W. Cook et al
EPI_ISL_1267799	Helix/Illumina	Centers for Disease Control and Prevention Division of Viral Diseases, Pathogen Discovery	Peter W. Cook et al
EPI_ISL_1088103	Quest Diagnostics Incorporated	Respiratory Viruses Branch, Division of Viral Diseases, Centers for Disease Control and Prevention	Peter W. Cook et al
EPI_ISL_889336	The University Hospital Brno	Institute of Applied Biotechnologies a.s.	Petr Klempt et al
EPI_ISL_889346	The University Hospital Brno	Institute of Applied Biotechnologies a.s.	Petr Klempt et al
EPI_ISL_889349	The University Hospital Brno	Institute of Applied Biotechnologies a.s.	Petr Klempt et al

EPI_ISL_889350	The University Hospital Brno	Institute of Applied Biotechnologies a.s.	Petr Klempt et al
EPI_ISL_889351	The University Hospital Brno	Institute of Applied Biotechnologies a.s.	Petr Klempt et al
EPI_ISL_889371	Olomouc University Hospital	Institute of Applied Biotechnologies a.s.	Petr Klempt et al
EPI_ISL_889359	Motol University Hospital	Institute of Applied Biotechnologies a.s.	Petr Klempt et al
EPI_ISL_889362	Motol University Hospital	Institute of Applied Biotechnologies a.s.	Petr Klempt et al
EPI_ISL_889428	Olomouc University Hospital	Institute of Applied Biotechnologies a.s.	Petr Klempt et al
EPI_ISL_889347	The University Hospital Brno	Institute of Applied Biotechnologies a.s.	Petr Klempt et al
EPI_ISL_889360	Motol University Hospital	Institute of Applied Biotechnologies a.s.	Petr Klempt et al
EPI_ISL_1588739	Bioptická laboratoř s.r.o.	Bioptická laboratoř s.r.o.	Petr Šteiner et al
EPI_ISL_1588734	Bioptická laboratoř s.r.o.	Bioptická laboratoř s.r.o.	Petr Šteiner et al
EPI_ISL_1588736	Bioptická laboratoř s.r.o.	Bioptická laboratoř s.r.o.	Petr Šteiner et al
EPI_ISL_1588735	Bioptická laboratoř s.r.o.	Bioptická laboratoř s.r.o.	Petr Šteiner et al
EPI_ISL_1299856	Unit of lab surveillance of viral emerging diseases, National Lab of Influenza	Respiratory Virus Unit, National Infection Service, Public Health England	PHE Covid Sequencing Team et al
EPI_ISL_1299858	Unit of lab surveillance of viral emerging diseases, National Lab of Influenza	Respiratory Virus Unit, National Infection Service, Public Health England	PHE Covid Sequencing Team et al
EPI_ISL_1299861	Unit of lab surveillance of viral emerging diseases, National Lab of Influenza	Respiratory Virus Unit, National Infection Service, Public Health England	PHE Covid Sequencing Team et al
EPI_ISL_1299862	Unit of lab surveillance of viral emerging diseases, National Lab of Influenza	Respiratory Virus Unit, National Infection Service, Public Health England	PHE Covid Sequencing Team et al
EPI_ISL_1299863	Unit of lab surveillance of viral emerging diseases, National Lab of Influenza	Respiratory Virus Unit, National Infection Service, Public Health England	PHE Covid Sequencing Team et al
EPI_ISL_1299864	Unit of lab surveillance of viral emerging diseases, National Lab of Influenza	Respiratory Virus Unit, National Infection Service, Public Health England	PHE Covid Sequencing Team et al
EPI_ISL_1299867	Unit of lab surveillance of viral emerging diseases, National Lab of Influenza	Respiratory Virus Unit, National Infection Service, Public Health England	PHE Covid Sequencing Team et al
EPI_ISL_1299871	Unit of lab surveillance of viral emerging diseases, National Lab of Influenza	Respiratory Virus Unit, National Infection Service, Public Health England	PHE Covid Sequencing Team et al
EPI_ISL_1299873	Unit of lab surveillance of viral emerging diseases, National Lab of Influenza	Respiratory Virus Unit, National Infection Service, Public Health England	PHE Covid Sequencing Team et al
EPI_ISL_1299878	Unit of lab surveillance of viral emerging diseases, National Lab of Influenza	Respiratory Virus Unit, National Infection Service, Public Health England	PHE Covid Sequencing Team et al
EPI_ISL_1299882	Unit of lab surveillance of viral emerging diseases, National Lab of Influenza	Respiratory Virus Unit, National Infection Service, Public Health England	PHE Covid Sequencing Team et al
EPI_ISL_1299884	Unit of lab surveillance of viral emerging diseases, National Lab of Influenza	Respiratory Virus Unit, National Infection Service, Public Health England	PHE Covid Sequencing Team et al
EPI_ISL_780385	Bermuda Government Molecular Diagnostics Laboratory (MDL)	Respiratory Virus Unit, National Infection Service, Public Health England	PHE Covid Sequencing Team et al
EPI_ISL_780386	Bermuda Government Molecular Diagnostics Laboratory (MDL)	Respiratory Virus Unit, National Infection Service, Public Health England	PHE Covid Sequencing Team et al
EPI_ISL_780392	Bermuda Government Molecular Diagnostics Laboratory (MDL)	Respiratory Virus Unit, National Infection Service, Public Health England	PHE Covid Sequencing Team et al
EPI_ISL_780393	Bermuda Government Molecular Diagnostics Laboratory (MDL)	Respiratory Virus Unit, National Infection Service, Public Health England	PHE Covid Sequencing Team et al
EPI_ISL_780384	Bermuda Government Molecular Diagnostics Laboratory (MDL)	Respiratory Virus Unit, National Infection Service, Public Health England	PHE Covid Sequencing Team et al
EPI_ISL_780397	Bermuda Government Molecular Diagnostics Laboratory (MDL)	Respiratory Virus Unit, National Infection Service, Public Health England	PHE Covid Sequencing Team et al

EPI_ISL_780398	Bermuda Government Molecular Diagnostics Laboratory (MDL)	Respiratory Virus Unit, National Infection Service, Public Health England	PHE Covid Sequencing Team et al
EPI_ISL_780402	Bermuda Government Molecular Diagnostics Laboratory (MDL)	Respiratory Virus Unit, National Infection Service, Public Health England	PHE Covid Sequencing Team et al
EPI_ISL_780403	Bermuda Government Molecular Diagnostics Laboratory (MDL)	Respiratory Virus Unit, National Infection Service, Public Health England	PHE Covid Sequencing Team et al
EPI_ISL_780383	Bermuda Government Molecular Diagnostics Laboratory (MDL)	Respiratory Virus Unit, National Infection Service, Public Health England	PHE Covid Sequencing Team et al
EPI_ISL_780404	Bermuda Government Molecular Diagnostics Laboratory (MDL)	Respiratory Virus Unit, National Infection Service, Public Health England	PHE Covid Sequencing Team et al
EPI_ISL_780405	Bermuda Government Molecular Diagnostics Laboratory (MDL)	Respiratory Virus Unit, National Infection Service, Public Health England	PHE Covid Sequencing Team et al
EPI_ISL_780406	Bermuda Government Molecular Diagnostics Laboratory (MDL)	Respiratory Virus Unit, National Infection Service, Public Health England	PHE Covid Sequencing Team et al
EPI_ISL_780407	Bermuda Government Molecular Diagnostics Laboratory (MDL)	Respiratory Virus Unit, National Infection Service, Public Health England	PHE Covid Sequencing Team et al
EPI_ISL_780408	Bermuda Government Molecular Diagnostics Laboratory (MDL)	Respiratory Virus Unit, National Infection Service, Public Health England	PHE Covid Sequencing Team et al
EPI_ISL_780409	Bermuda Government Molecular Diagnostics Laboratory (MDL)	Respiratory Virus Unit, National Infection Service, Public Health England	PHE Covid Sequencing Team et al
EPI_ISL_780410	Bermuda Government Molecular Diagnostics Laboratory (MDL)	Respiratory Virus Unit, National Infection Service, Public Health England	PHE Covid Sequencing Team et al
EPI_ISL_464189	Respiratory Virus Unit, Microbiology Services Colindale, Public Health England	Respiratory Virus Unit, Microbiology Services Colindale, Public Health England	PHE Covid Sequencing Team et al
EPI_ISL_464212	Respiratory Virus Unit, Microbiology Services Colindale, Public Health England	Respiratory Virus Unit, Microbiology Services Colindale, Public Health England	PHE Covid Sequencing Team et al
EPI_ISL_464247	Respiratory Virus Unit, Microbiology Services Colindale, Public Health England	Respiratory Virus Unit, Microbiology Services Colindale, Public Health England	PHE Covid Sequencing Team et al
EPI_ISL_466061	Respiratory Virus Unit, Microbiology Services Colindale, Public Health England	Respiratory Virus Unit, Microbiology Services Colindale, Public Health England	PHE Covid Sequencing Team et al
EPI_ISL_465195	Respiratory Virus Unit, Microbiology Services Colindale, Public Health England	Respiratory Virus Unit, Microbiology Services Colindale, Public Health England	PHE Covid Sequencing Team et al
EPI_ISL_547433	Microbiology, Department of Pathology, St. Bernard's Hospital, Gibraltar Health Authority	Respiratory Virus Unit, Microbiology Services Colindale, Public Health England	PHE Covid Sequencing Team et al
EPI_ISL_547434	Microbiology, Department of Pathology, St. Bernard's Hospital, Gibraltar Health Authority	Respiratory Virus Unit, Microbiology Services Colindale, Public Health England	PHE Covid Sequencing Team et al
EPI_ISL_547435	Microbiology, Department of Pathology, St. Bernard's Hospital, Gibraltar Health Authority	Respiratory Virus Unit, Microbiology Services Colindale, Public Health England	PHE Covid Sequencing Team et al
EPI_ISL_547437	Microbiology, Department of Pathology, St. Bernard's Hospital, Gibraltar Health Authority	Respiratory Virus Unit, Microbiology Services Colindale, Public Health England	PHE Covid Sequencing Team et al
EPI_ISL_487433	Queen Astrid Military Hospital	Institute of Tropical Medicine	Philippe Selhorst et al
EPI_ISL_487434	Queen Astrid Military Hospital	Institute of Tropical Medicine	Philippe Selhorst et al
EPI_ISL_487435	Queen Astrid Military Hospital	Institute of Tropical Medicine	Philippe Selhorst et al
EPI_ISL_487436	Queen Astrid Military Hospital	Institute of Tropical Medicine	Philippe Selhorst et al
EPI_ISL_877765	UCO Igiene e Sanità Pubblica, ASUGI, Trieste, Italy; Laboratorio di Virologia Molecolare, ICGEB, Trieste, Italy	Laboratorio di Genomica ed Epigenomica sistema Argo, Area SciencePark, Trieste, Italy;	Pierlanfranco D'Agaro et al
EPI_ISL_450196	bumrungrad international hospital	National Institute of Health. Department of medical Sciences, Ministry of Public Health, Thailand	Pilailuk et al

EPI_ISL_447920	n/a	National Institute of Health. Department of medical Sciences, Ministry of Public Health, Thailand	Pilailuk et al
EPI_ISL_447913	n/a	National Institute of Health. Department of medical Sciences, Ministry of Public Health, Thailand	Pilailuk et al
EPI_ISL_447918	n/a	National Institute of Health. Department of medical Sciences, Ministry of Public Health, Thailand	Pilailuk et al
EPI_ISL_434695	unknown	National Institute of Health. Department of medical Sciences, Ministry of Public Health, Thailand	Pilailuk et al
EPI_ISL_434700	Ramkhamhaeng Hospital	National Institute of Health. Department of medical Sciences, Ministry of Public Health, Thailand	Pilailuk et al
EPI_ISL_434702	Praram 9 Hospital	National Institute of Health. Department of medical Sciences, Ministry of Public Health, Thailand	Pilailuk et al
EPI_ISL_434707	Thammasat University Hospital	National Institute of Health. Department of medical Sciences, Ministry of Public Health, Thailand	Pilailuk et al
EPI_ISL_447919	n/a	National Institute of Health. Department of medical Sciences, Ministry of Public Health, Thailand	Pilailuk et al
EPI_ISL_708815	Urban Institute for Disease Prevention and Control	National Institute of Health, Department of Medical Sciences, Ministry of Public Health, Thailand	Pilailuk Okada et al
EPI_ISL_708817	Urban Institute for Disease Prevention and Control	National Institute of Health, Department of Medical Sciences, Ministry of Public Health, Thailand	Pilailuk Okada et al
EPI_ISL_708813	World Medical Hospital	National Institute of Health, Department of Medical Sciences, Ministry of Public Health, Thailand	Pilailuk Okada et al
EPI_ISL_708812	World Medical Hospital	National Institute of Health, Department of Medical Sciences, Ministry of Public Health, Thailand	Pilailuk Okada et al
EPI_ISL_768614	Regional Medical Sciences Center 1/1 Chiang Rai	National Institute of Health, Department of Medical Sciences, Ministry of Public Health, Thailand	Pilailuk Okada et al
EPI_ISL_708737	Regional medical sciences center 6 chonburi	National Institute of Health, Department of Medical Sciences, Ministry of Public Health, Thailand	Pilailuk Okada et al
EPI_ISL_708804	Regional medical sciences center 6 chonburi	National Institute of Health, Department of Medical Sciences, Ministry of Public Health, Thailand	Pilailuk Okada et al
EPI_ISL_708802	Regional medical sciences center 6 chonburi	National Institute of Health, Department of Medical Sciences, Ministry of Public Health, Thailand	Pilailuk Okada et al
EPI_ISL_708797	Regional medical sciences center 6 chonburi	National Institute of Health, Department of Medical Sciences, Ministry of Public Health, Thailand	Pilailuk Okada et al
EPI_ISL_708800	Regional medical sciences center 6 chonburi	National Institute of Health, Department of Medical Sciences, Ministry of Public Health, Thailand	Pilailuk Okada et al
EPI_ISL_708807	Regional medical sciences center 6 chonburi	National Institute of Health, Department of Medical Sciences, Ministry of Public Health, Thailand	Pilailuk Okada et al
EPI_ISL_708808	Regional medical sciences center 6 chonburi	National Institute of Health, Department of Medical Sciences, Ministry of Public Health, Thailand	Pilailuk Okada et al
EPI_ISL_708801	Regional medical sciences center 6 chonburi	National Institute of Health, Department of Medical Sciences, Ministry of Public Health, Thailand	Pilailuk Okada et al
EPI_ISL_768610	Regional medical sciences center 6 Chonburi	National Institute of Health, Department of Medical Sciences, Ministry of Public Health, Thailand	Pilailuk Okada et al

EPI_ISL_768611	Regional medical sciences center 6 Chonburi	National Institute of Health, Department of Medical Sciences, Ministry of Public Health, Thailand	Pilailuk Okada et al
EPI_ISL_1497597	The office of Disease Prevention and Control 6, Chonburi	National Institute of Health, Department of Medical Sciences, Ministry of Public Health, Thailand	Pilailuk Okada et al
EPI_ISL_755588	Regional medical sciences center 6 Chonburi	National Institute of Health, Department of Medical Sciences, Ministry of Public Health, Thailand	Pilailuk Okada et al
EPI_ISL_768617	Regional Medical Sciences Center 5 Samut Songkhram	National Institute of Health, Department of Medical Sciences, Ministry of Public Health, Thailand	Pilailuk Okada et al
EPI_ISL_768542	Regional Medical Sciences Center 5 Samut Songkhram	National Institute of Health, Department of Medical Sciences, Ministry of Public Health, Thailand	Pilailuk Okada et al
EPI_ISL_708811	Regional medical sciences center 2 Phitsanulok	National Institute of Health, Department of Medical Sciences, Ministry of Public Health, Thailand	Pilailuk Okada et al
EPI_ISL_471412	Viral Respiratory Lab, National Institute for Biomedical Research (INRB)	Pathogen Sequencing Lab, National Institute for Biomedical Research (INRB)	Placide Mbala-Kingebeni et al
EPI_ISL_471413	Viral Respiratory Lab, National Institute for Biomedical Research (INRB)	Pathogen Sequencing Lab, National Institute for Biomedical Research (INRB)	Placide Mbala-Kingebeni et al
EPI_ISL_527539	Viral Respiratory Lab, National Institute for Biomedical Research (INRB)	Pathogen Sequencing Lab, National Institute for Biomedical Research (INRB)	Placide Mbala-Kingebeni et al
EPI_ISL_447596	Viral Respiratory Lab, National Institute for Biomedical Research (INRB)	Pathogen Sequencing Lab, National Institute for Biomedical Research (INRB)	Placide Mbala-Kingebeni et al
EPI_ISL_965122	Viral Respiratory Lab, National Institute for Biomedical Research (INRB)	Pathogen Sequencing Lab, National Institute for Biomedical Research (INRB)	Placide Mbala-Kingebeni et al
EPI_ISL_417950	Viral Respiratory Lab, National Institute for Biomedical Research (INRB)	Pathogen Sequencing Lab, National Institute for Biomedical Research (INRB)	Placide Mbala-Kingebeni et al
EPI_ISL_591083	Viral Respiratory Lab, National Institute for Biomedical Research (INRB)	Pathogen Sequencing Lab, National Institute for Biomedical Research (INRB)	Placide Mbala-Kingebeni et al
EPI_ISL_591084	Viral Respiratory Lab, National Institute for Biomedical Research (INRB)	Pathogen Sequencing Lab, National Institute for Biomedical Research (INRB)	Placide Mbala-Kingebeni et al
EPI_ISL_591085	Viral Respiratory Lab, National Institute for Biomedical Research (INRB)	Pathogen Sequencing Lab, National Institute for Biomedical Research (INRB)	Placide Mbala-Kingebeni et al
EPI_ISL_591086	Viral Respiratory Lab, National Institute for Biomedical Research (INRB)	Pathogen Sequencing Lab, National Institute for Biomedical Research (INRB)	Placide Mbala-Kingebeni et al
EPI_ISL_420840	Viral Respiratory Lab, National Institute for Biomedical Research (INRB)	Pathogen Sequencing Lab, National Institute for Biomedical Research (INRB)	Placide Mbala-Kingebeni et al
EPI_ISL_420841	Viral Respiratory Lab, National Institute for Biomedical Research (INRB)	Pathogen Sequencing Lab, National Institute for Biomedical Research (INRB)	Placide Mbala-Kingebeni et al
EPI_ISL_447247	Viral Respiratory Lab, National Institute for Biomedical Research (INRB)	Pathogen Sequencing Lab, National Institute for Biomedical Research (INRB)	Placide Mbala-Kingebeni et al
EPI_ISL_420853	Viral Respiratory Lab, National Institute for Biomedical Research (INRB)	Pathogen Sequencing Lab, National Institute for Biomedical Research (INRB)	Placide Mbala-Kingebeni et al
EPI_ISL_513597	Viral Respiratory Lab, National Institute for Biomedical Research (INRB)	Pathogen Sequencing Lab, National Institute for Biomedical Research (INRB)	Placide Mbala-Kingebeni et al
EPI_ISL_496498	Viral Respiratory Lab, National Institute for Biomedical Research (INRB)	Pathogen Sequencing Lab, National Institute for Biomedical Research (INRB)	Placide Mbala-Kingebeni et al

EPI_ISL_961010	Viral Respiratory Lab, National Institute for Biomedical Research (INRB)	Pathogen Sequencing Lab, National Institute for Biomedical Research (INRB)	Placide Mbala-Kingebeni et al
EPI_ISL_1623756	Viral Respiratory Lab, National Institute for Biomedical Research (INRB)	Pathogen Sequencing Lab, National Institute for Biomedical Research (INRB)	Placide Mbala-Kingebeni et al
EPI_ISL_1623757	Viral Respiratory Lab, National Institute for Biomedical Research (INRB)	Pathogen Sequencing Lab, National Institute for Biomedical Research (INRB)	Placide Mbala-Kingebeni et al
EPI_ISL_417944	Viral Respiratory Lab, National Institute for Biomedical Research (INRB)	Pathogen Sequencing Lab, National Institute for Biomedical Research (INRB)	Placide Mbala-Kingebeni et al
EPI_ISL_965123	Viral Respiratory Lab, National Institute for Biomedical Research (INRB)	Pathogen Sequencing Lab, National Institute for Biomedical Research (INRB)	Placide Mbala-Kingebeni et al
EPI_ISL_965124	Viral Respiratory Lab, National Institute for Biomedical Research (INRB)	Pathogen Sequencing Lab, National Institute for Biomedical Research (INRB)	Placide Mbala-Kingebeni et al
EPI_ISL_1623758	Viral Respiratory Lab, National Institute for Biomedical Research (INRB)	Pathogen Sequencing Lab, National Institute for Biomedical Research (INRB)	Placide Mbala-Kingebeni et al
EPI_ISL_417434	Viral Respiratory Lab, National Institute for Biomedical Research (INRB)	Pathogen Sequencing Lab, National Institute for Biomedical Research (INRB)	Placide Mbala-Kingebeni et al
EPI_ISL_413522	Indian Council of Medical Research - National Institute of Virology	National Influenza Center, Indian Council of Medical Research - National Institute of Virology	Potdar V et al
EPI_ISL_413523	Indian Council of Medical Research-National Institute of Virology	National Influenza Center, Indian Council of Medical Research-National Institute of Virology	Potdar V et al
EPI_ISL_454528	NIV Influenza	NIV Influenza	Potdar V et al
EPI_ISL_454534	NIV Influenza	NIV Influenza	Potdar V et al
EPI_ISL_454537	NIV Influenza	NIV Influenza	Potdar V et al
EPI_ISL_479572	NIV Influenza	NIV Influenza	Potdar V et al
EPI_ISL_1034165	NIV Influenza	NIV Influenza	Potdar V et al
EPI_ISL_1034166	NIV Influenza	NIV Influenza	Potdar V et al
EPI_ISL_754068	Nepal Korea Friendship Municipality Hospital	Nepal Health Research Council	Pradip Gyanwali et al
EPI_ISL_754069	Nepal Korea Friendship Municipality Hospital	Nepal Health Research Council	Pradip Gyanwali et al
EPI_ISL_754062	Kathmandu University	Nepal Health Research Council	Pradip Gyanwali et al
EPI_ISL_754063	Kathmandu University	Nepal Health Research Council	Pradip Gyanwali et al
EPI_ISL_754064	Kathmandu University	Nepal Health Research Council	Pradip Gyanwali et al
EPI_ISL_754065	Kathmandu University	Nepal Health Research Council	Pradip Gyanwali et al
EPI_ISL_754067	Kathmandu University	Nepal Health Research Council	Pradip Gyanwali et al
EPI_ISL_754073	National Public Health Laboratory	Nepal Health Research Council	Pradip Gyanwali et al
EPI_ISL_754071	Nepal Korea Friendship Municipality Hospital	Nepal Health Research Council	Pradip Gyanwali et al
EPI_ISL_516650	Institute of Microbiology, Universidad San Francisco de Quito	Institute of Microbiology, Universidad San Francisco de Quito	Prado-Vivar et al
EPI_ISL_516651	Institute of Microbiology, Universidad San Francisco de Quito	Institute of Microbiology, Universidad San Francisco de Quito	Prado-Vivar et al

EPI_ISL_516652	Institute of Microbiology, Universidad San Francisco de Quito	Institute of Microbiology, Universidad San Francisco de Quito	Prado-Vivar et al
EPI_ISL_421670	National Influenza Center, Indian Council of Medical Research - National Institute of Virology	Indian Council of Medical Research-National Institute of Virology, Microbial Containment Complex	Pragya D. Yadav et al
EPI_ISL_1061417	Indian Council of Medical Research-National Institute of Virology, Microbial Containment Complex	Indian Council of Medical Research-National Institute of Virology, Microbial Containment Complex	Pragya D. Yadav et al
EPI_ISL_637099	Indian Council of Medical Research-National Institute of Virology, Microbial Containment Complex	Indian Council of Medical Research-National Institute of Virology, Microbial Containment Complex	Pragya D. Yadav et al
EPI_ISL_637102	Indian Council of Medical Research-National Institute of Virology, Microbial Containment Complex	Indian Council of Medical Research-National Institute of Virology, Microbial Containment Complex	Pragya D. Yadav et al
EPI_ISL_538348	Kingston Health Sciences Centre / Queen's University	Ontario Institute for Cancer Research	Prameet M. Sheth et al
EPI_ISL_538349	Kingston Health Sciences Centre / Queen's University	Ontario Institute for Cancer Research	Prameet M. Sheth et al
EPI_ISL_435089	National Centre for Disease control (NCDC), CSIR-Institute of Genomics and Integrative Biology (CSIR-IGIB)	NCDC/CSIR-IGIB	Pramod Kumar et al
EPI_ISL_435073	National Centre for Disease control (NCDC), CSIR-Institute of Genomics and Integrative Biology (CSIR-IGIB)	NCDC/CSIR-IGIB	Pramod Kumar et al
EPI_ISL_482628	National Centre for Disease control (NCDC)	NCDC/CSIR-IGIB	Pramod Kumar# et al
EPI_ISL_482629	National Centre for Disease control (NCDC)	NCDC/CSIR-IGIB	Pramod Kumar# et al
EPI_ISL_482630	National Centre for Disease control (NCDC)	NCDC/CSIR-IGIB	Pramod Kumar# et al
EPI_ISL_482632	National Centre for Disease control (NCDC)	NCDC/CSIR-IGIB	Pramod Kumar# et al
EPI_ISL_482641	National Centre for Disease control (NCDC)	NCDC/CSIR-IGIB	Pramod Kumar# et al
EPI_ISL_436453	National Centre for Disease control (NCDC)	NCDC/CSIR-IGIB	Pramod Kumar# et al
EPI_ISL_436463	National Centre for Disease control (NCDC)	NCDC/CSIR-IGIB	Pramod Kumar# et al
EPI_ISL_1499201	Associação Fundo de Incentivo à Pesquisa (AFIP)	Associação Fundo de Incentivo à Pesquisa (AFIP)	Priscila Farias Tempaku et al
EPI_ISL_1499202	Associação Fundo de Incentivo à Pesquisa (AFIP)	Associação Fundo de Incentivo à Pesquisa (AFIP)	Priscila Farias Tempaku et al
EPI_ISL_1499297	Associação Fundo de Incentivo à Pesquisa (AFIP)	Associação Fundo de Incentivo à Pesquisa (AFIP)	Priscila Farias Tempaku et al
EPI_ISL_1499205	Associação Fundo de Incentivo à Pesquisa (AFIP)	Associação Fundo de Incentivo à Pesquisa (AFIP)	Priscila Farias Tempaku et al
EPI_ISL_1499298	Associação Fundo de Incentivo à Pesquisa (AFIP)	Associação Fundo de Incentivo à Pesquisa (AFIP)	Priscila Farias Tempaku et al
EPI_ISL_1499299	Associação Fundo de Incentivo à Pesquisa (AFIP)	Associação Fundo de Incentivo à Pesquisa (AFIP)	Priscila Farias Tempaku et al
EPI_ISL_1499114	Associação Fundo de Incentivo à Pesquisa (AFIP)	Associação Fundo de Incentivo à Pesquisa (AFIP)	Priscila Farias Tempaku et al
EPI_ISL_1499300	Associação Fundo de Incentivo à Pesquisa (AFIP)	Associação Fundo de Incentivo à Pesquisa (AFIP)	Priscila Farias Tempaku et al
EPI_ISL_1654211	Fondation Congolaise pour la recherche medicale (FCRM), Francine Ntoumi	Institute of Tropical Medicine	Prof. Francine Ntoumi et al
EPI_ISL_1654212	Fondation Congolaise pour la recherche medicale (FCRM), Francine Ntoumi	Institute of Tropical Medicine	Prof. Francine Ntoumi et al
EPI_ISL_1654213	Fondation Congolaise pour la recherche medicale (FCRM), Francine Ntoumi	Institute of Tropical Medicine	Prof. Francine Ntoumi et al
EPI_ISL_1654214	Fondation Congolaise pour la recherche medicale (FCRM), Francine Ntoumi	Institute of Tropical Medicine	Prof. Francine Ntoumi et al

EPI_ISL_1654217	Fondation Congolaise pour la recherche medicale (FCRM), Francine Ntoumi	Institute of Tropical Medicine	Prof. Francine Ntoumi et al
EPI_ISL_1654218	Fondation Congolaise pour la recherche medicale (FCRM), Francine Ntoumi	Institute of Tropical Medicine	Prof. Francine Ntoumi et al
EPI_ISL_968754	BCCDC Public Health Laboratory	BCCDC Public Health Laboratory	Prystajecky Natalie et al
EPI_ISL_969102	BCCDC Public Health Laboratory	BCCDC Public Health Laboratory	Prystajecky Natalie et al
EPI_ISL_969908	BCCDC Public Health Laboratory	BCCDC Public Health Laboratory	Prystajecky Natalie et al
EPI_ISL_968466	BCCDC Public Health Laboratory	BCCDC Public Health Laboratory	Prystajecky Natalie et al
EPI_ISL_968521	BCCDC Public Health Laboratory	BCCDC Public Health Laboratory	Prystajecky Natalie et al
EPI_ISL_969755	BCCDC Public Health Laboratory	BCCDC Public Health Laboratory	Prystajecky Natalie et al
EPI_ISL_976424	BCCDC Public Health Laboratory	BCCDC Public Health Laboratory	Prystajecky Natalie et al
EPI_ISL_976425	BCCDC Public Health Laboratory	BCCDC Public Health Laboratory	Prystajecky Natalie et al
EPI_ISL_974609	BCCDC Public Health Laboratory	BCCDC Public Health Laboratory	Prystajecky Natalie et al
EPI_ISL_974610	BCCDC Public Health Laboratory	BCCDC Public Health Laboratory	Prystajecky Natalie et al
EPI_ISL_1250695	Wellington SCL (WN)	Institute of Environmental Science and Research (ESR)	Rachel Boyle et al
EPI_ISL_1082263	LabPLUS	Institute of Environmental Science and Research (ESR)	Rachel Boyle et al
EPI_ISL_1172032	LabPLUS	Institute of Environmental Science and Research (ESR)	Rachel Boyle et al
EPI_ISL_1172030	LabPLUS	Institute of Environmental Science and Research (ESR)	Rachel Boyle et al
EPI_ISL_1172029	LabPLUS	Institute of Environmental Science and Research (ESR)	Rachel Boyle et al
EPI_ISL_1250697	Middlemore Hospital	Institute of Environmental Science and Research (ESR)	Rachel Boyle et al
EPI_ISL_1250700	LabPLUS	Institute of Environmental Science and Research (ESR)	Rachel Boyle et al
EPI_ISL_1250699	LabPLUS	Institute of Environmental Science and Research (ESR)	Rachel Boyle et al
EPI_ISL_1315318	Middlemore Hospital	Institute of Environmental Science and Research (ESR)	Rachel Boyle et al
EPI_ISL_1469106	LabPLUS	Institute of Environmental Science and Research (ESR)	Rachel Boyle et al
EPI_ISL_1621303	LabPLUS	Institute of Environmental Science and Research (ESR)	Rachel Boyle et al
EPI_ISL_1621294	LabPLUS	Institute of Environmental Science and Research (ESR)	Rachel Boyle et al
EPI_ISL_1621311	Middlemore Hospital	Institute of Environmental Science and Research (ESR)	Rachel Boyle et al
EPI_ISL_1621325	LabPLUS	Institute of Environmental Science and Research (ESR)	Rachel Boyle et al
EPI_ISL_1621305	LabPLUS	Institute of Environmental Science and Research (ESR)	Rachel Boyle et al
EPI_ISL_1621304	LabPLUS	Institute of Environmental Science and Research (ESR)	Rachel Boyle et al
EPI_ISL_1651917	WSSE w Gdańsku	1. Academic Center for Pathomorphological and Genetic-Molecular Diagnostics ltd, Bialystok, Poland 2. National Institute of Public Health - National Institute of Hygiene, Warsaw, Poland	Radosław Charkiewicz et al

EPI_ISL_1651931	WSSE w Gdańsku	1. Academic Center for Pathomorphological and Genetic-Molecular Diagnostics ltd, Bialystok, Poland 2. National Institute of Public Health - National Institute of Hygiene, Warsaw, Poland	Radosław Charkiewicz et al
EPI_ISL_1659587	National Institute of Public Health - National Institute of Hygiene	1. Academic Center for Pathomorphological and Genetic-Molecular Diagnostics ltd, Bialystok, Poland 2. National Institute of Public Health - National Institute of Hygiene, Warsaw, Poland	Radosław Charkiewicz et al
EPI_ISL_1659598	National Institute of Public Health - National Institute of Hygiene	1. Academic Center for Pathomorphological and Genetic-Molecular Diagnostics ltd, Bialystok, Poland 2. National Institute of Public Health - National Institute of Hygiene, Warsaw, Poland	Radosław Charkiewicz et al
EPI_ISL_1659607	National Institute of Public Health - National Institute of Hygiene	1. Academic Center for Pathomorphological and Genetic-Molecular Diagnostics ltd, Bialystok, Poland 2. National Institute of Public Health - National Institute of Hygiene, Warsaw, Poland	Radosław Charkiewicz et al
EPI_ISL_512880	Pathogen Genomics Lab King Abdullah University of Science and Technology (KAUST)	Pathogen Genomics Lab King Abdullah University of Science and Technology(KAUST)	Raeece Naeem et al
EPI_ISL_512889	Pathogen Genomics Lab King Abdullah University of Science and Technology (KAUST)	Pathogen Genomics Lab King Abdullah University of Science and Technology(KAUST)	Raeece Naeem et al
EPI_ISL_512891	Pathogen Genomics Lab King Abdullah University of Science and Technology (KAUST)	Pathogen Genomics Lab King Abdullah University of Science and Technology(KAUST)	Raeece Naeem et al
EPI_ISL_512893	Pathogen Genomics Lab King Abdullah University of Science and Technology (KAUST)	Pathogen Genomics Lab King Abdullah University of Science and Technology(KAUST)	Raeece Naeem et al
EPI_ISL_512894	Pathogen Genomics Lab King Abdullah University of Science and Technology (KAUST)	Pathogen Genomics Lab King Abdullah University of Science and Technology(KAUST)	Raeece Naeem et al
EPI_ISL_512897	Pathogen Genomics Lab King Abdullah University of Science and Technology (KAUST)	Pathogen Genomics Lab King Abdullah University of Science and Technology(KAUST)	Raeece Naeem et al
EPI_ISL_678191	Pathogen Genomics Lab King Abdullah University of Science and Technology (KAUST)	Pathogen Genomics Lab King Abdullah University of Science and Technology(KAUST)	Raeece Naeem et al
EPI_ISL_513036	Pathogen Genomics Lab King Abdullah University of Science and Technology (KAUST)	Pathogen Genomics Lab King Abdullah University of Science and Technology(KAUST)	Rahul P Salunke et al
EPI_ISL_513049	Pathogen Genomics Lab King Abdullah University of Science and Technology (KAUST)	Pathogen Genomics Lab King Abdullah University of Science and Technology(KAUST)	Rahul P Salunke et al
EPI_ISL_513054	Pathogen Genomics Lab King Abdullah University of Science and Technology (KAUST)	Pathogen Genomics Lab King Abdullah University of Science and Technology(KAUST)	Rahul P Salunke et al
EPI_ISL_513055	Pathogen Genomics Lab King Abdullah University of Science and Technology (KAUST)	Pathogen Genomics Lab King Abdullah University of Science and Technology(KAUST)	Rahul P Salunke et al
EPI_ISL_513198	Pathogen Genomics Lab King Abdullah University of Science and Technology (KAUST)	Pathogen Genomics Lab King Abdullah University of Science and Technology(KAUST)	Rahul P Salunke et al
EPI_ISL_528422	TNMC & BYL NAIR CH. HOSPITAL	Institute of Genomics and Integrative Biology - Council of Scientific and Industrial Research	Rajesh Pandey et al
EPI_ISL_459913	Devki Devi Foundation, a unit of Max Healthcare	CSIR-IGIB/Max	Rajesh Pandey# et al
EPI_ISL_459914	Devki Devi Foundation, a unit of Max Healthcare	CSIR-IGIB/Max	Rajesh Pandey# et al
EPI_ISL_459916	Devki Devi Foundation, a unit of Max Healthcare	CSIR-IGIB/Max	Rajesh Pandey# et al
EPI_ISL_459924	Devki Devi Foundation, a unit of Max Healthcare	CSIR-IGIB/Max	Rajesh Pandey# et al
EPI_ISL_412972	Instituto Nacional de Enfermedades Respiratorias	Instituto de Diagnostico y Referencia Epidemiologicos (INDRE)	Ramirez-Gonzalez Ernesto et al

EPI_ISL_452139	Instituto de Diagnostico y Referencia Epidemiologicos (INDRE)	Instituto de diagnóstico y Referencia Epidemiologicos (INDRE)	Ramirez-Gonzalez Ernesto et al
EPI_ISL_985237	Biorepository and Clinical Virology Laboratory	Ozer Lab	Ramon Lorenzo-Redondo et al
EPI_ISL_985238	Biorepository and Clinical Virology Laboratory	Ozer Lab	Ramon Lorenzo-Redondo et al
EPI_ISL_1191597	Virology Department, Victoria Hospital, Plaine-Wilhems, Mauritius	National Institute for Communicable Diseases of the National Health Laboratory Service	Ramuth M et al
EPI_ISL_1191600	Virology Department, Victoria Hospital, Plaine-Wilhems, Mauritius	National Institute for Communicable Diseases of the National Health Laboratory Service	Ramuth M et al
EPI_ISL_1191602	Virology Department, Victoria Hospital, Plaine-Wilhems, Mauritius	National Institute for Communicable Diseases of the National Health Laboratory Service	Ramuth M et al
EPI_ISL_1191604	Virology Department, Victoria Hospital, Plaine-Wilhems, Mauritius	National Institute for Communicable Diseases of the National Health Laboratory Service	Ramuth M et al
EPI_ISL_1191605	Virology Department, Victoria Hospital, Plaine-Wilhems, Mauritius	National Institute for Communicable Diseases of the National Health Laboratory Service	Ramuth M et al
EPI_ISL_569988	Unity Health Toronto	Ontario Institute for Cancer Research	Ramzi Fattouh et al
EPI_ISL_526267	Unity Health Toronto	Ontario Institute for Cancer Research	Ramzi Fattouh et al
EPI_ISL_1248874	Unity Health Toronto	Ontario Institute for Cancer Research	Ramzi Fattouh et al
EPI_ISL_1248878	Unity Health Toronto	Ontario Institute for Cancer Research	Ramzi Fattouh et al
EPI_ISL_1248880	Unity Health Toronto	Ontario Institute for Cancer Research	Ramzi Fattouh et al
EPI_ISL_1248884	Unity Health Toronto	Ontario Institute for Cancer Research	Ramzi Fattouh et al
EPI_ISL_1248890	Unity Health Toronto	Ontario Institute for Cancer Research	Ramzi Fattouh et al
EPI_ISL_1248891	Unity Health Toronto	Ontario Institute for Cancer Research	Ramzi Fattouh et al
EPI_ISL_1248887	Unity Health Toronto	Ontario Institute for Cancer Research	Ramzi Fattouh et al
EPI_ISL_1045717	Randox Laboratories	Wellcome Sanger Institute for the COVID-19 Genomics UK (COG-UK) Consortium	Randox Laboratories et al
EPI_ISL_1631841	Randox Laboratories	Wellcome Sanger Institute for the COVID-19 Genomics UK (COG-UK) Consortium	Randox Laboratories et al
EPI_ISL_1631888	Randox Laboratories	Wellcome Sanger Institute for the COVID-19 Genomics UK (COG-UK) Consortium	Randox Laboratories et al
EPI_ISL_1632206	Randox Laboratories	Wellcome Sanger Institute for the COVID-19 Genomics UK (COG-UK) Consortium	Randox Laboratories et al
EPI_ISL_410301	National Influenza Centre, National Public Health Laboratory, Kathmandu, Nepal	The University of Hong Kong	Ranjit Sah et al
EPI_ISL_444851	Department of Virus and Microbiological Special Diagnostics, Statens Serum Institut, Copenhagen, Denmark, Artillerivej 5, 2300 Copenahgen S	Albertsen lab, Department of Chemistry and Bioscience, Aalborg University, Denmark	Rasmus Kirkegaard et al
EPI_ISL_444919	Department of Virus and Microbiological Special Diagnostics, Statens Serum Institut, Copenhagen, Denmark, Artillerivej 5, 2300 Copenahgen S	Albertsen lab, Department of Chemistry and Bioscience, Aalborg University, Denmark	Rasmus Kirkegaard et al
EPI_ISL_429498	Department of Virus and Microbiological Special Diagnostics, Statens Serum Institut, Copenhagen, Denmark, Artillerivej 5, 2300 Copenahgen S	Albertsen lab, Department of Chemistry and Bioscience, Aalborg University, Denmark	Rasmus Kirkegaard et al

EPI_ISL_527019	Area of Virology, Serology and Virology Division (SAViD), New South Wales Health Pathology Randwick	Area of Virology, Serology and Virology Division (SAViD), New South Wales Health Pathology Randwick	Rawlinson et al
EPI_ISL_527032	Area of Virology, Serology and Virology Division (SAViD), New South Wales Health Pathology Randwick	Area of Virology, Serology and Virology Division (SAViD), New South Wales Health Pathology Randwick	Rawlinson et al
EPI_ISL_877727	Clinical Molecular Microbiology Laboratory, UNC Hospital	Dirk Dittmer	Razia Moorad et al
EPI_ISL_678253	Institute for Lung Diseases in Children - Skopje	Research Center for Genetic Engineering and Biotechnology Georgi D. Efremov" , Macedonian Academy of Sciences and Arts	RCGEB - MASA et al
EPI_ISL_678256	General Hospital - Ohrid	Research Center for Genetic Engineering and Biotechnology Georgi D. Efremov" , Macedonian Academy of Sciences and Arts	RCGEB - MASA et al
EPI_ISL_678257	PHI Institute of Nephrology - Struga	Research Center for Genetic Engineering and Biotechnology Georgi D. Efremov" , Macedonian Academy of Sciences and Arts	RCGEB - MASA et al
EPI_ISL_678259	Clinical Hospital - Shtip	Research Center for Genetic Engineering and Biotechnology Georgi D. Efremov" , Macedonian Academy of Sciences and Arts	RCGEB - MASA et al
EPI_ISL_918492	General Hospital - Kumanovo	Research Center for Genetic Engineering and Biotechnology Georgi D. Efremov" , Macedonian Academy of Sciences and Arts	RCGEB - MASA et al
EPI_ISL_918488	General Hospital - Strumica	Research Center for Genetic Engineering and Biotechnology Georgi D. Efremov" , Macedonian Academy of Sciences and Arts	RCGEB - MASA et al
EPI_ISL_933579	General Hospital - Strumica	Research Center for Genetic Engineering and Biotechnology Georgi D. Efremov" , Macedonian Academy of Sciences and Arts	RCGEB - MASA et al
EPI_ISL_933717	General Hospital - Strumica	Research Center for Genetic Engineering and Biotechnology Georgi D. Efremov" , Macedonian Academy of Sciences and Arts	RCGEB - MASA et al
EPI_ISL_933772	General Hospital - Bitola	Research Center for Genetic Engineering and Biotechnology Georgi D. Efremov" , Macedonian Academy of Sciences and Arts	RCGEB - MASA et al
EPI_ISL_933786	General Hospital - Bitola	Research Center for Genetic Engineering and Biotechnology Georgi D. Efremov" , Macedonian Academy of Sciences and Arts	RCGEB - MASA et al
EPI_ISL_934314	General Hospital - Bitola	Research Center for Genetic Engineering and Biotechnology Georgi D. Efremov" , Macedonian Academy of Sciences and Arts	RCGEB - MASA et al
EPI_ISL_934413	General Hospital - Shtip	Research Center for Genetic Engineering and Biotechnology Georgi D. Efremov" , Macedonian Academy of Sciences and Arts	RCGEB - MASA et al
EPI_ISL_677708	Center for public health - Skopje	Research Center for Genetic Engineering and Biotechnology Georgi D. Efremov" , Macedonian Academy of Sciences and Arts	RCGEB - MASA et al
EPI_ISL_516428	Clinical Hospital - Shtip	Research Center for Genetic Engineering and Biotechnology Georgi D. Efremov" , Macedonian Academy of Sciences and Arts	RCGEB - MASA et al
EPI_ISL_516429	Clinical Hospital - Shtip	Research Center for Genetic Engineering and Biotechnology Georgi D. Efremov" , Macedonian Academy of Sciences and Arts	RCGEB - MASA et al
EPI_ISL_677716	Clinical Hospital - Shtip	Research Center for Genetic Engineering and Biotechnology Georgi D. Efremov" , Macedonian Academy of Sciences and Arts	RCGEB - MASA et al
EPI_ISL_677674	General Hospital - Prilep	Research Center for Genetic Engineering and Biotechnology Georgi D. Efremov" , Macedonian Academy of Sciences and Arts	RCGEB - MASA et al
EPI_ISL_677724	Clinical Hospital - Shtip	Research Center for Genetic Engineering and Biotechnology Georgi D. Efremov" , Macedonian Academy of Sciences and Arts	RCGEB - MASA et al
EPI_ISL_678248	Center for public health - Skopje	Research Center for Genetic Engineering and Biotechnology Georgi D. Efremov" , Macedonian Academy of Sciences and Arts	RCGEB - MASA et al

EPI_ISL_678249	Center for public health - Skopje	Research Center for Genetic Engineering and Biotechnology Georgi D. Efremov" , Macedonian Academy of Sciences and Arts	RCGEB - MASA et al
EPI_ISL_678250	Clinical Hospital - Shtip	Research Center for Genetic Engineering and Biotechnology Georgi D. Efremov" , Macedonian Academy of Sciences and Arts	RCGEB - MASA et al
EPI_ISL_933566	General Hospital - Bitola	Research Center for Genetic Engineering and Biotechnology Georgi D. Efremov" , Macedonian Academy of Sciences and Arts	RCGEB - MASA et al
EPI_ISL_1315064	Armed Forces Medical Research Laboratories and Blood Bank, Egypt	Department of Microbial Biotechnology, Genetic Engineering Division, National Research Centre,	Reham Dawood et al
EPI_ISL_1585247	GHC Genetics, s.r.o.	GHC Genetics, s.r.o.	Renata Michalovská et al
EPI_ISL_467300	General Hospital Abdulah Nakas	Alea Genetic Center	Rijad Konjhodzic et al
EPI_ISL_1653935	Alea Genetic Center	Alea Genetic Center	Rijad Konjhodzic et al
EPI_ISL_910329	ALEA dr Kandic	Alea Genetic Centre	Rijad Konjhodzic et al
EPI_ISL_922076	General Hospital #Abdulah Nakas Sarajevo	Alea Genetic Centre	Rijad Konjhodzic et al
EPI_ISL_910334	ALEA dr Kandic	Alea Genetic Centre	Rijad Konjhodzic et al
EPI_ISL_560991	RSUD Dr. Soetomo	Institute of Tropical Disease, Universitas Airlangga	Rima R Prasetya et al
EPI_ISL_1165070	Siti Khodijah Hospital	Institute of Tropical Disease, Universitas Airlangga	Rima R Prasetya et al
EPI_ISL_1324435	National Food and Veterinary Risk Assessment Institute	Vilnius University Hospital Santaros Klinikos, Center of Laboratory Medicine	Rimvydas Norvilas et al
EPI_ISL_1041030	Dr. Cipto Mangunkusumo (RSCM) Hospital	Faculty of Medicine, Universitas Indonesia	Rina La Distia Nora et al
EPI_ISL_450509	Rafik Hariri University Hospital	Rafik Hariri University Hospital	Rita Feghali et al
EPI_ISL_450512	Rafik Hariri University Hospital	Rafik Hariri University Hospital	Rita Feghali et al
EPI_ISL_450514	Rafik Hariri University Hospital	Rafik Hariri University Hospital	Rita Feghali et al
EPI_ISL_450515	Rafik Hariri University Hospital	Rafik Hariri University Hospital	Rita Feghali et al
EPI_ISL_671974	Laboratorio de Virología y Microbiología Molecular, Depto. de Microbiología, Facultad de Medicina, Universidad de El Salvador/INS-laboratorio de Ref. Ministerio de Salud	Laboratorio de Virología y Microbiología Molecular, Depto. de Microbiología, Facultad de Medicina, Universidad de El Salvador/INS-laboratorio de Ref. Ministerio de Salud	Rivera NR et al
EPI_ISL_1187189	Lighthouse Lab in Cambridge	Wellcome Sanger Institute for the COVID-19 Genomics UK (COG-UK) Consortium	Rob Howes et al
EPI_ISL_1327496	Lighthouse Lab in Cambridge	Wellcome Sanger Institute for the COVID-19 Genomics UK (COG-UK) Consortium	Rob Howes et al
EPI_ISL_1327150	Lighthouse Lab in Cambridge	Wellcome Sanger Institute for the COVID-19 Genomics UK (COG-UK) Consortium	Rob Howes et al
EPI_ISL_1455639	Lighthouse Lab in Cambridge	Wellcome Sanger Institute for the COVID-19 Genomics UK (COG-UK) Consortium	Rob Howes et al
EPI_ISL_1653315	Lighthouse Lab in Cambridge	Wellcome Sanger Institute for the COVID-19 Genomics UK (COG-UK) Consortium	Rob Howes et al
EPI_ISL_1653472	Lighthouse Lab in Cambridge	Wellcome Sanger Institute for the COVID-19 Genomics UK (COG-UK) Consortium	Rob Howes et al

EPI_ISL_673528	Lighthouse Lab in Cambridge	Wellcome Sanger Institute for the COVID-19 Genomics UK (COG-UK) Consortium	Rob Howes et al
EPI_ISL_710574	University Hospital Dubrava	Ruđer Boškovic Institute; Forensic Science Centre Ivan Vučetić; University of Zagreb Faculty of Science	Robert Belužić et al
EPI_ISL_710568	University Hospital Dubrava	Ruđer Boškovic Institute; Forensic Science Centre Ivan Vučetić; University of Zagreb Faculty of Science	Robert Belužić et al
EPI_ISL_710549	University Hospital Dubrava	Ruđer Boškovic Institute; Forensic Science Centre Ivan Vučetić; University of Zagreb Faculty of Science	Robert Belužić et al
EPI_ISL_710555	University Hospital Dubrava	Ruđer Boškovic Institute; Forensic Science Centre Ivan Vučetić; University of Zagreb Faculty of Science	Robert Belužić et al
EPI_ISL_710548	University Hospital Dubrava	Ruđer Boškovic Institute; Forensic Science Centre Ivan Vučetić; University of Zagreb Faculty of Science	Robert Belužić et al
EPI_ISL_710567	University Hospital Dubrava	Ruđer Boškovic Institute; Forensic Science Centre Ivan Vučetić; University of Zagreb Faculty of Science	Robert Belužić et al
EPI_ISL_710566	University Hospital Dubrava	Ruđer Boškovic Institute; Forensic Science Centre Ivan Vučetić; University of Zagreb Faculty of Science	Robert Belužić et al
EPI_ISL_710570	University Hospital Dubrava	Ruđer Boškovic Institute; Forensic Science Centre Ivan Vučetić; University of Zagreb Faculty of Science	Robert Belužić et al
EPI_ISL_710565	University Hospital Dubrava	Ruđer Boškovic Institute; Forensic Science Centre Ivan Vučetić; University of Zagreb Faculty of Science	Robert Belužić et al
EPI_ISL_710573	University Hospital Dubrava	Ruđer Boškovic Institute; Forensic Science Centre Ivan Vučetić; University of Zagreb Faculty of Science	Robert Belužić et al
EPI_ISL_710547	Sestre Milosrdnice University Hospital Center	Ruđer Boškovic Institute; Forensic Science Centre Ivan Vučetić; University of Zagreb Faculty of Science	Robert Belužić et al
EPI_ISL_710554	Sestre Milosrdnice University Hospital Center	Ruđer Boškovic Institute; Forensic Science Centre Ivan Vučetić; University of Zagreb Faculty of Science	Robert Belužić et al
EPI_ISL_710556	University Hospital Dubrava	Ruđer Boškovic Institute; Forensic Science Centre Ivan Vučetić; University of Zagreb Faculty of Science	Robert Belužić et al
EPI_ISL_710571	University Hospital Dubrava	Ruđer Boškovic Institute; Forensic Science Centre Ivan Vučetić; University of Zagreb Faculty of Science	Robert Belužić et al
EPI_ISL_710560	University Hospital Dubrava	Ruđer Boškovic Institute; Forensic Science Centre Ivan Vučetić; University of Zagreb Faculty of Science	Robert Belužić et al
EPI_ISL_710557	University Hospital Dubrava	Ruđer Boškovic Institute; Forensic Science Centre Ivan Vučetić; University of Zagreb Faculty of Science	Robert Belužić et al
EPI_ISL_710572	University Hospital Dubrava	Ruđer Boškovic Institute; Forensic Science Centre Ivan Vučetić; University of Zagreb Faculty of Science	Robert Belužić et al
EPI_ISL_710558	University Hospital Dubrava	Ruđer Boškovic Institute; Forensic Science Centre Ivan Vučetić; University of Zagreb Faculty of Science	Robert Belužić et al
EPI_ISL_525467	Universidad Iberoamericana	International Centre for Genetic Engineering and Biotechnology (ICGEB) and ARGO Open Lab Platform	Robert Paulino-Ramirez et al
EPI_ISL_525468	Universidad Iberoamericana	International Centre for Genetic Engineering and Biotechnology (ICGEB) and ARGO Open Lab Platform	Robert Paulino-Ramirez et al
EPI_ISL_525469	Universidad Iberoamericana	International Centre for Genetic Engineering and Biotechnology (ICGEB) and ARGO Open Lab Platform	Robert Paulino-Ramirez et al

EPI_ISL_523811	Universidad Iberoamericana, Instituto de Medicina Tropical & Salud Global	International Centre for Genetic Engineering and Biotechnology (ICGEB) and ARGO Open Lab Platform	Robert Paulino-Ramirez et al
EPI_ISL_523812	Universidad Iberoamericana, Instituto de Medicina Tropical & Salud Global	International Centre for Genetic Engineering and Biotechnology (ICGEB) and ARGO Open Lab Platform	Robert Paulino-Ramirez et al
EPI_ISL_1123293	Abdul Malek Ukil Medical College, Noakhali	Central Biological Research Laboratory and Department of Biochemistry and Molecular Biology	Robiul Hasan Bhuiyan et al
EPI_ISL_413595	Centre for Infectious Diseases and Microbiology Laboratory Services	NSW Health Pathology - Institute of Clinical Pathology and Medical Research; Westmead Hospital; University of Sydney	Rockett R et al
EPI_ISL_437620	unknown	Faculty of Medicine	Rodpan et al
EPI_ISL_437618	unknown	Faculty of Medicine	Rodpan et al
EPI_ISL_437615	unknown	Faculty of Medicine	Rodpan et al
EPI_ISL_437614	unknown	Faculty of Medicine	Rodpan et al
EPI_ISL_437613	unknown	Faculty of Medicine	Rodpan et al
EPI_ISL_437612	unknown	Faculty of Medicine	Rodpan et al
EPI_ISL_877766	Thai Red Cross Emerging Infectious Diseases Health Science Centre, Chulalongkorn Hospital, Faculty of Medicine, Chulalongkorn University	Thai Red Cross Emerging Infectious Diseases Center and Faculty of Medicine, Chulalongkorn University	Rome Buathong et al
EPI_ISL_981004	Thai Red Cross Emerging Infectious Diseases Health Science Centre, Chulalongkorn Hospital, Faculty of Medicine, Chulalongkorn University	Thai Red Cross Emerging Infectious Diseases Center and Faculty of Medicine, Chulalongkorn University	Rome Buathong et al
EPI_ISL_1007658	Thai Red Cross Emerging Infectious Diseases Health Science Centre, Chulalongkorn Hospital, Faculty of Medicine, Chulalongkorn University	Thai Red Cross Emerging Infectious Diseases Center and Faculty of Medicine, Chulalongkorn University	Rome Buathong et al
EPI_ISL_1111083	Laboratorio de Referencia Nacional de Virus Respiratorio. Instituto Nacional de Salud Perú	Laboratorio de Referencia Nacional de Enteropatógenos. Instituto Nacional de Salud del Perú	Ronnie Gavilan Chavez et al
EPI_ISL_1111112	Laboratorio de Referencia Nacional de Virus Respiratorio. Instituto Nacional de Salud Perú	Laboratorio de Referencia Nacional de Enteropatógenos. Instituto Nacional de Salud del Perú	Ronnie Gavilan Chavez et al
EPI_ISL_1111137	Laboratorio de Referencia Nacional de Virus Respiratorio. Instituto Nacional de Salud Perú	Laboratorio de Referencia Nacional de Enteropatógenos. Instituto Nacional de Salud del Perú	Ronnie Gavilan Chavez et al
EPI_ISL_1111142	Laboratorio de Referencia Nacional de Virus Respiratorio. Instituto Nacional de Salud Perú	Laboratorio de Referencia Nacional de Enteropatógenos. Instituto Nacional de Salud del Perú	Ronnie Gavilan Chavez et al
EPI_ISL_1111143	Laboratorio de Referencia Nacional de Virus Respiratorio. Instituto Nacional de Salud Perú	Laboratorio de Referencia Nacional de Enteropatógenos. Instituto Nacional de Salud del Perú	Ronnie Gavilan Chavez et al
EPI_ISL_1111151	Laboratorio de Referencia Nacional de Virus Respiratorio. Instituto Nacional de Salud Perú	Laboratorio de Referencia Nacional de Enteropatógenos. Instituto Nacional de Salud del Perú	Ronnie Gavilan Chavez et al
EPI_ISL_1111152	Laboratorio de Referencia Nacional de Virus Respiratorio. Instituto Nacional de Salud Perú	Laboratorio de Referencia Nacional de Enteropatógenos. Instituto Nacional de Salud del Perú	Ronnie Gavilan Chavez et al
EPI_ISL_1111169	Laboratorio de Referencia Nacional de Virus Respiratorio. Instituto Nacional de Salud Perú	Laboratorio de Referencia Nacional de Enteropatógenos. Instituto Nacional de Salud del Perú	Ronnie Gavilan Chavez et al
EPI_ISL_1111214	Laboratorio de Referencia Nacional de Virus Respiratorio. Instituto Nacional de Salud Perú	Laboratorio de Referencia Nacional de Enteropatógenos. Instituto Nacional de Salud del Perú	Ronnie Gavilan Chavez et al
EPI_ISL_1111218	Laboratorio de Referencia Nacional de Virus Respiratorio. Instituto Nacional de Salud Perú	Laboratorio de Referencia Nacional de Enteropatógenos. Instituto Nacional de Salud del Perú	Ronnie Gavilan Chavez et al
EPI_ISL_1111220	Laboratorio de Referencia Nacional de Virus Respiratorio. Instituto Nacional de Salud Perú	Laboratorio de Referencia Nacional de Enteropatógenos. Instituto Nacional de Salud del Perú	Ronnie Gavilan Chavez et al

EPI_ISL_1111223	Laboratorio de Referencia Nacional de Virus Respiratorio. Instituto Nacional de Salud Perú	Laboratorio de Referencia Nacional de Enteropatógenos. Instituto Nacional de Salud del Perú	Ronnie Gavilan Chavez et al
EPI_ISL_1111242	Laboratorio de Referencia Nacional de Virus Respiratorio. Instituto Nacional de Salud Perú	Laboratorio de Referencia Nacional de Enteropatógenos. Instituto Nacional de Salud del Perú	Ronnie Gavilan Chavez et al
EPI_ISL_1111249	Laboratorio de Referencia Nacional de Virus Respiratorio. Instituto Nacional de Salud Perú	Laboratorio de Referencia Nacional de Enteropatógenos. Instituto Nacional de Salud del Perú	Ronnie Gavilan Chavez et al
EPI_ISL_1111252	Laboratorio de Referencia Nacional de Virus Respiratorio. Instituto Nacional de Salud Perú	Laboratorio de Referencia Nacional de Enteropatógenos. Instituto Nacional de Salud del Perú	Ronnie Gavilan Chavez et al
EPI_ISL_1111266	Laboratorio de Referencia Nacional de Virus Respiratorio. Instituto Nacional de Salud Perú	Laboratorio de Referencia Nacional de Enteropatógenos. Instituto Nacional de Salud del Perú	Ronnie Gavilan Chavez et al
EPI_ISL_1111267	Laboratorio de Referencia Nacional de Virus Respiratorio. Instituto Nacional de Salud Perú	Laboratorio de Referencia Nacional de Enteropatógenos. Instituto Nacional de Salud del Perú	Ronnie Gavilan Chavez et al
EPI_ISL_1111271	Laboratorio de Referencia Nacional de Virus Respiratorio. Instituto Nacional de Salud Perú	Laboratorio de Referencia Nacional de Enteropatógenos. Instituto Nacional de Salud del Perú	Ronnie Gavilan Chavez et al
EPI_ISL_1111275	Laboratorio de Referencia Nacional de Virus Respiratorio. Instituto Nacional de Salud Perú	Laboratorio de Referencia Nacional de Enteropatógenos. Instituto Nacional de Salud del Perú	Ronnie Gavilan Chavez et al
EPI_ISL_1111312	Laboratorio de Referencia Nacional de Virus Respiratorio. Instituto Nacional de Salud Perú	Laboratorio de Referencia Nacional de Enteropatógenos. Instituto Nacional de Salud del Perú	Ronnie Gavilan Chavez et al
EPI_ISL_1111374	Laboratorio de Referencia Nacional de Virus Respiratorio. Instituto Nacional de Salud Perú	Laboratorio de Referencia Nacional de Enteropatógenos. Instituto Nacional de Salud del Perú	Ronnie Gavilan Chavez et al
EPI_ISL_1111384	Laboratorio de Referencia Nacional de Virus Respiratorio. Instituto Nacional de Salud Perú	Laboratorio de Referencia Nacional de Enteropatógenos. Instituto Nacional de Salud del Perú	Ronnie Gavilan Chavez et al
EPI_ISL_1111397	Laboratorio de Referencia Nacional de Enteropatógenos. Instituto Nacional de Salud del Perú	Laboratorio de Referencia Nacional de Enteropatógenos. Instituto Nacional de Salud del Perú	Ronnie Gavilan Chavez et al
EPI_ISL_1111414	Laboratorio de Referencia Nacional de Enteropatógenos. Instituto Nacional de Salud del Perú	Laboratorio de Referencia Nacional de Enteropatógenos. Instituto Nacional de Salud del Perú	Ronnie Gavilan Chavez et al
EPI_ISL_1111415	Laboratorio de Referencia Nacional de Enteropatógenos. Instituto Nacional de Salud del Perú	Laboratorio de Referencia Nacional de Enteropatógenos. Instituto Nacional de Salud del Perú	Ronnie Gavilan Chavez et al
EPI_ISL_1111450	Laboratorio de Referencia Nacional de Virus Respiratorio. Instituto Nacional de Salud Perú	Laboratorio de Referencia Nacional de Enteropatógenos. Instituto Nacional de Salud del Perú	Ronnie Gavilan Chavez et al
EPI_ISL_1111451	Laboratorio de Referencia Nacional de Virus Respiratorio. Instituto Nacional de Salud Perú	Laboratorio de Referencia Nacional de Enteropatógenos. Instituto Nacional de Salud del Perú	Ronnie Gavilan Chavez et al
EPI_ISL_1111461	Laboratorio de Referencia Nacional de Virus Respiratorio. Instituto Nacional de Salud Perú	Laboratorio de Referencia Nacional de Enteropatógenos. Instituto Nacional de Salud del Perú	Ronnie Gavilan Chavez et al
EPI_ISL_1111467	Laboratorio de Referencia Nacional de Virus Respiratorio. Instituto Nacional de Salud Perú	Laboratorio de Referencia Nacional de Enteropatógenos. Instituto Nacional de Salud del Perú	Ronnie Gavilan Chavez et al
EPI_ISL_1111472	Laboratorio de Referencia Nacional de Virus Respiratorio. Instituto Nacional de Salud Perú	Laboratorio de Referencia Nacional de Enteropatógenos. Instituto Nacional de Salud del Perú	Ronnie Gavilan Chavez et al
EPI_ISL_1111474	Laboratorio de Referencia Nacional de Virus Respiratorio. Instituto Nacional de Salud Perú	Laboratorio de Referencia Nacional de Enteropatógenos. Instituto Nacional de Salud del Perú	Ronnie Gavilan Chavez et al
EPI_ISL_1111475	Laboratorio de Referencia Nacional de Virus Respiratorio. Instituto Nacional de Salud Perú	Laboratorio de Referencia Nacional de Enteropatógenos. Instituto Nacional de Salud del Perú	Ronnie Gavilan Chavez et al
EPI_ISL_1111483	Laboratorio de Referencia Nacional de Virus Respiratorio. Instituto Nacional de Salud Perú	Laboratorio de Referencia Nacional de Enteropatógenos. Instituto Nacional de Salud del Perú	Ronnie Gavilan Chavez et al

EPI_ISL_1111484	Laboratorio de Referencia Nacional de Virus Respiratorio. Instituto Nacional de Salud Perú	Laboratorio de Referencia Nacional de Enteropatógenos. Instituto Nacional de Salud del Perú	Ronnie Gavilan Chavez et al
EPI_ISL_1111486	Laboratorio de Referencia Nacional de Virus Respiratorio. Instituto Nacional de Salud Perú	Laboratorio de Referencia Nacional de Enteropatógenos. Instituto Nacional de Salud del Perú	Ronnie Gavilan Chavez et al
EPI_ISL_1111490	Laboratorio de Referencia Nacional de Virus Respiratorio. Instituto Nacional de Salud Perú	Laboratorio de Referencia Nacional de Enteropatógenos. Instituto Nacional de Salud del Perú	Ronnie Gavilan Chavez et al
EPI_ISL_1111496	Laboratorio de Referencia Nacional de Virus Respiratorio. Instituto Nacional de Salud Perú	Laboratorio de Referencia Nacional de Enteropatógenos. Instituto Nacional de Salud del Perú	Ronnie Gavilan Chavez et al
EPI_ISL_1395790	Laboratorio de salud pública, Facultad de Ciencias Exactas, UNLP	Área de Secuenciación del Laboratorio de Virología del Hospital de Niños Dr. Ricardo Gutierrez on behalf of 'Proyecto Argentino Interinstitucional de genomica de SARS-CoV-2' (PAIS Consortium)	Rosana Isabel Toro et al
EPI_ISL_537988	Servicio de Microbiología. Hospital General Universitario de Castellón	SeqCOVID-SPAIN consortium/IBV(CSIC)	Rosario Moreno et al
EPI_ISL_794599	Central Laboratories, Egyptian Ministry of Health and Population	Central Laboratories, Egyptian Ministry of Health and Population	Roshdy et al
EPI_ISL_794595	Central Laboratories, Egyptian Ministry of Health and Population	Central Laboratories, Egyptian Ministry of Health and Population	Roshdy et al
EPI_ISL_794594	Central Laboratories, Egyptian Ministry of Health and Population	Central Laboratories, Egyptian Ministry of Health and Population	Roshdy et al
EPI_ISL_794593	Central Laboratories, Egyptian Ministry of Health and Population	Central Laboratories, Egyptian Ministry of Health and Population	Roshdy et al
EPI_ISL_981226	National Virus Reference Laboratory	Irish Coronavirus Sequencing Consortium - Helixworks	Sachin Chalapati et al
EPI_ISL_981259	National Virus Reference Laboratory	Irish Coronavirus Sequencing Consortium - Helixworks	Sachin Chalapati et al
EPI_ISL_981262	National Virus Reference Laboratory	Irish Coronavirus Sequencing Consortium - Helixworks	Sachin Chalapati et al
EPI_ISL_981269	National Virus Reference Laboratory	Irish Coronavirus Sequencing Consortium - Helixworks	Sachin Chalapati et al
EPI_ISL_981231	National Virus Reference Laboratory	Irish Coronavirus Sequencing Consortium - Helixworks	Sachin Chalapati et al
EPI_ISL_935039	Biolab Diagnostic Laboratories	Princess Haya Biotechnology Center, Jordan University of Science and Technology	Saied Jaradat et al
EPI_ISL_935041	Biolab Diagnostic Laboratories	Princess Haya Biotechnology Center, Jordan University of Science and Technology	Saied Jaradat et al
EPI_ISL_935043	Biolab Diagnostic Laboratories	Princess Haya Biotechnology Center/ Jordan University of Science & Technology	Saied Jaradat et al
EPI_ISL_525423	Oman-National Influenza Center	Biotechnology & OMICs Laboratory	Sajjad Asaf et al
EPI_ISL_525426	Oman-National Influenza Center	Biotechnology & OMICs Laboratory	Sajjad Asaf et al
EPI_ISL_525428	Oman-National Influenza Center	Biotechnology & OMICs Laboratory	Sajjad Asaf et al
EPI_ISL_639739	Centre of Nanotechnologies, INCD IMT-Bucuresti (National Institute for Research and Development in Microtechnologies - Bucharest)	Centre of Nanotechnologies, INCD IMT-Bucuresti (National Institute for Research and Development in Microtechnologies - Bucharest)	Salceanu et al
EPI_ISL_722201	Vitalis Mostar	Alea Genetic Center	Salihefendic L. et al
EPI_ISL_722209	Dom Zdravlja Sarajevo	Alea Genetic Center	Salihefendic L. et al
EPI_ISL_903383	MOH - Jaber Al-Ahmad Hospital (Innovation Research Laboratory)	MOH - Jaber Al-Ahmad Hospital (Innovation Research Laboratory)	Salman Al-Sabah et al
EPI_ISL_903376	MOH - Jaber Al-Ahmad Hospital (Innovation Research Laboratory)	MOH - Jaber Al-Ahmad Hospital (Innovation Research Laboratory)	Salman Al-Sabah et al

EPI_ISL_903384	MOH - Jaber Al-Ahmad Hospital (Innovation Research Laboratory)	MOH - Jaber Al-Ahmad Hospital (Innovation Research Laboratory)	Salman Al-Sabah et al
EPI_ISL_754180	Department for Virology, Molecular Biology and Genome Research, R. G. Lugar Center for Public Health Research, National Center for Disease Control and Public Health (NCDC) of Georgia.	Department for Virology, Molecular Biology and Genome Research, R. G. Lugar Center for Public Health Research, National Center for Disease Control and Public Health (NCDC) of Georgia.	Salome Javashvili et al
EPI_ISL_453359	Liverpool Clinical Laboratories	COVID-19 Genomics UK (COG-UK) Consortium	Sam Haldenby et al
EPI_ISL_1208399	Laboratoire de virologie clinique - Institut Pasteur de Tunis	1-Laboratory of Microbiology, National Reference Lab, Charles Nicolle Hospital; 2-University of Tunis ElManar, Faculty of Medicine of Tunis, LR99ES09, Tunis, Tunisia	Sameh Trabelsi et al
EPI_ISL_1197037	Laboratoire de virologie clinique - Institut Pasteur de Tunis	1-Laboratory of Microbiology, National Reference Lab, Charles Nicolle Hospital; 2-University of Tunis ElManar, Faculty of Medicine of Tunis, LR99ES09, Tunis, Tunisia	Sameh Trabelsi et al
EPI_ISL_766569	Oman-National Influenza Center	Oman-National Influenza Center	Samiha Al-Kharusi et al
EPI_ISL_709542	National Institute of Blood Diseases (NIBD), Molecular Biology Lab	Genomics Lab NIBD	Samina Naz Mukry et al
EPI_ISL_525424	Oman-National Influenza Center	Biotechnology & OMICs Laboratory	Samira Al-Mahruqi et al
EPI_ISL_525425	Oman-National Influenza Center	Biotechnology & OMICs Laboratory	Samira Al-Mahruqi et al
EPI_ISL_525429	Oman-National Influenza Center	Biotechnology & OMICs Laboratory	Samira Al-Mahruqi et al
EPI_ISL_457706	Oman-NIC	Oman-NIC	Samira Al-Maruqi et al
EPI_ISL_457937	Oman-NIC	Oman-NIC	Samira Al-Maruqi et al
EPI_ISL_457991	Oman-NIC	Oman-NIC	Samira Al-Maruqi et al
EPI_ISL_457701	Oman-NIC	Oman-NIC	Samira Al-Maruqi et al
EPI_ISL_457704	Oman-NIC	Oman-NIC	Samira Al-Maruqi et al
EPI_ISL_877428	Institute of Microbiology and Immunology, Faculty of Medicine, University of Ljubljana	Institute of Microbiology and Immunology, Faculty of Medicine, University of Ljubljana	Samo Zakotnik et al
EPI_ISL_877439	Institute of Microbiology and Immunology, Faculty of Medicine, University of Ljubljana	Institute of Microbiology and Immunology, Faculty of Medicine, University of Ljubljana	Samo Zakotnik et al
EPI_ISL_877443	Institute of Microbiology and Immunology, Faculty of Medicine, University of Ljubljana	Institute of Microbiology and Immunology, Faculty of Medicine, University of Ljubljana	Samo Zakotnik et al
EPI_ISL_979246	Institute of Microbiology and Immunology, Faculty of Medicine, University of Ljubljana	Institute of Microbiology and Immunology, Faculty of Medicine, University of Ljubljana	Samo Zakotnik et al
EPI_ISL_635201	Institute of Microbiology and Immunology, Faculty of Medicine, University of Ljubljana	Institute of Microbiology and Immunology, Faculty of Medicine, University of Ljubljana	Samo Zakotnik et al
EPI_ISL_877432	National laboratory of health, environment and food Celje	Institute of Microbiology and Immunology, Faculty of Medicine, University of Ljubljana	Samo Zakotnik et al
EPI_ISL_984640	Laboratory of Virology of Federal Budget Health Care Institution Center of Hygiene and Epidemiology in Arkhangelsk region	Group of Genomics and Postgenomic Technologies of Central Research Institute of Epidemiology	Samoilov AE et al
EPI_ISL_1082311	Laboratory of Virology of Federal Budget Health Care Institution Center of Hygiene and Epidemiology in Arkhangelsk region	Group of Genomics and Postgenomic Technologies of Central Research Institute of Epidemiology	Samoilov AE et al
EPI_ISL_1527385	Laboratory of Virology of Federal Budget Health Care Institution Center of Hygiene and Epidemiology in Arkhangelsk region	Group of Genomics and Postgenomic Technologies of Central Research Institute of Epidemilology	Samoilov AE et al

EPI_ISL_1527368	Molecular diagnostic laboratory of Federal Budget Institution of Science Central Research Institute of Epidemiology of The Federal Service on Customers' Rights Protection and Human Well-being Surveillance	Group of Genomics and Postgenomic Technologies of Central Research Institute of Epidemilology	Samoilov AE et al
EPI_ISL_476259	Hospital da Clínicas da Faculdade de Medicina da Universidade de São Paulo	Instituto de Medicina Tropical da Univesidade de São Paulo	Samples: Ingra Morales Claro et al
EPI_ISL_476204	Hospital da Clínicas da Faculdade de Medicina da Universidade de São Paulo	Instituto de Medicina Tropical da Univesidade de São Paulo	Samples: Ingra Morales Claro et al
EPI_ISL_476266	Hospital da Clínicas da Faculdade de Medicina da Universidade de São Paulo	Instituto de Medicina Tropical da Univesidade de São Paulo	Samples: Ingra Morales Claro et al
EPI_ISL_476444	Hospital da Clínicas da Faculdade de Medicina da Universidade de São Paulo	Instituto de Medicina Tropical da Univesidade de São Paulo	Samples: Ingra Morales Claro et al
EPI_ISL_476446	Hospital da Clínicas da Faculdade de Medicina da Universidade de São Paulo	Instituto de Medicina Tropical da Univesidade de São Paulo	Samples: Ingra Morales Claro et al
EPI_ISL_476450	Hospital da Clínicas da Faculdade de Medicina da Universidade de São Paulo	Instituto de Medicina Tropical da Univesidade de São Paulo	Samples: Ingra Morales Claro et al
EPI_ISL_476475	Hospital da Clínicas da Faculdade de Medicina da Universidade de São Paulo	Instituto de Medicina Tropical da Univesidade de São Paulo	Samples: Ingra Morales Claro et al
EPI_ISL_476484	Hospital da Clínicas da Faculdade de Medicina da Universidade de São Paulo	Instituto de Medicina Tropical da Univesidade de São Paulo	Samples: Ingra Morales Claro et al
EPI_ISL_476486	Hospital da Clínicas da Faculdade de Medicina da Universidade de São Paulo	Instituto de Medicina Tropical da Univesidade de São Paulo	Samples: Ingra Morales Claro et al
EPI_ISL_476224	DB Diagnósticos do Brasil	Instituto de Medicina Tropical da Univesidade de São Paulo	Samples: Nelson Gaburo Jr et al
EPI_ISL_476304	DB Diagnósticos do Brasil	Instituto de Medicina Tropical da Univesidade de São Paulo	Samples: Nelson Gaburo Jr et al
EPI_ISL_476305	DB Diagnósticos do Brasil	Instituto de Medicina Tropical da Univesidade de São Paulo	Samples: Nelson Gaburo Jr et al
EPI_ISL_476182	DB Diagnósticos do Brasil	Instituto de Medicina Tropical da Univesidade de São Paulo	Samples: Nelson Gaburo Jr et al
EPI_ISL_476184	DB Diagnósticos do Brasil	Instituto de Medicina Tropical da Univesidade de São Paulo	Samples: Nelson Gaburo Jr et al
EPI_ISL_476200	DB Diagnósticos do Brasil	Instituto de Medicina Tropical da Univesidade de São Paulo	Samples: Nelson Gaburo Jr et al
EPI_ISL_476218	DB Diagnósticos do Brasil	Instituto de Medicina Tropical da Univesidade de São Paulo	Samples: Nelson Gaburo Jr et al
EPI_ISL_476281	DB Diagnósticos do Brasil	Instituto de Medicina Tropical da Univesidade de São Paulo	Samples: Nelson Gaburo Jr et al
EPI_ISL_476283	DB Diagnósticos do Brasil	Instituto de Medicina Tropical da Univesidade de São Paulo	Samples: Nelson Gaburo Jr et al
EPI_ISL_476288	DB Diagnósticos do Brasil	Instituto de Medicina Tropical da Univesidade de São Paulo	Samples: Nelson Gaburo Jr et al
EPI_ISL_476289	DB Diagnósticos do Brasil	Instituto de Medicina Tropical da Univesidade de São Paulo	Samples: Nelson Gaburo Jr et al
EPI_ISL_476307	DB Diagnósticos do Brasil	Instituto de Medicina Tropical da Univesidade de São Paulo	Samples: Nelson Gaburo Jr et al
EPI_ISL_476331	DB Diagnósticos do Brasil	Instituto de Medicina Tropical da Univesidade de São Paulo	Samples: Nelson Gaburo Jr et al
EPI_ISL_476369	DB Diagnósticos do Brasil	Instituto de Medicina Tropical da Univesidade de São Paulo	Samples: Nelson Gaburo Jr et al
EPI_ISL_476333	DB Diagnósticos do Brasil	Instituto de Medicina Tropical da Univesidade de São Paulo	Samples: Nelson Gaburo Jr et al
EPI_ISL_476336	DB Diagnósticos do Brasil	Instituto de Medicina Tropical da Univesidade de São Paulo	Samples: Nelson Gaburo Jr et al
EPI_ISL_476309	DB Diagnósticos do Brasil	Instituto de Medicina Tropical da Univesidade de São Paulo	Samples: Nelson Gaburo Jr et al
EPI_ISL_476327	DB Diagnósticos do Brasil	Instituto de Medicina Tropical da Univesidade de São Paulo	Samples: Nelson Gaburo Jr et al
EPI_ISL_476328	DB Diagnósticos do Brasil	Instituto de Medicina Tropical da Univesidade de São Paulo	Samples: Nelson Gaburo Jr et al

EPI_ISL_476330	DB Diagnósticos do Brasil	Instituto de Medicina Tropical da Univesidade de São Paulo	Samples: Nelson Gaburo Jr et al
EPI_ISL_476194	DB Diagnósticos do Brasil	Instituto de Medicina Tropical da Univesidade de São Paulo	Samples: Nelson Gaburo Jr et al
EPI_ISL_476290	DB Diagnósticos do Brasil	Instituto de Medicina Tropical da Univesidade de São Paulo	Samples: Nelson Gaburo Jr et al
EPI_ISL_476191	DB Diagnósticos do Brasil	Instituto de Medicina Tropical da Univesidade de São Paulo	Samples: Nelson Gaburo Jr et al
EPI_ISL_476282	DB Diagnósticos do Brasil	Instituto de Medicina Tropical da Univesidade de São Paulo	Samples: Nelson Gaburo Jr et al
EPI_ISL_476311	DB Diagnósticos do Brasil	Instituto de Medicina Tropical da Univesidade de São Paulo	Samples: Nelson Gaburo Jr et al
EPI_ISL_476312	DB Diagnósticos do Brasil	Instituto de Medicina Tropical da Univesidade de São Paulo	Samples: Nelson Gaburo Jr et al
EPI_ISL_476313	DB Diagnósticos do Brasil	Instituto de Medicina Tropical da Univesidade de São Paulo	Samples: Nelson Gaburo Jr et al
EPI_ISL_476314	DB Diagnósticos do Brasil	Instituto de Medicina Tropical da Univesidade de São Paulo	Samples: Nelson Gaburo Jr et al
EPI_ISL_476318	DB Diagnósticos do Brasil	Instituto de Medicina Tropical da Univesidade de São Paulo	Samples: Nelson Gaburo Jr et al
EPI_ISL_476370	DB Diagnósticos do Brasil	Instituto de Medicina Tropical da Univesidade de São Paulo	Samples: Nelson Gaburo Jr et al
EPI_ISL_476178	DB Diagnósticos do Brasil	Instituto de Medicina Tropical da Univesidade de São Paulo	Samples: Nelson Gaburo Jr et al
EPI_ISL_476180	DB Diagnósticos do Brasil	Instituto de Medicina Tropical da Univesidade de São Paulo	Samples: Nelson Gaburo Jr et al
EPI_ISL_476210	DB Diagnósticos do Brasil	Instituto de Medicina Tropical da Univesidade de São Paulo	Samples: Nelson Gaburo Jr et al
EPI_ISL_476326	DB Diagnósticos do Brasil	Instituto de Medicina Tropical da Univesidade de São Paulo	Samples: Nelson Gaburo Jr et al
EPI_ISL_476188	DB Diagnósticos do Brasil	Instituto de Medicina Tropical da Univesidade de São Paulo	Samples: Nelson Gaburo Jr et al
EPI_ISL_476192	DB Diagnósticos do Brasil	Instituto de Medicina Tropical da Univesidade de São Paulo	Samples: Nelson Gaburo Jr et al
EPI_ISL_476316	DB Diagnósticos do Brasil	Instituto de Medicina Tropical da Univesidade de São Paulo	Samples: Nelson Gaburo Jr et al
EPI_ISL_476319	DB Diagnósticos do Brasil	Instituto de Medicina Tropical da Univesidade de São Paulo	Samples: Nelson Gaburo Jr et al
EPI_ISL_476335	DB Diagnósticos do Brasil	Instituto de Medicina Tropical da Univesidade de São Paulo	Samples: Nelson Gaburo Jr et al
EPI_ISL_476278	DB Diagnósticos do Brasil	Instituto de Medicina Tropical da Univesidade de São Paulo	Samples: Nelson Gaburo Jr et al
EPI_ISL_476292	DB Diagnósticos do Brasil	Instituto de Medicina Tropical da Univesidade de São Paulo	Samples: Nelson Gaburo Jr et al
EPI_ISL_476297	DB Diagnósticos do Brasil	Instituto de Medicina Tropical da Univesidade de São Paulo	Samples: Nelson Gaburo Jr et al
EPI_ISL_476317	DB Diagnósticos do Brasil	Instituto de Medicina Tropical da Univesidade de São Paulo	Samples: Nelson Gaburo Jr et al
EPI_ISL_476324	DB Diagnósticos do Brasil	Instituto de Medicina Tropical da Univesidade de São Paulo	Samples: Nelson Gaburo Jr et al
EPI_ISL_476201	DB Diagnósticos do Brasil	Instituto de Medicina Tropical da Univesidade de São Paulo	Samples: Nelson Gaburo Jr et al
EPI_ISL_476211	DB Diagnósticos do Brasil	Instituto de Medicina Tropical da Univesidade de São Paulo	Samples: Nelson Gaburo Jr et al
EPI_ISL_476366	DB Diagnósticos do Brasil	Instituto de Medicina Tropical da Univesidade de São Paulo	Samples: Nelson Gaburo Jr et al
EPI_ISL_897845	University Hospitals of Geneva, Laboratory of Virology	HUG, Laboratory of Virology and the Health2030 Genome Center	Samuel Cordey et al
EPI_ISL_981706	University Hospitals of Geneva, Laboratory of Virology	HUG, Laboratory of Virology and the Health2030 Genome Center	Samuel Cordey et al

EPI_ISL_981707	University Hospitals of Geneva, Laboratory of Virology	HUG, Laboratory of Virology and the Health2030 Genome Center	Samuel Cordey et al
EPI_ISL_981708	University Hospitals of Geneva, Laboratory of Virology	HUG, Laboratory of Virology and the Health2030 Genome Center	Samuel Cordey et al
EPI_ISL_981709	University Hospitals of Geneva, Laboratory of Virology	HUG, Laboratory of Virology and the Health2030 Genome Center	Samuel Cordey et al
EPI_ISL_981710	University Hospitals of Geneva, Laboratory of Virology	HUG, Laboratory of Virology and the Health2030 Genome Center	Samuel Cordey et al
EPI_ISL_981711	University Hospitals of Geneva, Laboratory of Virology	HUG, Laboratory of Virology and the Health2030 Genome Center	Samuel Cordey et al
EPI_ISL_981712	University Hospitals of Geneva, Laboratory of Virology	HUG, Laboratory of Virology and the Health2030 Genome Center	Samuel Cordey et al
EPI_ISL_1040363	University Hospitals of Geneva, Laboratory of Virology	HUG, Laboratory of Virology and the Health2030 Genome Center	Samuel Cordey et al
EPI_ISL_1084930	University Hospitals of Geneva, Laboratory of Virology	HUG, Laboratory of Virology and the Health2030 Genome Center	Samuel Cordey et al
EPI_ISL_1369531	University Hospitals of Geneva, Laboratory of Virology	HUG, Laboratory of Virology and the Health2030 Genome Center	Samuel Cordey et al
EPI_ISL_1533404	University Hospitals of Geneva, Laboratory of Virology	HUG, Laboratory of Virology and the Health2030 Genome Center	Samuel Cordey et al
EPI_ISL_1533406	University Hospitals of Geneva, Laboratory of Virology	HUG, Laboratory of Virology and the Health2030 Genome Center	Samuel Cordey et al
EPI_ISL_1228877	University Hospitals of Geneva, Laboratory of Virology	HUG, Laboratory of Virology and the Health2030 Genome Center	Samuel Cordey et al
EPI_ISL_981713	University Hospitals of Geneva, Laboratory of Virology	HUG, Laboratory of Virology and the Health2030 Genome Center	Samuel Cordey et al
EPI_ISL_981714	University Hospitals of Geneva, Laboratory of Virology	HUG, Laboratory of Virology and the Health2030 Genome Center	Samuel Cordey et al
EPI_ISL_1369541	University Hospitals of Geneva, Laboratory of Virology	HUG, Laboratory of Virology and the Health2030 Genome Center	Samuel Cordey et al
EPI_ISL_1448613	University Hospitals of Geneva, Laboratory of Virology	HUG, Laboratory of Virology and the Health2030 Genome Center	Samuel Cordey et al
EPI_ISL_1665100	National Laboratory for Health, Environment and Food, OMM, Maribor	NLZOH (National Laboratory for Health, Environment and Food) / CISLD (Clinical Institute of Special Laboratory Diagnostics), University Children's Hospital, University Medical Center Ljubljana	Sandra Janezic et al
EPI_ISL_1664822	National Laboratory for Health, Environment and Food, OMM, Celje	NLZOH (National Laboratory for Health, Environment and Food) / CISLD (Clinical Institute of Special Laboratory Diagnostics), University Children's Hospital, University Medical Center Ljubljana	Sandra Janezic et al
EPI_ISL_1664811	National Laboratory for Health, Environment and Food, OMM, Celje	NLZOH (National Laboratory for Health, Environment and Food) / CISLD (Clinical Institute of Special Laboratory Diagnostics), University Children's Hospital, University Medical Center Ljubljana	Sandra Janezic et al
EPI_ISL_1664862	National Laboratory for Health, Environment and Food, OMM, Celje	NLZOH (National Laboratory for Health, Environment and Food) / CISLD (Clinical Institute of Special Laboratory Diagnostics), University Children's Hospital, University Medical Center Ljubljana	Sandra Janezic et al
EPI_ISL_1523487	Laboratoire de santé publique du Québec	Laboratoire de santé publique du Québec	Sandrine Moreira et al
EPI_ISL_1404870	Laboratoire de santé publique du Québec	Laboratoire de santé publique du Québec	Sandrine Moreira et al
EPI_ISL_1241439	Laboratoire de santé publique du Québec	Laboratoire de santé publique du Québec	Sandrine Moreira et al
EPI_ISL_1241665	Laboratoire de santé publique du Québec	Laboratoire de santé publique du Québec	Sandrine Moreira et al
EPI_ISL_1534181	Laboratoire de santé publique du Québec	Laboratoire de santé publique du Québec	Sandrine Moreira et al
EPI_ISL_1167303	Laboratoire de santé publique du Québec	Laboratoire de santé publique du Québec	Sandrine Moreira et al
EPI_ISL_826002	Laboratoire de santé publique du Québec	Laboratoire de santé publique du Québec	Sandrine Moreira et al

EPI_ISL_826072	Laboratoire de santé publique du Québec	Laboratoire de santé publique du Québec	Sandrine Moreira et al
EPI_ISL_826243	Laboratoire de santé publique du Québec	Laboratoire de santé publique du Québec	Sandrine Moreira et al
EPI_ISL_1403122	Laboratoire de santé publique du Québec	Laboratoire de santé publique du Québec	Sandrine Moreira et al
EPI_ISL_1403125	Laboratoire de santé publique du Québec	Laboratoire de santé publique du Québec	Sandrine Moreira et al
EPI_ISL_1365947	Laboratoire de santé publique du Québec	Laboratoire de santé publique du Québec	Sandrine Moreira et al
EPI_ISL_535716	Hôpital de Verdun	Laboratoire de santé publique du Québec	Sandrine Moreira et al
EPI_ISL_450303	Centre hospitalier Anna-Laberge	Laboratoire de santé publique du Québec	Sandrine Moreira et al
EPI_ISL_465693	Hôpital du Suroît	Laboratoire de santé publique du Québec	Sandrine Moreira et al
EPI_ISL_465696	Hôpital Pierre-Boucher	Laboratoire de santé publique du Québec	Sandrine Moreira et al
EPI_ISL_848599	Evandro Chagas Institute	Evandro Chagas Institute	Santos et al
EPI_ISL_848602	Evandro Chagas Institute	Evandro Chagas Institute	Santos et al
EPI_ISL_458139	Evandro Chagas Institute	Evandro Chagas Institute	Santos et al
EPI_ISL_848600	Evandro Chagas Institute	Evandro Chagas Institute	Santos et al
EPI_ISL_848601	Evandro Chagas Institute	Evandro Chagas Institute	Santos et al
EPI_ISL_848627	Evandro Chagas Institute	Evandro Chagas Institute	Santos et al
EPI_ISL_848573	Evandro Chagas Institute	Evandro Chagas Institute	Santos et al
EPI_ISL_848574	Evandro Chagas Institute	Evandro Chagas Institute	Santos et al
EPI_ISL_848612	Evandro Chagas Institute	Evandro Chagas Institute	Santos et al
EPI_ISL_848613	Evandro Chagas Institute	Evandro Chagas Institute	Santos et al
EPI_ISL_925846	LACEN - Laboratório Central de Saúde Pública do Amazonas	Evandro Chagas Institute Virology	Santos et al
EPI_ISL_918532	LACEN - Laboratório Central de Saúde Pública do Amazonas	Evandro Chagas Institute	Santos et al
EPI_ISL_918533	LACEN - Laboratório Central de Saúde Pública do Amazonas	Evandro Chagas Institute	Santos et al
EPI_ISL_1261694	LACEN - Laboratório Central de Saúde Pública do Amazonas	Evandro Chagas Institute	Santos et al
EPI_ISL_918551	LACEN - Laboratório Central de Saúde Pública do Amapa	Evandro Chagas Institute	Santos et al
EPI_ISL_918553	LACEN - Laboratório Central de Saúde Pública do Amapa	Evandro Chagas Institute	Santos et al
EPI_ISL_918554	LACEN - Laboratório Central de Saúde Pública do Amapa	Evandro Chagas Institute	Santos et al
EPI_ISL_918555	LACEN - Laboratório Central de Saúde Pública do Amapa	Evandro Chagas Institute	Santos et al
EPI_ISL_918556	LACEN - Laboratório Central de Saúde Pública do Amapa	Evandro Chagas Institute	Santos et al
EPI_ISL_918557	LACEN - Laboratório Central de Saúde Pública do Amapa	Evandro Chagas Institute	Santos et al
EPI_ISL_918558	LACEN - Laboratório Central de Saúde Pública do Amapa	Evandro Chagas Institute	Santos et al

EPI_ISL_918559	LACEN - Laboratório Central de Saúde Pública do Amapa	Evandro Chagas Institute	Santos et al
EPI_ISL_918560	LACEN - Laboratório Central de Saúde Pública do Amapa	Evandro Chagas Institute	Santos et al
EPI_ISL_918561	LACEN - Laboratório Central de Saúde Pública do Amapa	Evandro Chagas Institute	Santos et al
EPI_ISL_1164985	LACEN - Laboratório Central de Saúde Pública do Amapá	Evandro Chagas Institute	Santos et al
EPI_ISL_1164984	LACEN - Laboratório Central de Saúde Pública do Amapá	Evandro Chagas Institute	Santos et al
EPI_ISL_1164981	LACEN - Laboratório Central de Saúde Pública do Amapá	Evandro Chagas Institute	Santos et al
EPI_ISL_1164982	LACEN - Laboratório Central de Saúde Pública do Amapá	Evandro Chagas Institute	Santos et al
EPI_ISL_1164976	LACEN - Laboratório Central de Saúde Pública do Amapá	Evandro Chagas Institute	Santos et al
EPI_ISL_1261686	LACEN - Laboratório Central de Saúde Pública do Amapá	Evandro Chagas Institute	Santos et al
EPI_ISL_1261696	LACEN - Laboratório Central de Saúde Pública do Amapá	Evandro Chagas Institute	Santos et al
EPI_ISL_1261692	LACEN - Laboratório Central de Saúde Pública do Amapá	Evandro Chagas Institute	Santos et al
EPI_ISL_1261695	LACEN - Laboratório Central de Saúde Pública do Amapá	Evandro Chagas Institute	Santos et al
EPI_ISL_1261688	LACEN - Laboratório Central de Saúde Pública do Amapá	Evandro Chagas Institute	Santos et al
EPI_ISL_1261689	LACEN - Laboratório Central de Saúde Pública do Amapá	Evandro Chagas Institute	Santos et al
EPI_ISL_450873	Evandro Chagas Institute	Evandro Chagas Institute	Santos et al
EPI_ISL_524792	Evandro Chagas Institute	Evandro Chagas Institute	Santos et al
EPI_ISL_458138	Evandro Chagas Institute	Evandro Chagas Institute	Santos et al
EPI_ISL_458142	Evandro Chagas Institute	Evandro Chagas Institute	Santos et al
EPI_ISL_524795	Evandro Chagas Institute	Evandro Chagas Institute	Santos et al
EPI_ISL_458144	Evandro Chagas Institute	Evandro Chagas Institute	Santos et al
EPI_ISL_458143	Evandro Chagas Institute	Evandro Chagas Institute	Santos et al
EPI_ISL_458145	Evandro Chagas Institute	Evandro Chagas Institute	Santos et al
EPI_ISL_524796	Evandro Chagas Institute	Evandro Chagas Institute	Santos et al
EPI_ISL_524784	Evandro Chagas Institute	Evandro Chagas Institute	Santos et al
EPI_ISL_524793	Evandro Chagas Institute	Evandro Chagas Institute	Santos et al
EPI_ISL_848584	Evandro Chagas Institute	Evandro Chagas Institute	Santos et al
EPI_ISL_848578	Evandro Chagas Institute	Evandro Chagas Institute	Santos et al
EPI_ISL_848579	Evandro Chagas Institute	Evandro Chagas Institute	Santos et al
EPI_ISL_848577	Evandro Chagas Institute	Evandro Chagas Institute	Santos et al
EPI_ISL_848575	Evandro Chagas Institute	Evandro Chagas Institute	Santos et al

EPI_ISL_848576	Evandro Chagas Institute	Evandro Chagas Institute	Santos et al
EPI_ISL_1261697	LACEN - Laboratório Central de Saúde Pública do Ceará	Evandro Chagas Institute	Santos et al
EPI_ISL_918537	LACEN - Laboratório Central de Saúde Pública do Ceara	Evandro Chagas Institute	Santos et al
EPI_ISL_918538	LACEN - Laboratório Central de Saúde Pública do Ceara	Evandro Chagas Institute	Santos et al
EPI_ISL_918539	LACEN - Laboratório Central de Saúde Pública do Ceara	Evandro Chagas Institute	Santos et al
EPI_ISL_918540	LACEN - Laboratório Central de Saúde Pública do Ceara	Evandro Chagas Institute	Santos et al
EPI_ISL_918541	LACEN - Laboratório Central de Saúde Pública do Ceara	Evandro Chagas Institute	Santos et al
EPI_ISL_918542	LACEN - Laboratório Central de Saúde Pública do Ceara	Evandro Chagas Institute	Santos et al
EPI_ISL_918543	LACEN - Laboratório Central de Saúde Pública do Ceara	Evandro Chagas Institute	Santos et al
EPI_ISL_918544	LACEN - Laboratório Central de Saúde Pública do Ceara	Evandro Chagas Institute	Santos et al
EPI_ISL_1164980	LACEN - Laboratório Central de Saúde Pública do Ceará	Evandro Chagas Institute	Santos et al
EPI_ISL_1164986	LACEN - Laboratório Central de Saúde Pública do Ceará	Evandro Chagas Institute	Santos et al
EPI_ISL_1164995	LACEN - Laboratório Central de Saúde Pública do Ceará	Evandro Chagas Institute	Santos et al
EPI_ISL_1164993	LACEN - Laboratório Central de Saúde Pública do Ceará	Evandro Chagas Institute	Santos et al
EPI_ISL_1164973	LACEN - Laboratório Central de Saúde Pública do Ceará	Evandro Chagas Institute	Santos et al
EPI_ISL_1164970	LACEN - Laboratório Central de Saúde Pública do Ceará	Evandro Chagas Institute	Santos et al
EPI_ISL_1164971	LACEN - Laboratório Central de Saúde Pública do Ceará	Evandro Chagas Institute	Santos et al
EPI_ISL_1261693	LACEN - Laboratório Central de Saúde Pública do Ceará	Evandro Chagas Institute	Santos et al
EPI_ISL_1261684	LACEN - Laboratório Central de Saúde Pública do Ceará	Evandro Chagas Institute	Santos et al
EPI_ISL_1164996	LACEN - Laboratório Central de Saúde Pública do Maranhao	Evandro Chagas Institute	Santos et al
EPI_ISL_1164998	LACEN - Laboratório Central de Saúde Pública do Maranhao	Evandro Chagas Institute	Santos et al
EPI_ISL_524797	Evandro Chagas Institute	Evandro Chagas Institute	Santos et al
EPI_ISL_848626	Evandro Chagas Institute	Evandro Chagas Institute	Santos et al
EPI_ISL_524791	Evandro Chagas Institute	Evandro Chagas Institute	Santos et al
EPI_ISL_458149	Evandro Chagas Institute	Evandro Chagas Institute	Santos et al
EPI_ISL_524788	Evandro Chagas Institute	Evandro Chagas Institute	Santos et al
EPI_ISL_524790	Evandro Chagas Institute	Evandro Chagas Institute	Santos et al
EPI_ISL_524794	Evandro Chagas Institute	Evandro Chagas Institute	Santos et al
EPI_ISL_524789	Evandro Chagas Institute	Evandro Chagas Institute	Santos et al
EPI_ISL_524799	Evandro Chagas Institute	Evandro Chagas Institute	Santos et al

EPI_ISL_848580	Evandro Chagas Institute	Evandro Chagas Institute	Santos et al
EPI_ISL_848581	Evandro Chagas Institute	Evandro Chagas Institute	Santos et al
EPI_ISL_1086373	LACEN - Laboratório Central de Saúde Pública do Maranhao	Evandro Chagas Institute	Santos et al
EPI_ISL_918514	Evandro Chagas Institute	Evandro Chagas Institute	Santos et al
EPI_ISL_918515	LACEN - Laboratório Central de Saúde Pública do Para	Evandro Chagas Institute	Santos et al
EPI_ISL_918516	LACEN - Laboratório Central de Saúde Pública do Para	Evandro Chagas Institute	Santos et al
EPI_ISL_918517	LACEN - Laboratório Central de Saúde Pública do Para	Evandro Chagas Institute	Santos et al
EPI_ISL_918518	Evandro Chagas Institute	Evandro Chagas Institute	Santos et al
EPI_ISL_918519	Evandro Chagas Institute	Evandro Chagas Institute	Santos et al
EPI_ISL_918520	Evandro Chagas Institute	Evandro Chagas Institute	Santos et al
EPI_ISL_918521	Evandro Chagas Institute	Evandro Chagas Institute	Santos et al
EPI_ISL_918523	LACEN - Laboratório Central de Saúde Pública do Para	Evandro Chagas Institute	Santos et al
EPI_ISL_918524	LACEN - Laboratório Central de Saúde Pública do Para	Evandro Chagas Institute	Santos et al
EPI_ISL_918525	LACEN - Laboratório Central de Saúde Pública do Para	Evandro Chagas Institute	Santos et al
EPI_ISL_918527	LACEN - Laboratório Central de Saúde Pública do Para	Evandro Chagas Institute	Santos et al
EPI_ISL_918528	LACEN - Laboratório Central de Saúde Pública do Para	Evandro Chagas Institute	Santos et al
EPI_ISL_918529	LACEN - Laboratório Central de Saúde Pública do Para	Evandro Chagas Institute	Santos et al
EPI_ISL_918545	LACEN - Laboratório Central de Saúde Pública do Para	Evandro Chagas Institute	Santos et al
EPI_ISL_918546	LACEN - Laboratório Central de Saúde Pública do Para	Evandro Chagas Institute	Santos et al
EPI_ISL_918547	LACEN - Laboratório Central de Saúde Pública do Para	Evandro Chagas Institute	Santos et al
EPI_ISL_918548	LACEN - Laboratório Central de Saúde Pública do Para	Evandro Chagas Institute	Santos et al
EPI_ISL_918549	LACEN - Laboratório Central de Saúde Pública do Para	Evandro Chagas Institute	Santos et al
EPI_ISL_904120	LACEN - Laboratório Central de Saúde Pública do Pará	Evandro Chagas Institute	Santos et al
EPI_ISL_904121	LACEN - Laboratório Central de Saúde Pública do Pará	Evandro Chagas Institute	Santos et al
EPI_ISL_918550	LACEN - Laboratório Central de Saúde Pública do Para	Evandro Chagas Institute	Santos et al
EPI_ISL_918552	LACEN - Laboratório Central de Saúde Pública do Para	Evandro Chagas Institute	Santos et al
EPI_ISL_1164972	LACEN - Laboratório Central de Saúde Pública do Pará	Evandro Chagas Institute	Santos et al
EPI_ISL_1164978	LACEN - Laboratório Central de Saúde Pública do Pará	Evandro Chagas Institute	Santos et al
EPI_ISL_1164983	LACEN - Laboratório Central de Saúde Pública do Pará	Evandro Chagas Institute	Santos et al
EPI_ISL_1164974	LACEN - Laboratório Central de Saúde Pública do Pará	Evandro Chagas Institute	Santos et al

EPI_ISL_1164975	LACEN - Laboratório Central de Saúde Pública do Pará	Evandro Chagas Institute	Santos et al
EPI_ISL_1261691	Laboratório Paulo C. Azevedo	Evandro Chagas Institute	Santos et al
EPI_ISL_450874	Evandro Chagas Institute	Evandro Chagas Institute	Santos et al
EPI_ISL_458140	Evandro Chagas Institute	Evandro Chagas Institute	Santos et al
EPI_ISL_848624	Evandro Chagas Institute	Evandro Chagas Institute	Santos et al
EPI_ISL_524787	Evandro Chagas Institute	Evandro Chagas Institute	Santos et al
EPI_ISL_458146	Evandro Chagas Institute	Evandro Chagas Institute	Santos et al
EPI_ISL_458147	Evandro Chagas Institute	Evandro Chagas Institute	Santos et al
EPI_ISL_458141	Evandro Chagas Institute	Evandro Chagas Institute	Santos et al
EPI_ISL_524783	Evandro Chagas Institute	Evandro Chagas Institute	Santos et al
EPI_ISL_524786	Evandro Chagas Institute	Evandro Chagas Institute	Santos et al
EPI_ISL_524785	Evandro Chagas Institute	Evandro Chagas Institute	Santos et al
EPI_ISL_524800	Evandro Chagas Institute	Evandro Chagas Institute	Santos et al
EPI_ISL_1063789	Evandro Chagas Institute	Evandro Chagas Institute Virology	Santos et al
EPI_ISL_848622	Evandro Chagas Institute	Evandro Chagas Institute	Santos et al
EPI_ISL_848628	Evandro Chagas Institute	Evandro Chagas Institute	Santos et al
EPI_ISL_848629	Evandro Chagas Institute	Evandro Chagas Institute	Santos et al
EPI_ISL_848597	Evandro Chagas Institute	Evandro Chagas Institute	Santos et al
EPI_ISL_848596	Evandro Chagas Institute	Evandro Chagas Institute	Santos et al
EPI_ISL_848625	Evandro Chagas Institute	Evandro Chagas Institute	Santos et al
EPI_ISL_848598	Evandro Chagas Institute	Evandro Chagas Institute	Santos et al
EPI_ISL_848617	Evandro Chagas Institute	Evandro Chagas Institute	Santos et al
EPI_ISL_848618	Evandro Chagas Institute	Evandro Chagas Institute	Santos et al
EPI_ISL_848620	Evandro Chagas Institute	Evandro Chagas Institute	Santos et al
EPI_ISL_848593	Evandro Chagas Institute	Evandro Chagas Institute	Santos et al
EPI_ISL_848591	Evandro Chagas Institute	Evandro Chagas Institute	Santos et al
EPI_ISL_848588	Evandro Chagas Institute	Evandro Chagas Institute	Santos et al
EPI_ISL_848590	Evandro Chagas Institute	Evandro Chagas Institute	Santos et al
EPI_ISL_848571	Evandro Chagas Institute	Evandro Chagas Institute	Santos et al
EPI_ISL_848583	Evandro Chagas Institute	Evandro Chagas Institute	Santos et al

EPI_ISL_848615	Evandro Chagas Institute	Evandro Chagas Institute	Santos et al
EPI_ISL_848582	Evandro Chagas Institute	Evandro Chagas Institute	Santos et al
EPI_ISL_848611	Evandro Chagas Institute	Evandro Chagas Institute	Santos et al
EPI_ISL_848568	Evandro Chagas Institute	Evandro Chagas Institute	Santos et al
EPI_ISL_848567	Evandro Chagas Institute	Evandro Chagas Institute	Santos et al
EPI_ISL_848572	Evandro Chagas Institute	Evandro Chagas Institute	Santos et al
EPI_ISL_848566	Evandro Chagas Institute	Evandro Chagas Institute	Santos et al
EPI_ISL_848614	Evandro Chagas Institute	Evandro Chagas Institute	Santos et al
EPI_ISL_848570	Evandro Chagas Institute	Evandro Chagas Institute	Santos et al
EPI_ISL_848569	Evandro Chagas Institute	Evandro Chagas Institute	Santos et al
EPI_ISL_848564	Evandro Chagas Institute	Evandro Chagas Institute	Santos et al
EPI_ISL_848609	Evandro Chagas Institute	Evandro Chagas Institute	Santos et al
EPI_ISL_848561	Evandro Chagas Institute	Evandro Chagas Institute	Santos et al
EPI_ISL_848565	Evandro Chagas Institute	Evandro Chagas Institute	Santos et al
EPI_ISL_848562	Evandro Chagas Institute	Evandro Chagas Institute	Santos et al
EPI_ISL_848563	Evandro Chagas Institute	Evandro Chagas Institute	Santos et al
EPI_ISL_848608	Evandro Chagas Institute	Evandro Chagas Institute	Santos et al
EPI_ISL_848555	Evandro Chagas Institute	Evandro Chagas Institute	Santos et al
EPI_ISL_848607	Evandro Chagas Institute	Evandro Chagas Institute	Santos et al
EPI_ISL_848559	Evandro Chagas Institute	Evandro Chagas Institute	Santos et al
EPI_ISL_848560	Evandro Chagas Institute	Evandro Chagas Institute	Santos et al
EPI_ISL_848558	Evandro Chagas Institute	Evandro Chagas Institute	Santos et al
EPI_ISL_848606	Evandro Chagas Institute	Evandro Chagas Institute	Santos et al
EPI_ISL_848557	Evandro Chagas Institute	Evandro Chagas Institute	Santos et al
EPI_ISL_1164994	LACEN - Laboratório Central de Saúde Pública do Paraiba	Evandro Chagas Institute	Santos et al
EPI_ISL_1164992	LACEN - Laboratório Central de Saúde Pública do Paraiba	Evandro Chagas Institute	Santos et al
EPI_ISL_1164989	LACEN - Laboratório Central de Saúde Pública do Paraiba	Evandro Chagas Institute	Santos et al
EPI_ISL_1164991	LACEN - Laboratório Central de Saúde Pública do Paraiba	Evandro Chagas Institute	Santos et al
EPI_ISL_524801	Evandro Chagas Institute	Evandro Chagas Institute	Santos et al
EPI_ISL_1086377	LACEN - Laboratório Central de Saúde Pública do Paraiba	Evandro Chagas Institute	Santos et al

EPI_ISL_1261699	LACEN - Laboratório Central de Saúde Pública de Pernambuco	Evandro Chagas Institute	Santos et al
EPI_ISL_1261698	LACEN - Laboratório Central de Saúde Pública de Pernambuco	Evandro Chagas Institute	Santos et al
EPI_ISL_524798	Evandro Chagas Institute	Evandro Chagas Institute	Santos et al
EPI_ISL_1164977	LACEN - Laboratório Central de Saúde Pública do Rio Grande do Norte	Evandro Chagas Institute	Santos et al
EPI_ISL_1164997	LACEN - Laboratório Central de Saúde Pública do Rio Grande do Norte	Evandro Chagas Institute	Santos et al
EPI_ISL_1086376	LACEN - Laboratório Central de Saúde Pública do Rio Grande do Norte	Evandro Chagas Institute	Santos et al
EPI_ISL_1086375	LACEN - Laboratório Central de Saúde Pública do Rio Grande do Norte	Evandro Chagas Institute	Santos et al
EPI_ISL_918513	LACEN - Laboratório Central de Saúde Pública do Roraima	Evandro Chagas Institute	Santos et al
EPI_ISL_1261687	LACEN - Laboratório Central de Saúde Pública de Roraima	Evandro Chagas Institute	Santos et al
EPI_ISL_848586	Evandro Chagas Institute	Evandro Chagas Institute	Santos et al
EPI_ISL_848610	Evandro Chagas Institute	Evandro Chagas Institute	Santos et al
EPI_ISL_751235	Pathogen Genomics Lab King Abdullah University of Science and Technology (KAUST)	Pathogen Genomics Lab King Abdullah University of Science and Technology(KAUST)	Sara Mfarrej et al
EPI_ISL_751230	Pathogen Genomics Lab King Abdullah University of Science and Technology (KAUST)	Pathogen Genomics Lab King Abdullah University of Science and Technology(KAUST)	Sara Mfarrej et al
EPI_ISL_751217	Pathogen Genomics Lab King Abdullah University of Science and Technology (KAUST)	Pathogen Genomics Lab King Abdullah University of Science and Technology(KAUST)	Sara Mfarrej et al
EPI_ISL_678215	Pathogen Genomics Lab King Abdullah University of Science and Technology (KAUST)	Pathogen Genomics Lab King Abdullah University of Science and Technology(KAUST)	Sara Mfarrej et al
EPI_ISL_678182	Pathogen Genomics Lab King Abdullah University of Science and Technology (KAUST)	Pathogen Genomics Lab King Abdullah University of Science and Technology(KAUST)	Sara Mfarrej et al
EPI_ISL_678220	Pathogen Genomics Lab King Abdullah University of Science and Technology (KAUST)	Pathogen Genomics Lab King Abdullah University of Science and Technology(KAUST)	Sara Mfarrej et al
EPI_ISL_508822	Florida Bureau of Public Health Laboratories	Florida Bureau of Public Health Laboratories	Sarah Schmedes et al
EPI_ISL_514175	Florida Bureau of Public Health Laboratories	Florida Bureau of Public Health Laboratories	Sarah Schmedes et al
EPI_ISL_514209	Florida Bureau of Public Health Laboratories	Florida Bureau of Public Health Laboratories	Sarah Schmedes et al
EPI_ISL_594371	Florida Bureau of Public Health Laboratories	Florida Bureau of Public Health Laboratories	Sarah Schmedes et al
EPI_ISL_594383	Florida Bureau of Public Health Laboratories	Florida Bureau of Public Health Laboratories	Sarah Schmedes et al
EPI_ISL_848773	Florida Bureau of Public Health Laboratories	Florida Bureau of Public Health Laboratories	Sarah Schmedes et al
EPI_ISL_1532786	Translational Health Science and Technology Institute -ESIC medical college and hospital, Faridabad	THSTI Bioassay laboratory	Saurabh Kumar et al
EPI_ISL_476832	Medical Biology Department, Kocaeli University	Medical Genetics Department, Kocaeli University	Savli H et al
EPI_ISL_907077	Biology, MCL	Biology, MCL	Seadawy et al
EPI_ISL_907085	Biology, MCL	Biology, MCL	Seadawy et al
EPI_ISL_1531880	Centro de Diagnostico COVID-19 UABC Tijuana	Andersen lab at Scripps Research	SEARCH Alliance San Diego with Idanya R

EPI_ISL_1531899	Centro de Diagnostico COVID-19 UABC Tijuana	Andersen lab at Scripps Research	SEARCH Alliance San Diego with Idanya R
EPI_ISL_1531917	Centro de Diagnostico COVID-19 UABC Tijuana	Andersen lab at Scripps Research	SEARCH Alliance San Diego with Idanya R
EPI_ISL_635488	Centro de Diagn√≥stico COVID-19 UABC Tijuana	Andersen lab at Scripps Research	SEARCH Alliance San Diego with Idanya R
EPI_ISL_635549	Centro de Diagn√≥stico COVID-19 UABC Tijuana	Andersen lab at Scripps Research	SEARCH Alliance San Diego with Idanya R
EPI_ISL_730223	Genomica Lab Molecular, M√©xico	Andersen lab at Scripps Research	SEARCH Alliance San Diego with Jonathan
EPI_ISL_563576	Microbiological Diagnostic Unit - Public Health Laboratory (MDU-PHL)	MDU-PHL	Seemann et al
EPI_ISL_564763	Microbiological Diagnostic Unit - Public Health Laboratory (MDU-PHL)	MDU-PHL	Seemann et al
EPI_ISL_565504	Microbiological Diagnostic Unit - Public Health Laboratory (MDU-PHL)	MDU-PHL	Seemann et al
EPI_ISL_419731	Microbiological Diagnostic Unit Public Health Laboratory	Microbiological Diagnostic Unit Public Health Laboratory	Seemann T. et al
EPI_ISL_592747	Microbiological Diagnostic Unit - Public Health Laboratory (MDU-PHL)	MDU-PHL	Seemann T. et al
EPI_ISL_641220	Microbiological Diagnostic Unit - Public Health Laboratory (MDU-PHL)	MDU-PHL	Seemann T. et al
EPI_ISL_521641	Microbiological Diagnostic Unit - Public Health Laboratory (MDU-PHL)	MDU-PHL	Seemann T. et al
EPI_ISL_520615	Microbiological Diagnostic Unit - Public Health Laboratory (MDU-PHL)	MDU-PHL	Seemann T. et al
EPI_ISL_430644	Microbiological Diagnostic Unit Public Health Laboratory	Microbiological Diagnostic Unit Public Health Laboratory	Seemann T. et al
EPI_ISL_468071	Child Health Research Foundation	Child Health Research Foundation	Senjuti Saha et al
EPI_ISL_468072	Child Health Research Foundation	Child Health Research Foundation	Senjuti Saha et al
EPI_ISL_492028	Child Health Research Foundation	Child Health Research Foundation	Senjuti Saha et al
EPI_ISL_625468	Child Health Research Foundation	Child Health Research Foundation	Senjuti Saha et al
EPI_ISL_700339	Child Health Research Foundation	Child Health Research Foundation	Senjuti Saha et al
EPI_ISL_700332	Child Health Research Foundation	Child Health Research Foundation	Senjuti Saha et al
EPI_ISL_700344	Child Health Research Foundation	Child Health Research Foundation	Senjuti Saha et al
EPI_ISL_700345	Child Health Research Foundation	Child Health Research Foundation	Senjuti Saha et al
EPI_ISL_700347	Child Health Research Foundation	Child Health Research Foundation	Senjuti Saha et al
EPI_ISL_906091	Shimantik Pathology and Diagnostic Center	Child Health Research Foundation	Senjuti Saha et al
EPI_ISL_591070	College of Veterinary Medicine, Chungnam National University	College of Veterinary Medicine, Chungnam National University	Seo et al
EPI_ISL_428901	State Research Center of Virology and Biotechnology VECTOR, Department of Collection of Microorganisms	State Research Center of Virology and Biotechnology VECTOR, Department of Collection of Microorganisms	Sergey A. Bodnev et al
EPI_ISL_428898	State Research Center of Virology and Biotechnology VECTOR, Department of Collection of Microorganisms	State Research Center of Virology and Biotechnology VECTOR, Department of Collection of Microorganisms	Sergey A. Bodnev et al
EPI_ISL_428897	State Research Center of Virology and Biotechnology VECTOR, Department of Collection of Microorganisms	State Research Center of Virology and Biotechnology VECTOR, Department of Collection of Microorganisms	Sergey A. Bodnev et al
EPI_ISL_985408	Hospital General Universitario Gregorio Marañón	Hospital General Universitario Gregorio Marañón	Sergio Buenestado Serrano et al

ubi Serafin Higuera et al

ubi Serafin Higuera et al

ubí Serafín Higuera et al

ubí Serafín Higuera et al

Gonzalez Garcia et al

EPI_ISL_985409	Hospital General Universitario Gregorio Marañón	Hospital General Universitario Gregorio Marañón	Sergio Buenestado Serrano et al
EPI_ISL_471158	MRCG at LSHTM Genomics lab	MRCG at LSHTM Genomics lab	Sesay et al
EPI_ISL_471159	MRCG at LSHTM Genomics lab	MRCG at LSHTM Genomics lab	Sesay et al
EPI_ISL_471160	MRCG at LSHTM Genomics lab	MRCG at LSHTM Genomics lab	Sesay et al
EPI_ISL_471165	MRCG at LSHTM Genomics lab	MRCG at LSHTM Genomics lab	Sesay et al
EPI_ISL_428855	MRCG at LSHTM Geomics lab	MRCG at LSHTM Genomics lab	Sesay et al
EPI_ISL_428856	MRCG at LSHTM Genomics Lab	MRCG at LSHTM Genomics lab	Sesay et al
EPI_ISL_954776	National Institute of Laboratory Medicine and Referral Center	Genomic Research Lab, BCSIR	Shahina Akter et al
EPI_ISL_1233953	Jamil-ur-Rahman Center for Genome Research, Dr. Panjwani Center for Molecular Medicine and Drug Research	Jamil-ur-Rahman Center for Genome Research, Dr. Panjwani Center for Molecular Medicine and Drug Research	Shakeel et al
EPI_ISL_779264	Jamil-ur-Rahman Center for Genome Research, Dr. Panjwani Center for Molecular Medicine and Drug Research	Jamil-ur-Rahman Center for Genome Research, Dr. Panjwani Center for Molecular Medicine and Drug Research	Shakeel et al
EPI_ISL_779255	Jamil-ur-Rahman Center for Genome Research, Dr. Panjwani Center for Molecular Medicine and Drug Research	Jamil-ur-Rahman Center for Genome Research, Dr. Panjwani Center for Molecular Medicine and Drug Research	Shakeel et al
EPI_ISL_779256	Jamil-ur-Rahman Center for Genome Research, Dr. Panjwani Center for Molecular Medicine and Drug Research	Jamil-ur-Rahman Center for Genome Research, Dr. Panjwani Center for Molecular Medicine and Drug Research	Shakeel et al
EPI_ISL_779258	Jamil-ur-Rahman Center for Genome Research, Dr. Panjwani Center for Molecular Medicine and Drug Research	Jamil-ur-Rahman Center for Genome Research, Dr. Panjwani Center for Molecular Medicine and Drug Research	Shakeel et al
EPI_ISL_779290	Jamil-ur-Rahman Center for Genome Research, Dr. Panjwani Center for Molecular Medicine and Drug Research	Jamil-ur-Rahman Center for Genome Research, Dr. Panjwani Center for Molecular Medicine and Drug Research	Shakeel et al
EPI_ISL_779272	Jamil-ur-Rahman Center for Genome Research, Dr. Panjwani Center for Molecular Medicine and Drug Research	Jamil-ur-Rahman Center for Genome Research, Dr. Panjwani Center for Molecular Medicine and Drug Research	Shakeel et al
EPI_ISL_779276	Jamil-ur-Rahman Center for Genome Research, Dr. Panjwani Center for Molecular Medicine and Drug Research	Jamil-ur-Rahman Center for Genome Research, Dr. Panjwani Center for Molecular Medicine and Drug Research	Shakeel et al
EPI_ISL_779257	Jamil-ur-Rahman Center for Genome Research, Dr. Panjwani Center for Molecular Medicine and Drug Research	Jamil-ur-Rahman Center for Genome Research, Dr. Panjwani Center for Molecular Medicine and Drug Research	Shakeel et al
EPI_ISL_779289	Jamil-ur-Rahman Center for Genome Research, Dr. Panjwani Center for Molecular Medicine and Drug Research	Jamil-ur-Rahman Center for Genome Research, Dr. Panjwani Center for Molecular Medicine and Drug Research	Shakeel et al
EPI_ISL_779282	Jamil-ur-Rahman Center for Genome Research, Dr. Panjwani Center for Molecular Medicine and Drug Research	Jamil-ur-Rahman Center for Genome Research, Dr. Panjwani Center for Molecular Medicine and Drug Research	Shakeel et al
EPI_ISL_779263	Jamil-ur-Rahman Center for Genome Research, Dr. Panjwani Center for Molecular Medicine and Drug Research	Jamil-ur-Rahman Center for Genome Research, Dr. Panjwani Center for Molecular Medicine and Drug Research	Shakeel et al
EPI_ISL_451958	Jamil-ur-Rahman Center for Genome Research, Dr. Panjwani Center for Molecular Medicine and Drug Research, International Center for Chemical and Biological Sciences, University of Karachi	Jamil-ur-Rahman Center for Genome Research, Dr. Panjwani Center for Molecular Medicine and Drug Research, International Center for Chemical and Biological Sciences, University of Karachi	Shakeel et al
EPI_ISL_413015	Public Health Ontario Laboratory	National Microbiology Laboratory	Shari Tyson et al
EPI_ISL_678079	Pathogen Genomics Lab King Abdullah University of Science and Technology (KAUST)	Pathogen Genomics Lab King Abdullah University of Science and Technology(KAUST)	Sharif Hala et al
EPI_ISL_510531	Communicable Disease Laboratory, Public Health Directorate	Communicable Disease Laboratory, Public Health Directorate	Shehab et al

EPI_ISL_416344	Shanghai Public Health Clinical Center, Shanghai Medical College, Fudan University	National Research Center for Translational Medicine (Shanghai), Ruijin Hospital affiliated to Shanghai Jiao Tong University School of Medicine & Shanghai Public Health Clinical Center	Shengyue Wang et al
EPI_ISL_416346	Shanghai Public Health Clinical Center, Shanghai Medical College, Fudan University	National Research Center for Translational Medicine (Shanghai), Ruijin Hospital affiliated to Shanghai Jiao Tong University School of Medicine & Shanghai Public Health Clinical Center	Shengyue Wang et al
EPI_ISL_416356	Shanghai Public Health Clinical Center, Shanghai Medical College, Fudan University	National Research Center for Translational Medicine (Shanghai), Ruijin Hospital affiliated to Shanghai Jiao Tong University School of Medicine & Shanghai Public Health Clinical Center	Shengyue Wang et al
EPI_ISL_416371	Shanghai Public Health Clinical Center, Shanghai Medical College, Fudan University	National Research Center for Translational Medicine (Shanghai), Ruijin Hospital affiliated to Shanghai Jiao Tong University School of Medicine & Shanghai Public Health Clinical Center	Shengyue Wang et al
EPI_ISL_416385	Shanghai Public Health Clinical Center, Shanghai Medical College, Fudan University	National Research Center for Translational Medicine (Shanghai), Ruijin Hospital affiliated to Shanghai Jiao Tong University School of Medicine & Shanghai Public Health Clinical Center	Shengyue Wang et al
EPI_ISL_1365364	Laboratory of virology, National center of expertise	RSE National Center for Biotechnology and RSE National Center of Expertise	Shevtsov Alexandr et al
EPI_ISL_1334570	Laboratory of virology, National center of expertise	RSE National Center for Biotechnology and RSE National Center of Expertise	Shevtsov Alexandr et al
EPI_ISL_1365365	Laboratory of virology, National center of expertise	RSE National Center for Biotechnology and RSE National Center of Expertise	Shevtsov Alexandr et al
EPI_ISL_1364617	Laboratory of virology, National center of expertise	RSE National Center for Biotechnology and RSE National Center of Expertise	Shevtsov Alexandr et al
EPI_ISL_1364865	Laboratory of virology, National center of expertise	RSE National Center for Biotechnology and RSE National Center of Expertise	Shevtsov Alexandr et al
EPI_ISL_1364864	Laboratory of virology, National center of expertise	RSE National Center for Biotechnology and RSE National Center of Expertise	Shevtsov Alexandr et al
EPI_ISL_1364879	Laboratory of virology, National center of expertise	RSE National Center for Biotechnology and RSE National Center of Expertise	Shevtsov Alexandr et al
EPI_ISL_1334572	Laboratory of virology, National center of expertise	RSE National Center for Biotechnology and RSE National Center of Expertise	Shevtsov Alexandr et al
EPI_ISL_1334580	Laboratory of virology, National center of expertise	RSE National Center for Biotechnology and RSE National Center of Expertise	Shevtsov Alexandr et al
EPI_ISL_1364880	Laboratory of virology, National center of expertise	RSE National Center for Biotechnology and RSE National Center of Expertise	Shevtsov Alexandr et al
EPI_ISL_1448018	Laboratory of virology, National center of expertise	RSE National Center for Biotechnology and RSE National Center of Expertise	Shevtsov Alexandr et al
EPI_ISL_1334569	Laboratory of virology, National center of expertise	RSE National Center for Biotechnology and RSE National Center of Expertise	Shevtsov Alexandr et al
EPI_ISL_1334574	Laboratory of virology, National center of expertise	RSE National Center for Biotechnology and RSE National Center of Expertise	Shevtsov Alexandr et al
EPI_ISL_1448020	Laboratory of virology, National center of expertise	RSE National Center for Biotechnology and RSE National Center of Expertise	Shevtsov Alexandr et al
EPI_ISL_408489	Department of Laboratory Medicine, National Taiwan University Hospital	Microbial Genomics Core Lab, National Taiwan University Centers of Genomic and Precision Medicine	Shiou-Hwei Yeh et al

EPI_ISL_447622	Department of Laboratory Medicine, National Taiwan University Hospital	Microbial Genomics Core Lab, National Taiwan University Centers of Genomic and Precision Medicine	Shiou-Hwei Yeh et al
EPI_ISL_534336	Department of Laboratory Medicine, National Taiwan University Hospital	Microbial Genomics Core Lab, National Taiwan University Centers of Genomic and Precision Medicine	Shiou-Hwei Yeh et al
EPI_ISL_693303	Department of Laboratory Medicine, National Taiwan University Hospital	Microbial Genomics Core Lab, National Taiwan University Centers of Genomic and Precision Medicine	Shiou-Hwei Yeh et al
EPI_ISL_693305	Department of Laboratory Medicine, National Taiwan University Hospital	Microbial Genomics Core Lab, National Taiwan University Centers of Genomic and Precision Medicine	Shiou-Hwei Yeh et al
EPI_ISL_693306	Department of Laboratory Medicine, National Taiwan University Hospital	Microbial Genomics Core Lab, National Taiwan University Centers of Genomic and Precision Medicine	Shiou-Hwei Yeh et al
EPI_ISL_738064	Department of Laboratory Medicine, National Taiwan University Hospital	Microbial Genomics Core Lab, National Taiwan University Centers of Genomic and Precision Medicine	Shiou-Hwei Yeh et al
EPI_ISL_740547	Department of Laboratory Medicine, National Taiwan University Hospital	Microbial Genomics Core Lab, National Taiwan University Centers of Genomic and Precision Medicine	Shiou-Hwei Yeh et al
EPI_ISL_872591	Department of Laboratory Medicine, National Taiwan University Hospital	Microbial Genomics Core Lab, National Taiwan University Centers of Genomic and Precision Medicine	Shiou-Hwei Yeh et al
EPI_ISL_872592	Department of Laboratory Medicine, National Taiwan University Hospital	Microbial Genomics Core Lab, National Taiwan University Centers of Genomic and Precision Medicine	Shiou-Hwei Yeh et al
EPI_ISL_872594	Department of Laboratory Medicine, National Taiwan University Hospital	Microbial Genomics Core Lab, National Taiwan University Centers of Genomic and Precision Medicine	Shiou-Hwei Yeh et al
EPI_ISL_872597	Department of Laboratory Medicine, National Taiwan University Hospital	Microbial Genomics Core Lab, National Taiwan University Centers of Genomic and Precision Medicine	Shiou-Hwei Yeh et al
EPI_ISL_872598	Department of Laboratory Medicine, National Taiwan University Hospital	Microbial Genomics Core Lab, National Taiwan University Centers of Genomic and Precision Medicine	Shiou-Hwei Yeh et al
EPI_ISL_872599	Department of Laboratory Medicine, National Taiwan University Hospital	Microbial Genomics Core Lab, National Taiwan University Centers of Genomic and Precision Medicine	Shiou-Hwei Yeh et al
EPI_ISL_1667469	Department of Laboratory Medicine, National Taiwan University Hospital	Microbial Genomics Core Lab, National Taiwan University Centers of Genomic and Precision Medicine	Shiou-Hwei Yeh et al
EPI_ISL_1667471	Department of Laboratory Medicine, National Taiwan University Hospital	Microbial Genomics Core Lab, National Taiwan University Centers of Genomic and Precision Medicine	Shiou-Hwei Yeh et al
EPI_ISL_1667472	Department of Laboratory Medicine, National Taiwan University Hospital	Microbial Genomics Core Lab, National Taiwan University Centers of Genomic and Precision Medicine	Shiou-Hwei Yeh et al
EPI_ISL_1667474	Department of Laboratory Medicine, National Taiwan University Hospital	Microbial Genomics Core Lab, National Taiwan University Centers of Genomic and Precision Medicine	Shiou-Hwei Yeh et al
EPI_ISL_1667475	Department of Laboratory Medicine, National Taiwan University Hospital	Microbial Genomics Core Lab, National Taiwan University Centers of Genomic and Precision Medicine	Shiou-Hwei Yeh et al
EPI_ISL_796018	Hebei Provincial Center for Disease Control and Prevention, Shijiazhuang, Hebei Province; National Institute for Viral Disease Control and Prevention, China CDC	Hebei Provincial Center for Disease Control and Prevention, Shijiazhuang, Hebei Province; National Institute for Viral Disease Control and Prevention, China CDC	Shunxiang Qi et al
EPI_ISL_796022	Hebei Provincial Center for Disease Control and Prevention, Shijiazhuang, Hebei Province; National Institute for Viral Disease Control and Prevention, China CDC	Hebei Provincial Center for Disease Control and Prevention, Shijiazhuang, Hebei Province; National Institute for Viral Disease Control and Prevention, China CDC	Shunxiang Qi et al
EPI_ISL_796023	Hebei Provincial Center for Disease Control and Prevention, Shijiazhuang, Hebei Province; National Institute for Viral Disease Control and Prevention, China CDC	Hebei Provincial Center for Disease Control and Prevention, Shijiazhuang, Hebei Province; National Institute for Viral Disease Control and Prevention, China CDC	Shunxiang Qi et al

EPI_ISL_1516870	Botswana Harvard HIV Reference Laboratory	Botswana Harvard HIV Reference Laboratory	Sikhulile Dorcas Maruapula et al
EPI_ISL_1516871	Botswana Harvard HIV Reference Laboratory	Botswana Harvard HIV Reference Laboratory	Sikhulile Dorcas Maruapula et al
EPI_ISL_1516874	Botswana Harvard HIV Reference Laboratory	Botswana Harvard HIV Reference Laboratory	Sikhulile Dorcas Maruapula et al
EPI_ISL_1516877	Botswana Harvard HIV Reference Laboratory	Botswana Harvard HIV Reference Laboratory	Sikhulile Dorcas Maruapula et al
EPI_ISL_949068	Botswana Harvard HIV Reference Laboratory	Botswana Harvard HIV Reference Laboratory	Sikhulile Moyo et al
EPI_ISL_965264	Botswana Harvard HIV Reference Laboratory	Botswana Harvard HIV Reference Laboratory	Sikhulile Moyo et al
EPI_ISL_968234	Botswana Harvard HIV Reference Laboratory	Botswana Harvard HIV Reference Laboratory	Sikhulile Moyo et al
EPI_ISL_969034	Botswana Harvard HIV Reference Laboratory	Botswana Harvard HIV Reference Laboratory	Sikhulile Moyo et al
EPI_ISL_1307686	Botswana Harvard HIV Reference Laboratory	Botswana Harvard HIV Reference Laboratory	Sikhulile Moyo et al
EPI_ISL_1516834	Botswana Harvard HIV Reference Laboratory	Botswana Harvard HIV Reference Laboratory	Sikhulile Wonderful T. Choga et al
EPI_ISL_1516835	Botswana Harvard HIV Reference Laboratory	Botswana Harvard HIV Reference Laboratory	Sikhulile Wonderful T. Choga et al
EPI_ISL_1516836	Botswana Harvard HIV Reference Laboratory	Botswana Harvard HIV Reference Laboratory	Sikhulile Wonderful T. Choga et al
EPI_ISL_1516837	Botswana Harvard HIV Reference Laboratory	Botswana Harvard HIV Reference Laboratory	Sikhulile Wonderful T. Choga et al
EPI_ISL_1516838	Botswana Harvard HIV Reference Laboratory	Botswana Harvard HIV Reference Laboratory	Sikhulile Wonderful T. Choga et al
EPI_ISL_1516853	Botswana Harvard HIV Reference Laboratory	Botswana Harvard HIV Reference Laboratory	Sikhulile Wonderful T. Choga et al
EPI_ISL_1516829	Botswana Harvard HIV Reference Laboratory	Botswana Harvard HIV Reference Laboratory	Sikhulile Wonderful T. Choga et al
EPI_ISL_1629722	SOMER	Universidad Nacional de Colombia - Laboratorio Genómico One Health	Simón Villegas Velásquez et al
EPI_ISL_610210	Department of Health Technology and Informatics, The Hong Kong Polytechnic University	Department of Health Technology and Informatics, The Hong Kong Polytechnic University	Siu et al
EPI_ISL_610216	Department of Health Technology and Informatics, The Hong Kong Polytechnic University	Department of Health Technology and Informatics, The Hong Kong Polytechnic University	Siu et al
EPI_ISL_610225	Department of Health Technology and Informatics, The Hong Kong Polytechnic University	Department of Health Technology and Informatics, The Hong Kong Polytechnic University	Siu et al
EPI_ISL_452334	Laboratory of Infectious Diseases Center of Beijing Ditan Hospital	Laboratory of Infectious Diseases Center of Beijing Ditan Hospital	Siyuan Yang et al
EPI_ISL_452336	Laboratory of Infectious Diseases Center of Beijing Ditan Hospital	Laboratory of Infectious Diseases Center of Beijing Ditan Hospital	Siyuan Yang et al
EPI_ISL_456598	National Health Laboratory, Timor-Leste	Microbiological Diagnostic Unit Public Health Laboratory, The Peter Doherty Institute for Infection and Immunity	Soares da Silva et al
EPI_ISL_456601	National Health Laboratory, Timor-Leste	Microbiological Diagnostic Unit Public Health Laboratory, The Peter Doherty Institute for Infection and Immunity	Soares da Silva et al
EPI_ISL_456602	National Health Laboratory, Timor-Leste	Microbiological Diagnostic Unit Public Health Laboratory, The Peter Doherty Institute for Infection and Immunity	Soares da Silva et al
EPI_ISL_456604	National Health Laboratory, Timor-Leste	Microbiological Diagnostic Unit Public Health Laboratory, The Peter Doherty Institute for Infection and Immunity	Soares da Silva et al
EPI_ISL_456605	National Health Laboratory, Timor-Leste	Microbiological Diagnostic Unit Public Health Laboratory, The Peter Doherty Institute for Infection and Immunity	Soares da Silva et al

EPI_ISL_456610	National Health Laboratory, Timor-Leste	Microbiological Diagnostic Unit Public Health Laboratory, The Peter Doherty Institute for Infection and Immunity	Soares da Silva et al
EPI_ISL_456599	National Health Laboratory, Timor-Leste	Microbiological Diagnostic Unit Public Health Laboratory, The Peter Doherty Institute for Infection and Immunity	Soares da Silva et al
EPI_ISL_456612	National Health Laboratory, Timor-Leste	Microbiological Diagnostic Unit Public Health Laboratory, The Peter Doherty Institute for Infection and Immunity	Soares da Silva et al
EPI_ISL_456600	National Health Laboratory, Timor-Leste	Microbiological Diagnostic Unit Public Health Laboratory, The Peter Doherty Institute for Infection and Immunity	Soares da Silva et al
EPI_ISL_918368	Virology Unit, Institut Pasteur du Cambodge	Virology Unit, Institut Pasteur du Cambodge	Sokhoun Yann et al
EPI_ISL_918366	Virology Unit, Institut Pasteur du Cambodge	Virology Unit, Institut Pasteur du Cambodge	Sokhoun Yann et al
EPI_ISL_918359	Virology Unit, Institut Pasteur du Cambodge	Virology Unit, Institut Pasteur du Cambodge	Sokhoun Yann et al
EPI_ISL_918361	Virology Unit, Institut Pasteur du Cambodge	Virology Unit, Institut Pasteur du Cambodge	Sokhoun Yann et al
EPI_ISL_1532806	Virology Unit, Institut Pasteur du Cambodge	Virology Unit, Institut Pasteur du Cambodge	Sokhoun Yann et al
EPI_ISL_1532811	Virology Unit, Institut Pasteur du Cambodge	Virology Unit, Institut Pasteur du Cambodge	Sokhoun Yann et al
EPI_ISL_1534542	Virology Unit, Institut Pasteur du Cambodge	Virology Unit, Institut Pasteur du Cambodge	Sokhoun Yann et al
EPI_ISL_1534540	Virology Unit, Institut Pasteur du Cambodge	Virology Unit, Institut Pasteur du Cambodge	Sokhoun Yann et al
EPI_ISL_1534536	Virology Unit, Institut Pasteur du Cambodge	Virology Unit, Institut Pasteur du Cambodge	Sokhoun Yann et al
EPI_ISL_1534543	Virology Unit, Institut Pasteur du Cambodge	Virology Unit, Institut Pasteur du Cambodge	Sokhoun Yann et al
EPI_ISL_1532803	Cambodian National Public Health Laboratory, National Institute of Public Health	Virology Unit, Institut Pasteur du Cambodge	Sokhoun Yann et al
EPI_ISL_1096139	Cambodian National Public Health Laboratory, National Institute of Public Health	Virology Unit, Institut Pasteur du Cambodge	Sokhoun Yann et al
EPI_ISL_1096140	Cambodian National Public Health Laboratory, National Institute of Public Health	Virology Unit, Institut Pasteur du Cambodge	Sokhoun Yann et al
EPI_ISL_576143	Public Health Virology Laboratory, Forensic and Scientific Services (PHV-FSS)	Public Health Virology Laboratory, Forensic and Scientific Services (PHV-FSS)	Son Nguyen et al
EPI_ISL_693257	Public Health Virology Laboratory, Forensic and Scientific Services (PHV-FSS)	Public Health Virology Laboratory, Forensic and Scientific Services (PHV-FSS)	Son Nguyen et al
EPI_ISL_693278	Public Health Virology Laboratory, Forensic and Scientific Services (PHV-FSS)	Public Health Virology Laboratory, Forensic and Scientific Services (PHV-FSS)	Son Nguyen et al
EPI_ISL_849680	Public Health Virology Laboratory, Forensic and Scientific Services (PHV-FSS)	Public Health Virology Laboratory, Forensic and Scientific Services (PHV-FSS)	Son Nguyen et al
EPI_ISL_849730	Public Health Virology Laboratory, Forensic and Scientific Services (PHV-FSS)	Public Health Virology Laboratory, Forensic and Scientific Services (PHV-FSS)	Son Nguyen et al
EPI_ISL_849733	Public Health Virology Laboratory, Forensic and Scientific Services (PHV-FSS)	Public Health Virology Laboratory, Forensic and Scientific Services (PHV-FSS)	Son Nguyen et al
EPI_ISL_1300529	Public Health Virology-Forensic and Scientific Services (PHV-FSS)	Public Health Virology-Forensic and Scientific Services (PHV-FSS)	Son Nguyen et al
EPI_ISL_906056	Tilia Laboratories s.r.o.	Tilia Laboratories s.r.o.	Sona Pekova et al

EPI_ISL_906063	Tilia Laboratories s.r.o.	Tilia Laboratories s.r.o.	Sona Pekova et al
EPI_ISL_906064	Tilia Laboratories s.r.o.	Tilia Laboratories s.r.o.	Sona Pekova et al
EPI_ISL_906054	Tilia Laboratories s.r.o.	Tilia Laboratories s.r.o.	Sona Pekova et al
EPI_ISL_906057	Tilia Laboratories s.r.o.	Tilia Laboratories s.r.o.	Sona Pekova et al
EPI_ISL_906062	Tilia Laboratories s.r.o.	Tilia Laboratories s.r.o.	Sona Pekova et al
EPI_ISL_906053	Tilia Laboratories s.r.o.	Tilia Laboratories s.r.o.	Sona Pekova et al
EPI_ISL_906055	Tilia Laboratories s.r.o.	Tilia Laboratories s.r.o.	Sona Pekova MD et al
EPI_ISL_728237	LNR National Reference Laboratory, Mohammed VI University of Health Sciences	Medical Biotechnology Laboratory, Rabat Medical and Pharmacy School, Mohammed The Vth University in Rabat	Souad KARTTI et al
EPI_ISL_1116467	Laboratoire de Microbiologie- CHU Habib Bourguiba – Sfax	Laboratoire des Procédés de Criblage Moléculaire et Cellulaire-Centre de Biotechnologie de Sfax	Souissi et al
EPI_ISL_1118884	Laboratoire de Microbiologie- CHU Habib Bourguiba – Sfax	Laboratoire des Procédés de Criblage Moléculaire et Cellulaire-Centre de Biotechnologie de Sfax	Souissi et al
EPI_ISL_1116464	Laboratoire de Microbiologie- CHU Habib Bourguiba – Sfax	Laboratoire des Procédés de Criblage Moléculaire et Cellulaire-Centre de Biotechnologie de Sfax	Souissi et al
EPI_ISL_1116469	Laboratoire de Microbiologie- CHU Habib Bourguiba – Sfax	Laboratoire des Procédés de Criblage Moléculaire et Cellulaire-Centre de Biotechnologie de Sfax	Souissi et al
EPI_ISL_1118923	Laboratoire de Microbiologie- CHU Habib Bourguiba – Sfax	Laboratoire des Procédés de Criblage Moléculaire et Cellulaire-Centre de Biotechnologie de Sfax	Souissi et al
EPI_ISL_1116468	Laboratoire de Microbiologie- CHU Habib Bourguiba – Sfax	Laboratoire des Procédés de Criblage Moléculaire et Cellulaire-Centre de Biotechnologie de Sfax	Souissi et al
EPI_ISL_710575	Hôpital Fattouma-Bourguiba de Monastir	Laboratoire des Procédés de Criblage Moléculaire et Cellulaire-Centre de Biotechnologie de Sfax	Souissi et al
EPI_ISL_710537	Hôpital Fattouma-Bourguiba de Monastir	Laboratoire des Procédés de Criblage Moléculaire et Cellulaire-Centre de Biotechnologie de Sfax	Souissi et al
EPI_ISL_711057	Hôpital Fattouma-Bourguiba de Monastir	Laboratoire des Procédés de Criblage Moléculaire et Cellulaire-Centre de Biotechnologie de Sfax	Souissi et al
EPI_ISL_712069	Laboratoire de Microbiologie- CHU Habib Bourguiba – Sfax adresse	Laboratoire des Procédés de Criblage Moléculaire et Cellulaire-Centre de Biotechnologie de Sfax	Souissi et al
EPI_ISL_712065	Laboratoire de Microbiologie- CHU Habib Bourguiba – Sfax adresse	Laboratoire des Procédés de Criblage Moléculaire et Cellulaire-Centre de Biotechnologie de Sfax	Souissi et al
EPI_ISL_712066	Laboratoire de Microbiologie- CHU Habib Bourguiba – Sfax adresse	Laboratoire des Procédés de Criblage Moléculaire et Cellulaire-Centre de Biotechnologie de Sfax	Souissi et al
EPI_ISL_1137609	Département de Maladies Infectieuses, CHU Farhat Hached Sousse, Tunisie	Laboratoire des Procédés de Criblage Moléculaire et Cellulaire-Centre de Biotechnologie de Sfax	Souissi et al
EPI_ISL_1167128	Iressef Genomics lab	L'institut de Recherche en Santé, de Surveillance Épidémiologique et de Formation (IRESSEF)	Souleymane MBOUP et al
EPI_ISL_1633465	Institute for Health Research, Epidemiological Surveillance and Training (IRESSEF)	Abbott	Souleymane Mboup et al
EPI_ISL_1633466	Institute for Health Research, Epidemiological Surveillance and Training (IRESSEF)	Abbott	Souleymane Mboup et al

EPI_ISL_1633467	Institute for Health Research, Epidemiological Surveillance and Training (IRESSEF)	Abbott	Souleymane Mboup et al
EPI_ISL_1630272	IRESSEF	Abbott	Souleymane Mboup et al
EPI_ISL_1630258	IRESSEF	Abbott	Souleymane Mboup et al
EPI_ISL_1167181	Iressef Genomics lab	L'institut de Recherche en Santé, de Surveillance Épidémiologique et de Formation (IRESSEF)	Souleymane MBOUP et al
EPI_ISL_1167131	Iressef Genomics lab	L'institut de Recherche en Santé, de Surveillance Épidémiologique et de Formation (IRESSEF)	Souleymane MBOUP et al
EPI_ISL_1167125	Iressef Genomics lab	L'institut de Recherche en Santé, de Surveillance Épidémiologique et de Formation (IRESSEF)	Souleymane MBOUP et al
EPI_ISL_1167162	Iressef Genomics lab	L'institut de Recherche en Santé, de Surveillance Épidémiologique et de Formation (IRESSEF)	Souleymane MBOUP et al
EPI_ISL_1167163	Iressef Genomics lab	L'institut de Recherche en Santé, de Surveillance Épidémiologique et de Formation (IRESSEF)	Souleymane MBOUP et al
EPI_ISL_1167134	Iressef Genomics lab	L'institut de Recherche en Santé, de Surveillance Épidémiologique et de Formation (IRESSEF)	Souleymane MBOUP et al
EPI_ISL_1167127	Iressef Genomics lab	L'institut de Recherche en Santé, de Surveillance Épidémiologique et de Formation (IRESSEF)	Souleymane MBOUP et al
EPI_ISL_1167171	Iressef Genomics lab	L'institut de Recherche en Santé, de Surveillance Épidémiologique et de Formation (IRESSEF)	Souleymane MBOUP et al
EPI_ISL_1167126	Iressef Genomics lab	L'institut de Recherche en Santé, de Surveillance Épidémiologique et de Formation (IRESSEF)	Souleymane MBOUP et al
EPI_ISL_1630257	IRESSEF	Abbott	Souleymane Mboup et al
EPI_ISL_1630267	IRESSEF	Abbott	Souleymane Mboup et al
EPI_ISL_1630268	IRESSEF	Abbott	Souleymane Mboup et al
EPI_ISL_1261763	Iressef Genomics lab	IRESSEF	Souleymane MBOUP et al
EPI_ISL_1630264	IRESSEF	Abbott	Souleymane Mboup et al
EPI_ISL_636838	Laboratório de Imunofarmacologia - Instituto Oswaldo Cruz	Laboratório de Imunofarmacologia - Instituto Oswaldo Cruz	Souza et al
EPI_ISL_1020200	RS Trimitra, Bogor, West java	Biosafety Level-3 Laboratory, Indonesian Institute of Sciences (LIPI)	Sri Swasthikawati et al
EPI_ISL_413019	Department of Internal Medicine, Triemli Hospital	Institute of Medical Virology, University of Zurich	Stefan Schmutz et al
EPI_ISL_413021	Klinik Hirslanden Zurich	Institute of Medical Virology, University of Zurich	Stefan Schmutz et al
EPI_ISL_413023	Division of Infectious Diseases, University Hospital Zurich	Institute of Medical Virology, University of Zurich	Stefan Schmutz et al
EPI_ISL_1068315	Central Public Health Laboratory - LACEN -Bahia, Salvador, Brazil	Central Public Health Laboratory - LACEN -Bahia, Salvador, Brazil	Stephane Tosta et al
EPI_ISL_1068316	Central Public Health Laboratory - LACEN -Bahia, Salvador, Brazil	Central Public Health Laboratory - LACEN -Bahia, Salvador, Brazil	Stephane Tosta et al
EPI_ISL_1068317	Central Public Health Laboratory - LACEN -Bahia, Salvador, Brazil	Central Public Health Laboratory - LACEN -Bahia, Salvador, Brazil	Stephane Tosta et al
EPI_ISL_1068319	Central Public Health Laboratory - LACEN -Bahia, Salvador, Brazil	Central Public Health Laboratory - LACEN -Bahia, Salvador, Brazil	Stephane Tosta et al
EPI_ISL_1068320	Central Public Health Laboratory - LACEN -Bahia, Salvador, Brazil	Central Public Health Laboratory - LACEN -Bahia, Salvador, Brazil	Stephane Tosta et al

EPI_ISL_1068323	Central Public Health Laboratory - LACEN -Bahia, Salvador, Brazil	Central Public Health Laboratory - LACEN -Bahia, Salvador, Brazil	Stephane Tosta et al
EPI_ISL_978495	Central Public Health Laboratory - LACEN -Bahia, Salvador, Brazil	Central Public Health Laboratory - LACEN -Bahia, Salvador, Brazil	Stephane Tosta et al
EPI_ISL_1068385	Central Public Health Laboratory - LACEN -Bahia, Salvador, Brazil	Central Public Health Laboratory - LACEN -Bahia, Salvador, Brazil	Stephane Tosta et al
EPI_ISL_1068387	Central Public Health Laboratory - LACEN -Bahia, Salvador, Brazil	Central Public Health Laboratory - LACEN -Bahia, Salvador, Brazil	Stephane Tosta et al
EPI_ISL_1068388	Central Public Health Laboratory - LACEN -Bahia, Salvador, Brazil	Central Public Health Laboratory - LACEN -Bahia, Salvador, Brazil	Stephane Tosta et al
EPI_ISL_1068389	Central Public Health Laboratory - LACEN -Bahia, Salvador, Brazil	Central Public Health Laboratory - LACEN -Bahia, Salvador, Brazil	Stephane Tosta et al
EPI_ISL_1068390	Central Public Health Laboratory - LACEN -Bahia, Salvador, Brazil	Central Public Health Laboratory - LACEN -Bahia, Salvador, Brazil	Stephane Tosta et al
EPI_ISL_978496	Central Public Health Laboratory - LACEN -Bahia, Salvador, Brazil	Central Public Health Laboratory - LACEN -Bahia, Salvador, Brazil	Stephane Tosta et al
EPI_ISL_1068393	Central Public Health Laboratory - LACEN -Bahia, Salvador, Brazil	Central Public Health Laboratory - LACEN -Bahia, Salvador, Brazil	Stephane Tosta et al
EPI_ISL_1068394	Central Public Health Laboratory - LACEN -Bahia, Salvador, Brazil	Central Public Health Laboratory - LACEN -Bahia, Salvador, Brazil	Stephane Tosta et al
EPI_ISL_978497	Central Public Health Laboratory - LACEN -Bahia, Salvador, Brazil	Central Public Health Laboratory - LACEN -Bahia, Salvador, Brazil	Stephane Tosta et al
EPI_ISL_978498	Central Public Health Laboratory - LACEN -Bahia, Salvador, Brazil	Central Public Health Laboratory - LACEN -Bahia, Salvador, Brazil	Stephane Tosta et al
EPI_ISL_978499	Central Public Health Laboratory - LACEN -Bahia, Salvador, Brazil	Central Public Health Laboratory - LACEN -Bahia, Salvador, Brazil	Stephane Tosta et al
EPI_ISL_978500	Central Public Health Laboratory - LACEN -Bahia, Salvador, Brazil	Central Public Health Laboratory - LACEN -Bahia, Salvador, Brazil	Stephane Tosta et al
EPI_ISL_978502	Central Public Health Laboratory - LACEN -Bahia, Salvador, Brazil	Central Public Health Laboratory - LACEN -Bahia, Salvador, Brazil	Stephane Tosta et al
EPI_ISL_978503	Central Public Health Laboratory - LACEN -Bahia, Salvador, Brazil	Central Public Health Laboratory - LACEN -Bahia, Salvador, Brazil	Stephane Tosta et al
EPI_ISL_978504	Central Public Health Laboratory - LACEN -Bahia, Salvador, Brazil	Central Public Health Laboratory - LACEN -Bahia, Salvador, Brazil	Stephane Tosta et al
EPI_ISL_978505	Central Public Health Laboratory - LACEN -Bahia, Salvador, Brazil	Central Public Health Laboratory - LACEN -Bahia, Salvador, Brazil	Stephane Tosta et al
EPI_ISL_978506	Central Public Health Laboratory - LACEN -Bahia, Salvador, Brazil	Central Public Health Laboratory - LACEN -Bahia, Salvador, Brazil	Stephane Tosta et al
EPI_ISL_978507	Central Public Health Laboratory - LACEN -Bahia, Salvador, Brazil	Central Public Health Laboratory - LACEN -Bahia, Salvador, Brazil	Stephane Tosta et al
EPI_ISL_978508	Central Public Health Laboratory - LACEN -Bahia, Salvador, Brazil	Central Public Health Laboratory - LACEN -Bahia, Salvador, Brazil	Stephane Tosta et al
EPI_ISL_978510	Central Public Health Laboratory - LACEN -Bahia, Salvador, Brazil	Central Public Health Laboratory - LACEN -Bahia, Salvador, Brazil	Stephane Tosta et al
EPI_ISL_978512	Central Public Health Laboratory - LACEN -Bahia, Salvador, Brazil	Central Public Health Laboratory - LACEN -Bahia, Salvador, Brazil	Stephane Tosta et al
EPI_ISL_978513	Central Public Health Laboratory - LACEN -Bahia, Salvador, Brazil	Central Public Health Laboratory - LACEN -Bahia, Salvador, Brazil	Stephane Tosta et al
EPI_ISL_978514	Central Public Health Laboratory - LACEN -Bahia, Salvador, Brazil	Central Public Health Laboratory - LACEN -Bahia, Salvador, Brazil	Stephane Tosta et al
EPI_ISL_978515	Central Public Health Laboratory - LACEN -Bahia, Salvador, Brazil	Central Public Health Laboratory - LACEN -Bahia, Salvador, Brazil	Stephane Tosta et al
EPI_ISL_978516	Central Public Health Laboratory - LACEN -Bahia, Salvador, Brazil	Central Public Health Laboratory - LACEN -Bahia, Salvador, Brazil	Stephane Tosta et al
EPI_ISL_978517	Central Public Health Laboratory - LACEN -Bahia, Salvador, Brazil	Central Public Health Laboratory - LACEN -Bahia, Salvador, Brazil	Stephane Tosta et al
EPI_ISL_978518	Central Public Health Laboratory - LACEN -Bahia, Salvador, Brazil	Central Public Health Laboratory - LACEN -Bahia, Salvador, Brazil	Stephane Tosta et al
EPI_ISL_978520	Central Public Health Laboratory - LACEN -Bahia, Salvador, Brazil	Central Public Health Laboratory - LACEN -Bahia, Salvador, Brazil	Stephane Tosta et al

EPI_ISL_978522	Central Public Health Laboratory - LACEN -Bahia, Salvador, Brazil	Central Public Health Laboratory - LACEN -Bahia, Salvador, Brazil	Stephane Tosta et al
EPI_ISL_978525	Central Public Health Laboratory - LACEN -Bahia, Salvador, Brazil	Central Public Health Laboratory - LACEN -Bahia, Salvador, Brazil	Stephane Tosta et al
EPI_ISL_978528	Central Public Health Laboratory - LACEN -Bahia, Salvador, Brazil	Central Public Health Laboratory - LACEN -Bahia, Salvador, Brazil	Stephane Tosta et al
EPI_ISL_978529	Central Public Health Laboratory - LACEN -Bahia, Salvador, Brazil	Central Public Health Laboratory - LACEN -Bahia, Salvador, Brazil	Stephane Tosta et al
EPI_ISL_978530	Central Public Health Laboratory - LACEN -Bahia, Salvador, Brazil	Central Public Health Laboratory - LACEN -Bahia, Salvador, Brazil	Stephane Tosta et al
EPI_ISL_978531	Central Public Health Laboratory - LACEN -Bahia, Salvador, Brazil	Central Public Health Laboratory - LACEN -Bahia, Salvador, Brazil	Stephane Tosta et al
EPI_ISL_978532	Central Public Health Laboratory - LACEN -Bahia, Salvador, Brazil	Central Public Health Laboratory - LACEN -Bahia, Salvador, Brazil	Stephane Tosta et al
EPI_ISL_1067737	Central Public Health Laboratory - LACEN -Bahia, Salvador, Brazil	Central Public Health Laboratory - LACEN -Bahia, Salvador, Brazil	Stephane Tosta et al
EPI_ISL_1067730	Central Public Health Laboratory - LACEN -Bahia, Salvador, Brazil	Central Public Health Laboratory - LACEN -Bahia, Salvador, Brazil	Stephane Tosta et al
EPI_ISL_1067738	Central Public Health Laboratory - LACEN -Bahia, Salvador, Brazil	Central Public Health Laboratory - LACEN -Bahia, Salvador, Brazil	Stephane Tosta et al
EPI_ISL_1067731	Central Public Health Laboratory - LACEN -Bahia, Salvador, Brazil	Central Public Health Laboratory - LACEN -Bahia, Salvador, Brazil	Stephane Tosta et al
EPI_ISL_1067733	Central Public Health Laboratory - LACEN -Bahia, Salvador, Brazil	Central Public Health Laboratory - LACEN -Bahia, Salvador, Brazil	Stephane Tosta et al
EPI_ISL_1067729	Central Public Health Laboratory - LACEN -Bahia, Salvador, Brazil	Central Public Health Laboratory - LACEN -Bahia, Salvador, Brazil	Stephane Tosta et al
EPI_ISL_1067734	Central Public Health Laboratory - LACEN -Bahia, Salvador, Brazil	Central Public Health Laboratory - LACEN -Bahia, Salvador, Brazil	Stephane Tosta et al
EPI_ISL_1067735	Central Public Health Laboratory - LACEN -Bahia, Salvador, Brazil	Central Public Health Laboratory - LACEN -Bahia, Salvador, Brazil	Stephane Tosta et al
EPI_ISL_1067736	Center for Biotechnology and Cell Therapy, São Rafael Hospital, Salvador, Brazil	Central Public Health Laboratory - LACEN -Bahia, Salvador, Brazil	Stephane Tosta et al
EPI_ISL_1067728	Center for Biotechnology and Cell Therapy, São Rafael Hospital, Salvador, Brazil	Central Public Health Laboratory - LACEN -Bahia, Salvador, Brazil	Stephane Tosta et al
EPI_ISL_1067732	Center for Biotechnology and Cell Therapy, São Rafael Hospital, Salvador, Brazil	Central Public Health Laboratory - LACEN -Bahia, Salvador, Brazil	Stephane Tosta et al
EPI_ISL_1068363	Central Public Health Laboratory - LACEN -Bahia, Salvador, Brazil	Central Public Health Laboratory - LACEN -Bahia, Salvador, Brazil	Stephane Tosta et al
EPI_ISL_1068364	Central Public Health Laboratory - LACEN -Bahia, Salvador, Brazil	Central Public Health Laboratory - LACEN -Bahia, Salvador, Brazil	Stephane Tosta et al
EPI_ISL_1068366	Central Public Health Laboratory - LACEN -Bahia, Salvador, Brazil	Central Public Health Laboratory - LACEN -Bahia, Salvador, Brazil	Stephane Tosta et al
EPI_ISL_1068367	Central Public Health Laboratory - LACEN -Bahia, Salvador, Brazil	Central Public Health Laboratory - LACEN -Bahia, Salvador, Brazil	Stephane Tosta et al
EPI_ISL_1068368	Central Public Health Laboratory - LACEN -Bahia, Salvador, Brazil	Central Public Health Laboratory - LACEN -Bahia, Salvador, Brazil	Stephane Tosta et al
EPI_ISL_1068369	Central Public Health Laboratory - LACEN -Bahia, Salvador, Brazil	Central Public Health Laboratory - LACEN -Bahia, Salvador, Brazil	Stephane Tosta et al
EPI_ISL_1068370	Central Public Health Laboratory - LACEN -Bahia, Salvador, Brazil	Central Public Health Laboratory - LACEN -Bahia, Salvador, Brazil	Stephane Tosta et al
EPI_ISL_1068371	Central Public Health Laboratory - LACEN -Bahia, Salvador, Brazil	Central Public Health Laboratory - LACEN -Bahia, Salvador, Brazil	Stephane Tosta et al
EPI_ISL_1068372	Central Public Health Laboratory - LACEN -Bahia, Salvador, Brazil	Central Public Health Laboratory - LACEN -Bahia, Salvador, Brazil	Stephane Tosta et al
EPI_ISL_1068373	Central Public Health Laboratory - LACEN -Bahia, Salvador, Brazil	Central Public Health Laboratory - LACEN -Bahia, Salvador, Brazil	Stephane Tosta et al
EPI_ISL_1068374	Central Public Health Laboratory - LACEN -Bahia, Salvador, Brazil	Central Public Health Laboratory - LACEN -Bahia, Salvador, Brazil	Stephane Tosta et al

EPI_ISL_1068375	Central Public Health Laboratory - LACEN -Bahia, Salvador, Brazil	Central Public Health Laboratory - LACEN -Bahia, Salvador, Brazil	Stephane Tosta et al
EPI_ISL_1068376	Central Public Health Laboratory - LACEN -Bahia, Salvador, Brazil	Central Public Health Laboratory - LACEN -Bahia, Salvador, Brazil	Stephane Tosta et al
EPI_ISL_1068377	Central Public Health Laboratory - LACEN -Bahia, Salvador, Brazil	Central Public Health Laboratory - LACEN -Bahia, Salvador, Brazil	Stephane Tosta et al
EPI_ISL_1068378	Central Public Health Laboratory - LACEN -Bahia, Salvador, Brazil	Central Public Health Laboratory - LACEN -Bahia, Salvador, Brazil	Stephane Tosta et al
EPI_ISL_1068379	Central Public Health Laboratory - LACEN -Bahia, Salvador, Brazil	Central Public Health Laboratory - LACEN -Bahia, Salvador, Brazil	Stephane Tosta et al
EPI_ISL_1068380	Central Public Health Laboratory - LACEN -Bahia, Salvador, Brazil	Central Public Health Laboratory - LACEN -Bahia, Salvador, Brazil	Stephane Tosta et al
EPI_ISL_1068382	Central Public Health Laboratory - LACEN -Bahia, Salvador, Brazil	Central Public Health Laboratory - LACEN -Bahia, Salvador, Brazil	Stephane Tosta et al
EPI_ISL_1583642	Central Public Health Laboratory - LACEN -Bahia, Salvador, Brazil	Central Public Health Laboratory - LACEN -Bahia, Salvador, Brazil	Stephane Tosta et al
EPI_ISL_1583643	Central Public Health Laboratory - LACEN -Bahia, Salvador, Brazil	Central Public Health Laboratory - LACEN -Bahia, Salvador, Brazil	Stephane Tosta et al
EPI_ISL_1583644	Central Public Health Laboratory - LACEN -Bahia, Salvador, Brazil	Central Public Health Laboratory - LACEN -Bahia, Salvador, Brazil	Stephane Tosta et al
EPI_ISL_1583645	Central Public Health Laboratory - LACEN -Bahia, Salvador, Brazil	Central Public Health Laboratory - LACEN -Bahia, Salvador, Brazil	Stephane Tosta et al
EPI_ISL_1583646	Central Public Health Laboratory - LACEN -Bahia, Salvador, Brazil	Central Public Health Laboratory - LACEN -Bahia, Salvador, Brazil	Stephane Tosta et al
EPI_ISL_1583647	Central Public Health Laboratory - LACEN -Bahia, Salvador, Brazil	Central Public Health Laboratory - LACEN -Bahia, Salvador, Brazil	Stephane Tosta et al
EPI_ISL_1583648	Central Public Health Laboratory - LACEN -Bahia, Salvador, Brazil	Central Public Health Laboratory - LACEN -Bahia, Salvador, Brazil	Stephane Tosta et al
EPI_ISL_1583649	Central Public Health Laboratory - LACEN -Bahia, Salvador, Brazil	Central Public Health Laboratory - LACEN -Bahia, Salvador, Brazil	Stephane Tosta et al
EPI_ISL_1583651	Central Public Health Laboratory - LACEN -Bahia, Salvador, Brazil	Central Public Health Laboratory - LACEN -Bahia, Salvador, Brazil	Stephane Tosta et al
EPI_ISL_1583652	Central Public Health Laboratory - LACEN -Bahia, Salvador, Brazil	Central Public Health Laboratory - LACEN -Bahia, Salvador, Brazil	Stephane Tosta et al
EPI_ISL_1583654	Central Public Health Laboratory - LACEN -Bahia, Salvador, Brazil	Central Public Health Laboratory - LACEN -Bahia, Salvador, Brazil	Stephane Tosta et al
EPI_ISL_1583656	Central Public Health Laboratory - LACEN -Bahia, Salvador, Brazil	Central Public Health Laboratory - LACEN -Bahia, Salvador, Brazil	Stephane Tosta et al
EPI_ISL_1583659	Central Public Health Laboratory - LACEN -Bahia, Salvador, Brazil	Central Public Health Laboratory - LACEN -Bahia, Salvador, Brazil	Stephane Tosta et al
EPI_ISL_1583660	Central Public Health Laboratory - LACEN -Bahia, Salvador, Brazil	Central Public Health Laboratory - LACEN -Bahia, Salvador, Brazil	Stephane Tosta et al
EPI_ISL_1583661	Central Public Health Laboratory - LACEN -Bahia, Salvador, Brazil	Central Public Health Laboratory - LACEN -Bahia, Salvador, Brazil	Stephane Tosta et al
EPI_ISL_1583663	Central Public Health Laboratory - LACEN -Bahia, Salvador, Brazil	Central Public Health Laboratory - LACEN -Bahia, Salvador, Brazil	Stephane Tosta et al
EPI_ISL_1583664	Central Public Health Laboratory - LACEN -Bahia, Salvador, Brazil	Central Public Health Laboratory - LACEN -Bahia, Salvador, Brazil	Stephane Tosta et al
EPI_ISL_1583665	Central Public Health Laboratory - LACEN -Bahia, Salvador, Brazil	Central Public Health Laboratory - LACEN -Bahia, Salvador, Brazil	Stephane Tosta et al
EPI_ISL_1583668	Central Public Health Laboratory - LACEN -Bahia, Salvador, Brazil	Central Public Health Laboratory - LACEN -Bahia, Salvador, Brazil	Stephane Tosta et al
EPI_ISL_1583669	Central Public Health Laboratory - LACEN -Bahia, Salvador, Brazil	Central Public Health Laboratory - LACEN -Bahia, Salvador, Brazil	Stephane Tosta et al
EPI_ISL_1583670	Central Public Health Laboratory - LACEN -Bahia, Salvador, Brazil	Central Public Health Laboratory - LACEN -Bahia, Salvador, Brazil	Stephane Tosta et al
EPI_ISL_1583672	Central Public Health Laboratory - LACEN -Bahia, Salvador, Brazil	Central Public Health Laboratory - LACEN -Bahia, Salvador, Brazil	Stephane Tosta et al
EPI_ISL_1583673	Central Public Health Laboratory - LACEN -Bahia, Salvador, Brazil	Central Public Health Laboratory - LACEN -Bahia, Salvador, Brazil	Stephane Tosta et al

EPI_ISL_1583675	DNA Laboratory	Central Public Health Laboratory - LACEN -Bahia, Salvador, Brazil	Stephane Tosta et al
EPI_ISL_1583676	Central Public Health Laboratory - LACEN -Bahia, Salvador, Brazil	Central Public Health Laboratory - LACEN -Bahia, Salvador, Brazil	Stephane Tosta et al
EPI_ISL_1583679	Central Public Health Laboratory - LACEN -Bahia, Salvador, Brazil	Central Public Health Laboratory - LACEN -Bahia, Salvador, Brazil	Stephane Tosta et al
EPI_ISL_1583680	Central Public Health Laboratory - LACEN -Bahia, Salvador, Brazil	Central Public Health Laboratory - LACEN -Bahia, Salvador, Brazil	Stephane Tosta et al
EPI_ISL_1583681	Central Public Health Laboratory - LACEN -Bahia, Salvador, Brazil	Central Public Health Laboratory - LACEN -Bahia, Salvador, Brazil	Stephane Tosta et al
EPI_ISL_1583686	Central Public Health Laboratory - LACEN -Bahia, Salvador, Brazil	Central Public Health Laboratory - LACEN -Bahia, Salvador, Brazil	Stephane Tosta et al
EPI_ISL_1583689	Central Public Health Laboratory - LACEN -Bahia, Salvador, Brazil	Central Public Health Laboratory - LACEN -Bahia, Salvador, Brazil	Stephane Tosta et al
EPI_ISL_1583694	Central Public Health Laboratory - LACEN -Bahia, Salvador, Brazil	Central Public Health Laboratory - LACEN -Bahia, Salvador, Brazil	Stephane Tosta et al
EPI_ISL_1583697	Central Public Health Laboratory - LACEN -Bahia, Salvador, Brazil	Central Public Health Laboratory - LACEN -Bahia, Salvador, Brazil	Stephane Tosta et al
EPI_ISL_1583700	Central Public Health Laboratory - LACEN -Bahia, Salvador, Brazil	Central Public Health Laboratory - LACEN -Bahia, Salvador, Brazil	Stephane Tosta et al
EPI_ISL_1583703	Central Public Health Laboratory - LACEN -Bahia, Salvador, Brazil	Central Public Health Laboratory - LACEN -Bahia, Salvador, Brazil	Stephane Tosta et al
EPI_ISL_1583705	Central Public Health Laboratory - LACEN -Bahia, Salvador, Brazil	Central Public Health Laboratory - LACEN -Bahia, Salvador, Brazil	Stephane Tosta et al
EPI_ISL_1583714	Central Public Health Laboratory - LACEN -Bahia, Salvador, Brazil	Central Public Health Laboratory - LACEN -Bahia, Salvador, Brazil	Stephane Tosta et al
EPI_ISL_1583716	Central Public Health Laboratory - LACEN -Bahia, Salvador, Brazil	Central Public Health Laboratory - LACEN -Bahia, Salvador, Brazil	Stephane Tosta et al
EPI_ISL_1583725	Central Public Health Laboratory - LACEN -Bahia, Salvador, Brazil	Central Public Health Laboratory - LACEN -Bahia, Salvador, Brazil	Stephane Tosta et al
EPI_ISL_1583727	Central Public Health Laboratory - LACEN -Bahia, Salvador, Brazil	Central Public Health Laboratory - LACEN -Bahia, Salvador, Brazil	Stephane Tosta et al
EPI_ISL_1583730	Central Public Health Laboratory - LACEN -Bahia, Salvador, Brazil	Central Public Health Laboratory - LACEN -Bahia, Salvador, Brazil	Stephane Tosta et al
EPI_ISL_444008	PHE South West Regional Laboratory, National Infection Service	Wellcome Sanger Institute for the COVID-19 Genomics UK (COG-UK) consortium	Stephanie Hutchings et al
EPI_ISL_447469	Clinical Microbiology Laboratory, Sheba Medical Center	Stern Lab	Stern Lab et al
EPI_ISL_447464	Clinical Microbiology Laboratory, Sheba Medical Center	Stern Lab	Stern Lab et al
EPI_ISL_447462	Clinical Microbiology Laboratory, Sheba Medical Center	Stern Lab	Stern Lab et al
EPI_ISL_1425044	Clalit Health Services Laboratories, Israel	Stern Lab	Stern Lab et al
EPI_ISL_1278926	Clalit Health Services Laboratories, Israel	Stern Lab	Stern Lab et al
EPI_ISL_1278865	Clalit Health Services Laboratories, Israel	Stern Lab	Stern Lab et al
EPI_ISL_1424934	Clalit Health Services Laboratories, Israel	Stern Lab	Stern Lab et al
EPI_ISL_1424942	Clalit Health Services Laboratories, Israel	Stern Lab	Stern Lab et al
EPI_ISL_1424974	Clalit Health Services Laboratories, Israel	Stern Lab	Stern Lab et al
EPI_ISL_447398	Clinical Microbiology Laboratory, The Baruch Padeh Medical Center, Poriya	Stern Lab	Stern Lab et al
EPI_ISL_1278872	Clalit Health Services Laboratories, Israel	Stern Lab	Stern Lab et al
EPI_ISL_1278853	Clalit Health Services Laboratories, Israel	Stern Lab	Stern Lab et al

EPI_ISL_1278775	Clalit Health Services Laboratories, Israel	Stern Lab	Stern Lab et al
EPI_ISL_1424887	Clalit Health Services Laboratories, Israel	Stern Lab	Stern Lab et al
EPI_ISL_447304	Microbiology Division, Barzilai University Medical Center	Stern Lab	Stern Lab et al
EPI_ISL_447298	Microbiology Division, Barzilai University Medical Center	Stern Lab	Stern Lab et al
EPI_ISL_447270	Microbiology laboratory, Assuta Ashdod University-Affiliated Hospital	Stern Lab	Stern Lab et al
EPI_ISL_1222766	Central Scientific Research Department, Gomel State Medical University,	Laboratory of Genomics and Bioinformatics of the Forest Research Institute of the NAS of Belarus	Stoma et al
EPI_ISL_1169052	National Institute of Health Research and Development	National Institute of Health Research and Development	Subangkit et al
EPI_ISL_1534706	National Institute Health Research and Development	National Institute of Health Research and Development	Subangkit et al
EPI_ISL_1469264	National Institute Health Research and Development	National Institute of Health Research and Development	Subangkit et al
EPI_ISL_775599	Balai Besar Veteriner Maros	National Institute of Health Research and Development	Subangkit et al
EPI_ISL_944762	CSIR-Indian Institute of Chemical Biology, MEDICA Supercpecialty Hospital Kolkata	CSIR-Indian Institute of Chemical Biology, MEDICA Supercpecialty Hospital Kolkata	Sujay Krishna Maity et al
EPI_ISL_1265456	BTKLPP Kelas I Makassar	Eijkman Institute for Molecular Biology, Ministry of Research and Technology/National Agency for Research and Innovation; National Institute of Health Research and Development	Sukma Oktavianthi et al
EPI_ISL_417482	Institute of Microbiology, Universidad San Francisco de Quito	Institute of Microbiology, Universidad San Francisco de Quito	Sully Márquez et al
EPI_ISL_471267	Hospital IESS Babahoyo	Institute of Microbiology, Universidad San Francisco de Quito	Sully Márquez et al
EPI_ISL_477015	Institute of Microbiology, Universidad San Francisco de Quito	Institute of Microbiology, Universidad San Francisco de Quito	Sully Márquez et al
EPI_ISL_660531	Institute of Microbiology, Universidad San Francisco de Quito	Institute of Microbiology, Universidad San Francisco de Quito	Sully Márquez et al
EPI_ISL_660532	Institute of Microbiology, Universidad San Francisco de Quito	Institute of Microbiology, Universidad San Francisco de Quito	Sully Márquez et al
EPI_ISL_660533	Institute of Microbiology, Universidad San Francisco de Quito	Institute of Microbiology, Universidad San Francisco de Quito	Sully Márquez et al
EPI_ISL_660534	Institute of Microbiology, Universidad San Francisco de Quito	Institute of Microbiology, Universidad San Francisco de Quito	Sully Márquez et al
EPI_ISL_660535	Institute of Microbiology, Universidad San Francisco de Quito	Institute of Microbiology, Universidad San Francisco de Quito	Sully Márquez et al
EPI_ISL_660536	Institute of Microbiology, Universidad San Francisco de Quito	Institute of Microbiology, Universidad San Francisco de Quito	Sully Márquez et al
EPI_ISL_660538	Institute of Microbiology, Universidad San Francisco de Quito	Institute of Microbiology, Universidad San Francisco de Quito	Sully Márquez et al
EPI_ISL_728202	Institute of Microbiology, Universidad San Francisco de Quito	Institute of Microbiology, Universidad San Francisco de Quito	Sully Márquez et al
EPI_ISL_728204	Institute of Microbiology, Universidad San Francisco de Quito	Institute of Microbiology, Universidad San Francisco de Quito	Sully Márquez et al
EPI_ISL_803098	Institute of Microbiology, Universidad San Francisco de Quito	Institute of Microbiology, Universidad San Francisco de Quito	Sully Márquez et al
EPI_ISL_1372080	Microbiology Department , Late Shri Lakhiram Agrawal Memory Hospital & College, Raigarh, Chhattisgarh	Institute of Life Sciences - INSACOG	Sunil K. Raghav et al
EPI_ISL_1372081	Microbiology Department , Late Shri Lakhiram Agrawal Memory Hospital & College, Raigarh, Chhattisgarh	Institute of Life Sciences - INSACOG	Sunil K. Raghav et al
EPI_ISL_1372086	Immunogenomics lab, Institute of Life Sciences, Bhubaneswar	Institute of Life Sciences - INSACOG	Sunil K. Raghav et al

EPI_ISL_1372087	Immunogenomics lab, Institute of Life Sciences, Bhubaneswar	Institute of Life Sciences - INSACOG	Sunil K. Raghav et al
EPI_ISL_1663490	Capital hospital, Bhubaneswar	Institute of Life Sciences - INSACOG	Sunil K. Raghav et al
EPI_ISL_1663491	Capital hospital, Bhubaneswar	Institute of Life Sciences - INSACOG	Sunil K. Raghav et al
EPI_ISL_1663492	Capital hospital, Bhubaneswar	Institute of Life Sciences - INSACOG	Sunil K. Raghav et al
EPI_ISL_1663538	MGM Medical College, Jamshedpur	Institute of Life Sciences - INSACOG	Sunil K. Raghav et al
EPI_ISL_1063848	REGIONAL VRDL, ICMR-RMRC BBSR	Immunogenomics lab, Institute of Life Sciences, Bhubaneswar	Sunil K. Raghav et al
EPI_ISL_481161	Immunogenomics lab, Institute of Life Sciences, Bhubaneswar	Immunogenomics lab, Institute of Life Sciences, Bhubaneswar	Sunil Raghav et al
EPI_ISL_718291	Institute for Medical Research, Infectious Disease Research Centre, National Institutes of Health, Ministry of Health Malaysia	Institute for Medical Research, Infectious Disease Research Centre, National Institutes of Health, Ministry of Health Malaysia	Suppiah J et al
EPI_ISL_718300	Institute for Medical Research, Infectious Disease Research Centre, National Institutes of Health, Ministry of Health Malaysia	Institute for Medical Research, Infectious Disease Research Centre, National Institutes of Health, Ministry of Health Malaysia	Suppiah J et al
EPI_ISL_877329	University College Sedaya International (UCSI University)	Institute for Medical Research, Infectious Disease Research Centre, National Institutes of Health, Ministry of Health Malaysia	Suppiah J et al
EPI_ISL_718284	Institute for Medical Research, Infectious Disease Research Centre, National Institutes of Health, Ministry of Health Malaysia	Institute for Medical Research, Infectious Disease Research Centre, National Institutes of Health, Ministry of Health Malaysia	Suppiah J et al
EPI_ISL_728157	Institute for Medical Research, Infectious Disease Research Centre, National Institutes of Health, Ministry of Health Malaysia	Institute for Medical Research, Infectious Disease Research Centre, National Institutes of Health, Ministry of Health Malaysia	Suppiah J et al
EPI_ISL_728161	Institute for Medical Research, Infectious Disease Research Centre, National Institutes of Health, Ministry of Health Malaysia	Institute for Medical Research, Infectious Disease Research Centre, National Institutes of Health, Ministry of Health Malaysia	Suppiah J et al
EPI_ISL_728165	Institute for Medical Research, Infectious Disease Research Centre, National Institutes of Health, Ministry of Health Malaysia	Institute for Medical Research, Infectious Disease Research Centre, National Institutes of Health, Ministry of Health Malaysia	Suppiah J et al
EPI_ISL_728241	Institute for Medical Research, Infectious Disease Research Centre, National Institutes of Health, Ministry of Health Malaysia	Institute for Medical Research, Infectious Disease Research Centre, National Institutes of Health, Ministry of Health Malaysia	Suppiah J et al
EPI_ISL_728242	Institute for Medical Research, Infectious Disease Research Centre, National Institutes of Health, Ministry of Health Malaysia	Institute for Medical Research, Infectious Disease Research Centre, National Institutes of Health, Ministry of Health Malaysia	Suppiah J et al
EPI_ISL_728248	Institute for Medical Research, Infectious Disease Research Centre, National Institutes of Health, Ministry of Health Malaysia	Institute for Medical Research, Infectious Disease Research Centre, National Institutes of Health, Ministry of Health Malaysia	Suppiah J et al
EPI_ISL_489994	Institute for Medical Research, Infectious Disease Research Centre, National Institutes of Health, Ministry of Health Malaysia	Institute for Medical Research, Infectious Disease Research Centre, National Institutes of Health, Ministry of Health Malaysia	Suppiah J et al
EPI_ISL_718270	Institute for Medical Research, Infectious Disease Research Centre, National Institutes of Health, Ministry of Health Malaysia	Institute for Medical Research, Infectious Disease Research Centre, National Institutes of Health, Ministry of Health Malaysia	Suppiah J et al
EPI_ISL_718274	Institute for Medical Research, Infectious Disease Research Centre, National Institutes of Health, Ministry of Health Malaysia	Institute for Medical Research, Infectious Disease Research Centre, National Institutes of Health, Ministry of Health Malaysia	Suppiah J et al
EPI_ISL_718272	Institute for Medical Research, Infectious Disease Research Centre, National Institutes of Health, Ministry of Health Malaysia	Institute for Medical Research, Infectious Disease Research Centre, National Institutes of Health, Ministry of Health Malaysia	Suppiah J et al
EPI_ISL_718275	Institute for Medical Research, Infectious Disease Research Centre, National Institutes of Health, Ministry of Health Malaysia	Institute for Medical Research, Infectious Disease Research Centre, National Institutes of Health, Ministry of Health Malaysia	Suppiah J et al
EPI_ISL_718276	Institute for Medical Research, Infectious Disease Research Centre, National Institutes of Health, Ministry of Health Malaysia	Institute for Medical Research, Infectious Disease Research Centre, National Institutes of Health, Ministry of Health Malaysia	Suppiah J et al
EPI_ISL_718278	Institute for Medical Research, Infectious Disease Research Centre, National Institutes of Health, Ministry of Health Malaysia	Institute for Medical Research, Infectious Disease Research Centre, National Institutes of Health, Ministry of Health Malaysia	Suppiah J et al

EPI_ISL_718279	Institute for Medical Research, Infectious Disease Research Centre, National Institutes of Health, Ministry of Health Malaysia	Institute for Medical Research, Infectious Disease Research Centre, National Institutes of Health, Ministry of Health Malaysia	Suppiah J et al
EPI_ISL_718311	Institute for Medical Research, Infectious Disease Research Centre, National Institutes of Health, Ministry of Health Malaysia	Institute for Medical Research, Infectious Disease Research Centre, National Institutes of Health, Ministry of Health Malaysia	Suppiah J et al
EPI_ISL_1186028	Institute for Medical Research, Infectious Disease Research Centre, National Institutes of Health, Ministry of Health Malaysia	Institute for Medical Research, Infectious Disease Research Centre, National Institutes of Health, Ministry of Health Malaysia	Suppiah J et al
EPI_ISL_1585270	Labuan Hospital	Institute for Medical Research, Infectious Disease Research Centre, National Institutes of Health, Ministry of Health Malaysia	Suppiah J et al
EPI_ISL_644672	Institute for Medical Research, Infectious Disease Research Centre, National Institutes of Health, Ministry of Health Malaysia	Institute for Medical Research, Infectious Disease Research Centre, National Institutes of Health, Ministry of Health Malaysia	Suppiah J et al
EPI_ISL_1424130	Institute for Medical Research, Infectious Disease Research Centre, National Institutes of Health, Ministry of Health Malaysia	Institute for Medical Research, Infectious Disease Research Centre, National Institutes of Health, Ministry of Health Malaysia	Suppiah J et al
EPI_ISL_1263458	Institute for Medical Research, Infectious Disease Research Centre, National Institutes of Health, Ministry of Health Malaysia	Institute for Medical Research, Infectious Disease Research Centre, National Institutes of Health, Ministry of Health Malaysia	Suppiah J et al
EPI_ISL_1424143	Institute for Medical Research, Infectious Disease Research Centre, National Institutes of Health, Ministry of Health Malaysia	Institute for Medical Research, Infectious Disease Research Centre, National Institutes of Health, Ministry of Health Malaysia	Suppiah J et al
EPI_ISL_1424466	Institute for Medical Research, Infectious Disease Research Centre, National Institutes of Health, Ministry of Health Malaysia	Institute for Medical Research, Infectious Disease Research Centre, National Institutes of Health, Ministry of Health Malaysia	Suppiah J et al
EPI_ISL_1264762	Institute for Medical Research, Infectious Disease Research Centre, National Institutes of Health, Ministry of Health Malaysia	Institute for Medical Research, Infectious Disease Research Centre, National Institutes of Health, Ministry of Health Malaysia	Suppiah J et al
EPI_ISL_1424471	Institute for Medical Research, Infectious Disease Research Centre, National Institutes of Health, Ministry of Health Malaysia	Institute for Medical Research, Infectious Disease Research Centre, National Institutes of Health, Ministry of Health Malaysia	Suppiah J et al
EPI_ISL_1424470	Institute for Medical Research, Infectious Disease Research Centre, National Institutes of Health, Ministry of Health Malaysia	Institute for Medical Research, Infectious Disease Research Centre, National Institutes of Health, Ministry of Health Malaysia	Suppiah J et al
EPI_ISL_1424468	Institute for Medical Research, Infectious Disease Research Centre, National Institutes of Health, Ministry of Health Malaysia	Institute for Medical Research, Infectious Disease Research Centre, National Institutes of Health, Ministry of Health Malaysia	Suppiah J et al
EPI_ISL_1424467	Institute for Medical Research, Infectious Disease Research Centre, National Institutes of Health, Ministry of Health Malaysia	Institute for Medical Research, Infectious Disease Research Centre, National Institutes of Health, Ministry of Health Malaysia	Suppiah J et al
EPI_ISL_1424513	Institute for Medical Research, Infectious Disease Research Centre, National Institutes of Health, Ministry of Health Malaysia	Institute for Medical Research, Infectious Disease Research Centre, National Institutes of Health, Ministry of Health Malaysia	Suppiah J et al
EPI_ISL_1583006	Institute for Medical Research, Infectious Disease Research Centre, National Institutes of Health, Ministry of Health Malaysia	Institute for Medical Research, Infectious Disease Research Centre, National Institutes of Health, Ministry of Health Malaysia	Suppiah J et al
EPI_ISL_1583007	Institute for Medical Research, Infectious Disease Research Centre, National Institutes of Health, Ministry of Health Malaysia	Institute for Medical Research, Infectious Disease Research Centre, National Institutes of Health, Ministry of Health Malaysia	Suppiah J et al
EPI_ISL_912397	Institute for Medical Research, Infectious Disease Research Centre, National Institutes of Health, Ministry of Health Malaysia	Institute for Medical Research, Infectious Disease Research Centre, National Institutes of Health, Ministry of Health Malaysia	Suppiah J et al
EPI_ISL_944096	Institute for Medical Research, Infectious Disease Research Centre, National Institutes of Health, Ministry of Health Malaysia	Institute for Medical Research, Infectious Disease Research Centre, National Institutes of Health, Ministry of Health Malaysia	Suppiah J et al
EPI_ISL_944103	Institute for Medical Research, Infectious Disease Research Centre, National Institutes of Health, Ministry of Health Malaysia	Institute for Medical Research, Infectious Disease Research Centre, National Institutes of Health, Ministry of Health Malaysia	Suppiah J et al
EPI_ISL_944102	Institute for Medical Research, Infectious Disease Research Centre, National Institutes of Health, Ministry of Health Malaysia	Institute for Medical Research, Infectious Disease Research Centre, National Institutes of Health, Ministry of Health Malaysia	Suppiah J et al
EPI_ISL_944104	Institute for Medical Research, Infectious Disease Research Centre, National Institutes of Health, Ministry of Health Malaysia	Institute for Medical Research, Infectious Disease Research Centre, National Institutes of Health, Ministry of Health Malaysia	Suppiah J et al

EPI_ISL_944099	Institute for Medical Research, Infectious Disease Research Centre, National Institutes of Health, Ministry of Health Malaysia	Institute for Medical Research, Infectious Disease Research Centre, National Institutes of Health, Ministry of Health Malaysia	Suppiah J et al
EPI_ISL_1138750	Institute for Medical Research, Infectious Disease Research Centre, National Institutes of Health, Ministry of Health Malaysia	Institute for Medical Research, Infectious Disease Research Centre, National Institutes of Health, Ministry of Health Malaysia	Suppiah J et al
EPI_ISL_1138598	Institute for Medical Research, Infectious Disease Research Centre, National Institutes of Health, Ministry of Health Malaysia	Institute for Medical Research, Infectious Disease Research Centre, National Institutes of Health, Ministry of Health Malaysia	Suppiah J et al
EPI_ISL_1263540	Institute for Medical Research, Infectious Disease Research Centre, National Institutes of Health, Ministry of Health Malaysia	Institute for Medical Research, Infectious Disease Research Centre, National Institutes of Health, Ministry of Health Malaysia	Suppiah J et al
EPI_ISL_1265161	Institute for Medical Research, Infectious Disease Research Centre, National Institutes of Health, Ministry of Health Malaysia	Institute for Medical Research, Infectious Disease Research Centre, National Institutes of Health, Ministry of Health Malaysia	Suppiah J et al
EPI_ISL_490094	Institute for Medical Research, Infectious Disease Research Centre, National Institutes of Health, Ministry of Health Malaysia	Institute for Medical Research, Infectious Disease Research Centre, National Institutes of Health, Ministry of Health Malaysia	Suppiah J et al
EPI_ISL_490096	Institute for Medical Research, Infectious Disease Research Centre, National Institutes of Health, Ministry of Health Malaysia	Institute for Medical Research, Infectious Disease Research Centre, National Institutes of Health, Ministry of Health Malaysia	Suppiah J et al
EPI_ISL_490099	Institute for Medical Research, Infectious Disease Research Centre, National Institutes of Health, Ministry of Health Malaysia	Institute for Medical Research, Infectious Disease Research Centre, National Institutes of Health, Ministry of Health Malaysia	Suppiah J et al
EPI_ISL_718269	Institute for Medical Research, Infectious Disease Research Centre, National Institutes of Health, Ministry of Health Malaysia	Institute for Medical Research, Infectious Disease Research Centre, National Institutes of Health, Ministry of Health Malaysia	Suppiah J et al
EPI_ISL_936494	Hospital Enche Besar Hajjah Khalsom Kluang	Institute for Medical Research, Infectious Disease Research Centre, National Institutes of Health, Ministry of Health Malaysia	Suppiah J et al
EPI_ISL_936496	Institute for Medical Research, Infectious Disease Research Centre, National Institutes of Health, Ministry of Health Malaysia	Institute for Medical Research, Infectious Disease Research Centre, National Institutes of Health, Ministry of Health Malaysia	Suppiah J et al
EPI_ISL_430443	Institute for Medical Research, Infectious Disease Research Centre, National Institutes of Health, Ministry of Health Malaysia	Institute for Medical Research, Infectious Disease Research Centre, National Institutes of Health, Ministry of Health Malaysia	Suppiah.J et al
EPI_ISL_430441	Institute for Medical Research, Infectious Disease Research Centre, National Institutes of Health, Ministry of Health Malaysia	Institute for Medical Research, Infectious Disease Research Centre, National Institutes of Health, Ministry of Health Malaysia	Suppiah.J et al
EPI_ISL_430444	Institute for Medical Research, Infectious Disease Research Centre, National Institutes of Health, Ministry of Health Malaysia	Institute for Medical Research, Infectious Disease Research Centre, National Institutes of Health, Ministry of Health Malaysia	Suppiah.J et al
EPI_ISL_745162	Elsies River CHC wc ERP	National Health Laboratory Service (NHLS), Tygerberg	Susan Engelbrecht et al
EPI_ISL_745185	Tygerberg Hospital wc TBH	National Health Laboratory Service (NHLS), Tygerberg	Susan Engelbrecht et al
EPI_ISL_912401	PathCare	National Health Laboratory Service (NHLS), Tygerberg	Susan Engelbrecht et al
EPI_ISL_1591408	Division of Medical Virology, National Health Laboratory Service (NHLS), Tygerberg Hospital / Stellenbosch University	Division of Medical Virology, Stellenbosch University and NHLS Tygerberg Hospital	Susan Engelbrecht et al
EPI_ISL_1502148	Division of Medical Virology, National Health Laboratory Service (NHLS), Tygerberg Hospital / Stellenbosch University	Division of Medical Virology, Stellenbosch University and NHLS Tygerberg Hospital	Susan Engelbrecht et al
EPI_ISL_1502167	Division of Medical Virology, National Health Laboratory Service (NHLS), Tygerberg Hospital / Stellenbosch University	Division of Medical Virology, Stellenbosch University and NHLS Tygerberg Hospital	Susan Engelbrecht et al
EPI_ISL_491296	Instituto Gulbenkian de Ciência	Instituto Gulbenkian de Ciência	Susana Ladeiro et al
EPI_ISL_855569	Department of Virology, Principal Military Hospital of Instruction of Tunis	Bundeswehr Institute of Microbiology	Susann Handrick et al
EPI_ISL_855560	Department of Virology, Principal Military Hospital of Instruction of Tunis	Bundeswehr Institute of Microbiology	Susann Handrick et al
EPI_ISL_855557	Department of Virology, Principal Military Hospital of Instruction of Tunis	Bundeswehr Institute of Microbiology	Susann Handrick et al

EPI_ISL_1661454	Microvida	Microvida	Suzan D. Pas et al
EPI_ISL_510535	Molecular Virology, Instituto Carlos Chagas / Fiocruz Paraná	Universidade Federal do Parana (UFPR)	Suzukawa et al
EPI_ISL_1617677	Swedish national genomic surveillance program of SARS-CoV-2	The Public Health Agency of Sweden	Swedish national genomic surveillance prog
EPI_ISL_1606241	Swedish national genomic surveillance program of SARS-CoV-2	The Public Health Agency of Sweden	Swedish national genomic surveillance prog
EPI_ISL_1364175	Swedish national genomic surveillance program of SARS-CoV-2	The Public Health Agency of Sweden	Swedish national genomic surveillance prog
EPI_ISL_1618017	Swedish national genomic surveillance program of SARS-CoV-2	The Public Health Agency of Sweden	Swedish national genomic surveillance prog
EPI_ISL_1601927	Swedish national genomic surveillance program of SARS-CoV-2	The Public Health Agency of Sweden	Swedish national genomic surveillance prog
EPI_ISL_1605767	Swedish national genomic surveillance program of SARS-CoV-2	The Public Health Agency of Sweden	Swedish national genomic surveillance prog
EPI_ISL_1606420	Swedish national genomic surveillance program of SARS-CoV-2	The Public Health Agency of Sweden	Swedish national genomic surveillance prog
EPI_ISL_1604430	Swedish national genomic surveillance program of SARS-CoV-2	The Public Health Agency of Sweden	Swedish national genomic surveillance prog
EPI_ISL_1290087	Swedish national genomic surveillance program of SARS-CoV-2	The Public Health Agency of Sweden	Swedish national genomic surveillance prog
EPI_ISL_1290603	Swedish national genomic surveillance program of SARS-CoV-2	The Public Health Agency of Sweden	Swedish national genomic surveillance prog
EPI_ISL_1290605	Swedish national genomic surveillance program of SARS-CoV-2	The Public Health Agency of Sweden	Swedish national genomic surveillance prog
EPI_ISL_1200012	Swedish national genomic surveillance program of SARS-CoV-2	The Public Health Agency of Sweden	Swedish national genomic surveillance prog
EPI_ISL_1200011	Swedish national genomic surveillance program of SARS-CoV-2	The Public Health Agency of Sweden	Swedish national genomic surveillance prog
EPI_ISL_833497	Klinik Apotek Dein, Jakarta, Indonesia	Biosafety Level-3 Laboratory, Indonesian Institute of Sciences (LIPI)	Syam Budi Iryanto et al
EPI_ISL_560587	Hopital	National Reference Center for Viruses of Respiratory Infections, Institut Pasteur, Paris	Sylvie Behillil et al
EPI_ISL_560590	Hopital	National Reference Center for Viruses of Respiratory Infections, Institut Pasteur, Paris	Sylvie Behillil et al
EPI_ISL_560636	Hospital	National Reference Center for Viruses of Respiratory Infections, Institut Pasteur, Paris	Sylvie Behillil et al
EPI_ISL_560637	Labo Analyses Med	National Reference Center for Viruses of Respiratory Infections, Institut Pasteur, Paris	Sylvie Behillil et al
EPI_ISL_593911	Hospital, Le Chesnay Cédex	National Reference Center for Viruses of Respiratory Infections, Institut Pasteur, Paris	Sylvie Behillil et al
EPI_ISL_593929	Labo Analyses Med, Puteaux	National Reference Center for Viruses of Respiratory Infections, Institut Pasteur, Paris	Sylvie Behillil et al
EPI_ISL_1627241	Wojewodzka Stacja Sanitarno-Epidemiologiczna w Olsztynie, Laboratorium Badan Epidemiologiczno-Klinicznych	Wojewodzka Stacja Sanitarno-Epidemiologiczna w Olsztynie, Laboratorium Badan Epidemiologiczno-Klinicznych	Sylwia Krzetowska et al
EPI_ISL_1627090	Pro-Vitam Diagnostics and Research Laboratory	Pro-Vitam Diagnostics and Research Laboratory	Szilard N. Fejer et al
EPI_ISL_582032	Institute of Human Genetics, Polish Academy of Sciences	Institute of Human Genetics, Polish Academy of Sciences	Szymon Hryhorowicz et al
EPI_ISL_485399	Institute of Human Genetics, Polish Academy of Sciences	Institute of Human Genetics, Polish Academy of Sciences	Szymon Hryhorowicz et al
EPI_ISL_455432	Instituto de Diagnostico y Referencia Epidemiologicos (INDRE)	Instituto de Diagnostico y Referencia Epidemiologicos (INDRE)	Taboada Ramírez Blanca. Ramirez-Gonzale
EPI_ISL_847827	COVID-19 National Reference Laboratory	COVID-19 National Reference Laboratory	Tahmineh Jalali et al

ɡram of SARS-CoV-2 et al
ɡram of SARS-CoV-2 et al
ɡram of SARS-CoV-2 et al
ɡram of SARS-CoV-2 et al
ɡram of SARS-CoV-2 et al
ɡram of SARS-CoV-2 et al
ɡram of SARS-CoV-2 et al
ɡram of SARS-CoV-2 et al
ɡram of SARS-CoV-2 et al
ɡram of SARS-CoV-2 et al
ɡram of SARS-CoV-2 et al
ɡram of SARS-CoV-2 et al
ɡram of SARS-CoV-2 et al

ez Ernesto et al

EPI_ISL_429676	Central Public Health Laboratory/Octávio Magalhães Institute (IOM) from the Ezequiel Dias Foundation (FUNED)	Instituto Octávio Magalhães / Fundação Ezequiel Dias (IOM/Funed)	Talita Adelino et al
EPI_ISL_429679	Central Public Health Laboratory/Octávio Magalhães Institute (IOM) from the Ezequiel Dias Foundation (FUNED)	Instituto Octávio Magalhães / Fundação Ezequiel Dias (IOM/Funed)	Talita Adelino et al
EPI_ISL_429665	Central Public Health Laboratory/Octávio Magalhães Institute (IOM) from the Ezequiel Dias Foundation (FUNED)	Instituto Octávio Magalhães / Fundação Ezequiel Dias (IOM/Funed)	Talita Adelino et al
EPI_ISL_429684	Central Public Health Laboratory/Octávio Magalhães Institute (IOM) from the Ezequiel Dias Foundation (FUNED)	Instituto Octávio Magalhães / Fundação Ezequiel Dias (IOM/Funed)	Talita Adelino et al
EPI_ISL_429688	Central Public Health Laboratory/Octávio Magalhães Institute (IOM) from the Ezequiel Dias Foundation (FUNED)	Instituto Octávio Magalhães / Fundação Ezequiel Dias (IOM/Funed)	Talita Adelino et al
EPI_ISL_429689	Central Public Health Laboratory/Octávio Magalhães Institute (IOM) from the Ezequiel Dias Foundation (FUNED)	Instituto Octávio Magalhães / Fundação Ezequiel Dias (IOM/Funed)	Talita Adelino et al
EPI_ISL_429701	Central Public Health Laboratory/Octávio Magalhães Institute (IOM) from the Ezequiel Dias Foundation (FUNED)	Instituto Octávio Magalhães / Fundação Ezequiel Dias (IOM/Funed)	Talita Adelino et al
EPI_ISL_429671	Central Public Health Laboratory/Octávio Magalhães Institute (IOM) from the Ezequiel Dias Foundation (FUNED)	Instituto Octávio Magalhães / Fundação Ezequiel Dias (IOM/Funed)	Talita Adelino et al
EPI_ISL_429672	Central Public Health Laboratory/Octávio Magalhães Institute (IOM) from the Ezequiel Dias Foundation (FUNED)	Instituto Octávio Magalhães / Fundação Ezequiel Dias (IOM/Funed)	Talita Adelino et al
EPI_ISL_943561	National Institute of Laboratory Medicine and Referral Center	Genomic Research Lab, BCSIR	Tanjina Akhtar Banu et al
EPI_ISL_1544143	SSG Medical College, Baroda	Gujarat Biotechnology Research Centre	Tanuja B. Javadekar et al
EPI_ISL_651545	Oxford Viromics, NDM, University of Oxford; Oxford University Hospitals; Basingstoke and North Hampshire Hospital	COVID-19 Genomics UK (COG-UK) Consortium	Tanya Golubchik et al
EPI_ISL_906851	Respiratory Viruses Branch, Centers for Disease Control and Prevention	Respiratory Viruses Branch, Centers for Disease Control and Prevention	Tao et al
EPI_ISL_477169	Department for Virology, Molecular Biology and Genome Research, R. G. Lugar Center for Public Health Research, National Center for Disease Control and Public Health (NCDC) of Georgia.	Department for Virology, Molecular Biology and Genome Research, R. G. Lugar Center for Public Health Research, National Center for Disease Control and Public Health (NCDC) of Georgia.	Tata Imnadze et al
EPI_ISL_1280095	Department of Genetics, Medirex	Laboratory of Genomics and Bioinformatics, Comenius University Science Park	Tatiana Sedláčková et al
EPI_ISL_1280161	Department of Genetics, Medirex	Laboratory of Genomics and Bioinformatics, Comenius University Science Park	Tatiana Sedláčková et al
EPI_ISL_1280115	Department of Genetics, Medirex	Laboratory of Genomics and Bioinformatics, Comenius University Science Park	Tatiana Sedláčková et al
EPI_ISL_1280124	Department of Genetics, Medirex	Laboratory of Genomics and Bioinformatics, Comenius University Science Park	Tatiana Sedláčková et al
EPI_ISL_1280140	Department of Genetics, Medirex	Laboratory of Genomics and Bioinformatics, Comenius University Science Park	Tatiana Sedláčková et al
EPI_ISL_1280146	Department of Genetics, Medirex	Laboratory of Genomics and Bioinformatics, Comenius University Science Park	Tatiana Sedláčková et al
EPI_ISL_1280151	Department of Genetics, Medirex	Laboratory of Genomics and Bioinformatics, Comenius University Science Park	Tatiana Sedláčková et al
EPI_ISL_1280152	Department of Genetics, Medirex	Laboratory of Genomics and Bioinformatics, Comenius University Science Park	Tatiana Sedláčková et al

EPI_ISL_1280121	Department of Genetics, Medirex	Laboratory of Genomics and Bioinformatics, Comenius University Science Park	Tatiana Sedláčková et al
EPI_ISL_1599311	Public Health Authority of the Slovak Republic	Laboratory of Genomics and Bioinformatics, Comenius University Science Park	Tatiana Sedláčková et al
EPI_ISL_1599337	Public Health Authority of the Slovak Republic	Laboratory of Genomics and Bioinformatics, Comenius University Science Park	Tatiana Sedláčková et al
EPI_ISL_1653929	Public Health Authority of the Slovak Republic	Laboratory of Genomics and Bioinformatics, Comenius University Science Park	Tatiana Sedláčková et al
EPI_ISL_1647652	Public Health Authority of the Slovak Republic	Laboratory of Genomics and Bioinformatics, Comenius University Science Park	Tatiana Sedláčková et al
EPI_ISL_1647755	Public Health Authority of the Slovak Republic	Laboratory of Genomics and Bioinformatics, Comenius University Science Park	Tatiana Sedláčková et al
EPI_ISL_1647718	Public Health Authority of the Slovak Republic	Laboratory of Genomics and Bioinformatics, Comenius University Science Park	Tatiana Sedláčková et al
EPI_ISL_1647687	Public Health Authority of the Slovak Republic	Laboratory of Genomics and Bioinformatics, Comenius University Science Park	Tatiana Sedláčková et al
EPI_ISL_1401572	Public Health Authority of the Slovak Republic	Laboratory of Genomics and Bioinformatics, Comenius University Science Park	Tatiana Sedláčková et al
EPI_ISL_1599275	Public Health Authority of the Slovak Republic	Laboratory of Genomics and Bioinformatics, Comenius University Science Park	Tatiana Sedláčková et al
EPI_ISL_1524015	Public Health Authority of the Slovak Republic	Laboratory of Genomics and Bioinformatics, Comenius University Science Park	Tatiana Sedláčková et al
EPI_ISL_1524032	Public Health Authority of the Slovak Republic	Laboratory of Genomics and Bioinformatics, Comenius University Science Park	Tatiana Sedláčková et al
EPI_ISL_407079	Lapland Central Hospital	Department of Virology, University of Helsinki and Helsinki University Hospital, Helsinki, Finland	Teemu Smura et al
EPI_ISL_1496712	Department of Virology and Immunology, University of Helsinki and Helsinki University Hospital, Huslab Finland	Department of Virology, Faculty of Medicine, University of Helsinki, Helsinki, Finland	Teemu Smura et al
EPI_ISL_481533	Department of Virology and Immunology, University of Helsinki and Helsinki University Hospital, Huslab Finland	Department of Virology, Faculty of Medicine, University of Helsinki, Helsinki, Finland	Teemu Smura et al
EPI_ISL_1218646	Department of Virology and Immunology, University of Helsinki and Helsinki University Hospital, Huslab Finland	Department of Virology, Faculty of Medicine, University of Helsinki, Helsinki, Finland	Teemu Smura et al
EPI_ISL_1218648	Department of Virology and Immunology, University of Helsinki and Helsinki University Hospital, Huslab Finland	Department of Virology, Faculty of Medicine, University of Helsinki, Helsinki, Finland	Teemu Smura et al
EPI_ISL_732569	Department of Virology and Immunology, University of Helsinki and Helsinki University Hospital, Huslab Finland	Department of Virology, Faculty of Medicine, University of Helsinki, Helsinki, Finland	Teemu Smura et al
EPI_ISL_1595762	Department of Virology and Immunology, University of Helsinki and Helsinki University Hospital, Huslab Finland	Department of Virology, Faculty of Medicine, University of Helsinki, Helsinki, Finland	Teemu Smura et al
EPI_ISL_1595773	Department of Virology and Immunology, University of Helsinki and Helsinki University Hospital, Huslab Finland	Department of Virology, Faculty of Medicine, University of Helsinki, Helsinki, Finland	Teemu Smura et al
EPI_ISL_1595775	Department of Virology and Immunology, University of Helsinki and Helsinki University Hospital, Huslab Finland	Department of Virology, Faculty of Medicine, University of Helsinki, Helsinki, Finland	Teemu Smura et al
EPI_ISL_1595791	Department of Virology and Immunology, University of Helsinki and Helsinki University Hospital, Huslab Finland	Department of Virology, Faculty of Medicine, University of Helsinki, Helsinki, Finland	Teemu Smura et al

EPI_ISL_756247	Department of Virology and Immunology, University of Helsinki and Helsinki University Hospital, Huslab Finland	Department of Virology, Faculty of Medicine, University of Helsinki, Helsinki, Finland	Teemu Smura et al
EPI_ISL_737210	Department of Virology and Immunology, University of Helsinki and Helsinki University Hospital, Huslab Finland	Department of Virology, Faculty of Medicine, University of Helsinki, Helsinki, Finland	Teemu Smura et al
EPI_ISL_737228	Department of Virology and Immunology, University of Helsinki and Helsinki University Hospital, Huslab Finland	Department of Virology, Faculty of Medicine, University of Helsinki, Helsinki, Finland	Teemu Smura et al
EPI_ISL_1217198	Department of Virology and Immunology, University of Helsinki and Helsinki University Hospital, Huslab Finland	Department of Virology, Faculty of Medicine, University of Helsinki, Helsinki, Finland	Teemu Smura et al
EPI_ISL_737234	Department of Virology and Immunology, University of Helsinki and Helsinki University Hospital, Huslab Finland	Department of Virology, Faculty of Medicine, University of Helsinki, Helsinki, Finland	Teemu Smura et al
EPI_ISL_1497002	Department of Virology and Immunology, University of Helsinki and Helsinki University Hospital, Huslab Finland	Department of Virology, Faculty of Medicine, University of Helsinki, Helsinki, Finland	Teemu Smura et al
EPI_ISL_1219684	Department of Virology and Immunology, University of Helsinki and Helsinki University Hospital, Huslab Finland	Department of Virology, Faculty of Medicine, University of Helsinki, Helsinki, Finland	Teemu Smura et al
EPI_ISL_1219696	Department of Virology and Immunology, University of Helsinki and Helsinki University Hospital, Huslab Finland	Department of Virology, Faculty of Medicine, University of Helsinki, Helsinki, Finland	Teemu Smura et al
EPI_ISL_1219721	Department of Virology and Immunology, University of Helsinki and Helsinki University Hospital, Huslab Finland	Department of Virology, Faculty of Medicine, University of Helsinki, Helsinki, Finland	Teemu Smura et al
EPI_ISL_757302	Department of Virology and Immunology, University of Helsinki and Helsinki University Hospital, Huslab Finland	Department of Virology, Faculty of Medicine, University of Helsinki, Helsinki, Finland	Teemu Smura et al
EPI_ISL_756284	Department of Virology and Immunology, University of Helsinki and Helsinki University Hospital, Huslab Finland	Department of Virology, Faculty of Medicine, University of Helsinki, Helsinki, Finland	Teemu Smura et al
EPI_ISL_757336	Department of Virology and Immunology, University of Helsinki and Helsinki University Hospital, Huslab Finland	Department of Virology, Faculty of Medicine, University of Helsinki, Helsinki, Finland	Teemu Smura et al
EPI_ISL_757377	Department of Virology and Immunology, University of Helsinki and Helsinki University Hospital, Huslab Finland	Department of Virology, Faculty of Medicine, University of Helsinki, Helsinki, Finland	Teemu Smura et al
EPI_ISL_757379	Department of Virology and Immunology, University of Helsinki and Helsinki University Hospital, Huslab Finland	Department of Virology, Faculty of Medicine, University of Helsinki, Helsinki, Finland	Teemu Smura et al
EPI_ISL_1233202	Department of Virology and Immunology, University of Helsinki and Helsinki University Hospital, Huslab Finland	Department of Virology, Faculty of Medicine, University of Helsinki, Helsinki, Finland	Teemu Smura et al
EPI_ISL_1233188	Department of Virology and Immunology, University of Helsinki and Helsinki University Hospital, Huslab Finland	Department of Virology, Faculty of Medicine, University of Helsinki, Helsinki, Finland	Teemu Smura et al
EPI_ISL_759839	Department of Virology and Immunology, University of Helsinki and Helsinki University Hospital, Huslab Finland	Department of Virology, Faculty of Medicine, University of Helsinki, Helsinki, Finland	Teemu Smura et al
EPI_ISL_759940	Department of Virology and Immunology, University of Helsinki and Helsinki University Hospital, Huslab Finland	Department of Virology, Faculty of Medicine, University of Helsinki, Helsinki, Finland	Teemu Smura et al
EPI_ISL_902786	Department of Virology and Immunology, University of Helsinki and Helsinki University Hospital, Huslab Finland	Department of Virology, Faculty of Medicine, University of Helsinki, Helsinki, Finland	Teemu Smura et al
EPI_ISL_1240078	Department of Virology and Immunology, University of Helsinki and Helsinki University Hospital, Huslab Finland	Department of Virology, Faculty of Medicine, University of Helsinki, Helsinki, Finland	Teemu Smura et al
EPI_ISL_1240150	Department of Virology and Immunology, University of Helsinki and Helsinki University Hospital, Huslab Finland	Department of Virology, Faculty of Medicine, University of Helsinki, Helsinki, Finland	Teemu Smura et al
EPI_ISL_1240168	Department of Virology and Immunology, University of Helsinki and Helsinki University Hospital, Huslab Finland	Department of Virology, Faculty of Medicine, University of Helsinki, Helsinki, Finland	Teemu Smura et al

EPI_ISL_1240166	Department of Virology and Immunology, University of Helsinki and Helsinki University Hospital, Huslab Finland	Department of Virology, Faculty of Medicine, University of Helsinki, Helsinki, Finland	Teemu Smura et al
EPI_ISL_862052	Department of Virology and Immunology, University of Helsinki and Helsinki University Hospital, Huslab Finland	Department of Virology, Faculty of Medicine, University of Helsinki, Helsinki, Finland	Teemu Smura et al
EPI_ISL_1240950	Department of Virology and Immunology, University of Helsinki and Helsinki University Hospital, Huslab Finland	Department of Virology, Faculty of Medicine, University of Helsinki, Helsinki, Finland	Teemu Smura et al
EPI_ISL_1240964	Department of Virology and Immunology, University of Helsinki and Helsinki University Hospital, Huslab Finland	Department of Virology, Faculty of Medicine, University of Helsinki, Helsinki, Finland	Teemu Smura et al
EPI_ISL_481705	Department of Virology and Immunology, University of Helsinki and Helsinki University Hospital, Huslab Finland	Department of Virology, Faculty of Medicine, University of Helsinki, Helsinki, Finland	Teemu Smura et al
EPI_ISL_1240972	Department of Virology and Immunology, University of Helsinki and Helsinki University Hospital, Huslab Finland	Department of Virology, Faculty of Medicine, University of Helsinki, Helsinki, Finland	Teemu Smura et al
EPI_ISL_1240984	Department of Virology and Immunology, University of Helsinki and Helsinki University Hospital, Huslab Finland	Department of Virology, Faculty of Medicine, University of Helsinki, Helsinki, Finland	Teemu Smura et al
EPI_ISL_481718	Department of Virology and Immunology, University of Helsinki and Helsinki University Hospital, Huslab Finland	Department of Virology, Faculty of Medicine, University of Helsinki, Helsinki, Finland	Teemu Smura et al
EPI_ISL_755956	Department of Virology and Immunology, University of Helsinki and Helsinki University Hospital, Huslab Finland	Department of Virology, Faculty of Medicine, University of Helsinki, Helsinki, Finland	Teemu Smura et al
EPI_ISL_412971	HUS Diagnostiikkakeskus, Hallinto	Department of Virology Faculty of Medicine, Medicum University of Helsinki	Teemu Smura et al
EPI_ISL_1279111	Department of Virology and Immunology, University of Helsinki and Helsinki University Hospital, Huslab Finland	Department of Virology, Faculty of Medicine, University of Helsinki, Helsinki, Finland	Teemu Smura et al
EPI_ISL_1279107	Department of Virology and Immunology, University of Helsinki and Helsinki University Hospital, Huslab Finland	Department of Virology, Faculty of Medicine, University of Helsinki, Helsinki, Finland	Teemu Smura et al
EPI_ISL_1278956	Department of Virology and Immunology, University of Helsinki and Helsinki University Hospital, Huslab Finland	Department of Virology, Faculty of Medicine, University of Helsinki, Helsinki, Finland	Teemu Smura et al
EPI_ISL_1278946	Department of Virology and Immunology, University of Helsinki and Helsinki University Hospital, Huslab Finland	Department of Virology, Faculty of Medicine, University of Helsinki, Helsinki, Finland	Teemu Smura et al
EPI_ISL_1278953	Department of Virology and Immunology, University of Helsinki and Helsinki University Hospital, Huslab Finland	Department of Virology, Faculty of Medicine, University of Helsinki, Helsinki, Finland	Teemu Smura et al
EPI_ISL_732630	Department of Virology and Immunology, University of Helsinki and Helsinki University Hospital, Huslab Finland	Department of Virology, Faculty of Medicine, University of Helsinki, Helsinki, Finland	Teemu Smura et al
EPI_ISL_737062	Department of Virology and Immunology, University of Helsinki and Helsinki University Hospital, Huslab Finland	Department of Virology, Faculty of Medicine, University of Helsinki, Helsinki, Finland	Teemu Smura et al
EPI_ISL_555586	Lighthouse Lab in Alderley Park	Wellcome Sanger Institute for the COVID-19 Genomics UK (COG-UK) consortium	The Lighthouse Lab in Alderley Park et al
EPI_ISL_558200	Lighthouse Lab in Alderley Park	Wellcome Sanger Institute for the COVID-19 Genomics UK (COG-UK) consortium	The Lighthouse Lab in Alderley Park et al
EPI_ISL_551467	Lighthouse Lab in Alderley Park	Wellcome Sanger Institute for the COVID-19 Genomics UK (COG-UK) consortium	The Lighthouse Lab in Alderley Park et al
EPI_ISL_552985	Lighthouse Lab in Alderley Park	Wellcome Sanger Institute for the COVID-19 Genomics UK (COG-UK) consortium	The Lighthouse Lab in Alderley Park et al
EPI_ISL_552955	Lighthouse Lab in Alderley Park	Wellcome Sanger Institute for the COVID-19 Genomics UK (COG-UK) consortium	The Lighthouse Lab in Alderley Park et al

EPI_ISL_555725	Lighthouse Lab in Alderley Park	Wellcome Sanger Institute for the COVID-19 Genomics UK (COG-UK) consortium	The Lighthouse Lab in Alderley Park et al
EPI_ISL_554084	Lighthouse Lab in Alderley Park	Wellcome Sanger Institute for the COVID-19 Genomics UK (COG-UK) consortium	The Lighthouse Lab in Alderley Park et al
EPI_ISL_553594	Lighthouse Lab in Alderley Park	Wellcome Sanger Institute for the COVID-19 Genomics UK (COG-UK) consortium	The Lighthouse Lab in Alderley Park et al
EPI_ISL_1084155	Lighthouse Lab in Milton Keynes	Wellcome Sanger Institute for the COVID-19 Genomics UK (COG-UK) Consortium	The Lighthouse Lab in Milton Keynes et al
EPI_ISL_1084042	Lighthouse Lab in Milton Keynes	Wellcome Sanger Institute for the COVID-19 Genomics UK (COG-UK) Consortium	The Lighthouse Lab in Milton Keynes et al
EPI_ISL_550429	Lighthouse Lab in Milton Keynes	Wellcome Sanger Institute for the COVID-19 Genomics UK (COG-UK) consortium	The Lighthouse Lab in Milton Keynes et al
EPI_ISL_552436	Lighthouse Lab in Milton Keynes	Wellcome Sanger Institute for the COVID-19 Genomics UK (COG-UK) consortium	The Lighthouse Lab in Milton Keynes et al
EPI_ISL_549833	Lighthouse Lab in Milton Keynes	Wellcome Sanger Institute for the COVID-19 Genomics UK (COG-UK) consortium	The Lighthouse Lab in Milton Keynes et al
EPI_ISL_631036	Lighthouse Lab in Milton Keynes	Wellcome Sanger Institute for the COVID-19 Genomics UK (COG-UK) consortium	The Lighthouse Lab in Milton Keynes et al
EPI_ISL_630998	Lighthouse Lab in Milton Keynes	Wellcome Sanger Institute for the COVID-19 Genomics UK (COG-UK) consortium	The Lighthouse Lab in Milton Keynes et al
EPI_ISL_760963	Lighthouse Lab in Milton Keynes	Wellcome Sanger Institute for the COVID-19 Genomics UK (COG-UK) Consortium	The Lighthouse Lab in Milton Keynes et al
EPI_ISL_762436	Lighthouse Lab in Milton Keynes	Wellcome Sanger Institute for the COVID-19 Genomics UK (COG-UK) Consortium	The Lighthouse Lab in Milton Keynes et al
EPI_ISL_762502	Lighthouse Lab in Milton Keynes	Wellcome Sanger Institute for the COVID-19 Genomics UK (COG-UK) Consortium	The Lighthouse Lab in Milton Keynes et al
EPI_ISL_924202	Virology Department, Sheffield Teaching Hospitals NHS Foundation Trust/Department of Infection, Immunity and Cardiovascular Disease, The Medical School, University of Sheffield	COVID-19 Genomics UK (COG-UK) Consortium	Thushan de Silva et al
EPI_ISL_420268	Virology Department, Sheffield Teaching Hospitals NHS Foundation Trust	Department of Infection, Immunity and Cardiovascular Disease, The Florey Institute, The Medical School, University of Sheffield	Thushan de Silva et al
EPI_ISL_1233628	Labormedizinisches Zentrum Dr Risch	University Hospital Basel, Clinical Bacteriology	Tim Roloff et al
EPI_ISL_1233629	Labormedizinisches Zentrum Dr Risch	University Hospital Basel, Clinical Bacteriology	Tim Roloff et al
EPI_ISL_1233630	Labormedizinisches Zentrum Dr Risch	University Hospital Basel, Clinical Bacteriology	Tim Roloff et al
EPI_ISL_1233663	Labormedizinisches Zentrum Dr Risch	University Hospital Basel, Clinical Bacteriology	Tim Roloff et al
EPI_ISL_1233664	Labormedizinisches Zentrum Dr Risch	University Hospital Basel, Clinical Bacteriology	Tim Roloff et al
EPI_ISL_1233691	Labormedizinisches Zentrum Dr Risch	University Hospital Basel, Clinical Bacteriology	Tim Roloff et al
EPI_ISL_1233692	Labormedizinisches Zentrum Dr Risch	University Hospital Basel, Clinical Bacteriology	Tim Roloff et al
EPI_ISL_1299921	Labormedizinisches Zentrum Dr Risch	University Hospital Basel, Clinical Bacteriology	Tim Roloff et al
EPI_ISL_1299915	Labormedizinisches Zentrum Dr Risch	University Hospital Basel, Clinical Bacteriology	Tim Roloff et al

EPI_ISL_1036127	Labormedizinisches Zentrum Dr Risch	University Hospital Basel, Clinical Bacteriology	Tim Roloff et al
EPI_ISL_1036070	Labormedizinisches Zentrum Dr Risch	University Hospital Basel, Clinical Bacteriology	Tim Roloff et al
EPI_ISL_830810	University Hospital Basel, Clinical Virology	University Hospital Basel, Clinical Bacteriology	Tim Roloff et al
EPI_ISL_1014691	University Hospital Basel, Clinical Virology	University Hospital Basel, Clinical Bacteriology	Tim Roloff et al
EPI_ISL_450408	unknown	Microbiology	To et al
EPI_ISL_1260768	National Laboratory for Health, Environment and Food	Department for Public Health Microbiology Ljubljana, National Laboratory for Health, Environment and Food	Tom Koritnik et al
EPI_ISL_1402180	Department of Public Health Microbiology Ljubljana, National Laboratory for Health, Environment and Food	Department of Public Health Microbiology Ljubljana, National Laboratory for Health, Environment and Food	Tom Koritnik et al
EPI_ISL_1654321	Department of Public Health Microbiology Ljubljana, National Laboratory for Health, Environment and Food	Department for Public Health Microbiology Ljubljana, National Laboratory for Health, Environment and Food	Tom Koritnik et al
EPI_ISL_636460	Institute of Microbiology and Immunology, Faculty of Medicine, University of Ljubljana	Institute of Microbiology and Immunology, Faculty of Medicine, University of Ljubljana	Tomaž Mark Zorec et al
EPI_ISL_635272	Institute of Microbiology and Immunology, Faculty of Medicine, University of Ljubljana	Institute of Microbiology and Immunology, Faculty of Medicine, University of Ljubljana	Tomaž Mark Zorec et al
EPI_ISL_635279	Institute of Microbiology and Immunology, Faculty of Medicine, University of Ljubljana	Institute of Microbiology and Immunology, Faculty of Medicine, University of Ljubljana	Tomaž Mark Zorec et al
EPI_ISL_635283	Institute of Microbiology and Immunology, Faculty of Medicine, University of Ljubljana	Institute of Microbiology and Immunology, Faculty of Medicine, University of Ljubljana	Tomaž Mark Zorec et al
EPI_ISL_635297	Institute of Microbiology and Immunology, Faculty of Medicine, University of Ljubljana	Institute of Microbiology and Immunology, Faculty of Medicine, University of Ljubljana	Tomaž Mark Zorec et al
EPI_ISL_458235	KU Leuven, Rega Institute, Clinical and Epidemiological Virology	KU Leuven, Rega Institute, Clinical and Epidemiological Virology	Tony Wawina-Bokalanga et al
EPI_ISL_464075	KU Leuven, Rega Institute, Clinical and Epidemiological Virology	KU Leuven, Rega Institute, Clinical and Epidemiological Virology	Tony Wawina-Bokalanga et al
EPI_ISL_734637	UZ Leuven, National Reference Laboratory for Coronaviruses, Laboratory Medicine, Leuven, Belgium	KU Leuven, Rega Institute, Clinical and Epidemiological Virology	Tony Wawina-Bokalanga et al
EPI_ISL_734645	UZ Leuven, National Reference Laboratory for Coronaviruses, Laboratory Medicine, Leuven, Belgium	KU Leuven, Rega Institute, Clinical and Epidemiological Virology	Tony Wawina-Bokalanga et al
EPI_ISL_734687	UZ Leuven, National Reference Laboratory for Coronaviruses, Laboratory Medicine, Leuven, Belgium	KU Leuven, Rega Institute, Clinical and Epidemiological Virology	Tony Wawina-Bokalanga et al
EPI_ISL_734720	UZ Leuven, National Reference Laboratory for Coronaviruses, Laboratory Medicine, Leuven, Belgium	KU Leuven, Rega Institute, Clinical and Epidemiological Virology	Tony Wawina-Bokalanga et al
EPI_ISL_477007	KU Leuven, Rega Institute, Clinical and Epidemiological Virology	KU Leuven, Rega Institute, Clinical and Epidemiological Virology	Tony Wawina-Bokalanga et al
EPI_ISL_734730	UZ Leuven, National Reference Laboratory for Coronaviruses, Laboratory Medicine, Leuven, Belgium	KU Leuven, Rega Institute, Clinical and Epidemiological Virology	Tony Wawina-Bokalanga et al
EPI_ISL_734732	UZ Leuven, National Reference Laboratory for Coronaviruses, Laboratory Medicine, Leuven, Belgium	KU Leuven, Rega Institute, Clinical and Epidemiological Virology	Tony Wawina-Bokalanga et al
EPI_ISL_734733	UZ Leuven, National Reference Laboratory for Coronaviruses, Laboratory Medicine, Leuven, Belgium	KU Leuven, Rega Institute, Clinical and Epidemiological Virology	Tony Wawina-Bokalanga et al
EPI_ISL_734763	UZ Leuven, National Reference Laboratory for Coronaviruses, Laboratory Medicine, Leuven, Belgium	KU Leuven, Rega Institute, Clinical and Epidemiological Virology	Tony Wawina-Bokalanga et al

EPI_ISL_734764	UZ Leuven, National Reference Laboratory for Coronaviruses, Laboratory Medicine, Leuven, Belgium	KU Leuven, Rega Institute, Clinical and Epidemiological Virology	Tony Wawina-Bokalanga et al
EPI_ISL_734768	UZ Leuven, National Reference Laboratory for Coronaviruses, Laboratory Medicine, Leuven, Belgium	KU Leuven, Rega Institute, Clinical and Epidemiological Virology	Tony Wawina-Bokalanga et al
EPI_ISL_734782	UZ Leuven, National Reference Laboratory for Coronaviruses, Laboratory Medicine, Leuven, Belgium	KU Leuven, Rega Institute, Clinical and Epidemiological Virology	Tony Wawina-Bokalanga et al
EPI_ISL_522350	KU Leuven, Rega Institute, Clinical and Epidemiological Virology	KU Leuven, Rega Institute, Clinical and Epidemiological Virology	Tony Wawina-Bokalanga et al
EPI_ISL_734825	UZ Leuven, National Reference Laboratory for Coronaviruses, Laboratory Medicine, Leuven, Belgium	KU Leuven, Rega Institute, Clinical and Epidemiological Virology	Tony Wawina-Bokalanga et al
EPI_ISL_734865	UZ Leuven, National Reference Laboratory for Coronaviruses, Laboratory Medicine, Leuven, Belgium	KU Leuven, Rega Institute, Clinical and Epidemiological Virology	Tony Wawina-Bokalanga et al
EPI_ISL_735016	UZ Leuven, National Reference Laboratory for Coronaviruses, Laboratory Medicine, Leuven, Belgium	KU Leuven, Rega Institute, Clinical and Epidemiological Virology	Tony Wawina-Bokalanga et al
EPI_ISL_735085	UZ Leuven, National Reference Laboratory for Coronaviruses, Laboratory Medicine, Leuven, Belgium	KU Leuven, Rega Institute, Clinical and Epidemiological Virology	Tony Wawina-Bokalanga et al
EPI_ISL_735087	UZ Leuven, National Reference Laboratory for Coronaviruses, Laboratory Medicine, Leuven, Belgium	KU Leuven, Rega Institute, Clinical and Epidemiological Virology	Tony Wawina-Bokalanga et al
EPI_ISL_1322686	KU Leuven, Rega Institute, Clinical and Epidemiological Virology	KU Leuven, Rega Institute, Clinical and Epidemiological Virology	Tony Wawina-Bokalanga et al
EPI_ISL_1322689	KU Leuven, Rega Institute, Clinical and Epidemiological Virology	KU Leuven, Rega Institute, Clinical and Epidemiological Virology	Tony Wawina-Bokalanga et al
EPI_ISL_1380845	KU Leuven, Rega Institute, Clinical and Epidemiological Virology	KU Leuven, Rega Institute, Clinical and Epidemiological Virology	Tony Wawina-Bokalanga et al
EPI_ISL_1382423	KU Leuven, Rega Institute, Clinical and Epidemiological Virology	KU Leuven, Rega Institute, Clinical and Epidemiological Virology	Tony Wawina-Bokalanga et al
EPI_ISL_1382789	KU Leuven, Rega Institute, Clinical and Epidemiological Virology	KU Leuven, Rega Institute, Clinical and Epidemiological Virology	Tony Wawina-Bokalanga et al
EPI_ISL_966940	Technical Support Units for Scientific Research (UATRS), National Centre for Scientific and Technical Research (CNRST)	Technical Support Units for Scientific Research (UATRS), National Centre for Scientific and Technical Research (CNRST)	Touil et al
EPI_ISL_1205370	Arizona State Public Health Laboratory	Arizona State Public Health Laboratory	Trung Huynh et al
EPI_ISL_1184565	Arizona State Public Health Laboratory	Arizona State Public Health Laboratory	Trung Huynh et al
EPI_ISL_407084	Department of Virology III, National Institute of Infectious Diseases	Pathogen Genomics Center, National Institute of Infectious Diseases	Tsuyoshi Sekizuka et al
EPI_ISL_416568	Japanese Quarantine Stations	Pathogen Genomics Center, National Institute of Infectious Diseases	Tsuyoshi Sekizuka et al
EPI_ISL_416613	Japanese Quarantine Stations	Pathogen Genomics Center, National Institute of Infectious Diseases	Tsuyoshi Sekizuka et al
EPI_ISL_416615	Japanese Quarantine Stations	Pathogen Genomics Center, National Institute of Infectious Diseases	Tsuyoshi Sekizuka et al
EPI_ISL_591352	Pathogen Genomics Center, National Institute of Infectious Diseases	Pathogen Genomics Center, National Institute of Infectious Diseases	Tsuyoshi Sekizuka et al
EPI_ISL_591372	Pathogen Genomics Center, National Institute of Infectious Diseases	Pathogen Genomics Center, National Institute of Infectious Diseases	Tsuyoshi Sekizuka et al
EPI_ISL_591390	Pathogen Genomics Center, National Institute of Infectious Diseases	Pathogen Genomics Center, National Institute of Infectious Diseases	Tsuyoshi Sekizuka et al
EPI_ISL_591402	Pathogen Genomics Center, National Institute of Infectious Diseases	Pathogen Genomics Center, National Institute of Infectious Diseases	Tsuyoshi Sekizuka et al
EPI_ISL_591411	Pathogen Genomics Center, National Institute of Infectious Diseases	Pathogen Genomics Center, National Institute of Infectious Diseases	Tsuyoshi Sekizuka et al
EPI_ISL_591449	Pathogen Genomics Center, National Institute of Infectious Diseases	Pathogen Genomics Center, National Institute of Infectious Diseases	Tsuyoshi Sekizuka et al

EPI_ISL_667560	Pathogen Genomics Center, National Institute of Infectious Diseases	Pathogen Genomics Center, National Institute of Infectious Diseases	Tsuyoshi Sekizuka et al
EPI_ISL_667626	Pathogen Genomics Center, National Institute of Infectious Diseases	Pathogen Genomics Center, National Institute of Infectious Diseases	Tsuyoshi Sekizuka et al
EPI_ISL_667639	Pathogen Genomics Center, National Institute of Infectious Diseases	Pathogen Genomics Center, National Institute of Infectious Diseases	Tsuyoshi Sekizuka et al
EPI_ISL_667649	Pathogen Genomics Center, National Institute of Infectious Diseases	Pathogen Genomics Center, National Institute of Infectious Diseases	Tsuyoshi Sekizuka et al
EPI_ISL_667650	Pathogen Genomics Center, National Institute of Infectious Diseases	Pathogen Genomics Center, National Institute of Infectious Diseases	Tsuyoshi Sekizuka et al
EPI_ISL_667663	Pathogen Genomics Center, National Institute of Infectious Diseases	Pathogen Genomics Center, National Institute of Infectious Diseases	Tsuyoshi Sekizuka et al
EPI_ISL_667738	Pathogen Genomics Center, National Institute of Infectious Diseases	Pathogen Genomics Center, National Institute of Infectious Diseases	Tsuyoshi Sekizuka et al
EPI_ISL_721601	Pathogen Genomics Center, National Institute of Infectious Diseases	Pathogen Genomics Center, National Institute of Infectious Diseases	Tsuyoshi Sekizuka et al
EPI_ISL_768654	Pathogen Genomics Center, National Institute of Infectious Diseases	Pathogen Genomics Center, National Institute of Infectious Diseases	Tsuyoshi Sekizuka et al
EPI_ISL_768709	Pathogen Genomics Center, National Institute of Infectious Diseases	Pathogen Genomics Center, National Institute of Infectious Diseases	Tsuyoshi Sekizuka et al
EPI_ISL_779207	Pathogen Genomics Center, National Institute of Infectious Diseases	Pathogen Genomics Center, National Institute of Infectious Diseases	Tsuyoshi Sekizuka et al
EPI_ISL_792680	Pathogen Genomics Center, National Institute of Infectious Diseases	Pathogen Genomics Center, National Institute of Infectious Diseases	Tsuyoshi Sekizuka et al
EPI_ISL_792682	Pathogen Genomics Center, National Institute of Infectious Diseases	Pathogen Genomics Center, National Institute of Infectious Diseases	Tsuyoshi Sekizuka et al
EPI_ISL_792683	Pathogen Genomics Center, National Institute of Infectious Diseases	Pathogen Genomics Center, National Institute of Infectious Diseases	Tsuyoshi Sekizuka et al
EPI_ISL_779245	Pathogen Genomics Center, National Institute of Infectious Diseases	Pathogen Genomics Center, National Institute of Infectious Diseases	Tsuyoshi Sekizuka et al
EPI_ISL_779246	Pathogen Genomics Center, National Institute of Infectious Diseases	Pathogen Genomics Center, National Institute of Infectious Diseases	Tsuyoshi Sekizuka et al
EPI_ISL_779676	Pathogen Genomics Center, National Institute of Infectious Diseases	Pathogen Genomics Center, National Institute of Infectious Diseases	Tsuyoshi Sekizuka et al
EPI_ISL_1425749	SARS-CoV-2 testing team, National Institute of Infectious Diseases	Pathogen Genomics Center, National Institute of Infectious Diseases	Tsuyoshi Sekizuka et al
EPI_ISL_1425720	SARS-CoV-2 testing team, National Institute of Infectious Diseases	Pathogen Genomics Center, National Institute of Infectious Diseases	Tsuyoshi Sekizuka et al
EPI_ISL_1425714	SARS-CoV-2 testing team, National Institute of Infectious Diseases	Pathogen Genomics Center, National Institute of Infectious Diseases	Tsuyoshi Sekizuka et al
EPI_ISL_408669	Dept. of Virology III, National Institute of Infectious Diseases	Pathogen Genomics Center, National Institute of Infectious Diseases	Tsuyoshi Sekizuka et al
EPI_ISL_410531	Dept. of Pathology, National Institute of Infectious Diseases	Pathogen Genomics Center, National Institute of Infectious Diseases	Tsuyoshi Sekizuka et al
EPI_ISL_410532	Dept. of Pathology, National Institute of Infectious Diseases	Pathogen Genomics Center, National Institute of Infectious Diseases	Tsuyoshi Sekizuka et al
EPI_ISL_479799	Sapporo City Institute of Public Health	Pathogen Genomics Center, National Institute of Infectious Diseases	Tsuyoshi Sekizuka et al
EPI_ISL_479800	Sapporo City Institute of Public Health	Pathogen Genomics Center, National Institute of Infectious Diseases	Tsuyoshi Sekizuka et al
EPI_ISL_684112	Pathogen Genomics Center, National Institute of Infectious Diseases	Pathogen Genomics Center, National Institute of Infectious Diseases	Tsuyoshi Sekizuka et al
EPI_ISL_684127	Gunma Prefectural Institute of Public Health and Environmental Sciences	Pathogen Genomics Center, National Institute of Infectious Diseases	Tsuyoshi Sekizuka et al
EPI_ISL_684147	Sakai City Institute of Public Health	Pathogen Genomics Center, National Institute of Infectious Diseases	Tsuyoshi Sekizuka et al
EPI_ISL_480044	Nagoya City Public Health Research Institute	Pathogen Genomics Center, National Institute of Infectious Diseases	Tsuyoshi Sekizuka et al
EPI_ISL_900909	Pathogen Genomics Center, National Institute of Infectious Diseases	Pathogen Genomics Center, National Institute of Infectious Diseases	Tsuyoshi Sekizuka et al

EPI_ISL_901462	Gunma Prefectural Institute of Public Health and Environmental Sciences	Pathogen Genomics Center, National Institute of Infectious Diseases	Tsuyoshi Sekizuka et al
EPI_ISL_901731	Pathogen Genomics Center, National Institute of Infectious Diseases	Pathogen Genomics Center, National Institute of Infectious Diseases	Tsuyoshi Sekizuka et al
EPI_ISL_898049	Sakai City Institute of Public Health	Pathogen Genomics Center, National Institute of Infectious Diseases	Tsuyoshi Sekizuka et al
EPI_ISL_896614	Pathogen Genomics Center, National Institute of Infectious Diseases	Pathogen Genomics Center, National Institute of Infectious Diseases	Tsuyoshi Sekizuka et al
EPI_ISL_892373	Pathogen Genomics Center, National Institute of Infectious Diseases	Pathogen Genomics Center, National Institute of Infectious Diseases	Tsuyoshi Sekizuka et al
EPI_ISL_892381	Pathogen Genomics Center, National Institute of Infectious Diseases	Pathogen Genomics Center, National Institute of Infectious Diseases	Tsuyoshi Sekizuka et al
EPI_ISL_892382	Pathogen Genomics Center, National Institute of Infectious Diseases	Pathogen Genomics Center, National Institute of Infectious Diseases	Tsuyoshi Sekizuka et al
EPI_ISL_892388	Pathogen Genomics Center, National Institute of Infectious Diseases	Pathogen Genomics Center, National Institute of Infectious Diseases	Tsuyoshi Sekizuka et al
EPI_ISL_892533	Osaka Institute of Public Health, Morinomiya Center	Pathogen Genomics Center, National Institute of Infectious Diseases	Tsuyoshi Sekizuka et al
EPI_ISL_892811	Pathogen Genomics Center, National Institute of Infectious Diseases	Pathogen Genomics Center, National Institute of Infectious Diseases	Tsuyoshi Sekizuka et al
EPI_ISL_685238	Pathogen Genomics Center, National Institute of Infectious Diseases	Pathogen Genomics Center, National Institute of Infectious Diseases	Tsuyoshi Sekizuka et al
EPI_ISL_1131143	Osaka Institute of Public Health, Morinomiya Center	Pathogen Genomics Center, National Institute of Infectious Diseases	Tsuyoshi Sekizuka et al
EPI_ISL_1127231	Pathogen Genomics Center, National Institute of Infectious Diseases	Pathogen Genomics Center, National Institute of Infectious Diseases	Tsuyoshi Sekizuka et al
EPI_ISL_1127124	Pathogen Genomics Center, National Institute of Infectious Diseases	Pathogen Genomics Center, National Institute of Infectious Diseases	Tsuyoshi Sekizuka et al
EPI_ISL_1426592	Sakai City Institute of Public Health	Pathogen Genomics Center, National Institute of Infectious Diseases	Tsuyoshi Sekizuka et al
EPI_ISL_1429772	Pathogen Genomics Center, National Institute of Infectious Diseases	Pathogen Genomics Center, National Institute of Infectious Diseases	Tsuyoshi Sekizuka et al
EPI_ISL_1429779	Pathogen Genomics Center, National Institute of Infectious Diseases	Pathogen Genomics Center, National Institute of Infectious Diseases	Tsuyoshi Sekizuka et al
EPI_ISL_1429787	Pathogen Genomics Center, National Institute of Infectious Diseases	Pathogen Genomics Center, National Institute of Infectious Diseases	Tsuyoshi Sekizuka et al
EPI_ISL_1429788	Pathogen Genomics Center, National Institute of Infectious Diseases	Pathogen Genomics Center, National Institute of Infectious Diseases	Tsuyoshi Sekizuka et al
EPI_ISL_1429790	Pathogen Genomics Center, National Institute of Infectious Diseases	Pathogen Genomics Center, National Institute of Infectious Diseases	Tsuyoshi Sekizuka et al
EPI_ISL_1430687	Saitama Prefectural Institute of Public Health	Pathogen Genomics Center, National Institute of Infectious Diseases	Tsuyoshi Sekizuka et al
EPI_ISL_1430688	Saitama Prefectural Institute of Public Health	Pathogen Genomics Center, National Institute of Infectious Diseases	Tsuyoshi Sekizuka et al
EPI_ISL_1430690	Saitama Prefectural Institute of Public Health	Pathogen Genomics Center, National Institute of Infectious Diseases	Tsuyoshi Sekizuka et al
EPI_ISL_1430697	SARS-CoV-2 testing team, National Institute of Infectious Diseases	Pathogen Genomics Center, National Institute of Infectious Diseases	Tsuyoshi Sekizuka et al
EPI_ISL_690818	Kanagawa Prefectural Institute of Public Health	Pathogen Genomics Center, National Institute of Infectious Diseases	Tsuyoshi Sekizuka et al
EPI_ISL_690824	Kanagawa Prefectural Institute of Public Health	Pathogen Genomics Center, National Institute of Infectious Diseases	Tsuyoshi Sekizuka et al
EPI_ISL_1430698	Saitama Prefectural Institute of Public Health	Pathogen Genomics Center, National Institute of Infectious Diseases	Tsuyoshi Sekizuka et al
EPI_ISL_690635	Pathogen Genomics Center, National Institute of Infectious Diseases	Pathogen Genomics Center, National Institute of Infectious Diseases	Tsuyoshi Sekizuka et al
EPI_ISL_690267	Pathogen Genomics Center, National Institute of Infectious Diseases	Pathogen Genomics Center, National Institute of Infectious Diseases	Tsuyoshi Sekizuka et al
EPI_ISL_690519	Fukuoka Institute of Health and Environmental Sciences	Pathogen Genomics Center, National Institute of Infectious Diseases	Tsuyoshi Sekizuka et al

EPI_ISL_688923	Fukuoka Institute of Health and Environmental Sciences	Pathogen Genomics Center, National Institute of Infectious Diseases	Tsuyoshi Sekizuka et al
EPI_ISL_689271	Pathogen Genomics Center, National Institute of Infectious Diseases	Pathogen Genomics Center, National Institute of Infectious Diseases	Tsuyoshi Sekizuka et al
EPI_ISL_689294	Pathogen Genomics Center, National Institute of Infectious Diseases	Pathogen Genomics Center, National Institute of Infectious Diseases	Tsuyoshi Sekizuka et al
EPI_ISL_686339	Pathogen Genomics Center, National Institute of Infectious Diseases	Pathogen Genomics Center, National Institute of Infectious Diseases	Tsuyoshi Sekizuka et al
EPI_ISL_686346	Pathogen Genomics Center, National Institute of Infectious Diseases	Pathogen Genomics Center, National Institute of Infectious Diseases	Tsuyoshi Sekizuka et al
EPI_ISL_686354	Pathogen Genomics Center, National Institute of Infectious Diseases	Pathogen Genomics Center, National Institute of Infectious Diseases	Tsuyoshi Sekizuka et al
EPI_ISL_902344	Toyama Institute of Health	Pathogen Genomics Center, National Institute of Infectious Diseases	Tsuyoshi Sekizuka et al
EPI_ISL_408665	Dept. of Virology III, National Institute of Infectious Diseases	Pathogen Genomics Center, National Institute of Infectious Diseases	Tsuyoshi Sekizuka et al
EPI_ISL_408666	Dept. of Virology III, National Institute of Infectious Diseases	Pathogen Genomics Center, National Institute of Infectious Diseases	Tsuyoshi Sekizuka et al
EPI_ISL_408667	Dept. of Virology III, National Institute of Infectious Diseases	Pathogen Genomics Center, National Institute of Infectious Diseases	Tsuyoshi Sekizuka et al
EPI_ISL_491298	Rural Health Unit - Calauan, Laguna	Research Institute for Tropical Medicine	Tujan et al
EPI_ISL_1122419	National Public Health Laboratory, National Centre for Infectious Diseases	National Virology Reference Laboratory	Tze Minn Mak et al
EPI_ISL_1122420	National Public Health Laboratory, National Centre for Infectious Diseases	National Virology Reference Laboratory	Tze Minn Mak et al
EPI_ISL_1122421	National Public Health Laboratory, National Centre for Infectious Diseases	National Virology Reference Laboratory	Tze Minn Mak et al
EPI_ISL_693471	Central Public Health Laboratory	National Public Health Laboratory, National Centre for Infectious Diseases	Tze Minn Mak et al
EPI_ISL_693480	Central Public Health Laboratory	National Public Health Laboratory, National Centre for Infectious Diseases	Tze Minn Mak et al
EPI_ISL_693481	Central Public Health Laboratory	National Public Health Laboratory, National Centre for Infectious Diseases	Tze Minn Mak et al
EPI_ISL_693482	Central Public Health Laboratory	National Public Health Laboratory, National Centre for Infectious Diseases	Tze Minn Mak et al
EPI_ISL_1322329	Central Public Health Laboratory	National Public Health Laboratory, National Centre for Infectious Diseases	Tze Minn Mak et al
EPI_ISL_693483	Central Public Health Laboratory	National Public Health Laboratory, National Centre for Infectious Diseases	Tze Minn Mak et al
EPI_ISL_1322330	Central Public Health Laboratory	National Public Health Laboratory, National Centre for Infectious Diseases	Tze Minn Mak et al
EPI_ISL_693472	Central Public Health Laboratory	National Public Health Laboratory, National Centre for Infectious Diseases	Tze Minn Mak et al
EPI_ISL_693473	Central Public Health Laboratory	National Public Health Laboratory, National Centre for Infectious Diseases	Tze Minn Mak et al
EPI_ISL_1307659	Central Public Health Laboratory	National Public Health Laboratory, National Centre for Infectious Diseases	Tze Minn Mak et al
EPI_ISL_1307660	Central Public Health Laboratory	National Public Health Laboratory, National Centre for Infectious Diseases	Tze Minn Mak et al
EPI_ISL_693474	Central Public Health Laboratory	National Public Health Laboratory, National Centre for Infectious Diseases	Tze Minn Mak et al
EPI_ISL_693475	Central Public Health Laboratory	National Public Health Laboratory, National Centre for Infectious Diseases	Tze Minn Mak et al
EPI_ISL_693476	Central Public Health Laboratory	National Public Health Laboratory, National Centre for Infectious Diseases	Tze Minn Mak et al
EPI_ISL_693477	Central Public Health Laboratory	National Public Health Laboratory, National Centre for Infectious Diseases	Tze Minn Mak et al
EPI_ISL_1307677	Central Public Health Laboratory	National Public Health Laboratory, National Centre for Infectious Diseases	Tze Minn Mak et al

EPI_ISL_693478	Central Public Health Laboratory	National Public Health Laboratory, National Centre for Infectious Diseases	Tze Minn Mak et al
EPI_ISL_693479	Central Public Health Laboratory	National Public Health Laboratory, National Centre for Infectious Diseases	Tze Minn Mak et al
EPI_ISL_574531	National Public Health Laboratory, National Centre for Infectious Diseases	National Public Health Laboratory, National Centre for Infectious Diseases	Tze Minn Mak et al
EPI_ISL_891244	National Public Health Laboratory, National Centre for Infectious Diseases	National Public Health Laboratory, National Centre for Infectious Diseases	Tze Minn Mak et al
EPI_ISL_574541	National Public Health Laboratory, National Centre for Infectious Diseases	National Public Health Laboratory, National Centre for Infectious Diseases	Tze Minn Mak et al
EPI_ISL_596478	National Public Health Laboratory, National Centre for Infectious Diseases	National Public Health Laboratory, National Centre for Infectious Diseases	Tze Minn Mak et al
EPI_ISL_596483	National Public Health Laboratory, National Centre for Infectious Diseases	National Public Health Laboratory, National Centre for Infectious Diseases	Tze Minn Mak et al
EPI_ISL_596488	National Public Health Laboratory, National Centre for Infectious Diseases	National Public Health Laboratory, National Centre for Infectious Diseases	Tze Minn Mak et al
EPI_ISL_626646	National Public Health Laboratory, National Centre for Infectious Diseases	National Public Health Laboratory, National Centre for Infectious Diseases	Tze Minn Mak et al
EPI_ISL_906840	National Public Health Laboratory, National Centre for Infectious Diseases	National Public Health Laboratory, National Centre for Infectious Diseases	Tze Minn Mak et al
EPI_ISL_937515	National Public Health Laboratory, National Centre for Infectious Diseases	National Public Health Laboratory, National Centre for Infectious Diseases	Tze Minn Mak et al
EPI_ISL_937518	National Public Health Laboratory, National Centre for Infectious Diseases	National Public Health Laboratory, National Centre for Infectious Diseases	Tze Minn Mak et al
EPI_ISL_937528	National Public Health Laboratory, National Centre for Infectious Diseases	National Public Health Laboratory, National Centre for Infectious Diseases	Tze Minn Mak et al
EPI_ISL_645115	National Public Health Laboratory, National Centre for Infectious Diseases	National Public Health Laboratory, National Centre for Infectious Diseases	Tze Minn Mak et al
EPI_ISL_645117	National Public Health Laboratory, National Centre for Infectious Diseases	National Public Health Laboratory, National Centre for Infectious Diseases	Tze Minn Mak et al
EPI_ISL_654813	National Public Health Laboratory, National Centre for Infectious Diseases	National Public Health Laboratory, National Centre for Infectious Diseases	Tze Minn Mak et al
EPI_ISL_693308	National Public Health Laboratory, National Centre for Infectious Diseases	National Public Health Laboratory, National Centre for Infectious Diseases	Tze Minn Mak et al
EPI_ISL_693314	National Public Health Laboratory, National Centre for Infectious Diseases	National Public Health Laboratory, National Centre for Infectious Diseases	Tze Minn Mak et al
EPI_ISL_693318	National Public Health Laboratory, National Centre for Infectious Diseases	National Public Health Laboratory, National Centre for Infectious Diseases	Tze Minn Mak et al
EPI_ISL_953388	National Public Health Laboratory, National Centre for Infectious Diseases	National Public Health Laboratory, National Centre for Infectious Diseases	Tze Minn Mak et al
EPI_ISL_728187	National Public Health Laboratory, National Centre for Infectious Diseases	National Public Health Laboratory, National Centre for Infectious Diseases	Tze Minn Mak et al
EPI_ISL_754079	National Public Health Laboratory, National Centre for Infectious Diseases	National Public Health Laboratory, National Centre for Infectious Diseases	Tze Minn Mak et al
EPI_ISL_981027	National Public Health Laboratory, National Centre for Infectious Diseases	National Public Health Laboratory, National Centre for Infectious Diseases	Tze Minn Mak et al
EPI_ISL_1034270	National Public Health Laboratory, National Centre for Infectious Diseases	National Public Health Laboratory, National Centre for Infectious Diseases	Tze Minn Mak et al
EPI_ISL_1164354	National Public Health Laboratory, National Centre for Infectious Diseases	National Public Health Laboratory, National Centre for Infectious Diseases	Tze Minn Mak et al
EPI_ISL_1367558	National Public Health Laboratory, National Centre for Infectious Diseases	National Public Health Laboratory, National Centre for Infectious Diseases	Tze Minn Mak et al
EPI_ISL_1524783	National Public Health Laboratory, National Centre for Infectious Diseases	National Public Health Laboratory, National Centre for Infectious Diseases	Tze Minn Mak et al
EPI_ISL_1524796	National Public Health Laboratory, National Centre for Infectious Diseases	National Public Health Laboratory, National Centre for Infectious Diseases	Tze Minn Mak et al
EPI_ISL_1519355	National Public Health Laboratory, National Centre for Infectious Diseases	National Public Health Laboratory, National Centre for Infectious Diseases	Tze Minn Mak et al
EPI_ISL_1543939	National Public Health Laboratory, National Centre for Infectious Diseases	National Public Health Laboratory, National Centre for Infectious Diseases	Tze Minn Mak et al

EPI_ISL_833376	National Public Health Laboratory, National Centre for Infectious Diseases	National Public Health Laboratory, National Centre for Infectious Diseases	Tze Minn Mak et al
EPI_ISL_1620144	National Public Health Laboratory, National Centre for Infectious Diseases	National Public Health Laboratory, National Centre for Infectious Diseases	Tze Minn Mak et al
EPI_ISL_1620131	National Public Health Laboratory, National Centre for Infectious Diseases	National Public Health Laboratory, National Centre for Infectious Diseases	Tze Minn Mak et al
EPI_ISL_1652091	National Public Health Laboratory, National Centre for Infectious Diseases	National Public Health Laboratory, National Centre for Infectious Diseases	Tze Minn Mak et al
EPI_ISL_833384	National Public Health Laboratory, National Centre for Infectious Diseases	National Public Health Laboratory, National Centre for Infectious Diseases	Tze Minn Mak et al
EPI_ISL_1652117	National Public Health Laboratory, National Centre for Infectious Diseases	National Public Health Laboratory, National Centre for Infectious Diseases	Tze Minn Mak et al
EPI_ISL_1652120	National Public Health Laboratory, National Centre for Infectious Diseases	National Public Health Laboratory, National Centre for Infectious Diseases	Tze Minn Mak et al
EPI_ISL_1652123	National Public Health Laboratory, National Centre for Infectious Diseases	National Public Health Laboratory, National Centre for Infectious Diseases	Tze Minn Mak et al
EPI_ISL_891236	National Public Health Laboratory, National Centre for Infectious Diseases	National Public Health Laboratory, National Centre for Infectious Diseases	Tze Minn Mak et al
EPI_ISL_1271944	Universidade Federal do Norte do Tocantins (UFNT)	Laboratório de Bioinformática e Biotecnologia (Labinftec/UFT)	Ueric José Borges de Souza et al
EPI_ISL_1272074	Universidade Federal do Norte do Tocantins (UFNT)	Laboratório de Bioinformática e Biotecnologia (Labinftec/UFT)	Ueric José Borges de Souza et al
EPI_ISL_1272076	Universidade Federal do Norte do Tocantins (UFNT)	Laboratório de Bioinformática e Biotecnologia (Labinftec/UFT)	Ueric José Borges de Souza et al
EPI_ISL_1272188	Universidade Federal do Norte do Tocantins (UFNT)	Laboratório de Bioinformática e Biotecnologia (Labinftec/UFT)	Ueric José Borges de Souza et al
EPI_ISL_1272236	Universidade Federal do Norte do Tocantins (UFNT)	Laboratório de Bioinformática e Biotecnologia (Labinftec/UFT)	Ueric José Borges de Souza et al
EPI_ISL_516934	Nicolae Testemitanu State University of Medicine and Pharmacy	International Centre for Genetic Engineering and Biotechnology (ICGEB) and ARGO Open Lab Platform for Genome Sequencing	Ulinici M et al
EPI_ISL_516938	Nicolae Testemitanu State University of Medicine and Pharmacy	International Centre for Genetic Engineering and Biotechnology (ICGEB) and ARGO Open Lab Platform for Genome Sequencing	Ulinici M et al
EPI_ISL_516936	Nicolae Testemitanu State University of Medicine and Pharmacy	International Centre for Genetic Engineering and Biotechnology (ICGEB) and ARGO Open Lab Platform for Genome Sequencing	Ulinici M et al
EPI_ISL_1373189	Protzer Lab	Protzer Lab, Institut für Medizinische Mikrobiologie und Hygiene, Gagneur Lab	Ulrike Protzer et al
EPI_ISL_1373184	Protzer Lab	Protzer Lab, Institut für Medizinische Mikrobiologie und Hygiene, Gagneur Lab	Ulrike Protzer et al
EPI_ISL_1373167	Protzer Lab	Protzer Lab, Institut für Medizinische Mikrobiologie und Hygiene, Gagneur Lab	Ulrike Protzer et al
EPI_ISL_1373258	Protzer Lab	Protzer Lab, Institut für Medizinische Mikrobiologie und Hygiene, Gagneur Lab	Ulrike Protzer et al
EPI_ISL_1373269	Protzer Lab	Protzer Lab, Institut für Medizinische Mikrobiologie und Hygiene, Gagneur Lab	Ulrike Protzer et al
EPI_ISL_1373163	Protzer Lab	Protzer Lab, Institut für Medizinische Mikrobiologie und Hygiene, Gagneur Lab	Ulrike Protzer et al
EPI_ISL_1373186	Protzer Lab	Protzer Lab, Institut für Medizinische Mikrobiologie und Hygiene, Gagneur Lab	Ulrike Protzer et al
EPI_ISL_1373276	Protzer Lab	Protzer Lab, Institut für Medizinische Mikrobiologie und Hygiene, Gagneur Lab	Ulrike Protzer et al
EPI_ISL_1373281	Protzer Lab	Protzer Lab, Institut für Medizinische Mikrobiologie und Hygiene, Gagneur Lab	Ulrike Protzer et al

EPI_ISL_1373222	Protzer Lab	Protzer Lab, Institut für Medizinische Mikrobiologie und Hygiene, Gagneur Lab	Ulrike Protzer et al
EPI_ISL_1373214	Protzer Lab	Protzer Lab, Institut für Medizinische Mikrobiologie und Hygiene, Gagneur Lab	Ulrike Protzer et al
EPI_ISL_1373294	Protzer Lab	Protzer Lab, Institut für Medizinische Mikrobiologie und Hygiene, Gagneur Lab	Ulrike Protzer et al
EPI_ISL_1373303	Protzer Lab	Protzer Lab, Institut für Medizinische Mikrobiologie und Hygiene, Gagneur Lab	Ulrike Protzer et al
EPI_ISL_1373229	Protzer Lab	Protzer Lab, Institut für Medizinische Mikrobiologie und Hygiene, Gagneur Lab	Ulrike Protzer et al
EPI_ISL_1373188	Protzer Lab	Protzer Lab, Institut für Medizinische Mikrobiologie und Hygiene, Gagneur Lab	Ulrike Protzer et al
EPI_ISL_1373177	Protzer Lab	Protzer Lab, Institut für Medizinische Mikrobiologie und Hygiene, Gagneur Lab	Ulrike Protzer et al
EPI_ISL_1373180	Protzer Lab	Protzer Lab, Institut für Medizinische Mikrobiologie und Hygiene, Gagneur Lab	Ulrike Protzer et al
EPI_ISL_466680	Nebraska Public Health Laboratory	UNMC COVID-19 Response Team	UNMC COVID-19 Response Team et al
EPI_ISL_959275	National Influenza Center, Virology Department	National Influenza Center	V Salimi et al
EPI_ISL_1014676	National Influenza Center, Virology Department	National Influenza Center	V Salimi et al
EPI_ISL_862075	National Influenza Center, Virology Department	National Influenza Center	V Salimi et al
EPI_ISL_1239127	Laboratório Central de Saúde Pública do Espírito Santo	Coordenação Geral de Laboratórios de Saúde Pública (CGLAB)	Vagner Fonseca et al
EPI_ISL_1239136	Laboratório Central de Saúde Pública do Espírito Santo	Coordenação Geral de Laboratórios de Saúde Pública (CGLAB)	Vagner Fonseca et al
EPI_ISL_1239117	Laboratório Central de Saúde Pública do Espírito Santo	Coordenação Geral de Laboratórios de Saúde Pública (CGLAB)	Vagner Fonseca et al
EPI_ISL_1239125	Laboratório Central de Saúde Pública do Espírito Santo	Coordenação Geral de Laboratórios de Saúde Pública (CGLAB)	Vagner Fonseca et al
EPI_ISL_1239135	Laboratório Central de Saúde Pública do Espírito Santo	Coordenação Geral de Laboratórios de Saúde Pública (CGLAB)	Vagner Fonseca et al
EPI_ISL_1239131	Laboratório Central de Saúde Pública do Espírito Santo	Coordenação Geral de Laboratórios de Saúde Pública (CGLAB)	Vagner Fonseca et al
EPI_ISL_1239126	Laboratório Central de Saúde Pública do Espírito Santo	Coordenação Geral de Laboratórios de Saúde Pública (CGLAB)	Vagner Fonseca et al
EPI_ISL_1239115	Laboratório Central de Saúde Pública do Espírito Santo	Coordenação Geral de Laboratórios de Saúde Pública (CGLAB)	Vagner Fonseca et al
EPI_ISL_1239133	Laboratório Central de Saúde Pública do Espírito Santo	Coordenação Geral de Laboratórios de Saúde Pública (CGLAB)	Vagner Fonseca et al
EPI_ISL_1239118	Laboratório Central de Saúde Pública do Espírito Santo	Coordenação Geral de Laboratórios de Saúde Pública (CGLAB)	Vagner Fonseca et al
EPI_ISL_1239128	Laboratório Central de Saúde Pública do Espírito Santo	Coordenação Geral de Laboratórios de Saúde Pública (CGLAB)	Vagner Fonseca et al
EPI_ISL_1239134	Laboratório Central de Saúde Pública do Espírito Santo	Coordenação Geral de Laboratórios de Saúde Pública (CGLAB)	Vagner Fonseca et al
EPI_ISL_1239120	Laboratório Central de Saúde Pública do Espírito Santo	Coordenação Geral de Laboratórios de Saúde Pública (CGLAB)	Vagner Fonseca et al
EPI_ISL_1239123	Laboratório Central de Saúde Pública do Espírito Santo	Coordenação Geral de Laboratórios de Saúde Pública (CGLAB)	Vagner Fonseca et al
EPI_ISL_1239132	Laboratório Central de Saúde Pública do Espírito Santo	Coordenação Geral de Laboratórios de Saúde Pública (CGLAB)	Vagner Fonseca et al

EPI_ISL_1239114	Laboratório Central de Saúde Pública do Espírito Santo	Coordenação Geral de Laboratórios de Saúde Pública (CGLAB)	Vagner Fonseca et al
EPI_ISL_1239122	Laboratório Central de Saúde Pública do Espírito Santo	Coordenação Geral de Laboratórios de Saúde Pública (CGLAB)	Vagner Fonseca et al
EPI_ISL_1239116	Laboratório Central de Saúde Pública do Espírito Santo	Coordenação Geral de Laboratórios de Saúde Pública (CGLAB)	Vagner Fonseca et al
EPI_ISL_1239121	Laboratório Central de Saúde Pública do Espírito Santo	Coordenação Geral de Laboratórios de Saúde Pública (CGLAB)	Vagner Fonseca et al
EPI_ISL_1239129	Laboratório Central de Saúde Pública do Espírito Santo	Coordenação Geral de Laboratórios de Saúde Pública (CGLAB)	Vagner Fonseca et al
EPI_ISL_1239130	Laboratório Central de Saúde Pública do Espírito Santo	Coordenação Geral de Laboratórios de Saúde Pública (CGLAB)	Vagner Fonseca et al
EPI_ISL_1182574	Fundação Ezequiel Dias (FUNED)	Coordenação Geral de Laboratórios de Saúde Pública (CGLAB/DAEVS/SVS/MS)	Vagner Fonseca et al
EPI_ISL_1182573	Fundação Ezequiel Dias (FUNED)	Coordenação Geral de Laboratórios de Saúde Pública (CGLAB/DAEVS/SVS/MS)	Vagner Fonseca et al
EPI_ISL_1182555	Fundação Ezequiel Dias (FUNED)	Coordenação Geral de Laboratórios de Saúde Pública (CGLAB/DAEVS/SVS/MS)	Vagner Fonseca et al
EPI_ISL_1182548	Fundação Ezequiel Dias (FUNED)	Coordenação Geral de Laboratórios de Saúde Pública (CGLAB/DAEVS/SVS/MS)	Vagner Fonseca et al
EPI_ISL_1182544	Fundação Ezequiel Dias (FUNED)	Coordenação Geral de Laboratórios de Saúde Pública (CGLAB/DAEVS/SVS/MS)	Vagner Fonseca et al
EPI_ISL_1182550	Fundação Ezequiel Dias (FUNED)	Coordenação Geral de Laboratórios de Saúde Pública (CGLAB/DAEVS/SVS/MS)	Vagner Fonseca et al
EPI_ISL_1182579	Fundação Ezequiel Dias (FUNED)	Coordenação Geral de Laboratórios de Saúde Pública (CGLAB/DAEVS/SVS/MS)	Vagner Fonseca et al
EPI_ISL_1182569	Fundação Ezequiel Dias (FUNED)	Coordenação Geral de Laboratórios de Saúde Pública (CGLAB/DAEVS/SVS/MS)	Vagner Fonseca et al
EPI_ISL_1182561	Fundação Ezequiel Dias (FUNED)	Coordenação Geral de Laboratórios de Saúde Pública (CGLAB/DAEVS/SVS/MS)	Vagner Fonseca et al
EPI_ISL_1182560	Fundação Ezequiel Dias (FUNED)	Coordenação Geral de Laboratórios de Saúde Pública (CGLAB/DAEVS/SVS/MS)	Vagner Fonseca et al
EPI_ISL_1182566	Fundação Ezequiel Dias (FUNED)	Coordenação Geral de Laboratórios de Saúde Pública (CGLAB/DAEVS/SVS/MS)	Vagner Fonseca et al
EPI_ISL_1182564	Fundação Ezequiel Dias (FUNED)	Coordenação Geral de Laboratórios de Saúde Pública (CGLAB/DAEVS/SVS/MS)	Vagner Fonseca et al
EPI_ISL_1182551	Fundação Ezequiel Dias (FUNED)	Coordenação Geral de Laboratórios de Saúde Pública (CGLAB/DAEVS/SVS/MS)	Vagner Fonseca et al
EPI_ISL_1182559	Fundação Ezequiel Dias (FUNED)	Coordenação Geral de Laboratórios de Saúde Pública (CGLAB/DAEVS/SVS/MS)	Vagner Fonseca et al
EPI_ISL_1182541	Fundação Ezequiel Dias (FUNED)	Coordenação Geral de Laboratórios de Saúde Pública (CGLAB/DAEVS/SVS/MS)	Vagner Fonseca et al
EPI_ISL_1239124	Fundação Ezequiel Dias	Coordenação Geral de Laboratórios de Saúde Pública (CGLAB)	Vagner Fonseca et al
EPI_ISL_1239139	Fundação Ezequiel Dias	Coordenação Geral de Laboratórios de Saúde Pública (CGLAB)	Vagner Fonseca et al
EPI_ISL_1182625	Fundação Ezequiel Dias (FUNED)	Coordenação Geral de Laboratórios de Saúde Pública (CGLAB/DAEVS/SVS/MS)	Vagner Fonseca et al

EPI_ISL_1182618	Fundação Ezequiel Dias (FUNED)	Coordenação Geral de Laboratórios de Saúde Pública (CGLAB/DAEVS/SVS/MS)	Vagner Fonseca et al
EPI_ISL_1182615	Fundação Ezequiel Dias (FUNED)	Coordenação Geral de Laboratórios de Saúde Pública (CGLAB/DAEVS/SVS/MS)	Vagner Fonseca et al
EPI_ISL_1182612	Fundação Ezequiel Dias (FUNED)	Coordenação Geral de Laboratórios de Saúde Pública (CGLAB/DAEVS/SVS/MS)	Vagner Fonseca et al
EPI_ISL_1182611	Fundação Ezequiel Dias (FUNED)	Coordenação Geral de Laboratórios de Saúde Pública (CGLAB/DAEVS/SVS/MS)	Vagner Fonseca et al
EPI_ISL_1182609	Fundação Ezequiel Dias (FUNED)	Coordenação Geral de Laboratórios de Saúde Pública (CGLAB/DAEVS/SVS/MS)	Vagner Fonseca et al
EPI_ISL_1182607	Fundação Ezequiel Dias (FUNED)	Coordenação Geral de Laboratórios de Saúde Pública (CGLAB/DAEVS/SVS/MS)	Vagner Fonseca et al
EPI_ISL_1182598	Fundação Ezequiel Dias (FUNED)	Coordenação Geral de Laboratórios de Saúde Pública (CGLAB/DAEVS/SVS/MS)	Vagner Fonseca et al
EPI_ISL_1182600	Fundação Ezequiel Dias (FUNED)	Coordenação Geral de Laboratórios de Saúde Pública (CGLAB/DAEVS/SVS/MS)	Vagner Fonseca et al
EPI_ISL_1182601	Fundação Ezequiel Dias (FUNED)	Coordenação Geral de Laboratórios de Saúde Pública (CGLAB/DAEVS/SVS/MS)	Vagner Fonseca et al
EPI_ISL_1182593	Fundação Ezequiel Dias (FUNED)	Coordenação Geral de Laboratórios de Saúde Pública (CGLAB/DAEVS/SVS/MS)	Vagner Fonseca et al
EPI_ISL_1182591	Fundação Ezequiel Dias (FUNED)	Coordenação Geral de Laboratórios de Saúde Pública (CGLAB/DAEVS/SVS/MS)	Vagner Fonseca et al
EPI_ISL_1182570	Fundação Ezequiel Dias (FUNED)	Coordenação Geral de Laboratórios de Saúde Pública (CGLAB/DAEVS/SVS/MS)	Vagner Fonseca et al
EPI_ISL_1182587	Fundação Ezequiel Dias (FUNED)	Coordenação Geral de Laboratórios de Saúde Pública (CGLAB/DAEVS/SVS/MS)	Vagner Fonseca et al
EPI_ISL_1182578	Fundação Ezequiel Dias (FUNED)	Coordenação Geral de Laboratórios de Saúde Pública (CGLAB/DAEVS/SVS/MS)	Vagner Fonseca et al
EPI_ISL_1182586	Fundação Ezequiel Dias (FUNED)	Coordenação Geral de Laboratórios de Saúde Pública (CGLAB/DAEVS/SVS/MS)	Vagner Fonseca et al
EPI_ISL_1182577	Fundação Ezequiel Dias (FUNED)	Coordenação Geral de Laboratórios de Saúde Pública (CGLAB/DAEVS/SVS/MS)	Vagner Fonseca et al
EPI_ISL_1182571	Laboratório Central do Estado do Paraná	Coordenação Geral de Laboratórios de Saúde Pública (CGLAB/DAEVS/SVS/MS)	Vagner Fonseca et al
EPI_ISL_1182575	Laboratório Central do Estado do Paraná	Coordenação Geral de Laboratórios de Saúde Pública (CGLAB/DAEVS/SVS/MS)	Vagner Fonseca et al
EPI_ISL_1182568	Laboratório Central do Estado do Paraná	Coordenação Geral de Laboratórios de Saúde Pública (CGLAB/DAEVS/SVS/MS)	Vagner Fonseca et al
EPI_ISL_1182567	Laboratório Central do Estado do Paraná	Coordenação Geral de Laboratórios de Saúde Pública (CGLAB/DAEVS/SVS/MS)	Vagner Fonseca et al
EPI_ISL_1182565	Laboratório Central do Estado do Paraná	Coordenação Geral de Laboratórios de Saúde Pública (CGLAB/DAEVS/SVS/MS)	Vagner Fonseca et al
EPI_ISL_1182546	Laboratório Central do Estado do Paraná	Coordenação Geral de Laboratórios de Saúde Pública (CGLAB/DAEVS/SVS/MS)	Vagner Fonseca et al

EPI_ISL_1182562	Laboratório Central do Estado do Paraná	Coordenação Geral de Laboratórios de Saúde Pública (CGLAB/DAEVS/SVS/MS)	Vagner Fonseca et al
EPI_ISL_1182563	Laboratório Central do Estado do Paraná	Coordenação Geral de Laboratórios de Saúde Pública (CGLAB/DAEVS/SVS/MS)	Vagner Fonseca et al
EPI_ISL_1182542	Laboratório Central do Estado do Paraná	Coordenação Geral de Laboratórios de Saúde Pública (CGLAB/DAEVS/SVS/MS)	Vagner Fonseca et al
EPI_ISL_1182624	Laboratório Central do Estado do Paraná	Coordenação Geral de Laboratórios de Saúde Pública (CGLAB/DAEVS/SVS/MS)	Vagner Fonseca et al
EPI_ISL_1182622	Laboratório Central do Estado do Paraná	Coordenação Geral de Laboratórios de Saúde Pública (CGLAB/DAEVS/SVS/MS)	Vagner Fonseca et al
EPI_ISL_1182617	Laboratório Central do Estado do Paraná	Coordenação Geral de Laboratórios de Saúde Pública (CGLAB/DAEVS/SVS/MS)	Vagner Fonseca et al
EPI_ISL_1182613	Laboratório Central do Estado do Paraná	Coordenação Geral de Laboratórios de Saúde Pública (CGLAB/DAEVS/SVS/MS)	Vagner Fonseca et al
EPI_ISL_1182614	Laboratório Central do Estado do Paraná	Coordenação Geral de Laboratórios de Saúde Pública (CGLAB/DAEVS/SVS/MS)	Vagner Fonseca et al
EPI_ISL_1182603	Laboratório Central do Estado do Paraná	Coordenação Geral de Laboratórios de Saúde Pública (CGLAB/DAEVS/SVS/MS)	Vagner Fonseca et al
EPI_ISL_1182604	Laboratório Central do Estado do Paraná	Coordenação Geral de Laboratórios de Saúde Pública (CGLAB/DAEVS/SVS/MS)	Vagner Fonseca et al
EPI_ISL_1182596	Laboratório Central do Estado do Paraná	Coordenação Geral de Laboratórios de Saúde Pública (CGLAB/DAEVS/SVS/MS)	Vagner Fonseca et al
EPI_ISL_1182594	Laboratório Central do Estado do Paraná	Coordenação Geral de Laboratórios de Saúde Pública (CGLAB/DAEVS/SVS/MS)	Vagner Fonseca et al
EPI_ISL_1182595	Laboratório Central do Estado do Paraná	Coordenação Geral de Laboratórios de Saúde Pública (CGLAB/DAEVS/SVS/MS)	Vagner Fonseca et al
EPI_ISL_1182592	Laboratório Central do Estado do Paraná	Coordenação Geral de Laboratórios de Saúde Pública (CGLAB/DAEVS/SVS/MS)	Vagner Fonseca et al
EPI_ISL_1182589	Laboratório Central do Estado do Paraná	Coordenação Geral de Laboratórios de Saúde Pública (CGLAB/DAEVS/SVS/MS)	Vagner Fonseca et al
EPI_ISL_1182588	Laboratório Central do Estado do Paraná	Coordenação Geral de Laboratórios de Saúde Pública (CGLAB/DAEVS/SVS/MS)	Vagner Fonseca et al
EPI_ISL_1182584	Laboratório Central do Estado do Paraná	Coordenação Geral de Laboratórios de Saúde Pública (CGLAB/DAEVS/SVS/MS)	Vagner Fonseca et al
EPI_ISL_1182583	Laboratório Central do Estado do Paraná	Coordenação Geral de Laboratórios de Saúde Pública (CGLAB/DAEVS/SVS/MS)	Vagner Fonseca et al
EPI_ISL_1182582	Laboratório Central do Estado do Paraná	Coordenação Geral de Laboratórios de Saúde Pública (CGLAB/DAEVS/SVS/MS)	Vagner Fonseca et al
EPI_ISL_1182580	Laboratório Central do Estado do Paraná	Coordenação Geral de Laboratórios de Saúde Pública (CGLAB/DAEVS/SVS/MS)	Vagner Fonseca et al
EPI_ISL_1182581	Laboratório Central do Estado do Paraná	Coordenação Geral de Laboratórios de Saúde Pública (CGLAB/DAEVS/SVS/MS)	Vagner Fonseca et al
EPI_ISL_1182576	Laboratório Central do Estado do Paraná	Coordenação Geral de Laboratórios de Saúde Pública (CGLAB/DAEVS/SVS/MS)	Vagner Fonseca et al

EPI_ISL_1182572	Laboratório Central do Estado do Paraná	Coordenação Geral de Laboratórios de Saúde Pública (CGLAB/DAEVS/SVS/MS)	Vagner Fonseca et al
EPI_ISL_1182557	Laboratório Central do Estado do Rio de Janeiro	Coordenação Geral de Laboratórios de Saúde Pública (CGLAB/DAEVS/SVS/MS)	Vagner Fonseca et al
EPI_ISL_1182556	Laboratório Central do Estado do Rio de Janeiro	Coordenação Geral de Laboratórios de Saúde Pública (CGLAB/DAEVS/SVS/MS)	Vagner Fonseca et al
EPI_ISL_1182552	Laboratório Central do Estado do Rio de Janeiro	Coordenação Geral de Laboratórios de Saúde Pública (CGLAB/DAEVS/SVS/MS)	Vagner Fonseca et al
EPI_ISL_1182554	Laboratório Central do Estado do Rio de Janeiro	Coordenação Geral de Laboratórios de Saúde Pública (CGLAB/DAEVS/SVS/MS)	Vagner Fonseca et al
EPI_ISL_1182553	Laboratório Central do Estado do Rio de Janeiro	Coordenação Geral de Laboratórios de Saúde Pública (CGLAB/DAEVS/SVS/MS)	Vagner Fonseca et al
EPI_ISL_1239112	Laboratório Central de Saúde Pública Noel Nutels	Coordenação Geral de Laboratórios de Saúde Pública (CGLAB)	Vagner Fonseca et al
EPI_ISL_1239113	Laboratório Central de Saúde Pública Noel Nutels	Coordenação Geral de Laboratórios de Saúde Pública (CGLAB)	Vagner Fonseca et al
EPI_ISL_1239111	Laboratório Central de Saúde Pública Noel Nutels	Coordenação Geral de Laboratórios de Saúde Pública (CGLAB)	Vagner Fonseca et al
EPI_ISL_1182621	Laboratório Central de Saúde Pública do Rio Grande do Sul	Coordenação Geral de Laboratórios de Saúde Pública (CGLAB/DAEVS/SVS/MS)	Vagner Fonseca et al
EPI_ISL_1182547	Laboratório Central de Saúde Pública do Rio Grande do Sul	Coordenação Geral de Laboratórios de Saúde Pública (CGLAB/DAEVS/SVS/MS)	Vagner Fonseca et al
EPI_ISL_1182623	Laboratório Central de Saúde Pública do Rio Grande do Sul	Coordenação Geral de Laboratórios de Saúde Pública (CGLAB/DAEVS/SVS/MS)	Vagner Fonseca et al
EPI_ISL_1182619	Laboratório Central de Saúde Pública do Rio Grande do Sul	Coordenação Geral de Laboratórios de Saúde Pública (CGLAB/DAEVS/SVS/MS)	Vagner Fonseca et al
EPI_ISL_1182620	Laboratório Central de Saúde Pública do Rio Grande do Sul	Coordenação Geral de Laboratórios de Saúde Pública (CGLAB/DAEVS/SVS/MS)	Vagner Fonseca et al
EPI_ISL_1182616	Laboratório Central de Saúde Pública do Rio Grande do Sul	Coordenação Geral de Laboratórios de Saúde Pública (CGLAB/DAEVS/SVS/MS)	Vagner Fonseca et al
EPI_ISL_1182606	Laboratório Central de Saúde Pública do Rio Grande do Sul	Coordenação Geral de Laboratórios de Saúde Pública (CGLAB/DAEVS/SVS/MS)	Vagner Fonseca et al
EPI_ISL_1182608	Laboratório Central de Saúde Pública do Rio Grande do Sul	Coordenação Geral de Laboratórios de Saúde Pública (CGLAB/DAEVS/SVS/MS)	Vagner Fonseca et al
EPI_ISL_1182610	Laboratório Central de Saúde Pública do Rio Grande do Sul	Coordenação Geral de Laboratórios de Saúde Pública (CGLAB/DAEVS/SVS/MS)	Vagner Fonseca et al
EPI_ISL_1182605	Laboratório Central de Saúde Pública do Rio Grande do Sul	Coordenação Geral de Laboratórios de Saúde Pública (CGLAB/DAEVS/SVS/MS)	Vagner Fonseca et al
EPI_ISL_1182599	Laboratório Central de Saúde Pública do Rio Grande do Sul	Coordenação Geral de Laboratórios de Saúde Pública (CGLAB/DAEVS/SVS/MS)	Vagner Fonseca et al
EPI_ISL_1182597	Laboratório Central de Saúde Pública do Rio Grande do Sul	Coordenação Geral de Laboratórios de Saúde Pública (CGLAB/DAEVS/SVS/MS)	Vagner Fonseca et al
EPI_ISL_1240640	Laboratório Central de Saúde Pública do Espírito Santo	Coordenação Geral de Laboratórios de Saúde Pública (CGLAB)	Vagner Fonseca et al.
EPI_ISL_1240641	Laboratório Central de Saúde Pública do Espírito Santo	Coordenação Geral de Laboratórios de Saúde Pública (CGLAB)	Vagner Fonseca et al.
EPI_ISL_1240639	Laboratório Central de Saúde Pública do Espírito Santo	Coordenação Geral de Laboratórios de Saúde Pública (CGLAB)	Vagner Fonseca et al.

EPI_ISL_1240642	Fundação Ezequiel Dias	Coordenação Geral de Laboratórios de Saúde Pública (CGLAB)	Vagner Fonseca et al.
EPI_ISL_417034	Laboratorio de Ecologia de Doencas Transmissiveis na Amazonia, Instituto Leonidas e Maria Deane - Fiocruz Amazonia	Laboratorio de Ecologia de Doencas Transmissiveis na Amazonia, Instituto Leonidas e Maria Deane - Fiocruz Amazonia	Valdinete Nascimento et al
EPI_ISL_1068078	Laboratorio de Ecologia de Doencas Transmissiveis na Amazonia, Instituto Leonidas e Maria Deane - Fiocruz Amazonia	Laboratorio de Ecologia de Doencas Transmissiveis na Amazonia, Instituto Leonidas e Maria Deane - Fiocruz Amazonia	Valdinete Nascimento et al
EPI_ISL_811148	Laboratorio de Ecologia de Doencas Transmissiveis na Amazonia, Instituto Leonidas e Maria Deane - Fiocruz Amazonia	Laboratorio de Ecologia de Doencas Transmissiveis na Amazonia, Instituto Leonidas e Maria Deane - Fiocruz Amazonia	Valdinete Nascimento et al
EPI_ISL_1068079	Laboratorio de Ecologia de Doencas Transmissiveis na Amazonia, Instituto Leonidas e Maria Deane - Fiocruz Amazonia	Laboratorio de Ecologia de Doencas Transmissiveis na Amazonia, Instituto Leonidas e Maria Deane - Fiocruz Amazonia	Valdinete Nascimento et al
EPI_ISL_1068082	Laboratorio de Ecologia de Doencas Transmissiveis na Amazonia, Instituto Leonidas e Maria Deane - Fiocruz Amazonia	Laboratorio de Ecologia de Doencas Transmissiveis na Amazonia, Instituto Leonidas e Maria Deane - Fiocruz Amazonia	Valdinete Nascimento et al
EPI_ISL_1068083	Laboratorio de Ecologia de Doencas Transmissiveis na Amazonia, Instituto Leonidas e Maria Deane - Fiocruz Amazonia	Laboratorio de Ecologia de Doencas Transmissiveis na Amazonia, Instituto Leonidas e Maria Deane - Fiocruz Amazonia	Valdinete Nascimento et al
EPI_ISL_1068084	Laboratorio de Ecologia de Doencas Transmissiveis na Amazonia, Instituto Leonidas e Maria Deane - Fiocruz Amazonia	Laboratorio de Ecologia de Doencas Transmissiveis na Amazonia, Instituto Leonidas e Maria Deane - Fiocruz Amazonia	Valdinete Nascimento et al
EPI_ISL_1068086	Laboratorio de Ecologia de Doencas Transmissiveis na Amazonia, Instituto Leonidas e Maria Deane - Fiocruz Amazonia	Laboratorio de Ecologia de Doencas Transmissiveis na Amazonia, Instituto Leonidas e Maria Deane - Fiocruz Amazonia	Valdinete Nascimento et al
EPI_ISL_1068089	Laboratorio de Ecologia de Doencas Transmissiveis na Amazonia, Instituto Leonidas e Maria Deane - Fiocruz Amazonia	Laboratorio de Ecologia de Doencas Transmissiveis na Amazonia, Instituto Leonidas e Maria Deane - Fiocruz Amazonia	Valdinete Nascimento et al
EPI_ISL_1068090	Laboratorio de Ecologia de Doencas Transmissiveis na Amazonia, Instituto Leonidas e Maria Deane - Fiocruz Amazonia	Laboratorio de Ecologia de Doencas Transmissiveis na Amazonia, Instituto Leonidas e Maria Deane - Fiocruz Amazonia	Valdinete Nascimento et al
EPI_ISL_1068091	Laboratorio de Ecologia de Doencas Transmissiveis na Amazonia, Instituto Leonidas e Maria Deane - Fiocruz Amazonia	Laboratorio de Ecologia de Doencas Transmissiveis na Amazonia, Instituto Leonidas e Maria Deane - Fiocruz Amazonia	Valdinete Nascimento et al
EPI_ISL_1068092	Laboratorio de Ecologia de Doencas Transmissiveis na Amazonia, Instituto Leonidas e Maria Deane - Fiocruz Amazonia	Laboratorio de Ecologia de Doencas Transmissiveis na Amazonia, Instituto Leonidas e Maria Deane - Fiocruz Amazonia	Valdinete Nascimento et al
EPI_ISL_1068095	Laboratorio de Ecologia de Doencas Transmissiveis na Amazonia, Instituto Leonidas e Maria Deane - Fiocruz Amazonia	Laboratorio de Ecologia de Doencas Transmissiveis na Amazonia, Instituto Leonidas e Maria Deane - Fiocruz Amazonia	Valdinete Nascimento et al
EPI_ISL_1068096	Laboratorio de Ecologia de Doencas Transmissiveis na Amazonia, Instituto Leonidas e Maria Deane - Fiocruz Amazonia	Laboratorio de Ecologia de Doencas Transmissiveis na Amazonia, Instituto Leonidas e Maria Deane - Fiocruz Amazonia	Valdinete Nascimento et al
EPI_ISL_1068097	Laboratorio de Ecologia de Doencas Transmissiveis na Amazonia, Instituto Leonidas e Maria Deane - Fiocruz Amazonia	Laboratorio de Ecologia de Doencas Transmissiveis na Amazonia, Instituto Leonidas e Maria Deane - Fiocruz Amazonia	Valdinete Nascimento et al
EPI_ISL_1068098	Laboratorio de Ecologia de Doencas Transmissiveis na Amazonia, Instituto Leonidas e Maria Deane - Fiocruz Amazonia	Laboratorio de Ecologia de Doencas Transmissiveis na Amazonia, Instituto Leonidas e Maria Deane - Fiocruz Amazonia	Valdinete Nascimento et al
EPI_ISL_801386	Laboratorio de Ecologia de Doencas Transmissiveis na Amazonia, Instituto Leonidas e Maria Deane - Fiocruz Amazonia	Laboratorio de Ecologia de Doencas Transmissiveis na Amazonia, Instituto Leonidas e Maria Deane - Fiocruz Amazonia	Valdinete Nascimento et al
EPI_ISL_801387	Laboratorio de Ecologia de Doencas Transmissiveis na Amazonia, Instituto Leonidas e Maria Deane - Fiocruz Amazonia	Laboratorio de Ecologia de Doencas Transmissiveis na Amazonia, Instituto Leonidas e Maria Deane - Fiocruz Amazonia	Valdinete Nascimento et al
EPI_ISL_801388	Laboratorio de Ecologia de Doencas Transmissiveis na Amazonia, Instituto Leonidas e Maria Deane - Fiocruz Amazonia	Laboratorio de Ecologia de Doencas Transmissiveis na Amazonia, Instituto Leonidas e Maria Deane - Fiocruz Amazonia	Valdinete Nascimento et al
EPI_ISL_1068099	Laboratorio de Ecologia de Doencas Transmissiveis na Amazonia, Instituto Leonidas e Maria Deane - Fiocruz Amazonia	Laboratorio de Ecologia de Doencas Transmissiveis na Amazonia, Instituto Leonidas e Maria Deane - Fiocruz Amazonia	Valdinete Nascimento et al
EPI_ISL_801389	Laboratorio de Ecologia de Doencas Transmissiveis na Amazonia, Instituto Leonidas e Maria Deane - Fiocruz Amazonia	Laboratorio de Ecologia de Doencas Transmissiveis na Amazonia, Instituto Leonidas e Maria Deane - Fiocruz Amazonia	Valdinete Nascimento et al

EPI_ISL_1068101	Laboratorio de Ecologia de Doencas Transmissiveis na Amazonia, Instituto Leonidas e Maria Deane - Fiocruz Amazonia	Laboratorio de Ecologia de Doencas Transmissiveis na Amazonia, Instituto Leonidas e Maria Deane - Fiocruz Amazonia	Valdinete Nascimento et al
EPI_ISL_1068102	Laboratorio de Ecologia de Doencas Transmissiveis na Amazonia, Instituto Leonidas e Maria Deane - Fiocruz Amazonia	Laboratorio de Ecologia de Doencas Transmissiveis na Amazonia, Instituto Leonidas e Maria Deane - Fiocruz Amazonia	Valdinete Nascimento et al
EPI_ISL_1068103	Laboratorio de Ecologia de Doencas Transmissiveis na Amazonia, Instituto Leonidas e Maria Deane - Fiocruz Amazonia	Laboratorio de Ecologia de Doencas Transmissiveis na Amazonia, Instituto Leonidas e Maria Deane - Fiocruz Amazonia	Valdinete Nascimento et al
EPI_ISL_1068104	Laboratorio de Ecologia de Doencas Transmissiveis na Amazonia, Instituto Leonidas e Maria Deane - Fiocruz Amazonia	Laboratorio de Ecologia de Doencas Transmissiveis na Amazonia, Instituto Leonidas e Maria Deane - Fiocruz Amazonia	Valdinete Nascimento et al
EPI_ISL_801390	Laboratorio de Ecologia de Doencas Transmissiveis na Amazonia, Instituto Leonidas e Maria Deane - Fiocruz Amazonia	Laboratorio de Ecologia de Doencas Transmissiveis na Amazonia, Instituto Leonidas e Maria Deane - Fiocruz Amazonia	Valdinete Nascimento et al
EPI_ISL_1068107	Laboratorio de Ecologia de Doencas Transmissiveis na Amazonia, Instituto Leonidas e Maria Deane - Fiocruz Amazonia	Laboratorio de Ecologia de Doencas Transmissiveis na Amazonia, Instituto Leonidas e Maria Deane - Fiocruz Amazonia	Valdinete Nascimento et al
EPI_ISL_801391	Laboratorio de Ecologia de Doencas Transmissiveis na Amazonia, Instituto Leonidas e Maria Deane - Fiocruz Amazonia	Laboratorio de Ecologia de Doencas Transmissiveis na Amazonia, Instituto Leonidas e Maria Deane - Fiocruz Amazonia	Valdinete Nascimento et al
EPI_ISL_801392	Laboratorio de Ecologia de Doencas Transmissiveis na Amazonia, Instituto Leonidas e Maria Deane - Fiocruz Amazonia	Laboratorio de Ecologia de Doencas Transmissiveis na Amazonia, Instituto Leonidas e Maria Deane - Fiocruz Amazonia	Valdinete Nascimento et al
EPI_ISL_833131	Laboratorio de Ecologia de Doencas Transmissiveis na Amazonia, Instituto Leonidas e Maria Deane - Fiocruz Amazonia	Laboratorio de Ecologia de Doencas Transmissiveis na Amazonia, Instituto Leonidas e Maria Deane - Fiocruz Amazonia	Valdinete Nascimento et al
EPI_ISL_1068116	Laboratorio de Ecologia de Doencas Transmissiveis na Amazonia, Instituto Leonidas e Maria Deane - Fiocruz Amazonia	Laboratorio de Ecologia de Doencas Transmissiveis na Amazonia, Instituto Leonidas e Maria Deane - Fiocruz Amazonia	Valdinete Nascimento et al
EPI_ISL_1068120	Laboratorio de Ecologia de Doencas Transmissiveis na Amazonia, Instituto Leonidas e Maria Deane - Fiocruz Amazonia	Laboratorio de Ecologia de Doencas Transmissiveis na Amazonia, Instituto Leonidas e Maria Deane - Fiocruz Amazonia	Valdinete Nascimento et al
EPI_ISL_1068122	Laboratorio de Ecologia de Doencas Transmissiveis na Amazonia, Instituto Leonidas e Maria Deane - Fiocruz Amazonia	Laboratorio de Ecologia de Doencas Transmissiveis na Amazonia, Instituto Leonidas e Maria Deane - Fiocruz Amazonia	Valdinete Nascimento et al
EPI_ISL_1068123	Laboratorio de Ecologia de Doencas Transmissiveis na Amazonia, Instituto Leonidas e Maria Deane - Fiocruz Amazonia	Laboratorio de Ecologia de Doencas Transmissiveis na Amazonia, Instituto Leonidas e Maria Deane - Fiocruz Amazonia	Valdinete Nascimento et al
EPI_ISL_1068124	Laboratorio de Ecologia de Doencas Transmissiveis na Amazonia, Instituto Leonidas e Maria Deane - Fiocruz Amazonia	Laboratorio de Ecologia de Doencas Transmissiveis na Amazonia, Instituto Leonidas e Maria Deane - Fiocruz Amazonia	Valdinete Nascimento et al
EPI_ISL_801393	Laboratorio de Ecologia de Doencas Transmissiveis na Amazonia, Instituto Leonidas e Maria Deane - Fiocruz Amazonia	Laboratorio de Ecologia de Doencas Transmissiveis na Amazonia, Instituto Leonidas e Maria Deane - Fiocruz Amazonia	Valdinete Nascimento et al
EPI_ISL_801394	Laboratorio de Ecologia de Doencas Transmissiveis na Amazonia, Instituto Leonidas e Maria Deane - Fiocruz Amazonia	Laboratorio de Ecologia de Doencas Transmissiveis na Amazonia, Instituto Leonidas e Maria Deane - Fiocruz Amazonia	Valdinete Nascimento et al
EPI_ISL_1068125	Laboratorio de Ecologia de Doencas Transmissiveis na Amazonia, Instituto Leonidas e Maria Deane - Fiocruz Amazonia	Laboratorio de Ecologia de Doencas Transmissiveis na Amazonia, Instituto Leonidas e Maria Deane - Fiocruz Amazonia	Valdinete Nascimento et al
EPI_ISL_801395	Laboratorio de Ecologia de Doencas Transmissiveis na Amazonia, Instituto Leonidas e Maria Deane - Fiocruz Amazonia	Laboratorio de Ecologia de Doencas Transmissiveis na Amazonia, Instituto Leonidas e Maria Deane - Fiocruz Amazonia	Valdinete Nascimento et al
EPI_ISL_1661251	Laboratorio de Ecologia de Doencas Transmissiveis na Amazonia, Instituto Leonidas e Maria Deane - Fiocruz Amazonia	Laboratorio de Ecologia de Doencas Transmissiveis na Amazonia, Instituto Leonidas e Maria Deane - Fiocruz Amazonia	Valdinete Nascimento et al
EPI_ISL_1068129	Laboratorio de Ecologia de Doencas Transmissiveis na Amazonia, Instituto Leonidas e Maria Deane - Fiocruz Amazonia	Laboratorio de Ecologia de Doencas Transmissiveis na Amazonia, Instituto Leonidas e Maria Deane - Fiocruz Amazonia	Valdinete Nascimento et al
EPI_ISL_1068130	Laboratorio de Ecologia de Doencas Transmissiveis na Amazonia, Instituto Leonidas e Maria Deane - Fiocruz Amazonia	Laboratorio de Ecologia de Doencas Transmissiveis na Amazonia, Instituto Leonidas e Maria Deane - Fiocruz Amazonia	Valdinete Nascimento et al
EPI_ISL_801396	Laboratorio de Ecologia de Doencas Transmissiveis na Amazonia, Instituto Leonidas e Maria Deane - Fiocruz Amazonia	Laboratorio de Ecologia de Doencas Transmissiveis na Amazonia, Instituto Leonidas e Maria Deane - Fiocruz Amazonia	Valdinete Nascimento et al

EPI_ISL_1068134	Laboratorio de Ecologia de Doencas Transmissiveis na Amazonia, Instituto Leonidas e Maria Deane - Fiocruz Amazonia	Laboratorio de Ecologia de Doencas Transmissiveis na Amazonia, Instituto Leonidas e Maria Deane - Fiocruz Amazonia	Valdinete Nascimento et al
EPI_ISL_1068137	Laboratorio de Ecologia de Doencas Transmissiveis na Amazonia, Instituto Leonidas e Maria Deane - Fiocruz Amazonia	Laboratorio de Ecologia de Doencas Transmissiveis na Amazonia, Instituto Leonidas e Maria Deane - Fiocruz Amazonia	Valdinete Nascimento et al
EPI_ISL_1068139	Laboratorio de Ecologia de Doencas Transmissiveis na Amazonia, Instituto Leonidas e Maria Deane - Fiocruz Amazonia	Laboratorio de Ecologia de Doencas Transmissiveis na Amazonia, Instituto Leonidas e Maria Deane - Fiocruz Amazonia	Valdinete Nascimento et al
EPI_ISL_1068142	Laboratorio de Ecologia de Doencas Transmissiveis na Amazonia, Instituto Leonidas e Maria Deane - Fiocruz Amazonia	Laboratorio de Ecologia de Doencas Transmissiveis na Amazonia, Instituto Leonidas e Maria Deane - Fiocruz Amazonia	Valdinete Nascimento et al
EPI_ISL_801397	Laboratório Central de Saúde Pública do Estado do Amazonas (LACEN-AM)	Laboratorio de Ecologia de Doencas Transmissiveis na Amazonia, Instituto Leonidas e Maria Deane - Fiocruz Amazonia	Valdinete Nascimento et al
EPI_ISL_801398	Laboratório Central de Saúde Pública do Estado do Amazonas (LACEN-AM)	Laboratorio de Ecologia de Doencas Transmissiveis na Amazonia, Instituto Leonidas e Maria Deane - Fiocruz Amazonia	Valdinete Nascimento et al
EPI_ISL_1068163	Laboratorio de Ecologia de Doencas Transmissiveis na Amazonia, Instituto Leonidas e Maria Deane - Fiocruz Amazonia	Laboratorio de Ecologia de Doencas Transmissiveis na Amazonia, Instituto Leonidas e Maria Deane - Fiocruz Amazonia	Valdinete Nascimento et al
EPI_ISL_801399	Laboratório Central de Saúde Pública do Estado do Amazonas (LACEN-AM)	Laboratorio de Ecologia de Doencas Transmissiveis na Amazonia, Instituto Leonidas e Maria Deane - Fiocruz Amazonia	Valdinete Nascimento et al
EPI_ISL_1068166	Laboratorio de Ecologia de Doencas Transmissiveis na Amazonia, Instituto Leonidas e Maria Deane - Fiocruz Amazonia	Laboratorio de Ecologia de Doencas Transmissiveis na Amazonia, Instituto Leonidas e Maria Deane - Fiocruz Amazonia	Valdinete Nascimento et al
EPI_ISL_1068167	Laboratorio de Ecologia de Doencas Transmissiveis na Amazonia, Instituto Leonidas e Maria Deane - Fiocruz Amazonia	Laboratorio de Ecologia de Doencas Transmissiveis na Amazonia, Instituto Leonidas e Maria Deane - Fiocruz Amazonia	Valdinete Nascimento et al
EPI_ISL_1068168	Laboratorio de Ecologia de Doencas Transmissiveis na Amazonia, Instituto Leonidas e Maria Deane - Fiocruz Amazonia	Laboratorio de Ecologia de Doencas Transmissiveis na Amazonia, Instituto Leonidas e Maria Deane - Fiocruz Amazonia	Valdinete Nascimento et al
EPI_ISL_1068171	Laboratorio de Ecologia de Doencas Transmissiveis na Amazonia, Instituto Leonidas e Maria Deane - Fiocruz Amazonia	Laboratorio de Ecologia de Doencas Transmissiveis na Amazonia, Instituto Leonidas e Maria Deane - Fiocruz Amazonia	Valdinete Nascimento et al
EPI_ISL_1068172	Laboratorio de Ecologia de Doencas Transmissiveis na Amazonia, Instituto Leonidas e Maria Deane - Fiocruz Amazonia	Laboratorio de Ecologia de Doencas Transmissiveis na Amazonia, Instituto Leonidas e Maria Deane - Fiocruz Amazonia	Valdinete Nascimento et al
EPI_ISL_1068173	Laboratorio de Ecologia de Doencas Transmissiveis na Amazonia, Instituto Leonidas e Maria Deane - Fiocruz Amazonia	Laboratorio de Ecologia de Doencas Transmissiveis na Amazonia, Instituto Leonidas e Maria Deane - Fiocruz Amazonia	Valdinete Nascimento et al
EPI_ISL_1068175	Laboratorio de Ecologia de Doencas Transmissiveis na Amazonia, Instituto Leonidas e Maria Deane - Fiocruz Amazonia	Laboratorio de Ecologia de Doencas Transmissiveis na Amazonia, Instituto Leonidas e Maria Deane - Fiocruz Amazonia	Valdinete Nascimento et al
EPI_ISL_801400	Laboratório Central de Saúde Pública do Estado do Amazonas (LACEN-AM)	Laboratorio de Ecologia de Doencas Transmissiveis na Amazonia, Instituto Leonidas e Maria Deane - Fiocruz Amazonia	Valdinete Nascimento et al
EPI_ISL_801401	Laboratório Central de Saúde Pública do Estado do Amazonas (LACEN-AM)	Laboratorio de Ecologia de Doencas Transmissiveis na Amazonia, Instituto Leonidas e Maria Deane - Fiocruz Amazonia	Valdinete Nascimento et al
EPI_ISL_1068180	Laboratorio de Ecologia de Doencas Transmissiveis na Amazonia, Instituto Leonidas e Maria Deane - Fiocruz Amazonia	Laboratorio de Ecologia de Doencas Transmissiveis na Amazonia, Instituto Leonidas e Maria Deane - Fiocruz Amazonia	Valdinete Nascimento et al
EPI_ISL_1068182	Laboratorio de Ecologia de Doencas Transmissiveis na Amazonia, Instituto Leonidas e Maria Deane - Fiocruz Amazonia	Laboratorio de Ecologia de Doencas Transmissiveis na Amazonia, Instituto Leonidas e Maria Deane - Fiocruz Amazonia	Valdinete Nascimento et al
EPI_ISL_1068186	Laboratorio de Ecologia de Doencas Transmissiveis na Amazonia, Instituto Leonidas e Maria Deane - Fiocruz Amazonia	Laboratorio de Ecologia de Doencas Transmissiveis na Amazonia, Instituto Leonidas e Maria Deane - Fiocruz Amazonia	Valdinete Nascimento et al
EPI_ISL_801402	Laboratório Central de Saúde Pública do Estado do Amazonas (LACEN-AM)	Laboratorio de Ecologia de Doencas Transmissiveis na Amazonia, Instituto Leonidas e Maria Deane - Fiocruz Amazonia	Valdinete Nascimento et al
EPI_ISL_1068189	Laboratorio de Ecologia de Doencas Transmissiveis na Amazonia, Instituto Leonidas e Maria Deane - Fiocruz Amazonia	Laboratorio de Ecologia de Doencas Transmissiveis na Amazonia, Instituto Leonidas e Maria Deane - Fiocruz Amazonia	Valdinete Nascimento et al

EPI_ISL_1068190	Laboratorio de Ecologia de Doencas Transmissiveis na Amazonia, Instituto Leonidas e Maria Deane - Fiocruz Amazonia	Laboratorio de Ecologia de Doencas Transmissiveis na Amazonia, Instituto Leonidas e Maria Deane - Fiocruz Amazonia	Valdinete Nascimento et al
EPI_ISL_1068192	Laboratorio de Ecologia de Doencas Transmissiveis na Amazonia, Instituto Leonidas e Maria Deane - Fiocruz Amazonia	Laboratorio de Ecologia de Doencas Transmissiveis na Amazonia, Instituto Leonidas e Maria Deane - Fiocruz Amazonia	Valdinete Nascimento et al
EPI_ISL_1068195	Laboratorio de Ecologia de Doencas Transmissiveis na Amazonia, Instituto Leonidas e Maria Deane - Fiocruz Amazonia	Laboratorio de Ecologia de Doencas Transmissiveis na Amazonia, Instituto Leonidas e Maria Deane - Fiocruz Amazonia	Valdinete Nascimento et al
EPI_ISL_1068197	Laboratorio de Ecologia de Doencas Transmissiveis na Amazonia, Instituto Leonidas e Maria Deane - Fiocruz Amazonia	Laboratorio de Ecologia de Doencas Transmissiveis na Amazonia, Instituto Leonidas e Maria Deane - Fiocruz Amazonia	Valdinete Nascimento et al
EPI_ISL_1068200	Laboratorio de Ecologia de Doencas Transmissiveis na Amazonia, Instituto Leonidas e Maria Deane - Fiocruz Amazonia	Laboratorio de Ecologia de Doencas Transmissiveis na Amazonia, Instituto Leonidas e Maria Deane - Fiocruz Amazonia	Valdinete Nascimento et al
EPI_ISL_1068201	Laboratorio de Ecologia de Doencas Transmissiveis na Amazonia, Instituto Leonidas e Maria Deane - Fiocruz Amazonia	Laboratorio de Ecologia de Doencas Transmissiveis na Amazonia, Instituto Leonidas e Maria Deane - Fiocruz Amazonia	Valdinete Nascimento et al
EPI_ISL_1068202	Laboratorio de Ecologia de Doencas Transmissiveis na Amazonia, Instituto Leonidas e Maria Deane - Fiocruz Amazonia	Laboratorio de Ecologia de Doencas Transmissiveis na Amazonia, Instituto Leonidas e Maria Deane - Fiocruz Amazonia	Valdinete Nascimento et al
EPI_ISL_1068204	Laboratorio de Ecologia de Doencas Transmissiveis na Amazonia, Instituto Leonidas e Maria Deane - Fiocruz Amazonia	Laboratorio de Ecologia de Doencas Transmissiveis na Amazonia, Instituto Leonidas e Maria Deane - Fiocruz Amazonia	Valdinete Nascimento et al
EPI_ISL_1068205	Laboratorio de Ecologia de Doencas Transmissiveis na Amazonia, Instituto Leonidas e Maria Deane - Fiocruz Amazonia	Laboratorio de Ecologia de Doencas Transmissiveis na Amazonia, Instituto Leonidas e Maria Deane - Fiocruz Amazonia	Valdinete Nascimento et al
EPI_ISL_1068206	Laboratorio de Ecologia de Doencas Transmissiveis na Amazonia, Instituto Leonidas e Maria Deane - Fiocruz Amazonia	Laboratorio de Ecologia de Doencas Transmissiveis na Amazonia, Instituto Leonidas e Maria Deane - Fiocruz Amazonia	Valdinete Nascimento et al
EPI_ISL_1068207	Laboratorio de Ecologia de Doencas Transmissiveis na Amazonia, Instituto Leonidas e Maria Deane - Fiocruz Amazonia	Laboratorio de Ecologia de Doencas Transmissiveis na Amazonia, Instituto Leonidas e Maria Deane - Fiocruz Amazonia	Valdinete Nascimento et al
EPI_ISL_1068208	Laboratorio de Ecologia de Doencas Transmissiveis na Amazonia, Instituto Leonidas e Maria Deane - Fiocruz Amazonia	Laboratorio de Ecologia de Doencas Transmissiveis na Amazonia, Instituto Leonidas e Maria Deane - Fiocruz Amazonia	Valdinete Nascimento et al
EPI_ISL_1068209	Laboratorio de Ecologia de Doencas Transmissiveis na Amazonia, Instituto Leonidas e Maria Deane - Fiocruz Amazonia	Laboratorio de Ecologia de Doencas Transmissiveis na Amazonia, Instituto Leonidas e Maria Deane - Fiocruz Amazonia	Valdinete Nascimento et al
EPI_ISL_1068210	Laboratorio de Ecologia de Doencas Transmissiveis na Amazonia, Instituto Leonidas e Maria Deane - Fiocruz Amazonia	Laboratorio de Ecologia de Doencas Transmissiveis na Amazonia, Instituto Leonidas e Maria Deane - Fiocruz Amazonia	Valdinete Nascimento et al
EPI_ISL_1068211	Laboratorio de Ecologia de Doencas Transmissiveis na Amazonia, Instituto Leonidas e Maria Deane - Fiocruz Amazonia	Laboratorio de Ecologia de Doencas Transmissiveis na Amazonia, Instituto Leonidas e Maria Deane - Fiocruz Amazonia	Valdinete Nascimento et al
EPI_ISL_1068213	Laboratorio de Ecologia de Doencas Transmissiveis na Amazonia, Instituto Leonidas e Maria Deane - Fiocruz Amazonia	Laboratorio de Ecologia de Doencas Transmissiveis na Amazonia, Instituto Leonidas e Maria Deane - Fiocruz Amazonia	Valdinete Nascimento et al
EPI_ISL_1068214	Laboratorio de Ecologia de Doencas Transmissiveis na Amazonia, Instituto Leonidas e Maria Deane - Fiocruz Amazonia	Laboratorio de Ecologia de Doencas Transmissiveis na Amazonia, Instituto Leonidas e Maria Deane - Fiocruz Amazonia	Valdinete Nascimento et al
EPI_ISL_1068216	Laboratorio de Ecologia de Doencas Transmissiveis na Amazonia, Instituto Leonidas e Maria Deane - Fiocruz Amazonia	Laboratorio de Ecologia de Doencas Transmissiveis na Amazonia, Instituto Leonidas e Maria Deane - Fiocruz Amazonia	Valdinete Nascimento et al
EPI_ISL_1068217	Laboratorio de Ecologia de Doencas Transmissiveis na Amazonia, Instituto Leonidas e Maria Deane - Fiocruz Amazonia	Laboratorio de Ecologia de Doencas Transmissiveis na Amazonia, Instituto Leonidas e Maria Deane - Fiocruz Amazonia	Valdinete Nascimento et al
EPI_ISL_1068218	Laboratorio de Ecologia de Doencas Transmissiveis na Amazonia, Instituto Leonidas e Maria Deane - Fiocruz Amazonia	Laboratorio de Ecologia de Doencas Transmissiveis na Amazonia, Instituto Leonidas e Maria Deane - Fiocruz Amazonia	Valdinete Nascimento et al
EPI_ISL_1068220	Laboratorio de Ecologia de Doencas Transmissiveis na Amazonia, Instituto Leonidas e Maria Deane - Fiocruz Amazonia	Laboratorio de Ecologia de Doencas Transmissiveis na Amazonia, Instituto Leonidas e Maria Deane - Fiocruz Amazonia	Valdinete Nascimento et al
EPI_ISL_1068224	Laboratorio de Ecologia de Doencas Transmissiveis na Amazonia, Instituto Leonidas e Maria Deane - Fiocruz Amazonia	Laboratorio de Ecologia de Doencas Transmissiveis na Amazonia, Instituto Leonidas e Maria Deane - Fiocruz Amazonia	Valdinete Nascimento et al

EPI_ISL_1068227	Laboratorio de Ecologia de Doencas Transmissiveis na Amazonia, Instituto Leonidas e Maria Deane - Fiocruz Amazonia	Laboratorio de Ecologia de Doencas Transmissiveis na Amazonia, Instituto Leonidas e Maria Deane - Fiocruz Amazonia	Valdinete Nascimento et al
EPI_ISL_1068228	Laboratorio de Ecologia de Doencas Transmissiveis na Amazonia, Instituto Leonidas e Maria Deane - Fiocruz Amazonia	Laboratorio de Ecologia de Doencas Transmissiveis na Amazonia, Instituto Leonidas e Maria Deane - Fiocruz Amazonia	Valdinete Nascimento et al
EPI_ISL_1068229	Laboratorio de Ecologia de Doencas Transmissiveis na Amazonia, Instituto Leonidas e Maria Deane - Fiocruz Amazonia	Laboratorio de Ecologia de Doencas Transmissiveis na Amazonia, Instituto Leonidas e Maria Deane - Fiocruz Amazonia	Valdinete Nascimento et al
EPI_ISL_1068230	Laboratorio de Ecologia de Doencas Transmissiveis na Amazonia, Instituto Leonidas e Maria Deane - Fiocruz Amazonia	Laboratorio de Ecologia de Doencas Transmissiveis na Amazonia, Instituto Leonidas e Maria Deane - Fiocruz Amazonia	Valdinete Nascimento et al
EPI_ISL_1068231	Laboratorio de Ecologia de Doencas Transmissiveis na Amazonia, Instituto Leonidas e Maria Deane - Fiocruz Amazonia	Laboratorio de Ecologia de Doencas Transmissiveis na Amazonia, Instituto Leonidas e Maria Deane - Fiocruz Amazonia	Valdinete Nascimento et al
EPI_ISL_1068232	Laboratorio de Ecologia de Doencas Transmissiveis na Amazonia, Instituto Leonidas e Maria Deane - Fiocruz Amazonia	Laboratorio de Ecologia de Doencas Transmissiveis na Amazonia, Instituto Leonidas e Maria Deane - Fiocruz Amazonia	Valdinete Nascimento et al
EPI_ISL_1068233	Laboratorio de Ecologia de Doencas Transmissiveis na Amazonia, Instituto Leonidas e Maria Deane - Fiocruz Amazonia	Laboratorio de Ecologia de Doencas Transmissiveis na Amazonia, Instituto Leonidas e Maria Deane - Fiocruz Amazonia	Valdinete Nascimento et al
EPI_ISL_1068234	Laboratorio de Ecologia de Doencas Transmissiveis na Amazonia, Instituto Leonidas e Maria Deane - Fiocruz Amazonia	Laboratorio de Ecologia de Doencas Transmissiveis na Amazonia, Instituto Leonidas e Maria Deane - Fiocruz Amazonia	Valdinete Nascimento et al
EPI_ISL_801403	Laboratório Central de Saúde Pública do Estado do Amazonas (LACEN-AM)	Laboratorio de Ecologia de Doencas Transmissiveis na Amazonia, Instituto Leonidas e Maria Deane - Fiocruz Amazonia	Valdinete Nascimento et al
EPI_ISL_1068236	Laboratorio de Ecologia de Doencas Transmissiveis na Amazonia, Instituto Leonidas e Maria Deane - Fiocruz Amazonia	Laboratorio de Ecologia de Doencas Transmissiveis na Amazonia, Instituto Leonidas e Maria Deane - Fiocruz Amazonia	Valdinete Nascimento et al
EPI_ISL_1068240	Laboratorio de Ecologia de Doencas Transmissiveis na Amazonia, Instituto Leonidas e Maria Deane - Fiocruz Amazonia	Laboratorio de Ecologia de Doencas Transmissiveis na Amazonia, Instituto Leonidas e Maria Deane - Fiocruz Amazonia	Valdinete Nascimento et al
EPI_ISL_1068241	Laboratorio de Ecologia de Doencas Transmissiveis na Amazonia, Instituto Leonidas e Maria Deane - Fiocruz Amazonia	Laboratorio de Ecologia de Doencas Transmissiveis na Amazonia, Instituto Leonidas e Maria Deane - Fiocruz Amazonia	Valdinete Nascimento et al
EPI_ISL_1068242	Laboratorio de Ecologia de Doencas Transmissiveis na Amazonia, Instituto Leonidas e Maria Deane - Fiocruz Amazonia	Laboratorio de Ecologia de Doencas Transmissiveis na Amazonia, Instituto Leonidas e Maria Deane - Fiocruz Amazonia	Valdinete Nascimento et al
EPI_ISL_1068244	Laboratorio de Ecologia de Doencas Transmissiveis na Amazonia, Instituto Leonidas e Maria Deane - Fiocruz Amazonia	Laboratorio de Ecologia de Doencas Transmissiveis na Amazonia, Instituto Leonidas e Maria Deane - Fiocruz Amazonia	Valdinete Nascimento et al
EPI_ISL_1068245	Laboratorio de Ecologia de Doencas Transmissiveis na Amazonia, Instituto Leonidas e Maria Deane - Fiocruz Amazonia	Laboratorio de Ecologia de Doencas Transmissiveis na Amazonia, Instituto Leonidas e Maria Deane - Fiocruz Amazonia	Valdinete Nascimento et al
EPI_ISL_1068246	Laboratorio de Ecologia de Doencas Transmissiveis na Amazonia, Instituto Leonidas e Maria Deane - Fiocruz Amazonia	Laboratorio de Ecologia de Doencas Transmissiveis na Amazonia, Instituto Leonidas e Maria Deane - Fiocruz Amazonia	Valdinete Nascimento et al
EPI_ISL_1068247	Laboratorio de Ecologia de Doencas Transmissiveis na Amazonia, Instituto Leonidas e Maria Deane - Fiocruz Amazonia	Laboratorio de Ecologia de Doencas Transmissiveis na Amazonia, Instituto Leonidas e Maria Deane - Fiocruz Amazonia	Valdinete Nascimento et al
EPI_ISL_1068250	Laboratorio de Ecologia de Doencas Transmissiveis na Amazonia, Instituto Leonidas e Maria Deane - Fiocruz Amazonia	Laboratorio de Ecologia de Doencas Transmissiveis na Amazonia, Instituto Leonidas e Maria Deane - Fiocruz Amazonia	Valdinete Nascimento et al
EPI_ISL_1068252	Laboratorio de Ecologia de Doencas Transmissiveis na Amazonia, Instituto Leonidas e Maria Deane - Fiocruz Amazonia	Laboratorio de Ecologia de Doencas Transmissiveis na Amazonia, Instituto Leonidas e Maria Deane - Fiocruz Amazonia	Valdinete Nascimento et al
EPI_ISL_1068253	Laboratorio de Ecologia de Doencas Transmissiveis na Amazonia, Instituto Leonidas e Maria Deane - Fiocruz Amazonia	Laboratorio de Ecologia de Doencas Transmissiveis na Amazonia, Instituto Leonidas e Maria Deane - Fiocruz Amazonia	Valdinete Nascimento et al
EPI_ISL_1068254	Laboratorio de Ecologia de Doencas Transmissiveis na Amazonia, Instituto Leonidas e Maria Deane - Fiocruz Amazonia	Laboratorio de Ecologia de Doencas Transmissiveis na Amazonia, Instituto Leonidas e Maria Deane - Fiocruz Amazonia	Valdinete Nascimento et al
EPI_ISL_1533609	Laboratorio de Ecologia de Doencas Transmissiveis na Amazonia, Instituto Leonidas e Maria Deane - Fiocruz Amazonia	Laboratorio de Ecologia de Doencas Transmissiveis na Amazonia, Instituto Leonidas e Maria Deane - Fiocruz Amazonia	Valdinete Nascimento et al

EPI_ISL_1068258	Laboratorio de Ecologia de Doencas Transmissiveis na Amazonia, Instituto Leonidas e Maria Deane - Fiocruz Amazonia	Laboratorio de Ecologia de Doencas Transmissiveis na Amazonia, Instituto Leonidas e Maria Deane - Fiocruz Amazonia	Valdinete Nascimento et al
EPI_ISL_1015159	Department of Virology, Pitié-Salpêtrière hospital	Department of Virology, Pitié-Salpêtrière hospital	Valentin Leducq et al
EPI_ISL_1395782	Instituto de Investigaciones Biomédicas en Retrovirus y SIDA (INBIRS)	Área de Secuenciación del Laboratorio de Virología del Hospital de Niños Dr. Ricardo Gutierrez on behalf of 'Proyecto Argentino Interinstitucional de genomica de SARS-CoV-2' (PAIS Consortium)	Vanesa Seery et al
EPI_ISL_1395783	Instituto de Investigaciones Biomédicas en Retrovirus y SIDA (INBIRS)	Área de Secuenciación del Laboratorio de Virología del Hospital de Niños Dr. Ricardo Gutierrez on behalf of 'Proyecto Argentino Interinstitucional de genomica de SARS-CoV-2' (PAIS Consortium)	Vanesa Seery et al
EPI_ISL_1395784	Instituto de Investigaciones Biomédicas en Retrovirus y SIDA (INBIRS)	Área de Secuenciación del Laboratorio de Virología del Hospital de Niños Dr. Ricardo Gutierrez on behalf of 'Proyecto Argentino Interinstitucional de genomica de SARS-CoV-2' (PAIS Consortium)	Vanesa Seery et al
EPI_ISL_1395802	Instituto de Investigaciones Biomédicas en Retrovirus y SIDA (INBIRS)	Área de Secuenciación del Laboratorio de Virología del Hospital de Niños Dr. Ricardo Gutierrez on behalf of 'Proyecto Argentino Interinstitucional de genomica de SARS-CoV-2' (PAIS Consortium)	Vanesa Seery et al
EPI_ISL_1251212	Raimundo Reginaldo de Almeida	MOA Lab	Vanessa C. Nicolete et al
EPI_ISL_1251214	Raimundo Reginaldo de Almeida	MOA Lab	Vanessa C. Nicolete et al
EPI_ISL_1251217	Raimundo Reginaldo de Almeida	MOA Lab	Vanessa C. Nicolete et al
EPI_ISL_1251224	Raimundo Reginaldo de Almeida	MOA Lab	Vanessa C. Nicolete et al
EPI_ISL_1251222	Raimundo Reginaldo de Almeida	MOA Lab	Vanessa C. Nicolete et al
EPI_ISL_1251225	Raimundo Reginaldo de Almeida	MOA Lab	Vanessa C. Nicolete et al
EPI_ISL_1251213	Raimundo Reginaldo de Almeida	MOA Lab	Vanessa C. Nicolete et al
EPI_ISL_1251216	Raimundo Reginaldo de Almeida	MOA Lab	Vanessa C. Nicolete et al
EPI_ISL_1251223	Raimundo Reginaldo de Almeida	MOA Lab	Vanessa C. Nicolete et al
EPI_ISL_430469	Hellenic Pasteur Institute, Public Health Laboratories	Hellenic Pasteur Institute, Public Health Laboratories, Unit of Bioinformatics and Applied Genomics	Vasiliki Pogka et al
EPI_ISL_501248	Hellenic Pasteur Institute, National Influenza Reference laboratory of Southern Greece & Unit of Bioinformatics and Applied Genomics	Hellenic Pasteur Institute, National Influenza Reference laboratory of Southern Greece & Unit of Bioinformatics and Applied Genomics	Vasiliki Pogka et al
EPI_ISL_501249	Hellenic Pasteur Institute, National Influenza Reference laboratory of Southern Greece & Unit of Bioinformatics and Applied Genomics	Hellenic Pasteur Institute, National Influenza Reference laboratory of Southern Greece & Unit of Bioinformatics and Applied Genomics	Vasiliki Pogka et al
EPI_ISL_501230	Hellenic Pasteur Institute, Public Health Laboratories	Hellenic Pasteur Institute, National Influenza Reference laboratory of Southern Greece & Unit of Bioinformatics and Applied Genomics	Vasiliki Pogka et al
EPI_ISL_501233	Hellenic Pasteur Institute, National Influenza Reference laboratory of Southern Greece & Unit of Bioinformatics and Applied Genomics	Hellenic Pasteur Institute, National Influenza Reference laboratory of Southern Greece & Unit of Bioinformatics and Applied Genomics	Vasiliki Pogka et al
EPI_ISL_501250	Hellenic Pasteur Institute, National Influenza Reference laboratory of Southern Greece & Unit of Bioinformatics and Applied Genomics	Hellenic Pasteur Institute, National Influenza Reference laboratory of Southern Greece & Unit of Bioinformatics and Applied Genomics	Vasiliki Pogka et al
EPI_ISL_501242	Hellenic Pasteur Institute, National Influenza Reference laboratory of Southern Greece & Unit of Bioinformatics and Applied Genomics	Hellenic Pasteur Institute, National Influenza Reference laboratory of Southern Greece & Unit of Bioinformatics and Applied Genomics	Vasiliki Pogka et al
EPI_ISL_501251	Hellenic Pasteur Institute, National Influenza Reference laboratory of Southern Greece & Unit of Bioinformatics and Applied Genomics	Hellenic Pasteur Institute, National Influenza Reference laboratory of Southern Greece & Unit of Bioinformatics and Applied Genomics	Vasiliki Pogka et al

EPI_ISL_451311	Hellenic Pasteur Institute, National Influenza Reference laboratory of Southern Greece & Unit of Bioinformatics and Applied Genomics	Hellenic Pasteur Institute, National Influenza Reference laboratory of Southern Greece & Unit of Bioinformatics and Applied Genomics	Vasiliki Pogka et al
EPI_ISL_451310	Hellenic Pasteur Institute, National Influenza Reference laboratory of Southern Greece & Unit of Bioinformatics and Applied Genomics	Hellenic Pasteur Institute, National Influenza Reference laboratory of Southern Greece & Unit of Bioinformatics and Applied Genomics	Vasiliki Pogka et al
EPI_ISL_1360411	Laboratorio de Enfermedades Emergentes y Reemergentes	Laboratorio de Enfermedades Emergentes y Reemergentes	Vega-Magaña Natali et al
EPI_ISL_1278371	Thai Red Cross Emerging Infectious Diseases Health Science Centre, Chulalongkorn Hospital, Faculty of Medicine, Chulalongkorn University	Thai Red Cross Emerging Infectious Diseases Center and Faculty of Medicine, Chulalongkorn University	Vichan Pawun et al
EPI_ISL_861457	WHO/Minsk	Charité Universitätsmedizin Berlin, Institut für Virologie	Victor M Corman et al
EPI_ISL_860805	WHO/Minsk	Charité Universitätsmedizin Berlin, Institut für Virologie	Victor M Corman et al
EPI_ISL_860811	WHO/Minsk	Charité Universitätsmedizin Berlin, Institut für Virologie	Victor M Corman et al
EPI_ISL_462450	Clinical Center, University of Sarajevo	Charite Universitatsmedizin Berlin, Institute of Virology	Victor M Corman et al
EPI_ISL_462462	Clinical Center, University of Sarajevo	Charite Universitatsmedizin Berlin, Institute of Virology	Victor M Corman et al
EPI_ISL_462463	Clinical Center, University of Sarajevo	Charite Universitatsmedizin Berlin, Institute of Virology	Victor M Corman et al
EPI_ISL_462467	Clinical Center, University of Sarajevo	Charite Universitatsmedizin Berlin, Institute of Virology	Victor M Corman et al
EPI_ISL_462476	Clinical Center, University of Sarajevo	Charite Universitatsmedizin Berlin, Institute of Virology	Victor M Corman et al
EPI_ISL_462472	Clinical Center, University of Sarajevo	Charite Universitatsmedizin Berlin, Institute of Virology	Victor M Corman et al
EPI_ISL_462461	Clinical Center, University of Sarajevo	Charite Universitatsmedizin Berlin, Institute of Virology	Victor M Corman et al
EPI_ISL_462466	Clinical Center, University of Sarajevo	Charite Universitatsmedizin Berlin, Institute of Virology	Victor M Corman et al
EPI_ISL_462470	Clinical Center, University of Sarajevo	Charite Universitatsmedizin Berlin, Institute of Virology	Victor M Corman et al
EPI_ISL_416742	NRL for Influenza, Centrum Epidemiology and Microbiology of National Institute of Public Health, Czech Republic	Charite Universitaetsmedizin Berlin, Institute of Virology	Victor M Corman et al
EPI_ISL_753746	Charité Universitätsmedizin Berlin, Institut für Virologie/Labor Berlin	Charité Universitätsmedizin Berlin, Institut für Virologie	Victor M Corman et al
EPI_ISL_753706	Charité Universitätsmedizin Berlin, Institut für Virologie/Labor Berlin	Charité Universitätsmedizin Berlin, Institut für Virologie	Victor M Corman et al
EPI_ISL_753726	Charité Universitätsmedizin Berlin, Institut für Virologie/Labor Berlin	Charité Universitätsmedizin Berlin, Institut für Virologie	Victor M Corman et al
EPI_ISL_753982	Charité Universitätsmedizin Berlin, Institut für Virologie/Labor Berlin	Charité Universitätsmedizin Berlin, Institut für Virologie	Victor M Corman et al
EPI_ISL_753986	Charité Universitätsmedizin Berlin, Institut für Virologie/Labor Berlin	Charité Universitätsmedizin Berlin, Institut für Virologie	Victor M Corman et al
EPI_ISL_753708	Charité Universitätsmedizin Berlin, Institut für Virologie/Labor Berlin	Charité Universitätsmedizin Berlin, Institut für Virologie	Victor M Corman et al
EPI_ISL_729473	Charité Universitätsmedizin Berlin, Institut für Virologie/Labor Berlin	Charité Universitätsmedizin Berlin, Institut für Virologie	Victor M Corman et al
EPI_ISL_406862	Charité Universitätsmedizin Berlin, Institute of Virology; Institut für Mikrobiologie der Bundeswehr, Munich	Charité Universitätsmedizin Berlin, Institute of Virology	Victor M Corman et al
EPI_ISL_729573	A. Krumbholz, Labor Dr. Krause und Kollegen MVZ GmbH, Kiel	Charité Universitätsmedizin Berlin, Institut für Virologie	Victor M Corman et al
EPI_ISL_729589	A. Krumbholz, Labor Dr. Krause und Kollegen MVZ GmbH, Kiel	Charité Universitätsmedizin Berlin, Institut für Virologie	Victor M Corman et al
EPI_ISL_729660	A. Krumbholz, Labor Dr. Krause und Kollegen MVZ GmbH, Kiel	Charité Universitätsmedizin Berlin, Institut für Virologie	Victor M Corman et al

EPI_ISL_1027653	Department of Microbiology, National Institute for Public Health of Kosova	Charité Universitätsmedizin Berlin, Institut für Virologie	Victor M Corman et al
EPI_ISL_1027654	Department of Microbiology, National Institute for Public Health of Kosova	Charité Universitätsmedizin Berlin, Institut für Virologie	Victor M Corman et al
EPI_ISL_1027652	Department of Microbiology, National Institute for Public Health of Kosova	Charité Universitätsmedizin Berlin, Institut für Virologie	Victor M Corman et al
EPI_ISL_1027638	Department of Microbiology, National Institute for Public Health of Kosova	Charité Universitätsmedizin Berlin, Institut für Virologie	Victor M Corman et al
EPI_ISL_1027640	Department of Microbiology, National Institute for Public Health of Kosova	Charité Universitätsmedizin Berlin, Institut für Virologie	Victor M Corman et al
EPI_ISL_1027645	Department of Microbiology, National Institute for Public Health of Kosova	Charité Universitätsmedizin Berlin, Institut für Virologie	Victor M Corman et al
EPI_ISL_1027646	Department of Microbiology, National Institute for Public Health of Kosova	Charité Universitätsmedizin Berlin, Institut für Virologie	Victor M Corman et al
EPI_ISL_1027648	Department of Microbiology, National Institute for Public Health of Kosova	Charité Universitätsmedizin Berlin, Institut für Virologie	Victor M Corman et al
EPI_ISL_1027650	Department of Microbiology, National Institute for Public Health of Kosova	Charité Universitätsmedizin Berlin, Institut für Virologie	Victor M Corman et al
EPI_ISL_1027651	Department of Microbiology, National Institute for Public Health of Kosova	Charité Universitätsmedizin Berlin, Institut für Virologie	Victor M Corman et al
EPI_ISL_523960	National Agency for Public Health, Republic of Moldova	Charite Universitatsmedizin Berlin, Institute of Virology	Victor M Corman et al
EPI_ISL_523962	National Agency for Public Health, Republic of Moldova	Charite Universitatsmedizin Berlin, Institute of Virology	Victor M Corman et al
EPI_ISL_523964	National Agency for Public Health, Republic of Moldova	Charite Universitatsmedizin Berlin, Institute of Virology	Victor M Corman et al
EPI_ISL_523968	National Agency for Public Health, Republic of Moldova	Charite Universitatsmedizin Berlin, Institute of Virology	Victor M Corman et al
EPI_ISL_523966	National Agency for Public Health, Republic of Moldova	Charite Universitatsmedizin Berlin, Institute of Virology	Victor M Corman et al
EPI_ISL_516922	Department for Molecular Diagnostics, Centre for Medical Microbiology, Institute of Public Health of Montenegro	Charité Universitätsmedizin Berlin, Institut für Virologie	Victor M Corman et al
EPI_ISL_516927	Department for Molecular Diagnostics, Centre for Medical Microbiology, Institute of Public Health of Montenegro	Charité Universitätsmedizin Berlin, Institut für Virologie	Victor M Corman et al
EPI_ISL_516928	Department for Molecular Diagnostics, Centre for Medical Microbiology, Institute of Public Health of Montenegro	Charité Universitätsmedizin Berlin, Institut für Virologie	Victor M Corman et al
EPI_ISL_516929	Department for Molecular Diagnostics, Centre for Medical Microbiology, Institute of Public Health of Montenegro	Charité Universitätsmedizin Berlin, Institut für Virologie	Victor M Corman et al
EPI_ISL_516930	Department for Molecular Diagnostics, Centre for Medical Microbiology, Institute of Public Health of Montenegro	Charité Universitätsmedizin Berlin, Institut für Virologie	Victor M Corman et al
EPI_ISL_516931	Department for Molecular Diagnostics, Centre for Medical Microbiology, Institute of Public Health of Montenegro	Charité Universitätsmedizin Berlin, Institut für Virologie	Victor M Corman et al
EPI_ISL_516932	Department for Molecular Diagnostics, Centre for Medical Microbiology, Institute of Public Health of Montenegro	Charité Universitätsmedizin Berlin, Institut für Virologie	Victor M Corman et al
EPI_ISL_1013598	Department for Molecular Diagnostics, Centre for Medical Microbiology, Institute of Public Health, Montenegro	Charité Universitätsmedizin Berlin, Institut für Virologie	Victor M Corman et al
EPI_ISL_1013601	Department for Molecular Diagnostics, Centre for Medical Microbiology, Institute of Public Health, Montenegro	Charité Universitätsmedizin Berlin, Institut für Virologie	Victor M Corman et al
EPI_ISL_1013614	Department for Molecular Diagnostics, Centre for Medical Microbiology, Institute of Public Health, Montenegro	Charité Universitätsmedizin Berlin, Institut für Virologie	Victor M Corman et al
EPI_ISL_492076	Institute for Public Health of the Republic of North Macedonia	Charite Universitatsmedizin Berlin, Institute of Virology	Victor M Corman et al
EPI_ISL_541658	Laboratory Diagnostic, Veterinary Specialized Institute Kraljevo	Laboratory Diagnostic, Veterinary Specialized Institute Kraljevo	Vidanovic et al

EPI_ISL_582530	Laboratory Diagnostic, Veterinary Specialized Institute Kraljevo	Laboratory Diagnostic, Veterinary Specialized Institute Kraljevo	Vidanovic et al
EPI_ISL_644567	Laboratory Diagnostic, Veterinary Specialized Institute Kraljevo	Laboratory Diagnostic, Veterinary Specialized Institute Kraljevo	Vidanovic et al
EPI_ISL_541655	Laboratory Diagnostic, Veterinary Specialized Institute Kraljevo	Laboratory Diagnostic, Veterinary Specialized Institute Kraljevo	Vidanovic et al
EPI_ISL_541657	Laboratory Diagnostic, Veterinary Specialized Institute Kraljevo	Laboratory Diagnostic, Veterinary Specialized Institute Kraljevo	Vidanovic et al
EPI_ISL_904005	Scientific Veterinary Institute Novi Sad	Veterinary Specialized Institute Kraljevo, Serbia	Vidanovic et al
EPI_ISL_582525	Veterinary Specialized Institute Kraljevo, Serbia	Veterinary Specialized Institute Kraljevo, Serbia	Vidanovic et al
EPI_ISL_904007	Veterinary Specialized Institute Kraljevo	Veterinary Specialized Institute Kraljevo, Serbia	Vidanovic et al
EPI_ISL_462435	unknown	Laboratory Diagnostic	Vidanovic et al
EPI_ISL_514426	Laboratory Diagnostic, Veterinary Specialized Institute Kraljevo	Laboratory Diagnostic, Veterinary Specialized Institute Kraljevo	Vidanovic et al
EPI_ISL_514428	Laboratory Diagnostic, Veterinary Specialized Institute Kraljevo	Laboratory Diagnostic, Veterinary Specialized Institute Kraljevo	Vidanovic et al
EPI_ISL_462438	unknown	Laboratory Diagnostic	Vidanovic et al
EPI_ISL_661189	Scientific Veterinary Institute Novi Sad	Veterinary Specialized Institute Kraljevo, Serbia	Vidanovic et al
EPI_ISL_644572	Veterinary Specialized Institute Kraljevo, Serbia	Veterinary Specialized Institute Kraljevo, Serbia	Vidanovic et al
EPI_ISL_582532	Veterinary Specialized Institute Kraljevo, Serbia	Veterinary Specialized Institute Kraljevo, Serbia	Vidanovic et al
EPI_ISL_904010	Veterinary Specialized Institute Kraljevo	Veterinary Specialized Institute Kraljevo, Serbia	Vidanovic et al
EPI_ISL_541652	Laboratory Diagnostic, Veterinary Specialized Institute Kraljevo	Laboratory Diagnostic, Veterinary Specialized Institute Kraljevo	Vidanovic et al
EPI_ISL_541654	Laboratory Diagnostic, Veterinary Specialized Institute Kraljevo	Laboratory Diagnostic, Veterinary Specialized Institute Kraljevo	Vidanovic et al
EPI_ISL_541661	Laboratory Diagnostic, Veterinary Specialized Institute Kraljevo	Laboratory Diagnostic, Veterinary Specialized Institute Kraljevo	Vidanovic et al
EPI_ISL_833517	Veterinary Specialized Instute Nis	Veterinary Specialized Institute Kraljevo, Serbia	Vidanovic et al
EPI_ISL_661185	Scientific Veterinary Institute Novi Sad	Veterinary Specialized Institute Kraljevo, Serbia	Vidanovic et al
EPI_ISL_676583	Scientific Veterinary Institute Novi Sad	Veterinary Specialized Institute Kraljevo, Serbia	Vidanovic et al
EPI_ISL_661192	Scientific Veterinary Institute Novi Sad	Veterinary Specialized Institute Kraljevo, Serbia	Vidanovic et al
EPI_ISL_904009	Scientific Veterinary Institute Novi Sad	Veterinary Specialized Institute Kraljevo, Serbia	Vidanovic et al
EPI_ISL_676576	Scientific Veterinary Institute Novi Sad	Veterinary Specialized Institute Kraljevo, Serbia	Vidanovic et al
EPI_ISL_676594	Scientific Veterinary Institute Novi Sad	Veterinary Specialized Institute Kraljevo, Serbia	Vidanovic et al
EPI_ISL_661188	Scientific Veterinary Institute Novi Sad	Veterinary Specialized Institute Kraljevo, Serbia	Vidanovic et al
EPI_ISL_833574	Veterinary Specialized Instute Nis	Veterinary Specialized Institute Sabac, Serbia	Vidanovic et al
EPI_ISL_678488	Veterinary Specialized Institute Sabac, Serbia	Veterinary Specialized Institute Kraljevo, Serbia	Vidanovic et al
EPI_ISL_904011	Veterinary Specialized Institute Kraljevo	Veterinary Specialized Institute Kraljevo, Serbia	Vidanovic et al
EPI_ISL_676585	Scientific Veterinary Institute Novi Sad	Veterinary Specialized Institute Kraljevo, Serbia	Vidanovic et al

EPI_ISL_676589	Scientific Veterinary Institute Novi Sad	Veterinary Specialized Institute Kraljevo, Serbia	Vidanovic et al
EPI_ISL_676595	Scientific Veterinary Institute Novi Sad	Veterinary Specialized Institute Kraljevo, Serbia	Vidanovic et al
EPI_ISL_1626760	Institute of Virology, Biomedical Research Center of the Slovak Academy of Sciences, Bratislava	Faculty of Natural Sciences, Comenius University, Bratislava	Viktoria Cabanova et al
EPI_ISL_959638	Institute of Virology, Biomedical Research Center of the Slovak Academy of Sciences, Bratislava	Faculty of Natural Sciences, Comenius University, Bratislava	Viktória Čabanová et al
EPI_ISL_1239416	Institute of Virology, Biomedical Research Center of the Slovak Academy of Sciences, Bratislava	Faculty of Natural Sciences, Comenius University, Bratislava	Viktória Čabanová et al
EPI_ISL_875538	Institute of Virology, Biomedical Research Center of the Slovak Academy of Sciences, Bratislava	Faculty of Natural Sciences, Comenius University, Bratislava	Viktória Čabanová et al
EPI_ISL_577734	Institute of Virology, Biomedical Research Center of the Slovak Academy of Sciences, Bratislava	Faculty of Natural Sciences, Comenius University, Bratislava	Viktória Hodorová et al
EPI_ISL_577736	Institute of Virology, Biomedical Research Center of the Slovak Academy of Sciences, Bratislava	Faculty of Natural Sciences, Comenius University, Bratislava	Viktória Hodorová et al
EPI_ISL_577738	Institute of Virology, Biomedical Research Center of the Slovak Academy of Sciences, Bratislava	Faculty of Natural Sciences, Comenius University, Bratislava	Viktória Hodorová et al
EPI_ISL_583481	Institute of Virology, Biomedical Research Center of the Slovak Academy of Sciences, Bratislava	Faculty of Natural Sciences, Comenius University, Bratislava	Viktória Hodorová et al
EPI_ISL_1608137	LBM ALPHABIO, Marseille	LBM ALPHABIO, Marseille	Vincent GARCIA et al
EPI_ISL_1608138	LBM ALPHABIO, Marseille	LBM ALPHABIO, Marseille	Vincent GARCIA et al
EPI_ISL_831474	Laboratório de Microbiologia Molecular - Universidade FEEVALE	Universidade Federal de Ciências da Saúde de Porto Alegre	Vinícius Bonetti Franceschi et al
EPI_ISL_831645	Laboratório de Microbiologia Molecular - Universidade FEEVALE	Universidade Federal de Ciências da Saúde de Porto Alegre	Vinícius Bonetti Franceschi et al
EPI_ISL_831646	Laboratório de Microbiologia Molecular - Universidade FEEVALE	Universidade Federal de Ciências da Saúde de Porto Alegre	Vinícius Bonetti Franceschi et al
EPI_ISL_831660	Laboratório de Microbiologia Molecular - Universidade FEEVALE	Universidade Federal de Ciências da Saúde de Porto Alegre	Vinícius Bonetti Franceschi et al
EPI_ISL_831681	Laboratório de Microbiologia Molecular - Universidade FEEVALE	Universidade Federal de Ciências da Saúde de Porto Alegre	Vinícius Bonetti Franceschi et al
EPI_ISL_831683	Laboratório de Microbiologia Molecular - Universidade FEEVALE	Universidade Federal de Ciências da Saúde de Porto Alegre	Vinícius Bonetti Franceschi et al
EPI_ISL_831685	Laboratório de Microbiologia Molecular - Universidade FEEVALE	Universidade Federal de Ciências da Saúde de Porto Alegre	Vinícius Bonetti Franceschi et al
EPI_ISL_831688	Laboratório de Microbiologia Molecular - Universidade FEEVALE	Universidade Federal de Ciências da Saúde de Porto Alegre	Vinícius Bonetti Franceschi et al
EPI_ISL_831689	Laboratório de Microbiologia Molecular - Universidade FEEVALE	Universidade Federal de Ciências da Saúde de Porto Alegre	Vinícius Bonetti Franceschi et al
EPI_ISL_831892	Laboratório de Microbiologia Molecular - Universidade FEEVALE	Universidade Federal de Ciências da Saúde de Porto Alegre	Vinícius Bonetti Franceschi et al
EPI_ISL_831898	Laboratório de Microbiologia Molecular - Universidade FEEVALE	Universidade Federal de Ciências da Saúde de Porto Alegre	Vinícius Bonetti Franceschi et al
EPI_ISL_831913	Laboratório de Microbiologia Molecular - Universidade FEEVALE	Universidade Federal de Ciências da Saúde de Porto Alegre	Vinícius Bonetti Franceschi et al
EPI_ISL_831938	Laboratório de Microbiologia Molecular - Universidade FEEVALE	Universidade Federal de Ciências da Saúde de Porto Alegre	Vinícius Bonetti Franceschi et al
EPI_ISL_831939	Laboratório de Microbiologia Molecular - Universidade FEEVALE	Universidade Federal de Ciências da Saúde de Porto Alegre	Vinícius Bonetti Franceschi et al
EPI_ISL_831940	Laboratório de Microbiologia Molecular - Universidade FEEVALE	Universidade Federal de Ciências da Saúde de Porto Alegre	Vinícius Bonetti Franceschi et al

EPI_ISL_832009	Laboratório de Microbiologia Molecular - Universidade FEEVALE	Universidade Federal de Ciências da Saúde de Porto Alegre	Vinícius Bonetti Franceschi et al
EPI_ISL_832010	Laboratório de Microbiologia Molecular - Universidade FEEVALE	Universidade Federal de Ciências da Saúde de Porto Alegre	Vinícius Bonetti Franceschi et al
EPI_ISL_832011	Laboratório de Microbiologia Molecular - Universidade FEEVALE	Universidade Federal de Ciências da Saúde de Porto Alegre	Vinícius Bonetti Franceschi et al
EPI_ISL_832012	Laboratório de Microbiologia Molecular - Universidade FEEVALE	Universidade Federal de Ciências da Saúde de Porto Alegre	Vinícius Bonetti Franceschi et al
EPI_ISL_832013	Laboratório de Microbiologia Molecular - Universidade FEEVALE	Universidade Federal de Ciências da Saúde de Porto Alegre	Vinícius Bonetti Franceschi et al
EPI_ISL_831678	Laboratório de Microbiologia Molecular - Universidade FEEVALE	Universidade Federal de Ciências da Saúde de Porto Alegre	Vinícius Bonetti Franceschi et al
EPI_ISL_956391	Isolation - Virology Unit, Institut Pasteur du Cambodge; Sequencing - US National Institute of Allergy and Infectious Diseases Cambodia, US Naval Medical Research Unit -2, Cambodia National Institute for Public Health	Virology Unit, Institut Pasteur du Cambodge	Vireak Heang et al
EPI_ISL_956392	Isolation - Virology Unit, Institut Pasteur du Cambodge; Sequencing - US National Institute of Allergy and Infectious Diseases Cambodia, US Naval Medical Research Unit -2, Cambodia National Institute for Public Health	Virology Unit, Institut Pasteur du Cambodge	Vireak Heang et al
EPI_ISL_956393	Isolation - Virology Unit, Institut Pasteur du Cambodge; Sequencing - US National Institute of Allergy and Infectious Diseases Cambodia, US Naval Medical Research Unit -2, Cambodia National Institute for Public Health	Virology Unit, Institut Pasteur du Cambodge	Vireak Heang et al
EPI_ISL_1469286	RS Khusus Paru Karawang	National Institute of Health Research and Development	Vivi Setiawaty et al
EPI_ISL_1469254	RS Lira Medika Karawang	National Institute of Health Research and Development	Vivi Setiawaty et al
EPI_ISL_1257832	BTKL-PP Ambon	National Institute of Health Research and Development	Vivi Setiawaty et al
EPI_ISL_444610	U.S. Naval Medical Research Center Biological Defense Research Directorate	U.S. Naval Medical Research Center Biological Defense Research Directorate	Voegtly et al
EPI_ISL_406798	General Hospital of Central Theater Command of People's Liberation Army of China	BGI & Institute of Microbiology, Chinese Academy of Sciences & Shandong First Medical University & Shandong Academy of Medical Sciences & General Hospital of Central Theater Command of People's Liberation Army of China	Weijun Chen et al
EPI_ISL_1170957	HIV Molecular laboratory, Ethiopian Public Health Institute	HIV Molecular Laboratory	Weldemariam et al
EPI_ISL_1170958	HIV Molecular Laboratory, Ethiopian Public Health Institute, Ethiopia	HIV molecular lab, Ethiopian Public Health Institute, Ethiopian	Weldemariam et al
EPI_ISL_1169919	HIV Molecular Laboratory, Ethiopian Public Health Institute	HIV Molecular Laboratory, Ethiopian Public Health Institute	Weldemariam et al
EPI_ISL_1170956	HIV Molecular Lab, Ethiopian Public Health Institute	HIV Molecular Lab	Weldemariam et al
EPI_ISL_469254	National Institute for Viral Disease Control and Prevention, China CDC	Institute of Viral Disease Control and Prevention, China CDC	Wenjie Tan et al
EPI_ISL_824656	Cedars-Sinai Medical Center, Department of Pathology & Laboratory Medicine, Molecular Pathology Laboratory	Cedars-Sinai Medical Center, Molecular Pathology Laboratory of Department of Pathology & Laboratory Medicine and Genomic Core	Wenjuan Zhang et al
EPI_ISL_1117388	Nucleo de Pesquisa em Inovacao Terapeutica - UFPE	LABBE, Federal University of Pernambuco	Wilson Jose da Silva Junior et al
EPI_ISL_1117390	Nucleo de Pesquisa em Inovacao Terapeutica - UFPE	LABBE, Federal University of Pernambuco	Wilson Jose da Silva Junior et al
EPI_ISL_1117440	Nucleo de Pesquisa em Inovacao Terapeutica - UFPE	LABBE, Federal University of Pernambuco	Wilson Jose da Silva Junior et al
EPI_ISL_1117399	Nucleo de Pesquisa em Inovacao Terapeutica - UFPE	LABBE, Federal University of Pernambuco	Wilson Jose da Silva Junior et al
EPI_ISL_1117393	Nucleo de Pesquisa em Inovacao Terapeutica - UFPE	LABBE, Federal University of Pernambuco	Wilson Jose da Silva Junior et al

EPI_ISL_1117395	Nucleo de Pesquisa em Inovacao Terapeutica - UFPE	LABBE, Federal University of Pernambuco	Wilson Jose da Silva Junior et al
EPI_ISL_1117443	Nucleo de Pesquisa em Inovacao Terapeutica - UFPE	LABBE, Federal University of Pernambuco	Wilson Jose da Silva Junior et al
EPI_ISL_1117435	Nucleo de Pesquisa em Inovacao Terapeutica - UFPE	LABBE, Federal University of Pernambuco	Wilson Jose da Silva Junior et al
EPI_ISL_1117428	Nucleo de Pesquisa em Inovacao Terapeutica - UFPE	LABBE, Federal University of Pernambuco	Wilson Jose da Silva Junior et al
EPI_ISL_1117405	Nucleo de Pesquisa em Inovacao Terapeutica - UFPE	LABBE, Federal University of Pernambuco	Wilson Jose da Silva Junior et al
EPI_ISL_1117384	Nucleo de Pesquisa em Inovacao Terapeutica - UFPE	LABBE, Federal University of Pernambuco	Wilson Jose da Silva Junior et al
EPI_ISL_1117386	Nucleo de Pesquisa em Inovacao Terapeutica - UFPE	LABBE, Federal University of Pernambuco	Wilson Jose da Silva Junior et al
EPI_ISL_1117432	Nucleo de Pesquisa em Inovacao Terapeutica - UFPE	LABBE, Federal University of Pernambuco	Wilson Jose da Silva Junior et al
EPI_ISL_1117433	Nucleo de Pesquisa em Inovacao Terapeutica - UFPE	LABBE, Federal University of Pernambuco	Wilson Jose da Silva Junior et al
EPI_ISL_1117434	Nucleo de Pesquisa em Inovacao Terapeutica - UFPE	LABBE, Federal University of Pernambuco	Wilson Jose da Silva Junior et al
EPI_ISL_1117436	Nucleo de Pesquisa em Inovacao Terapeutica - UFPE	LABBE, Federal University of Pernambuco	Wilson Jose da Silva Junior et al
EPI_ISL_1117442	Nucleo de Pesquisa em Inovacao Terapeutica - UFPE	LABBE, Federal University of Pernambuco	Wilson Jose da Silva Junior et al
EPI_ISL_1117404	Nucleo de Pesquisa em Inovacao Terapeutica - UFPE	LABBE, Federal University of Pernambuco	Wilson Jose da Silva Junior et al
EPI_ISL_1117401	Nucleo de Pesquisa em Inovacao Terapeutica - UFPE	LABBE, Federal University of Pernambuco	Wilson Jose da Silva Junior et al
EPI_ISL_1117402	Nucleo de Pesquisa em Inovacao Terapeutica - UFPE	LABBE, Federal University of Pernambuco	Wilson Jose da Silva Junior et al
EPI_ISL_1117379	Nucleo de Pesquisa em Inovacao Terapeutica - UFPE	LABBE, Federal University of Pernambuco	Wilson Jose da Silva Junior et al
EPI_ISL_1117380	Nucleo de Pesquisa em Inovacao Terapeutica - UFPE	LABBE, Federal University of Pernambuco	Wilson Jose da Silva Junior et al
EPI_ISL_1117381	Nucleo de Pesquisa em Inovacao Terapeutica - UFPE	LABBE, Federal University of Pernambuco	Wilson Jose da Silva Junior et al
EPI_ISL_1117382	Nucleo de Pesquisa em Inovacao Terapeutica - UFPE	LABBE, Federal University of Pernambuco	Wilson Jose da Silva Junior et al
EPI_ISL_1117449	Nucleo de Pesquisa em Inovacao Terapeutica - UFPE	LABBE, Federal University of Pernambuco	Wilson Jose da Silva Junior et al
EPI_ISL_1117385	Nucleo de Pesquisa em Inovacao Terapeutica - UFPE	LABBE, Federal University of Pernambuco	Wilson Jose da Silva Junior et al
EPI_ISL_1117429	Nucleo de Pesquisa em Inovacao Terapeutica - UFPE	LABBE, Federal University of Pernambuco	Wilson Jose da Silva Junior et al
EPI_ISL_1117408	Nucleo de Pesquisa em Inovacao Terapeutica - UFPE	LABBE, Federal University of Pernambuco	Wilson Jose da Silva Junior et al
EPI_ISL_1117417	Nucleo de Pesquisa em Inovacao Terapeutica - UFPE	LABBE, Federal University of Pernambuco	Wilson Jose da Silva Junior et al
EPI_ISL_882957	Specjalistyczny Szpital im. Dra A. Sokołowskiego	National Institute of Public Health - National Institute of Hygiene	Wołkowicz Tomasz et al
EPI_ISL_882960	Laboratorium Diagnostyi Mikrobiologicznej z Pracownią Prątka Gruźlicy SPSzW im. Jana Bożego w Lublinie	National Institute of Public Health - National Institute of Hygiene	Wołkowicz Tomasz et al
EPI_ISL_882961	Laboratorium Diagnostyi Mikrobiologicznej z Pracownią Prątka Gruźlicy SPSzW im. Jana Bożego w Lublinie	National Institute of Public Health - National Institute of Hygiene	Wołkowicz Tomasz et al
EPI_ISL_1239619	Laboratorum Epidemiologii WSSE w Szczecinie	1. National Institute of Public Health - National Institute of Hygiene; 2. Eurofins Genomics Europe Sequencing GmbH	Wołkowicz Tomasz et al
EPI_ISL_1393660	Dział Diagnostyki Laboratoryjnej SPZOZ	1. National Institute of Public Health - National Institute of Hygiene; 2. Eurofins Genomics Europe Sequencing GmbH	Wołkowicz Tomasz et al

EPI_ISL_1393683	Labolatorium Biologi Molekularnej USK	1. National Institute of Public Health - National Institute of Hygiene; 2. Eurofins Genomics Europe Sequencing GmbH	Wołkowicz Tomasz et al
EPI_ISL_1393827	Laboratorium Medyczne LAB-MED	1. National Institute of Public Health - National Institute of Hygiene; 2. Eurofins Genomics Europe Sequencing GmbH	Wołkowicz Tomasz et al
EPI_ISL_413557	California Department of Public Health	Chiu Laboratory, University of California, San Francisco	Xianding Deng et al
EPI_ISL_435585	Santa Clara County Public Health Department	Chiu Laboratory, University of California, San Francisco	Xianding Deng et al
EPI_ISL_850948	National Institute for Viral Disease Control and Prevention, China CDC	National Institute for Viral Disease Control and Prevention, China CDC	Xiang Zhao et al
EPI_ISL_469255	National Institute for Viral Disease Control and Prevention, China CDC	Institute of Viral Disease Control and Prevention, China CDC	Xiang Zhao et al
EPI_ISL_850946	National Institute for Viral Disease Control and Prevention, China CDC	National Institute for Viral Disease Control and Prevention, China CDC	Xiang Zhao et al
EPI_ISL_850950	National Institute for Viral Disease Control and Prevention, China CDC	National Institute for Viral Disease Control and Prevention, China CDC	Xiang Zhao et al
EPI_ISL_498692	National Institute for Viral Disease Control and Prevention, China CDC	National Institute for Viral Disease Control and Prevention, China CDC	Xiang Zhao et al
EPI_ISL_498694	National Institute for Viral Disease Control and Prevention, China CDC	National Institute for Viral Disease Control and Prevention, China CDC	Xiang Zhao et al
EPI_ISL_498693	National Institute for Viral Disease Control and Prevention, China CDC	National Institute for Viral Disease Control and Prevention, China CDC	Xiang Zhao et al
EPI_ISL_498691	National Institute for Viral Disease Control and Prevention, China CDC	National Institute for Viral Disease Control and Prevention, China CDC	Xiang Zhao et al
EPI_ISL_850949	National Institute for Viral Disease Control and Prevention, China CDC	National Institute for Viral Disease Control and Prevention, China CDC	Xiang Zhao et al
EPI_ISL_962578	Infectious Disease Control and Prevention Institute	Infectious Disease Control and Prevention Institute	Xiaolin Jiang et al
EPI_ISL_962568	Infectious Disease Control and Prevention Institute	Infectious Disease Control and Prevention Institute	Xiaolin Jiang et al
EPI_ISL_962872	Infectious Disease Control and Prevention Institute	Infectious Disease Control and Prevention Institute	Xiaolin Jiang et al
EPI_ISL_962544	Infectious Disease Control and Prevention Institute	Infectious Disease Control and Prevention Institute	Xiaolin Jiang et al
EPI_ISL_962871	Infectious Disease Control and Prevention Institute	Infectious Disease Control and Prevention Institute	Xiaolin Jiang et al
EPI_ISL_962868	Infectious Disease Control and Prevention Institute	Infectious Disease Control and Prevention Institute	Xiaolin Jiang et al
EPI_ISL_548104	LabTests	Institute of Environmental Science and Research (ESR)	Xiaoyun Ren et al
EPI_ISL_548105	LabTests	Institute of Environmental Science and Research (ESR)	Xiaoyun Ren et al
EPI_ISL_579059	LabPLUS	Institute of Environmental Science and Research (ESR)	Xiaoyun Ren et al
EPI_ISL_548110	LabPLUS	Institute of Environmental Science and Research (ESR)	Xiaoyun Ren et al
EPI_ISL_548114	Middlemore Hospital	Institute of Environmental Science and Research (ESR)	Xiaoyun Ren et al
EPI_ISL_548115	Middlemore Hospital	Institute of Environmental Science and Research (ESR)	Xiaoyun Ren et al
EPI_ISL_622788	Canterbury Health Laboratories	Institute of Environmental Science and Research (ESR)	Xiaoyun Ren et al
EPI_ISL_579100	Middlemore Hospital	Institute of Environmental Science and Research (ESR)	Xiaoyun Ren et al
EPI_ISL_579114	LabPLUS	Institute of Environmental Science and Research (ESR)	Xiaoyun Ren et al
EPI_ISL_622774	LabPLUS	Institute of Environmental Science and Research (ESR)	Xiaoyun Ren et al
EPI_ISL_622772	Middlemore Hospital	Institute of Environmental Science and Research (ESR)	Xiaoyun Ren et al

EPI_ISL_622818	Middlemore Hospital	Institute of Environmental Science and Research (ESR)	Xiaoyun Ren et al
EPI_ISL_661260	Canterbury Health Laboratories	Institute of Environmental Science and Research (ESR)	Xiaoyun Ren et al
EPI_ISL_682291	LabPLUS	Institute of Environmental Science and Research (ESR)	Xiaoyun Ren et al
EPI_ISL_707794	Waikato Hospital	Institute of Environmental Science and Research (ESR)	Xiaoyun Ren et al
EPI_ISL_707800	LabPLUS	Institute of Environmental Science and Research (ESR)	Xiaoyun Ren et al
EPI_ISL_732962	LabPLUS	Institute of Environmental Science and Research (ESR)	Xiaoyun Ren et al
EPI_ISL_755623	Canterbury Health Laboratories	Institute of Environmental Science and Research (ESR)	Xiaoyun Ren et al
EPI_ISL_755626	PathLab Bay of Plenty	Institute of Environmental Science and Research (ESR)	Xiaoyun Ren et al
EPI_ISL_755633	LabPLUS	Institute of Environmental Science and Research (ESR)	Xiaoyun Ren et al
EPI_ISL_755634	Middlemore Hospital	Institute of Environmental Science and Research (ESR)	Xiaoyun Ren et al
EPI_ISL_755635	Middlemore Hospital	Institute of Environmental Science and Research (ESR)	Xiaoyun Ren et al
EPI_ISL_755638	Canterbury Health Laboratories	Institute of Environmental Science and Research (ESR)	Xiaoyun Ren et al
EPI_ISL_579220	Canterbury Health Laboratories	Institute of Environmental Science and Research (ESR)	Xiaoyun Ren et al
EPI_ISL_579403	LabPLUS	Institute of Environmental Science and Research (ESR)	Xiaoyun Ren et al
EPI_ISL_579406	North Shore Hospital	Institute of Environmental Science and Research (ESR)	Xiaoyun Ren et al
EPI_ISL_579407	North Shore Hospital	Institute of Environmental Science and Research (ESR)	Xiaoyun Ren et al
EPI_ISL_579422	LabTests	Institute of Environmental Science and Research (ESR)	Xiaoyun Ren et al
EPI_ISL_579425	LabPLUS	Institute of Environmental Science and Research (ESR)	Xiaoyun Ren et al
EPI_ISL_579426	Canterbury Health Laboratories	Institute of Environmental Science and Research (ESR)	Xiaoyun Ren et al
EPI_ISL_548130	Middlemore Hospital	Institute of Environmental Science and Research (ESR)	Xiaoyun Ren et al
EPI_ISL_548133	Middlemore Hospital	Institute of Environmental Science and Research (ESR)	Xiaoyun Ren et al
EPI_ISL_548138	Canterbury Health Laboratories	Institute of Environmental Science and Research (ESR)	Xiaoyun Ren et al
EPI_ISL_548139	Canterbury Health Laboratories	Institute of Environmental Science and Research (ESR)	Xiaoyun Ren et al
EPI_ISL_548140	Canterbury Health Laboratories	Institute of Environmental Science and Research (ESR)	Xiaoyun Ren et al
EPI_ISL_548145	Middlemore Hospital	Institute of Environmental Science and Research (ESR)	Xiaoyun Ren et al
EPI_ISL_548095	Middlemore Hospital	Institute of Environmental Science and Research (ESR)	Xiaoyun Ren et al
EPI_ISL_843193	LabPLUS	Institute of Environmental Science and Research (ESR)	Xiaoyun Ren et al
EPI_ISL_877209	LabPLUS	Institute of Environmental Science and Research (ESR)	Xiaoyun Ren et al
EPI_ISL_877212	LabPLUS	Institute of Environmental Science and Research (ESR)	Xiaoyun Ren et al
EPI_ISL_877216	Middlemore Hospital	Institute of Environmental Science and Research (ESR)	Xiaoyun Ren et al

EPI_ISL_877217	Middlemore Hospital	Institute of Environmental Science and Research (ESR)	Xiaoyun Ren et al
EPI_ISL_877221	LabPLUS	Institute of Environmental Science and Research (ESR)	Xiaoyun Ren et al
EPI_ISL_877226	LabPLUS	Institute of Environmental Science and Research (ESR)	Xiaoyun Ren et al
EPI_ISL_1016868	Middlemore Hospital	Institute of Environmental Science and Research (ESR)	Xiaoyun Ren et al
EPI_ISL_1016874	Middlemore Hospital	Institute of Environmental Science and Research (ESR)	Xiaoyun Ren et al
EPI_ISL_1016847	LabPLUS	Institute of Environmental Science and Research (ESR)	Xiaoyun Ren et al
EPI_ISL_1016846	LabPLUS	Institute of Environmental Science and Research (ESR)	Xiaoyun Ren et al
EPI_ISL_1016869	LabPLUS	Institute of Environmental Science and Research (ESR)	Xiaoyun Ren et al
EPI_ISL_1016865	Middlemore Hospital	Institute of Environmental Science and Research (ESR)	Xiaoyun Ren et al
EPI_ISL_1016873	LabPLUS	Institute of Environmental Science and Research (ESR)	Xiaoyun Ren et al
EPI_ISL_1016872	Middlemore Hospital	Institute of Environmental Science and Research (ESR)	Xiaoyun Ren et al
EPI_ISL_1016844	Middlemore Hospital	Institute of Environmental Science and Research (ESR)	Xiaoyun Ren et al
EPI_ISL_1016871	LabPLUS	Institute of Environmental Science and Research (ESR)	Xiaoyun Ren et al
EPI_ISL_1016854	LabPLUS	Institute of Environmental Science and Research (ESR)	Xiaoyun Ren et al
EPI_ISL_476827	Laboratoire des Fièvres Hémorragiques Virales du Benin	Charité-Universitätsmedizin Berlin	Yadouleton et al
EPI_ISL_476830	Laboratoire des Fièvres Hémorragiques Virales du Benin	Charité-Universitätsmedizin Berlin	Yadouleton et al
EPI_ISL_476831	Laboratoire des Fièvres Hémorragiques Virales du Benin	Charité-Universitätsmedizin Berlin	Yadouleton et al
EPI_ISL_476823	Laboratoire des Fièvres Hémorragiques Virales du Benin	Charité-Universitätsmedizin Berlin	Yadouleton et al
EPI_ISL_476834	Laboratoire des Fièvres Hémorragiques Virales du Benin	Charité-Universitätsmedizin Berlin	Yadouleton et al
EPI_ISL_476833	Laboratoire des Fièvres Hémorragiques Virales du Benin	Charité-Universitätsmedizin Berlin	Yadouleton et al
EPI_ISL_476829	Laboratoire des Fièvres Hémorragiques Virales du Benin	Charité-Universitätsmedizin Berlin	Yadouleton et al
EPI_ISL_1225337	Gorgas Memorial Laboratory of Health Studies	Gorgas Memorial Laboratory of Health Studies	Yamilka Diaz et al
EPI_ISL_1225364	Gorgas Memorial Laboratory of Health Studies	Gorgas Memorial Laboratory of Health Studies	Yamilka Diaz et al
EPI_ISL_1225373	Gorgas Memorial Laboratory of Health Studies	Gorgas Memorial Laboratory of Health Studies	Yamilka Diaz et al
EPI_ISL_1225382	Gorgas Memorial Laboratory of Health Studies	Gorgas Memorial Laboratory of Health Studies	Yamilka Diaz et al
EPI_ISL_1225391	Gorgas Memorial Laboratory of Health Studies	Gorgas Memorial Laboratory of Health Studies	Yamilka Diaz et al
EPI_ISL_1225424	Gorgas Memorial Laboratory of Health Studies	Gorgas Memorial Laboratory of Health Studies	Yamilka Diaz et al
EPI_ISL_1225429	Gorgas Memorial Laboratory of Health Studies	Gorgas Memorial Laboratory of Health Studies	Yamilka Diaz et al
EPI_ISL_1225463	Gorgas Memorial Laboratory of Health Studies	Gorgas Memorial Laboratory of Health Studies	Yamilka Diaz et al
EPI_ISL_1225481	Gorgas Memorial Laboratory of Health Studies	Gorgas Memorial Laboratory of Health Studies	Yamilka Diaz et al

EPI_ISL_1225490	Gorgas Memorial Laboratory of Health Studies	Gorgas Memorial Laboratory of Health Studies	Yamilka Diaz et al
EPI_ISL_1225491	Gorgas Memorial Laboratory of Health Studies	Gorgas Memorial Laboratory of Health Studies	Yamilka Diaz et al
EPI_ISL_1225534	Gorgas Memorial Laboratory of Health Studies	Gorgas Memorial Laboratory of Health Studies	Yamilka Diaz et al
EPI_ISL_1225537	Gorgas Memorial Laboratory of Health Studies	Gorgas Memorial Laboratory of Health Studies	Yamilka Diaz et al
EPI_ISL_1225555	Gorgas Memorial Laboratory of Health Studies	Gorgas Memorial Laboratory of Health Studies	Yamilka Diaz et al
EPI_ISL_1225556	Gorgas Memorial Laboratory of Health Studies	Gorgas Memorial Laboratory of Health Studies	Yamilka Diaz et al
EPI_ISL_1225560	Gorgas Memorial Laboratory of Health Studies	Gorgas Memorial Laboratory of Health Studies	Yamilka Diaz et al
EPI_ISL_1225572	Gorgas Memorial Laboratory of Health Studies	Gorgas Memorial Laboratory of Health Studies	Yamilka Diaz et al
EPI_ISL_1225574	Gorgas Memorial Laboratory of Health Studies	Gorgas Memorial Laboratory of Health Studies	Yamilka Diaz et al
EPI_ISL_1225577	Gorgas Memorial Laboratory of Health Studies	Gorgas Memorial Laboratory of Health Studies	Yamilka Diaz et al
EPI_ISL_450793	Jamaica Ministry of Health and Wellness	Pathogen Discovery, Respiratory Viruses Branch, Division of Viral Diseases, Centers for Disease Control and Prevention	Yan Li et al
EPI_ISL_450794	Jamaica Ministry of Health and Wellness	Pathogen Discovery, Respiratory Viruses Branch, Division of Viral Diseases, Centers for Disease Control and Prevention	Yan Li et al
EPI_ISL_450795	Jamaica Ministry of Health and Wellness	Pathogen Discovery, Respiratory Viruses Branch, Division of Viral Diseases, Centers for Disease Control and Prevention	Yan Li et al
EPI_ISL_450796	Jamaica Ministry of Health and Wellness	Pathogen Discovery, Respiratory Viruses Branch, Division of Viral Diseases, Centers for Disease Control and Prevention	Yan Li et al
EPI_ISL_450797	Jamaica Ministry of Health and Wellness	Pathogen Discovery, Respiratory Viruses Branch, Division of Viral Diseases, Centers for Disease Control and Prevention	Yan Li et al
EPI_ISL_450799	Jamaica Ministry of Health and Wellness	Pathogen Discovery, Respiratory Viruses Branch, Division of Viral Diseases, Centers for Disease Control and Prevention	Yan Li et al
EPI_ISL_1591094	NAMRU-6	Pathogen Discovery, Respiratory Viruses Branch, Division of Viral Diseases, Centers for Disease Control and Prevention	Yan Li et al
EPI_ISL_1591098	NAMRU-6	Pathogen Discovery, Respiratory Viruses Branch, Division of Viral Diseases, Centers for Disease Control and Prevention	Yan Li et al
EPI_ISL_1591097	NAMRU-6	Pathogen Discovery, Respiratory Viruses Branch, Division of Viral Diseases, Centers for Disease Control and Prevention	Yan Li et al
EPI_ISL_1591096	NAMRU-6	Pathogen Discovery, Respiratory Viruses Branch, Division of Viral Diseases, Centers for Disease Control and Prevention	Yan Li et al
EPI_ISL_1591095	NAMRU-6	Pathogen Discovery, Respiratory Viruses Branch, Division of Viral Diseases, Centers for Disease Control and Prevention	Yan Li et al
EPI_ISL_410045	IL Department of Public Health Chicago Laboratory	Pathogen Discovery, Respiratory Viruses Branch, Division of Viral Diseases, Centers for Disease Control and Prevention	Yan Li et al
EPI_ISL_1117378	Beijing Center for Disease Prevention and Control	Beijing Center for Disease Prevention and Control	Yang Pan et al
EPI_ISL_1117377	Beijing Center for Disease Prevention and Control	Beijing Center for Disease Prevention and Control	Yang Pan et al
EPI_ISL_1117376	Beijing Center for Disease Prevention and Control	Beijing Center for Disease Prevention and Control	Yang Pan et al
EPI_ISL_1121993	Emergency department of COVID-19, Beijing Ditan Hospital	National Institute for Viral Disease Control and Prevention	Yang Song2* et al

EPI_ISL_1122015	Emergency department of COVID-19, Beijing Ditan Hospital	National Institute for Viral Disease Control and Prevention	Yang Song2* et al
EPI_ISL_1122016	Emergency department of COVID-19, Beijing Ditan Hospital	National Institute for Viral Disease Control and Prevention	Yang Song2* et al
EPI_ISL_1122017	Emergency department of COVID-19, Beijing Ditan Hospital, Capital Medical University, Beijing, China.	National Institute for Viral Disease Control and Prevention	Yang Song2* et al
EPI_ISL_548945	Institute of Microbiology, University of Veterinary and Animal sciences	Institute of Microbiology, University of Veterinary and Animal sciences	Yaqub et al
EPI_ISL_509695	Guatemala Ministry of Public Health	Pathogen Discovery, Respiratory Viruses Branch, Division of Viral Diseases, Centers for Disease Control and Prevention	Ying Tao et al
EPI_ISL_509696	Guatemala Ministry of Public Health	Pathogen Discovery, Respiratory Viruses Branch, Division of Viral Diseases, Centers for Disease Control and Prevention	Ying Tao et al
EPI_ISL_509700	Guatemala Ministry of Public Health	Pathogen Discovery, Respiratory Viruses Branch, Division of Viral Diseases, Centers for Disease Control and Prevention	Ying Tao et al
EPI_ISL_509701	Guatemala Ministry of Public Health	Pathogen Discovery, Respiratory Viruses Branch, Division of Viral Diseases, Centers for Disease Control and Prevention	Ying Tao et al
EPI_ISL_509702	Guatemala Ministry of Public Health	Pathogen Discovery, Respiratory Viruses Branch, Division of Viral Diseases, Centers for Disease Control and Prevention	Ying Tao et al
EPI_ISL_509703	Guatemala Ministry of Public Health	Pathogen Discovery, Respiratory Viruses Branch, Division of Viral Diseases, Centers for Disease Control and Prevention	Ying Tao et al
EPI_ISL_406223	Arizona Department of Health Services	Pathogen Discovery, Respiratory Viruses Branch, Division of Viral Diseases, Centers for Disease Control and Prevention	Ying Tao et al
EPI_ISL_408010	California Department of Health	Pathogen Discovery, Respiratory Viruses Branch, Division of Viral Diseases, Centers for Disease Control and Prevention	Ying Tao et al
EPI_ISL_404253	IL Department of Public Health Chicago Laboratory	Pathogen Discovery, Respiratory Viruses Branch, Division of Viral Diseases, Centers for Disease Control and Prevention	Ying Tao et al
EPI_ISL_501197	Department of Medical Microbiology, University Malaya Medical Centre	Department of Medical Microbiology, Faculty of Medicine, University of Malaya	Yoong Min CHONG et al
EPI_ISL_506998	Department of Medical Microbiology, University Malaya Medical Centre	Department of Medical Microbiology, Faculty of Medicine, University of Malaya	Yoong Min CHONG et al
EPI_ISL_501202	Department of Medical Microbiology, University Malaya Medical Centre	Department of Medical Microbiology, Faculty of Medicine, University of Malaya	Yoong Min CHONG et al
EPI_ISL_501208	Department of Medical Microbiology, University Malaya Medical Centre	Department of Medical Microbiology, Faculty of Medicine, University of Malaya	Yoong Min CHONG et al
EPI_ISL_501211	Department of Medical Microbiology, University Malaya Medical Centre	Department of Medical Microbiology, Faculty of Medicine, University of Malaya	Yoong Min CHONG et al
EPI_ISL_506999	Department of Medical Microbiology, University Malaya Medical Centre	Department of Medical Microbiology, Faculty of Medicine, University of Malaya	Yoong Min CHONG et al
EPI_ISL_501213	Department of Medical Microbiology, University Malaya Medical Centre	Department of Medical Microbiology, Faculty of Medicine, University of Malaya	Yoong Min CHONG et al
EPI_ISL_501216	Department of Medical Microbiology, University Malaya Medical Centre	Department of Medical Microbiology, Faculty of Medicine, University of Malaya	Yoong Min CHONG et al
EPI_ISL_501217	Department of Medical Microbiology, University Malaya Medical Centre	Department of Medical Microbiology, Faculty of Medicine, University of Malaya	Yoong Min CHONG et al
EPI_ISL_501220	Department of Medical Microbiology, University Malaya Medical Centre	Department of Medical Microbiology, Faculty of Medicine, University of Malaya	Yoong Min CHONG et al

EPI_ISL_507000	Department of Medical Microbiology, University Malaya Medical Centre	Department of Medical Microbiology, Faculty of Medicine, University of Malaya	Yoong Min CHONG et al
EPI_ISL_1595847	Genome Analysis Center, Yamanashi Central Hospital	Genome Analysis Center, Yamanashi Central Hospital	Yosuke Hirotsu et al
EPI_ISL_1595849	Genome Analysis Center, Yamanashi Central Hospital	Genome Analysis Center, Yamanashi Central Hospital	Yosuke Hirotsu et al
EPI_ISL_1595851	Genome Analysis Center, Yamanashi Central Hospital	Genome Analysis Center, Yamanashi Central Hospital	Yosuke Hirotsu et al
EPI_ISL_1669765	Genome Analysis Center, Yamanashi Central Hospital	Genome Analysis Center, Yamanashi Central Hospital	Yosuke Hirotsu et al
EPI_ISL_925850	Nucleic Acid Testing, National Reference Laboratory	GIGA Medical Genomics	Yvan Butera et al
EPI_ISL_925851	Nucleic Acid Testing, National Reference Laboratory	GIGA Medical Genomics	Yvan Butera et al
EPI_ISL_925852	Nucleic Acid Testing, National Reference Laboratory	GIGA Medical Genomics	Yvan Butera et al
EPI_ISL_925883	Nucleic Acid Testing, National Reference Laboratory	GIGA Medical Genomics	Yvan Butera et al
EPI_ISL_925898	Nucleic Acid Testing, National Reference Laboratory	GIGA Medical Genomics	Yvan Butera et al
EPI_ISL_925901	Nucleic Acid Testing, National Reference Laboratory	GIGA Medical Genomics	Yvan Butera et al
EPI_ISL_925902	Nucleic Acid Testing, National Reference Laboratory	GIGA Medical Genomics	Yvan Butera et al
EPI_ISL_925904	Nucleic Acid Testing, National Reference Laboratory	GIGA Medical Genomics	Yvan Butera et al
EPI_ISL_1020288	Nucleic Acid Testing, National Reference Laboratory	GIGA Medical Genomics	Yvan Butera et al
EPI_ISL_960228	Nucleic Acid Testing, National Reference Laboratory	GIGA Medical Genomics	Yvan Butera et al
EPI_ISL_960278	Nucleic Acid Testing, National Reference Laboratory	GIGA Medical Genomics	Yvan Butera et al
EPI_ISL_960302	Nucleic Acid Testing, National Reference Laboratory	GIGA Medical Genomics	Yvan Butera et al
EPI_ISL_960301	Nucleic Acid Testing, National Reference Laboratory	GIGA Medical Genomics	Yvan Butera et al
EPI_ISL_960300	Nucleic Acid Testing, National Reference Laboratory	GIGA Medical Genomics	Yvan Butera et al
EPI_ISL_960288	Nucleic Acid Testing, National Reference Laboratory	GIGA Medical Genomics	Yvan Butera et al
EPI_ISL_1301779	Nucleic Acid Testing, National Reference Laboratory	GIGA Medical Genomics	Yvan Butera et al
EPI_ISL_1301784	Nucleic Acid Testing, National Reference Laboratory	GIGA Medical Genomics	Yvan Butera et al
EPI_ISL_1301790	Nucleic Acid Testing, National Reference Laboratory	GIGA Medical Genomics	Yvan Butera et al
EPI_ISL_1301741	Nucleic Acid Testing, National Reference Laboratory	GIGA Medical Genomics	Yvan Butera et al
EPI_ISL_1301793	Nucleic Acid Testing, National Reference Laboratory	GIGA Medical Genomics	Yvan Butera et al
EPI_ISL_1301742	Nucleic Acid Testing, National Reference Laboratory	GIGA Medical Genomics	Yvan Butera et al
EPI_ISL_1301794	Nucleic Acid Testing, National Reference Laboratory	GIGA Medical Genomics	Yvan Butera et al
EPI_ISL_1301736	Nucleic Acid Testing, National Reference Laboratory	GIGA Medical Genomics	Yvan Butera et al
EPI_ISL_625683	National Reference Laboratory for COVID-19, Pasteur Institute of Iran	National Reference Laboratory for COVID-19, Pasteur Institute of Iran	Zahra Ahmadi et al
EPI_ISL_833578	National Reference Laboratory for COVID-19, Pasteur Institute of Iran	National Reference Laboratory for COVID-19, Pasteur Institute of Iran	Zahra Ahmadi et al

EPI_ISL_1139147	National Center of Disease Control and Prevention of the Republic of Armenia	Institute of Molecular Biology NAS RA, Republic of Armenia, Department of Bioengineering, BioinformaticsInstitute and Molecular Biology IBMPh RAU, Republic of Armenia	Zaven Karalyan et al
EPI_ISL_1299979	OKMI FN Brno	CEITEC MU	Zdrazilova Dubska L. et al
EPI_ISL_1300005	OKMI FN Brno	CEITEC MU	Zdrazilova Dubska L. et al
EPI_ISL_468062	Egyptian National Cancer Institute (ENCI)	Egyptian National Cancer Institute (ENCI)	Zekri et al
EPI_ISL_907096	Cancer Biology Department, National Cancer Institute	Cancer Biology Department, National Cancer Institute	Zekri et al
EPI_ISL_907097	Cancer Biology Department, National Cancer Institute	Cancer Biology Department, National Cancer Institute	Zekri et al
EPI_ISL_907099	Cancer Biology Department, National Cancer Institute	Cancer Biology Department, National Cancer Institute	Zekri et al
EPI_ISL_475724	Egyptian National Cancer Institute (ENCI)	Egyptian National Cancer Institute (ENCI)	Zekri et al
EPI_ISL_857332	Cancer Biology Department, National Cancer Institute	Cancer Biology Department, National Cancer Institute	Zekri et al
EPI_ISL_857330	Cancer Biology Department, National Cancer Institute	Cancer Biology Department, National Cancer Institute	Zekri et al
EPI_ISL_857336	Cancer Biology Department, National Cancer Institute	Cancer Biology Department, National Cancer Institute	Zekri et al
EPI_ISL_857347	Cancer Biology Department, National Cancer Institute	Cancer Biology Department, National Cancer Institute	Zekri et al
EPI_ISL_402125	National Institute for Communicable Disease Control and Prevention (ICDC) Chinese Center for Disease Control and Prevention (China CDC)	National Institute for Communicable Disease Control and Prevention (ICDC) Chinese Center for Disease Control and Prevention (China CDC)	Zhang et al
EPI_ISL_430847	HS mikrobiologi virus	The Public Health Agency of Sweden	Zhibing Yun et al
EPI_ISL_1055106	National Virus Reference Laboratory	National Virus Reference Laboratory	Zoe Yandle et al
EPI_ISL_1180385	National Virus Reference Laboratory	National Virus Reference Laboratory	Zoe Yandle et al
EPI_ISL_1180388	National Virus Reference Laboratory	National Virus Reference Laboratory	Zoe Yandle et al
EPI_ISL_1623011	National Virus Reference Laboratory	National Virus Reference Laboratory	Zoe Yandle et al
EPI_ISL_1623005	National Virus Reference Laboratory	National Virus Reference Laboratory	Zoe Yandle et al
EPI_ISL_1623006	National Virus Reference Laboratory	National Virus Reference Laboratory	Zoe Yandle et al
EPI_ISL_1552560	National Virus Reference Laboratory	National Virus Reference Laboratory	Zoe Yandle et al
EPI_ISL_1117702	National Virus Reference Laboratory	National Virus Reference Laboratory	Zoe Yandle et al
EPI_ISL_1657504	National Virus Reference Laboratory	National Virus Reference Laboratory	Zoe Yandle et al
EPI_ISL_1657422	National Virus Reference Laboratory	National Virus Reference Laboratory	Zoe Yandle et al
EPI_ISL_1164141	National Virus Reference Laboratory	National Virus Reference Laboratory	Zoe Yandle et al
EPI_ISL_1191448	National Virus Reference Laboratory	National Virus Reference Laboratory	Zoe Yandle et al
EPI_ISL_1577467	National Virus Reference Laboratory	National Virus Reference Laboratory	Zoe Yandle et al
EPI_ISL_1577559	National Virus Reference Laboratory	National Virus Reference Laboratory	Zoe Yandle et al
EPI_ISL_594188	Department of Pathology, School of Medicine, Imam Khomeini Hospital, Tehran University of Medical Sciences	Genetics Research Center, University of Social Welfare and Rehabilitation Sciences	Zohreh Fattahi et al

EPI_ISL_596451	Department of Pathology, School of Medicine, Imam Khomeini Hospital, Tehran University of Medical Sciences	Genetics Research Center, University of Social Welfare and Rehabilitation Sciences	Zohreh Fattahi et al
EPI_ISL_596454	Infectious Disease and Tropical Medicine Research Center, Resistant Tuberculosis Institute, Zahedan University of Medical Sciences, Zahedan, Iran.	Genetics Research Center, University of Social Welfare and Rehabilitation Sciences	Zohreh Fattahi et al
EPI_ISL_596452	Department of Pathology, School of Medicine, Imam Khomeini Hospital, Tehran University of Medical Sciences	Genetics Research Center, University of Social Welfare and Rehabilitation Sciences	Zohreh Fattahi et al
EPI_ISL_596455	Department of Pathology, School of Medicine, Imam Khomeini Hospital, Tehran University of Medical Sciences	Genetics Research Center, University of Social Welfare and Rehabilitation Sciences	Zohreh Fattahi et al
