# Supplementary figures and images for: Predominance of the SARS-CoV-2 Lineage P.1 and Its Sublineage P.1.2 in Patients from the Metropolitan Region of Porto Alegre, Southern Brazil in March 2021
Source: Pathogens. 2021 Aug 5;10(8):988. doi: 10.3390/pathogens10080988 (PMC8402156; doi:10.3390/pathogens10080988)

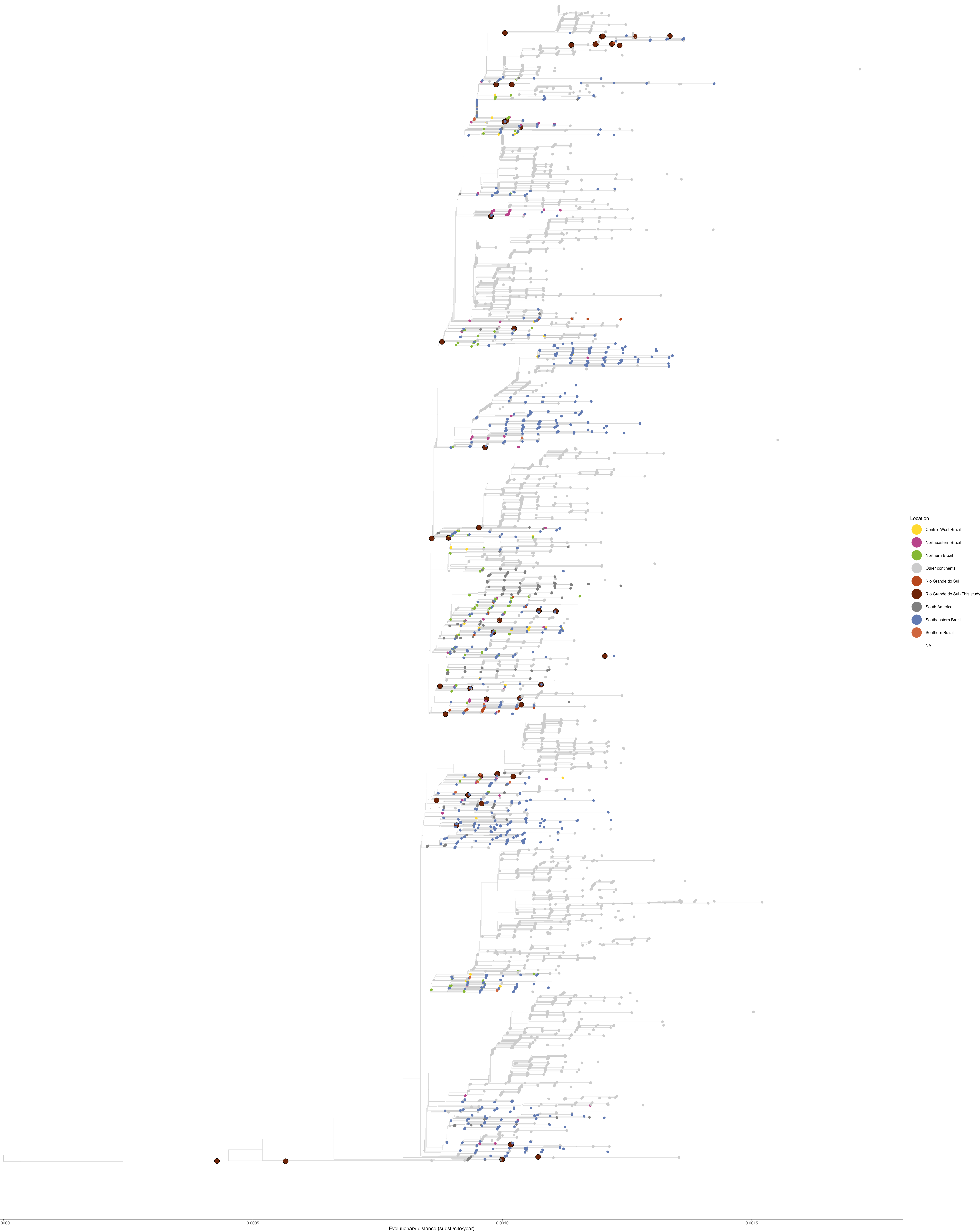

Supplement: Supplementary file 1 [file pathogens-10-00988-s001.zip › FigureS7.pdf]
