# Supplementary material for: Predominance of the SARS-CoV-2 Lineage P.1 and Its Sublineage P.1.2 in Patients from the Metropolitan Region of Porto Alegre, Southern Brazil in March 2021
Source: Pathogens. 2021 Aug 5;10(8):988. doi: 10.3390/pathogens10080988 (PMC8402156; doi:10.3390/pathogens10080988)
Supplement: Supplementary file 1 [file pathogens-10-00988-s001.zip › SupplementaryFileS1.zip!/SupplementaryFileS1/nextstrain/docs/_layouts/default.html]

{% seo %}


## {{ site.title | default: site.github.repository\_name }}

### Analysis

1. Setup and installation  
2. Preparing your data  
3. Orientation: analysis workflow  
4. Orientation: files  
5. Running & troubleshooting  
6. Customizing your analysis  
7. Customizing your visualization

### Visualization & interpretation

8. Options: visualizing and sharing  
9. Interpreting your results  
10. Writing a narrative

- Download **ZIP File**
- Download **TAR Ball**
- View On **GitHub**

#### Stuck? Ask us on the discussion board. We're happy to help!

This project is maintained by {{ site.github.owner\_name }}

{{ content }}
